# Profiling Ethylene-Responsive Genes Expressed in the Latex of the Mature Virgin Rubber Trees Using cDNA Microarray

**DOI:** 10.1371/journal.pone.0152039

**Published:** 2016-03-17

**Authors:** Zhiyi Nie, Guijuan Kang, Cuifang Duan, Yu Li, Longjun Dai, Rizhong Zeng

**Affiliations:** Key Laboratory of Biology and Genetic Resources of Rubber Tree, Ministry of Agriculture, Rubber Research Institute, Chinese Academy of Tropical Agricultural Sciences (CATAS), Danzhou, Hainan, China; National Taiwan University, TAIWAN

## Abstract

Ethylene is commonly used as a latex stimulant of *Hevea brasiliensis* by application of ethephon (chloro-2-ethylphosphonic acid); however, the molecular mechanism by which ethylene increases latex production is not clear. To better understand the effects of ethylene stimulation on the laticiferous cells of rubber trees, a latex expressed sequence tag (EST)-based complementary DNA microarray containing 2,973 unique genes (probes) was first developed and used to analyze the gene expression changes in the latex of the mature virgin rubber trees after ethephon treatment at three different time-points: 8, 24 and 48 h. Transcript levels of 163 genes were significantly altered with fold-change values ≥ 2 or ≤ –2 (*q*-value < 0.05) in ethephon-treated rubber trees compared with control trees. Of the 163 genes, 92 were up-regulated and 71 down-regulated. The microarray results were further confirmed using real-time quantitative reverse transcript-PCR for 20 selected genes. The 163 ethylene-responsive genes were involved in several biological processes including organic substance metabolism, cellular metabolism, primary metabolism, biosynthetic process, cellular response to stimulus and stress. The presented data suggest that the laticifer water circulation, production and scavenging of reactive oxygen species, sugar metabolism, and assembly and depolymerization of the latex actin cytoskeleton might play important roles in ethylene-induced increase of latex production. The results may provide useful insights into understanding the molecular mechanism underlying the effect of ethylene on latex metabolism of *H*. *brasiliensis*.

## Introduction

*Hevea brasiliensis* (para rubber tree) is so far recognized as the only species producing commercially viable quantities of high-quality natural rubber (NR) [1], i.e. *cis*-1, 4-polyisoprene [2]. NR is synthesized and stored in the rubber particle [3, 4], a special organelle suspended in the latex of the special laticiferous cells that form a ubiquitous network of tubes in the phloem of rubber trees [5]. The latex is actually the cytoplasm of *H*. *brasiliensis* laticifers, and contains 30–50% (w/w) of NR formed from sequential condensation of IPP (isopentenyl diphosphate) units [6]. The rubber biosynthesis of rubber trees uses the basic precursor of sucrose in the laticifer’s latex in a typical isoprenoid secondary metabolism [7], which is similar to the isoprenoid biosynthesis of other plant species using IPP as the precursor [8]. Many latex-expressed genes, such as HMGR (3-hydroxy-3-methylglutaryl-coenzyme A reductase), farnesyl diphosphate synthase, geranylgeranyl diphosphate synthase (GGPS), rubber elongation factor (REF), small rubber particle protein (SRPP) and *cis*-prenyltransferase (CPT) participate in the latex regeneration and rubber biosynthesis [9–16]; therefore, the gene expression profile and its kinetic change in *H*. *brasiliensis* laticifers is very important for investigating the related molecular events of latex metabolism and rubber biosynthesis [17].

A great deal of effort has been made to increase *H*. *brasiliensis* latex production and rubber yield. In the last half-century, various plant hormones and other chemicals were extensively tested to enhance latex production; and ethylene (ET), induced by application of ethephon (chloro-2-ethylphosphonic acid, a releaser of ET), was identified as the most efficient stimulant of latex production in *H*. *brasiliensis* [18]. Ethephon and ET gas are commonly applied as latex stimulants in rubber plantations worldwide. To better understand the physiological and molecular events by which ET increases latex yield, many studies have focused on the involvement of ET in latex regeneration and rubber biosynthesis of rubber trees [10, 19]. Two representative hypotheses have been suggested to demonstrate the roles of ET in rubber trees. One hypothesis is the enhancement of latex metabolic activity, mainly owing to the accelerated metabolic levels of sucrose and energy (ATP) in the laticiferous cells under ET stimulation [20–24]. Recent studies demonstrated that a group of latex sucrose transporters was shown to be responsible for the increased latex yield through importation of sucrose into the laticifers of rubber trees stimulated with ethephon [25–27]. The other hypothesis is prolongation of latex flow after bark tapping of the ethephon-treated rubber trees, mainly attributed to water circulation between laticifers and their surrounding tissues [28, 29]. More recently, the identified ET-responsive plasma membrane intrinsic protein (PIP) aquaporins of HbPIP2;1, HbTIP1;1 and HbPIP2;3 were found to favor prolongation of latex flow and are thus involved in ET-induced increase of latex production [30–32].

ET is a structurally simple gaseous hormone that regulates complex physiological processes of plants [33, 34]. Stimulation of rubber trees with ET is associated with marked changes in the physiology and biochemistry of the bark tissues especially the laticiferous cells, which not only induce an increase in latex production [35], but also cause some undesirable side-effects in rubber plantations. Evidence has accumulated that ET stimulation is a major factor leading to tapping panel dryness (TPD), a syndrome of no latex flow resulting in greatly decreased latex production [36–38]. However, little information is available concerning the effects of ET stimulation on induction of TPD.

Transcriptional regulation in ET response plays pivotal roles in plant physiological processes. Complementary DNA (cDNA) microarray analysis can simultaneously detect expression levels of thousands of genes [39], and is extensively used to examine plant growth and development processes, and responses to wounding and to phytohormones such as ET [40–42]. ET-responsive gene expression profiles in the laticiferous cells would orchestrate the physiological and biochemical changes that underlie the fundamental basis of the activated latex metabolism and the prolonged latex flow, and finally induce increased latex production. To monitor the comprehensive ET-responsive gene expression profile in *H*. *brasiliensis* laticifers, custom-designed cDNA microarrays developed from the latex expressed sequence tags (ESTs) were generated and used to analyze gene expression in the latex cells of rubber trees under ethephon stimulation. Most of the early ET-regulated genes are greatly implicated in disease and defense responses [34, 43] and consequently, in this research, three different time-points of 8, 24 and 48 h for ethephon treatment were selected to investigate the temporal cascade of the latex gene expression in response to ET, mainly focused on the transcriptional profiling of the latex ET-responsive genes that might potentially be involved in latex metabolism and rubber biosynthesis during longer periods of ET stimulation.

## Materials and Methods

### Plant materials and treatments

The rubber trees were cultivated at the experimental farm of the Chinese Academy of Tropical Agricultural Sciences (Danzhou, Hainan, China), and the study was approved by the experimental farm of the Chinese Academy of Tropical Agricultural Sciences. Field experiments were performed using mature, seven-year-old virgin (unexploited) rubber trees (Clone Reyan 7-33-97) that had never been tapped. Stimulation assays with exogenous ethephon were carried out according to the method previously described [44, 45]. Briefly, 0.5% (w/w) ethephon (Sigma-Aldrich, USA) in water was applied to the bark below the half-spiral of the tapping cut. Water was used as the mock stimulation for the control samples. The trunks of the treated trees were wrapped with black plastic film around the tapping cut and stimulated for 8, 24 or 48 h, after which they were tapped for latex early in the morning on the same day. Each sample included three independent biological replicates, and each replicate comprised the latex collected from six trees.

### Measurement of latex yield and physiological parameters

The latex yield and physiological parameters of the unexploited rubber trees were measured in October. To assay the physiological parameters, an aliquot of 20 ml of latex per tree was collected 5 min after tapping in a 50 ml centrifuge tube placed in ice, and transported to the laboratory for immediate analysis. Latex yield were measured as described by Tungngoen et al. [31]. The parameters of total solid content (TSC), thiol (RSH), and sucrose contents were determined according to Eschbach et al. [46]. Plugging index was measured according to Milford et al. [47]. The duration of latex flow was denoted that the duration from tapping to flow cessation.

### Latex total RNA extraction

Total RNAs were isolated from the latex collected at each time-point using our previously described method [44]. RNA quality and quantity were determined with a Nanodrop 2000 instrument (Thermo Scientific, Wilmington, DE, USA) and a Bioanalyzer Chip RNA 7500 series II (Agilent, Santa Clara, USA). For enrichment of ET-responsive genes, total latex RNAs from two groups of rubber trees at three different time-points were equally pooled, respectively, and used for cDNA library construction.

### Latex cDNA library construction and EST analysis

To enrich the ET-responsive genes expressed in the latex of rubber trees, total latex RNAs prepared from the latexes of the ethephon-treated rubber trees and the control samples at the three time-points of 8, 24 and 48 h were equally pooled, and used for the latex cDNA library construction [48]. Before cDNA synthesis, the pooled RNA preparations were incubated for 20 min at 37°C with 10 units of RNase-free DNase I (Roche Applied Science, USA) to remove residual genomic DNA. The cDNA synthesis and library construction were performed as previously described [48], using pBluescript II cDNA library construction kit (Stratagene, USA).

A total of 12,000 colonies were randomly selected, and plasmid DNAs were isolated and preserved in 96-well plates. The template DNAs for sequencing were amplified using the TemphliPhi DNA sequencing template amplification kit (GE Healthcare, USA). PCR products were purified using AxyPrep^™^ PCR cleanup kit (Axygen, USA), and subjected to single-pass sequencing from the 5ʹ-end on an ABI 3730 DNA sequencer (Applied Biosystems, Foster City, CA, USA) using BigDye terminator cycle sequencing kit (Applied Biosystems, USA).

The EST sequences were edited manually to remove the vector contaminations, short sequences (< 100 bp) and sequences containing > 1.5% of unreadable nucleotides. All edited sequences were clustered using TGICL software [49], and then assembled using CAP3 with default settings [50]. All unigenes were annotated by comparison with the NCBI non-redundant protein sequences (nr) database (http://www.ncbi.nlm.nih.gov/) with the blastx algorithm using an *E*-value cut-off of 10^−5^. The latex unigenes were verified by searching the NCBI transcriptome shotgun assembly sequence database (TSA) and *H*. *brasiliensis* genome database that was recently sequenced and assembled by the Rubber Research Institute of China. GO (gene ontology) annotation and the GO functional classifications of these unigenes were performed using the Blast2GO program with an *E*-value threshold of 10^−3^ [51].

### Latex cDNA microarray construction

A *Hevea* latex cDNA microarray was constructed in-house at CapitalBio Corporation (Beijing, China) using amplified cDNAs from the latex cDNA library. Briefly, 2973 unique sequences from the latex cDNA library were selected, and the corresponding cDNA clones were amplified by PCR using T3 and T7 primers. The amplified cDNA products were then purified, and spotted onto amino silaned glass slides using a SmartArray microarrayer (CapitalBio, Beijing, China). On one slide, there were 48 blocks and each block had 18 rows and 16 columns. In addition to the positive controls, negative controls and exogenous controls, five latex-expressed house-keeping genes were used as reference or control genes: actin (GenBank acc. No.: JF775488), 18S rRNA (GenBank acc. No.: AB268099), GAPDH (GenBank acc. No.: KR905466), α-tubulin (GenBank acc. No.: KC333454) and β-tubulin (GenBank acc. No.: JT928408). The primers for amplification were as follows: actin (forward 5ʹ-CAG TGG TCG TAC AAC TGG TAT-3ʹ, reverse 5ʹ-ATC CTC CAA TCC ATA CAC TGT-3ʹ), 18S rRNA (forward 5ʹ-GGT CGC AAG GCT GAA ACT-3ʹ, reverse 5ʹ-ACG GGC GGT GTG TAC AAA-3ʹ), GAPDH (forward 5ʹ-GAA GAT GAT GTG GTT TCC AGT G-3ʹ, reverse 5ʹ-CAC CCA TAG AAG GCA AGG AT-3ʹ), α-tubulin (forward 5ʹ-TGG TGA AGG CAT GGA AGA AG-3ʹ, reverse 5ʹ- TCA TGA AAA TGA CAA ACT CTT-3ʹ) and β-tubulin (forward 5ʹ- AAA GAT GAG CAC CAA GGA AGT T-3ʹ, reverse 5ʹ- GTT CTT ATG CTC CTG GTG AAG TT-3ʹ).

### RNA amplification and labeling

In the assays of microarray hybridization, total RNAs from the latexes of ethephon-treated rubber trees at three time-points of 8, 24 and 48 h were used as three ethephon-treated samples; total RNAs from the latexes of the control rubber trees collected at corresponding three time-points were the three control samples. Each experimental sample was divided into three biological replicates in which total latex RNAs from six random rubber trees were equally pooled. Double-stranded cDNAs containing T7 RNA polymerase promoter sequence were synthesized with 5 μg of total latex RNA using reverse transcription system, RNase H, DNase I and T4 DNA polymerase according to the manufacturer’s protocol (Promega, USA). The cDNAs were then labeled with fluorescent dyes of Cy5- and Cy3-dCTP (GE Healthcare, USA) as previously described [52–54]. The resulting labeled DNAs were quantitatively adjusted based on the efficiency of Cy5- or Cy3-dCTP incorporation and dissolved in 80 μL of hybridization solution (3 × SSC, 0.2% SDS, 25% formamide and 5 × Denhart’s).

### Probe hybridization

Probe hybridization with the generated latex cDNA microarrays was performed in a BioMixer^™^ II hybridization station (CapitalBio, Beijing, China) as previously described [53, 54]. The microarray slides were hybridized with labeled DNAs prepared from three biological replicate samples for the control or ethephon-treated rubber trees. To minimize the error due to fluor-associated bias, one reciprocal (dye-swap) experiment was carried out for each of the biological samples so that a total of six data points were available for every unigene on the microarray slides. The microarray slides were hybridized overnight at 42°C, and then washed with two consecutive washing solutions (0.2% SDS, 2 × SSC at 42°C for 5 min and 0.2% SSC for 5 min at room temperature).

### Image acquisition and data analysis

After probe hybridization, microarrays were scanned with a confocal LuxScan^™^ scanner (CapitalBio, Beijing, Chian), and the data for the obtained images were extracted with SpotData software (CapitalBio, Beijing, China). Normalization and analysis of microarray data were performed using LuxScan 3.0 software (CapitalBio, Beijing, China). Faint spots with intensities < 400 units after background subtraction in both Cy3 and Cy5 channels were removed for individual channel data extraction. A space- and intensity-dependent normalization was performed using the LOWESS program [55]. The differentially expressed genes were determined and analyzed using a *t*-test method and SAM software [56]. Significantly up- or down-regulated genes were filtered with fold-change values ≥ 2 or ≤ –2, respectively, in at least one time-point with *q-*value ≤ 0.05 in *t*-test. The Treeview program was used to visualize the cluster data of the differentially expressed genes [57]. The microarray dataset has been submitted to the Gene Expression Omnibus database with an accession number of GSE74060.

### Validation of gene expression by real-time quantitative reverse transcript-PCR (RT-qPCR)

RT-qPCR analysis of 20 genes selected was performed as previously described [45, 58]. Briefly, total RNA from *H*. *brasiliensis* latex was isolated using an RNeasy plant mini-kit (Qiagen, USA) according to the manufacturer’s instructions. Reverse transcription was performed with SuperScript III reverse transcriptase (Invitrogen, USA), followed by incubation with RNase H (Invitrogen, USA). Primers were designed for the selected genes with Vector NTI software (Invitrogen, USA) that provided a PCR product of ≈200 bp. The forward and reverse primer sequences used to detect each mRNA and their efficiencies are shown in S1 Table. The cDNAs were synthesized by reverse transcriptase, and quantitative gene expression analysis was carried out by RT-qPCR using a LightCycler 2.0 (Roche, Basel, Switzerland) and the following parameters: 30 s at 94°C for denaturation, followed by 45 cycles of 94°C for 5 s, 60°C for 20 s and 72°C for 20 s. Each RT-qPCR reaction was replicated three times. For normalization purposes, according to Tang et al. [27] and Li et al. [38], the *H*. *brasiliensis* 18S rRNA gene was used as the internal reference for all RT-qPCR analyses.

## Results

### Latex yields and physiological parameters

**Fig 1:**
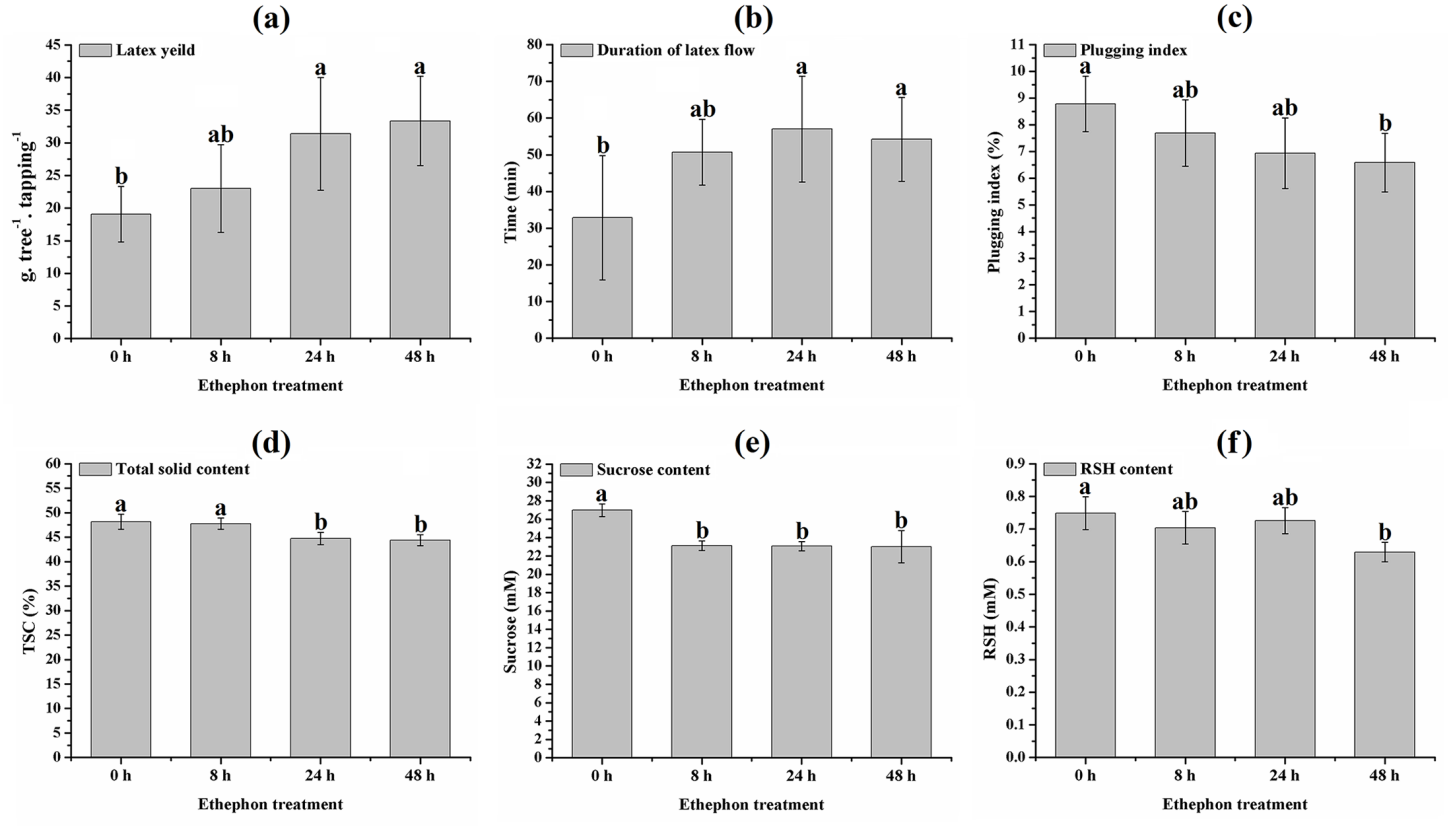
Kinetics of latex yeild (a), duration of latex flow (b), plugging index (c), TSC (d), sucrose content (e) and RSH content (f) in the latex of the mature virgin rubber trees treated with ethephon. The latex yeild and physiological parameters i.e., duration of latex flow, TSC, plugging index, sucrose content and RSH content were measured at 0, 8, 24 and 48 h after ethephon treatment. The values were shown as the means ± standard deviation (n = 3) for each including six trees. One-way ANOVA was performed using SPSS 19.0 software. The Student–Newman–Keuls test was used for multiple comparisons testing to investigate the significant differences between groups. Bars with different letters were significantly different at the *p* < 0.05 level.

To get a better understanding about the effects of ethephon stimulation on the latex production of unexploited rubber trees, the latex yeild and physiological parameters i.e., duration of latex flow, TSC, plugging index, contents of sucrose and RSH were firstly measured. Significant increases in the latex yield and the duration of latex flow were observed from unexploited rubber trees stimulated with ethephon for 24 h (Fig 1a and 1b), and remarkable decreases in the plugging index and the TSC were detected from unexploited rubber trees treated with ethephon after 24 h and 48 h (Fig 1c and 1d). The contents of the latex sucrose and the RSH greatly decreased in the latex of the unexploited rubber trees stimulated with ethephon for 8 h and 48 h, respectively (Fig 1e and 1f). In this research, no remarkable alterations of the the latex physiological parameters were observed in the latex of the unexploited rubber trees stimulated with ethephon only for 1 h or 4 h (S1 Fig).

### *H*. *brasiliensis* latex cDNA library construction and ESTs analysis

**Fig 2:**
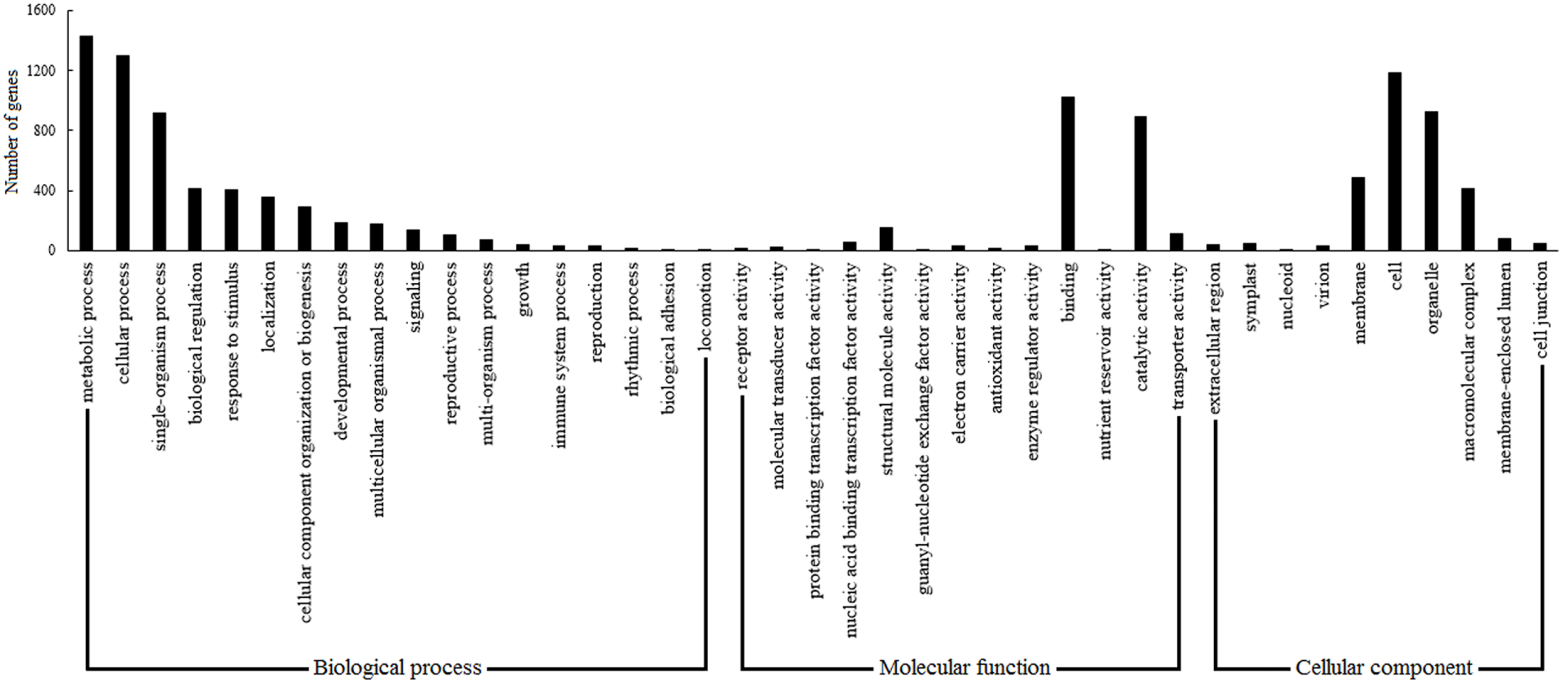
GO classification of the probes used in the latex cDNA microarray. The 1689 unigenes (probes) with significant similarity in nr protein databases were assigned to GO classifications.

The latex cDNA microarray was developed from the cDNAs of the latex-expressed genes in *H*. *brasiliensis* laticifers. The latex cDNA library construction and EST sequencing were the first step for the preparation of the cDNA microarray. A total of 12,000 colonies were randomly selected and sequenced from the 5ʹ-terminus, resulting in 11,838 latex ESTs after quality editing and vector sequence removing. The 11,838 latex ESTs were then clustered and assembled into 2973 unigenes, among which 4.5% (133) had no reported homologs or showed homology to genes coding for predicted proteins (*E*-value < 1.0 E^-5^) as analyzed by the blastx program against the nr database (S2 Table). In total, 1689 unigenes were assigned to GO classes with 11,513 functional terms. As shown in Fig 2, the assignments to ‘biological process’ were the majority (5907, 51.3%), followed by ‘cellular component’ (3238, 28.1%) and ‘molecular function’ (2368, 20.6%). Under the category ‘biological process’, ‘metabolic process’ (1426, 24.1%) and ‘cellular process’ (1297, 22.0%) were prominently represented. Under the classification ‘cellular component’, ‘cell’ (1181, 36.5%) and ‘organelle’ (925, 28.6%) were separately the first and second largest categories. Two categories, ‘binding’ and ‘catalytic activity’, were approximately 81.0% of ‘molecular function’.

### Preparation of latex cDNA microarray

The entire set of cDNAs from the 2973 latex unigenes were amplified by RT-PCR, and printed on amino silane glass slides to construct the latex cDNA microarray, which contained 48 blocks and each block had 18 rows and 16 columns. All information concerning the cDNA sequence data on the microarray is available in S3 Table.

### Identification of ET-regulated genes in *H*. *brasiliensis* latex

**Fig 3:**
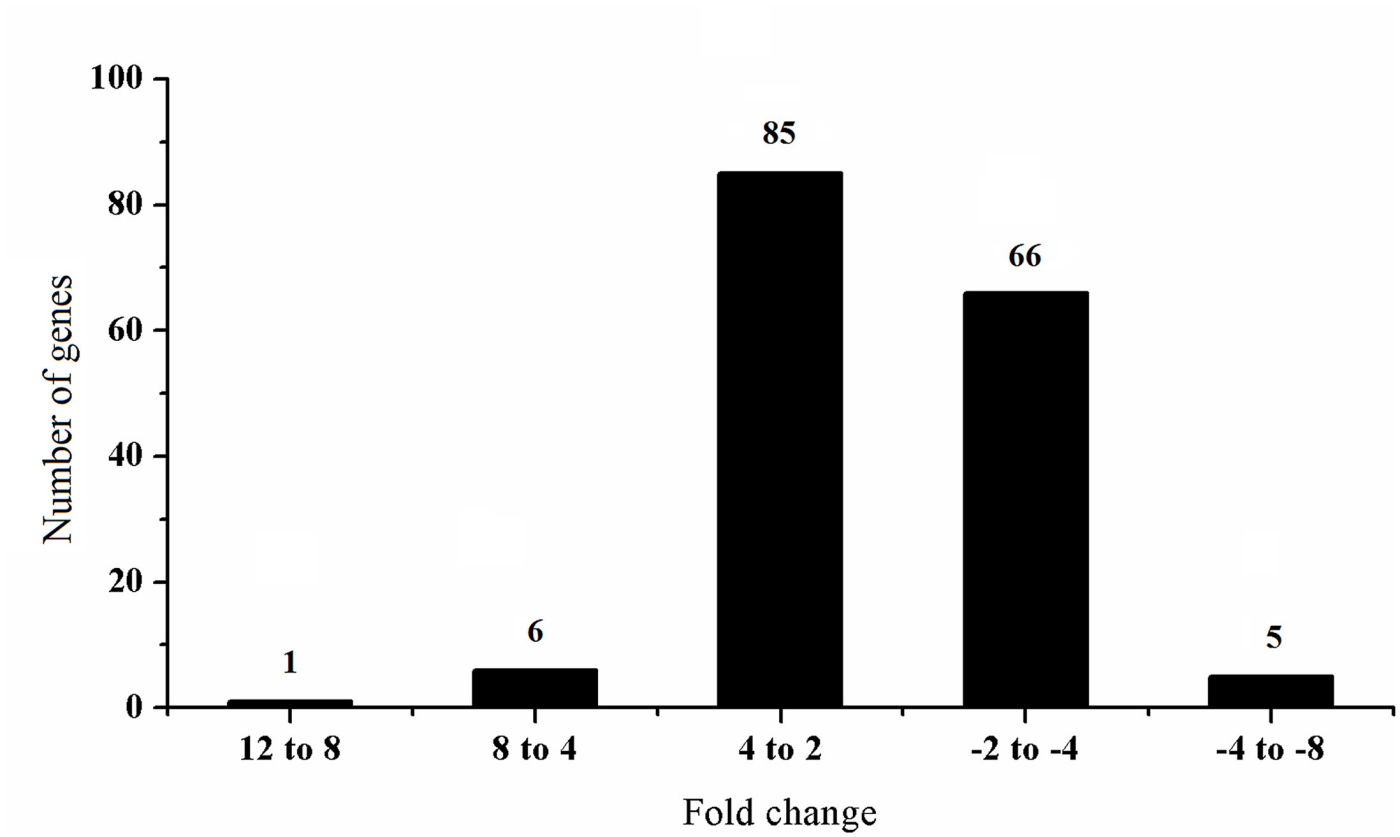
Distribution of the latex genes significantly regulated by ET stimulation in rubber trees. The fold-changes of gene expression were analyzed by cDNA microarray. The positive and negative numbers on the x-axis represented up- and down-regulation of latex genes, respectively.

**Fig 4:**
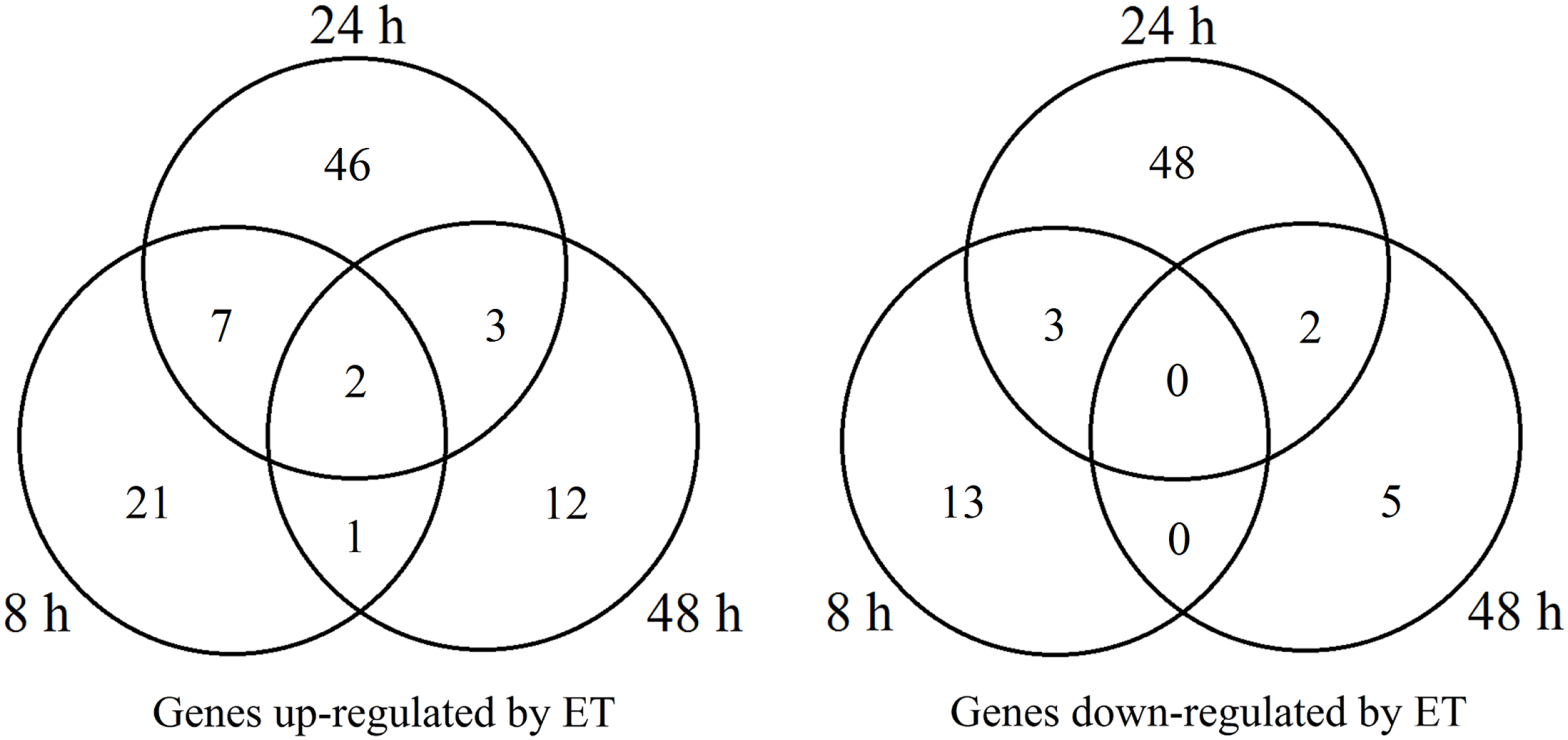
Venn diagram analysis for the ET-responsive genes under the different times of ET treatment. Overlapping circles visually represented the commonalities among sets of information. The number of differentially expressed genes shared by different time-points (8, 24 and 48 h) under ET treatment was displayed in the overlapping circles.

**Fig 5:**
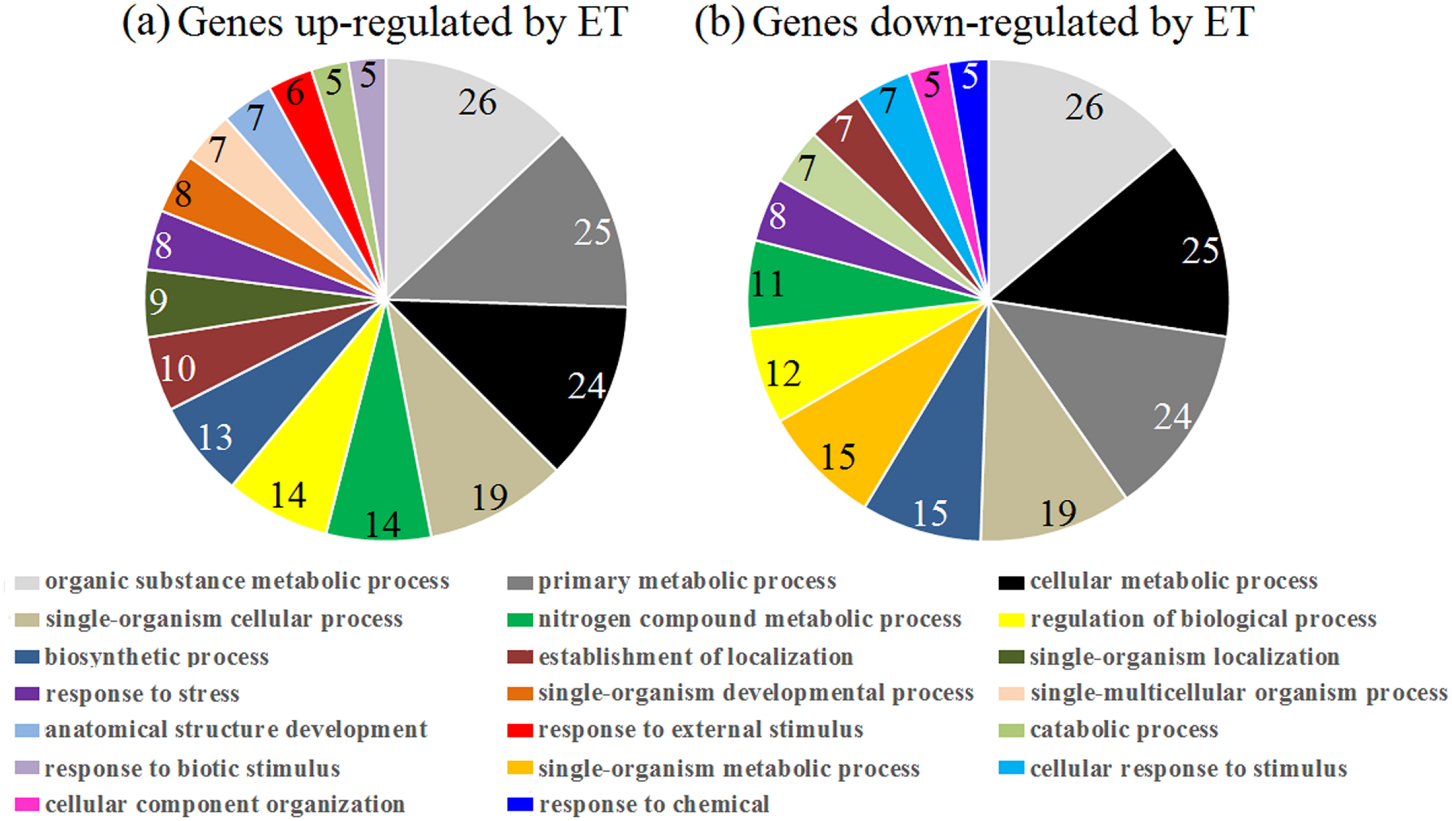
GO terms belonging to the biological process domain attributed to ET-responsive genes in the latex. (a) GO term classification of ET-responsive genes with fold-change values ≥ 2 (*q-*value < 0.05) (b) GO term classification of ET-responsive genes with fold-change values ≤ –2 (*q-*value < 0.05). GO term classifications populated by at least five genes were shown, and the number of genes annotated within each term was indicated.

This study was mainly focused on profiling of the latex ET-responsive genes in the ethephon-treated unexploited rubber trees, i.e., 8 h, 24 h and 48 h post-stimulation. Transcript levels of 163 latex genes were significantly altered with fold-change values ≥ 2 or ≤ –2 (*q-*value < 0.05) in ethephon-treated compared to control rubber trees (Fig 3, Tables 1 and 2). Of these 163 genes, 92 were up-regulated (Table 1) and 71 down-regulated (Table 2). After the trees were stimulated by ethephon, 31, 58 and 18 genes were up-regulated (fold-change values ≥ 2, *q-*value < 0.05) and 16, 53 and 7 genes were down-regulated (fold-change values ≤ –2, *q-*value < 0.05) at 8, 24 and 48 h post-treatment, respectively (Fig 4). Among the 163 ET-induced genes, 160 had reported homologs or showed homology to genes coding for predicted proteins (*E*-value < 1.0 E^–5^) using the blastx program against the nr database, and 92 ET-induced genes with blast matches to known proteins were assigned to GO classes. The most frequent GO terms belonging to the ‘biological process’ domains are depicted in Fig 5. Several of these terms were prominently represented, such as ‘organic substance metabolic process’, ‘primary metabolic process’, ‘cellular metabolic process’, ‘single-organism cellular process’, ‘biosynthetic process’ and ‘regulation of biological process’, indicating that some important metabolic activities and cell processes were induced by ethephon in rubber tree laticifers. Among the latex ET-induced genes that matched known proteins, a PIP gene, an AP2/ethylene response factor (ERF) super family gene and a bidirectional sugar transporter (SWEET) gene were strongly up-regulated; and a Cu/Zn superoxide dismutase (Cu/Zn SOD) gene, a cytochrome c oxidase subunit 5b-like protein (COX5B) gene and two formin-like protein gene were strongly down-regulated. This all suggested that laticifer water circulation, production and scavenging of reactive oxygen species (ROS), sugar metabolism and regulation of actin cytoskeleton assembly might play important roles in ET-induced increase of latex production in rubber trees. In order to present an overall understanding of the ethylene-mediated responses in the laticifers, the differentially expressed genes from an earlier ethephon treatment (1 h and 4 h) were provided as S4 and S5 Tables, because much fewer genes were identified with fold-change values ≥ 2 or ≤ –2 after ethephon treatment only for 1 h and 4 h.

**Table 1:** Up-regulated genes under ethephon stimulation through cDNA microarray analysis.

Gene ID	*H*.*brasiliensis* homolog	Description	Fold Change			*q-*value
			8 h	24 h	48 h	
L0012	JT960862.1	protein RALF-like 34	1.72	2.38	1.93	0.00
L0025	JT949537.1	methylesterase 10-like	1.73	4.04	1.14	0.00
L0036	JT955983.1	HSP20-like chaperones superfamily protein	2.38	2.67	1.12	0.00
L0043	JR350842.1	conserved hypothetical protein	1.14	2.01	1.30	0.00
L0046	JT930900.1	fatty acyl- reductase 3-like	3.40	1.67	0.91	0.00
L0051	JT957340.1	diphthamide biosynthesis protein 3-like	1.32	2.27	0.92	0.00
L0055	JT924152.1	NAC domain-containing protein 78	3.27	1.76	0.98	0.00
L0092	JT953466.1	hypothetical protein JCGZ_12701	3.10	1.76	1.53	0.00
L0094	JT973817.1	DNAJ homolog subfamily b member 6-like	1.00	1.13	2.40	0.00
L0097	JT934437.1	ARM repeat superfamily protein isoform 1	1.70	2.09	1.12	0.00
L0129	JT946385.1	hypothetical protein JCGZ_22853	2.21	2.47	1.00	0.01
L0154	JR364176.1	maturation-associated SRC1-like protein*	1.07	1.30	2.55	0.00
L0200		AP2/ERF super family protein	1.57	2.28	1.21	0.00
L0296	JT939262.1	RING finger protein 214 isoform 1	1.48	2.32	1.01	0.00
L0335	JT977880.1	Stem-specific protein TSJT1	1.24	2.40	1.90	0.00
L0366	JR348559.1	methionine aminopeptidase 1a	1.29	2.14	1.05	0.00
L0381	JT924607.1	E3 ubiquitin-protein ligase RLIM-like	1.42	2.31	1.04	0.00
L0383	JT932391.1	probable inactive poly[ADP-ribose] polymerase SRO3	1.40	3.05	1.38	0.00
L0400	JT961349.1	ubiquitin carboxyl-terminal hydrolase 12-like	2.18	0.75	0.93	0.00
L0407	JT945196.1	exonuclease chloroplastic mitochondrial	1.70	2.96	1.41	0.00
L0410	JR348033.1	protein light-dependent short hypocotyls 4	2.41	1.23	1.25	0.00
L0437	JT958324.1	heavy metal-associated isoprenylated plant protein 26-like	1.53	2.96	1.13	0.00
L0447	JT950737.1	protein n-terminal glutamine amidohydrolase	1.38	2.24	1.48	0.00
L0459	JT930276.1	mitochondrial metalloendopeptidase oma1	1.26	2.23	2.38	0.00
L0528	JT963312.1	profilin 3 family protein	1.39	4.75	2.79	0.00
L0542	JT962677.1	hypothetical protein POPTR_0018s06230g	2.01	1.46	1.10	0.00
L0570	JT971674.1	acetyltransferase at1g77540-like	2.15	2.29	1.39	0.00
L0587	JT938546.1	GTP binding protein, putative	2.05	1.39	0.97	0.00
L0657	JT941656.1	lipid phosphate phosphatase chloroplastic	0.87	2.02	1.69	0.00
L0740	JR344506.1	interferon-induced GTP-binding protein	2.11	1.84	1.38	0.00
L0793	JT951926.1	HSP40 cysteine-rich domain superfamily protein	1.37	2.72	1.13	0.00
L0814	JT953136.1	latex allergen Hev b 5*	0.98	1.01	2.27	0.00
L0954	JT952968.1	sterol carrier	1.10	2.40	1.37	0.00
L0965	JT961102.1	zinc finger a20 and an1 domain-containing stress-associated protein 4	1.49	2.04	1.09	0.00
L0990	JT937737.1	nodulin family protein	2.35	2.07	1.24	0.00
L1016	JT946494.1	uridylate kinase	2.75	1.66	1.23	0.00
L1026	JT963696.1	hypothetical protein JCGZ_17913	1.02	2.81	1.45	0.00
L1029	JT957910.1	vacuolar protein sorting-associated protein 25	1.25	2.07	1.20	0.00
L1068	JT953517.1	dessication responsive protein	1.45	2.45	1.55	0.00
L1127	JR362827.1	ethylene-responsive element binding protein 2	1.92	2.48	1.19	0.00
L1173	JT945540.1	cytochrome P450 704c1-like	2.04	0.55	0.69	0.00
L1185	JT962615.1	x-linked retinitis pigmentosa GTPase regulator-interacting protein 1-like	2.61	1.67	0.84	0.00
L1186	JR364404.1	cysteine proteinase inhibitor	1.05	2.45	1.86	0.00
L1343	JR366829.1	sucrose transporter 1	1.61	2.21	1.27	0.00
L1370	JT942690.1	1-aminocyclopropane-1-carboxylate deaminase	1.18	0.96	2.01	0.00
L1401	JT980862.1	conserved hypothetical protein	0.91	2.18	1.02	0.00
L1428	JT951761.1	DOF zinc finger protein	0.58	2.08	1.47	0.00
L1433	JT944001.1	fatty acid 2-hydroxylase 1-like	1.50	3.52	0.95	0.00
L1499	JT936619.1	bidirectional sugar transporter SWEET10-like	1.33	4.90	0.59	0.00
L1504	JT962614.1	hypothetical protein JCGZ_11378	1.14	2.07	1.23	0.00
L1643	JT944734.1	hypothetical protein JCGZ_04721	2.43	1.78	1.28	0.00
L1654	JT947142.1	conserved hypothetical protein	1.15	2.10	1.59	0.00
L1678	JT951441.1	18.2 kDa class I heat shock protein	2.61	0.93	1.30	0.00
L1680	JT948897.1	histone H1	1.14	1.72	2.12	0.00
L1716	JT974820.1	hypothetical protein JCGZ_06201	1.42	2.31	1.02	0.00
L1736	JT939515.1	protein BPS1, chloroplastic-like	2.04	1.84	1.88	0.00
L1775	JT942366.1	ubiquitin	1.05	2.02	0.98	0.00
L1783	JT969215.1	conserved hypothetical protein	1.05	2.86	1.43	0.00
L1901	JR364493.1	NAC domain-containing protein 2	1.73	4.57	2.04	0.00
L1911	JR344767.1	ETHYLENE-INSENSITIVE3 protein	2.11	1.72	1.31	0.00
L1912	JT950758.1	hypothetical protein JCGZ_11253	1.85	1.85	3.25	0.00
L1921	JT945335.1	18.2 kDa class I heat shock family protein	2.14	1.04	1.32	0.00
L1986	JT924225.1	GRAS family transcription factor (GRAS59)	2.13	1.86	2.07	0.00
L2033	JT934826.1	phosphatase 2c family protein	1.62	2.07	1.01	0.00
L2117	JT966645.1	fiber protein fb11	1.55	2.54	1.58	0.00
L2145	JT942483.1	plasma membrane intrinsic protein PIP2;3	5.13	2.69	1.53	0.00
L2170	JR346155.1	triacylglycerol lipase	2.63	0.73	1.05	0.00
L2174	JT927768.1	PREDICTED: uncharacterized protein LOC104905041	2.94	1.74	1.14	0.00
L2223	JT942604.1	hypothetical protein JCGZ_04721	3.48	2.16	1.08	0.00
L2261	JT957175.1	PREDICTED: uncharacterized protein LOC103953524	1.08	2.53	1.32	0.00
L2300	JT927700.1	allene oxide synthase	2.57	1.48	1.35	0.00
L2303	JR357022.1	glutaredoxin-c6-like	1.48	1.55	2.30	0.01
L2305	JT940465.1	ubiquitin	1.28	2.46	1.03	0.00
L2343	JR347496.1	pyruvate dehydrogenase family protein	1.12	2.95	1.30	0.00
L2359	JT941742.1	zinc finger A20 and AN1 domain-containing stress-associated protein 7	1.48	2.52	1.50	0.00
L2360	JT972872.1	protease inhibitor protein 1 (PI1)	1.44	2.10	1.19	0.00
L2390	JT951309.1	guanine nucleotide-binding protein subunit gamma 2-like	0.81	2.51	1.02	0.00
L2412	JR364234.1	chaperone protein DNAJ-related	1.15	2.17	1.16	0.00
L2452	JR363991.1	ecotropic viral integration site 5 protein	1.42	2.71	1.96	0.00
L2477	JT945193.1	dehydrin protein	1.39	1.31	2.22	0.00
L2487	JR366057.1	AP2/ERF super family protein	2.14	5.01	7.40	0.00
L2497	JT935376.1	AP2/ERF super family protein	1.15	1.44	2.38	0.00
L2522	JR355065.1	conserved hypothetical protein	7.15	8.27	2.78	0.00
L2648	JT962967.1	hypothetical protein JCGZ_10888	2.02	1.30	0.79	0.00
L2649	JT959274.1	profilin 5	1.14	2.11	1.38	0.00
L2677	JR359565.1	23.6 kDa heat shock, mitochondrial -like protein	1.68	2.06	1.45	0.00
L2682	JT957840.1	F-box/LRR-repeat protein at3g26922-like	1.07	2.07	1.91	0.00
L2721		SAUR family protein	2.22	1.53	1.05	0.00
L2723	JT951227.1	AP2/ERF super family protein	1.84	1.16	2.75	0.00
L2773	JR365113.1	gata zinc finger domain-containing protein	1.29	1.58	2.02	0.00
L2914	JT952575.1	probable ribose-5-phosphate isomerase 2	2.13	2.93	1.13	0.00
L2924	JR366554.1	fasciclin-like arabinogalactan protein 8	0.84	1.95	2.09	0.00

*The unigene was annotated by comparison with the NCBI Non-redundant protein sequences (nr) database (http://www.ncbi.nlm.nih.gov/) with the BlastN algorithm using an *E*-value cut-off of 10^−5^.

**Table 2:** Down-regulated genes under ethephon stimulation through cDNA microarray analysis.

Gene ID	*H*.*brasiliensis* homolog	Description	Fold Change			*q-*value
			8 h	24 h	48 h	
L0013	JT916877.1	enolase-phosphatase e1-like	1.00	0.50	0.97	0.01
L0026	JT929934.1	F-box protein skip16	0.97	0.49	0.80	0.00
L0031	JT941097.1	U-box domain-containing protein 3	1.00	0.44	0.62	0.00
L0060	JT940201.1	fasciclin-like arabinogalactan protein 7	0.27	0.62	1.75	0.00
L0104	JT962900.1	acyl-CoA-binding protein	1.06	0.47	0.58	0.00
L0112	JT939599.1	endonuclease V	1.17	0.48	0.86	0.00
L0118	JT949014.1	cytochrome c oxidase subunit 5b-like	0.42	0.18	0.73	0.00
L0140	JT932367.1	proton-coupled amino acid transporter 3-like	0.56	0.28	0.69	0.00
L0203	JT950602.1	elicitor-responsive protein 1-like	1.01	0.46	0.89	0.00
L0208	JT927484.1	B2 protein	0.83	0.36	0.77	0.00
L0236		casbene chloroplast	0.97	0.33	0.77	0.00
L0246	JT936642.1	NAC domain-containing protein 8-like	0.74	0.44	0.69	0.00
L0283	JT915455.1	copia protein	0.99	1.11	0.46	0.02
L0291		cis-prenyltransferase	0.99	0.41	0.79	0.00
L0331	JT942435.1	calcium-binding ef hand family protein	0.35	1.02	0.80	0.00
L0380	JT945417.1	zinc finger family protein	0.76	0.50	0.46	0.00
L0433	JT971783.1	ubiquitin extension protein 1	0.64	0.35	0.57	0.00
L0442	JT914930.1	kinase family protein	1.30	0.46	0.78	0.00
L0448	JT935206.1	biotin protein ligase	0.64	0.54	0.47	0.00
L0457	JR365945.1	ethylene-inducible protein (ER1)	0.50	0.56	0.66	0.00
L0506	JT942847.1	caffeic acid o-methyltransferase	0.88	0.46	1.11	0.00
L0686	JR347987.1	selenoprotein h-like	0.85	0.49	0.81	0.00
L0698	JT926734.1	aspartic proteinase precursor	0.64	0.47	0.65	0.00
L0792	JT924112.1	probable alpha-amylase 2	0.85	0.49	0.61	0.00
L0799	JT944013.1	gibberellin receptor	0.39	0.95	0.85	0.00
L0801	JT942867.1	DUF593-containing protein	0.52	0.46	0.74	0.00
L0807	JT924572.1	inositol transporter 4 like protein	1.05	0.44	1.26	0.02
L0815	JT943548.1	DNAJ chaperone c-terminal domain-containing family protein	0.84	0.40	0.54	0.00
L0838	JT916724.1	autophagy-related protein 18f-like	0.58	0.45	0.69	0.00
L0891	JT966473.1	dynein light chain cytoplasmic-like	0.44	1.75	1.05	0.00
L0960	JT945149.1	two-component response regulator arr5-like	0.42	0.81	1.42	0.00
L0994	JT945230.1	conserved hypothetical protein	1.06	0.35	0.94	0.00
L1049	JT945364.1	glutamine amido-transferase YLR126C	1.40	0.49	0.67	0.00
L1170	JR360041.1	LOB domain-containing protein 37-like	1.05	0.43	0.83	0.00
L1198	JT950118.1	conserved hypothetical protein	1.03	0.49	0.78	0.00
L1227	JR345627.1	hypothetical protein JCGZ_08192	0.23	0.91	1.66	0.00
L1233	JT962766.1	Cu/Zn superoxide dismutase	1.06	0.32	0.51	0.00
L1330	JT936894.1	myosin phosphatase rho-interacting	1.14	0.44	0.97	0.00
L1525	JT915944.1	multiple C2 and transmembrane domain-containing protein 1-like	0.98	0.38	0.89	0.00
L1579	JT960330.1	epidermal patterning factor-like protein 3	0.72	0.49	1.03	0.00
L1607	JT940720.1	endonuclease or glycosyl hydrolase	0.73	0.42	0.76	0.00
L1645	JT925274.1	UDP-D-glucuronate 4-epimerase 6	0.37	0.55	0.79	0.00
L1756	JT950231.1	hypothetical protein POPTR_0002s08440g	0.70	0.48	0.60	0.00
L1809	JT941974.1	formin-like protein 18	0.25	0.62	0.94	0.00
L1810	JR366157.1	probable phospholipid hydroperoxide glutathione peroxidase	0.79	0.48	0.81	0.00
L1831	JT931867.1	SOUL heme-binding family protein	1.22	0.41	1.04	0.00
L1845	JT915709.1	structural constituent of nuclear pore, putative	0.84	0.43	0.63	0.00
L1853	JT953751.1	SAUR family protein (SAUR23)	0.43	0.59	1.54	0.00
L1890	JT933643.1	hypothetical protein JCGZ_17861	0.67	0.34	0.54	0.00
L1906	JT966408.1	protein RALF-like 24	0.49	0.73	0.77	0.00
L1909	JT919017.1	mannan endo-1, 4-beta-mannosidase 7-like	0.63	0.47	0.74	0.00
L1914	JT958801.1	NUC-1 negative regulatory protein	0.80	0.37	0.96	0.00
L1918	JT937667.1	NAC domain-containing protein	0.44	0.42	0.79	0.00
L1925	JT944694.1	plasma membrane intrinsic protein isoform 1	0.87	0.58	0.42	0.00
L2068	JT955937.1	thioredoxin h	1.30	0.49	0.72	0.00
L2116	JT962238.1	mitochondrial import inner membrane translocase subunit TIM13-like	1.17	0.93	0.44	0.00
L2188	JT925241.1	cysteine desulfurylase	1.09	0.39	0.64	0.00
L2191	JT929780.1	probable WRKY transcription factor 21	0.35	0.29	0.69	0.00
L2279	JR366494.1	basic leucine zipper 9-like	1.12	0.46	0.90	0.00
L2396	JT948911.1	short-chain dehydrogenase/reductase	1.24	0.23	0.61	0.00
L2471	JT959931.1	formin-like protein 4	1.01	0.28	0.45	0.00
L2527	JT948191.1	late embryogenesis abundant hydroxyproline-rich glycoprotein	1.16	0.47	0.81	0.00
L2557	JT951143.1	conserved hypothetical protein	1.27	0.37	0.88	0.00
L2691	JT928560.1	C2 domain-containing family protein	0.63	0.49	0.80	0.00
L2712	JT963599.1	MLP-like protein 329	1.10	0.86	0.47	0.00
L2724	JT942002.1	MAP 7 domain-containing protein 1-like	0.93	0.47	0.78	0.00
L2735	JT928745.1	ribosomal protein L16	0.52	0.37	0.60	0.00
L2759	JR349544.1	conserved hypothetical protein 12	0.47	0.61	0.70	0.00
L2760	JT935810.1	hypothetical protein JCGZ_18332	0.34	0.54	0.72	0.00
L2804	JT952582.1	RNA exonuclease 3	0.87	0.42	0.82	0.00
L2817	JT956946.1	peroxiredoxin family protein	1.17	0.44	0.72	0.00

### Validation of gene expression by RT-qPCR

**Fig 6:**
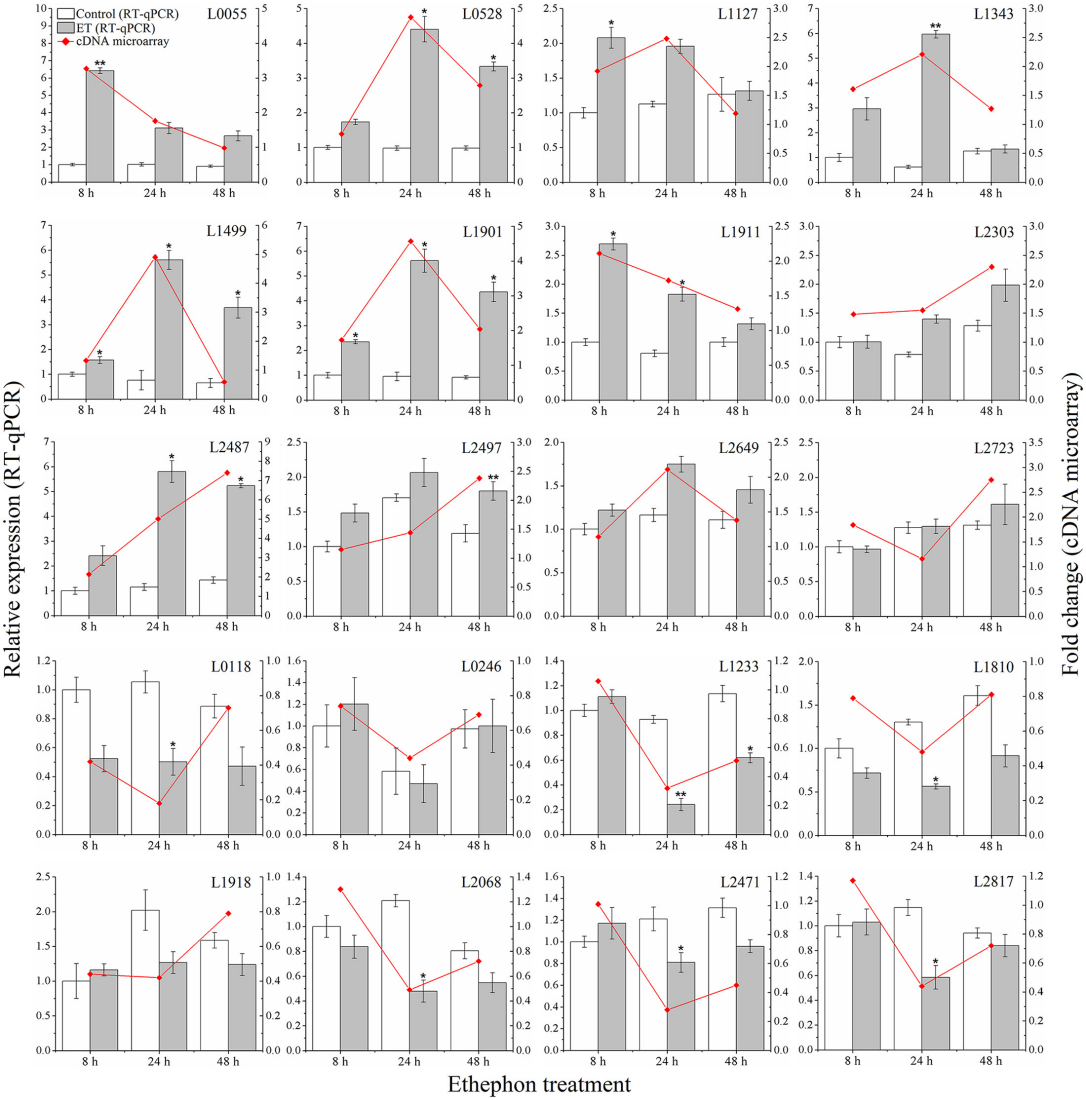
Validation of cDNA microarray data using RT-qPCR. The RT-qPCR analysis was performed as described in ‘Materials and methods’. The transcript abundance of each gene was determined by RT-qPCR and the values were shown as the mean ± standard deviation (n = 3). Statistical significance was determined by Student’s *t*-test using SPSS 19.0 software. Compared with the control, one asterisk showed a significant difference at *p* < 0.05 and two asterisks at *p* < 0.01.

To validate the analysis result of the cDNA microarray, 20 ET-induced genes (selected based on cDNA microarray data) were analyzed using RT-qPCR (Fig 6). Amplification efficiencies of the selected genes primer pairs ranged within 1.75–2.011 (S1 Table). Of the 20 genes, 15 showed the same expression pattern (up- or down-regulation, significance determined by Student’s *t*-test using SPSS 19.0 software, *p* < 0.05) in both cDNA microarray and RT-qPCR analysis. According to the RT-qPCR analysis, five genes (a glutaredoxin gene L2303, a profilin gene L2649, an AP2/ERF super family gene L2723 and two NAC domain-containing genes L0246 and L1918) that were not significantly different from the control (Student’s *t*-test, *p* < 0.05), showed different expression patterns in cDNA microarray and RT-qPCR analysis. There was a significant correlation between the fold-changes obtained under both techniques for each same expression pattern gene (rho = 0.867, *p* < 0.01, data analyzed by Spearman’s rho using SPSS 19.0 software). Morey et al. [59] reported that correlations between microarray and RT-qPCR data can vary widely within 0.48–0.94; they also indicated that criteria for the determination of an acceptable validation of microarray results by RT-qPCR are rarely defined. Therefore, taking into account that 15 of the studied ET-induced genes showed the same expression pattern under RT-qPCR and microarray analysis, we considered our microarray data validated by RT-qPCR analysis.

## Discussion

### ET is involved in several metabolic processes of the *H*. *brasiliensis* laticifers

Latex flow and *in situ* regeneration are the two primary yield-limiting factors of rubber trees, and they are closely connected with the latex physiological parameters, which reflect the metabolic activity of the laticifers [60]. The decreased levels of the plugging index and the TSC can delay the plugging of latex vessels and finally favor latex flow and latex production of the ethephon-treated rubber trees [30, 31, 61–63]. Latex regeneration depends on the supply and utilization of sucrose in the rubber tree’s laticifers. A lower sucrose content in the latex after ethephon stimulation indicated an active metabolism for latex regeneration, which is related to latex production [24, 25]. Emerging evidence demonstrated that ET is employed in these physiological processes of the laticifers by regulating expression of related genes [26, 27, 30–32]. And therefore, comprehensively profiling the genes involved in the latex production would help to elucidate the underlying basis of ethylene in regulation of the latex yield of rubber trees.

### ET is not an effective regulator of rubber biosynthesis-related genes

The NR biosynthesis of rubber trees is a typical plant secondary metabolism process with the basic precursor of sucrose, followed by the mevalonate (MVA)/2-C-methyl-D-erythritol 4-phosphate (MEP) pathway, which provide the direct precursor of IPP [10]. On the rubber particle, NR is formed through sequential condensation of hundreds of thousands of IPP units and is finally compartmentalized within a special organelle suspended in the latex of the laticiferous cells of rubber trees [3, 4, 64]. In this study, the gene L0291 encoding a rubber particle-bound CPT [65] was down-regulated in the latex of ethephon-treated rubber trees compared to control. Other genes of key enzymes or proteins involved in rubber biosynthesis, such as acetyl-coenzyme A acetyltransferase (AACT), 3-hydroxy-3-methylglutaryl-coenzyme A synthase, HMGR, mevalonate kinase (MVK), GGPS, REF and SRPP, did not significantly differ from controls (fold-changes ≥ 2 or ≤ –2, *q-*value < 0.05) (Table 3). The results showed that the NR biosynthesis of rubber trees might not be directly regulated by ET.

**Table 3:** Expression analysis of the rubber biosynthesis-related genes in the latex of the ethephon-treated rubber trees.

Gene ID	*H*.*brasiliensis* homolog	Description	Fold Change			*q*–value
			8 h	24 h	48 h	
L0291		cis-prenyltransferase	0.99	0.41	0.79	0.00
L1820	JT937325.1	cis-prenyltransferase				>0.05
L2531	JT937656.1	farnesyl diphosphate synthase	1.05	0.80	0.85	0.01
L0288	JT951018.1	rubber elongation factor	1.07	0.56	0.63	0.00
L0370	JT976134.1	rubber elongation factor	0.92	1.48	0.94	0.00
L0659	JT952916.1	rubber elongation factor	1.34	0.76	0.77	0.00
L0892	JT948343.1	rubber elongation factor				>0.05
L2039	JT956147.1	rubber elongation factor	0.80	0.85	0.89	0.01
L2040	JT939205.1	rubber elongation factor	1.09	0.91	1.09	0.00
L0823	JT949604.1	small rubber particle protein	1.17	1.05	1.03	0.04
L1443	JT952181.1	small rubber particle protein	1.14	1.01	1.15	0.03
L2308	JT933647.1	acetyl-CoA C-acetyltransferas	0.81	1.55	1.12	0.00
L2728	JT934477.1	acetyl-CoA C-acetyltransferas	1.05	0.79	1.07	0.01
L1887	JT929060.1	3-hydroxy-3-methylglutaryl coenzyme A synthase	0.66	0.79	1.12	0.00
L1099	JR346094.1	3-hydroxy-3-methylglutaryl coenzyme A reductase				>0.05
L1356	JT929971.1	3-hydroxy-3-methylglutaryl coenzyme A reductase				>0.05
L1919		3-hydroxy-4-methylglutaryl coenzyme A reductase	1.42	1.04	1.15	0.00
L2544	JT933044.1	isopentenyl pyrophosphate isomerase	0.95	0.96	0.79	0.04
L2266	JT971226.1	mevalonate kinase				>0.05
L1679	JT933986.1	geranylgeranyl-diphosphate synthase	1.02	0.90	0.94	0.04
L2442	JT929251.1	geranylgeranyl-diphosphate synthase	1.16	1.18	1.05	0.04
L1999	JT928900.1	5-phosphomevelonate kinase	1.39	0.76	0.86	0.00
L0593	JT938439.1	mevalonate diphosphate decarboxylase	1.16	1.46	1.11	0.04

### ET signal transduction-related genes are controlled by ethephon

ET plays an important role as a signaling molecule in plant development and stress response. Many proteins, such as ethylene resistant 1 (ETR1) [66], constitutive triple response 1 (CTR1) [67], ethylene insensitive 2 (EIN2) [68], ethylene insensitive 3 (EIN3) [69, 70] and ERF [70], participate in the plant ET signal transduction. The various members of the ET signaling transduction-related genes in the laticiferous cells of rubber trees are differentially regulated by ethephon treatment. In this study, five genes probably involved in ET signal transduction were identified. The latex gene L1911 encoding EIN3 was up-regulated in the ethephon-treated rubber trees. EIN3 is a novel nuclear protein, located downstream of the ET signal transduction pathway, that binds to the promoter of ERF gene and activates its transcription in an ET-dependent manner [71, 72]. Four latex genes, L0200, L1127, L2487 and L2497 encoding members of the AP2/ERF super family protein, were all up-regulated by ethephon stimulation. Transcription factors ERF1 and other AP2/ERF super family proteins can interact with the GCC box or CRT/DRE element in the promoter of target genes and activate downstream ET responses [71–73]. It was reported that the up-regulation of EIN3 and ERFs may play important roles in ET signal transduction of laticiferous cells in rubber trees. In addition, two genes, L0055 and L1901 encoding NAC domain-containing proteins were up-regulated. NAC domain-containing proteins are plant-specific transcription factors that play vital roles in plant development, stress responses and various metabolic processes. The expression of some NAC domain-containing proteins can be regulated by ET. Of the two genes, L1901 is a close homolog of *A*. *thaliana* NAC2, which is involved in ET signal transduction and regulated by EIN3 [74]. Further research would give insight into the biological functions that the NAC gene performs in increasing the latex metabolism of rubber trees stimulated with ethephon, which would reveal molecular mechanisms by which ET signal transduction regulates the latex metabolism of *H*. *brasiliensis*.

### ET down-regulates expression of ROS scavenging-related genes

ROS are the byproducts of plant aerobic metabolism, and are considered to have both harmful and beneficial functions in many physiological processes of plants. As important plant cell signaling molecules, ROS are involved in responses to biotic and abiotic stresses and in developmental and physiological processes. However, as toxic molecules that cause oxidative damage to lipids, proteins and nucleic acids, ROS can injure cells [75]. Under physiological steady-state conditions, ROS are scavenged by several anti-oxidative defense components, thereby maintaining a balance between ROS production and scavenging [76]. This equilibrium may be perturbed by a number of adverse environmental factors that generate ROS by activating various oxidases and peroxidases, or lead to the rapid accumulation of ROS known as an oxidative burst [77]. In this study, a significant decrease in RSH content of latex was observed after ethephon stimulation (Fig 1f) in unexploited rubber trees, and analysis of gene expression showed that seven latex genes probably involved in scavenging ROS were down-regulated in ethephon-treated rubber trees within 24 h, including COX5B gene (L0118), selenoprotein h-like protein gene (L0686), Cu/Zn SOD gene (L1233), glycosyl hydrolase family gene (L1607), phospholipid hydroperoxide glutathione peroxidase gene (L1810), thioredoxin h gene (L2068) and peroxiredoxin family protein gene (L2817) (Table 1). In the latex of ethephon-treated rubber trees, the NAD(P)H oxidase activities increased [36], whereas the expression level of ROS scavenging-related genes decreased, which may break the equilibrium between producing and scavenging of ROS, and result in rapid accumulation of ROS. Previous studies demonstrated that higher ROS levels can stimulate the production of endogenous ET [78, 79] in exploited rubber trees laticifers and was shown to be involved in latex yield [80]; however, excessive ROS accumulation can damage the lutoids and initiate programmed cell death (PCD) of the *H*. *brasiliensis* laticifers, which may lead to TPD [36, 38]. Taken all the data together a possible mechanism is suggested that the TPD of rubber trees might be caused by excessive ET stimulation, since ET can inhibit the expression of ROS scavenging-related genes, which might break the balance between producing and scavenging of ROS, and lead to excessive ROS accumulation in the laticifers and ultimately induce the TPD.

### ET up-regulates expression of sugar metabolism-related genes

Latex regeneration and rubber biosynthesis occur in the cytoplasm of highly specialized latex cells and requires sucrose as the unique precursor. ET stimulation of latex production results in high sugar flow from the surrounding cells of the inner bark towards the latex cells [20–24]. Latex is harvested by bark tapping that is a regular abiotic mechanical stress for the exploited rubber trees. Bark tapping can activate the latex cells to regenerate lost cytoplasm after latex expulsion, which causes a higher sucrose demand in the laticifers of exploited rubber trees compared to unexploited rubber trees [27]. In this study, a significant decrease in the sucrose content of latex was observed in unexploited rubber trees after ethephon stimulation (Fig 1e), and gene expression analysis showed that two genes probably involved in sucrose transport (SUT) were up-regulated in ethephon-treated unexploited rubber trees. The first SUT gene was L1343 that matched HbSUT1, which is related to sucrose importation into laticifers. Latex production of the ET-stimulated rubber trees was associated with high gene expression of putative sucrose transporter HbSUT1 in the unexploited rubber tree [25]. The second was a potential sugar transporter SWEET10-like gene (L1499) that was involved in multiple physiological processes by facilitating ion transport via interaction with ion transporters or as sugar transporters [81, 82], and this gene was strongly up-regulated in the laticifers of the ethephon-treated unexploited rubber trees. In addition, the L2343 encoding pyruvate dehydrogenase that catalyzes the pivotal irreversible reaction of glucose metabolism in the aerobic energy-generating pathways [83] was also up-regulated in ethephon-treated rubber trees. These results indicate that the ET stimulation of latex production was associated with accelerating the importation and metabolism of sugar, especially sucrose which improves the supply of carbon (e.g. acetyl-CoA) and energy (e.g. ATP) for rubber biosynthesis in laticifers of rubber trees.

### ET promotes latex flow by increasing expression of genes related to laticifer water circulation

Efficient water inflow into laticifers is crucial for latex flow and production, since it is the determinant of the latex TSC and fluidity after rubber tree tapping [6, 61]. A significant increase in the duration of latex flow and a remarkable decrease in TSC were observed after ethephon stimulation (Fig 1b and 1d). The results indicated an enhanced water importation to the laticifers, which would cause the latex dilution and favor latex flow. As the vessel rings of mature laticifers are devoid of plasmodesmata, water circulation between laticifers and surrounding cells is believed to be governed by PIPs [30–32]. In the present study, the latex gene L2145 encoding HbPIP2;3, which is involved in exploited rubber trees laticifer water circulation and regulated by ET [30], was strongly up-regulated in ethephon-treated rubber trees. The results showed a similar gene expression pattern to previous researches, and indicated that the HbPIP2;3 might play an important role in water circulation and ET stimulation of latex production in rubber tree laticifers.

### ET delays the plugging of latex vessels by modifying expression of actin cytoskeleton assembly-related genes

Duration of latex flow is a key limiting factor of rubber yield [62], and is mainly determined by plugging formation at the end of severed latex vessels after tapping. The results demonstrated that ethephon stimulation can significantly increase the duration of latex flow and decrease the plugging index of the unexploited rubber trees (Fig 1b and 1c). These results were similar to those of the tapped trees stimulated by ethephon [63], which indicates that ET can delay the plugging of latex vessels so as to prolong the duration of latex flow and favor latex flow. A protein-network constituted by an actin cytoskeleton forms at the end of severed laticifers, and plays a critical role in laticifer plugging [63]. In addition, the depolymerization of the actin cytoskeleton has been reported to stimulate the latex yield of rubber trees [84]. Profilin is an important member of the actin-binding proteins, and is best known as a regulator of actin filament non-equilibrium assembly and disassembly [85, 86]. In this study, two latex profilin genes (L0528 and L2649) were up-regulated in ethephon-treated rubber trees, and two formin protein genes were down-regulated. As an actin-binding protein, formin can interact with profilin and initiators of actin assembly, playing a central regulator role in actin polymerization [87, 88]. Taken all the results together, one suggestion is put forward that ET might modify the optimum equilibrium between unpolymerized actin molecules and assembled actin filaments by regulation of profilin and formin, which delays the plugging of laticifers and increases the latex production of the ethephon-treated rubber trees.

### Concluding Remarks

**Fig 7:**
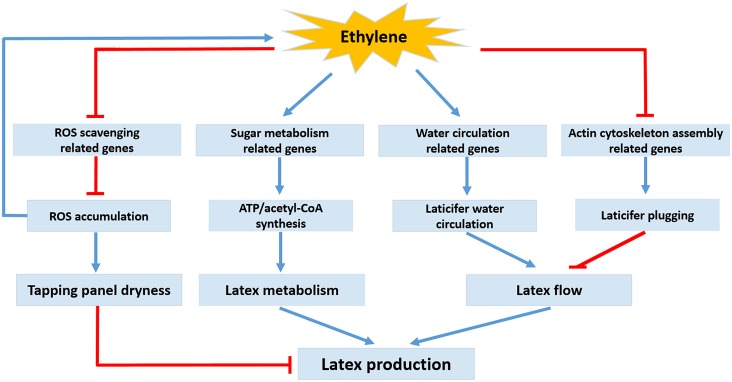
Schematic diagram of possible mechanisms of ET responses in the laticifers of the mature virgin rubber trees based on the presented data and previous references. ET stimulation up-regulates expression of sugar metabolism-related genes in the latex, which accelerates the importation and metabolism of sugar and improves the supply of carbon (e.g. acetyl-CoA) and energy (e.g. ATP) for rubber biosynthesis. In addition, ET favors promotion of latex flow by increasing expression of genes related to laticifer water circulation, and thus delays the plugging of the laticiferous ducts by modifying expression of genes related to actin cytoskeleton assembly, which prolongs the duration of latex flow and increases latex production. Nevertheless, ET stimulation can also down-regulate expression of several ROS scavenging-related genes, which may break the equilibrium between producing and scavenging of ROS, and result in rapid ROS accumulation in the laticifers. Excessive ROS accumulation would damage lutoids and initiate programmed cell death (apoptosis) of the laticifers, which might cause the TPD of rubber trees.

ET is a structurally simple gaseous hormone that regulates complex physiological processes of plants. Ethephon stimulation can induce remarkable physiological and biochemical changes within the laticiferous cells of rubber trees, which collectively contribute to increased latex production. Many laticifer-expressed genes that were regulated by ET were identified using a cDNA microarray in this research. The presented data suggest that ethephon increases the latex production of the mature virgin rubber trees mainly by regulating expression of many genes significantly involved in laticifer water circulation, production and scavenging of ROS, sugar metabolism and actin cytoskeleton assembly—rather than by regulating expression of genes involved directly in latex regeneration or rubber biosynthesis (Fig 7). Although mature virgin trees were used in this research, the altered gene expression profile in the laticifers of ethephon-treated rubber trees will also provide valuable insights into the molecular events and regulatory mechanisms by which ethephon stimulation increases the latex yield of *H*. *brasiliensis*.

## Supporting Information

S1 Fig Kinetics of latex yeild (a), duration of latex flow (b), plugging index (c), TSC (d), sucrose content (e) and RSH content (f) subjected to different duration of ethephon treatment. The latex yeild and physiological parameters i.e., duration of latex flow, TSC, plugging index, sucrose content and RSH content were measured at 0, 1, 4, 48, 24 and 48 h after ethephon treatment. The values were shown as the means ± standard deviation (n = 3) for each including six trees. One-way ANOVA was performed using SPSS 19.0 software. The Student–Newman–Keuls test was used for multiple comparisons testing to investigate the significant differences between groups. Bars with different letters were significantly different at the *p* < 0.05 level.
(TIF)

S1 Table Oligonucleotide primers used for the RT-qPCR reactions in this study. (DOC)

S2 Table Detailed inventory of the probes used in the *H*. *brasiliensis* latex cDNA microarray. (DOC)

S3 Table Probe sequences of the *H*. *brasiliensis* latex cDNA microarray. (DOC)

S4 Table The differentially expressed genes in the laticifers of rubber trees stimulated with ethephon for 1 h. (DOC)

S5 Table The differentially expressed genes in the laticifers of rubber trees stimulated with ethephon for 4 h. (DOC)
