# Supplementary figures and images for: Profiling Ethylene-Responsive Genes Expressed in the Latex of the Mature Virgin Rubber Trees Using cDNA Microarray
Source: PLoS One. 2016 Mar 17;11(3):e0152039. doi: 10.1371/journal.pone.0152039 (PMC4795647; doi:10.1371/journal.pone.0152039)

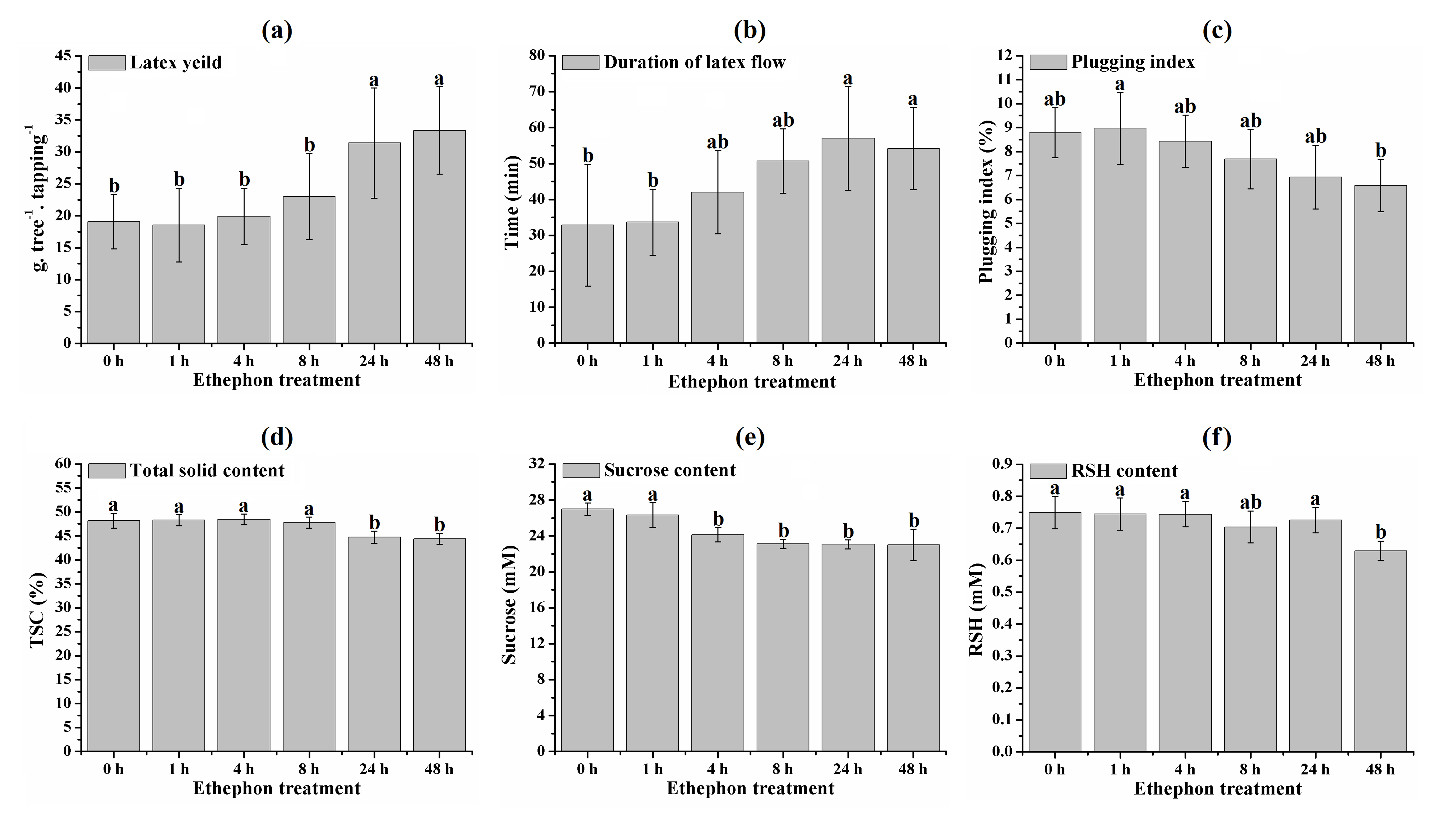

Supplement: S1 Fig — The latex yeild and physiological parameters i.e., duration of latex flow, TSC, plugging index, sucrose content and RSH content were measured at 0, 1, 4, 48, 24 and 48 h after ethephon treatment. The values were shown as the means ± standard deviation (n = 3) for each including six trees. One-way ANOVA was performed using SPSS 19.0 software. The Student–Newman–Keuls test was used for multiple comparisons testing to investigate the significant differences between groups. Bars with different letters were significantly different at the p < 0.05 level. (TIF) [file pone.0152039.s001.tif]
