# Supplementary material for: Profiling Ethylene-Responsive Genes Expressed in the Latex of the Mature Virgin Rubber Trees Using cDNA Microarray
Source: PLoS One. 2016 Mar 17;11(3):e0152039. doi: 10.1371/journal.pone.0152039 (PMC4795647; doi:10.1371/journal.pone.0152039)
Supplement: S1 Table — (DOC) [file pone.0152039.s002.doc]

**S1 Table.** Oligonucleotide primers used for the RT-qPCR reactions in this work

| Primer name | Sequence (5’-3’) | Primer efficiency | PCR product (bp) |
| --- | --- | --- | --- |
| L0055_FW | AACAGTGCTAATGCTGCTGATG | 1.851 | 125 |
| L0055_RV | GGCTCCACAGGATTTGAAAGAT |
| L0528_FW | ACCGGCCAAGCTCTCATTATT | 1.742 | 193 |
| L0528_RV | ACTAATGCCAGTTCATAGCCAGC |
| L1127_FW | TGTTTCAGGAGTCGTCAGAACTG | 1.903 | 117 |
| L1127_RV | TCCACACACCAGATGCTTGATA |
| L1343 _FW | CCACCCTCTTCAGCCTCTATTAG | 1.877 | 161 |
| L1343 _RV | ATAGGTCCACAGAGCCAAATGA |
| L1499 _FW | AAGTGAAGGATTCCAGTCCATTC | 1.823 | 117 |
| L1499 _RV | GTGAAGGAGTTGATGGTGATGAG |
| L1901 _FW | TACAGAGTGAGCCTAAGTGGAAAG | 1.779 | 106 |
| L1901 _RV | GGATACAGTGACCCGAACTCTT |
| L1911 _FW | CGATGAGGAGATTGATGTTGAT | 1.882 | 101 |
| L1911 _RV | CGACACCTTCCTTACTCTTGTTC |
| L2303_FW | TCTTTATCCTTTGACGCTGTCG | 1.787 | 196 |
| L2303_RV | GTGGAAGAGGGAGAGCAGAGAT |
| L2487_FW | CCTAATAATGCCAATAAAGAGCAG | 1.843 | 132 |
| L2487_RV | CATTACAGTGGCAGTCTTCTCACA |
| L2497 _FW | TTCCACCGATACCTTCTCCATA | 1.852 | 190 |
| L2497 _RV | GGGCTGAGTTTAGAGAAGGATGA |
| L2649_FW | GGCAAACTTACGTAGACGAACA | 1.871 | 156 |
| L2649_RV | CTTTCATGATGTCAGTGATCTCCT |
| L2723_FW | GGAGGTAGAGAAGGTGAACAATG | 1.965 | 151 |
| L2723_RV | TGATTGTCCATCCAGATAAGGA |
| L0118_FW | ATTTCCAGGATACGATGAAGCA | 1.977 | 119 |
| L0118_RV | AAAGATACCGTCCCATCAGTTG |
| L0246_FW | GGTTATGCACCAATACCATCTTG | 2.011 | 152 |
| L0246_RV | CATGGTCAATTCGATTTCTCAAG |
| L1233 _FW | CAACCACCGTAACTGGAAGTGT | 1.917 | 196 |
| L1233 _RV | CCATCATCACCAACATTGACAT |
| L1810_FW | TGAACAGATTGTGGAGTTTGCT | 1.802 | 185 |
| L1810_RV | CATTGCCATCTTATCAAACCAG |
| L1918_FW | CCTGAGAATGAGGAGGAAGGAG | 1.922 | 100 |
| L1918_RV | ATATAGTGCGCAAGCTGAGGAA |
| L2068_FW | GCCATGAACAGGTGATGAAGTC | 1.791 | 117 |
| L2068_RV | GCATGCACCAGGAAGCTGTA |
| L2471_FW | AATCGTTCTCATCATTGCTGCTA | 1.821 | 110 |
| L2471_RV | AAGTCACGATGCCTTCTTGTTG |
| L2817_FW | TCGCTACTGCTTGCTCTACGAT | 1.863 | 190 |
| L2817_RV | GAACACCGACGATTACAACCTT |
| Hb18SrRNA_FW | GCTCGAAGACGATCAGATACC | 1.880 | 146 |
| Hb18SrRNA_RV | TTCAGCCTTGCGACCATAC |

* *H.brasiliensis* 18S rRNA gene with GenBank acc. No.: AB268099 was used as internal reference.
