# Supplementary material for: Profiling Ethylene-Responsive Genes Expressed in the Latex of the Mature Virgin Rubber Trees Using cDNA Microarray
Source: PLoS One. 2016 Mar 17;11(3):e0152039. doi: 10.1371/journal.pone.0152039 (PMC4795647; doi:10.1371/journal.pone.0152039)
Supplement: S2 Table — (DOC) [file pone.0152039.s003.doc]

**S2 Table.** Detailed inventory of the latex cDNA microarray probes

| **EST ID** | ***H.brasiliensis* homolog** | **Description** | **e-Value** | **Identity** | **Length (bp)** |
| --- | --- | --- | --- | --- | --- |
| L0001 | JR366223.1 | C-repeat dehydration-responsive element-binding factor 4 | 2.24E-49 | 73.30% | 451 |
| L0002 | JT952757.1 | non-specific phospholipase c3-like | 1.01E-76 | 90.05% | 391 |
| L0003 | JT948713.1 | thiamine pyrophosphokinase 1-like isoform x2 | 8.04E-73 | 93.85% | 343 |
| L0004 | JT934287.1 | DOF-type zinc finger domain-containing family protein | 1.21E-65 | 66.25% | 639 |
| L0005 | JT964365.1 | 60S ribosomal protein L27a-3-like | 2.02E-57 | 95.30% | 377 |
| L0006 | JT934286.1 | elongator complex protein 4 | 1.27E-93 | 83.80% | 1075 |
| L0007 | JT965470.1 | Transmembrane emp24 domain-containing protein p24delta9 | 1.11E-56 | 91.05% | 546 |
| L0008 | JT939329.1 | 60S ribosomal protein L7a | 2.97E-59 | 97.15% | 458 |
| L0009 | JT919883.1 | U-box domain-containing protein 13 | 5.71E-110 | 92.70% | 523 |
| L0010 | JR364885.1 | protein light-dependent short hypocotyls 10-like | 8.15E-34 | 98.30% | 561 |
| L0011 | JT937029.1 | abscisic acid receptor pyl8 | 1.16E-125 | 91.05% | 879 |
| L0012 | JT960862.1 | protein ralf-like 34 | 3.72E-24 | 91.90% | 367 |
| L0013 | JT916877.1 | enolase-phosphatase e1-like | 3.00E-24 | 55.75% | 368 |
| L0014 | JT964595.1 | bifunctional protein 2-like | 9.51E-69 | 93.50% | 360 |
| L0015 | JT916228.1 | mitochondrial substrate carrier family protein c-like | 4.84E-20 | 90.25% | 302 |
| L0016 | JT934651.1 | tetratricopeptide repeat -like superfamily protein | 1.85E-178 | 85.30% | 1189 |
| L0017 | JT944052.1 | hypothetical protein JCGZ_13358 | 2.32E-80 | 87.45% | 470 |
| L0018 | JR344567.1 | mitochondrial import inner membrane translocase subunit Tim16 | 3.34E-73 | 93.05% | 423 |
| L0019 | JT931643.1 | acetylornithine deacetylase | 7.53E-77 | 95.60% | 365 |
| L0020 | JT946419.1 | monodehydroascorbate reductase | 1.11E-46 | 93.80% | 517 |
| L0021 | JT945993.1 | f-box protein at1g47056-like | 5.60E-80 | 89.35% | 405 |
| L0022 | JT921863.1 | o-fucosyltransferase family protein isoform partial | 1.03E-74 | 87.15% | 445 |
| L0023 | JT926563.1 | BTB/POZ domain-containing family protein | 3.27E-48 | 94.00% | 253 |
| L0024 | JT929656.1 | tbc1 domain family member | 5.30E-92 | 92.55% | 681 |
| L0025 | JT949537.1 | methylesterase 10-like | 8.38E-47 | 83.40% | 295 |
| L0026 | JT929934.1 | f-box protein skip16 | 7.14E-43 | 83.75% | 452 |
| L0027 | JT938808.1 | plastidial lipoyltransferase 2-like | 1.68E-60 | 81.60% | 629 |
| L0028 | JR348991.1 | Agmatine deiminase | 4.45E-86 | 91.20% | 479 |
| L0029 | JT926115.1 | frigida-like protein 3 | 5.69E-05 | 54.25% | 108 |
| L0030 | JT930106.1 | macpf domain-containing protein at4g24290 | 2.69E-131 | 91.85% | 602 |
| L0031 | JT941097.1 | U-box domain-containing protein 3 | 1.43E-46 | 89.30% | 268 |
| L0032 | JT928637.1 | dihydroorotate dehydrogenase | 5.14E-80 | 92.35% | 510 |
| L0033 | JT930698.1 | eukaryotic translation initiation factor 3 subunit e | 2.96E-162 | 96.55% | 696 |
| L0034 | JT947773.1 | cucumisin | 2.88E-26 | 85.15% | 620 |
| L0035 | JT931112.1 | peter pan-like protein | 1.71E-49 | 97.50% | 548 |
| L0036 | JT955983.1 | HSP20-like chaperones superfamily protein | 2.74E-47 | 69.35% | 484 |
| L0037 | JT941809.1 | Biotin carboxyl carrier protein subunit of of Het-ACCase (BCCP1) | 1.58E-16 | 65.65% | 300 |
| L0038 | JT926595.1 | GTPase activating protein | 4.06E-154 | 95.55% | 712 |
| L0039 | JR366632.1 | 3 -5 exonuclease domain-containing family protein | 6.03E-63 | 91.70% | 315 |
| L0040 | JT949254.1 | vesicle transport V-snare 12 | 1.93E-112 | 89.95% | 660 |
| L0041 | JT923948.1 | probable polyamine oxidase 2 | 1.39E-46 | 92.00% | 258 |
| L0042 | JT971891.1 | casein kinase II subunit alpha-2 | 6.21E-56 | 97.85% | 480 |
| L0043 | JR350842.1 |  |  |  | 206 |
| L0044 | JT917150.1 | gsk3 shaggy-like protein kinase 1 | 1.59E-169 | 97.00% | 838 |
| L0045 | JT950490.1 | GDP dissociation inhibitor family protein | 3.48E-54 | 97.00% | 264 |
| L0046 | JT930900.1 | fatty acyl- reductase 3-like | 1.77E-89 | 77.35% | 441 |
| L0047 | JT967874.1 | mateRNAl effect embryo arrest 60 | 3.94E-49 | 78.45% | 626 |
| L0048 | JT942712.1 | bax inhibitor 1-like | 2.66E-20 | 97.35% | 308 |
| L0049 | JR344512.1 | regulatory components of ABA receptor 3 | 1.11E-41 | 90.40% | 353 |
| L0050 | JT928273.1 | carbamoyl phosphate synthetase a | 1.53E-74 | 90.85% | 381 |
| L0051 | JT957340.1 | diphthamide biosynthesis protein 3-like | 1.01E-48 | 90.70% | 449 |
| L0052 | JT960556.1 | 60S ribosomal protein L22-2 | 5.58E-34 | 96.10% | 273 |
| L0053 | JT957493.1 | 40S ribosomal protein S13* | 2.00E-22 | 79.00% | 311 |
| L0054 | JT928632.1 | DNA-binding family protein | 1.65E-61 | 70.20% | 395 |
| L0055 | JT924152.1 | NAC domain-containing protein 78 | 1.45E-57 | 63.40% | 470 |
| L0056 | JT944977.1 | inositol transporter 1 like protein | 3.77E-34 | 92.35% | 267 |
| L0057 | JT945836.1 | proteasome subunit alpha type-6 | 2.36E-45 | 97.55% | 300 |
| L0058 | JT960731.1 | conserved hypothetical protein | 6.55E-09 | 66.67% | 430 |
| L0059 | JR365955.1 | transcription factor MYB1r1-like | 1.25E-51 | 63.90% | 612 |
| L0060 | JT940201.1 | fasciclin-like arabinogalactan protein 7 | 7.34E-21 | 96.90% | 239 |
| L0061 | JT969124.1 | gdsl esterase lipase at4g01130-like | 1.46E-48 | 87.95% | 291 |
| L0062 | JT946137.1 | transcription factor MYC | 8.62E-116 | 78.20% | 589 |
| L0063 | JT943015.1 | tetratricopeptide repeat protein 1-like | 1.70E-52 | 94.35% | 422 |
| L0064 | JT946196.1 | tropinone reductase homolog | 5.10E-54 | 81.95% | 294 |
| L0065 | JT963832.1 | conserved hypothetical protein | 8.34E-43 | 75.65% | 287 |
| L0066 | JT923851.1 | AT-rich interactive domain-containing protein 5-like | 1.57E-50 | 93.00% | 254 |
| L0067 | JT936952.1 | 60S acidic ribosomal protein P0 | 6.88E-75 | 96.60% | 352 |
| L0068 | JT957884.1 | light-induced chloroplastic | 9.93E-100 | 92.05% | 550 |
| L0069 | JT938614.1 | RaS-related protein Rabc1 | 5.68E-138 | 95.25% | 891 |
| L0070 | JT944039.1 | NC domain-containing family protein | 2.99E-36 | 62.50% | 669 |
| L0071 | JT925359.1 | poly RNA polymerase protein cid1 | 9.84E-73 | 89.80% | 372 |
| L0072 | JT948433.1 | 40S ribosomal protein S2-3-like | 1.06E-149 | 98.55% | 651 |
| L0073 | JT955945.1 | protein pxr1 | 2.08E-37 | 90.85% | 597 |
| L0074 | JT968654.1 | coronatine-insensitive 1 (COI1) * | 3.00E-178 | 99.00% | 362 |
| L0075 | JT946782.1 | V-type proton ATPase subunit e | 9.91E-91 | 96.00% | 541 |
| L0076 | JT931509.1 | myosin-h heavy chain isoform 2 | 4.76E-78 | 82.15% | 536 |
| L0077 | JT963485.1 |  |  |  | 549 |
| L0078 | JT938905.1 | Ras-GTPase-activating protein-binding protein, putative* | 3.00E-05 | 69.00% | 226 |
| L0079 | JT954636.1 |  |  |  | 490 |
| L0080 | JT961831.1 | conserved hypothetical protein | 3.62E-42 | 68.25% | 539 |
| L0081 | JT960873.1 | FAT domain-containing family protein* | 2.00E-11 | 88.00% | 561 |
| L0082 | JT950884.1 | 14-3-3-like protein b | 1.03E-63 | 97.45% | 488 |
| L0083 | JT927759.1 | flowering time control protein | 2.15E-31 | 55.45% | 574 |
| L0084 | JT950731.1 | Ubiquitin carboxyl-terminal hydrolase isozyme l3 | 1.58E-72 | 93.25% | 525 |
| L0085 | JT951922.1 | conserved hypothetical protein | 1.07E-70 | 76.05% | 303 |
| L0086 | JT968446.1 | uroporphyrinogen decarboxylase | 2.70E-39 | 80.90% | 491 |
| L0087 | JT949678.1 | Ubiquitin-like superfamily isoform 1 | 1.62E-51 | 51.95% | 588 |
| L0088 | JR355713.1 |  |  |  | 330 |
| L0089 | JT947919.1 | werner syndrome-like exonuclease | 6.69E-93 | 58.50% | 562 |
| L0090 | JR349839.1 | arginine/Serine-rich splicing factor | 1.40E-18 | 74.40% | 561 |
| L0091 | JT950401.1 | PHD finger-like domain-containing protein 5b | 3.96E-71 | 98.50% | 258 |
| L0092 | JT953466.1 | hypothetical protein JCGZ_12701 | 7.07E-14 | 71.00% | 465 |
| L0093 | JT955491.1 | chaperone protein DNAj 72 | 7.05E-35 | 89.45% | 366 |
| L0094 | JT973817.1 | DNAj homolog subfamily b member 6-like | 5.05E-07 | 75.40% | 297 |
| L0095 | JT977253.1 | conserved hypothetical protein | 5.85E-25 | 93.00% | 132 |
| L0096 | JT925195.1 | protein IQ-domain 1-like | 2.18E-39 | 73.60% | 330 |
| L0097 | JT934437.1 | ARM repeat superfamily protein isoform 1 | 1.06E-124 | 86.65% | 690 |
| L0098 | JT947019.1 | FAR1-RELATED SEQUENCE 5-like* | 2.00E-11 | 74.00% | 508 |
| L0099 | JR361141.1 | RNA recognition motif-containing family protein | 5.74E-13 | 75.30% | 479 |
| L0100 | JT940639.1 | pentatricopeptide repeat-containing protein | 1.31E-63 | 89.05% | 395 |
| L0101 | JT919774.1 | dynamin family protein | 6.43E-18 | 94.40% | 202 |
| L0102 | JT959875.1 | hypothetical protein JCGZ_08138 | 1.59E-36 | 89.85% | 246 |
| L0103 | JT954163.1 | protein binding protein | 1.59E-28 | 61.20% | 589 |
| L0104 | JT962900.1 | acyl-CoA-binding protein | 1.89E-33 | 75.95% | 481 |
| L0105 |  | translation initiation factor EIF-2b subunit delta | 2.68E-41 | 71.10% | 556 |
| L0106 | JT967537.1 | heat shock protein | 2.57E-37 | 93.50% | 583 |
| L0107 | JT966412.1 | DNA-binding protein s1fa | 5.88E-21 | 89.05% | 608 |
| L0108 | JR365812.1 | conserved hypothetical protein | 2.08E-17 | 76.70% | 235 |
| L0109 | JT953748.1 | Alba DNA RNA-binding protein | 1.48E-85 | 93.50% | 595 |
| L0110 | JT917882.1 | outer ARM dynein light chain 1 isoform 1 | 3.29E-87 | 82.55% | 529 |
| L0111 | JT929574.1 | triadin-like isoform x3 | 6.48E-31 | 56.85% | 464 |
| L0112 | JT939599.1 | Endonuclease V | 1.69E-58 | 86.00% | 461 |
| L0113 | JR348845.1 | 3-ketoacyl- synthase 11 | 9.98E-74 | 97.65% | 523 |
| L0114 | JT955222.1 | krueppel-like factor 17 | 2.36E-45 | 89.55% | 678 |
| L0115 | JT962406.1 | DET1- and DDB1-associated protein 1 | 7.09E-60 | 91.55% | 587 |
| L0116 | JT934597.1 | retroelement pol polyprotein | 1.06E-30 | 71.25% | 601 |
| L0117 | JT953613.1 | mitochondrial import inner membrane translocase subunit Tim17 Tim22 Tim23 family protein | 2.28E-104 | 82.95% | 740 |
| L0118 | JT949014.1 | cytochrome c oxidase subunit 5b-like | 5.30E-49 | 96.85% | 585 |
| L0119 | JR366703.1 | eukaryotic translation initiation factor 3 subunit d-like | 2.26E-61 | 83.15% | 615 |
| L0120 | JR349675.1 | calcium ion binding protein | 6.05E-35 | 87.50% | 456 |
| L0121 | JR365790.1 | cytochrome b5 isoform Cb5-B | 1.77E-85 | 91.75% | 691 |
| L0122 | JT934476.1 | formate mitochondrial | 4.18E-62 | 93.25% | 467 |
| L0123 | JR365133.1 | protein early responsive to dehydration 15-like | 2.41E-57 | 71.50% | 622 |
| L0124 | JT958789.1 | 17.5 kDa class II heat shock protein | 9.11E-71 | 92.10% | 648 |
| L0125 | JR366769.1 | hypothetical protein JCGZ_25941 | 3.14E-12 | 90.65% | 483 |
| L0126 | JT968142.1 | band 7 family protein | 1.07E-49 | 96.65% | 658 |
| L0127 | JT960909.1 | hypothetical protein POPTR_0002s10550g | 8.36E-55 | 93.45% | 478 |
| L0128 | JT952423.1 | Rab6-interacting golgin | 7.25E-76 | 88.50% | 500 |
| L0129 | JT946385.1 | hypothetical protein JCGZ_22853 | 1.07E-87 | 86.90% | 627 |
| L0130 | JR346118.1 | conserved hypothetical protein* | 2.00E-13 | 69.00% | 623 |
| L0131 | JT915140.1 | lysine-specific demethylase JMJ25-like | 7.00E-50 | 69.20% | 551 |
| L0132 | JT935608.1 | leukocyte receptor cluster member 1 homolog | 5.28E-62 | 77.75% | 564 |
| L0133 | JT937994.1 | OB-fold- isoform 3 | 1.23E-33 | 82.55% | 570 |
| L0134 | JT956040.1 |  |  |  | 626 |
| L0135 | JT968693.1 | josephin-like protein | 4.32E-31 | 70.50% | 634 |
| L0136 | JT975752.1 |  |  |  | 568 |
| L0137 | JT939267.1 | RNA-binding protein 25 | 2.54E-81 | 93.45% | 370 |
| L0138 | JR364586.1 | activator of basal transcription | 1.47E-25 | 66.00% | 642 |
| L0139 | JT956020.1 | unnamed protein product | 8.40E-43 | 75.90% | 533 |
| L0140 | JT932367.1 | proton-coupled amino acid transporter 3-like | 9.66E-49 | 76.15% | 609 |
| L0141 | JT939202.1 | uncharacterized loc101208715 | 1.01E-122 | 89.25% | 651 |
| L0142 | JT942750.1 | esterase lipase thioesterase family protein | 1.85E-156 | 87.35% | 589 |
| L0143 | JR364744.1 | protein light-dependent short hypocotyls 10-like | 4.60E-14 | 93.40% | 448 |
| L0144 | JT939789.1 | hydroxyphenylpyruvate reductase-like | 2.82E-166 | 86.25% | 107 |
| L0145 | JT956630.1 | mitochondrial ribosomal protein L37 isoform 1 | 2.39E-46 | 91.80% | 565 |
| L0146 | JT922841.1 | hypothetical protein JCGZ_06297 | 1.53E-84 | 83.25% | 381 |
| L0147 | JT957981.1 | bifunctional aas | 4.52E-50 | 79.90% | 598 |
| L0148 | JT960476.1 | splicing factor 3b subunit | 1.90E-58 | 99.20% | 490 |
| L0149 | JT915819.1 | methyltransferase-like protein | 1.12E-12 | 92.00% | 103 |
| L0150 | JT957547.1 | iron-sulfur assembly mitochondrial | 3.50E-79 | 92.85% | 688 |
| L0151 | JT935593.1 | ATP synthase subunit mitochondrial-like | 1.48E-36 | 88.40% | 552 |
| L0152 | JT949477.1 | mateRNAl effect embryo arrest 59 | 3.50E-67 | 72.55% | 630 |
| L0153 | JT924229.1 | Serine-threonine protein kinase, plant-type, putative | 9.45E-121 | 85.75% | 619 |
| L0154 | JR364176.1 | maturation-associated SRC1-like protein* | 5.00E-39 | 72.00% | 574 |
| L0155 | JR362728.1 | E3 ubiquitin-protein ligase rha1b-like | 1.11E-99 | 81.05% | 645 |
| L0156 | JT921481.1 | glycine--tRNA ligase mitochondrial-like | 6.86E-31 | 88.60% | 486 |
| L0157 | JT961633.1 | mitochondrial pyruvate carrier 2-like | 4.21E-63 | 97.10% | 614 |
| L0158 | JT954252.1 | senescence-associated family protein | 5.94E-45 | 79.60% | 526 |
| L0159 | JT947303.1 | rRNA intron-encoded homing endonuclease | 3.87E-47 | 70.35% | 618 |
| L0160 |  | DNA (cytosine-5)-methyltransferase 1-like | 3.78E-38 | 55.13% | 642 |
| L0161 | JT960932.1 | Protein CREG1 precursor, putative | 5.91E-57 | 90.10% | 583 |
| L0162 | JT960024.1 | signal recognition particle 9 kDa protein | 5.94E-66 | 94.55% | 623 |
| L0163 | JT939782.1 | p-type ATPase | 2.01E-63 | 77.60% | 657 |
| L0164 | JT949522.1 | NADH dehydrogenase | 3.38E-49 | 89.10% | 542 |
| L0165 | JT918636.1 | f-box protein fbw2 | 7.85E-44 | 77.10% | 598 |
| L0166 | JT934114.1 | histidine--tRNA ligase-like | 9.67E-40 | 65.30% | 648 |
| L0167 | JT952964.1 | Ubiquitin-conjugating enzyme e2-17 kDa | 4.17E-104 | 98.05% | 655 |
| L0168 | JT959999.1 |  |  |  | 534 |
| L0169 | JT916838.1 | ATP-NAD kinase family protein | 2.68E-78 | 98.00% | 702 |
| L0170 | JT962091.1 | 60S ribosomal protein L35a | 5.02E-75 | 98.35% | 659 |
| L0171 | JT949447.1 | C-jun-amino-terminal kinase-interacting protein 3 | 1.51E-72 | 70.25% | 620 |
| L0172 | JT979351.1 |  |  |  | 570 |
| L0173 | JT931456.1 | sec-independent protein translocase protein chloroplastic-like | 6.58E-85 | 95.60% | 676 |
| L0174 |  | protein rough sheath 2 homolog | 1.36E-66 | 61.20% | 643 |
| L0175 | JT947578.1 | Ras-related protein Raba1f | 1.80E-144 | 98.30% | 134 |
| L0176 | JR360674.1 | protein vacuoleless1 | 1.09E-89 | 90.80% | 627 |
| L0177 | JT938902.1 | adipor-like receptor cg5315-like | 6.39E-178 | 79.65% | 500 |
| L0178 | JT964319.1 | hypothetical protein JCGZ_12700 | 1.41E-12 | 70.00% | 427 |
| L0179 | JT959921.1 | DNA-directed RNA polymerases IV and V subunit 11 | 3.56E-78 | 97.00% | 595 |
| L0180 | JT924829.1 | tyrosyl-DNA phosphodiesterase 1-like | 9.21E-79 | 79.00% | 561 |
| L0181 | JR347470.1 | Protein MSF1 | 5.44E-97 | 94.40% | 670 |
| L0182 | JT959613.1 | latex abundant protein 1 | 3.48E-67 | 75.50% | 659 |
| L0183 | JT925292.1 | conserved oligomeric golgi complex subunit 4 | 3.47E-114 | 90.40% | 668 |
| L0184 | JR363887.1 | 60S ribosomal protein L27-like | 1.90E-62 | 85.85% | 515 |
| L0185 | JT976505.1 |  |  |  | 553 |
| L0186 | JT957969.1 | cytochrome b5 isoform Cb5-A | 1.75E-80 | 90.75% | 629 |
| L0187 | JT920789.1 | probable lrr receptor-like Serine threonine-protein kinase at5g10290 | 2.13E-52 | 92.60% | 572 |
| L0188 | JT934542.1 | integral membrane yip1 family protein | 4.40E-19 | 83.00% | 365 |
| L0189 | JT936558.1 | sec14p-like phosphatidylinositol transfer family protein isoform 1 | 1.11E-37 | 66.65% | 505 |
| L0190 |  | zinc finger protein zat9-like | 1.76E-74 | 77.20% | 559 |
| L0191 | JT968096.1 | DNA polymerase delta subunit 4 family protein | 6.66E-49 | 72.15% | 600 |
| L0192 | JT961092.1 | mitochondrial ATP synthase subunit g protein | 6.69E-68 | 95.50% | 452 |
| L0193 | JT937105.1 | probable protein phosphatase 2C 51 | 3.86E-54 | 72.30% | 517 |
| L0194 | JT969609.1 | ASR-like protein 2 | 3.90E-36 | 100.00% | 599 |
| L0195 | JT932989.1 | transmembrane protein 128 | 1.04E-88 | 86.30% | 714 |
| L0196 | JT939904.1 | RNA exonuclease 4 | 3.73E-134 | 85.45% | 583 |
| L0197 | JT949939.1 | TATA-binding protein-associated phosphoprotein | 2.50E-97 | 90.60% | 667 |
| L0198 | JT959051.1 | outer envelope protein of 80 kDa isoform 2 | 9.82E-45 | 52.65% | 654 |
| L0199 | JR366916.1 | conserved hypothetical protein | 8.47E-52 | 83.22% | 646 |
| L0200 |  | ethylene-responsive element binding protein 2 | 3.73E-32 | 52.75% | 435 |
| L0201 | JT941260.1 | elongation factor 1-beta 1 | 1.01E-57 | 97.45% | 651 |
| L0202 | JT977810.1 | DNA-directed RNA polymerase III subunit rpc9 | 1.53E-18 | 74.25% | 624 |
| L0203 | JT950602.1 | elicitor-responsive protein 1-like | 9.09E-69 | 82.90% | 628 |
| L0204 | JT967135.1 | metal ion binding protein | 6.57E-26 | 96.15% | 635 |
| L0205 | JT930945.1 | DnaJ protein | 1.08E-140 | 93.80% | 683 |
| L0206 | JR365304.1 | plasminogen activator inhibitor 1 RNA-binding protein | 7.19E-48 | 70.40% | 608 |
| L0207 | JT937925.1 | centromere protein o | 1.03E-24 | 79.50% | 660 |
| L0208 | JT927484.1 | B2 protein | 8.56E-119 | 88.80% | 663 |
| L0209 | JT934802.1 | peptidyl-prolyl cis-trans isomerase nima-interacting 4 | 4.26E-76 | 97.55% | 657 |
| L0210 | JR349921.1 | protein light-dependent short hypocotyls 10-like | 7.50E-107 | 89.25% | 717 |
| L0211 | JT965126.1 | Ribosomal pseudouRidine synthase | 1.16E-75 | 64.70% | 636 |
| L0212 | JT947508.1 | 30S ribosomal protein S13 | 3.71E-78 | 75.15% | 632 |
| L0213 | JT965617.1 | transcription factor ibh1-like | 5.15E-19 | 66.90% | 657 |
| L0214 | JR362520.1 | conserved hypothetical protein* | 3.00E-18 | 77.00% | 697 |
| L0215 | JT968625.1 | NAC domain protein | 1.17E-58 | 70.25% | 581 |
| L0216 | JT917659.1 | oxysterol-binding protein 1d-like | 3.43E-43 | 73.45% | 637 |
| L0217 | JR364433.1 | uncharacterized Serine-rich protein | 3.64E-15 | 77.70% | 625 |
| L0218 | JT970974.1 | hypothetical protein CICLE_v10006890mg, partial | 6.10E-41 | 57.10% | 609 |
| L0219 | JT945278.1 | mip18 family protein at1g68310 | 4.44E-11 | 83.65% | 307 |
| L0220 | JT942672.1 | scarecrow-like transcription factor isoform 1 | 1.48E-39 | 68.65% | 307 |
| L0221 | JT931073.1 | cytochrome P450 cyp736a12-like | 1.09E-17 | 88.30% | 313 |
| L0222 | JT936402.1 | NAC domain-containing protein 8-like | 1.47E-138 | 83.80% | 466 |
| L0223 | JT915340.1 | o-glycosyl hydrolases family 17 isoform partial | 9.46E-44 | 67.50% | 591 |
| L0224 | JR349286.1 | structural constituent of ribosome | 9.54E-30 | 61.70% | 627 |
| L0225 | JT914585.1 | ATP-dependent RNA | 2.00E-63 | 85.70% | 682 |
| L0226 | JR365712.1 | coproporphyrinogen-III aerobic | 1.75E-27 | 90.25% | 236 |
| L0227 | JT914260.1 | piezo-type mechanosensitive ion channel homolog isoform x1 | 7.14E-63 | 87.00% | 512 |
| L0228 | JT953895.1 | subtilisin-like protease | 6.22E-56 | 79.65% | 676 |
| L0229 | JT917992.1 | Serine/threonine-protein kinase EDR1 isoform X1 | 1.14E-38 | 84.20% | 638 |
| L0230 | JT944259.1 | desi-like protein at4g17486 isoform x1 | 1.72E-90 | 89.30% | 639 |
| L0231 | JR345975.1 | polyadenylate-binding protein-interacting protein 3-like | 1.16E-53 | 83.90% | 668 |
| L0232 | JT923391.1 | oxysterol-binding protein 1c-like isoform x1 | 4.95E-29 | 91.70% | 632 |
| L0233 |  | plant f1m20-13 protein | 2.78E-58 | 84.95% | 643 |
| L0234 | JT966423.1 | histone H4 | 8.00E-47 | 99.30% | 597 |
| L0235 | JR349846.1 | lil3 family protein | 2.70E-126 | 83.30% | 655 |
| L0236 |  | casbene chloroplast | 1.14E-78 | 71.80% | 660 |
| L0237 | JT943453.1 | chromophore lyase 3 | 1.91E-121 | 93.30% | 672 |
| L0238 | JT917330.1 | polyadenylate-binding protein-interacting protein 7 | 2.15E-113 | 91.15% | 675 |
| L0239 | JT948340.1 | zinc finger A20 and AN1 domain-containing stress-associated protein 8 | 5.63E-93 | 84.75% | 471 |
| L0240 | JT952473.1 | 60S ribosomal protein L24-like | 2.96E-58 | 99.30% | 666 |
| L0241 | JR347026.1 | auxin-binding protein t85 | 1.53E-71 | 88.30% | 658 |
| L0242 |  | hypothetical protein JCGZ_13997 | 3.06E-29 | 67.30% | 598 |
| L0243 | JR350028.1 | polyketide cyclase dehydrase and lipid transport superfamily protein isoform 2 | 3.17E-50 | 84.05% | 621 |
| L0244 | JT948660.1 | general transcription factor IIh subunit 5-like | 1.86E-39 | 95.15% | 615 |
| L0245 | JT934190.1 | transcription factor TGA4 | 8.13E-127 | 88.00% | 683 |
| L0246 | JT936642.1 | NAC domain-containing protein 8-like | 4.48E-121 | 82.70% | 638 |
| L0247 | JT947903.1 | Ubiquitin-associated 2 | 3.04E-92 | 91.80% | 675 |
| L0248 | JT932444.1 | transcription factor bZIP106 | 8.81E-88 | 83.20% | 672 |
| L0249 | JT918900.1 | cyclin-dependent kinase f-4-like isoform x2 | 3.58E-70 | 95.95% | 623 |
| L0250 | JT929945.1 | MYB-like protein x | 8.64E-14 | 80.30% | 519 |
| L0251 | JT967192.1 | uncharacterized loc101209235 | 3.78E-66 | 93.40% | 305 |
| L0252 | JR361974.1 | acylphosphatase family isoform 1 | 2.97E-62 | 78.55% | 645 |
| L0253 | JR366608.1 | alpha beta-hydrolases superfamily protein | 2.61E-116 | 81.75% | 641 |
| L0254 | JT924488.1 | laccase family protein | 1.70E-105 | 92.65% | 644 |
| L0255 | JT918748.1 | cytochrome P450 89a2-like | 2.70E-107 | 83.15% | 653 |
| L0256 | JT971374.1 | suppressor of ty | 3.89E-05 | 80.00% | 245 |
| L0257 | JT952991.1 | rho GDP-dissociation inhibitor 1-like | 3.02E-66 | 78.90% | 649 |
| L0258 | JT980582.1 |  |  |  | 368 |
| L0259 | JT932557.1 | protein red | 2.23E-46 | 84.25% | 500 |
| L0260 | JT924948.1 | triacylglycerol lipase sdp1 | 1.47E-138 | 93.25% | 119 |
| L0261 | JT937153.1 | 5 -adenylylsulfate reductase-like 4 | 2.72E-51 | 83.90% | 518 |
| L0262 | JT928148.1 | dynein heavy chain-like protein pf11_0240-like isoform x1 | 4.66E-58 | 48.85% | 668 |
| L0263 | JT930331.1 | RING finger protein 10 isoform x2 | 3.99E-45 | 68.80% | 383 |
| L0264 | JT952116.1 | gag-pol polyprotein | 2.50E-12 | 70.40% | 295 |
| L0265 | JR355927.1 | Populus trichocarpa clone Pop1-85E10* | 8.00E-34 | 74.00% | 353 |
| L0266 | JT917314.1 | dual specificity protein phosphatase dsp8 | 2.01E-54 | 85.20% | 117 |
| L0267 | JT960014.1 | selenoprotein k | 9.09E-34 | 90.90% | 445 |
| L0268 | JT938973.1 | 26S proteasome non-ATPase regulatory subunit 7 homolog | 1.47E-138 | 95.90% | 270 |
| L0269 | JT947938.1 | xin actin-binding repeat-containing protein | 9.70E-15 | 63.25% | 382 |
| L0270 | JT931850.1 | probable inactive purple acid phosphatase 29 | 1.37E-64 | 88.45% | 442 |
| L0271 | JT928804.1 | actin | 1.47E-138 | 99.30% | 443 |
| L0272 | JT922216.1 | programmed cell death protein 7 | 3.30E-63 | 75.45% | 537 |
| L0273 | JR362096.1 | DNA-3-methyladenine glycosylase | 1.57E-09 | 87.50% | 196 |
| L0274 | JR366480.1 | conserved hypothetical protein | 3.65E-22 | 79.50% | 231 |
| L0275 | JT926682.1 | transcription factor-related family protein* | 3.00E-07 | 78.00% | 359 |
| L0276 | JR350450.1 | beta-amylase 1, chloroplastic-like (LOC105129385)* | 4.00E-13 | 92.00% | 424 |
| L0277 | JT932806.1 | mediator-associated protein 1-like* | 4.00E-22 | 88.00% | 204 |
| L0278 | JT935684.1 | leucine-rich repeat disease resistance protein | 5.10E-29 | 82.20% | 397 |
| L0279 | JT967279.1 | 50S ribosomal protein L18 | 8.40E-36 | 92.70% | 308 |
| L0280 | JR345073.1 | ef-hand calcium binding protein* | 2.00E-08 | 79.00% | 324 |
| L0281 | JT939823.1 | d-3-phosphoglycerate dehydrogenase-like isoform x3 | 1.79E-20 | 81.30% | 206 |
| L0282 | JT974864.1 | golgi alpha-mannosidase II isoform 1 | 8.13E-46 | 70.75% | 529 |
| L0283 | JT915455.1 | copia protein | 3.03E-71 | 87.75% | 531 |
| L0284 | JR349644.1 | mitochondrial pyruvate dehydrogenase kinase isoform 1* | 1.00E-24 | 87.00% | 318 |
| L0285 | JR366283.1 | succinate dehydrogenase assembly factor mitochondrial | 4.91E-24 | 88.80% | 373 |
| L0286 | JT935882.1 | synaptotagmin-2-like isoform x2 | 3.20E-88 | 90.65% | 658 |
| L0287 | JT919265.1 | 14-3-3-like protein d | 1.77E-107 | 93.65% | 633 |
| L0288 | JT951018.1 | rubber elongation factor | 2.12E-07 | 86.50% | 420 |
| L0289 | JR344738.1 | n-alpha-acetyltransferase 11-like | 7.85E-22 | 99.25% | 214 |
| L0290 | JT948720.1 | probable dolichyl-diphosphooligosaccharide--protein glycosyltransferase subunit 3B | 2.25E-67 | 90.70% | 507 |
| L0291 |  | cis-prenyltransferase | 1.25E-49 | 90.20% | 413 |
| L0292 | JT939873.1 | 2-methyl-6-geranylgeranylbenzoquinone methyltranferase | 7.88E-53 | 78.45% | 556 |
| L0293 | JR349705.1 | eukaryotic release factor 1 family protein isoform 1* | 1.00E-26 | 83.00% | 175 |
| L0294 | JT948170.1 | lupus la ribonucleoprotein* | 1.00E-20 | 91.00% | 144 |
| L0295 | JT945567.1 | hypothetical protein POPTR_0011s15670g | 2.09E-47 | 60.60% | 535 |
| L0296 | JT939262.1 | RING finger protein 214 isoform 1 | 1.29E-95 | 89.15% | 610 |
| L0297 | JR361778.1 | conserved hypothetical protein | 2.53E-25 | 76.25% | 144 |
| L0298 | JT953189.1 | 40S ribosomal protein S4-like | 2.16E-63 | 95.65% | 578 |
| L0299 | JT948545.1 | conserved hypothetical protein | 1.12E-13 | 76.35% | 131 |
| L0300 | JT944793.1 | thioredoxin-related family protein | 5.82E-53 | 69.70% | 633 |
| L0301 | JR366359.1 | oligouridylate-binding protein 1b-like | 1.27E-121 | 93.05% | 538 |
| L0302 | JT943231.1 | er membrane protein complex subunit 3-like | 6.58E-93 | 95.80% | 514 |
| L0303 | JT962334.1 | conserved hypothetical protein | 7.75E-15 | 69.00% | 486 |
| L0304 | JT931582.1 | 26S proteasome non-ATPase regulatory subunit 4 homolog | 2.24E-58 | 90.65% | 504 |
| L0305 | JT937794.1 | aquaporin(PIP1;1) | 1.80E-90 | 91.55% | 527 |
| L0306 | JR348980.1 | DNA binding protein* | 6.00E-18 | 80.00% | 147 |
| L0307 | JT942785.1 | indole-3-acetic acid-induced protein arg7-like | 1.83E-07 | 71.64% | 362 |
| L0308 | JT952343.1 | pyruvate kinase family protein | 4.25E-87 | 97.00% | 419 |
| L0309 | JT939838.1 | glycosyl hydrolase family 9 | 4.10E-100 | 91.35% | 521 |
| L0310 | JT940024.1 | traf-like family protein | 2.67E-31 | 68.60% | 525 |
| L0311 | JR346939.1 | conserved hypothetical protein* | 6.00E-26 | 87.00% | 191 |
| L0312 | JR361078.1 | calcium-dependent lipid-binding family protein | 2.02E-71 | 84.55% | 467 |
| L0313 | JT916265.1 | tmv resistance protein n | 3.77E-20 | 58.65% | 442 |
| L0314 | JR365612.1 | 40S ribosomal S8 | 4.40E-34 | 93.75% | 453 |
| L0315 | JR364672.1 | hypothetical protein EUTSA_v10026968mg | 8.05E-05 | 73.00% | 242 |
| L0316 | JT932810.1 | thiosulfate 3-mercaptopyruvate sulfurtransferase mitochondrial-like | 4.14E-100 | 91.50% | 513 |
| L0317 | JT943154.1 | phosphoglycerate kinase | 3.44E-36 | 77.65% | 506 |
| L0318 | JR366627.1 | protein light-dependent short hypocotyls 1-like | 1.34E-42 | 85.10% | 474 |
| L0319 | JT943847.1 | holocarboxylase synthetase | 3.48E-47 | 83.20% | 548 |
| L0320 | JT968795.1 | histone H1 subtype 5 | 3.45E-21 | 89.85% | 481 |
| L0321 | JT936875.1 | telomere repeat-binding factor 2 | 4.33E-58 | 89.35% | 519 |
| L0322 | JT936109.1 | haloacid dehalogenase-like hydrolase superfamily protein | 7.18E-78 | 85.55% | 515 |
| L0323 | JT936498.1 | lanc-like protein gcl1 | 1.14E-74 | 89.40% | 444 |
| L0324 |  | conserved hypothetical protein | 4.74E-66 | 88.85% | 401 |
| L0325 | JR348086.1 | eukaryotic translation initiation factor 3 | 3.33E-06 | 95.60% | 300 |
| L0326 | JR359562.1 |  |  |  | 247 |
| L0327 | JT942714.1 | mesoderm induction early response protein isoform 1 | 3.97E-37 | 87.90% | 365 |
| L0328 | JR366021.1 | hypothetical protein JCGZ_09365 | 2.79E-06 | 61.67% | 496 |
| L0329 | JT940118.1 | ethylene-responsive transcription factor-like protein at4g13040 isoform x1 | 5.73E-20 | 73.60% | 398 |
| L0330 | JT978122.1 |  |  |  | 461 |
| L0331 | JT942435.1 | calcium-binding ef hand family protein | 2.49E-92 | 89.10% | 549 |
| L0332 | JR348608.1 | DNA-directed RNA polymerases IV and V subunit 3 | 7.60E-110 | 96.80% | 489 |
| L0333 | JR346803.1 | Exostosin 2 | 4.00E-13 | 71.00% | 391 |
| L0334 | JT961724.1 | Ubiquinol-cytochrome c reductase complex 6.7 kDa protein | 2.43E-15 | 74.85% | 341 |
| L0335 | JT977880.1 | Stem-specific protein TSJT1 | 9.09E-39 | 89.60% | 318 |
| L0336 | JT942058.1 | haloacid dehalogenase-like hydrolase superfamily protein | 4.53E-26 | 86.10% | 436 |
| L0337 | JR350055.1 | kinase family protein | 1.92E-19 | 71.63% | 367 |
| L0338 | JT947714.1 | V-type proton ATPase 16 kDa proteolipid subunit | 5.13E-49 | 98.95% | 525 |
| L0339 | JT946713.1 | Tetraspanin family protein (TRN2) | 2.56E-94 | 95.15% | 524 |
| L0340 | JT954001.1 | PRA1 family protein f3-like | 6.21E-73 | 85.15% | 169 |
| L0341 | JR345770.1 | protein vip1 isoform x1 | 1.24E-44 | 89.95% | 530 |
| L0342 | JT923651.1 | rpm1 interacting protein isoform 1 | 1.47E-07 | 56.80% | 521 |
| L0343 | JT930539.1 | actin partial | 9.25E-41 | 100.00% | 199 |
| L0344 | JT958848.1 | conserved hypothetical protein | 1.48E-19 | 74.05% | 366 |
| L0345 | JT961839.1 | RING-H2 finger protein atl48-like | 5.48E-47 | 92.25% | 526 |
| L0346 | JT955693.1 |  |  |  | 485 |
| L0347 | JT941285.1 | translocase of chloroplast chloroplastic isoform x2 | 1.67E-67 | 91.10% | 409 |
| L0348 | JT972443.1 | ATP-dependent DNA helicase 2 subunit ku80-like isoform x1 | 3.47E-60 | 92.15% | 549 |
| L0349 | JT942172.1 | U-box domain-containing protein 38 | 3.50E-85 | 76.50% | 495 |
| L0350 | JR346027.1 | Populus euphratica DNA-dependent metalloprotease WSS1* | 1.00E-20 | 88.00% | 405 |
| L0351 | JT950494.1 | upf0664 stress-induced protein | 2.80E-80 | 89.35% | 569 |
| L0352 | JT941833.1 | mitochondrial succinate-fumarate transporter 1-like | 1.19E-58 | 93.80% | 545 |
| L0353 | JT952933.1 | brain acid soluble protein 1 homolog | 1.60E-79 | 83.40% | 164 |
| L0354 | JT970706.1 |  |  |  | 319 |
| L0355 | JT938857.1 | CFE protein | 1.74E-68 | 67.65% | 581 |
| L0356 | JT919260.1 | cyclin family protein | 2.93E-29 | 86.50% | 462 |
| L0357 | JT939141.1 | Biotin carboxyl carrier protein of acetyl-CoA carboxylase | 4.78E-25 | 96.40% | 531 |
| L0358 | JT914440.1 | eukaryotic translation initiation factor 4g | 6.37E-74 | 88.70% | 532 |
| L0359 | JT960025.1 | Ribosomal L18p/L6e family protein | 1.31E-76 | 93.60% | 533 |
| L0360 | JT936813.1 | double-stranded RNA-binding protein 4 isoform x1 | 1.23E-118 | 68.20% | 354 |
| L0361 | JT977403.1 |  |  |  | 576 |
| L0362 | JT977074.1 | transmembrane protein 14* | 3.00E-10 | 78.00% | 492 |
| L0363 | JT964633.1 | hypothetical protein JCGZ_24748 | 9.97E-36 | 84.00% | 615 |
| L0364 | JT949182.1 | partner of Y14 and mago-like | 1.01E-89 | 79.90% | 583 |
| L0365 | JR348697.1 | Bidirectional sugar transporter SWEET12 | 4.44E-37 | 97.85% | 381 |
| L0366 | JR348559.1 | methionine aminopeptidase 1a | 4.74E-68 | 85.65% | 565 |
| L0367 | JR366267.1 | homeobox transcription factor | 3.46E-48 | 77.40% | 580 |
| L0368 | JT932567.1 | homeodomain-like superfamily | 1.20E-05 | 72.88% | 551 |
| L0369 | JT956106.1 |  |  |  | 315 |
| L0370 | JT976134.1 | rubber elongation factor* | 1.00E-42 | 81.00% | 270 |
| L0371 | JT975736.1 |  |  |  | 115 |
| L0372 | JT937638.1 | casein kinase II subunit alpha-2-like | 1.20E-61 | 92.40% | 607 |
| L0373 | JT946301.1 | chromatin structure-remodeling complex subunit rsc1-like | 1.16E-114 | 96.45% | 624 |
| L0374 | JT959563.1 | 14-3-3-like protein | 7.93E-64 | 96.85% | 451 |
| L0375 | JT957749.1 | cupin domain-containing protein | 1.97E-46 | 89.75% | 488 |
| L0376 | JT918520.1 | phospholipase D (PLDa) | 3.05E-20 | 97.40% | 140 |
| L0377 | JT980823.1 |  |  |  | 210 |
| L0378 | JT915651.1 | Munc13-like protein PATROL1 | 2.24E-101 | 86.80% | 581 |
| L0379 |  | hypothetical protein JCGZ_04706 | 6.96E-07 | 92.00% | 625 |
| L0380 | JT945417.1 | zinc finger family protein | 8.73E-93 | 83.80% | 606 |
| L0381 | JT924607.1 | E3 ubiquitin-protein ligase RLIM-like | 4.64E-52 | 82.10% | 443 |
| L0382 |  | Serine-threonine protein plant-type | 1.12E-38 | 68.11% | 450 |
| L0383 | JT932391.1 | probable inactive poly[ADP-ribose] polymerase SRO3 | 6.68E-60 | 73.95% | 495 |
| L0384 | JT932109.1 | Serine-threonine protein plant-type | 3.28E-78 | 72.75% | 535 |
| L0385 | JT954824.1 | ribonuclease 3 family protein | 9.80E-89 | 73.65% | 689 |
| L0386 | JT966564.1 | nitrogen regulatory protein p-II homolog | 9.29E-57 | 95.65% | 578 |
| L0387 | JT951307.1 |  |  |  | 624 |
| L0388 | JR362933.1 | Aspartic proteinase nepenthesin-1 precursor | 7.30E-81 | 94.50% | 569 |
| L0389 | JT941725.1 | transcriptional regulator atrx homolog isoform x1 | 1.22E-88 | 72.35% | 600 |
| L0390 | JT947766.1 | intracellular protease 1 | 9.68E-109 | 89.35% | 555 |
| L0391 | JT923243.1 | 60S ribosomal protein L37-1-like | 8.70E-16 | 65.95% | 541 |
| L0392 | JT946896.1 | stress response protein nst1-like | 2.26E-29 | 81.65% | 553 |
| L0393 | JT950334.1 | DNA-directed RNA polymerase I subunit rpa43-like | 2.25E-54 | 79.25% | 546 |
| L0394 | JT920086.1 | Serine threonine-protein kinase d6pkl1 | 1.61E-63 | 95.15% | 476 |
| L0395 | JT952444.1 | n-acetyltransferase 9-like protein | 1.97E-67 | 84.15% | 588 |
| L0396 | JT935456.1 | bZIP domain class transcription factor | 6.51E-75 | 89.65% | 512 |
| L0397 | JT931010.1 | UDP-arabinose 4-epimerase 1 | 8.54E-97 | 93.40% | 531 |
| L0398 | JT944714.1 | two-pore potassium channel 1-like isoform x1 | 8.05E-73 | 88.05% | 497 |
| L0399 | JT928683.1 | mediator-associated protein 1-like | 3.63E-36 | 79.35% | 545 |
| L0400 | JT961349.1 | Ubiquitin carboxyl-terminal hydrolase 12-like isoform x2 | 3.60E-28 | 93.40% | 252 |
| L0401 | JT943362.1 | proteasome subunit beta type-1 | 1.46E-97 | 97.95% | 493 |
| L0402 | JT936700.1 | DNA-directed RNA polymerases IV and V subunit 8b-like | 7.24E-90 | 91.55% | 580 |
| L0403 | JT943089.1 |  |  |  | 479 |
| L0404 | JT921674.1 | kinase superfamily protein isoform 1 | 1.71E-96 | 83.15% | 517 |
| L0405 | JT947147.1 | sbp family protein | 4.19E-47 | 63.10% | 498 |
| L0406 | JT920432.1 | ultraviolet-B receptor UVR8 (LOC101310374), transcript variant X1* | 4.00E-20 | 76.00% | 436 |
| L0407 | JT945196.1 | exonuclease chloroplastic/mitochondrial | 6.66E-107 | 77.00% | 599 |
| L0408 | JT942083.1 | 14-3-3-like protein gf14 kappa isoform x1 | 3.21E-80 | 97.15% | 494 |
| L0409 | JT938507.1 | annexin d4 | 1.55E-82 | 84.20% | 471 |
| L0410 | JR348033.1 | protein light-dependent short hypocotyls 4 | 1.00E-46 | 97.20% | 417 |
| L0411 | JT955791.1 |  |  |  | 556 |
| L0412 | JT941145.1 | very-long-chain 3-oxoacyl- reductase 1-like | 2.68E-76 | 81.80% | 573 |
| L0413 | JT965228.1 | mitochondrial import inner membrane translocase subunit Tim10 | 1.09E-55 | 82.60% | 381 |
| L0414 | JT924195.1 | f-box protein fbw2-like | 4.63E-60 | 81.40% | 397 |
| L0415 | JT972829.1 | DNA-binding family protein | 1.12E-29 | 95.45% | 324 |
| L0416 | JT916598.1 | LRR receptor-like Serine threonine-protein kinase rch1 | 2.99E-57 | 86.90% | 518 |
| L0417 | JT956666.1 | thioredoxin-like protein yls8 | 3.67E-96 | 99.80% | 499 |
| L0418 | JR357156.1 | hypothetical protein JCGZ_06668 | 4.70E-33 | 77.00% | 471 |
| L0419 | JT940900.1 | salt overly sensitive protein 2a isoform 1 | 4.81E-101 | 90.65% | 520 |
| L0420 | JT955812.1 | hypothetical protein JCGZ_07921 | 3.06E-09 | 68.70% | 569 |
| L0421 | JT953405.1 | exosome complex component csl4 | 1.93E-51 | 78.10% | 416 |
| L0422 | JT944837.1 | conserved hypothetical protein | 5.39E-15 | 73.47% | 477 |
| L0423 | JT922912.1 | ankyrin repeat-containing protein at2g01680-like | 2.14E-48 | 92.70% | 361 |
| L0424 | JT945539.1 | nuclear transport factor 2 family protein isoform 1 | 2.43E-73 | 74.70% | 592 |
| L0425 | JT963585.1 | Ubiquitin-like protein 5 | 3.16E-40 | 98.30% | 237 |
| L0426 | JT955341.1 | protein translation factor sui1 homolog | 1.12E-76 | 97.15% | 513 |
| L0427 | JT961894.1 |  |  |  | 415 |
| L0428 |  | homeobox protein knotted-1-like 6 | 3.18E-55 | 90.20% | 443 |
| L0429 | JT939578.1 | Anamorsin homolog | 9.17E-17 | 77.80% | 449 |
| L0430 | JR365829.1 | conserved hypothetical protein* | 5.00E-08 | 69.00% | 199 |
| L0431 | JT927872.1 | protein mos2 | 5.78E-13 | 70.80% | 475 |
| L0432 | JR365857.1 | hemiasterlin resistant protein 1 | 9.10E-10 | 72.89% | 529 |
| L0433 | JT971783.1 | Ubiquitin extension protein 1 | 3.28E-32 | 99.20% | 339 |
| L0434 | JT971116.1 | avr9 cf-9 rapidly elicited protein | 6.71E-11 | 55.33% | 363 |
| L0435 | JR358522.1 | coiled-coil-helix-coiled-coil-helix domain-containing protein 2, mitochondrial-like (LOC105110020)* | 5.00E-73 | 84.00% | 272 |
| L0436 | JT920541.1 | protein yls7-like | 1.11E-64 | 90.75% | 340 |
| L0437 | JT958324.1 | heavy metal-associated isoprenylated plant protein 26-like | 5.21E-63 | 95.05% | 595 |
| L0438 | JT951414.1 | protein farnesyltransferase geranylgeranyltransferase type-1 subunit alpha | 3.23E-62 | 80.05% | 563 |
| L0439 | JT918568.1 | proton pump-interactor 1-like | 7.27E-76 | 77.90% | 570 |
| L0440 | JT917619.1 | pleckstrin homology domain-containing family protein | 1.30E-85 | 95.20% | 573 |
| L0441 | JT923071.1 | conserved hypothetical protein | 2.26E-05 | 74.00% | 283 |
| L0442 | JT914930.1 | kinase family protein | 6.61E-08 | 71.50% | 472 |
| L0443 | JT918894.1 | major facilitator superfamily protein | 2.89E-39 | 93.85% | 246 |
| L0444 | JT949034.1 | protein CutA, chloroplastic | 5.30E-95 | 88.15% | 486 |
| L0445 | JT936380.1 | Ribosomal protein S14 | 3.42E-51 | 89.45% | 531 |
| L0446 | JT955167.1 | mediator of RNA polymerase II transcription subunit 19a-like isoform x1 | 1.48E-16 | 68.95% | 451 |
| L0447 | JT950737.1 | protein n-terminal glutamine amidohydrolase | 9.39E-75 | 87.60% | 381 |
| L0448 | JT935206.1 | biotin protein ligase | 2.94E-22 | 65.15% | 622 |
| L0449 | JT968984.1 | disease resistance protein RGA3 | 5.44E-11 | 56.15% | 589 |
| L0450 | JT926728.1 | sirohydrochlorin ferrochelatase | 1.14E-60 | 88.45% | 504 |
| L0451 | JT932267.1 | Serine threonine-protein kinase ht1-like | 5.20E-20 | 91.15% | 475 |
| L0452 | JT943594.1 | NADH dehydrogenase | 5.75E-30 | 78.35% | 579 |
| L0453 | JT932066.1 | adenylyl cyclase-associated protein | 7.01E-86 | 84.60% | 535 |
| L0454 | JT957636.1 | nudix family protein | 1.21E-10 | 67.35% | 565 |
| L0455 | JT928452.1 | bi1-like protein | 2.51E-46 | 92.35% | 637 |
| L0456 | JR344315.1 | glutathione s-transferase u17-like | 6.41E-39 | 83.90% | 240 |
| L0457 | JR365945.1 | ethylene-inducible protein (ER1) | 9.90E-55 | 97.45% | 558 |
| L0458 | JT939709.1 | calcineurin-like metallo-phosphoesterase superfamily | 1.67E-74 | 72.75% | 552 |
| L0459 | JT930276.1 | mitochondrial metalloendopeptidase oma1 | 9.26E-110 | 88.75% | 538 |
| L0460 | JT921838.1 | senescence-associated protein | 4.79E-20 | 95.71% | 159 |
| L0461 | JT941386.1 | GTP-binding protein | 2.87E-12 | 93.10% | 147 |
| L0462 | JT939139.1 | conserved hypothetical protein | 3.65E-13 | 73.70% | 283 |
| L0463 | JR364022.1 |  |  |  | 313 |
| L0464 | JT963567.1 | 60S ribosomal protein L18a-like protein | 1.21E-26 | 82.85% | 510 |
| L0465 | JT942498.1 | DNA-directed RNA polymerases I and III subunit rpac1 | 1.75E-45 | 95.05% | 455 |
| L0466 | JT975467.1 | polyadenylate-binding protein 2-like (LOC102620020)* | 2.00E-48 | 91.00% | 454 |
| L0467 | JR350034.1 | xyloglucan galactosyltransferase | 1.24E-17 | 69.50% | 525 |
| L0468 | JT917737.1 | erd1 family protein | 8.22E-108 | 94.55% | 515 |
| L0469 | JT936924.1 | probable inactive receptor kinase at2g26730 | 1.19E-107 | 96.45% | 562 |
| L0470 | JR355608.1 | mitochondrial import inner membrane translocase subunit Tim17-2-like | 2.75E-15 | 84.37% | 124 |
| L0471 | JT927189.1 | exocyst complex component exo84b | 7.90E-15 | 90.35% | 345 |
| L0472 | JT932122.1 | telomere repeat-binding protein 3-like | 6.41E-18 | 78.20% | 467 |
| L0473 | JT925478.1 | pleckstrin homology domain-containing family protein | 9.34E-41 | 82.85% | 587 |
| L0474 | JT922501.1 | heat shock protein 70 | 6.17E-23 | 85.25% | 489 |
| L0475 | JT923350.1 | probable galacturonosyltransferase 10-like | 8.93E-19 | 92.80% | 278 |
| L0476 | JT953425.1 | transmembrane protein 258-like | 6.86E-24 | 93.90% | 493 |
| L0477 | JT946525.1 | 60S ribosomal protein L13-1 | 1.99E-106 | 93.75% | 596 |
| L0478 | JT955288.1 | phosphopantetheine adenylyltransferase | 2.00E-67 | 92.05% | 362 |
| L0479 | JT920486.1 | histone acetyltransferase kat6b-like | 9.63E-13 | 62.35% | 503 |
| L0480 | JT952914.1 | Aspartic proteinase-like protein 1 | 6.56E-45 | 86.60% | 264 |
| L0481 | JR362250.1 | probable protein Pop3 | 1.86E-45 | 89.10% | 344 |
| L0482 | JT947163.1 | nucleic acid binding protein | 1.16E-35 | 60.80% | 426 |
| L0483 | JT920217.1 | uncharacterized membrane protein at1g75140-like | 4.14E-10 | 80.75% | 250 |
| L0484 | JT926229.1 | 2-C-methyl-D-erythritol 4-phosphate cytidylyltransferase | 6.81E-16 | 88.70% | 269 |
| L0485 | JR357020.1 | squamosa promoter-binding-like protein 3 (LOC105138885)* | 1.00E-06 | 88.00% | 123 |
| L0486 | JR346303.1 | vq motif-containing family protein | 2.69E-08 | 69.50% | 625 |
| L0487 | JT947214.1 | ethylene-responsive transcription factor 4-like | 7.87E-60 | 84.05% | 465 |
| L0488 | JR363912.1 | cytochrome c oxidase subunit mitochondrial-like | 1.62E-50 | 80.75% | 636 |
| L0489 | JR345338.1 | 40S ribosomal protein S26 | 2.63E-48 | 96.10% | 388 |
| L0490 | JT929970.1 | beta-ureidopropionase | 6.07E-07 | 92.30% | 181 |
| L0491 | JT915443.1 | FACT complex subunit spt16-like | 4.05E-88 | 91.70% | 519 |
| L0492 | JT925080.1 | nodulin family protein | 1.76E-65 | 92.20% | 598 |
| L0493 | JT951485.1 | gck domain-containing protein | 1.22E-46 | 81.65% | 645 |
| L0494 | JT937623.1 | translocon at the inner envelope membrane of chloroplasts 20 isoform 1 | 2.01E-88 | 96.85% | 571 |
| L0495 | JR364842.1 | conserved hypothetical protein | 3.69E-32 | 70.45% | 606 |
| L0496 | JT924854.1 | calmodulin binding protein | 8.15E-57 | 91.80% | 496 |
| L0497 | JR365035.1 | mitochondrial inner membrane organizing system 1 | 5.57E-41 | 84.00% | 532 |
| L0498 | JT973488.1 |  |  |  | 488 |
| L0499 | JT939894.1 | gdt1-like protein 4 | 4.76E-24 | 98.45% | 397 |
| L0500 | JR359103.1 | glyceraldehyde 3-phosphate dehydrogenase 1 family protein | 1.45E-05 | 69.00% | 515 |
| L0501 |  | 1-aminocyclopropane-1-carboxylate oxidase homolog 4-like | 2.80E-42 | 75.30% | 531 |
| L0502 | JR364606.1 | PI-PLC X domain-containing protein At5g67130-like* | 9.00E-42 | 74.00% | 517 |
| L0503 | JT959552.1 | 40S ribosomal protein S11-like | 5.82E-58 | 91.95% | 555 |
| L0504 | JT958148.1 | HVA22-like protein e isoform x2 | 4.20E-12 | 69.22% | 570 |
| L0505 | JT942882.1 | ADP-ribosylation factor | 8.68E-104 | 99.85% | 572 |
| L0506 | JT942847.1 | caffeic acid o-methyltransferase | 8.01E-77 | 91.55% | 387 |
| L0507 | JT980272.1 | hypothetical protein JCGZ_06119 | 1.73E-05 | 73.00% | 496 |
| L0508 |  | protein yippee-like at4g27745 | 6.15E-63 | 86.65% | 576 |
| L0509 | JT938014.1 | eukaryotic translation initiation factor 2 subunit alpha-like | 6.24E-93 | 95.50% | 460 |
| L0510 | JT967185.1 | trigger factor | 1.80E-11 | 56.80% | 469 |
| L0511 | JT964055.1 | DUF21 domain-containing protein at2g14520 | 2.25E-14 | 93.45% | 574 |
| L0512 | JT971320.1 | methionine aminopeptidase 1a-like | 8.59E-17 | 87.20% | 549 |
| L0513 | JT927580.1 | zinc finger CCCH domain-containing protein 69 isoform x1 | 8.58E-31 | 66.75% | 517 |
| L0514 | JT936721.1 | ankyrin repeat family protein | 1.15E-93 | 90.25% | 446 |
| L0515 | JT965502.1 | 40S ribosomal protein S15a-5-like | 4.77E-35 | 93.80% | 460 |
| L0516 | JT935084.1 | 2-phosphoglycerate kinase-related family protein | 4.21E-95 | 82.45% | 542 |
| L0517 | JR352793.1 |  |  |  | 112 |
| L0518 | JT949216.1 | uncharacterized protein isoform 1 | 8.30E-25 | 92.05% | 562 |
| L0519 | JT961204.1 | cytochrome b-c1 complex subunit 6 | 4.37E-36 | 90.50% | 567 |
| L0520 | JT932292.1 | rhodanese-like domain-containing protein 7 | 1.58E-26 | 93.90% | 516 |
| L0521 | JT944919.1 | succinate dehydrogenase 5 | 8.99E-72 | 70.15% | 510 |
| L0522 | JT946872.1 | b-cell receptor-associated protein 31 | 1.32E-09 | 74.10% | 551 |
| L0523 | JT918036.1 | yth domain-containing protein | 8.97E-15 | 75.90% | 409 |
| L0524 | JT941111.1 | glyceraldehyde-3-phosphate dehydrogenase | 6.58E-27 | 97.05% | 313 |
| L0525 | JT919694.1 | conserved hypothetical protein | 9.67E-39 | 94.15% | 590 |
| L0526 | JR359887.1 | non-functional NADPH-dependent codeinone reductase 2-like | 4.59E-75 | 84.50% | 565 |
| L0527 | JR349593.1 | 26S proteasome non-ATPase regulatory subunit 10 | 8.66E-28 | 92.05% | 416 |
| L0528 | JT963312.1 | profilin 3 family protein | 5.00E-32 | 95.30% | 557 |
| L0529 | JT952011.1 | 50S ribosomal protein chloroplastic-like | 1.77E-22 | 68.75% | 379 |
| L0530 |  | protein translation factor sui1 homolog | 1.21E-11 | 58.70% | 611 |
| L0531 | JR361861.1 | acyl-CoA-binding protein | 4.78E-42 | 97.30% | 244 |
| L0532 | JR348853.1 | hypothetical protein JCGZ_05827 | 1.42E-40 | 91.65% | 615 |
| L0533 | JR345671.1 | calmodulin | 2.65E-76 | 99.35% | 644 |
| L0534 | JT940163.1 | bZIP transcription factor 60-like | 1.74E-50 | 73.25% | 665 |
| L0535 | JT954184.1 | 40S ribosomal protein S14-like | 7.77E-46 | 96.95% | 531 |
| L0536 | JR365586.1 | NFU1 iron-sulfur mitochondrial | 1.15E-71 | 74.10% | 663 |
| L0537 | JT965241.1 |  |  |  | 569 |
| L0538 | JT962713.1 | protein-s-isoprenylcysteine o-methyltransferase a-like | 2.09E-72 | 87.20% | 583 |
| L0539 | JT970382.1 | protein mos2-like | 5.27E-16 | 70.90% | 481 |
| L0540 | JT932382.1 | nucleic acid binding protein | 1.08E-63 | 90.50% | 514 |
| L0541 | JT916649.1 | inositol transporter 1 like protein | 3.09E-21 | 91.65% | 569 |
| L0542 | JT962677.1 | hypothetical protein POPTR_0018s06230g | 9.68E-14 | 78.81% | 624 |
| L0543 | JR364380.1 | conserved hypothetical protein | 6.77E-67 | 85.95% | 591 |
| L0544 | JT937424.1 | DNA binding protein | 2.30E-43 | 87.80% | 493 |
| L0545 | JT931562.1 | probable dihydrofolate synthetase | 1.61E-49 | 85.75% | 541 |
| L0546 | JT917724.1 | delta-aminolevulinic acid dehydratase chloroplastic | 2.04E-60 | 84.10% | 574 |
| L0547 | JR358420.1 |  |  |  | 609 |
| L0548 | JT941863.1 | C2 domain-containing family protein | 2.16E-70 | 79.25% | 504 |
| L0549 | JT948143.1 | 60S ribosomal protein L23 | 3.81E-66 | 99.95% | 455 |
| L0550 | JT955888.1 | upf0587 protein c1orf123 homolog | 4.44E-45 | 87.20% | 524 |
| L0551 | JR361144.1 | double-stranded RNA binding protrin | 3.67E-29 | 55.67% | 510 |
| L0552 | JT953860.1 | ATP synthase delta chloroplastic | 3.77E-49 | 61.55% | 532 |
| L0553 | JR364043.1 | transmembrane protein 230-like | 2.01E-52 | 93.85% | 513 |
| L0554 | JT959030.1 | probable 26S proteasome complex subunit sem1-1 | 6.74E-08 | 91.60% | 635 |
| L0555 | JT972263.1 |  |  |  | 550 |
| L0556 | JT964512.1 | 50S ribosomal protein chloroplastic | 8.04E-17 | 74.05% | 463 |
| L0557 | JR353585.1 |  |  |  | 578 |
| L0558 | JT958921.1 | prefoldin subunit 1 | 2.20E-80 | 93.30% | 627 |
| L0559 | JT956830.1 | RNA binding protein | 1.16E-50 | 75.80% | 535 |
| L0560 | JT973065.1 | programmed cell death protein 4-like | 2.39E-35 | 77.85% | 585 |
| L0561 | JT919477.1 | RNA polymerase-associated protein rtf1 homolog | 1.02E-33 | 91.60% | 643 |
| L0562 | JT977462.1 |  |  |  | 533 |
| L0563 | JT932187.1 | universal stress family protein | 1.03E-45 | 95.10% | 731 |
| L0564 | JT921020.1 | mediator of RNA polymerase II transcription subunit 12 | 1.90E-58 | 53.80% | 601 |
| L0565 | JT924877.1 | conserved hypothetical protein | 1.47E-57 | 66.25% | 548 |
| L0566 | JT932542.1 | origin recognition complex subunit 2 | 5.24E-52 | 81.60% | 532 |
| L0567 | JT966885.1 | hypothetical protein JCGZ_04890 | 5.12E-13 | 66.25% | 631 |
| L0568 | JT945900.1 | Serine arginine-rich splicing factor sr34a-like isoform x1 | 1.90E-50 | 95.35% | 568 |
| L0569 | JT945744.1 | casein kinase I isoform delta-like | 8.96E-75 | 77.45% | 464 |
| L0570 | JT971674.1 | acetyltransferase at1g77540-like | 7.23E-57 | 90.65% | 574 |
| L0571 | JT926311.1 | squalene monooxygenase | 3.74E-16 | 86.35% | 471 |
| L0572 | JT915337.1 | dentin sialophosphoprotein | 4.96E-32 | 68.76% | 580 |
| L0573 | JT920754.1 | subtilisin precursor family protein | 1.39E-10 | 81.00% | 241 |
| L0574 |  | protein shi related sequence 1-like | 3.29E-79 | 71.35% | 611 |
| L0575 | JT968718.1 | o-fucosyltransferase family protein | 2.65E-18 | 77.45% | 403 |
| L0576 | JT922546.1 | leucine-rich repeat-containing protein | 1.90E-62 | 74.95% | 555 |
| L0577 | JT956517.1 | Thylakoid membrane phosphoprotein 14 kDa, chloroplast precursor | 5.07E-72 | 90.30% | 632 |
| L0578 | JT947899.1 | Ubiquitin-conjugating enzyme e2 2 | 1.53E-108 | 99.60% | 673 |
| L0579 | JT961045.1 |  |  |  | 544 |
| L0580 | JR349424.1 | f-box family protein | 2.04E-53 | 79.20% | 597 |
| L0581 | JT943421.1 | tetraspanin-6-like | 8.63E-89 | 94.30% | 617 |
| L0582 | JT926133.1 | C2 domain-containing protein at1g53590-like | 3.48E-140 | 93.25% | 674 |
| L0583 | JT935751.1 | probable protein phosphatase 2C 13 | 1.73E-14 | 66.06% | 525 |
| L0584 |  | nuclear transcription factor y subunit b-3 | 2.79E-48 | 91.50% | 519 |
| L0585 | JT916231.1 | zinc finger CCCH domain-containing protein 44 | 8.97E-60 | 64.45% | 639 |
| L0586 | JT914399.1 | DDB1- and CUL4-associated factor homolog 1 | 1.03E-99 | 82.70% | 564 |
| L0587 | JT938546.1 | GTP binding protein, putative | 1.55E-76 | 87.10% | 639 |
| L0588 | JT951112.1 | Plasminogen activator inhibitor 1 RNA-binding protein | 6.22E-45 | 82.55% | 641 |
| L0589 | JR348875.1 | protein brassinazole-resistant 1-like | 3.27E-49 | 90.55% | 456 |
| L0590 | JT931966.1 | solute carrier family 25 member 44-like | 2.14E-89 | 90.75% | 502 |
| L0591 | JR366332.1 | conserved hypothetical protein | 4.22E-09 | 68.00% | 381 |
| L0592 | JR364539.1 | NADH dehydrogenase | 4.00E-42 | 90.85% | 530 |
| L0593 | JT938439.1 | mevalonate diphosphate decarboxylase | 1.15E-98 | 91.00% | 557 |
| L0594 | JT918530.1 | exocyst complex component exo70a1-like | 6.28E-41 | 69.25% | 472 |
| L0595 | JT939519.1 | BSD domain-containing family protein | 8.79E-100 | 85.65% | 639 |
| L0596 | JT948869.1 | probable prefoldin subunit 3 | 5.18E-104 | 93.75% | 581 |
| L0597 | JT960665.1 |  |  |  | 525 |
| L0598 | JT954623.1 | Vacuolar ATPase assembly integral membrane protein vma21-like domain isoform 3 | 3.73E-35 | 89.90% | 569 |
| L0599 | JR365052.1 | cytosolic class I small heat shock protein partial | 7.29E-59 | 94.75% | 561 |
| L0600 | JR344781.1 | vesicle-associated protein 1-2-like | 1.04E-53 | 87.95% | 518 |
| L0601 | JT917279.1 | tom1-like protein 2 | 1.07E-36 | 69.35% | 500 |
| L0602 | JT944403.1 | protein s-acyltransferase 8-like | 6.93E-34 | 95.40% | 561 |
| L0603 | JT959825.1 | RNA recognition motif-containing family protein* | 8.00E-48 | 80.00% | 421 |
| L0604 | JT949403.1 | upf0235 protein at5g63440 | 9.48E-69 | 99.65% | 559 |
| L0605 | JT963385.1 | tetratricopeptide repeat 12 | 4.13E-44 | 78.20% | 559 |
| L0606 | JT944740.1 | DNA-directed RNA polymerase III subunit rpc31-like | 3.28E-64 | 73.95% | 550 |
| L0607 | JT955825.1 | nhp2-like protein 1 | 5.34E-75 | 97.80% | 604 |
| L0608 | JT948335.1 | pumilio homolog 2-like | 3.92E-26 | 95.65% | 631 |
| L0609 | JT932092.1 | protein lower cell density 1 | 1.25E-28 | 56.00% | 664 |
| L0610 | JT925632.1 | probable polyamine oxidase 4 | 2.90E-61 | 89.40% | 345 |
| L0611 | JT943170.1 | zinc finger A20 and AN1 domain-containing stress-associated protein 8-like | 2.33E-76 | 74.35% | 466 |
| L0612 | JT952460.1 | conserved hypothetical protein* | 6.00E-14 | 66.00% | 643 |
| L0613 | JT962182.1 | 60S acidic ribosomal protein isoform 1 | 3.24E-29 | 91.05% | 671 |
| L0614 | JT931199.1 | zinc finger family protein | 1.16E-109 | 76.85% | 698 |
| L0615 | JT977712.1 | histone H2A | 3.84E-19 | 100.00% | 242 |
| L0616 | JT959836.1 | ethylene-responsive transcription factor 4 | 2.81E-64 | 59.05% | 575 |
| L0617 | JR365244.1 | hypothetical protein JCGZ_04615 | 1.11E-37 | 87.85% | 385 |
| L0618 | JT959968.1 | f-box protein afr-like | 3.67E-15 | 88.25% | 484 |
| L0619 | JR364110.1 | fiber protein fb15 | 3.93E-45 | 94.80% | 264 |
| L0620 | JR366049.1 | nascent polypeptide-associated complex subunit alpha-like protein 2 | 1.48E-48 | 93.70% | 553 |
| L0621 | JT960057.1 | aminopeptidase-like protein | 8.51E-62 | 90.70% | 656 |
| L0622 | JR365374.1 | 40S ribosomal protein S18 | 9.58E-95 | 97.05% | 484 |
| L0623 | JT967236.1 | conserved hypothetical protein | 6.00E-08 | 75.50% | 642 |
| L0624 | JT928200.1 | glyoxalase 2-1 family protein | 4.63E-58 | 87.00% | 539 |
| L0625 | JT966304.1 | ethylene-responsive transcription factor erf008-like | 5.72E-33 | 58.45% | 536 |
| L0626 | JT922207.1 | uncharacterized membrane protein at1g75140-like | 8.87E-10 | 75.40% | 503 |
| L0627 | JT949931.1 | f-box protein at4g00755 | 3.18E-46 | 67.75% | 559 |
| L0628 | JR344511.1 | probable ubiquitin-conjugating enzyme E2 16-like | 1.58E-101 | 96.60% | 476 |
| L0629 | JR349364.1 | hypothetical protein POPTR_0001s25520g | 1.56E-05 | 85.00% | 544 |
| L0630 | JR349068.1 | vesicle-fusing ATPase | 5.84E-38 | 96.35% | 435 |
| L0631 | JT966779.1 | hypothetical protein JCGZ_07405 | 2.56E-16 | 65.00% | 429 |
| L0632 | JT916668.1 | Serine threonine-protein phosphatase 4 regulatory subunit 3-like | 3.00E-63 | 91.55% | 357 |
| L0633 | JT940463.1 | eukaryotic translation initiation factor 5b-like | 1.54E-43 | 76.45% | 368 |
| L0634 | JT964243.1 | maltase- intestinal | 1.33E-59 | 72.90% | 433 |
| L0635 | JT929150.1 | Protein BFR2 | 1.59E-25 | 93.45% | 425 |
| L0636 | JT952242.1 | Thylakoid membrane phosphoprotein 14 kDa, chloroplast precursor | 1.75E-55 | 85.65% | 573 |
| L0637 |  | poly polymerase | 6.95E-21 | 67.70% | 462 |
| L0638 | JR365466.1 | 14-3-3-like protein b | 6.66E-52 | 96.50% | 309 |
| L0639 |  | ABC transporter G family member 22 | 6.44E-41 | 83.70% | 509 |
| L0640 | JT943898.1 | DNAj homolog subfamily c member 3 homolog | 5.55E-26 | 93.30% | 536 |
| L0641 | JT939811.1 | nuclear transcription factor y subunit c-9-like | 1.07E-14 | 66.80% | 670 |
| L0642 | JR366705.1 | succinyl-CoA synthetase beta chain | 3.38E-40 | 96.65% | 546 |
| L0643 | JT942199.1 | 29 kDa ribonucleoprotein chloroplastic-like | 2.05E-31 | 94.10% | 583 |
| L0644 | JT945427.1 | 50S ribosomal protein L15 | 7.73E-68 | 78.45% | 451 |
| L0645 | JT945036.1 | at4g33625-like protein | 3.54E-15 | 88.15% | 429 |
| L0646 | JT950805.1 | 60S ribosomal protein L17-2-like | 2.26E-112 | 96.70% | 541 |
| L0647 | JT974391.1 | U-box domain-containing protein 62-like | 7.78E-27 | 89.80% | 515 |
| L0648 | JT959039.1 | Ribosomal protein S21 family protein isoform 1 | 4.29E-27 | 91.55% | 570 |
| L0649 | JT940231.1 | senescence-inducible chloroplast stay-green protein | 8.76E-99 | 89.65% | 488 |
| L0650 | JT951650.1 | eukaryotic elongation factor 5a-1 isoform partial | 5.16E-114 | 98.45% | 513 |
| L0651 | JT962370.1 | Cytochrome-c oxidases,electron carriers | 2.29E-32 | 95.05% | 545 |
| L0652 | JT952038.1 | 60S ribosomal protein L21 isoform 1 | 1.85E-75 | 94.45% | 535 |
| L0653 | JT955474.1 | 40S ribosomal protein S14-3 | 4.29E-68 | 97.20% | 538 |
| L0654 | JT929034.1 | alpha-1,3-mannosyltransferase | 1.91E-61 | 67.20% | 586 |
| L0655 | JR365915.1 | hydroxyproline-rich glycoprotein family protein | 1.10E-18 | 70.20% | 305 |
| L0656 | JT941283.1 | glycine-rich RNA-binding protein | 8.51E-13 | 82.35% | 478 |
| L0657 | JT941656.1 | lipid phosphate phosphatase chloroplastic | 5.50E-24 | 80.05% | 447 |
| L0658 | JT929289.1 | protein mak16 homolog a-like isoform x1 | 2.81E-80 | 94.60% | 514 |
| L0659 | JT952916.1 | rubber elongation factor isoform 2 | 6.15E-09 | 64.15% | 370 |
| L0660 | JT944959.1 | glutathione S-transferase l3-like | 2.87E-106 | 87.00% | 574 |
| L0661 | JT960555.1 | 60S ribosomal protein L12 | 7.86E-73 | 99.50% | 506 |
| L0662 | JT943978.1 | PREDICTED: uncharacterized protein LOC104449021 | 1.32E-05 | 69.00% | 241 |
| L0663 |  | calcium-transporting ATPase plasma membrane-type | 1.09E-92 | 94.30% | 503 |
| L0664 | JR348800.1 | ankyrin repeat family protein | 5.01E-50 | 89.10% | 359 |
| L0665 | JT933098.1 | probable quinone oxidoreductase | 2.32E-71 | 91.85% | 445 |
| L0666 | JT962243.1 | PREDICTED: uncharacterized protein LOC100797786 | 2.07E-30 | 85.60% | 526 |
| L0667 | JT914648.1 | myosin-15-like isoform x1 | 1.13E-58 | 88.60% | 546 |
| L0668 | JR347587.1 | 28 kDa heat- and acid-stable phosphoprotein | 2.33E-12 | 97.75% | 377 |
| L0669 | JT925230.1 | auxilin-related protein 2-like | 5.29E-05 | 65.90% | 137 |
| L0670 | JR361977.1 |  |  |  | 354 |
| L0671 | JR352803.1 | elongation factor 1-alpha (MeEF1)* | 7.00E-14 | 81.00% | 257 |
| L0672 | JT962631.1 | ATP-dependent clp protease proteolytic subunit chloroplastic-like | 7.68E-60 | 98.40% | 470 |
| L0673 | JT937150.1 | f-box family protein | 6.71E-13 | 76.00% | 131 |
| L0674 | JT957252.1 | protein tumorous imaginal mitochondrial-like | 3.26E-67 | 87.25% | 278 |
| L0675 | JT936377.1 | 60S ribosomal protein L3 | 3.78E-21 | 97.50% | 366 |
| L0676 | JT919884.1 | mitogen-activated protein kinase 10-like | 9.16E-12 | 76.35% | 466 |
| L0677 | JR366658.1 | RING finger containing protein | 3.93E-11 | 60.18% | 403 |
| L0678 | JT917112.1 | RNA-binding family protein | 2.92E-63 | 69.95% | 448 |
| L0679 | JT957041.1 |  |  |  | 472 |
| L0680 | JT955900.1 | 3r-hydroxymyristoyl- dehydratase-hydroxymyristoyl acp dehydrase isoform partial | 9.35E-44 | 72.10% | 477 |
| L0681 | JT919957.1 | pentatricopeptide repeat-containing protein mitochondrial-like | 2.43E-11 | 80.30% | 342 |
| L0682 | JT954325.1 | 40S ribosomal protein S7 | 1.76E-111 | 96.05% | 508 |
| L0683 | JT940876.1 | histidine biosynthesis bifunctional protein chloroplastic | 7.78E-24 | 79.05% | 378 |
| L0684 | JR364713.1 | interferon-related developmental regulator 1 | 8.68E-18 | 91.10% | 400 |
| L0685 | JT969857.1 | low temperature and salt responsive protein family isoform 1 | 1.21E-05 | 96.23% | 411 |
| L0686 | JR347987.1 | selenoprotein h-like | 5.76E-17 | 92.90% | 414 |
| L0687 | JT919251.1 | Serine threonine protein phosphatase 2a 59 kDa regulatory subunit b gamma isoform-like | 2.56E-14 | 88.65% | 372 |
| L0688 | JR362615.1 | protein light-dependent short hypocotyls 10-like | 1.51E-24 | 76.60% | 334 |
| L0689 | JR365382.1 | hypothetical protein JCGZ_06291 | 9.28E-26 | 74.40% | 516 |
| L0690 | JT967712.1 |  |  |  | 474 |
| L0691 | JT970460.1 |  |  |  | 570 |
| L0692 | JT962857.1 | f-box protein | 2.77E-24 | 77.85% | 592 |
| L0693 | JT954853.1 | translationally-controlled tumor protein homolog | 6.10E-61 | 92.20% | 502 |
| L0694 | JT979856.1 |  |  |  | 355 |
| L0695 | JT961833.1 | cysteine-rich and transmembrane domain-containing protein A-like (LOC105113396)* | 5.00E-70 | 85.00% | 492 |
| L0696 | JT941361.1 | maternal effect embryo arrest 14 isoform 1 | 1.77E-51 | 81.75% | 373 |
| L0697 | JT943724.1 | transcription factor TCP7-like (LOC105123927)* | 5.00E-30 | 80.00% | 319 |
| L0698 | JT926734.1 | Aspartic proteinase precursor | 3.20E-48 | 96.70% | 464 |
| L0699 | JT917055.1 | glutamate receptor precursor family protein | 2.00E-74 | 90.25% | 433 |
| L0700 | JT950122.1 | embryo defective 2735 | 1.50E-87 | 90.45% | 402 |
| L0701 | JT977363.1 |  |  |  | 468 |
| L0702 | JR360894.1 | 40S ribosomal protein S6 | 2.73E-12 | 97.45% | 352 |
| L0703 |  | profilin 1 isoform 1 | 4.57E-76 | 96.25% | 463 |
| L0704 | JT922259.1 | probable purine permease 5 isoform x2 | 1.10E-22 | 96.00% | 396 |
| L0705 | JR364573.1 | Ras-related protein Rabd1 | 4.82E-105 | 96.65% | 449 |
| L0706 | JT924276.1 | Serine threonine-protein phosphatase 7-like | 7.62E-56 | 81.90% | 618 |
| L0707 | JT935161.1 | protein tify 8-like | 1.23E-44 | 70.80% | 525 |
| L0708 | JT940751.1 | chlorophyllase 2 | 8.91E-61 | 72.85% | 438 |
| L0709 | JT921650.1 | leucine-rich repeat receptor-like protein clavata2 | 1.34E-75 | 94.70% | 499 |
| L0710 | JR345921.1 | leishmanolysin-like peptidase | 2.11E-22 | 80.30% | 523 |
| L0711 | JR365175.1 | Serine threonine-protein kinase ht1-like | 1.01E-18 | 91.70% | 528 |
| L0712 | JT958759.1 | mitochondrial import inner membrane translocase subunit Tim13-like | 4.77E-29 | 92.15% | 444 |
| L0713 | JT917381.1 | probable RNA helicase sde3 | 1.46E-11 | 77.25% | 357 |
| L0714 | JR361572.1 | NADH dehydrogenase | 1.19E-17 | 97.00% | 366 |
| L0715 | JT915078.1 | protein oberon 4 | 3.25E-10 | 78.05% | 286 |
| L0716 | JT968479.1 | 26S proteasome non-aATPase regulatory subunit 10 | 1.22E-05 | 61.80% | 307 |
| L0717 | JT943024.1 | probable protein phosphatase 2c 39 | 1.48E-64 | 85.95% | 467 |
| L0718 | JT926329.1 | NEDD8-activating enzyme e1 regulatory subunit | 3.12E-24 | 92.90% | 389 |
| L0719 | JT925333.1 | heat shock protein 70 -interacting | 2.50E-72 | 85.75% | 425 |
| L0720 | JT938333.1 | pith domain-containing protein 1 | 5.76E-74 | 93.30% | 502 |
| L0721 | JT915807.1 | ATP binding protein | 7.85E-12 | 72.70% | 388 |
| L0722 | JT923442.1 | chaperone protein | 2.57E-14 | 76.70% | 346 |
| L0723 | JT930735.1 | dolichyl-diphosphooligosaccharide--protein glycosyltransferase 48 kDa subunit | 2.96E-69 | 95.10% | 524 |
| L0724 | JT950515.1 | Serine threonine-protein kinase mhk | 5.26E-96 | 83.65% | 503 |
| L0725 | JT963564.1 | telomere repeat-binding factor 1 (LOC105129796), transcript variant X4* | 7.00E-44 | 72.00% | 576 |
| L0726 | JT948824.1 | transcription factor bHLH130-like | 5.47E-58 | 60.25% | 181 |
| L0727 |  | conserved hypothetical protein* | 2.00E-22 | 90.00% | 398 |
| L0728 | JR348030.1 | membrane-anchored ubiquitin-fold protein 3 | 8.42E-58 | 87.90% | 406 |
| L0729 | JT914766.1 | RNA polymerase II c-terminal domain phosphatase-like 3 | 1.59E-112 | 95.80% | 501 |
| L0730 | JR347160.1 | plastidic glucose transporter 4 | 4.16E-43 | 93.75% | 353 |
| L0731 | JT936010.1 | amino acid dehydrogenase family protein | 2.05E-31 | 62.90% | 299 |
| L0732 | JT942587.1 | Protein ABIL1 | 3.46E-12 | 81.80% | 498 |
| L0733 | JT944323.1 | 60S ribosomal protein L19 | 1.94E-50 | 92.05% | 563 |
| L0734 | JT928376.1 | apoptosis inhibitor 5-like | 2.00E-11 | 90.95% | 122 |
| L0735 | JT923498.1 | polyadenylate-binding protein 2 | 3.17E-65 | 94.85% | 538 |
| L0736 | JT962291.1 | ATP synthase e chain | 1.78E-18 | 85.30% | 261 |
| L0737 | JT932650.1 | HSP70-HSP90 organizing protein 3-like | 4.56E-55 | 76.20% | 419 |
| L0738 | JT956481.1 |  |  |  | 231 |
| L0739 | JT959160.1 | probable protein phosphatase 2c 55 | 1.40E-29 | 93.65% | 411 |
| L0740 | JR344506.1 | interferon-induced GTP-binding protein | 5.52E-32 | 80.35% | 471 |
| L0741 | JT932156.1 | o-glucosyltransferase rumi homolog isoform x1 | 3.73E-97 | 60.95% | 387 |
| L0742 | JT964250.1 | pollen-specific protein sf3-like | 7.26E-48 | 96.90% | 403 |
| L0743 | JT935795.1 | mitogen activated protein kinase kinase, mapkk2 | 3.80E-49 | 89.15% | 293 |
| L0744 | JR348209.1 | hypothetical protein JCGZ_14340 | 7.52E-34 | 78.95% | 406 |
| L0745 | JT953335.1 | germin-like protein 2-1 | 6.06E-65 | 88.90% | 462 |
| L0746 | JT939226.1 | methylenetetrahydrofolate reductase | 8.60E-16 | 67.15% | 309 |
| L0747 | JR364455.1 | 60S ribosomal protein L35 | 4.34E-65 | 98.00% | 453 |
| L0748 | JT939821.1 | nudix hydrolase mitochondrial-like isoform x1 | 8.75E-151 | 87.30% | 243 |
| L0749 | JT919385.1 | heat shock protein isoform 1 | 4.46E-51 | 53.65% | 473 |
| L0750 | JT914256.1 | calpain-type cysteine protease dek1 | 6.03E-86 | 96.50% | 421 |
| L0751 |  | gdsl esterase lipase at2g23540-like | 9.28E-44 | 91.50% | 432 |
| L0752 | JT975801.1 |  |  |  | 301 |
| L0753 | JT959743.1 | protein light-dependent short hypocotyls 1-like | 2.23E-28 | 96.90% | 503 |
| L0754 | JT948083.1 | huntingtin-interacting protein K-like | 6.70E-45 | 96.20% | 461 |
| L0755 | JT971657.1 | oligosaccharyltransferase complex subunit ostc-like | 3.89E-78 | 92.30% | 503 |
| L0756 | JT944797.1 | carbonic anhydrase family protein | 4.20E-76 | 80.75% | 623 |
| L0757 | JR364327.1 | Ubiquitin-40S ribosomal protein s27a-like | 8.38E-34 | 99.30% | 500 |
| L0758 | JT947610.1 | ADP-ribosylation factor 1 | 2.63E-125 | 99.30% | 663 |
| L0759 | JT976286.1 | conserved hypothetical protein | 6.16E-54 | 89.20% | 496 |
| L0760 | JT930064.1 | cytochrome c oxidase copper chaperone | 1.06E-46 | 85.50% | 590 |
| L0761 | JT955746.1 | protein mago nashi homolog | 3.17E-34 | 95.00% | 246 |
| L0762 | JT937759.1 | tetratricopeptide repeat-like superfamily protein isoform 1 | 2.64E-35 | 90.70% | 532 |
| L0763 | JT945269.1 | 50S ribosomal protein chloroplastic | 3.91E-84 | 95.05% | 588 |
| L0764 | JR364722.1 | Speckle-type POZ protein | 2.92E-17 | 83.95% | 552 |
| L0765 | JT937874.1 | YEATS domain-containing protein | 3.95E-171 | 87.85% | 589 |
| L0766 | JT960011.1 | accelerated cell death partial | 1.79E-104 | 92.05% | 627 |
| L0767 | JT942179.1 | DNA polymerase zeta catalytic subunit | 1.07E-50 | 63.45% | 585 |
| L0768 | JT934922.1 | low quality protein: 5 -adenylylsulfate reductase-like 5 | 7.93E-08 | 70.75% | 524 |
| L0769 | JT927882.1 | IAA-amino acid hydrolase ilr1-like 3 | 2.47E-127 | 86.00% | 662 |
| L0770 | JR346924.1 | dolichol phosphate-mannose biosynthesis regulatory protein | 3.60E-36 | 87.90% | 508 |
| L0771 | JR350045.1 | RNA and export factor binding | 3.30E-45 | 72.60% | 660 |
| L0772 |  | callose synthase 12-like | 1.75E-99 | 88.15% | 497 |
| L0773 | JR345824.1 | protein CURVATURE THYLAKOID 1D, chloroplastic | 3.15E-05 | 54.00% | 505 |
| L0774 | JT920463.1 | MYB sant-like DNA-binding domain protein | 6.61E-64 | 66.67% | 613 |
| L0775 | JT915662.1 | Serine threonine-protein kinase | 3.19E-104 | 88.40% | 574 |
| L0776 |  | lipid binding protein* | 2.00E-50 | 70.00% | 627 |
| L0777 | JR365150.1 | thioesterase superfamily protein | 2.42E-35 | 89.75% | 479 |
| L0778 | JT950957.1 | golgi apparatus membrane protein echiDNA | 1.73E-109 | 94.85% | 583 |
| L0779 | JT952222.1 | DNA-binding family protein | 6.17E-46 | 80.95% | 368 |
| L0780 | JT937457.1 | protein brassinazole-resistant 1-like | 7.39E-29 | 81.75% | 576 |
| L0781 | JT948548.1 | 40S ribosomal protein S3 | 4.59E-68 | 94.75% | 559 |
| L0782 | JT957217.1 | 60S ribosomal protein L30 | 3.00E-71 | 95.80% | 568 |
| L0783 | JT958386.1 | PHD finger protein alfin-like 1-like | 1.96E-43 | 88.60% | 599 |
| L0784 | JT931101.1 | zinc finger CCCH domain-containing protein 11-like | 2.02E-38 | 95.35% | 284 |
| L0785 | JT944718.1 | membrane steroid-binding protein 2-like | 2.37E-68 | 84.15% | 650 |
| L0786 | JT943366.1 | charged multivesicular body protein 5-like | 1.57E-73 | 93.15% | 616 |
| L0787 | JT928922.1 | kinesin-like protein nack1 | 3.89E-60 | 81.65% | 619 |
| L0788 | JT919608.1 | swap surp domain-containing protein d111 g-patch domain-containing protein isoform 1 | 4.68E-68 | 74.10% | 543 |
| L0789 |  | eukaryotic translation initiation factor 2 subunit gamma-like | 3.15E-14 | 70.15% | 572 |
| L0790 | JT933529.1 | eukaryotic translation initiation factor 3 subunit k-like | 1.42E-68 | 88.55% | 585 |
| L0791 | JR348651.1 | cap-binding protein | 1.33E-109 | 88.55% | 626 |
| L0792 | JT924112.1 | probable alpha-amylase 2 | 3.72E-58 | 91.65% | 527 |
| L0793 | JT951926.1 | HSP40 cysteine-rich domain superfamily protein | 8.63E-51 | 74.55% | 383 |
| L0794 | JT958815.1 | eukaryotic translation initiation factor 1a-like | 1.68E-26 | 80.50% | 610 |
| L0795 | JT923638.1 | oxysterol-binding protein 5 | 4.99E-15 | 67.90% | 386 |
| L0796 | JT930092.1 | f-box protein fbw2 | 1.14E-08 | 81.00% | 555 |
| L0797 | JT973670.1 | cofactor assembly of complex c | 2.96E-12 | 62.75% | 425 |
| L0798 | JT916061.1 | calmodulin-binding transcription activator 2 | 9.67E-74 | 83.60% | 444 |
| L0799 | JT944013.1 | gibberellin receptor | 1.56E-98 | 83.15% | 525 |
| L0800 | JT948377.1 | 40S ribosomal protein S9 | 6.55E-57 | 98.70% | 515 |
| L0801 | JT942867.1 | DUF593-containing protein | 9.85E-40 | 81.55% | 388 |
| L0802 | JR362412.1 | Ras-related protein Rab7-like | 1.00E-62 | 95.75% | 562 |
| L0803 | JT950658.1 | signal peptidase complex catalytic subunit sec11a | 5.13E-100 | 99.10% | 579 |
| L0804 | JT923258.1 | pyruvate dehydrogenase (acetyl-transferring) mitochondrial-like | 1.74E-34 | 92.90% | 475 |
| L0805 | JT923477.1 | glucose-6-phosphate 1-epimerase | 1.35E-66 | 91.75% | 628 |
| L0806 | JT935771.1 | caffeic acid o-methyltransferase | 8.96E-84 | 93.85% | 617 |
| L0807 | JT924572.1 | inositol transporter 4 like protein | 1.75E-43 | 83.00% | 542 |
| L0808 | JR366016.1 | prp38 family protein isoform 1 | 1.38E-110 | 94.20% | 586 |
| L0809 | JT954516.1 | hypothetical protein JCGZ_07980 | 4.99E-48 | 67.55% | 460 |
| L0810 | JR346921.1 | DNA-binding protein MNB2B | 1.46E-31 | 86.40% | 438 |
| L0811 | JT940724.1 | E3 ubiquitin-protein ligase rma1h1-like | 2.06E-60 | 79.65% | 559 |
| L0812 | JR345782.1 | casein kinase family protein | 1.84E-30 | 82.60% | 518 |
| L0813 | JT933351.1 | p-loop containing nucleoside triphosphate hydrolases superfamily protein isoform 1 | 1.87E-55 | 93.50% | 383 |
| L0814 | JT953136.1 | latex allergen Hev b 5* | 0.00E+00 | 100.00% | 573 |
| L0815 | JT943548.1 | DNAj chaperone c-terminal domain-containing family protein | 3.15E-58 | 94.40% | 576 |
| L0816 | JT926099.1 | plasma-membrane choline transporter family protein | 5.53E-85 | 89.40% | 496 |
| L0817 | JT928840.1 | 60S ribosomal protein L18a-1 | 1.32E-86 | 97.75% | 653 |
| L0818 | JR349859.1 | homeobox protein knotted-1-like 2 | 2.72E-61 | 97.80% | 578 |
| L0819 | JT952836.1 | alpha-glucan water dikinase, chloroplast precursor | 1.04E-41 | 95.00% | 394 |
| L0820 | JT932354.1 | early nodulin-like protein 3 | 3.87E-64 | 73.35% | 497 |
| L0821 | JT951662.1 | S-adenosylmethionine-dependent methyltransferase | 1.61E-31 | 74.05% | 512 |
| L0822 | JT925526.1 | E3 ubiquitin-protein ligase | 2.42E-68 | 79.55% | 616 |
| L0823 | JT949604.1 | small rubber particle protein | 2.32E-125 | 70.40% | 655 |
| L0824 | JT966221.1 | MADS box | 2.90E-71 | 75.60% | 551 |
| L0825 | JT932753.1 | tRNA pseudouridine synthase mitochondrial-like | 8.79E-10 | 72.91% | 407 |
| L0826 | JT914874.1 | eukaryotic translation initiation factor 5b-like | 1.43E-33 | 94.45% | 436 |
| L0827 | JR358736.1 | kdel motif-containing protein 1 | 3.01E-05 | 68.00% | 455 |
| L0828 | JT959842.1 | tmv resistance protein n-like | 1.33E-47 | 74.95% | 588 |
| L0829 | JT948874.1 | upf0690 protein c1orf52 homolog | 1.57E-50 | 84.45% | 185 |
| L0830 | JT973110.1 | Vacuolar protein sorting-associated protein 55 homolog | 6.68E-22 | 58.90% | 578 |
| L0831 | JT934598.1 | HSP70-binding protein 1-like | 1.58E-116 | 94.40% | 564 |
| L0832 | JT953232.1 | dead box ATP-dependent RNA helicase | 2.43E-26 | 55.71% | 673 |
| L0833 | JT940842.1 | transcription factor GTE4-like | 1.01E-10 | 85.95% | 670 |
| L0834 | JT922336.1 | sensory transduction histidine kinase | 6.86E-74 | 68.85% | 529 |
| L0835 | JT924724.1 | villin 1-4 | 5.50E-15 | 80.90% | 507 |
| L0836 | JT931364.1 | Serine threonine-protein phosphatase pp2a-3 catalytic subunit | 2.11E-96 | 96.95% | 564 |
| L0837 | JT955575.1 | bi1-like protein | 1.01E-41 | 91.65% | 296 |
| L0838 | JT916724.1 | autophagy-related protein 18f-like | 2.36E-100 | 85.80% | 541 |
| L0839 | JT945616.1 | probable E3 ubiquitin-protein ligase RNF144A-B | 1.32E-72 | 82.90% | 512 |
| L0840 | JT947845.1 | transcription initiation factor IIf subunit beta | 1.09E-97 | 88.45% | 579 |
| L0841 | JT968723.1 | S-adenosyl-L-methionine-dependent methyltransferases superfamily protein isoform 1 | 2.52E-65 | 95.65% | 558 |
| L0842 | JT955868.1 | peptidyl-prolyl cis-trans isomerase e | 4.98E-40 | 96.65% | 611 |
| L0843 | JR349346.1 | DNA-directed RNA polymerase III subunit rpc10-like | 1.54E-46 | 76.50% | 591 |
| L0844 | JT935326.1 | homogentisate phytyltransferase chloroplastic | 8.41E-17 | 85.10% | 583 |
| L0845 | JT924160.1 | heat shock protein 70 | 4.44E-05 | 85.00% | 423 |
| L0846 | JT932745.1 | Ras-related protein Rab11c | 8.98E-91 | 94.75% | 637 |
| L0847 | JT953821.1 | proteophosphoglycan-related family protein | 9.92E-12 | 71.88% | 539 |
| L0848 | JT928420.1 | protein binding protein | 1.24E-57 | 77.30% | 603 |
| L0849 | JT973140.1 | GDP-mannose transporter | 4.59E-66 | 91.30% | 581 |
| L0850 | JT916689.1 | structural maintenance of chromosomes protein 3-like | 1.87E-99 | 98.55% | 647 |
| L0851 | JT950973.1 | U6 snRNA-associated sm-like protein lsm7 | 2.28E-60 | 97.35% | 645 |
| L0852 | JT921870.1 | cytochrome c oxidase subunit 3 | 5.65E-18 | 61.20% | 570 |
| L0853 | JT965917.1 | cyclin-dependent protein kinase inhibitor smr2-like | 1.08E-29 | 62.30% | 543 |
| L0854 | JT938138.1 | conserved hypothetical protein | 1.63E-39 | 88.60% | 460 |
| L0855 | JT957380.1 | NADH dehydrogenase | 6.16E-48 | 91.90% | 484 |
| L0856 | JT969804.1 | Blue copper protein precursor | 1.55E-55 | 75.25% | 578 |
| L0857 | JR366088.1 | enoyl- hydratase isomerase family protein | 5.13E-71 | 83.85% | 547 |
| L0858 | JT924149.1 | axoneme-associated protein mst101 -like isoform x2 | 4.69E-49 | 67.35% | 475 |
| L0859 | JT954271.1 | Ubiquitin-conjugating enzyme e2 7 | 3.51E-71 | 98.25% | 454 |
| L0860 | JT961665.1 | t-box transcription factor isoform 1 | 6.42E-130 | 76.20% | 711 |
| L0861 | JT940255.1 | dcn1-like protein 4 | 5.50E-35 | 91.05% | 514 |
| L0862 | JT922864.1 | protein modifier of snc1 11 isoform x1 | 2.75E-16 | 82.75% | 531 |
| L0863 | JR365018.1 | yggt family protein | 2.17E-15 | 72.67% | 489 |
| L0864 | JT967526.1 | Ubiquitin-conjugating enzyme e2 7 | 2.66E-78 | 96.25% | 557 |
| L0865 | JT956763.1 | mitotic-spindle organizing protein 1b-like isoform x2 | 1.00E-25 | 87.10% | 591 |
| L0866 | JT958818.1 | heterogeneous nuclear ribonucleoprotein 1 | 9.07E-93 | 95.40% | 527 |
| L0867 | JT947647.1 | ABC transporter G family member 11 | 2.24E-80 | 91.65% | 495 |
| L0868 | JT927552.1 | probable solanesyl-diphosphate synthase chloroplastic | 5.21E-31 | 86.95% | 479 |
| L0869 | JT925615.1 | protein kinase chloroplastic | 1.36E-42 | 85.85% | 466 |
| L0870 | JT922847.1 | phosphoglucomutase family protein | 1.90E-64 | 94.45% | 562 |
| L0871 |  | pentatricopeptide repeat-containing protein at4g21170 | 3.79E-26 | 58.25% | 628 |
| L0872 | JT941825.1 | GTP-binding family protein | 1.28E-46 | 95.15% | 550 |
| L0873 | JR364054.1 | small ubiquitin-related modifier 1 | 9.26E-63 | 95.05% | 533 |
| L0874 | JR363442.1 | hypothetical protein JCGZ_11834 | 1.70E-28 | 92.60% | 569 |
| L0875 | JT961840.1 | tRNA pseudouridine(38 39) synthase | 1.16E-79 | 78.70% | 520 |
| L0876 | JT947046.1 | cell number regulator 6-like | 7.36E-98 | 89.05% | 553 |
| L0877 | JR361262.1 | protein exordium-like 2 | 2.71E-29 | 95.00% | 439 |
| L0878 | JT933554.1 |  |  |  | 628 |
| L0879 | JT944339.1 | hypothetical protein JCGZ_09365 | 1.51E-05 | 56.00% | 469 |
| L0880 | JT929782.1 | translationally controlled tumor protein | 8.91E-105 | 96.00% | 686 |
| L0881 | JT914463.1 | proteasome activator subunit 4 | 2.29E-86 | 89.35% | 500 |
| L0882 | JT914229.1 | DNAj heat shock n-terminal domain-containing family protein | 5.39E-32 | 79.65% | 624 |
| L0883 | JT966581.1 | hypothetical protein JCGZ_01429 | 9.98E-05 | 65.00% | 473 |
| L0884 | JT937268.1 | calcineurin b-like protein 10 | 1.11E-138 | 86.55% | 249 |
| L0885 | JT925665.1 | ABC transporter B family member 15-like | 7.14E-124 | 95.50% | 668 |
| L0886 | JT924099.1 | smr (small related) domain-containing protein | 7.80E-13 | 76.20% | 540 |
| L0887 | JT945312.1 | translation initiation factor IF-2 | 1.55E-40 | 73.60% | 647 |
| L0888 | JT919818.1 | hypothetical protein JCGZ_26391 | 2.12E-118 | 69.55% | 691 |
| L0889 | JR366419.1 | esterase lipase thioesterase family protein | 2.29E-123 | 86.70% | 673 |
| L0890 | JT917931.1 | peptidyl-prolyl cis-trans isomerase | 1.54E-89 | 78.70% | 641 |
| L0891 | JT966473.1 | dynein light chain cytoplasmic-like | 2.67E-14 | 91.30% | 508 |
| L0892 | JT948343.1 | rubber elongation factor | 3.06E-27 | 78.14% | 527 |
| L0893 | JT926093.1 | hydroxyproline-rich glycoprotein | 7.25E-51 | 84.70% | 652 |
| L0894 | JT954083.1 | E3 ubiquitin protein ligase drip2-like | 5.71E-29 | 92.75% | 459 |
| L0895 | JT918153.1 | pentatricopeptide repeat-containing protein at5g18950 | 6.34E-13 | 69.05% | 491 |
| L0896 | JT935611.1 | quinone-oxidoreductase chloroplastic | 1.06E-67 | 91.60% | 513 |
| L0897 | JT928881.1 | Ubiquitin-specific protease 6 family protein | 9.49E-100 | 93.30% | 555 |
| L0898 | JT955224.1 | Ubiquitin-conjugating enzyme e2 variant 1a | 1.71E-26 | 95.55% | 408 |
| L0899 | JT946736.1 | vesicle transport protein got1b-like | 3.21E-54 | 95.20% | 641 |
| L0900 | JT962764.1 | hypothetical protein JCGZ_27126 | 4.64E-18 | 75.90% | 688 |
| L0901 | JT963638.1 | uncharacterized protein TCM_030749 | 1.43E-27 | 86.70% | 546 |
| L0902 | JT960922.1 | 2-dehydro-3-deoxyphosphooctonate isoform 1 | 1.67E-26 | 71.75% | 687 |
| L0903 | JT950551.1 | nicotiana HR lesion-inducing family protein | 1.24E-54 | 80.75% | 710 |
| L0904 | JT930757.1 | zinc finger CCCH domain-containing protein 22 | 2.32E-62 | 77.20% | 659 |
| L0905 | JR345830.1 | pyrroline-5-carboxylate reductase | 1.20E-66 | 85.15% | 647 |
| L0906 | JT961680.1 | ozone-responsive stress related protein | 9.70E-43 | 90.05% | 504 |
| L0907 | JT936374.1 | 1-deoxy-D-xylulose 5-phosphate synthase (DXS2) | 9.31E-32 | 93.25% | 502 |
| L0908 | JR346087.1 | Cytochrome-c oxidases,electron carriers | 8.35E-37 | 93.85% | 508 |
| L0909 | JT948760.1 | 60S ribosomal protein L15 | 2.71E-47 | 97.00% | 548 |
| L0910 | JR357914.1 | hypothetical protein JCGZ_15748 | 2.56E-08 | 72.19% | 334 |
| L0911 | JT942109.1 | Alba DNA RNA-binding protein isoform partial | 2.25E-82 | 87.45% | 592 |
| L0912 | JT931800.1 | conserved hypothetical protein | 3.46E-20 | 75.05% | 605 |
| L0913 | JT947414.1 | hydroxysteroid dehydrogenase | 1.93E-77 | 80.00% | 522 |
| L0914 | JT927033.1 | dead-box ATP-dependent RNA helicase 5 | 7.60E-23 | 93.30% | 467 |
| L0915 | JT937900.1 | chaperone protein | 3.75E-28 | 92.75% | 438 |
| L0916 | JT942748.1 | clavaminate synthase-like protein at3g21360 | 4.18E-109 | 92.90% | 603 |
| L0917 | JR347951.1 | bifunctional 3-dehydroquinate dehydratase shikimate chloroplastic-like | 7.49E-68 | 94.30% | 570 |
| L0918 | JT948318.1 | phosphate transporter isoform 2 | 1.21E-35 | 61.55% | 619 |
| L0919 | JT941629.1 | cytochrome P450 (CYP98A27) | 4.37E-53 | 94.00% | 451 |
| L0920 | JT953513.1 | 40S ribosomal protein S13-like | 4.42E-55 | 100.00% | 644 |
| L0921 | JT954377.1 | histone deacetylase complex subunit sap18 | 1.59E-58 | 92.85% | 594 |
| L0922 | JT947856.1 | rRNA-processing protein fyv7 | 2.95E-21 | 85.11% | 592 |
| L0923 | JT962562.1 | vesicle transport protein sft2b | 3.42E-79 | 83.55% | 672 |
| L0924 | JT921425.1 | transcriptional adapter 1 | 5.00E-89 | 81.50% | 611 |
| L0925 | JT956786.1 | cytochrome c | 5.46E-73 | 95.65% | 427 |
| L0926 | JT952021.1 | membrane-anchored ubiquitin-fold protein 4-like | 3.52E-64 | 93.20% | 209 |
| L0927 | JR349082.1 | probable histone H2A | 8.84E-39 | 93.25% | 450 |
| L0928 | JT936406.1 | thump domain-containing protein 1 isoform 2 | 3.68E-08 | 88.00% | 445 |
| L0929 | JT931717.1 | protein shoot gravitropism 5 | 4.19E-38 | 88.25% | 499 |
| L0930 | JT947085.1 | protein translocase subunit | 5.32E-56 | 84.30% | 478 |
| L0931 | JT927609.1 | transcription initiation factor tfIId subunit 6 | 1.49E-81 | 72.45% | 668 |
| L0932 | JT918152.1 | PREDICTED: uncharacterized protein LOC101496870 | 5.41E-42 | 54.20% | 692 |
| L0933 | JT916384.1 | hypothetical protein PRUPE_ppa017051mg | 5.35E-66 | 67.33% | 511 |
| L0934 | JR365195.1 | small Ubiquitin-related modifier 1-like | 2.11E-47 | 98.10% | 525 |
| L0935 | JR365417.1 | zinc finger and btb domain-containing protein 11 isoform partial | 2.81E-45 | 80.70% | 425 |
| L0936 | JT950228.1 | DUF674 family protein | 6.97E-35 | 51.90% | 647 |
| L0937 | JT949450.1 | elongation factor 1-delta-like | 7.96E-36 | 96.00% | 552 |
| L0938 | JT974017.1 | 60S ribosomal protein L44 | 1.75E-20 | 66.65% | 613 |
| L0939 | JR366259.1 | ethylene-responsive transcription factor 3 | 3.46E-26 | 82.05% | 667 |
| L0940 | JT947514.1 | splicing factor 3b subunit 4 | 8.43E-35 | 66.60% | 578 |
| L0941 | JT934451.1 | hypothetical protein JCGZ_10167 | 3.47E-114 | 58.15% | 533 |
| L0942 | JT955986.1 | 50S ribosomal protein CL25, chloroplast precursor | 1.75E-26 | 90.55% | 553 |
| L0943 | JT943173.1 | DNA binding protein | 1.02E-73 | 84.15% | 620 |
| L0944 | JT919168.1 | methionine-tRNA synthetase | 2.20E-101 | 93.40% | 583 |
| L0945 | JT932094.1 | dr1-associated co-repressor | 2.33E-12 | 85.00% | 529 |
| L0946 | JT927577.1 | ATP synthase subunit alpha | 8.99E-103 | 76.05% | 482 |
| L0947 | JT961020.1 | hypothetical protein JCGZ_10107 | 4.96E-37 | 94.40% | 540 |
| L0948 | JR357889.1 | PREDICTED: uncharacterized protein LOC104601116 | 6.24E-24 | 65.50% | 648 |
| L0949 | JT955678.1 | Ubiquitin-nedd8-like protein rub2 | 7.52E-79 | 99.00% | 626 |
| L0950 | JT964098.1 | Ubiquitin carboxyl-terminal hydrolase 23 | 4.01E-10 | 62.50% | 669 |
| L0951 | JT945033.1 | snf1-related protein kinase regulatory subunit beta-3 | 2.14E-42 | 87.70% | 653 |
| L0952 | JT963739.1 | 40S ribosomal protein S28-like | 6.07E-25 | 99.40% | 594 |
| L0953 | JT947320.1 | was wasl-interacting protein family member 3-like | 6.65E-62 | 80.20% | 650 |
| L0954 |  | sterol carrier | 7.85E-27 | 76.95% | 490 |
| L0955 | JT955777.1 | Histone H2A | 3.06E-41 | 98.40% | 533 |
| L0956 | JT948425.1 | 60S ribosomal protein L18 | 1.93E-70 | 97.65% | 547 |
| L0957 | JT956151.1 | elicitor-responsive protein 3 | 5.42E-23 | 83.40% | 389 |
| L0958 | JT953584.1 | uncharacterized loc101209217 | 1.13E-18 | 67.59% | 623 |
| L0959 | JT928530.1 | probable protein phosphatase 2c 5 | 8.42E-65 | 92.25% | 542 |
| L0960 | JT945149.1 | two-component response regulator arr5-like | 1.22E-98 | 86.05% | 657 |
| L0961 | JT932200.1 | protein IQ-DOMAIN 31-like | 2.26E-57 | 73.75% | 580 |
| L0962 | JT950520.1 | rubredoxin family protein | 7.92E-67 | 87.45% | 635 |
| L0963 | JT968270.1 | hypothetical protein JCGZ_15745 | 4.18E-07 | 77.60% | 604 |
| L0964 | JR364123.1 | protein DCL, chloroplastic-like | 5.41E-72 | 73.50% | 479 |
| L0965 | JT961102.1 | zinc finger A20 and AN1 domain-containing stress-associated protein 4 | 2.44E-40 | 75.05% | 557 |
| L0966 | JR365807.1 | r2r3-MYB transcription factor | 5.92E-87 | 89.50% | 594 |
| L0967 | JT961635.1 | potassium transporter 12 | 9.74E-29 | 70.60% | 654 |
| L0968 | JT939569.1 | f-box protein at5g52880 | 8.96E-89 | 69.30% | 677 |
| L0969 | JT945891.1 | phosphatidylinositol n-acetylglucosaminyltransferase subunit p | 6.50E-47 | 86.40% | 518 |
| L0970 | JT957627.1 | 40S ribosomal protein S20-2 | 7.81E-71 | 97.70% | 644 |
| L0971 |  | CDP-diacylglycerol--glycerol-3-phosphate 3-phosphatidyltransferase, putative | 2.22E-35 | 75.55% | 514 |
| L0972 | JR348784.1 | upf0690 protein c1orf52 homolog | 3.33E-44 | 83.85% | 453 |
| L0973 | JT967948.1 | chaperone protein DNAj chloroplastic-like | 9.02E-78 | 68.20% | 630 |
| L0974 | JT954797.1 | nuclear transcription factor y subunit c-10-like | 1.32E-77 | 70.85% | 582 |
| L0975 | JR348971.1 | hypothetical protein JCGZ_08737 | 4.52E-95 | 95.40% | 569 |
| L0976 | JT948662.1 | sap domain-containing protein | 7.31E-35 | 80.20% | 583 |
| L0977 | JT969006.1 | Mitochondrial deoxynucleotide carrier | 2.26E-27 | 90.45% | 467 |
| L0978 | JT958576.1 | 40S ribosomal protein S27-2 | 5.24E-46 | 99.15% | 598 |
| L0979 | JT957728.1 | Ubiquitin-40S ribosomal protein s27a-like | 4.57E-65 | 99.55% | 571 |
| L0980 |  | LZ-NBS-LRR resistance protein | 8.82E-45 | 66.85% | 573 |
| L0981 | JT973373.1 |  |  |  | 321 |
| L0982 | JT915699.1 | trafficking protein particle complex subunit 11 | 3.69E-13 | 82.80% | 357 |
| L0983 | JT925401.1 | gibberellin receptor gid1b-like | 4.59E-31 | 94.00% | 442 |
| L0984 | JT950117.1 | tubulin beta-5 chain (LOC105159935), transcript variant X2* | 2.00E-37 | 86.00% | 451 |
| L0985 | JT963102.1 | peptidyl-prolyl cis-trans isomerase g | 3.13E-31 | 79.65% | 480 |
| L0986 | JT921398.1 | regulation of nuclear pre-mRNA domain-containing protein 1b-like | 4.91E-67 | 74.60% | 521 |
| L0987 | JT949130.1 | Serine arginine repetitive matrix protein 1-like isoform x1 | 1.76E-67 | 96.30% | 571 |
| L0988 | JT955898.1 | RmlC-like cupins superfamily protein | 1.05E-61 | 94.45% | 489 |
| L0989 | JT955990.1 | ethylene-responsive transcription factor 7-like | 5.99E-17 | 66.18% | 574 |
| L0990 | JT937737.1 | nodulin family protein | 8.39E-23 | 90.55% | 522 |
| L0991 | JT934918.1 | homeobox-leucine zipper protein athb-13 | 3.79E-63 | 84.90% | 560 |
| L0992 | JT960630.1 | rpm1-interacting protein 4-like isoform x2 | 1.25E-40 | 86.30% | 586 |
| L0993 | JT935403.1 | proteoglycan 4 | 1.04E-41 | 79.35% | 393 |
| L0994 | JT945230.1 | conserved hypothetical protein | 5.40E-58 | 64.10% | 585 |
| L0995 | JT922733.1 | nuclear transcription factor y subunit b-10 isoform 2 | 4.88E-57 | 88.90% | 613 |
| L0996 | JR347117.1 | ATP synthase subunit mitochondrial | 3.25E-56 | 97.50% | 618 |
| L0997 | JT956482.1 | p-loop containing nucleoside triphosphate hydrolases superfamily protein isoform 2 | 1.85E-30 | 91.45% | 615 |
| L0998 | JT958584.1 | macrophage migration inhibitory factor homolog | 1.34E-70 | 94.95% | 567 |
| L0999 | JR364472.1 | protein argonaute 10-like isoform x1 | 1.21E-20 | 71.70% | 487 |
| L1000 | JT953418.1 | WD repeat-containing protein 48-like (LOC105131295)* | 4.00E-72 | 73.00% | 516 |
| L1001 | JT950161.1 | 40S ribosomal protein S8 | 6.32E-69 | 92.30% | 508 |
| L1002 | JR361481.1 | glutaredoxin c4 | 7.25E-24 | 89.10% | 367 |
| L1003 | JT955830.1 | protein CURVATURE THYLAKOID 1D, chloroplastic | 4.35E-32 | 70.20% | 458 |
| L1004 | JT918808.1 | ATP-dependent zinc metalloprotease chloroplastic | 4.46E-80 | 87.80% | 483 |
| L1005 | JT949317.1 | monothiol glutaredoxin- chloroplastic | 4.19E-85 | 82.65% | 584 |
| L1006 | JT962750.1 |  |  |  | 581 |
| L1007 | JT932074.1 | signal peptide peptidase-like 2b | 2.65E-119 | 86.75% | 608 |
| L1008 | JT934829.1 | thioredoxin-related protein | 6.19E-87 | 90.50% | 556 |
| L1009 | JT963911.1 | mediator of RNA polymerase II transcription subunit 15a-like | 8.78E-31 | 79.00% | 654 |
| L1010 | JT963076.1 | cytochrome b-c1 complex subunit 9-like | 1.04E-37 | 87.05% | 610 |
| L1011 | JT920887.1 | wall-associated receptor kinase-like 14 | 1.88E-41 | 78.20% | 564 |
| L1012 | JR347444.1 | hypothetical protein POPTR_0002s26410g | 4.39E-29 | 93.60% | 478 |
| L1013 | JT955226.1 | signal recognition particle 14 kDa protein | 2.61E-09 | 91.00% | 510 |
| L1014 | JT966538.1 | hypothetical protein JCGZ_14517 | 4.57E-46 | 60.70% | 534 |
| L1015 | JT961150.1 | stress-associated endoplasmic reticulum protein 2 | 1.03E-39 | 95.85% | 477 |
| L1016 | JT946494.1 | uridylate kinase | 8.36E-34 | 74.15% | 614 |
| L1017 | JR345530.1 | small nuclear ribonucleoprotein sm d3-like | 1.07E-77 | 97.00% | 392 |
| L1018 | JT939412.1 | probable low-specificity l-threonine aldolase 1 | 4.84E-47 | 84.25% | 513 |
| L1019 | JR366143.1 | humj1 family protein | 1.60E-69 | 66.50% | 608 |
| L1020 | JT940016.1 | tetratricopeptide repeat protein 33 | 1.48E-39 | 90.75% | 540 |
| L1021 | JT969015.1 | profilin | 1.14E-19 | 91.80% | 544 |
| L1022 | JT938048.1 | methyl-CpG-binding domain-containing family protein | 3.98E-110 | 83.50% | 669 |
| L1023 | JT932401.1 | coiled-coil domain-containing protein r3hcc1l-like | 2.99E-16 | 79.60% | 658 |
| L1024 | JT951486.1 | uncharacterized loc102591911 | 1.51E-124 | 90.10% | 672 |
| L1025 | JT953412.1 | protein yls9-like | 1.12E-91 | 88.60% | 560 |
| L1026 | JT963696.1 | hypothetical protein JCGZ_17913 | 8.31E-56 | 92.90% | 548 |
| L1027 | JT924257.1 | upf0400 protein isoform x1 | 3.77E-93 | 78.90% | 652 |
| L1028 |  | ARM repeat superfamily protein isoform 2 | 2.59E-46 | 68.55% | 497 |
| L1029 | JT957910.1 | Vacuolar protein sorting-associated protein 25 | 8.02E-85 | 93.95% | 700 |
| L1030 | JT931089.1 | tga-type basic leucine zipper protein | 4.79E-10 | 85.55% | 475 |
| L1031 | JT936523.1 | transmembrane protein 205 | 1.84E-87 | 89.10% | 492 |
| L1032 | JT932326.1 | eukaryotic initiation factor 4a-10 | 2.10E-55 | 99.10% | 494 |
| L1033 | JT930737.1 | DNA-directed RNA polymerase subunit alpha | 5.10E-55 | 77.70% | 545 |
| L1034 | JT917174.1 | DNA mismatch repair protein msh2 | 1.11E-11 | 89.30% | 548 |
| L1035 | JT942830.1 | Ras-related protein Rabc1 | 1.51E-40 | 93.50% | 544 |
| L1036 | JT926298.1 | catalase 1 | 1.67E-72 | 96.90% | 511 |
| L1037 | JT917712.1 | leucine-rich repeat-containing protein ddb_g0290503 | 1.45E-20 | 76.30% | 479 |
| L1038 | JT935448.1 | expansin-a13-like | 6.16E-45 | 92.90% | 506 |
| L1039 | JT953879.1 | s-adenosyl-l-methionine-dependent methyltransferases superfamily protein | 3.04E-47 | 92.20% | 540 |
| L1040 |  | mlp-like protein 329 | 7.84E-13 | 63.30% | 501 |
| L1041 | JT966343.1 | L antigen family member 3 | 1.27E-48 | 88.70% | 547 |
| L1042 | JT957401.1 |  |  |  | 543 |
| L1043 | JR348217.1 | glycine cleavage system h protein mitochondrial | 5.87E-17 | 84.80% | 392 |
| L1044 | JT932905.1 | Rab geranylgeranyl transferase alpha subunit isoform 1 | 2.39E-83 | 78.05% | 538 |
| L1045 | JT949073.1 | keratin-associated protein 10-6 isoform 1 | 1.14E-30 | 86.80% | 474 |
| L1046 | JT963648.1 | pentatricopeptide repeat-containing protein mitochondrial | 4.54E-49 | 89.95% | 469 |
| L1047 | JT954755.1 |  |  |  | 396 |
| L1048 | JT941773.1 | uncharacterized loc101220013 | 5.02E-179 | 79.30% | 590 |
| L1049 | JT945364.1 | glutamine amidotransferase ylr126c | 3.68E-57 | 90.15% | 625 |
| L1050 | JT978406.1 | brain acid soluble protein 1 homolog | 1.36E-11 | 74.57% | 538 |
| L1051 | JT949459.1 | Heme-binding protein | 3.37E-86 | 77.35% | 531 |
| L1052 | JT959077.1 | elicitor-responsive protein 3 | 1.51E-94 | 84.40% | 639 |
| L1053 | JT933304.1 | trihelix transcription factor ptl-like | 6.01E-49 | 57.80% | 517 |
| L1054 | JT928327.1 | nuclease HARBI1 | 1.94E-162 | 85.60% | 619 |
| L1055 | JT937154.1 | Nuclear transcription factor Y subunit A-4 | 3.15E-79 | 82.40% | 495 |
| L1056 | JT936693.1 | Cytochrome c peroxidase, mitochondrial precursor | 2.15E-127 | 94.15% | 600 |
| L1057 | JT966693.1 | protein IQ-domain 1-like | 9.61E-18 | 79.30% | 456 |
| L1058 |  | nitrate transporter 1.5-like | 1.44E-44 | 79.40% | 354 |
| L1059 | JT925995.1 | latex abundant family protein | 6.96E-19 | 87.95% | 646 |
| L1060 | JR360115.1 | Rapid alkalinization factor | 4.21E-48 | 84.65% | 546 |
| L1061 | JT950594.1 | 60S ribosomal protein L10 | 1.69E-149 | 98.80% | 636 |
| L1062 | JT915263.1 | polyadenylation and cleavage factor | 1.13E-10 | 66.30% | 381 |
| L1063 | JT944690.1 | transcription regulator, putative | 5.28E-93 | 80.90% | 673 |
| L1064 | JR364959.1 | DNA binding protein* | 2.00E-48 | 75.00% | 442 |
| L1065 |  | mitochondrial fission protein ELM1-like | 6.81E-55 | 87.50% | 559 |
| L1066 | JT944407.1 | 50S ribosomal protein L3 | 1.01E-90 | 81.35% | 592 |
| L1067 | JR366204.1 | zinc finger CCCH domain-containing protein 13 | 1.00E-69 | 87.20% | 507 |
| L1068 | JT953517.1 | dessication responsive protein | 1.60E-53 | 95.25% | 509 |
| L1069 | JT950825.1 | HVA22-like protein I | 1.86E-100 | 91.50% | 561 |
| L1070 | JT953731.1 | RNA binding protein | 3.64E-18 | 87.35% | 604 |
| L1071 | JT939445.1 | protein trichome birefringence-like 43 | 1.74E-29 | 81.15% | 380 |
| L1072 | JR366225.1 | cell number regulator 8-like | 6.17E-45 | 77.00% | 552 |
| L1073 | JT957461.1 | macpf domain-containing cad1 -like protein | 6.77E-51 | 68.45% | 638 |
| L1074 | JT967915.1 | late embryogenesis abundant protein | 1.39E-32 | 63.30% | 616 |
| L1075 | JT969624.1 | Diacylglycerol Cholinephosphotransferase | 1.41E-66 | 97.45% | 608 |
| L1076 | JR344657.1 | adagio protein 1 | 6.60E-16 | 90.40% | 479 |
| L1077 | JT946738.1 |  |  |  | 442 |
| L1078 | JT950773.1 | ATP binding protein | 3.17E-63 | 77.05% | 601 |
| L1079 | JT951226.1 | nascent polypeptide-associated complex subunit alpha-like protein 1 | 7.79E-71 | 98.15% | 562 |
| L1080 | JT957717.1 | protein krtcap2 homolog | 2.95E-13 | 89.15% | 388 |
| L1081 | JT916078.1 | cytochrome dm13 and domon domain-containing protein at5g54830 | 8.88E-51 | 77.65% | 619 |
| L1082 | JT952856.1 | thioredoxin h9 | 7.66E-89 | 87.80% | 533 |
| L1083 | JT970685.1 | Protein BRICK1 | 1.93E-50 | 95.75% | 443 |
| L1084 | JT948029.1 | Ras-related protein Rabh1b | 2.76E-136 | 96.90% | 658 |
| L1085 | JT936552.1 | hypothetical protein RCOM_0038200 | 2.46E-23 | 81.10% | 616 |
| L1086 | JT961381.1 | U6 snRNA-associated sm-like protein lsm6 | 4.09E-49 | 100.00% | 545 |
| L1087 | JR346268.1 | transcription initiation factor tfIId subunit 10 | 8.30E-60 | 96.30% | 494 |
| L1088 | JT923344.1 | o-fucosyltransferase family protein isoform 1 | 1.45E-08 | 77.80% | 246 |
| L1089 | JT940798.1 | f-box family protein | 1.10E-78 | 86.45% | 691 |
| L1090 | JR365630.1 | fk506-binding protein 4-like | 3.24E-42 | 81.80% | 563 |
| L1091 | JT954366.1 | cyclophilin | 2.74E-110 | 95.95% | 718 |
| L1092 | JT917867.1 | Ubiquitin-associated ts-n domain-containing protein octicosapeptide phox bemp1 domain-containing | 5.24E-28 | 87.65% | 537 |
| L1093 | JT951963.1 | histone superfamily protein 2 | 3.45E-39 | 98.00% | 452 |
| L1094 | JT947159.1 | phosducin-like protein 3 | 7.03E-103 | 91.30% | 728 |
| L1095 | JR348135.1 | epidermal patterning factor-like protein 4 | 1.06E-45 | 82.45% | 508 |
| L1096 | JT926155.1 |  |  |  | 636 |
| L1097 |  | zinc finger A20 and AN1 domain-containing stress-associated protein 5-like | 1.78E-64 | 70.00% | 203 |
| L1098 | JT954896.1 | casp-like protein at2g28370 | 3.39E-46 | 84.80% | 643 |
| L1099 | JR346094.1 | 3-hydroxy-3-methylglutaryl coenzyme A reductase | 8.47E-85 | 77.20% | 537 |
| L1100 | JT955810.1 | 60S ribosomal protein L13a-4 | 2.87E-114 | 96.30% | 641 |
| L1101 | JT969484.1 | hypothetical protein L484_017734 | 3.12E-09 | 54.00% | 591 |
| L1102 | JT957990.1 | U6 snRNA-associated sm-like protein lsm1 | 9.51E-46 | 96.05% | 573 |
| L1103 | JT966323.1 |  |  |  | 533 |
| L1104 | JT921651.1 | hypothetical protein JCGZ_15057 | 2.26E-48 | 74.70% | 580 |
| L1105 | JR358381.1 | ethylene-responsive transcription factor rap2-12-like | 1.26E-18 | 88.95% | 510 |
| L1106 | JT918494.1 | WD repeat-containing protein 44-like | 4.29E-121 | 90.90% | 621 |
| L1107 | JT935831.1 | acyl-coenzyme A binding domain containing | 3.96E-69 | 72.80% | 673 |
| L1108 | JT954715.1 | transcription factor btf3 homolog 4-like | 6.56E-81 | 92.15% | 547 |
| L1109 | JT949811.1 | nucleic acid- OB-fold-like protein | 1.79E-65 | 97.00% | 697 |
| L1110 | JT941670.1 | zinc finger A20 and AN1 domain-containing stress-associated protein 5-like | 4.12E-35 | 86.30% | 690 |
| L1111 | JT919064.1 | heterogeneous nuclear ribonucleoprotein 1 | 1.32E-130 | 84.95% | 601 |
| L1112 |  | cytochrome b-c1 complex subunit rieske- mitochondrial-like | 1.47E-34 | 79.55% | 564 |
| L1113 | JR347363.1 | NC domain-containing family protein | 3.88E-27 | 83.55% | 510 |
| L1114 | JT944379.1 | 40S ribosomal protein S6 | 7.90E-78 | 96.85% | 706 |
| L1115 | JR349710.1 | hypothetical protein JCGZ_14316 | 2.79E-87 | 77.45% | 679 |
| L1116 | JT956924.1 | arginine Serine-rich splicing | 7.51E-23 | 75.00% | 647 |
| L1117 | JR366008.1 | iron-sulfur cluster assembly protein 1-like | 4.56E-119 | 88.25% | 740 |
| L1118 | JT936194.1 | Ribonuclease p protein subunit p29 isoform x1 | 4.44E-127 | 83.75% | 687 |
| L1119 | JT921374.1 | GDP-l-galactose phosphorylase | 3.67E-47 | 80.45% | 640 |
| L1120 | JT918084.1 | hypothetical protein JCGZ_19307 | 1.35E-07 | 79.00% | 617 |
| L1121 | JT953784.1 |  |  |  | 475 |
| L1122 | JT948039.1 |  |  |  | 614 |
| L1123 |  | heavy metal transport detoxification superfamily protein | 4.65E-70 | 79.25% | 632 |
| L1124 | JT964184.1 | kinase family protein | 2.26E-49 | 87.40% | 353 |
| L1125 | JT960631.1 | cytochrome B5 isoform 1 | 3.61E-45 | 83.65% | 490 |
| L1126 | JT972888.1 | mediator of RNA polymerase II transcription subunit 15a-like | 1.62E-16 | 81.30% | 346 |
| L1127 | JR362827.1 | ethylene-responsive element binding protein 2 | 2.59E-96 | 72.65% | 630 |
| L1128 | JT951078.1 | protein yippee-like at5g53940 | 6.97E-58 | 82.25% | 372 |
| L1129 | JT930692.1 | microtubule-associated protein | 4.01E-93 | 87.80% | 639 |
| L1130 | JT948309.1 | ormdl family protein | 8.55E-93 | 97.30% | 567 |
| L1131 | JT915293.1 | Ribosome biogenesis protein bms1 homolog isoform x2 | 6.28E-82 | 82.30% | 561 |
| L1132 | JT920459.1 | protein enhanced disease resistance 2-like isoform x1 | 7.27E-16 | 86.95% | 537 |
| L1133 | JT925156.1 | hypothetical protein JCGZ_08723 | 1.58E-22 | 69.25% | 490 |
| L1134 | JT919494.1 | dentin sialophosphoprotein-like isoform X4 | 5.36E-30 | 88.15% | 563 |
| L1135 | JR360452.1 | anaphase-promoting complex subunit 11 | 6.11E-33 | 91.50% | 323 |
| L1136 | JT947256.1 | Ubiquitin-associated translation elongation factor ef1b protein | 4.08E-42 | 85.30% | 317 |
| L1137 | JT937050.1 | syntaxin-42 isoform x2 | 5.58E-31 | 78.05% | 394 |
| L1138 | JT980186.1 | Serine threonine-protein kinase ht1 | 8.66E-23 | 76.90% | 513 |
| L1139 | JT966300.1 | PREDICTED: uncharacterized protein LOC103449856 | 1.41E-20 | 86.45% | 301 |
| L1140 | JT926681.1 | gamma aminobutyrate transaminase chloroplastic-like | 9.10E-51 | 94.90% | 289 |
| L1141 | JT944028.1 | rRNA-processing protein utp23 homolog | 3.75E-80 | 73.15% | 459 |
| L1142 | JT953254.1 | cytosolic Fe-S cluster assembly factor NAR1 | 5.54E-32 | 94.65% | 460 |
| L1143 | JR362058.1 | NADH dehydrogenase | 5.45E-42 | 77.75% | 354 |
| L1144 | JR347559.1 | probable 6-phosphogluconolactonase 1 | 2.75E-115 | 89.95% | 522 |
| L1145 | JT956116.1 | casp-like protein 5a1 | 2.92E-19 | 93.55% | 399 |
| L1146 | JT925597.1 |  |  |  | 550 |
| L1147 | JT956275.1 | Ras-related protein Rabe1a-like | 3.16E-58 | 99.90% | 504 |
| L1148 | JT926665.1 | target of myb protein 1-like | 1.94E-21 | 71.45% | 247 |
| L1149 | JT955532.1 | 60S ribosomal protein L23a | 3.51E-48 | 92.45% | 463 |
| L1150 | JR365645.1 | auxin-induced protein x15-like | 1.58E-53 | 85.35% | 460 |
| L1151 | JT923137.1 | U-box domain-containing protein 62-like | 3.43E-44 | 60.80% | 483 |
| L1152 | JT926422.1 | pyruvate cytosolic isozyme | 3.82E-13 | 91.60% | 479 |
| L1153 | JT933497.1 | inositol-tetrakisphosphate 1-kinase 1-like | 6.91E-90 | 75.95% | 606 |
| L1154 | JT948604.1 | 60S ribosomal protein L24 | 3.63E-79 | 95.45% | 496 |
| L1155 | JT955611.1 | kinesin light chain | 2.14E-62 | 69.00% | 467 |
| L1156 | JT957542.1 | UvrABC system C | 3.12E-38 | 92.10% | 520 |
| L1157 | JT956155.1 | embryo defective 2752 | 2.88E-57 | 93.60% | 111 |
| L1158 | JR349950.1 | protein n-lysine methyltransferase mettl21a-like | 1.54E-67 | 86.30% | 537 |
| L1159 | JR351174.1 | growth hormone receptor | 1.06E-28 | 80.05% | 441 |
| L1160 | JT945811.1 | hypothetical protein JCGZ_16104 | 2.32E-93 | 82.95% | 426 |
| L1161 | JT933822.1 | dolichyl-phosphate beta-glucosyltransferase-like | 2.81E-15 | 80.55% | 449 |
| L1162 | JT933676.1 | formin-like protein 8 | 3.22E-101 | 90.25% | 545 |
| L1163 | JT917663.1 | heat shock protein 70 | 1.76E-15 | 76.55% | 462 |
| L1164 | JT944840.1 | ATP synthase subunit delta mitochondrial-like | 5.21E-72 | 97.05% | 657 |
| L1165 | JT916235.1 | Serine arginine-rich splicing factor sr34a | 1.67E-91 | 88.05% | 347 |
| L1166 | JT936352.1 | E3 ubiquitin-protein ligase ring1-like | 2.13E-27 | 72.70% | 519 |
| L1167 | JT969792.1 |  |  |  | 539 |
| L1168 | JT949667.1 | ferredoxin i family protein | 3.24E-66 | 94.65% | 520 |
| L1169 | JT925661.1 | r3h domain containing protein | 4.54E-61 | 81.10% | 505 |
| L1170 | JR360041.1 | LOB domain-containing protein 37-like | 3.90E-82 | 87.55% | 523 |
| L1171 | JT922378.1 | probable polyamine oxidase 5 | 6.13E-88 | 87.15% | 559 |
| L1172 | JT955988.1 | thioredoxin h-type | 1.15E-23 | 90.00% | 525 |
| L1173 | JT945540.1 | cytochrome P450 704c1-like | 3.16E-80 | 92.35% | 458 |
| L1174 | JT967675.1 | hypothetical protein POPTR_0002s06090g | 1.19E-09 | 59.50% | 326 |
| L1175 | JT918263.1 | thioredoxin chloroplastic-like | 5.15E-77 | 65.15% | 462 |
| L1176 | JT941850.1 | adenine nucleotide alpha hydrolases-like protein | 1.62E-99 | 79.30% | 441 |
| L1177 | JT950781.1 | integrin-linked protein kinase family isoform 5 | 2.86E-30 | 88.80% | 526 |
| L1178 | JT945489.1 | 60S ribosomal protein L29-1-like | 1.99E-09 | 70.50% | 504 |
| L1179 | JT922687.1 | utp--glucose-1-phosphate uridylyltransferase-like isoform x1 | 9.89E-85 | 86.70% | 570 |
| L1180 | JT920607.1 | kinase interacting family isoform 2 | 7.58E-17 | 72.65% | 589 |
| L1181 | JT936957.1 | transcription factor bHLH68 | 1.19E-73 | 88.00% | 702 |
| L1182 |  | Rhicadhesin receptor precursor* | 7.00E-15 | 84.00% | 316 |
| L1183 | JT961110.1 | peroxidase 31 | 1.67E-66 | 92.15% | 577 |
| L1184 | JT954095.1 | hypothetical protein JCGZ_20968 | 3.77E-29 | 88.40% | 677 |
| L1185 | JT962615.1 | x-linked retinitis pigmentosa GTPase regulator-interacting protein 1-like | 3.04E-24 | 72.67% | 514 |
| L1186 | JR364404.1 | cysteine proteinase inhibitor | 2.62E-54 | 90.50% | 611 |
| L1187 | JT964469.1 | uncharacterized protein TCM_000929 | 2.40E-27 | 63.10% | 534 |
| L1188 | JT960331.1 | eukaryotic translation initiation factor 5A isoform VII (eIF-5A) | 1.45E-113 | 98.40% | 620 |
| L1189 | JT953558.1 | transmembrane protein 230 | 6.22E-35 | 82.20% | 675 |
| L1190 | JR350013.1 | altered inheritance of mitochondria protein 32-like | 1.13E-87 | 79.30% | 584 |
| L1191 | JT960147.1 |  |  |  | 549 |
| L1192 | JT933999.1 | pentatricopeptide repeat-containing protein mitochondrial-like | 5.60E-78 | 73.30% | 533 |
| L1193 | JT947901.1 | peptidyl-prolyl cis-trans isomerase cyp20- chloroplastic | 7.51E-111 | 96.65% | 687 |
| L1194 | JT956591.1 | Levanase | 1.48E-45 | 58.95% | 670 |
| L1195 | JR347484.1 | probable pectinesterase pectinesterase inhibitor 51 | 7.72E-46 | 87.20% | 488 |
| L1196 | JT961790.1 | protein spiral1-like 5 | 1.06E-33 | 78.15% | 560 |
| L1197 | JT953858.1 | phosphatidylinositolglycan-related family protein | 5.11E-55 | 85.90% | 595 |
| L1198 | JT950118.1 | conserved hypothetical protein | 6.08E-39 | 50.40% | 650 |
| L1199 | JT916938.1 | ABC1 family protein | 3.18E-53 | 74.10% | 671 |
| L1200 | JT935057.1 | BTB/POZ and TAZ domain-containing protein 1-like | 6.58E-58 | 64.55% | 624 |
| L1201 | JT918238.1 | Serine/threonine-protein kinase PBS1 | 1.70E-125 | 82.70% | 719 |
| L1202 | JR344294.1 | chaperonin cpn60- mitochondrial | 1.55E-135 | 98.30% | 605 |
| L1203 | JT944320.1 | Ran-binding protein 1 homolog b-like | 2.26E-102 | 89.00% | 715 |
| L1204 | JT925044.1 | histone-lysine n-methyltransferase ashh1-like | 2.92E-94 | 75.25% | 600 |
| L1205 | JR346449.1 | universal stress protein a-like protein | 1.96E-96 | 88.10% | 721 |
| L1206 | JT951739.1 | proteasome subunit beta type-3-a | 3.22E-145 | 96.95% | 646 |
| L1207 | JT971673.1 |  |  |  | 516 |
| L1208 | JT963106.1 | f-box protein skip24 | 8.47E-65 | 79.45% | 536 |
| L1209 | JT961342.1 | 60S ribosomal protein L27 | 9.27E-67 | 94.90% | 575 |
| L1210 | JR362259.1 |  |  |  | 534 |
| L1211 | JR348915.1 | mateRNAl effect embryo arrest 9 | 5.80E-51 | 83.75% | 683 |
| L1212 | JT977586.1 | programmed cell death protein | 3.76E-46 | 91.50% | 622 |
| L1213 | JT945380.1 | MYB-like transcription factor family protein isoform 1 | 3.32E-106 | 96.20% | 607 |
| L1214 | JT933200.1 | probable methyltransferase pmt2 | 8.26E-134 | 89.05% | 693 |
| L1215 | JT956072.1 | acyl carrier protein mitochondrial | 2.61E-81 | 88.20% | 659 |
| L1216 | JT945682.1 | pre-mRNA branch site p14-like protein | 1.82E-83 | 96.75% | 645 |
| L1217 | JR360853.1 | embryo defective 1303 | 1.15E-35 | 62.35% | 507 |
| L1218 | JT968842.1 | E3 ubiquitin-protein ligase atl23-like | 2.56E-78 | 81.45% | 656 |
| L1219 | JT957678.1 | acyl carrier protein chloroplastic-like | 3.15E-54 | 86.15% | 621 |
| L1220 | JT949431.1 | phosphoribosylformylglycinamidine synthase 1 | 3.60E-82 | 95.25% | 644 |
| L1221 | JR360371.1 | protein spiral1-like 1 | 2.57E-41 | 72.10% | 650 |
| L1222 | JR345582.1 | uncharacterized protein TCM_020114 | 7.61E-10 | 63.00% | 488 |
| L1223 | JT952708.1 | uncharacterized protein isoform 1 | 4.96E-56 | 81.50% | 675 |
| L1224 | JT953630.1 | membrane magnesium transporter | 1.07E-55 | 89.65% | 502 |
| L1225 | JT961540.1 | senescence-associated protein din1 | 3.69E-51 | 81.50% | 612 |
| L1226 | JT956574.1 | 60S ribosomal protein L18a-like protein | 1.30E-46 | 77.45% | 682 |
| L1227 | JR345627.1 | hypothetical protein JCGZ_08192 | 4.60E-11 | 65.00% | 662 |
| L1228 | JT954080.1 | protein kish-like | 1.03E-40 | 96.55% | 666 |
| L1229 | JT955412.1 | Spindle and kinetochore-associated 2 | 8.97E-66 | 86.30% | 609 |
| L1230 | JT943520.1 | electron transporter | 4.18E-79 | 83.60% | 616 |
| L1231 | JT952583.1 | Ras-related protein Rabb1b | 5.47E-109 | 97.10% | 685 |
| L1232 | JR353243.1 |  |  |  | 268 |
| L1233 | JT962766.1 | Cu/Zn superoxide dismutase | 2.20E-92 | 86.70% | 593 |
| L1234 | JT945747.1 | 60S ribosomal protein L9 | 4.54E-99 | 96.70% | 704 |
| L1235 |  | UDP-glycosyltransferase 85a1 | 2.69E-112 | 87.80% | 746 |
| L1236 | JR366023.1 | hypothetical protein JCGZ_23324 | 7.00E-06 | 93.00% | 645 |
| L1237 | JT947221.1 | NC domain-containing isoform partial | 2.98E-64 | 90.00% | 554 |
| L1238 | JT952825.1 | protein transport protein sec61 subunit beta-like | 7.12E-24 | 93.55% | 579 |
| L1239 | JR359031.1 | hypersensitive-induced response protein 4 | 1.11E-64 | 94.90% | 469 |
| L1240 | JT961146.1 | histone H3 | 3.79E-77 | 99.65% | 565 |
| L1241 | JT939630.1 | ferrochelatase-2 chloroplastic | 7.51E-17 | 70.35% | 476 |
| L1242 | JT968550.1 |  |  |  | 638 |
| L1243 | JT929990.1 | lanc-like protein gcr2 | 1.87E-131 | 93.00% | 614 |
| L1244 | JR344348.1 | 60S ribosomal protein L44 | 6.82E-67 | 99.05% | 556 |
| L1245 | JT945976.1 | uncharacterized loc101218430 | 2.24E-66 | 78.00% | 646 |
| L1246 | JT958256.1 | histidine triad nucleotide-binding protein 3 | 6.12E-75 | 82.65% | 625 |
| L1247 | JR365315.1 | actin-depolymerizing factor 2 | 1.68E-97 | 96.95% | 587 |
| L1248 | JR351774.1 | hypothetical protein CICLE_v10013263mg | 2.99E-06 | 86.00% | 666 |
| L1249 | JT936517.1 | macrophage erythroblast attacher-like | 1.48E-29 | 77.35% | 502 |
| L1250 | JT955901.1 | hypothetical protein POPTR_0006s13960g | 7.84E-37 | 76.80% | 555 |
| L1251 | JT953671.1 | l-lactate dehydrogenase a-like | 7.29E-130 | 97.35% | 686 |
| L1252 | JT944105.1 | protein pxr1-like | 3.07E-43 | 87.55% | 599 |
| L1253 | JT936073.1 | Serine threonine-protein kinase pepkr2 | 7.65E-20 | 81.90% | 535 |
| L1254 | JT930971.1 | GTP binding protein beta 1 isoform 1 | 1.66E-70 | 95.65% | 720 |
| L1255 | JT938417.1 | upf0613 protein | 2.10E-88 | 94.20% | 630 |
| L1256 | JT925217.1 | protein cypro4-like | 2.03E-73 | 97.05% | 489 |
| L1257 | JT958572.1 | mitochondrial import inner membrane translocase subunit Tim14-1 | 1.92E-58 | 93.50% | 707 |
| L1258 | JT925820.1 | 2,3-bisphosphoglycerate-independent phosphoglycerate mutase | 1.24E-94 | 94.95% | 663 |
| L1259 | JT958583.1 | 40S ribosomal protein S17 | 2.10E-86 | 96.45% | 630 |
| L1260 | JT949186.1 | glutaredoxin family protein | 1.57E-55 | 64.00% | 564 |
| L1261 | JT953892.1 | squamosa promoter-binding protein 1 | 6.24E-55 | 79.95% | 612 |
| L1262 | JT961762.1 | Coiled-coil domain-containing protein* | 4.00E-103 | 80.00% | 465 |
| L1263 | JT961162.1 | hypothetical protein JCGZ_14189 | 1.17E-24 | 82.60% | 518 |
| L1264 | JT962975.1 |  |  |  | 611 |
| L1265 | JT922709.1 | auxin response factor 8 | 1.44E-118 | 88.15% | 649 |
| L1266 | JT948152.1 | nematode resistance protein-like HSPRO2 | 1.46E-100 | 84.45% | 714 |
| L1267 | JR365737.1 | elongation factor 1-gamma-like | 4.93E-125 | 89.70% | 597 |
| L1268 | JR365329.1 | adrenodoxin-like mitochondrial | 1.06E-43 | 97.05% | 459 |
| L1269 | JT926641.1 | probable protein phosphatase 2c 38 isoform x1 | 4.01E-54 | 89.20% | 591 |
| L1270 | JT954779.1 | autophagy-related protein 8f | 2.87E-70 | 95.65% | 597 |
| L1271 | JT970521.1 | calcium-dependent lipid-binding family protein | 7.66E-12 | 94.15% | 626 |
| L1272 | JR366135.1 | protein tic 20- chloroplastic | 3.49E-67 | 84.20% | 579 |
| L1273 | JT957000.1 | mateRNAl effect embryo arrest 9 | 2.85E-39 | 87.95% | 707 |
| L1274 | JT937284.1 | CBL-interacting Serine threonine-protein kinase 23 isoform x1 | 3.14E-143 | 95.85% | 693 |
| L1275 | JR361721.1 | eukaryotic translation initiation factor 3 subunit f-like | 1.85E-146 | 93.45% | 674 |
| L1276 | JT932005.1 | mar-binding filament-like protein 1 isoform 1 | 7.53E-99 | 91.45% | 486 |
| L1277 | JT925829.1 | DNA-3-methyladenine glycosylase | 3.02E-39 | 60.95% | 583 |
| L1278 | JT955258.1 | eukaryotic initiation factor 4a | 4.16E-70 | 86.55% | 655 |
| L1279 | JT918495.1 | Vacuolar protein sorting-associated protein 28 homolog 2 | 2.74E-86 | 96.50% | 602 |
| L1280 |  | protein mnn4-like | 2.61E-32 | 64.70% | 642 |
| L1281 | JT944368.1 | lipid phosphate phosphatase 2 | 1.08E-63 | 93.50% | 547 |
| L1282 | JT914660.1 | multiple C2 and transmembrane domain-containing protein 1-like | 8.97E-81 | 98.15% | 632 |
| L1283 | JT962046.1 | copper transport protein ATOX1 | 2.62E-37 | 94.25% | 574 |
| L1284 | JT971169.1 | hypothetical protein JCGZ_16091 | 1.44E-32 | 68.92% | 542 |
| L1285 | JT949681.1 | Ubiquitin-conjugating enzyme e2 28 | 1.23E-87 | 99.50% | 621 |
| L1286 | JT957809.1 | 40S ribosomal protein S2-4-like | 1.01E-82 | 97.15% | 551 |
| L1287 | JT969848.1 | protease inhibitor protein 1 (PI1) | 6.15E-43 | 78.05% | 494 |
| L1288 | JT940745.1 | ino80 complex subunit d-like | 1.35E-63 | 84.55% | 563 |
| L1289 | JR366779.1 | protein ELC-like | 2.86E-85 | 85.25% | 709 |
| L1290 | JR366277.1 | selenoprotein | 1.73E-69 | 89.05% | 641 |
| L1291 | JT957418.1 | cupredoxin superfamily | 7.53E-19 | 77.70% | 574 |
| L1292 | JT919672.1 | NADP-specific glutamate dehydrogenase | 1.33E-114 | 95.65% | 725 |
| L1293 | JT931304.1 | transcription elongation factor s-II | 1.27E-95 | 73.65% | 583 |
| L1294 | JT926958.1 | DUF674 family protein | 5.57E-53 | 68.35% | 611 |
| L1295 | JT948986.1 | syntaxin-52-like isoform x1 | 2.47E-122 | 90.10% | 653 |
| L1296 | JT938775.1 | Serine acetyltransferase 5-like | 3.06E-79 | 95.25% | 659 |
| L1297 | JT945519.1 | Ubiquitin-conjugating enzyme 34 isoform 1 | 1.08E-35 | 78.40% | 443 |
| L1298 | JT964163.1 | transcription factor lux-like | 6.32E-09 | 97.95% | 596 |
| L1299 | JT937841.1 | protein auxin-regulated gene involved in organ size-like | 9.21E-29 | 79.89% | 507 |
| L1300 | JT961328.1 | conserved hypothetical protein | 1.07E-07 | 56.75% | 339 |
| L1301 | JT915296.1 | nucleotide binding protein | 1.83E-113 | 89.70% | 624 |
| L1302 | JT956270.1 | josephin-like protein | 3.52E-75 | 81.15% | 538 |
| L1303 | JT919930.1 | kinesin light chain | 1.22E-75 | 89.45% | 496 |
| L1304 | JT949037.1 | kinase superfamily protein isoform 1 | 8.50E-71 | 75.55% | 611 |
| L1305 | JT962128.1 | conserved hypothetical protein | 5.46E-44 | 90.60% | 549 |
| L1306 | JT952552.1 | CC-NBS-LRR resistance isoform 1 | 1.89E-48 | 69.60% | 699 |
| L1307 | JT966013.1 | outer envelope pore protein chloroplastic-like | 3.14E-90 | 75.80% | 633 |
| L1308 | JT953624.1 | 40S ribosomal protein S10-like | 2.67E-64 | 95.45% | 462 |
| L1309 | JR350010.1 | bes1 bzr1 homolog protein 4 | 9.21E-26 | 99.05% | 674 |
| L1310 | JT928456.1 | NADP-dependent glyceraldehyde-3-phosphate dehydrogenase-like | 1.09E-93 | 99.95% | 645 |
| L1311 | JR344903.1 | glutamine synthetase | 7.71E-25 | 97.40% | 500 |
| L1312 | JR364978.1 | latex abundant protein 1 | 1.83E-77 | 68.30% | 582 |
| L1313 | JT928201.1 | ankyrin repeat domain-containing protein 13b-like | 1.21E-06 | 89.85% | 198 |
| L1314 | JT957411.1 | zinc finger and btb domain-containing protein 47-like | 2.82E-16 | 64.00% | 667 |
| L1315 | JT937079.1 | membrin-11-like | 3.32E-75 | 88.80% | 631 |
| L1316 | JT961312.1 | calmodulin | 6.72E-90 | 100.00% | 711 |
| L1317 | JT976706.1 |  |  |  | 530 |
| L1318 | JT959474.1 | 60S ribosomal protein L22-2 | 1.93E-56 | 98.65% | 670 |
| L1319 | JT931123.1 | ATP synthase subunit mitochondrial | 7.23E-56 | 99.05% | 632 |
| L1320 | JT922906.1 | clathrin assembly protein at5g35200 | 6.55E-31 | 96.25% | 481 |
| L1321 | JT957808.1 | lyr motif-containing protein 7 isoform 2 | 5.93E-44 | 82.65% | 663 |
| L1322 | JT947192.1 | membrane-anchored ubiquitin-fold protein 6 precursor | 3.71E-56 | 89.75% | 619 |
| L1323 | JT952827.1 | hypothetical protein JCGZ_14408 | 3.27E-54 | 74.30% | 604 |
| L1324 | JT949945.1 | charged multivesicular body protein 1-like | 8.27E-100 | 97.25% | 636 |
| L1325 | JT955448.1 | dual specificity phosphatase cdc25 | 3.58E-81 | 89.35% | 614 |
| L1326 | JT965638.1 |  |  |  | 464 |
| L1327 | JT918389.1 | E3 ubiquitin-protein ligase rbbp6 | 7.63E-19 | 83.05% | 657 |
| L1328 | JR345548.1 | histone H3 | 3.12E-91 | 98.85% | 599 |
| L1329 | JT960020.1 | Ubiquitin-40S ribosomal protein s27a | 8.99E-90 | 98.30% | 604 |
| L1330 | JT936894.1 | myosin phosphatase rho-interacting | 2.00E-78 | 85.85% | 672 |
| L1331 | JT957838.1 | thioredoxin h2 | 4.02E-61 | 85.00% | 648 |
| L1332 | JT970820.1 |  |  |  | 513 |
| L1333 | JT959313.1 | histone H2B | 7.70E-22 | 100.00% | 488 |
| L1334 | JT970768.1 | coproporphyrinogen III oxidase | 4.04E-18 | 86.65% | 721 |
| L1335 | JT955364.1 | Cucumber peeling cupredoxin | 2.96E-40 | 64.10% | 659 |
| L1336 | JT922522.1 | enhancer of polycomb-like transcription factor protein | 2.53E-41 | 59.15% | 513 |
| L1337 | JT931303.1 | thioredoxin family protein isoform 1 | 7.48E-85 | 87.20% | 609 |
| L1338 | JT939981.1 | Ubiquitin-conjugating enzyme e2-17 kDa | 4.21E-105 | 99.30% | 628 |
| L1339 | JR348253.1 | CDGSH iron-sulfur domain-containing 2A | 4.03E-46 | 83.05% | 594 |
| L1340 | JT970632.1 | bidirectional sugar transporter sweet17-like | 2.22E-32 | 79.10% | 705 |
| L1341 | JT965549.1 |  |  |  | 568 |
| L1342 | JT914815.1 | metal transporter nramp5 | 3.14E-84 | 88.25% | 650 |
| L1343 | JR366829.1 | sucrose transporter 1 | 9.00E-62 | 99.00% | 482 |
| L1344 | JT936720.1 | ATP binding protein | 2.93E-80 | 76.30% | 646 |
| L1345 | JR348962.1 | OBP3-responsive gene 4 isoform 1 | 7.11E-87 | 91.50% | 706 |
| L1346 | JT928410.1 | phospho-2-dehydro-3-deoxyheptonate aldolase chloroplastic-like | 7.10E-127 | 97.80% | 531 |
| L1347 | JT962044.1 |  |  |  | 553 |
| L1348 | JT918586.1 | phosphatidic acid phosphatase | 1.87E-51 | 77.65% | 653 |
| L1349 | JT941842.1 | 50S ribosomal protein L19 | 1.09E-55 | 95.10% | 764 |
| L1350 | JT966825.1 | b-cell receptor-associated protein 31 | 2.74E-85 | 86.50% | 724 |
| L1351 | JT959078.1 | probable ubiquitin-conjugating enzyme E2 18 | 7.52E-66 | 97.30% | 632 |
| L1352 | JT955601.1 | outer envelope pore protein 16- chloroplastic | 2.70E-64 | 90.10% | 523 |
| L1353 | JT917391.1 | ADP-ribosylation factor GTPase-activating protein agd3 | 1.15E-18 | 91.90% | 552 |
| L1354 | JT947534.1 | Ras-GTPase-activating protein-binding protein | 2.48E-11 | 80.95% | 510 |
| L1355 | JR365416.1 | hypothetical protein JCGZ_08198 | 1.49E-60 | 81.80% | 657 |
| L1356 | JT929971.1 | 3-hydroxy-3-methylglutaryl coenzyme A reductase | 1.73E-66 | 98.00% | 445 |
| L1357 | JR364527.1 | NADH dehydrogenase | 1.22E-29 | 88.30% | 574 |
| L1358 | JT940187.1 | kinesin-4 -like protein | 3.51E-07 | 51.77% | 631 |
| L1359 | JT938948.1 | flavohemoprotein B5/b5r | 9.24E-73 | 93.95% | 583 |
| L1360 | JR366055.1 | MOB kinase activator-like 1 | 2.21E-134 | 98.30% | 593 |
| L1361 | JT945605.1 | malate dehydrogenase | 3.30E-41 | 95.35% | 514 |
| L1362 | JT950096.1 | 60S ribosomal protein L23 | 1.98E-71 | 99.95% | 534 |
| L1363 | JT921411.1 | hypothetical protein POPTR_0002s04800g | 7.74E-07 | 76.33% | 639 |
| L1364 | JT946712.1 | wound-responsive family protein | 6.47E-34 | 68.30% | 626 |
| L1365 | JT948119.1 | probable receptor-like protein kinase at1g67000 | 1.26E-115 | 79.60% | 733 |
| L1366 | JT962509.1 |  |  |  | 398 |
| L1367 | JT962184.1 | NADH-ubiquinone oxidoreductase 11 kDa subunit | 1.09E-57 | 93.40% | 607 |
| L1368 | JR349711.1 | aspartate kinase family protein | 1.11E-51 | 95.15% | 512 |
| L1369 | JT961447.1 | squamosa promoter-binding-like protein 13a | 6.80E-34 | 55.05% | 630 |
| L1370 | JT942690.1 | 1-aminocyclopropane-1-carboxylate deaminase | 1.77E-38 | 90.30% | 616 |
| L1371 | JT958283.1 |  |  |  | 619 |
| L1372 | JR345859.1 | small GTP-binding protein, RAB1C | 4.96E-88 | 97.20% | 624 |
| L1373 | JT964777.1 | Ubiquitin domain-containing protein 1-like | 9.17E-63 | 97.60% | 542 |
| L1374 | JR365556.1 | hypothetical protein JCGZ_17560 | 1.75E-59 | 73.50% | 625 |
| L1375 | JR350044.1 | vq motif-containing family protein | 2.80E-25 | 60.65% | 648 |
| L1376 | JT923459.1 | Serine hydroxymethyltransferase chloroplastic-like | 5.03E-127 | 95.90% | 558 |
| L1377 | JT962538.1 | metallothionein-like protein | 1.50E-22 | 80.70% | 644 |
| L1378 | JT951815.1 |  |  |  | 706 |
| L1379 | JT948056.1 | auxin-repressed 12.5 kDa | 4.99E-59 | 83.90% | 692 |
| L1380 | JR363077.1 |  |  |  | 591 |
| L1381 | JR350021.1 | transcription factor vip1 | 4.64E-16 | 71.27% | 619 |
| L1382 | JT977530.1 | fimbrin-like family protein | 1.31E-17 | 80.90% | 478 |
| L1383 | JT970232.1 | hypothetical protein JCGZ_03337 | 3.71E-16 | 59.60% | 591 |
| L1384 | JT958666.1 | dentin sialophosphoprotein-like isoform X1 | 3.81E-59 | 75.60% | 656 |
| L1385 | JR357173.1 | uncharacterized protein LOC100527274 | 8.50E-12 | 80.86% | 526 |
| L1386 | JT960639.1 | thiamine-phosphate synthase 2 | 4.43E-71 | 88.35% | 553 |
| L1387 | JT953304.1 |  |  |  | 566 |
| L1388 | JT947231.1 | calmodulin | 4.40E-32 | 98.10% | 615 |
| L1389 | JT954003.1 | skp1-like protein | 1.12E-107 | 75.45% | 632 |
| L1390 | JT932819.1 | probable Serine incorporator | 3.67E-73 | 92.55% | 595 |
| L1391 | JT942502.1 | Transmembrane emp24 domain-containing protein 10 precursor | 2.66E-95 | 85.20% | 677 |
| L1392 | JT927121.1 | chaperone protein DNAj 49 | 6.61E-19 | 91.05% | 576 |
| L1393 | JT916665.1 | auxin response factor 8-like | 2.97E-14 | 86.00% | 602 |
| L1394 | JT941873.1 | 60S acidic ribosomal protein P1 | 1.62E-30 | 79.95% | 689 |
| L1395 | JR366189.1 | Serine/arginine repetitive matrix protein 2 | 7.43E-52 | 78.10% | 686 |
| L1396 | JT945835.1 | vesicle-associated membrane protein 727 | 2.07E-111 | 93.75% | 688 |
| L1397 | JT946245.1 | C2 domain-containing family protein | 1.57E-28 | 52.80% | 667 |
| L1398 | JT919173.1 | zinc finger family protein | 1.43E-24 | 83.75% | 646 |
| L1399 | JT942284.1 | 1-aminocyclopropane-1-carboxylate oxidase-like | 3.77E-101 | 86.50% | 711 |
| L1400 | JT954648.1 |  |  |  | 605 |
| L1401 | JT980862.1 |  |  |  | 653 |
| L1402 | JT926483.1 | Aspartic proteinase-like protein 2 | 5.10E-18 | 91.50% | 536 |
| L1403 | JT952184.1 | Rop guanine nucleotide exchange factor 7-like | 6.56E-125 | 85.70% | 678 |
| L1404 | JR365132.1 | hemiasterlin resistant protein 1 | 2.81E-39 | 70.80% | 668 |
| L1405 | JT958905.1 | muscle m-line assembly protein unc-89-like isoform x1 | 2.84E-77 | 70.40% | 706 |
| L1406 | JT953460.1 | DNAj protein homolog 2-like | 4.92E-64 | 85.50% | 617 |
| L1407 | JT952663.1 | Vacuolar protein sorting-associated protein 55 homolog | 1.41E-66 | 91.35% | 639 |
| L1408 | JT927532.1 | probable ADP-ribosylation factor GTPase-activating protein agd5 | 6.01E-61 | 73.60% | 678 |
| L1409 | JT936130.1 | magnesium transporter -like family protein | 9.26E-86 | 90.10% | 670 |
| L1410 | JT959748.1 | web family protein at3g51220-like | 4.26E-83 | 75.50% | 561 |
| L1411 | JT963371.1 | hypothetical protein PRUPE_ppa014087mg | 6.34E-32 | 93.65% | 501 |
| L1412 | JR344489.1 | Ribosomal L18p/L5e family protein | 1.60E-65 | 83.95% | 620 |
| L1413 | JR356323.1 | PREDICTED: uncharacterized protein LOC101509988 | 9.21E-10 | 90.80% | 613 |
| L1414 | JT943015.1 | tetratricopeptide repeat protein 1-like | 1.40E-109 | 90.95% | 720 |
| L1415 | JT964452.1 | probable proteasome inhibitor | 3.95E-77 | 85.65% | 614 |
| L1416 | JT947924.1 | hypothetical protein JCGZ_22669 | 1.41E-91 | 92.05% | 645 |
| L1417 | JT959445.1 | uncharacterized loc101215337 | 1.28E-50 | 81.80% | 575 |
| L1418 | JR349302.1 |  |  |  | 674 |
| L1419 | JR348803.1 | conserved hypothetical protein | 7.40E-86 | 91.85% | 673 |
| L1420 | JT965774.1 | 40S ribosomal protein S28-like | 2.39E-28 | 99.40% | 510 |
| L1421 | JT950585.1 | n-alpha-acetyltransferase 38- auxiliary subunit | 7.45E-50 | 95.30% | 684 |
| L1422 | JT935885.1 | phosphoribosylamine-glycine ligase | 7.02E-107 | 80.25% | 690 |
| L1423 | JT946009.1 | Ubiquitin-conjugating enzyme e2 variant 1d | 1.71E-79 | 98.70% | 633 |
| L1424 | JT958056.1 | zinc finger protein 593 | 3.24E-71 | 93.05% | 635 |
| L1425 | JT959572.1 | nudc domain-containing protein 2-like | 2.94E-98 | 95.20% | 691 |
| L1426 | JT931152.1 | E3 ubiquitin-protein ligase RNF181 | 1.53E-25 | 76.70% | 614 |
| L1427 | JT970233.1 | polyubiquitin | 5.90E-06 | 44.00% | 628 |
| L1428 | JT951761.1 | DOF zinc finger protein | 2.39E-100 | 79.90% | 635 |
| L1429 | JT955944.1 | Ubiquitin-like protein 5 | 1.58E-42 | 97.45% | 400 |
| L1430 | JT959841.1 | dihydroflavonol 4-reductase | 8.28E-66 | 75.35% | 568 |
| L1431 | JT916433.1 | kinesin-like protein kif3a-like isoform x1 | 1.06E-17 | 60.00% | 646 |
| L1432 | JT960825.1 | Cucumber peeling cupredoxin | 1.76E-92 | 73.80% | 594 |
| L1433 | JT944001.1 | fatty acid 2-hydroxylase 1-like | 2.73E-142 | 90.70% | 627 |
| L1434 | JT935714.1 | cryptochrome 1 family protein | 9.18E-67 | 96.45% | 620 |
| L1435 | JT926372.1 | probable E3 ubiquitin ligase sud1 | 3.82E-81 | 97.70% | 473 |
| L1436 | JT916016.1 | inactive cadmium zinc-transporting ATPase hma3 isoform x1 | 1.33E-19 | 58.25% | 652 |
| L1437 | JT931448.1 | peptide methionine sulfoxide reductase-like | 3.55E-112 | 91.70% | 688 |
| L1438 | JR366192.1 | exostosin family protein | 1.81E-145 | 90.45% | 564 |
| L1439 | JT943152.1 |  |  |  | 481 |
| L1440 | JT959321.1 | hypothetical protein JCGZ_07980 | 1.50E-58 | 65.55% | 646 |
| L1441 | JT939366.1 | annexin d3-like | 1.79E-79 | 84.25% | 690 |
| L1442 | JT961379.1 | Ubiquitin-fold modifier 1 | 3.19E-51 | 99.50% | 591 |
| L1443 | JT952181.1 | small rubber particle protein | 3.31E-64 | 67.40% | 682 |
| L1444 | JT951738.1 | outer envelope pore protein 16- chloroplastic mitochondrial | 1.09E-91 | 93.75% | 640 |
| L1445 | JT953615.1 | cofactor assembly | 1.58E-72 | 76.10% | 669 |
| L1446 | JT944101.1 | mitochondrial inner membrane protease subunit 1 | 2.39E-123 | 90.20% | 648 |
| L1447 | JT929112.1 | senescence-associated protein | 1.21E-59 | 80.45% | 644 |
| L1448 | JT962024.1 | prefoldin chaperone subunit family protein | 1.53E-26 | 83.25% | 567 |
| L1449 | JT963879.1 | Serine arginine repetitive matrix protein 2 | 1.32E-45 | 74.70% | 479 |
| L1450 | JT943929.1 | conserved hypothetical protein | 1.24E-44 | 75.45% | 667 |
| L1451 | JT934763.1 | RING finger and CHY zinc finger domain-containing protein 1 isoform 2 | 4.58E-65 | 70.60% | 640 |
| L1452 | JT966237.1 | zeaxanthin chloroplastic | 6.34E-98 | 76.95% | 612 |
| L1453 | JR349010.1 | histidine triad (hit) protein | 3.06E-105 | 77.45% | 616 |
| L1454 | JT923061.1 | protein chromatin remodeling 4-like | 7.06E-102 | 64.90% | 660 |
| L1455 | JT938603.1 | neurofilament medium polypeptide | 2.28E-94 | 67.55% | 640 |
| L1456 | JT932065.1 | shaggy-related protein kinase alpha | 4.37E-75 | 97.05% | 696 |
| L1457 | JT946229.1 | glycine-rich isoform 1 | 3.48E-107 | 78.00% | 580 |
| L1458 | JT937220.1 | Ribosomal protein S1 | 6.98E-122 | 74.90% | 646 |
| L1459 | JR360599.1 | hypothetical protein POPTR_0011s11040g | 1.85E-14 | 89.50% | 227 |
| L1460 | JT951127.1 |  |  |  | 614 |
| L1461 | JT966094.1 |  |  |  | 527 |
| L1462 | JT947734.1 | protein ELF4-like 4 | 2.22E-63 | 95.65% | 613 |
| L1463 | JT923166.1 | Ubiquitin-protein ligase BRE1A | 5.92E-64 | 65.55% | 584 |
| L1464 | JR348395.1 | zinc finger protein nutcracker-like isoform x1 | 2.13E-53 | 64.70% | 594 |
| L1465 | JT932830.1 | DNA-binding protein RAV1 | 2.72E-83 | 87.05% | 665 |
| L1466 | JT969072.1 | general transcription factor IIe subunit 1-like | 7.41E-83 | 96.05% | 223 |
| L1467 | JR366362.1 | uncharacterized loc101222871 | 4.99E-89 | 82.00% | 619 |
| L1468 | JR364064.1 | reactive oxygen species modulator 1 | 9.04E-27 | 98.50% | 534 |
| L1469 | JR349531.1 | arginine/Serine-rich splicing factor | 2.52E-100 | 94.55% | 611 |
| L1470 | JT966488.1 | phytosulfokines 6 | 1.23E-12 | 71.55% | 533 |
| L1471 | JT956590.1 | localized to the inner membrane of the chloroplast | 5.33E-80 | 77.95% | 678 |
| L1472 | JT946920.1 | probable RNA-binding protein EIF1AD-like | 9.20E-69 | 90.90% | 694 |
| L1473 | JT944980.1 | gcn5-related n-acetyltransferase family protein | 5.70E-88 | 66.95% | 673 |
| L1474 | JT933079.1 | pyruvate dehydrogenase e1 component subunit beta- chloroplastic-like | 1.55E-72 | 99.60% | 348 |
| L1475 | JT931478.1 | actin | 5.72E-77 | 99.45% | 447 |
| L1476 | JT940910.1 | choline-phosphate cytidylyltransferase 1-like | 3.59E-108 | 91.20% | 625 |
| L1477 | JR366193.1 | Fe(2+) transport protein chloroplastic-like | 6.74E-87 | 80.45% | 699 |
| L1478 | JT967660.1 | conserved hypothetical protein | 1.32E-33 | 71.10% | 654 |
| L1479 | JR364866.1 | RING-H2 zinc finger protein | 8.32E-67 | 81.25% | 633 |
| L1480 | JT934279.1 | amsh-like ubiquitin thioesterase 3 | 4.15E-40 | 84.35% | 644 |
| L1481 | JT956029.1 | V-type proton ATPase subunit e-like | 1.58E-40 | 95.15% | 620 |
| L1482 | JT947283.1 | protein rtoa-like | 5.21E-38 | 75.20% | 577 |
| L1483 | JT957337.1 | 60S ribosomal protein L21 isoform 1 | 2.17E-112 | 97.20% | 523 |
| L1484 | JT924417.1 | chaperonin cpn60- mitochondrial | 2.50E-62 | 96.95% | 499 |
| L1485 | JT949571.1 | eukaryotic translation initiation factor 5a-like | 1.01E-84 | 97.60% | 693 |
| L1486 | JT962286.1 |  |  |  | 634 |
| L1487 | JT927099.1 | Serine threonine-protein kinase ht1-like | 1.78E-07 | 88.95% | 302 |
| L1488 | JR349576.1 | forkhead box protein isoform 1 | 9.53E-64 | 65.75% | 714 |
| L1489 | JT940515.1 | UDP-glucuronate 4-epimerase 3-like | 7.60E-63 | 91.10% | 659 |
| L1490 | JT964843.1 | major allergen Pru ar 1-like protein | 1.44E-12 | 69.81% | 565 |
| L1491 | JT964965.1 | conserved hypothetical protein* | 5.00E-58 | 73.00% | 576 |
| L1492 | JT945451.1 | sterile alpha motif domain-containing protein | 6.19E-93 | 66.65% | 683 |
| L1493 | JT961246.1 | f-box family protein | 6.94E-14 | 65.35% | 668 |
| L1494 | JT931343.1 | RING-H2 finger protein atl65-like | 4.21E-07 | 74.17% | 681 |
| L1495 | JT929407.1 | protein transparent testa 12-like | 5.53E-40 | 91.00% | 556 |
| L1496 | JR365121.1 | hypothetical protein JCGZ_06146 | 7.86E-72 | 85.50% | 656 |
| L1497 | JR365413.1 | glycine-rich RNA-binding protein* | 4.00E-35 | 75.00% | 691 |
| L1498 | JT958732.1 | rpm1-interacting protein 4-like | 1.25E-38 | 84.20% | 534 |
| L1499 | JT936619.1 | bidirectional sugar transporter SWEET10-like | 1.68E-108 | 83.50% | 593 |
| L1500 | JT917363.1 | beta-galactosidase 3 | 5.57E-32 | 91.95% | 658 |
| L1501 | JT939820.1 | probable glutathione peroxidase 8 | 2.99E-59 | 90.45% | 646 |
| L1502 | JR349376.1 | nodulin-related protein | 2.60E-24 | 72.25% | 580 |
| L1503 | JT918309.1 | benzoyl- reductase subunit c | 4.10E-76 | 75.20% | 706 |
| L1504 | JT962614.1 | hypothetical protein JCGZ_11378 | 5.56E-44 | 54.11% | 662 |
| L1505 | JR347816.1 | copper methylamine oxidase | 2.26E-47 | 77.85% | 701 |
| L1506 | JT952191.1 | otu domain-containing protein 5 | 2.60E-19 | 79.80% | 503 |
| L1507 | JT961135.1 | polyubiquitin | 1.22E-80 | 99.95% | 624 |
| L1508 | JT926847.1 | ferrochelatase- chloroplastic | 7.21E-63 | 92.65% | 565 |
| L1509 | JT915325.1 | camp-regulated phosphoprotein 19-related protein isoform 1 | 3.30E-47 | 84.00% | 628 |
| L1510 | JT917035.1 | protein kinase g11a-like | 3.52E-108 | 90.25% | 658 |
| L1511 | JT929165.1 | cytochrome P450 89a2-like | 7.88E-86 | 87.65% | 601 |
| L1512 | JT943212.1 | Rac-like GTP-binding protein arac1 | 2.93E-124 | 98.00% | 601 |
| L1513 | JR362918.1 | 60S acidic ribosomal protein P3 | 1.49E-05 | 95.00% | 632 |
| L1514 | JT948151.1 | Ribosomal RNA large subunit methyltransferase h | 6.29E-87 | 82.30% | 694 |
| L1515 | JT935862.1 | aldo-keto reductase family 4 member c9-like | 2.08E-65 | 81.25% | 664 |
| L1516 | JT941308.1 | BTB and MATH domain-containing protein | 7.27E-64 | 88.25% | 600 |
| L1517 | JT944939.1 | alpha-expansin 20 precursor family protein | 9.47E-103 | 90.55% | 650 |
| L1518 | JT956309.1 | peptidyl-prolyl cis-trans isomerase fkbp12 | 1.05E-69 | 94.05% | 722 |
| L1519 | JT918565.1 | zinc finger bed domain-containing protein ricesleeper 2-like | 3.41E-158 | 64.85% | 654 |
| L1520 | JT959901.1 |  |  |  | 158 |
| L1521 | JR365489.1 | superoxide dismutase | 1.88E-99 | 92.60% | 619 |
| L1522 | JT956311.1 | 40S ribosomal protein S14-3 | 5.73E-83 | 99.00% | 678 |
| L1523 | JT927834.1 | DNAj protein homolog 2-like | 1.88E-62 | 82.70% | 572 |
| L1524 | JR348979.1 | V-type proton ATPase subunit f | 1.95E-85 | 96.50% | 603 |
| L1525 | JT915944.1 | multiple C2 and transmembrane domain-containing protein 1-like | 3.27E-131 | 82.45% | 674 |
| L1526 | JT963534.1 | 40S ribosomal protein S27-2 | 2.99E-45 | 97.65% | 197 |
| L1527 | JR360547.1 | sucrose transporter 5 | 1.95E-48 | 86.60% | 402 |
| L1528 | JT965343.1 | hypothetical protein JCGZ_17658 | 1.91E-30 | 69.40% | 603 |
| L1529 | JT936132.1 | mitochondrial GTPase 1-like | 2.29E-80 | 91.90% | 614 |
| L1530 | JT960206.1 | E3 ubiquitin-protein ligase rha2a | 2.27E-69 | 75.75% | 575 |
| L1531 | JT960983.1 | zinc finger family protein | 2.88E-29 | 71.43% | 521 |
| L1532 | JT958963.1 | 60S ribosomal protein L31 | 1.19E-62 | 97.05% | 556 |
| L1533 | JT928961.1 | Inositol-tetrakisphosphate 1-kinase | 9.54E-11 | 66.00% | 513 |
| L1534 | JT924851.1 | f-box family protein | 7.22E-63 | 91.20% | 644 |
| L1535 | JT953795.1 | biogenesis of lysosome-related organelles complex 1 subunit 2 | 1.89E-71 | 91.30% | 534 |
| L1536 | JT955725.1 | deoxycytidylate deaminase | 5.40E-115 | 94.90% | 667 |
| L1537 | JT960678.1 | 40S ribosomal protein S17 | 1.64E-87 | 93.05% | 589 |
| L1538 | JT949893.1 | E3 ubiquitin-protein ligase sinat3-like | 2.66E-25 | 73.50% | 627 |
| L1539 | JT927466.1 | glutamyl-tRNA reductase chloroplastic | 2.19E-56 | 76.40% | 520 |
| L1540 | JT928152.1 | E3 ubiquitin-protein ligase rglg2 | 1.44E-130 | 82.75% | 692 |
| L1541 | JT954330.1 | peptidyl-prolyl cis-trans isomerase fkbp15-1 | 1.81E-78 | 92.75% | 535 |
| L1542 | JT957336.1 | protein with unknown function | 8.15E-11 | 71.00% | 489 |
| L1543 | JT950298.1 | f-box kelch-repeat protein at1g57790-like | 8.90E-106 | 93.65% | 519 |
| L1544 | JT960721.1 | superoxide dismutase | 5.85E-89 | 93.65% | 618 |
| L1545 | JT952027.1 | expansin-a13-like | 7.31E-57 | 93.85% | 610 |
| L1546 | JT930051.1 | snf1-related protein kinase regulatory subunit beta-2-like | 1.57E-11 | 89.64% | 208 |
| L1547 | JT950558.1 | 28 kDa heat- and acid-stable phosphoprotein | 1.06E-60 | 87.10% | 722 |
| L1548 |  | peroxisomal membrane protein 13-like | 6.86E-46 | 86.20% | 579 |
| L1549 | JT943124.1 | groes chaperonin | 1.01E-91 | 89.80% | 561 |
| L1550 | JT928756.1 | S-adenosylmethionine-dependent methyltransferase | 3.42E-146 | 93.65% | 700 |
| L1551 | JT928784.1 | arginase mitochondrial | 4.33E-55 | 96.15% | 662 |
| L1552 | JT955567.1 | b-cell receptor-associated 31-like | 4.49E-65 | 92.70% | 698 |
| L1553 | JR365500.1 | RING-H2 finger protein atl56-like | 5.51E-70 | 76.25% | 624 |
| L1554 | JT946172.1 | 40S ribosomal protein S3-3 | 6.22E-94 | 94.80% | 683 |
| L1555 | JT920549.1 | zinc finger CCCH domain-containing protein 41 | 1.95E-94 | 82.85% | 574 |
| L1556 | JT922364.1 | nuclear factor 1 a-type isoform 2 | 4.28E-39 | 89.95% | 493 |
| L1557 | JT949539.1 | Ribosomal L5e family protein isoform 3 | 5.59E-105 | 95.50% | 598 |
| L1558 | JT914478.1 | mitochondrion | 7.64E-26 | 93.36% | 194 |
| L1559 | JT943244.1 | autophagy-related protein 18a | 2.14E-98 | 90.10% | 671 |
| L1560 | JT966418.1 | s-adenosyl-l-methionine-dependent methyltransferases superfamily protein | 1.51E-21 | 85.55% | 589 |
| L1561 | JT954536.1 | protein aluminum sensitive 3 | 5.40E-116 | 96.90% | 663 |
| L1562 | JR366329.1 | peptide methionine sulfoxide reductase a1-like | 1.62E-106 | 84.65% | 546 |
| L1563 | JT960894.1 | 40S ribosomal protein S5-like | 6.57E-81 | 99.95% | 563 |
| L1564 | JT940395.1 | reticulon-like protein b2 | 1.07E-41 | 90.35% | 508 |
| L1565 | JT928786.1 | UBX domain-containing protein | 1.21E-87 | 80.55% | 673 |
| L1566 | JT925915.1 | acyl-CoA binding protein | 8.67E-74 | 82.30% | 682 |
| L1567 | JR347997.1 | leucine-rich repeat family protein isoform 1 | 4.16E-05 | 77.00% | 586 |
| L1568 | JR363946.1 | hypothetical protein 1 | 1.24E-44 | 69.95% | 617 |
| L1569 | JT946496.1 | proteasome subunit beta type-6-like | 2.61E-97 | 98.40% | 596 |
| L1570 | JT932622.1 | aquaporin 2 | 2.04E-120 | 94.05% | 620 |
| L1571 | JT945409.1 | U-box domain-containing protein 3 | 8.88E-109 | 90.05% | 610 |
| L1572 | JT959361.1 | myosin-9 isoform x2 | 3.73E-25 | 85.75% | 668 |
| L1573 | JT949575.1 | protein rdm1 | 1.05E-36 | 90.80% | 591 |
| L1574 | JT930378.1 | probable E3 ubiquitin-protein ligase log2 | 2.28E-90 | 90.60% | 688 |
| L1575 | JT959514.1 | Ribulose-1,5 bisphosphate carboxylase/oxygenase large subunit N-methyltransferase, chloroplast precurso | 1.46E-92 | 78.60% | 596 |
| L1576 | JR366516.1 | isoflavone reductase-like protein | 4.81E-119 | 84.70% | 640 |
| L1577 | JT944921.1 | trigger factor | 3.36E-90 | 86.75% | 562 |
| L1578 | JT916780.1 | hypothetical protein JCGZ_18313 | 4.44E-12 | 68.00% | 551 |
| L1579 | JT960330.1 | epidermal patterning factor-like protein 3 | 5.44E-55 | 70.80% | 676 |
| L1580 | JT957927.1 | bet1-like protein | 6.61E-69 | 89.45% | 638 |
| L1581 | JT952619.1 | Serine arginine repetitive matrix protein | 5.83E-69 | 71.75% | 609 |
| L1582 | JT930280.1 | proteoglycan 4-like isoform x1 | 2.82E-15 | 59.79% | 640 |
| L1583 | JT953889.1 | nucleoside diphosphate kinase 1 | 1.70E-86 | 92.65% | 640 |
| L1584 | JT940047.1 | 2,4-dienoyl-CoA reductase | 1.29E-85 | 92.85% | 513 |
| L1585 | JT948081.1 | glycine-rich RNA-binding protein | 1.74E-49 | 95.50% | 559 |
| L1586 | JR365213.1 |  |  |  | 680 |
| L1587 |  | wat1-related protein at2g39510-like | 3.63E-11 | 83.70% | 605 |
| L1588 |  | protein prd1 | 3.96E-94 | 73.15% | 654 |
| L1589 | JR366748.1 | myosin heavy chain-related family protein | 7.67E-14 | 76.71% | 704 |
| L1590 | JT941333.1 | peptidase pyroglutamyl peptidase i-like isoform 1 | 3.36E-111 | 91.70% | 702 |
| L1591 | JT937801.1 | DNA polymerase epsilon subunit c | 5.22E-35 | 82.60% | 699 |
| L1592 | JT959698.1 | protein binding protein | 3.99E-49 | 83.85% | 484 |
| L1593 | JT931483.1 | UDP-d-xylose:l-fucose alpha- -d-xylosyltransferase 2-like | 1.45E-18 | 87.50% | 307 |
| L1594 | JT941107.1 | probable histone H2B | 2.10E-59 | 100.00% | 652 |
| L1595 | JT926802.1 | leukocyte receptor cluster member 8 homolog | 1.64E-59 | 74.75% | 472 |
| L1596 | JR350036.1 | eukaryotic translation initiation factor 2c | 2.79E-85 | 88.25% | 623 |
| L1597 | JT916354.1 | 60S ribosomal protein L37-3 | 1.14E-56 | 95.60% | 630 |
| L1598 | JT924716.1 | protein phosphatase 2a, regulatory subunit | 7.66E-09 | 76.50% | 506 |
| L1599 | JT946986.1 | signal peptide peptidase-like 2 isoform 1 | 1.77E-16 | 83.15% | 597 |
| L1600 | JT955719.1 | mitochondrial thiamine pyrophosphate carrier | 1.34E-92 | 91.80% | 571 |
| L1601 | JT961529.1 | 40S ribosomal protein S15a-1 | 1.19E-85 | 98.70% | 580 |
| L1602 | JT961462.1 | unnamed protein product | 4.05E-06 | 48.00% | 605 |
| L1603 | JT946402.1 | senescence-associated family protein | 2.20E-20 | 81.75% | 565 |
| L1604 | JT959307.1 | conserved hypothetical protein | 7.15E-32 | 92.30% | 601 |
| L1605 | JT962255.1 | 60S ribosomal protein L27a-3-like | 3.67E-69 | 97.40% | 511 |
| L1606 | JT918273.1 | cyclopropane-fatty-acyl-phospholipid synthase isoform 1 | 1.93E-27 | 92.70% | 588 |
| L1607 | JT940720.1 | endonuclease or glycosyl hydrolase | 1.26E-73 | 80.05% | 578 |
| L1608 | JT925349.1 | 1-phosphatidylinositol-4,5-bisphosphate phosphodiesterase | 6.67E-82 | 80.70% | 543 |
| L1609 | JT930913.1 | somatic embryogenesis receptor kinase 2 | 2.38E-20 | 82.25% | 547 |
| L1610 | JT950009.1 | Populus trichocarpa clone JGIACSB265-L05* | 3.00E-53 | 86.00% | 467 |
| L1611 | JR364139.1 | zinc finger protein 6-like | 2.88E-29 | 71.50% | 648 |
| L1612 |  | protein IQ-DOMAIN 31-like | 7.86E-23 | 71.15% | 557 |
| L1613 | JT952148.1 | tRNA (cytosine-5-)-methyltransferase | 1.08E-21 | 84.25% | 164 |
| L1614 | JR365210.1 | 60S ribosomal protein L23a-like | 2.35E-69 | 95.75% | 628 |
| L1615 | JT971967.1 | RING/FYVE/PHD zinc finger superfamily protein | 6.51E-30 | 87.55% | 544 |
| L1616 | JT918449.1 | ethylene receptor | 2.49E-41 | 80.15% | 388 |
| L1617 | JT914355.1 | pollen-specific protein c13-like | 2.90E-80 | 84.25% | 631 |
| L1618 | JT915168.1 | 5-oxoprolinase | 7.11E-45 | 95.05% | 509 |
| L1619 | JT970482.1 | kinase family protein | 1.29E-29 | 65.25% | 559 |
| L1620 | JT953498.1 | pre-rRNA-processing protein TSR2 homolog | 4.50E-88 | 74.60% | 686 |
| L1621 | JT927975.1 | tubby-like f-box protein 8 | 7.52E-152 | 96.60% | 650 |
| L1622 | JT929760.1 | DNA double-strand break repair rad50 ATPase | 2.53E-82 | 77.65% | 612 |
| L1623 |  | chaperone protein | 2.02E-15 | 74.70% | 689 |
| L1624 | JT944652.1 | quinone oxidoreductase pig3 | 6.87E-69 | 86.70% | 568 |
| L1625 | JT939377.1 | Serine threonine-protein phosphatase pp2a-2 catalytic subunit | 1.33E-25 | 98.20% | 604 |
| L1626 | JT918642.1 | Serine acetyltransferase | 2.86E-16 | 78.10% | 648 |
| L1627 | JT942961.1 | 60S ribosomal protein L9 | 1.44E-107 | 96.50% | 630 |
| L1628 | JR365469.1 | sel1-like repeat-containing protein l18 | 6.72E-69 | 90.05% | 565 |
| L1629 | JR348799.1 | pentatricopeptide repeat-containing protein chloroplastic | 2.59E-99 | 92.45% | 519 |
| L1630 | JT952515.1 | Ubiquitin-conjugating enzyme e2 36 | 7.47E-109 | 99.70% | 547 |
| L1631 | JR348922.1 | WD repeat-containing protein 61 | 3.07E-104 | 94.45% | 579 |
| L1632 | JT959255.1 | tropomyosin-like isoform x1 | 5.19E-65 | 93.35% | 641 |
| L1633 | JT929119.1 | probable protein phosphatase 2c 10-like | 2.32E-42 | 90.25% | 614 |
| L1634 |  | isoflavone reductase family protein | 3.00E-22 | 55.45% | 645 |
| L1635 |  | conserved hypothetical protein* | 1.00E-27 | 79.00% | 590 |
| L1636 | JT960180.1 | pre-mRNA-processing factor 6-like | 1.70E-75 | 75.30% | 668 |
| L1637 | JR355349.1 | casp-like protein 5b2 | 3.87E-17 | 85.70% | 663 |
| L1638 | JT939419.1 | protein sensitivity to red light reduced 1 | 2.07E-41 | 76.30% | 614 |
| L1639 | JR347940.1 | f-box and leucine rich repeat domains containing isoform partial | 5.82E-15 | 84.60% | 547 |
| L1640 | JT924118.1 | Ubiquitin system component cue isoform 1 | 1.04E-105 | 75.50% | 611 |
| L1641 | JT960541.1 | type 1 phosphatases regulator ypi2-like | 1.30E-26 | 68.05% | 692 |
| L1642 | JT919296.1 | dead-box ATP-dependent RNA helicase chloroplastic | 6.95E-13 | 86.70% | 432 |
| L1643 | JT944734.1 | hypothetical protein JCGZ_04721 | 7.00E-49 | 86.95% | 619 |
| L1644 | JT951349.1 | general transcription factor 3c polypeptide 6 | 2.71E-54 | 83.15% | 531 |
| L1645 | JT925274.1 | UDP-D-glucuronate 4-epimerase 6 | 7.61E-38 | 92.70% | 614 |
| L1646 | JT920301.1 | glutaredoxin | 5.65E-72 | 88.45% | 519 |
| L1647 | JT934970.1 | hypothetical protein JCGZ_15532 | 3.53E-16 | 80.50% | 612 |
| L1648 | JT960597.1 | acyl carrier protein | 9.91E-59 | 79.50% | 615 |
| L1649 | JT922390.1 | hypothetical protein JCGZ_12385 | 1.46E-15 | 81.70% | 367 |
| L1650 | JT958631.1 | peroxiredoxin family protein | 2.40E-108 | 93.85% | 673 |
| L1651 | JR365291.1 | profilin 1 isoform 1 | 1.68E-77 | 96.00% | 680 |
| L1652 | JT928233.1 | transducin WD40 repeat-like superfamily protein isoform 2 | 5.36E-105 | 90.75% | 620 |
| L1653 | JR348750.1 | uncharacterized loc101221008 | 7.58E-26 | 71.00% | 618 |
| L1654 | JT947142.1 | conserved hypothetical protein | 2.05E-70 | 86.95% | 494 |
| L1655 | JT973830.1 |  |  |  | 544 |
| L1656 | JT956981.1 | probable 26S proteasome complex subunit sem1-1 | 1.04E-16 | 91.35% | 492 |
| L1657 | JR364476.1 | HSP1 family protein | 3.10E-31 | 86.20% | 599 |
| L1658 | JT921487.1 | Ubiquitin-protein ligase | 2.25E-17 | 79.30% | 610 |
| L1659 | JR366445.1 | polyubiquitin | 8.15E+00 | 99.40% | 617 |
| L1660 | JR365666.1 |  |  |  | 648 |
| L1661 | JT931359.1 | hypothetical protein JCGZ_18356 | 1.22E-48 | 92.55% | 552 |
| L1662 | JT957923.1 | 60S ribosomal protein L30 | 1.65E-64 | 98.65% | 605 |
| L1663 | JR365609.1 | DNA-directed RNA polymerase II subunit rpb7 | 9.17E-103 | 98.70% | 534 |
| L1664 | JT952939.1 | peptidyl-prolyl cis-trans isomerase cyp18-1 | 4.70E-111 | 96.30% | 654 |
| L1665 | JT915984.1 | interferon-induced guanylate-binding protein 2 | 3.51E-24 | 85.20% | 662 |
| L1666 | JT961394.1 | hypothetical protein POPTR_0004s16220g | 1.15E-34 | 79.25% | 561 |
| L1667 | JT916136.1 | alpha-mannosidase 2x | 7.11E-86 | 85.90% | 627 |
| L1668 | JT919378.1 | hydrolase family protein | 9.64E-13 | 82.76% | 502 |
| L1669 | JT952413.1 | hypothetical protein JCGZ_21466 | 3.35E-18 | 61.60% | 504 |
| L1670 | JT967873.1 | phosphosulfolactate synthase | 3.36E-32 | 54.60% | 593 |
| L1671 | JT958161.1 | 60S ribosomal protein L34-like | 3.00E-48 | 97.90% | 427 |
| L1672 | JT951796.1 | membrane-anchored ubiquitin-fold protein 3 | 3.06E-51 | 87.30% | 500 |
| L1673 | JT961480.1 | Coiled-coil domain-containing protein* | 6.00E-95 | 78.00% | 525 |
| L1674 | JT964509.1 | 2,3-bisphosphoglycerate-dependent phosphoglycerate mutase | 4.40E-44 | 81.40% | 488 |
| L1675 | JT948896.1 | protein lhcp translocation defect-like | 2.16E-23 | 94.40% | 203 |
| L1676 | JT922385.1 | adenylosuccinate lyase | 9.98E-18 | 87.50% | 597 |
| L1677 | JT962444.1 | conserved hypothetical protein* | 2.00E-15 | 70.00% | 274 |
| L1678 | JT951441.1 | 18.2 kDa class I heat shock protein | 2.68E-91 | 89.35% | 365 |
| L1679 | JT933986.1 | geranylgeranyl-diphosphate synthase | 1.04E-111 | 81.00% | 583 |
| L1680 | JT948897.1 | histone H1 | 1.20E-18 | 85.15% | 368 |
| L1681 | JT952292.1 | Vacuolar protein sorting-associated protein 29-like | 3.22E-80 | 96.30% | 532 |
| L1682 | JT916967.1 | K+ uptake permease 11 isoform 1 | 6.10E-21 | 92.50% | 402 |
| L1683 | JT926766.1 | adenylate kinase 1 | 5.36E-59 | 94.90% | 338 |
| L1684 | JT934165.1 | probable gpi-anchored adhesin-like protein pga55 isoform x2 | 1.11E-41 | 51.85% | 468 |
| L1685 | JT941165.1 | cycloartenol-C-24-methyltransferase | 3.22E-34 | 93.75% | 452 |
| L1686 | JT945833.1 | GTP-binding protein sar1a-like | 1.06E-82 | 98.50% | 360 |
| L1687 | JT960070.1 | coiled-coil domain-containing protein 124-like | 1.14E-21 | 92.05% | 508 |
| L1688 | JT914546.1 | bah helical bundle-like domain isoform 1 | 3.69E-56 | 75.45% | 393 |
| L1689 | JT950055.1 | DNA-3-methyladenine glycosylase | 8.66E-143 | 89.60% | 290 |
| L1690 | JT928886.1 | C2H2-like zinc finger protein | 2.96E-05 | 82.70% | 169 |
| L1691 |  | elongation factor 1-alpha | 1.20E-42 | 87.00% | 257 |
| L1692 | JT944520.1 | rRNA-processing protein efg1 | 3.03E-64 | 93.70% | 478 |
| L1693 | JT925633.1 | RNA binding protein | 6.22E-61 | 81.95% | 455 |
| L1694 | JR345440.1 | bag family molecular chaperone regulator 7-like | 1.85E-14 | 77.25% | 148 |
| L1695 | JR361831.1 |  |  |  | 222 |
| L1696 | JT924151.1 | interferon-induced GTP-binding protein | 2.71E-73 | 89.00% | 408 |
| L1697 | JT952340.1 | CCP-like protein | 1.55E-10 | 53.80% | 467 |
| L1698 | JT948698.1 | Ubiquitin-like protein | 9.66E-119 | 98.95% | 577 |
| L1699 | JT959436.1 | phosphatidylglycerol/phosphatidylinositol transfer protein DDB_G0282179-like | 5.91E-58 | 76.45% | 535 |
| L1700 | JT958348.1 | transcription elongation factor 1 homolog | 1.14E-34 | 95.80% | 458 |
| L1701 | JT946965.1 | Stem-specific protein TSJT1 | 3.59E-109 | 91.55% | 553 |
| L1702 | JT925666.1 | calcium ion binding protein | 1.03E-59 | 82.40% | 498 |
| L1703 | JT923953.1 | chloroplast stem-loop binding protein of 41 kDa chloroplastic-like | 1.78E-66 | 89.50% | 539 |
| L1704 | JT949923.1 |  |  |  | 452 |
| L1705 | JT954434.1 | GDP-l-galactose phosphorylase 1-like | 3.73E-31 | 77.60% | 411 |
| L1706 | JT961026.1 |  |  |  | 538 |
| L1707 | JT954984.1 | transferase-like superfamily protein isoform 1 | 7.00E-65 | 93.35% | 460 |
| L1708 | JR348736.1 | splicing factor 3a subunit 3-like | 8.30E-14 | 91.60% | 390 |
| L1709 | JT967983.1 | conserved hypothetical protein | 4.62E-51 | 66.75% | 469 |
| L1710 | JR355015.1 | hypothetical protein MTR_3g064100 | 2.95E-08 | 73.00% | 131 |
| L1711 | JT936082.1 | DNAj heat shock family protein | 1.50E-57 | 95.10% | 466 |
| L1712 | JT937798.1 | 28 kDa ribonucleoprotein, chloroplast like | 2.63E-75 | 63.20% | 587 |
| L1713 | JT919244.1 | eukaryotic translation initiation factor 3 subunit b-like | 2.15E-78 | 89.10% | 581 |
| L1714 | JR363592.1 | histone H4 | 8.32E-49 | 99.70% | 269 |
| L1715 | JT952046.1 | nucleobase-ascorbate transporter 3-like | 4.20E-48 | 83.65% | 458 |
| L1716 | JT974820.1 | hypothetical protein JCGZ_06201 | 3.19E-08 | 63.00% | 450 |
| L1717 | JT955783.1 | hth-type transcriptional regulator protein ptxe | 8.05E-66 | 88.40% | 544 |
| L1718 | JT944093.1 | probable 2-aminoethanethiol dioxygenase | 6.84E-81 | 93.00% | 368 |
| L1719 | JT923592.1 | NEDD8 ultimate buster 1-like | 8.21E-98 | 90.20% | 628 |
| L1720 | JT961018.1 | histone deacetylase 6 | 1.03E-82 | 87.15% | 636 |
| L1721 |  | probable carboxylesterase 2 | 2.83E-83 | 76.85% | 532 |
| L1722 | JT924312.1 | protein early flowering 4-like | 6.03E-38 | 80.80% | 544 |
| L1723 | JR365970.1 |  |  |  | 490 |
| L1724 | JT946323.1 | histone H2A | 7.94E-37 | 98.00% | 470 |
| L1725 | JT938330.1 | fasciclin-like arabinogalactan protein 4 | 2.70E-65 | 77.70% | 653 |
| L1726 | JR348879.1 | BTB/POZ domain-containing family protein | 1.86E-61 | 60.65% | 613 |
| L1727 | JT953249.1 | tom1-like protein 2 | 4.75E-34 | 73.95% | 508 |
| L1728 | JT964226.1 | DNA binding protein | 2.84E-12 | 72.00% | 605 |
| L1729 | JT930360.1 | mitochondrial glycoprotein | 1.68E-68 | 78.25% | 414 |
| L1730 | JT976028.1 |  |  |  | 516 |
| L1731 | JT953456.1 | protein yippee-like at4g27745 | 1.62E-62 | 89.15% | 649 |
| L1732 | JT934213.1 | beta-ureidopropionase | 6.56E-66 | 93.90% | 555 |
| L1733 | JT973265.1 |  |  |  | 680 |
| L1734 | JR358932.1 | amidase 1 | 4.42E-17 | 94.40% | 361 |
| L1735 | JT928679.1 | binding isoform 1 | 3.55E-150 | 88.85% | 492 |
| L1736 | JT939515.1 | protein BPS1, chloroplastic-like | 3.54E-88 | 70.65% | 525 |
| L1737 | JR363554.1 | cytochrome b-c1 complex subunit 8 | 2.97E-41 | 93.90% | 383 |
| L1738 | JT947113.1 | conserved hypothetical protein | 4.38E-74 | 79.20% | 620 |
| L1739 | JT963491.1 | E3 ubiquitin-protein ligase rha1b-like | 7.29E-105 | 79.55% | 551 |
| L1740 | JT965229.1 | NADH dehydrogenase | 3.93E-31 | 89.15% | 546 |
| L1741 | JT928360.1 | homeodomain-like superfamily protein isoform 1 | 8.34E-16 | 92.85% | 352 |
| L1742 | JT949435.1 | plastid transcriptionally active7 isoform 1 | 7.75E-71 | 81.15% | 590 |
| L1743 | JT962925.1 |  |  |  | 464 |
| L1744 | JT943129.1 | proteasome subunit beta type-3-a | 2.99E-123 | 98.20% | 535 |
| L1745 | JT945052.1 |  |  |  | 377 |
| L1746 | JR362212.1 | glucose-induced degradation protein 8 homolog | 1.20E-31 | 93.45% | 399 |
| L1747 | JT927992.1 | 26S protease regulatory subunit s10b b -like protein | 1.67E-103 | 97.95% | 533 |
| L1748 | JT961245.1 | protein-tyrosine-phosphatase ibr5 | 7.89E-43 | 92.45% | 481 |
| L1749 | JT938319.1 | C3HC4-type RING zinc finger protein (RGZF1) | 6.94E-70 | 73.50% | 669 |
| L1750 | JT947196.1 | eukaryotic translation initiation factor 1a | 2.48E-64 | 99.90% | 697 |
| L1751 | JT980971.1 |  |  |  | 129 |
| L1752 | JT940355.1 | triosphosphate isomerase-like protein type I | 1.12E-67 | 93.90% | 340 |
| L1753 | JT941466.1 | nucleotide-diphospho-sugar transferases superfamily protein isoform 1 | 6.24E-81 | 97.10% | 571 |
| L1754 | JT962773.1 | cytochrome c oxidase subunit mitochondrial-like | 8.64E-46 | 80.45% | 564 |
| L1755 | JT961513.1 | senescence-associated family protein | 7.56E-48 | 91.10% | 503 |
| L1756 | JT950231.1 | hypothetical protein POPTR_0002s08440g | 3.59E-38 | 70.25% | 566 |
| L1757 | JT927456.1 | E3 ubiquitin-protein ligase rhf1a-like | 1.39E-51 | 62.90% | 671 |
| L1758 | JT965960.1 |  |  |  | 647 |
| L1759 | JR364962.1 | V-type proton ATPase subunit h-like | 1.39E-56 | 93.45% | 477 |
| L1760 | JT946147.1 | oligouridylate-binding protein 1-like isoform x1 | 3.79E-16 | 72.70% | 410 |
| L1761 | JR362873.1 | LOB domain-containing protein 41-like | 5.95E-112 | 85.80% | 621 |
| L1762 | JT925597.1 |  |  |  | 653 |
| L1763 | JT934383.1 | glycerol-3-phosphate acyltransferase 8 | 6.38E-89 | 93.60% | 556 |
| L1764 | JT979891.1 | mitochondrial import inner membrane translocase subunit Tim17* | 1.00E-11 | 68.00% | 304 |
| L1765 | JT965428.1 | triacylglycerol lipase | 1.46E-86 | 86.60% | 536 |
| L1766 | JT933460.1 | nicotinamide adenine dinucleotide transporter chloroplastic | 1.30E-56 | 90.50% | 415 |
| L1767 | JT971478.1 | solanesyl diphosphate synthase 3 | 1.16E-58 | 73.20% | 694 |
| L1768 | JT945155.1 | hypothetical protein JCGZ_25276 | 5.03E-06 | 69.33% | 455 |
| L1769 | JR366316.1 | mRNA-decapping enzyme-like protein | 1.04E-23 | 73.20% | 497 |
| L1770 | JT927053.1 | calmodulin-binding receptor-like cytoplasmic kinase 3 | 9.65E-134 | 92.85% | 606 |
| L1771 | JT937198.1 | pyruvate cytosolic isozyme-like | 2.15E-65 | 85.25% | 613 |
| L1772 | JR363580.1 |  |  |  | 418 |
| L1773 | JT935432.1 | E3 ubiquitin-protein ligase sinat3-like | 1.04E-13 | 90.60% | 410 |
| L1774 | JR350891.1 | polyphenol oxidase chloroplastic | 4.79E-11 | 73.33% | 244 |
| L1775 | JT942366.1 | polyubiquitin | 2.70E-68 | 98.10% | 338 |
| L1776 | JR363860.1 | branched-chain amino acid aminotransferase | 3.29E-22 | 81.40% | 309 |
| L1777 | JR345857.1 | NADH dehydrogenase | 1.40E-65 | 91.50% | 617 |
| L1778 | JT934899.1 | rRNA-processing protein efg1-like isoform x1 | 1.03E-127 | 90.50% | 388 |
| L1779 | JT954179.1 | Ubiquitin-conjugating enzyme e2-23 kDa | 1.93E-58 | 92.05% | 568 |
| L1780 | JT946303.1 | BRI1-KD interacting protein | 1.07E-35 | 63.50% | 569 |
| L1781 | JT935582.1 | 26S proteasome non-ATPase regulatory subunit 6 homolog | 2.20E-41 | 84.90% | 337 |
| L1782 | JT957419.1 | HVA22-like protein e | 2.33E-50 | 81.65% | 498 |
| L1783 | JT969215.1 | conserved hypothetical protein | 1.01E-26 | 66.10% | 495 |
| L1784 | JT932317.1 | f-box protein at3g07870 | 1.11E-78 | 73.25% | 621 |
| L1785 | JR345685.1 | protein decapping 5 isoform x1 | 2.57E-23 | 90.35% | 632 |
| L1786 | JT927585.1 | V-type proton ATPase subunit b2 | 1.06E-47 | 99.40% | 570 |
| L1787 | JT938133.1 | 30S ribosomal protein S5 | 1.86E-23 | 76.90% | 534 |
| L1788 | JT914199.1 | E3 ubiquitin-protein ligase upl2-like isoform x1 | 4.20E-86 | 99.00% | 633 |
| L1789 | JT946132.1 | probable bifunctional methylthioribulose-1-phosphate dehydratase enolase-phosphatase partial | 4.84E-52 | 92.65% | 686 |
| L1790 | JR347915.1 | otu domain-containing protein at3g57810-like | 1.13E-57 | 66.45% | 538 |
| L1791 | JT956913.1 | Ubiquitin-conjugating enzyme e2 2 | 4.90E-73 | 98.20% | 353 |
| L1792 | JT927002.1 | phosphate transporter | 4.33E-108 | 97.75% | 587 |
| L1793 | JT938081.1 | probable inactive shikimate kinase like chloroplastic | 7.71E-91 | 75.65% | 598 |
| L1794 | JT930767.1 | endoglucanase 6-like | 5.07E-20 | 90.45% | 500 |
| L1795 | JT926079.1 | homeobox protein knotted-1-like 1 | 2.26E-34 | 65.05% | 555 |
| L1796 | JT943716.1 | transmembrane protein 115-like | 9.92E-63 | 91.30% | 494 |
| L1797 | JR365198.1 | RNA binding protein | 2.99E-13 | 75.00% | 452 |
| L1798 | JT934326.1 | metal tolerance protein 11-like | 2.59E-23 | 82.15% | 437 |
| L1799 | JT948771.1 | PREDICTED: uncharacterized protein LOC102612426 | 1.20E-10 | 59.00% | 536 |
| L1800 | JT960267.1 | 60S ribosomal protein L26-1-like | 1.41E-75 | 98.50% | 534 |
| L1801 | JT942878.1 | GTP-binding nuclear protein Ran-3 | 5.92E-53 | 96.55% | 630 |
| L1802 | JR348692.1 | molybdopterin cofactor sulfurase | 6.20E-67 | 88.80% | 545 |
| L1803 | JT956836.1 |  |  |  | 444 |
| L1804 | JT937004.1 | pre-mRNA-processing factor 39 | 3.15E-114 | 84.00% | 646 |
| L1805 | JT970556.1 |  |  |  | 521 |
| L1806 | JR366621.1 | CAX-interacting protein 4 | 2.33E-70 | 92.65% | 647 |
| L1807 | JR365176.1 | reticulon-like protein b22 | 5.33E-80 | 89.90% | 646 |
| L1808 | JT924535.1 | trafficking protein particle complex subunit 6b | 1.27E-117 | 92.05% | 647 |
| L1809 | JT941974.1 | formin-like protein 18 | 6.38E-133 | 90.90% | 685 |
| L1810 | JR366157.1 | probable phospholipid hydroperoxide glutathione peroxidase | 8.28E-128 | 88.45% | 635 |
| L1811 | JT945521.1 | zinc finger A20 and AN1 domain-containing stress-associated protein 5 | 1.40E-52 | 66.85% | 556 |
| L1812 | JT914739.1 | heat shock transcription factor a1d isoform 1 | 1.02E-40 | 97.05% | 527 |
| L1813 | JT945287.1 | 40S ribosomal protein S3a | 1.30E-123 | 97.30% | 640 |
| L1814 | JT928389.1 | sterol 14-demethylase-like | 7.31E-44 | 90.20% | 436 |
| L1815 | JR360706.1 | C2 domain-containing family protein | 1.65E-07 | 69.00% | 669 |
| L1816 | JT968444.1 | Hevea brasiliensis isolate SSH12 mRNA sequence* | 2.00E-104 | 100.00% | 270 |
| L1817 | JT937680.1 | enhancer of rudimentary homolog | 1.02E-41 | 94.90% | 374 |
| L1818 | JT925474.1 | hydroxyproline-rich glycoprotein | 1.09E-80 | 74.95% | 597 |
| L1819 | JT929899.1 | DNA binding protein | 2.40E-137 | 76.70% | 628 |
| L1820 | JT937325.1 | cis-prenyl transferase | 6.99E-31 | 96.55% | 579 |
| L1821 | JT921560.1 | DUF789 family protein | 5.28E-21 | 89.60% | 632 |
| L1822 | JT941886.1 | proteophosphoglycan-related family protein | 8.12E-29 | 69.30% | 647 |
| L1823 | JT950891.1 | 14-3-3-like protein d | 4.37E-16 | 94.30% | 518 |
| L1824 | JT930322.1 | inositol-tetrakisphosphate 1-kinase 1-like | 1.37E-12 | 78.45% | 586 |
| L1825 | JR346721.1 | Aspartic proteinase-like protein 1 isoform x2 | 4.81E-44 | 82.05% | 561 |
| L1826 | JR360998.1 | histone H1 | 2.43E-15 | 97.63% | 461 |
| L1827 | JT915377.1 | non-specific lipid-transfer protein 2-like | 2.49E-28 | 79.35% | 500 |
| L1828 | JT933592.1 | geranylgeranyl pyrophosphate synthase | 2.90E-171 | 87.95% | 350 |
| L1829 | JT926699.1 | V-type proton ATPase subunit b2 | 7.31E-14 | 98.00% | 398 |
| L1830 | JT939950.1 | CAX-interacting protein 4 (LOC105124539), transcript variant X1* | 4.00E-109 | 82.00% | 480 |
| L1831 | JT931867.1 | SOUL heme-binding family protein | 1.86E-27 | 89.95% | 546 |
| L1832 | JT950683.1 | KH domain-containing protein isoform 1 | 9.16E-63 | 74.80% | 550 |
| L1833 | JT915295.1 | cellulose synthase a catalytic subunit 2 | 1.58E-14 | 93.30% | 267 |
| L1834 | JT915904.1 | disease resistance protein at4g27190-like | 4.71E-24 | 81.80% | 181 |
| L1835 | JT970321.1 | proteinase inhibitor | 7.20E-11 | 58.00% | 485 |
| L1836 | JT954435.1 | type II cytoskeletal 2 epidermal-like isoform x1 | 6.21E-08 | 80.63% | 558 |
| L1837 | JT949404.1 | zinc finger family protein | 7.66E-91 | 75.75% | 557 |
| L1838 | JT961632.1 | disease resistance protein RGA3 | 6.54E-15 | 60.00% | 399 |
| L1839 | JR345942.1 | exocyst complex component exo70b1-like | 1.50E-53 | 81.20% | 509 |
| L1840 | JT945366.1 | NADH dehydrogenase | 2.44E-93 | 95.30% | 641 |
| L1841 | JT915537.1 | ATP binding protein | 5.23E-33 | 74.35% | 607 |
| L1842 | JT961105.1 | cytochrome b-c1 complex subunit 7-2-like | 4.43E-51 | 92.75% | 622 |
| L1843 | JT964854.1 | hypothetical protein 3 | 2.54E-45 | 71.90% | 550 |
| L1844 | JT917719.1 | probable glycerophosphoryl diester phosphodiesterase 2 | 2.29E-80 | 76.40% | 163 |
| L1845 | JT915709.1 | structural constituent of nuclear pore, putative | 8.38E-28 | 67.90% | 502 |
| L1846 | JT940004.1 | agamous-like MADS-box protein agl19 | 1.04E-52 | 90.20% | 533 |
| L1847 | JR358058.1 | glycine-rich family protein | 1.68E-32 | 90.30% | 358 |
| L1848 | JT946641.1 | NADH dehydrogenase | 8.41E-69 | 94.80% | 587 |
| L1849 | JR361274.1 | hypothetical protein JCGZ_16275 | 6.70E-29 | 75.10% | 545 |
| L1850 | JR365736.1 | isocitrate dehydrogenase | 4.30E-106 | 91.15% | 603 |
| L1851 | JT948379.1 | 50S ribosomal protein L32pa | 6.70E-88 | 74.75% | 520 |
| L1852 | JT953856.1 | 50S ribosomal protein L14 | 1.42E-50 | 96.75% | 608 |
| L1853 | JT953751.1 | SAUR family protein (SAUR23) | 4.04E-45 | 92.35% | 610 |
| L1854 | JT918040.1 | rela spot homolog 3 family protein | 4.64E-16 | 84.80% | 511 |
| L1855 | JR345303.1 | 40S ribosomal protein S21-2-like | 1.60E-54 | 95.45% | 479 |
| L1856 | JT954815.1 | protein shoot gravitropism 5-like | 1.03E-50 | 85.70% | 602 |
| L1857 | JT978166.1 |  |  |  | 516 |
| L1858 | JT944781.1 | probable histone 2 | 3.28E-17 | 98.20% | 240 |
| L1859 | JT973578.1 | uncharacterized loc101204080 | 1.12E-07 | 75.58% | 307 |
| L1860 | JT939867.1 | adenosine 3' -phospho 5' -phosphosulfate transporter | 2.10E-82 | 96.05% | 580 |
| L1861 | JT943118.1 | exosome complex component rrp4 | 8.17E-89 | 80.20% | 631 |
| L1862 | JT929680.1 | kinase family protein | 3.22E-101 | 84.65% | 519 |
| L1863 | JT936232.1 | 8-oxoguanine DNA glycosylase | 4.83E-90 | 87.50% | 607 |
| L1864 | JT945667.1 | la-related protein 7 | 5.67E-46 | 92.05% | 499 |
| L1865 | JT923738.1 | heterogeneous nuclear ribonucleoprotein 1 | 6.91E-133 | 84.95% | 552 |
| L1866 | JR354332.1 | domain-containing receptor-like kinase | 7.62E-17 | 67.15% | 305 |
| L1867 | JT971928.1 | DNAj heat shock n-terminal domain-containing isoform 1 | 8.09E-69 | 72.20% | 608 |
| L1868 | JT942783.1 | short chain dehydrogenase | 1.25E-46 | 84.20% | 535 |
| L1869 | JT964392.1 | 10 kDa chaperonin-like | 1.35E-51 | 91.25% | 523 |
| L1870 | JT970626.1 | hypothetical protein 33 | 7.99E-61 | 89.95% | 558 |
| L1871 | JT936365.1 | scarecrow-like protein 4 | 3.21E-83 | 75.85% | 597 |
| L1872 | JT934048.1 | omega-6 fatty acid desaturase (FAD) | 1.19E-131 | 92.30% | 666 |
| L1873 | JT936885.1 | zinc finger protein chloroplastic | 4.78E-11 | 74.10% | 457 |
| L1874 | JR365859.1 | histone H2A | 8.41E-45 | 97.80% | 579 |
| L1875 | JT955041.1 | cytochrome b5 isoform Cb5-C | 4.27E-89 | 93.15% | 638 |
| L1876 | JT930883.1 | zinc finger CCHC-type and RNA-binding motif-containing protein 1 | 3.02E-06 | 86.50% | 524 |
| L1877 | JT925187.1 | 3-ketoacyl- synthase 4 | 7.63E-78 | 87.35% | 536 |
| L1878 | JT945391.1 | broad-range acid phosphatase det1 | 4.15E-48 | 80.00% | 687 |
| L1879 |  | diacylglycerol kinase family protein | 1.21E-42 | 69.95% | 436 |
| L1880 | JT960422.1 | hypothetical protein (POPTR_0012s07910g)* | 3.00E-78 | 78.00% | 420 |
| L1881 | JT922340.1 | jacalin-related lectin 4-like | 7.89E-11 | 61.50% | 427 |
| L1882 | JT963271.1 | 60S ribosomal protein L37a | 1.25E-59 | 97.40% | 486 |
| L1883 | JT947754.1 | toc64 family protein | 3.64E-64 | 88.70% | 508 |
| L1884 | JT968698.1 | 60S ribosomal protein L29-1 | 7.43E-35 | 96.80% | 458 |
| L1885 | JT973651.1 | nucleolar protein 58-like | 6.36E-54 | 82.60% | 342 |
| L1886 | JT919387.1 | f-box family isoform 1 | 2.78E-62 | 70.85% | 451 |
| L1887 | JT929060.1 | 3-hydroxy-3-methylglutaryl coenzyme A synthase | 8.33E-66 | 91.35% | 474 |
| L1888 | JT939607.1 | magnesium transporter mrs2-i-like | 1.75E-43 | 85.75% | 546 |
| L1889 | JR346352.1 | Serine threonine-protein phosphatase pp2a catalytic subunit | 5.91E-89 | 98.15% | 650 |
| L1890 | JT933643.1 | hypothetical protein JCGZ_17861 | 2.33E-69 | 75.65% | 480 |
| L1891 | JR366181.1 | 60S acidic ribosomal protein P0 | 2.26E-36 | 96.60% | 544 |
| L1892 | JR349288.1 | kinase interacting (kip1-like) family | 3.69E-23 | 80.10% | 483 |
| L1893 | JT917289.1 | transcription factor GTE8 isoform x1 | 3.74E-31 | 84.75% | 285 |
| L1894 | JR345896.1 | hypothetical protein 20 | 1.30E-09 | 86.33% | 353 |
| L1895 | JT914263.1 | calpain-type cysteine protease dek1 | 4.80E-16 | 92.45% | 478 |
| L1896 | JT932555.1 | plant intracellular ras-group-related lrr protein 9-like | 2.89E-35 | 72.45% | 443 |
| L1897 | JT937849.1 | 3-methyl-2-oxobutanoate hydroxymethyltransferase | 3.37E-11 | 61.00% | 541 |
| L1898 | JT946043.1 | WD repeat-containing protein 5-like | 3.61E-43 | 93.30% | 453 |
| L1899 | JT959757.1 | centromere protein v-like | 2.41E-41 | 88.75% | 581 |
| L1900 | JR345381.1 | kinesin light chain | 6.49E-94 | 93.85% | 616 |
| L1901 | JR364493.1 | NAC domain-containing protein 2 | 1.47E-62 | 70.20% | 462 |
| L1902 | JT929140.1 | E3 ubiquitin-protein ligase upl7 | 8.25E-40 | 94.35% | 335 |
| L1903 | JT942967.1 | tyrosine decarboxylase 1-like | 4.18E-100 | 93.40% | 658 |
| L1904 | JR366884.1 | adenylosuccinate synthetase | 7.08E-112 | 84.15% | 613 |
| L1905 | JT936965.1 | E3 ubiquitin-protein ligase at1g63170-like | 1.25E-61 | 82.25% | 642 |
| L1906 | JT966408.1 | protein RALF-like 24 | 4.15E-56 | 82.25% | 619 |
| L1907 | JT951023.1 | RING-box protein 1a-like | 1.15E-48 | 100.00% | 567 |
| L1908 | JT915654.1 | t-complex protein 11-like protein 1 | 6.06E-53 | 78.65% | 568 |
| L1909 | JT919017.1 | mannan endo-1, 4-beta-mannosidase 7-like | 7.20E-63 | 84.45% | 533 |
| L1910 | JT928847.1 | polyadenylate-binding protein 2-like | 2.45E-12 | 93.10% | 166 |
| L1911 | JR344767.1 | ETHYLENE-INSENSITIVE3 protein | 2.00E-47 | 91.05% | 426 |
| L1912 | JT950758.1 | hypothetical protein JCGZ_11253 | 1.08E-53 | 75.45% | 695 |
| L1913 | JT951847.1 | mitochondrial import inner membrane translocase subunit Tim14-3-like | 3.65E-22 | 97.05% | 538 |
| L1914 | JT958801.1 | NUC-1 negative regulatory protein | 2.42E-81 | 92.50% | 600 |
| L1915 | JT934645.1 | ABC transporter I family member 19 | 4.97E-67 | 91.00% | 455 |
| L1916 | JT938245.1 | soluble diacylglycerol acyltransferase | 5.70E-40 | 60.00% | 537 |
| L1917 | JT938111.1 | nitric oxide synthase-interacting protein | 6.85E-42 | 98.75% | 372 |
| L1918 | JT937667.1 | no apical meristem family protein | 2.24E-20 | 80.90% | 479 |
| L1919 | JT929971.1 | 3-hydroxy-4-methylglutaryl coenzyme A reductase | 6.61E-19 | 97.40% | 484 |
| L1920 | JR361680.1 | probable pyridoxal biosynthesis protein pdx1 | 1.63E-48 | 97.05% | 337 |
| L1921 | JT945335.1 | 18.2 kDa class I heat shock family protein | 8.46E-62 | 87.75% | 492 |
| L1922 | JT951658.1 | calmodulin (CaM)* | 8.00E-80 | 76.00% | 475 |
| L1923 | JT922007.1 | mannan endo-1, 4-beta-mannosidase 2-like | 9.59E-14 | 72.85% | 448 |
| L1924 | JT956911.1 | protein Asterix | 1.63E-43 | 93.10% | 498 |
| L1925 | JT944694.1 | plasma membrane intrinsic protein isoform 1 | 6.94E-111 | 94.35% | 567 |
| L1926 |  | cationic peroxidase 1-like | 1.22E-44 | 82.80% | 624 |
| L1927 | JT917106.1 | f-box LRR-repeat protein 15-like | 1.25E-12 | 89.60% | 309 |
| L1928 | JR365346.1 | histone H3 | 6.06E-93 | 98.85% | 619 |
| L1929 | JT955482.1 | probable calcium-binding protein cml13 | 5.57E-97 | 95.65% | 516 |
| L1930 |  | E3 ubiquitin-protein ligase ring1-like | 3.23E-14 | 75.60% | 211 |
| L1931 | JT947009.1 | Ras-related protein Rabe1a-like | 5.10E-54 | 97.00% | 598 |
| L1932 | JT959300.1 | oligoribonuclease, mitochondrial | 5.93E-74 | 74.80% | 477 |
| L1933 | JT952522.1 | trafficking protein particle complex subunit 4-like | 4.95E-97 | 95.65% | 602 |
| L1934 |  | 17.5 kDa class II heat shock protein | 3.23E-39 | 74.40% | 501 |
| L1935 | JT961738.1 | acyl-coenzyme a thioesterase 13-like | 1.85E-87 | 89.30% | 606 |
| L1936 | JT960245.1 | cyclin-b1-2-like | 1.07E-71 | 89.55% | 528 |
| L1937 | JT954922.1 | microsomal glutathione s-transferase 3-like | 1.13E-80 | 89.75% | 552 |
| L1938 | JR364328.1 | s-adenosyl-l-homocysteine hydrolase | 2.62E-96 | 98.20% | 483 |
| L1939 | JT938780.1 | E3 ubiquitin-protein ligase cip8-like | 2.94E-72 | 55.55% | 220 |
| L1940 | JR365847.1 | 14 kDa zinc-binding protein | 7.23E-85 | 88.70% | 633 |
| L1941 | JT964622.1 | mitochondrial import receptor subunit TOM6 homolog | 6.79E-27 | 89.05% | 599 |
| L1942 | JT947390.1 | protein rad52-2 | 1.00E-70 | 91.15% | 611 |
| L1943 | JT916017.1 | nucleic acid binding protein | 6.40E-09 | 85.05% | 261 |
| L1944 | JT916156.1 | isoleucyl tRNA synthetase | 1.21E-14 | 68.78% | 471 |
| L1945 | JT959785.1 | cysteine-rich and transmembrane domain-containing protein A-like (LOC105113396)* | 5.00E-52 | 76.00% | 558 |
| L1946 | JT976119.1 | nudix hydrolase chloroplastic-like | 4.45E-25 | 78.45% | 630 |
| L1947 | JT923232.1 | protein far1-related sequence 9 | 4.20E-142 | 88.80% | 662 |
| L1948 | JT952822.1 | autophagy-related protein 8c-like | 1.82E-63 | 94.75% | 642 |
| L1949 | JT924213.1 | dihydrolipoyllysine-residue acetyltransferase component 2 of pyruvate dehydrogenase mitochondrial-like | 2.12E-86 | 96.50% | 506 |
| L1950 | JT953110.1 | f-box protein gid2-like | 2.55E-20 | 90.60% | 470 |
| L1951 | JT961337.1 | small acidic protein 1 | 6.12E-20 | 88.84% | 564 |
| L1952 | JT947604.1 | localized to the inner membrane of the chloroplast | 1.91E-43 | 91.80% | 537 |
| L1953 |  | anthocyanidin 3-o-glucosyltransferase 5-like | 3.03E-18 | 78.90% | 344 |
| L1954 | JR348866.1 | transcription factor TCP11-like | 4.69E-26 | 58.70% | 493 |
| L1955 |  | fanconi anemia group I protein | 5.26E-23 | 65.70% | 576 |
| L1956 | JT952212.1 | o-fucosyltransferase family protein | 3.73E-17 | 94.10% | 577 |
| L1957 | JT960335.1 | ATP synthase subunit mitochondrial | 9.10E-39 | 99.90% | 480 |
| L1958 | JT945887.1 | translation machinery-associated protein 22-like | 9.71E-91 | 90.85% | 628 |
| L1959 | JT925800.1 | DNA binding protein | 1.04E-18 | 75.20% | 558 |
| L1960 | JT945054.1 | Serine threonine-protein phosphatase pp1-like | 7.41E-55 | 96.70% | 629 |
| L1961 | JT926930.1 | Multiple inositol polyphosphate phosphatase 1 precursor | 1.87E-51 | 89.45% | 489 |
| L1962 | JT959021.1 | small nuclear ribonucleoprotein e-like | 5.38E-55 | 98.70% | 584 |
| L1963 | JT944179.1 | malectin receptor-like protein kinase family | 6.10E-59 | 71.45% | 527 |
| L1964 | JT957660.1 | xylem Serine proteinase 1 | 2.96E-50 | 90.15% | 362 |
| L1965 | JT958128.1 | kxdl motif-containing protein 1 | 1.87E-54 | 92.45% | 586 |
| L1966 | JT952171.1 | RING finger and CHY zinc finger domain-containing protein 1 | 3.86E-09 | 68.15% | 526 |
| L1967 | JT961694.1 | 40S ribosomal protein S25 | 2.60E-41 | 97.20% | 518 |
| L1968 | JT917892.1 | mitochondrial transcription termination factor family protein | 5.97E-30 | 85.50% | 534 |
| L1969 | JT953360.1 | eukaryotic elongation factor 5a-1 isoform partial | 8.62E-107 | 98.50% | 613 |
| L1970 | JR349429.1 | hypothetical protein JCGZ_02169 | 9.10E-18 | 60.33% | 580 |
| L1971 | JT942629.1 | f-box protein skip24 | 3.98E-19 | 83.85% | 617 |
| L1972 | JR362725.1 | 40S ribosomal protein S19-3-like | 2.10E-46 | 95.75% | 441 |
| L1973 | JT920603.1 | exocyst subunit exo70 family protein | 4.14E-17 | 86.60% | 388 |
| L1974 | JT922977.1 | maf-like protein | 2.95E-50 | 80.40% | 551 |
| L1975 | JR345211.1 | probable glutathione peroxidase 5 | 2.39E-45 | 87.60% | 472 |
| L1976 | JT934867.1 | Populus trichocarpa clone POP021-P07* | 5.00E-26 | 71.00% | 491 |
| L1977 | JT944173.1 | glycosyl hydrolase family protein | 4.16E-48 | 88.35% | 594 |
| L1978 | JR365553.1 | leucine-rich repeat extensin-like protein 3-like (LOC102626301)* | 5.00E-70 | 73.00% | 531 |
| L1979 | JR359602.1 | DOF zinc finger protein | 3.68E-18 | 69.60% | 449 |
| L1980 | JT930788.1 | probable protein phosphatase 2c 10-like | 3.90E-24 | 80.75% | 564 |
| L1981 | JT967789.1 | glycine-rich rna-binding protein | 7.84E-24 | 98.30% | 356 |
| L1982 | JT956769.1 | casein kinase II subunit beta-like isoform x2 | 8.25E-52 | 96.55% | 536 |
| L1983 | JR359320.1 | glycine-rich RNA-binding protein mitochondrial | 4.56E-16 | 91.60% | 225 |
| L1984 | JT937658.1 | uncharacterized loc101219977 | 7.91E-107 | 77.15% | 447 |
| L1985 | JR364633.1 | peptidyl-tRNA hydrolase ptrhd1 | 4.09E-57 | 87.70% | 400 |
| L1986 | JT924225.1 | GRAS family transcription factor (GRAS59) | 2.06E-97 | 73.60% | 644 |
| L1987 | JT917426.1 | topless-related protein 4-like | 3.05E-19 | 88.75% | 136 |
| L1988 | JR349667.1 | dead-box ATP-dependent RNA helicase 42 | 4.91E-58 | 79.75% | 483 |
| L1989 | JT930772.1 | mRNA-decapping enzyme-like protein | 2.83E-25 | 75.35% | 530 |
| L1990 | JT934440.1 | snf1-related protein kinase regulatory subunit gamma-1 | 8.09E-99 | 90.75% | 482 |
| L1991 | JR344936.1 | zinc finger mynd domain-containing protein 15 isoform 2 | 8.92E-62 | 83.20% | 479 |
| L1992 | JT955539.1 | Ubiquitin ligase SINAT3 | 4.59E-42 | 73.35% | 565 |
| L1993 | JT963480.1 | Populus euphratica uncharacterized LOC105130085* | 8.00E-29 | 94.00% | 450 |
| L1994 | JR354528.1 |  |  |  | 391 |
| L1995 | JT964192.1 | embryo defective isoform 2 | 1.99E-56 | 79.70% | 462 |
| L1996 | JR345375.1 | 40S ribosomal protein S16 | 1.21E-80 | 97.95% | 502 |
| L1997 | JT954729.1 | chloroplast Cu/Zn superoxide dismutase | 2.56E-09 | 80.45% | 368 |
| L1998 | JT926721.1 | lipase class 3 family protein | 4.66E-100 | 79.60% | 579 |
| L1999 | JT928900.1 | 5-phosphomevelonate kinase | 7.95E-81 | 90.65% | 486 |
| L2000 | JT920193.1 | f-box protein pp2-b10-like | 8.76E-09 | 81.70% | 490 |
| L2001 | JT947097.1 | Ribosome maturation factor Rimm | 5.08E-38 | 68.85% | 588 |
| L2002 | JT963213.1 | protein transport protein sec61 subunit gamma-1 | 5.38E-29 | 97.85% | 439 |
| L2003 | JT935178.1 | probable protein phosphatase 2c 63 | 2.37E-72 | 85.45% | 555 |
| L2004 | JT923996.1 | upf0392 protein rcom_0530710 | 2.53E-08 | 79.33% | 505 |
| L2005 | JT978945.1 |  |  |  | 293 |
| L2006 | JT929309.1 | tubulin alpha-5 | 1.26E-17 | 98.90% | 377 |
| L2007 | JR365994.1 | er membrane protein complex subunit 8/9 homolog | 2.94E-21 | 93.40% | 459 |
| L2008 | JT926846.1 | polyol transporter 5-like | 1.60E-31 | 88.80% | 522 |
| L2009 | JT926380.1 | SART-1 family protein DOT2 | 1.52E-34 | 68.25% | 518 |
| L2010 | JT970734.1 | probable tetraacyldisaccharide 4 - mitochondrial isoform x3 | 2.32E-11 | 71.75% | 320 |
| L2011 | JT931036.1 | presenilin-like protein at1g08700 | 1.10E-21 | 84.50% | 273 |
| L2012 | JR363926.1 | hypothetical protein F383_08210 | 1.07E-06 | 73.50% | 393 |
| L2013 | JT915378.1 | conserved hypothetical protein | 2.73E-51 | 85.75% | 494 |
| L2014 | JT947947.1 | mms19 nucleotide excision repair protein like | 8.78E-05 | 72.00% | 320 |
| L2015 | JT920420.1 | bromodomain and extraterminal domain protein isoform 6 | 2.02E-53 | 75.60% | 544 |
| L2016 | JT974150.1 |  |  |  | 213 |
| L2017 |  | ethylene-responsive transcription factor wri1 | 1.99E-41 | 62.95% | 470 |
| L2018 | JT915386.1 | pwwp domain-containing family protein | 1.28E-23 | 74.35% | 530 |
| L2019 | JT927459.1 | eukaryotic translation initiation factor 2 subunit 3 | 4.74E-80 | 98.95% | 396 |
| L2020 | JT957348.1 |  |  |  | 414 |
| L2021 | JR345560.1 | d-3-phosphoglycerate dehydrogenase family protein | 3.63E-30 | 88.95% | 376 |
| L2022 | JT971322.1 | RING finger containing protein | 2.22E-10 | 69.00% | 336 |
| L2023 | JT963084.1 | uncharacterized loc101207229 | 1.43E-17 | 92.50% | 346 |
| L2024 | JT952422.1 | bonsai protein | 4.94E-14 | 91.45% | 365 |
| L2025 | JT962725.1 | glycine-rich protein 2-like (LOC105141100), transcript variant X2* | 7.00E-16 | 75.00% | 353 |
| L2026 | JT938638.1 | uncharacterized protein TCM_000814 | 3.63E-08 | 62.11% | 434 |
| L2027 | JR363581.1 | protein SKIP34 | 3.77E-25 | 72.89% | 246 |
| L2028 | JT936344.1 | Vacuolar protein sorting protein | 8.89E-05 | 64.00% | 407 |
| L2029 | JT969606.1 | cysteine proteinase inhibitor 5 | 3.43E-38 | 77.85% | 387 |
| L2030 | JT934372.1 | strictosidine synthase 1-like | 8.08E-21 | 59.20% | 415 |
| L2031 | JR358099.1 | vesicle-associated membrane protein 726-like | 1.89E-18 | 99.85% | 244 |
| L2032 | JT953060.1 | hypothetical protein JCGZ_11711 | 3.21E-18 | 72.00% | 343 |
| L2033 | JT934826.1 | phosphatase 2c family protein | 6.59E-31 | 92.10% | 374 |
| L2034 | JT943664.1 | selenoprotein k | 1.34E-18 | 89.10% | 219 |
| L2035 | JR346858.1 | cleavage and polyadenylation specificity factor | 2.33E-12 | 80.14% | 361 |
| L2036 | JT946528.1 | zinc finger family protein | 1.12E-09 | 84.00% | 650 |
| L2037 | JT928282.1 | DUF593-containing protein | 1.53E-53 | 61.45% | 404 |
| L2038 | JT951326.1 | plant intracellular ras-group-related lrr protein 1-like | 4.68E-56 | 88.10% | 597 |
| L2039 | JT956147.1 | rubber elongation factor | 9.21E-94 | 76.00% | 576 |
| L2040 | JT939205.1 | rubber elongation factor family protein | 2.51E-64 | 84.25% | 627 |
| L2041 | JT938284.1 | hexose transporter 1 | 3.16E-34 | 83.40% | 417 |
| L2042 | JR346067.1 | transmembrane protein | 8.83E-21 | 86.35% | 314 |
| L2043 | JT942051.1 | Ras-related protein Raba1f | 1.54E-125 | 98.85% | 624 |
| L2044 | JT931082.1 | pyruvate dehydrogenase e1 component subunit alpha- chloroplastic-like | 1.32E-57 | 93.55% | 365 |
| L2045 | JR349870.1 | f-box protein skip2 | 3.19E-54 | 74.30% | 597 |
| L2046 | JR365344.1 | transcription factor bHLH149-like | 1.13E-79 | 69.95% | 633 |
| L2047 | JT920391.1 | nucleolar protein 10 | 7.48E-45 | 82.65% | 650 |
| L2048 | JT951354.1 | telomere repeat-binding factor 4-like | 5.17E-66 | 73.20% | 383 |
| L2049 | JT967801.1 | wound-induced basic protein | 1.72E-13 | 95.55% | 457 |
| L2050 | JT932799.1 | bel1-like homeodomain protein 1 | 8.83E-59 | 75.70% | 634 |
| L2051 |  | ATP binding protein | 4.06E-38 | 56.65% | 599 |
| L2052 | JT923353.1 | plant calmodulin-binding isoform 1 | 1.99E-20 | 81.95% | 502 |
| L2053 | JT931464.1 | yth domain-containing protein | 5.28E-50 | 73.65% | 507 |
| L2054 | JR345294.1 | conserved hypothetical protein* | 4.00E-58 | 87.00% | 453 |
| L2055 | JT923753.1 | f-box LRR-repeat protein 15 | 6.64E-124 | 93.85% | 632 |
| L2056 | JT948518.1 | phosphatidylinositol-glycan biosynthesis, class f | 1.10E-14 | 83.05% | 551 |
| L2057 | JT952755.1 | proteasome assembly chaperone 3 | 1.04E-65 | 86.95% | 449 |
| L2058 | JT928657.1 | hypothetical protein JCGZ_24585 | 2.18E-92 | 94.20% | 475 |
| L2059 | JR350353.1 | auxin-repressed protein-like protein ARP1 | 1.49E-54 | 88.70% | 376 |
| L2060 | JR364337.1 | hypothetical protein 30 | 1.18E-70 | 80.80% | 545 |
| L2061 | JT936371.1 | abhydrolase domain-containing protein 4 | 3.81E-76 | 91.20% | 521 |
| L2062 | JT954812.1 | 4-hydroxy-4-methyl-2-oxoglutarate aldolase 2 | 4.50E-67 | 96.30% | 508 |
| L2063 | JT958757.1 | conserved hypothetical protein* | 1.00E-45 | 74.00% | 517 |
| L2064 | JT938987.1 | Vacuolar protein sorting-associated protein 32 homolog 2 | 2.91E-75 | 97.15% | 551 |
| L2065 | JT966206.1 | cysteine-rich PDZ-binding protein | 4.83E-61 | 95.20% | 554 |
| L2066 | JT942172.1 | U-box domain-containing protein 38-like | 1.41E-59 | 81.25% | 485 |
| L2067 | JT938612.1 | glycine-rich RNA-binding protein mitochondrial-like | 1.15E-65 | 88.75% | 499 |
| L2068 | JT955937.1 | thioredoxin h | 2.18E-86 | 84.25% | 527 |
| L2069 | JR363535.1 | cysteine proteinase inhibitor 12 | 1.09E-80 | 80.45% | 642 |
| L2070 | JR364570.1 |  |  |  | 489 |
| L2071 | JT948404.1 | multiple myeloma tumor-associated protein 2 homolog | 2.76E-91 | 87.90% | 511 |
| L2072 | JT920734.1 |  |  |  | 409 |
| L2073 | JT924704.1 | f-box kelch-repeat protein at5g60570-like | 1.25E-19 | 97.30% | 453 |
| L2074 | JT942227.1 | ATP-dependent helicase deoxyribonuclease subunit b | 1.15E-100 | 95.25% | 474 |
| L2075 | JT950662.1 | proteasome subunit beta type-2-a | 2.38E-52 | 97.25% | 327 |
| L2076 | JR344577.1 | 60S ribosomal protein L11 | 1.94E-92 | 99.30% | 419 |
| L2077 | JT944719.1 | 60S ribosomal protein L7a | 2.03E-60 | 94.95% | 579 |
| L2078 | JR362463.1 |  |  |  | 486 |
| L2079 | JT955726.1 | transcription factor btf3 | 4.26E-83 | 95.05% | 544 |
| L2080 | JT959962.1 | conserved hypothetical protein | 9.55E-28 | 65.60% | 573 |
| L2081 | JR345999.1 | gamma carbonic anhydrase-like mitochondrial | 2.53E-102 | 89.05% | 498 |
| L2082 | JR366679.1 | metal ion binding protein | 5.35E-89 | 73.65% | 436 |
| L2083 | JR349929.1 | autophagy-related protein 13 | 8.42E-33 | 67.65% | 444 |
| L2084 | JT925015.1 | glycerol-3-phosphate transporter 4 | 5.30E-06 | 70.40% | 465 |
| L2085 | JR360236.1 | protein light-dependent short hypocotyls 4-like | 1.80E-30 | 95.80% | 633 |
| L2086 | JT941264.1 | gata transcription factor 28 | 5.23E-40 | 78.80% | 623 |
| L2087 | JT938110.1 | Coiled-coil domain-containing protein | 1.88E-31 | 78.35% | 428 |
| L2088 |  | hypothetical protein JCGZ_06668 | 6.17E-06 | 64.50% | 401 |
| L2089 | JT956248.1 | 60S ribosomal protein L32-1-like | 2.30E-81 | 98.95% | 568 |
| L2090 | JR361618.1 |  |  |  | 167 |
| L2091 | JT943181.1 | probable disease resistance protein at4g27220 | 8.42E-42 | 65.50% | 516 |
| L2092 | JT917526.1 | disease resistance protein | 5.25E-42 | 60.90% | 478 |
| L2093 | JR344679.1 | cation efflux protein/ zinc transporter | 4.32E-43 | 91.75% | 549 |
| L2094 | JT956228.1 | 40S ribosomal protein S19-3 | 3.93E-96 | 95.85% | 577 |
| L2095 | JT959963.1 | uncharacterized protein TCM_007167 | 1.65E-12 | 76.33% | 621 |
| L2096 | JT922026.1 | mediator of RNA polymerase II transcription subunit 33b-like | 3.62E-05 | 87.55% | 114 |
| L2097 | JT955420.1 | elongation factor 1-alpha | 3.88E-23 | 86.90% | 489 |
| L2098 | JT917754.1 | autophagy-related protein 9 | 3.02E-102 | 66.25% | 662 |
| L2099 | JR364717.1 | hypothetical protein JCGZ_13280 | 2.74E-48 | 69.25% | 561 |
| L2100 | JT932916.1 | tropomyosin- isoform 1 | 8.77E-31 | 67.20% | 384 |
| L2101 | JT936024.1 | RNA recognition motif-containing family protein | 2.89E-38 | 75.88% | 503 |
| L2102 | JT943821.1 | vesicle-associated protein 1-2 | 8.52E-100 | 89.15% | 515 |
| L2103 | JT949430.1 | conserved hypothetical protein | 2.66E-64 | 77.00% | 557 |
| L2104 | JT956915.1 | 60S ribosomal protein L37-1-like | 3.68E-45 | 95.85% | 440 |
| L2105 | JT955496.1 | protein translation factor sui1 homolog | 1.94E-82 | 97.50% | 610 |
| L2106 | JR348260.1 | heat shock factor-binding protein 1-like | 7.16E-46 | 90.30% | 431 |
| L2107 | JT918019.1 | protein pat1 homolog 1-like | 2.04E-23 | 79.70% | 525 |
| L2108 | JT957706.1 | desiccation protectant protein lea14 homolog | 1.41E-91 | 87.45% | 608 |
| L2109 | JT945429.1 | hypothetical protein JCGZ_10524 | 1.34E-48 | 74.75% | 560 |
| L2110 | JT930136.1 | RNA binding protein | 3.06E-58 | 72.25% | 586 |
| L2111 | JT935252.1 | auxin-responsive family protein | 1.32E-87 | 89.55% | 454 |
| L2112 | JT925444.1 | cytoskeletal protein mRNA | 2.87E-31 | 82.15% | 531 |
| L2113 | JT920476.1 | RNA polymerase II subunit B1 CTD phosphatase RPAP2 homolog | 7.51E-16 | 70.00% | 584 |
| L2114 | JR344729.1 | LOB domain-containing protein 4 | 3.60E-54 | 76.80% | 595 |
| L2115 | JT935639.1 | RING finger protein 214 isoform 1 | 3.37E-80 | 92.05% | 646 |
| L2116 | JT962238.1 | mitochondrial import inner membrane translocase subunit Tim13-like | 2.54E-30 | 93.00% | 366 |
| L2117 | JT966645.1 | fiber protein fb11 | 2.41E-28 | 90.70% | 559 |
| L2118 | JR366287.1 | mitochondrial thioredoxin 2 | 1.37E-21 | 100.00% | 270 |
| L2119 | JR347869.1 | 70 kDa peptidyl-prolyl isomerase-like | 4.35E-56 | 90.95% | 595 |
| L2120 | JT950734.1 | acid phosphatase vanadium-dependent haloperoxidase-related protein isoform 1 | 2.89E-76 | 90.70% | 485 |
| L2121 | JT929899.1 | DNA binding protein | 1.35E-24 | 80.91% | 200 |
| L2122 | JT933912.1 | histone-lysine n-methyltransferase ashr2 | 1.28E-19 | 80.45% | 508 |
| L2123 | JT965296.1 |  |  |  | 494 |
| L2124 | JR366165.1 | WRKY transcription | 3.26E-29 | 79.20% | 479 |
| L2125 | JT949857.1 | splicing factor, arginine/serine-rich 19 | 2.60E-30 | 94.35% | 456 |
| L2126 | JT967042.1 | eg45-like domain containing protein | 1.74E-67 | 84.80% | 557 |
| L2127 | JT945274.1 | zinc finger A20 and AN1 domain-containing stress-associated protein 3-like | 1.12E-43 | 61.95% | 375 |
| L2128 | JT962435.1 | PHD finger protein ALFIN-LIKE 2 | 1.04E-46 | 88.00% | 459 |
| L2129 | JT966799.1 | uncharacterized crm domain-containing protein chloroplastic | 5.42E-33 | 70.30% | 491 |
| L2130 | JT952847.1 | prefoldin subunit 6-like | 9.06E-79 | 95.85% | 554 |
| L2131 | JR356640.1 | zinc finger protein 5-like | 1.44E-09 | 66.60% | 484 |
| L2132 | JT951460.1 | E3 ubiquitin-protein ligase at1g63170 isoform x1 | 7.48E-33 | 87.80% | 497 |
| L2133 | JT957034.1 | hypothetical protein POPTR_0012s13810g | 6.90E-40 | 100.00% | 454 |
| L2134 | JT949255.1 | thioredoxin chloroplastic | 2.12E-40 | 93.55% | 498 |
| L2135 | JT945570.1 | rrp15-like protein | 7.18E-49 | 91.80% | 443 |
| L2136 | JR361845.1 | conserved hypothetical protein* | 5.00E-31 | 92.00% | 400 |
| L2137 | JT959344.1 | c globular | 3.73E-34 | 69.15% | 516 |
| L2138 | JT939682.1 | protein dehydration-induced 19 homolog 3-like | 2.49E-26 | 95.55% | 545 |
| L2139 | JT935624.1 | wall-associated receptor kinase 2-like | 2.67E-16 | 67.00% | 436 |
| L2140 | JT933538.1 | ABC transporter B family member 19 | 2.13E-123 | 95.00% | 584 |
| L2141 | JT964773.1 | conserved hypothetical protein | 8.50E-28 | 63.65% | 556 |
| L2142 | JT958537.1 | Serine hydroxymethyltransferase mitochondrial-like | 6.87E-33 | 55.05% | 660 |
| L2143 | JR345967.1 | hypothetical protein JCGZ_09364 | 4.70E-16 | 70.00% | 522 |
| L2144 | JR365799.1 | starch branching enzyme II | 2.28E-11 | 65.50% | 649 |
| L2145 | JT942483.1 | plasma membrane intrinsic protein PIP2;3 | 1.69E-149 | 100.00% | 619 |
| L2146 | JT946107.1 | chitinase family protein | 5.43E-97 | 87.60% | 671 |
| L2147 | JR349168.1 | tobamovirus multiplication protein 2a-like | 1.49E-51 | 86.20% | 606 |
| L2148 | JR347725.1 |  |  |  | 647 |
| L2149 | JT960955.1 | nucleic acid binding protein | 1.14E-38 | 81.80% | 646 |
| L2150 | JT963619.1 | 60S ribosomal protein L18a-like protein | 4.92E-21 | 91.55% | 481 |
| L2151 | JT975969.1 | hypothetical protein POPTR_0012s07860g | 2.03E-26 | 92.15% | 680 |
| L2152 | JT934106.1 | pre-mRNA splicing factor prp38 family protein | 4.43E-07 | 92.00% | 578 |
| L2153 | JR360270.1 | nuclear fusion defective 6 isoform 1 | 1.10E-33 | 83.85% | 678 |
| L2154 | JT926798.1 | prephenate dehydratase family protein | 4.68E-27 | 57.70% | 664 |
| L2155 | JT949912.1 | 60S ribosomal protein L19-2 | 1.35E-95 | 99.90% | 531 |
| L2156 | JT957573.1 | lipid phosphate phosphatase epsilon chloroplastic | 4.85E-24 | 63.71% | 617 |
| L2157 | JT958480.1 | 60S ribosomal protein L34-like | 4.38E-60 | 99.50% | 681 |
| L2158 | JR363533.1 | zinc finger family protein | 2.46E-57 | 90.45% | 517 |
| L2159 | JT962459.1 | eg5651 | 4.38E-34 | 68.70% | 579 |
| L2160 | JT914420.1 | enhancer of polycomb-like transcription factor isoform 1 | 6.50E-91 | 77.60% | 635 |
| L2161 | JT942985.1 | tubby like protein 3 isoform partial | 2.45E-29 | 96.70% | 194 |
| L2162 | JT962101.1 |  |  |  | 469 |
| L2163 | JT962531.1 | metallothionein-like protein | 8.12E-28 | 81.10% | 640 |
| L2164 | JR365360.1 | 60S ribosomal protein L14 | 7.00E-86 | 95.80% | 622 |
| L2165 | JT942256.1 | wound-induced protein win2 | 1.09E-47 | 89.60% | 620 |
| L2166 | JT953117.1 | 60S ribosomal protein L9 | 7.02E-90 | 97.05% | 605 |
| L2167 | JT956731.1 | dual specificity protein kinase spla | 3.27E-83 | 96.45% | 620 |
| L2168 | JT916889.1 | probable cyclic nucleotide-gated ion channel 5-like isoform x1 | 2.59E-27 | 82.80% | 429 |
| L2169 | JT940188.1 | RNA recognition motif-containing family protein | 1.20E-19 | 87.05% | 498 |
| L2170 | JR346155.1 | triacylglycerol lipase | 2.47E-37 | 69.00% | 585 |
| L2171 | JT936659.1 | hypothetical protein 28 | 1.14E-143 | 75.86% | 692 |
| L2172 | JT942348.1 | fh protein interacting protein fip2-like | 3.04E-112 | 76.50% | 600 |
| L2173 | JR365513.1 | peptidyl-prolyl cis-trans isomerase fkbp20-1 | 5.02E-82 | 93.25% | 662 |
| L2174 | JT927768.1 | PREDICTED: uncharacterized protein LOC104905041 | 1.90E-34 | 56.35% | 507 |
| L2175 | JT947475.1 | protein BUD31 homolog 2 | 9.63E-102 | 96.90% | 634 |
| L2176 | JR347307.1 |  |  |  | 641 |
| L2177 | JR361471.1 | mitochondrial DNA* | 4.00E-115 | 94.00% | 497 |
| L2178 | JR353939.1 | abscisic acid receptor pyl4-like | 1.83E-43 | 89.75% | 575 |
| L2179 | JT930790.1 | pore-forming toxin-like protein hfr-2 | 2.78E-58 | 61.10% | 628 |
| L2180 | JT965620.1 | eukaryotic translation initiation factor 4e-1-like | 3.36E-41 | 66.25% | 688 |
| L2181 | JT972279.1 | hypothetical protein JCGZ_19016 | 5.91E-14 | 86.75% | 353 |
| L2182 | JR366199.1 | dentin sialophosphoprotein-like | 7.53E-78 | 64.70% | 675 |
| L2183 | JT946523.1 | glutathione s-transferase | 1.07E-44 | 64.85% | 584 |
| L2184 | JT953793.1 | nuclear transport factor 2-like | 1.50E-81 | 95.80% | 669 |
| L2185 | JT947320.1 | hypothetical protein JCGZ_24786 | 3.28E-16 | 85.50% | 339 |
| L2186 | JT969420.1 | dctp pyrophosphatase 1-like | 3.15E-63 | 94.90% | 498 |
| L2187 | JT930067.1 | elongation factor 1-alpha | 3.96E-113 | 99.00% | 478 |
| L2188 | JT925241.1 | cysteine desulfurylase | 1.27E-93 | 74.20% | 728 |
| L2189 | JT929467.1 | chorismate synthase family protein | 9.04E-95 | 93.10% | 567 |
| L2190 | JT963390.1 | uncharacterized loc101212188 | 7.82E-86 | 86.50% | 667 |
| L2191 | JT929780.1 | probable WRKY transcription factor 21 | 4.34E-99 | 76.05% | 687 |
| L2192 |  | desi-like protein at4g17486 | 1.14E-92 | 93.35% | 553 |
| L2193 | JT954077.1 | embryo-specific family protein | 2.38E-81 | 80.75% | 597 |
| L2194 | JR366714.1 | Alpha-1,4-glucan-protein synthase [UDP-forming] | 6.20E-13 | 80.21% | 404 |
| L2195 | JT973106.1 | uncharacterized loc101212373 | 1.30E-25 | 93.50% | 531 |
| L2196 | JT951808.1 | ATP binding protein | 9.45E-79 | 86.85% | 530 |
| L2197 | JT953981.1 | f-box protein skip8-like | 6.21E-45 | 68.45% | 658 |
| L2198 | JR366465.1 | nucleic acid binding protein | 1.55E-121 | 88.20% | 673 |
| L2199 | JT961431.1 | 60S ribosomal protein L31 | 5.08E-64 | 94.60% | 592 |
| L2200 | JT957093.1 | hypothetical protein JCGZ_05881 | 6.23E-13 | 74.00% | 603 |
| L2201 | JT965909.1 | hypothetical protein JCGZ_25112 | 4.44E-13 | 83.50% | 502 |
| L2202 | JT955858.1 | probable calcium-binding protein cml20 | 1.49E-80 | 90.70% | 624 |
| L2203 | JR366768.1 | mediator of RNA polymerase II transcription subunit 4 | 1.52E-53 | 86.45% | 684 |
| L2204 | JR346726.1 | electron transport complex protein isoform 1 | 2.55E-15 | 67.00% | 595 |
| L2205 |  | plastid division protein pdv1 | 5.54E-52 | 72.15% | 652 |
| L2206 | JT945290.1 | NADPH-dependent pterin aldehyde reductase | 2.24E-121 | 90.65% | 667 |
| L2207 | JT948141.1 | transcription factor bHLH49-like (LOC105123366), transcript variant X4* | 2.00E-77 | 75.00% | 665 |
| L2208 | JT947608.1 | E3 ubiquitin-protein ligase rnf185 isoform x1 | 1.96E-110 | 88.70% | 721 |
| L2209 | JT967675.1 | transcription factor par1 | 2.02E-40 | 72.80% | 646 |
| L2210 | JT950699.1 | ADP-ribosylation factor 2 | 1.38E-82 | 96.00% | 680 |
| L2211 | JT925313.1 | ankyrin repeat family protein | 4.30E-36 | 89.75% | 485 |
| L2212 | JT966574.1 | wound-induced protein win1 | 1.30E-55 | 82.90% | 552 |
| L2213 | JT928079.1 | D-tagatose-1,6-bisphosphate aldolase subunit kbaZ | 2.66E-62 | 87.70% | 609 |
| L2214 | JT923008.1 | Serine threonine-protein kinase | 9.65E-73 | 84.40% | 675 |
| L2215 | JR364041.1 | DNA-directed RNA polymerases IV and V subunit 12 | 8.58E-28 | 94.80% | 557 |
| L2216 | JT930729.1 | E3 ubiquitin-protein ligase at3g02290-like | 9.94E-56 | 76.60% | 467 |
| L2217 | JT948224.1 | NADH dehydrogenase | 1.11E-122 | 92.05% | 629 |
| L2218 | JT931864.1 |  |  |  | 507 |
| L2219 | JR361486.1 | f-box only 46 | 8.33E-38 | 92.95% | 495 |
| L2220 | JR351585.1 | trihelix transcription factor gt-2-like | 7.12E-58 | 58.40% | 601 |
| L2221 | JT939270.1 | protein yls9-like | 2.13E-97 | 85.25% | 643 |
| L2222 | JT940299.1 | flavohemoprotein B5/b5r | 8.42E-33 | 65.55% | 544 |
| L2223 | JT942604.1 | hypothetical protein JCGZ_04721 | 2.32E-83 | 87.95% | 694 |
| L2224 | JT938366.1 | guanine nucleotide-binding protein subunit beta-like protein | 3.50E-82 | 93.25% | 564 |
| L2225 | JT957531.1 | conserved hypothetical protein | 5.48E-31 | 62.25% | 489 |
| L2226 | JT926339.1 | high mobility group beta isoform 1 | 1.29E-44 | 95.70% | 597 |
| L2227 | JT938754.1 | dehydrogenase reductase sdr family member 12 | 1.73E-74 | 87.35% | 637 |
| L2228 | JR356166.1 | transcription factor TCP15-like | 3.95E-40 | 95.35% | 638 |
| L2229 | JT956451.1 | werner syndrome-like exonuclease | 2.32E-115 | 76.45% | 692 |
| L2230 | JT935609.1 | DNA ligase 1 | 4.99E-69 | 74.20% | 489 |
| L2231 | JT949135.1 | sumo-conjugating enzyme sce1 | 1.04E-111 | 96.85% | 663 |
| L2232 | JT939642.1 | myosin heavy chain-related family protein | 3.30E-30 | 69.60% | 666 |
| L2233 | JT950868.1 | polypeptide n-acetylgalactosaminyltransferase 11 | 4.85E-94 | 64.60% | 633 |
| L2234 | JT916171.1 | mitochondrial ATP synthase subunit g protein | 1.32E-79 | 95.85% | 633 |
| L2235 | JR357418.1 | uncharacterized loc101212909 | 3.69E-43 | 66.00% | 625 |
| L2236 | JT980542.1 | RING-H2 finger protein ATL1Q | 2.04E-49 | 73.40% | 550 |
| L2237 | JT938943.1 | alg-2 interacting protein x-like | 4.47E-93 | 92.50% | 567 |
| L2238 | JT931826.1 | o-acyltransferase wsd1-like | 9.66E-43 | 71.95% | 594 |
| L2239 | JT916764.1 | pumilio homolog 2-like | 2.58E-14 | 92.40% | 657 |
| L2240 | JT961569.1 | PREDICTED: uncharacterized protein LOC104227135 | 3.07E-54 | 91.10% | 686 |
| L2241 | JR346584.1 | V-type proton ATPase subunit g 1-like | 8.08E-64 | 89.45% | 501 |
| L2242 | JT941242.1 | glycine-rich protein | 3.29E-81 | 70.50% | 636 |
| L2243 | JT945856.1 | calcyclin-binding | 2.58E-99 | 93.30% | 655 |
| L2244 | JR366661.1 | f-box protein skip19-like | 1.67E-17 | 78.00% | 593 |
| L2245 | JT944324.1 | tbc domain-containing partial | 1.16E-47 | 95.80% | 570 |
| L2246 | JT954590.1 | actin-depolymerizing factor | 8.67E-49 | 92.80% | 477 |
| L2247 | JT928803.1 | aspartate aminotransferase | 2.14E-49 | 83.50% | 527 |
| L2248 | JT945065.1 | Ras-related protein Rha1 | 2.71E-88 | 99.30% | 592 |
| L2249 | JT946770.1 | GTP-binding nuclear protein Ran-3 | 3.09E-60 | 84.00% | 414 |
| L2250 | JT949624.1 | probable gpi-anchored adhesin-like protein pga55 | 2.62E-34 | 55.37% | 507 |
| L2251 | JT956996.1 | PREDICTED: uncharacterized protein LOC103343500 | 5.69E-33 | 85.35% | 378 |
| L2252 | JT942978.1 | histone H1 | 5.69E-29 | 81.25% | 658 |
| L2253 | JR365652.1 | MFP1 attachment factor 1-like | 1.02E-67 | 78.20% | 667 |
| L2254 | JT937547.1 | cinnamyl alcohol dehydrogenase-like protein (CADL6) | 4.16E-27 | 90.25% | 683 |
| L2255 | JR349294.1 | f-box protein at1g47056-like | 6.64E-81 | 82.10% | 584 |
| L2256 | JT920578.1 | zinc finger CCCH domain-containing protein 30 | 2.26E-75 | 91.75% | 651 |
| L2257 | JT941727.1 | spx domain-containing protein 1-like | 5.14E-77 | 85.10% | 693 |
| L2258 | JT953995.1 | ankyrin repeat family isoform 1 | 1.10E-18 | 69.50% | 509 |
| L2259 | JT923392.1 | ATP-dependent clp protease ATP-binding subunit clpx- mitochondrial | 1.98E-30 | 84.75% | 503 |
| L2260 | JT930045.1 | cytochrome P450 51 family protein | 6.46E-124 | 95.30% | 594 |
| L2261 | JT957175.1 | PREDICTED: uncharacterized protein LOC103953524 | 2.82E-16 | 84.10% | 540 |
| L2262 | JT932744.1 | RNA polymerase II degradation factor 1-like isoform x5 | 4.96E-15 | 73.00% | 559 |
| L2263 | JT915852.1 | phytochrome A | 1.59E-93 | 87.80% | 585 |
| L2264 | JR363704.1 | U6 snRNA-associated sm-like protein lsm3 | 7.18E-53 | 98.00% | 553 |
| L2265 | JT930486.1 | protein far1-related sequence 7-like | 1.06E-60 | 90.00% | 339 |
| L2266 | JT971226.1 | mevalonate kinase | 1.38E-11 | 96.85% | 285 |
| L2267 | JT973007.1 |  |  |  | 347 |
| L2268 | JT936129.1 | peptidyl-prolyl cis-trans isomerase cyp20-1-like isoform x1 | 1.84E-85 | 85.65% | 618 |
| L2269 | JT933798.1 | transmembrane protein | 2.83E-32 | 69.60% | 460 |
| L2270 | JT935381.1 | arginine Serine-rich coiled-coil 2 | 5.78E-56 | 72.45% | 600 |
| L2271 | JT942451.1 | unnamed protein product | 4.76E-10 | 78.11% | 453 |
| L2272 | JT917867.1 | zinc ion binding protein | 9.68E-07 | 67.95% | 233 |
| L2273 | JT919378.1 | hydrolase family protein | 1.10E-68 | 88.45% | 661 |
| L2274 | JT951102.1 | f-box protein skip5 | 1.17E-78 | 88.20% | 491 |
| L2275 | JT949790.1 | mitochondrial adenine nucleotide transporter adnt1 | 3.16E-50 | 95.95% | 632 |
| L2276 | JT959252.1 | thioredoxin-like protein clot | 3.28E-58 | 94.45% | 577 |
| L2277 | JT954326.1 | 2-deoxyglucose-6-phosphate phosphatase | 8.63E-89 | 92.50% | 590 |
| L2278 | JT926687.1 | integrin-linked protein kinase family isoform 1 | 2.27E-46 | 83.25% | 407 |
| L2279 | JR366494.1 | basic leucine zipper 9-like | 1.31E-38 | 65.60% | 539 |
| L2280 | JR366375.1 | abscisic acid receptor pyl4-like | 1.30E-62 | 74.00% | 545 |
| L2281 | JT926541.1 | phosphoenolpyruvate carboxylase family protein isoform 2 | 7.23E-12 | 84.30% | 562 |
| L2282 | JT960927.1 | histone H2B | 5.02E-54 | 97.90% | 452 |
| L2283 | JR345633.1 | arginine/Serine-rich splicing factor | 5.36E-180 | 79.55% | 327 |
| L2284 | JT926060.1 | phosphatase 2c family protein | 6.81E-125 | 94.80% | 586 |
| L2285 | JT949974.1 | 6-phosphogluconate decarboxylating 3 | 6.37E-143 | 97.60% | 681 |
| L2286 | JT938112.1 | 50S ribosomal protein L3 | 1.52E-33 | 88.65% | 518 |
| L2287 | JT948250.1 | ganglioside-induced differentiation-associated protein 2-like | 1.89E-77 | 92.75% | 493 |
| L2288 | JT961776.1 | mitochondrial pyruvate carrier 1-like | 3.44E-67 | 90.95% | 491 |
| L2289 | JT915680.1 | chromodomain-helicase-DNA-binding protein 1-like isoform x1 | 2.40E-70 | 91.35% | 495 |
| L2290 | JT943002.1 | vesicle-associated membrane protein 711 | 1.37E-129 | 96.35% | 708 |
| L2291 | JT921140.1 | transmembrane 9 superfamily member 8 | 7.05E-41 | 97.95% | 507 |
| L2292 | JT946677.1 | senescence-associated family protein | 4.22E-34 | 58.40% | 438 |
| L2293 | JT925157.1 | g-box-binding factor 4-like | 2.78E-83 | 67.50% | 451 |
| L2294 | JT940222.1 | transcription factor TCP20-like | 5.78E-139 | 80.35% | 344 |
| L2295 | JT933970.1 | probable protein phosphatase 2c 38 isoform x1 | 5.36E-18 | 88.60% | 609 |
| L2296 | JR364293.1 | hypothetical protein JCGZ_20265 | 2.48E-46 | 69.60% | 267 |
| L2297 | JT942675.1 | 60S ribosomal protein L7-2 | 1.08E-141 | 92.95% | 143 |
| L2298 | JT922528.1 | ATPase family AAA domain-containing protein 3-like | 2.11E-21 | 93.20% | 372 |
| L2299 | JR365944.1 | eukarytic translation initiation factor 4e | 1.59E-09 | 87.17% | 379 |
| L2300 | JT927700.1 | allene oxide synthase | 1.22E-55 | 74.80% | 624 |
| L2301 | JT957937.1 | NADH dehydrogenase | 2.36E-22 | 85.50% | 449 |
| L2302 | JT932179.1 | transcriptional factor b3 family protein | 3.95E-20 | 74.45% | 242 |
| L2303 | JR357022.1 | glutaredoxin-c6-like isoform x3 | 3.07E-66 | 77.50% | 431 |
| L2304 | JT943091.1 | homeobox-leucine zipper protein hat22-like | 1.09E-70 | 77.05% | 564 |
| L2305 | JT940465.1 | polyubiquitin | 1.09E-91 | 99.55% | 618 |
| L2306 | JR364649.1 | hypothetical protein JCGZ_07434 | 5.35E-05 | 93.00% | 534 |
| L2307 | JT916138.1 | ATP-dependent zinc metalloprotease ftsh chloroplastic | 1.72E-99 | 84.20% | 585 |
| L2308 | JT933647.1 | acetyl-CoA C-acetyltransferas | 6.32E-22 | 95.50% | 486 |
| L2309 | JT934021.1 | uncharacterized membrane protein at4g09580 | 3.22E-73 | 96.05% | 558 |
| L2310 | JT931246.1 | syntaxin t-snare family isoform 1 | 5.15E-19 | 77.80% | 504 |
| L2311 | JT958760.1 |  |  |  | 444 |
| L2312 | JT914664.1 | RNA polymerase II-associated protein | 5.92E-34 | 70.95% | 463 |
| L2313 | JT939521.1 | ganglioside-induced differentiation-associated protein 2 | 3.18E-45 | 88.75% | 549 |
| L2314 | JR366074.1 | pectinesterase pectinesterase inhibitor 26 | 2.45E-49 | 69.15% | 562 |
| L2315 | JT932252.1 | protein sqs1-like | 5.81E-41 | 64.45% | 497 |
| L2316 | JT939872.1 | protein yippee-like at5g53940 | 3.90E-63 | 84.30% | 519 |
| L2317 | JT967947.1 | sulfate transporter | 8.76E-13 | 71.50% | 505 |
| L2318 | JT918493.1 | phosphatidylinositol-4-phosphate 5-kinase family protein | 3.35E-08 | 81.67% | 472 |
| L2319 | JT945910.1 | DNA-binding protein MNB1B | 2.47E-35 | 94.30% | 592 |
| L2320 | JT925007.1 | dicarboxylate transporter chloroplastic-like | 1.01E-65 | 89.50% | 536 |
| L2321 | JT932780.1 | 26S proteasome ATPase subunit | 4.25E-82 | 98.70% | 544 |
| L2322 | JT942148.1 | cwf19-like protein 2 | 5.78E-48 | 74.65% | 573 |
| L2323 | JT946229.1 | glycine-rich isoform 1 | 3.48E-107 | 78.00% | 234 |
| L2324 | JT963315.1 | senescence-associated protein | 3.25E-55 | 88.95% | 382 |
| L2325 | JT940183.1 | f-box protein pp2-a15-like | 2.43E-128 | 91.95% | 589 |
| L2326 | JT933885.1 | la-related protein 6a | 1.34E-54 | 79.70% | 594 |
| L2327 | JT945551.1 | rRNA biogenesis protein rrp36-like | 2.28E-44 | 88.50% | 472 |
| L2328 | JT939197.1 | phosphate-responsive family protein | 1.05E-88 | 80.15% | 528 |
| L2329 | JT922302.1 | apo protein mitochondrial-like | 3.12E-151 | 78.45% | 576 |
| L2330 | JT948749.1 | copper transport protein | 1.87E-24 | 95.65% | 600 |
| L2331 | JT927359.1 | f-box LRR-repeat protein 4 isoform x1 | 2.22E-121 | 93.20% | 600 |
| L2332 | JT947516.1 | DNA replication complex gins protein sld5 | 1.75E-75 | 77.15% | 472 |
| L2333 | JT933790.1 | probable gpi-anchored adhesin-like protein pga55 | 2.78E-29 | 53.60% | 583 |
| L2334 | JR366758.1 | RNA-binding family protein | 4.20E-61 | 71.90% | 386 |
| L2335 | JT955658.1 | 37S ribosomal protein S16 | 2.30E-68 | 97.40% | 437 |
| L2336 | JT915926.1 | probable ATP-dependent DNA helicase chr12 | 3.94E-33 | 91.85% | 579 |
| L2337 | JT944554.1 | ADP-ribosylation factor | 3.93E-100 | 99.25% | 569 |
| L2338 | JT964204.1 | CBS domain-containing protein chloroplastic-like | 6.11E-41 | 94.65% | 423 |
| L2339 | JT960149.1 |  |  |  | 589 |
| L2340 | JR362825.1 | l-type lectin-domain containing receptor kinase | 3.85E-05 | 67.00% | 666 |
| L2341 | JR344379.1 | wound-induced protein win2 | 8.06E-86 | 90.70% | 612 |
| L2342 | JR361981.1 | nutrient reservoir, putative | 6.00E-05 | 80.00% | 626 |
| L2343 | JR347496.1 | pyruvate dehydrogenase family protein | 1.04E-80 | 88.25% | 523 |
| L2344 | JT965694.1 | 40S ribosomal protein S9 | 4.44E-24 | 89.45% | 531 |
| L2345 | JT938377.1 | Aspartic proteinase-like protein 2 | 9.20E-122 | 88.20% | 590 |
| L2346 | JT962962.1 | conserved hypothetical protein | 6.38E-16 | 67.00% | 440 |
| L2347 | JT952580.1 | ADP-ribosylation factor-like protein | 1.49E-113 | 91.80% | 549 |
| L2348 | JT971116.1 | avr9 cf-9 rapidly elicited protein | 2.21E-24 | 61.45% | 551 |
| L2349 | JT926948.1 | calnexin precursor family protein | 5.70E-16 | 91.35% | 320 |
| L2350 | JR359213.1 | bi1-like protein | 3.69E-15 | 87.25% | 502 |
| L2351 | JT932486.1 | s-adenosylmethionine synthase 5 | 7.37E-48 | 94.55% | 562 |
| L2352 | JR365074.1 | wound-induced protein 1 | 3.88E-39 | 78.15% | 616 |
| L2353 | JT943037.1 | nodulin 35 family protein | 1.00E-79 | 89.75% | 638 |
| L2354 | JT965972.1 | hypothetical protein CICLE_v10010135mg | 1.40E-05 | 88.50% | 538 |
| L2355 | JT975761.1 | 40S ribosomal protein S5* | 3.00E-34 | 74.00% | 386 |
| L2356 | JT953522.1 | endoribonuclease l-psp family protein | 3.50E-30 | 93.15% | 508 |
| L2357 | JT952088.1 | zinc finger family protein | 9.42E-75 | 74.90% | 562 |
| L2358 | JR346599.1 | probable small nuclear ribonucleoprotein G | 5.52E-32 | 96.00% | 406 |
| L2359 | JT941742.1 | zinc finger A20 and AN1 domain-containing stress-associated protein 7 | 1.13E-39 | 67.30% | 579 |
| L2360 | JT972872.1 | protease inhibitor protein 1 (PI1) | 5.82E-42 | 78.85% | 300 |
| L2361 | JT921283.1 | k+ efflux antiporter 4 | 2.01E-12 | 79.93% | 563 |
| L2362 | JT940995.1 | proteasome subunit beta type-5 | 1.34E-37 | 89.05% | 251 |
| L2363 | JT969769.1 | hydroxyproline-rich glycoprotein family protein | 2.98E-51 | 82.90% | 390 |
| L2364 | JT935514.1 | enhancer of mRNA-decapping protein 4-like | 3.30E-85 | 68.00% | 432 |
| L2365 | JT925394.1 | myosin-j heavy chain-like isoform x2 | 7.43E-77 | 62.25% | 543 |
| L2366 | JT914561.1 | dynamin-like protein arc5 | 1.42E-107 | 90.25% | 519 |
| L2367 | JT950389.1 | skp1-like protein 1b | 6.59E-14 | 94.10% | 533 |
| L2368 | JT968005.1 |  |  |  | 494 |
| L2369 | JT965307.1 | hypothetical protein JCGZ_01521 | 1.27E-39 | 70.40% | 501 |
| L2370 | JT921866.1 | pyridoxine pyridoxamine 5 -phosphate oxidase chloroplastic | 6.37E-18 | 89.40% | 473 |
| L2371 | JT957626.1 | Ubiquitin-nedd8-like protein rub2 | 1.31E-42 | 97.45% | 486 |
| L2372 | JR364996.1 | 50S ribosomal protein L20 | 2.21E-81 | 96.25% | 488 |
| L2373 | JT923318.1 | pentatricopeptide repeat-containing protein at1g02420 | 5.72E-17 | 86.65% | 559 |
| L2374 | JT920176.1 | trehalose-6-phosphate synthase, putative | 2.98E-86 | 96.60% | 612 |
| L2375 | JT959134.1 | cytochrome c oxidase assembly protein cox19-like | 1.37E-34 | 83.00% | 558 |
| L2376 | JR344302.1 | Ribulose bisphosphate carboxylase small chain clone 512-like | 6.50E-103 | 88.75% | 509 |
| L2377 | JT957416.1 | neuronal pas domain-containing protein 4 | 1.08E-16 | 79.20% | 366 |
| L2378 | JT957334.1 | metal ion binding protein | 5.07E-22 | 61.80% | 384 |
| L2379 | JR345453.1 | probable u6 snRNA-associated sm-like protein lsm4 | 1.50E-30 | 99.50% | 536 |
| L2380 | JT944942.1 | DUF1639 family protein | 1.67E-98 | 62.55% | 496 |
| L2381 | JT972688.1 |  |  |  | 423 |
| L2382 | JT941158.1 | thylakoid lumenal kDa chloroplastic-like | 2.46E-20 | 73.05% | 519 |
| L2383 | JT941109.1 | groes chaperonin | 1.01E-68 | 94.95% | 511 |
| L2384 | JT936929.1 | acid phosphatase 1-like | 5.82E-29 | 81.40% | 552 |
| L2385 | JT939206.1 | translation elongation factor ef1b ribosomal protein s6 family protein isoform 2 | 1.84E-44 | 95.10% | 635 |
| L2386 | JT950889.1 | kinesin like protein | 1.41E-56 | 83.35% | 507 |
| L2387 | JT940715.1 | importin subunit alpha-1-like | 5.15E-123 | 94.25% | 635 |
| L2388 | JT930993.1 | 5-methyltetrahydropteroyltriglutamate--homocysteine methyltransferase-like | 5.43E-100 | 97.00% | 526 |
| L2389 | JT963119.1 | uncharacterized loc101219597 | 1.94E-26 | 87.55% | 511 |
| L2390 | JT951309.1 | guanine nucleotide-binding protein subunit gamma 2-like | 1.50E-42 | 77.70% | 628 |
| L2391 | JT926237.1 | t-complex protein 1 subunit eta-like | 7.16E-96 | 96.55% | 545 |
| L2392 | JT940362.1 | kinase superfamily protein isoform 1 | 9.09E-104 | 97.80% | 476 |
| L2393 | JR364545.1 | 50S ribosomal protein L34 | 4.54E-36 | 67.15% | 275 |
| L2394 | JT939156.1 | phenylalanyl-tRNA synthetase | 2.05E-62 | 88.40% | 549 |
| L2395 | JT953229.1 | early nodulin-93-like | 1.68E-44 | 77.75% | 624 |
| L2396 | JT948911.1 | uncharacterized oxidoreductase isoform x2 | 1.86E-59 | 94.40% | 573 |
| L2397 | JT944654.1 | secretory carrier membrane family protein | 1.52E-39 | 93.25% | 447 |
| L2398 | JT955989.1 | copper-transporting ATPase chloroplastic-like isoform x1 | 2.34E-34 | 88.65% | 631 |
| L2399 |  | protein EIN4 | 5.36E-14 | 88.05% | 438 |
| L2400 | JT929737.1 | 6-phosphofructokinase 3 | 1.29E-61 | 79.95% | 585 |
| L2401 | JT957216.1 | PHD finger-like domain-containing protein 5b | 9.51E-73 | 98.45% | 586 |
| L2402 | JT974122.1 | histone H3 | 3.38E-72 | 98.50% | 559 |
| L2403 | JT930507.1 | sphingosine kinase 2-like isoform x2 | 1.40E-62 | 87.65% | 494 |
| L2404 | JT961236.1 | polyubiquitin 9 | 2.99E-29 | 89.60% | 563 |
| L2405 | JT956115.1 | probable homogentisate phytyltransferase chloroplastic-like | 2.19E-127 | 84.50% | 606 |
| L2406 | JT952101.1 | quinone-oxidoreductase chloroplastic | 1.63E-116 | 89.50% | 628 |
| L2407 | JT947799.1 | general transcription factor IIe subunit 1-like | 2.43E-81 | 96.40% | 387 |
| L2408 | JT959351.1 | ATP synthase 6 kDa mitochondrial | 8.34E-30 | 95.05% | 552 |
| L2409 | JT935851.1 | early-responsive to dehydration family protein | 1.25E-21 | 83.50% | 610 |
| L2410 | JR365091.1 |  |  |  | 519 |
| L2411 | JR366753.1 | early-responsive to dehydration 7 family protein | 3.04E-73 | 89.00% | 623 |
| L2412 | JR364234.1 | chaperone protein DNAj-related | 5.64E-53 | 90.25% | 571 |
| L2413 | JT964828.1 | MADS-box transcription | 2.44E-24 | 70.75% | 650 |
| L2414 | JT945311.1 | histone H1 | 4.73E-63 | 73.30% | 634 |
| L2415 | JT940277.1 | homeobox-leucine zipper protein athb-12-like | 1.96E-69 | 67.75% | 500 |
| L2416 | JT916296.1 | transcription initiation factor | 5.26E-32 | 73.75% | 664 |
| L2417 | JT963078.1 | 60S ribosomal protein L6 | 5.76E-59 | 96.75% | 547 |
| L2418 | JT919514.1 | fg-gap repeat-containing family protein | 1.25E-56 | 93.15% | 736 |
| L2419 | JT925184.1 | DOF zinc finger protein | 1.01E-16 | 74.60% | 578 |
| L2420 | JT951021.1 | ap-1 complex subunit sigma-1 | 1.65E-79 | 99.45% | 576 |
| L2421 | JT960508.1 | small nuclear ribonucleoprotein f | 1.05E-47 | 97.45% | 528 |
| L2422 | JT946557.1 | ethylene-responsive transcription factor win1-like | 1.95E-25 | 96.75% | 510 |
| L2423 | JT949700.1 | peptide methionine sulfoxide reductase b5-like | 3.00E-83 | 89.15% | 633 |
| L2424 |  | nucleotide binding protein* | 5.00E-05 | 90.00% | 306 |
| L2425 | JT965619.1 | 40S ribosomal protein S30 | 3.95E-33 | 97.90% | 484 |
| L2426 | JR364171.1 | membrane insertase | 2.70E-28 | 74.90% | 595 |
| L2427 | JT950497.1 | HSP20-like chaperones superfamily protein isoform 1 | 8.59E-19 | 95.20% | 618 |
| L2428 | JR364574.1 |  |  |  | 601 |
| L2429 | JT947323.1 | peptide deformylase 1a | 1.41E-30 | 66.35% | 275 |
| L2430 | JT936824.1 | Photosystem I reaction center subunit XI, chloroplast precursor | 1.77E-100 | 90.20% | 602 |
| L2431 | JT915326.1 | pumilio homolog 2-like | 1.86E-22 | 100.00% | 615 |
| L2432 | JR345208.1 | probable NADH dehydrogenase | 1.21E-33 | 84.50% | 343 |
| L2433 | JT926478.1 | UDP-n-acetylglucosamine transferase subunit alg14 | 4.00E-60 | 89.00% | 616 |
| L2434 | JT954828.1 | 40S ribosomal protein S15 | 2.32E-67 | 98.50% | 604 |
| L2435 | JT967444.1 | DNA topoisomerase 2-like | 5.28E-32 | 69.15% | 590 |
| L2436 | JR363183.1 | 1d-myo-inositol 2-amino-2-deoxy-alpha-d-glucopyranoside ligase | 1.97E-30 | 86.50% | 531 |
| L2437 | JT945746.1 | nogo-b receptor | 1.77E-62 | 76.85% | 665 |
| L2438 | JT962019.1 | breast carcinoma amplified sequence | 4.50E-11 | 82.65% | 533 |
| L2439 | JT918404.1 | sphingoid long-chain bases kinase 1 | 6.53E-135 | 91.80% | 732 |
| L2440 | JT949953.1 | 60S ribosomal protein L17-1 | 1.98E-75 | 97.70% | 698 |
| L2441 | JT961435.1 | histone H4 | 2.85E-49 | 99.10% | 675 |
| L2442 | JT929251.1 | geranylgeranyl-diphosphate synthase | 1.76E-117 | 84.50% | 658 |
| L2443 | JT958479.1 | 40S ribosomal protein S24-1 | 1.47E-81 | 97.70% | 623 |
| L2444 | JR356915.1 | peptidyl-prolyl cis-trans isomerase | 3.07E-106 | 97.10% | 638 |
| L2445 | JR363324.1 | DNA-binding family protein | 1.47E-90 | 84.85% | 605 |
| L2446 | JT946559.1 | GTP-binding protein yptm2 | 6.24E-147 | 98.80% | 161 |
| L2447 | JT969277.1 |  |  |  | 497 |
| L2448 | JT936030.1 | sterile alpha motif (SAM) domain-containing protein | 4.93E-54 | 81.50% | 567 |
| L2449 | JT945223.1 | probable protein phosphatase 2c 59 | 9.13E-70 | 94.40% | 681 |
| L2450 | JT949055.1 | eukaryotic translation initiation factor 3 subunit j-like | 1.31E-25 | 91.60% | 584 |
| L2451 | JT962192.1 | nutrient reservoir | 1.73E-12 | 71.58% | 695 |
| L2452 | JR363991.1 | ecotropic viral integration site 5 protein | 5.13E-63 | 90.45% | 565 |
| L2453 | JT931488.1 | isocitrate dehydrogenase | 2.88E-100 | 97.95% | 648 |
| L2454 | JT937412.1 | glycosyl hydrolase family protein 43 isoform 2 | 1.80E-109 | 94.40% | 658 |
| L2455 | JT942827.1 | E3 ubiquitin-protein ligase rnf185-like | 1.87E-58 | 94.20% | 485 |
| L2456 | JT964507.1 | nicotinamidase 2 | 1.49E-76 | 82.50% | 570 |
| L2457 | JT917634.1 | protein IQ-DOMAIN 31-like | 1.81E-77 | 75.15% | 646 |
| L2458 | JT926028.1 | flocculation protein flo11-like isoform x3 | 7.91E-24 | 58.75% | 653 |
| L2459 | JT917107.1 | f-box protein skip14-like | 5.49E-13 | 76.90% | 596 |
| L2460 | JT977438.1 | 2-alkenal reductase (NADP(+)-dependent) | 5.28E-30 | 87.80% | 316 |
| L2461 | JT917315.1 | Ubiquitin carboxyl-terminal hydrolase 23 | 2.03E-33 | 79.60% | 518 |
| L2462 | JT932835.1 | ketol-acid reductoisomerase | 2.79E-52 | 94.60% | 569 |
| L2463 | JR364032.1 | transcription elongation factor 1 homolog | 1.75E-45 | 95.80% | 549 |
| L2464 | JR345442.1 | 26S protease regulatory subunit 6b homolog | 3.54E-18 | 71.35% | 238 |
| L2465 | JT947850.1 | heme-binding protein 2-like | 2.32E-84 | 82.65% | 609 |
| L2466 | JT925782.1 | protein transport protein sec61 subunit alpha-like | 1.04E-96 | 99.30% | 655 |
| L2467 | JT927101.1 | o-acyltransferase wsd1-like | 1.25E-88 | 65.30% | 683 |
| L2468 | JT915054.1 | DNA-directed RNA polymerase II subunit rpb2 | 1.85E-36 | 92.10% | 631 |
| L2469 | JT968000.1 | LOB domain-containing protein 27-like | 5.76E-66 | 86.45% | 508 |
| L2470 | JT960877.1 | hydroxyproline-rich glycoprotein (POPTR_0008s12810g)* | 2.00E-63 | 75.00% | 570 |
| L2471 | JT959931.1 | formin-like protein 4 | 2.93E-25 | 66.05% | 486 |
| L2472 | JT915237.1 | Ubiquitin carboxyl-terminal hydrolase-related protein isoform 1 | 1.79E-19 | 78.30% | 496 |
| L2473 | JT917566.1 | endoplasmic reticulum metallopeptidase 1 | 1.67E-36 | 67.95% | 556 |
| L2474 | JT926439.1 | zinc-dependent activator protein- isoform 1 | 5.05E-91 | 64.00% | 618 |
| L2475 | JT956498.1 | actin-depolymerizing factor 2-like | 2.16E-95 | 97.65% | 647 |
| L2476 | JT945921.1 | conserved hypothetical protein | 6.71E-30 | 72.05% | 601 |
| L2477 | JT945193.1 | dehydrin protein | 5.00E-33 | 99.00% | 220 |
| L2478 | JT961835.1 | hypothetical protein POPTR_0013s12040g | 1.11E-39 | 84.75% | 583 |
| L2479 | JR345822.1 | 26S proteasome regulatory subunit family protein | 2.14E-38 | 97.75% | 642 |
| L2480 | JT916400.1 | pleckstrin homology domain-containing family protein | 4.06E-30 | 96.60% | 554 |
| L2481 | JT945328.1 | pyridine nucleotide-disulfide oxidoreductase family protein | 1.38E-26 | 82.95% | 436 |
| L2482 | JT950951.1 | two-component response regulator arr5-like | 3.58E-99 | 87.25% | 702 |
| L2483 | JT936993.1 | auxin-repressed 12.5 kDa | 3.50E-38 | 90.20% | 589 |
| L2484 | JT945391.1 | broad-range acid phosphatase det1-like | 6.18E-87 | 88.40% | 435 |
| L2485 | JR349420.1 | protein phosphatase 2c 16-like | 2.75E-97 | 82.60% | 503 |
| L2486 | JT936907.1 | ultraviolet-B receptor uvr8 isoform x6 | 4.10E-68 | 77.85% | 508 |
| L2487 | JR366057.1 | AP2 domain transcription factor family protein | 2.31E-104 | 70.30% | 531 |
| L2488 | JT963563.1 | scarecrow-like transcription factor isoform 1 | 6.67E-10 | 48.60% | 487 |
| L2489 | JT952698.1 | 28 kDa heat- and acid-stable phosphoprotein* | 4.00E-46 | 86.00% | 488 |
| L2490 | JR362240.1 | MYB domain protein | 4.83E-34 | 58.29% | 602 |
| L2491 | JT944286.1 | chloroplast-targeted copper chaperone | 1.22E-52 | 56.45% | 643 |
| L2492 | JR363734.1 | RNA-binding family protein isoform 2 | 4.77E-56 | 94.80% | 641 |
| L2493 | JT926809.1 | NAD -binding rossmann-fold superfamily protein isoform 2 | 1.57E-72 | 86.60% | 684 |
| L2494 | JT921969.1 | probable indole-3-pyruvate monooxygenase yucca10 | 6.40E-18 | 83.10% | 407 |
| L2495 | JT956728.1 | 60S ribosomal protein L4 | 1.20E-40 | 90.35% | 572 |
| L2496 | JT938724.1 | hydroxycinnamoyl CoA shikimate/quinate hydroxycinnamoyltransferase (HCQ3) | 1.07E-61 | 90.70% | 633 |
| L2497 | JT935376.1 | AP2/ERF super family protein | 5.39E-61 | 77.35% | 464 |
| L2498 | JT950149.1 | hypothetical protein JCGZ_12065 | 4.80E-48 | 92.35% | 679 |
| L2499 | JT953622.1 | peptidyl-prolyl cis-trans isomerase cyp19-4-like | 2.19E-111 | 97.10% | 673 |
| L2500 | JT925299.1 | act-like protein tyrosine kinase family protein isoform 2 | 3.31E-74 | 72.65% | 568 |
| L2501 | JT923482.1 | adenosylhomocysteinase 1 -like protein | 3.94E-17 | 95.40% | 511 |
| L2502 | JR365407.1 | RNA polymerase II transcriptional coactivator kelp-like | 5.47E-90 | 78.60% | 666 |
| L2503 | JR362789.1 | 40S ribosomal protein S29 | 7.27E-34 | 96.25% | 527 |
| L2504 | JT923975.1 | pre-mRNA-splicing factor slu7-like | 1.29E-28 | 91.45% | 672 |
| L2505 | JR345047.1 | RNA recognition motif-containing protein | 4.79E-34 | 74.50% | 609 |
| L2506 | JR364014.1 | mitochondrial import receptor subunit TOM9-2-like | 5.34E-44 | 84.00% | 564 |
| L2507 | JR363606.1 |  |  |  | 346 |
| L2508 | JT956952.1 | HMG1/2-like protein | 1.21E-46 | 78.85% | 686 |
| L2509 | JT972725.1 |  |  |  | 219 |
| L2510 | JT929578.1 | Serine protease 45 | 2.23E-61 | 86.30% | 509 |
| L2511 | JT944484.1 | PRA1 family protein h | 1.02E-65 | 85.10% | 611 |
| L2512 | JT929052.1 | Serine threonine-protein kinase at5g01020 | 6.19E-93 | 90.35% | 636 |
| L2513 | JR347577.1 | hypothetical protein JCGZ_26382 | 4.13E-07 | 83.00% | 545 |
| L2514 | JT929758.1 | cytochrome P450 86b1-like | 4.37E-96 | 84.65% | 673 |
| L2515 | JT944798.1 | hypothetical protein L484_005901 | 4.49E-09 | 82.70% | 508 |
| L2516 | JT933124.1 | elongation factor 1-alpha-like | 3.97E-154 | 99.00% | 670 |
| L2517 | JT960768.1 | RING-box protein (RBX1a) | 3.42E-65 | 91.45% | 592 |
| L2518 | JT938297.1 | transcription initiation factor tfIId subunit 13 | 5.25E-80 | 91.50% | 676 |
| L2519 | JT939900.1 | prohibitin mitochondrial-like | 2.48E-69 | 94.10% | 661 |
| L2520 | JT920312.1 | Ubiquinone biosynthesis monooxygenase coq6 isoform x1 | 4.64E-111 | 87.95% | 589 |
| L2521 | JR347374.1 | Ribonucleoside-diphosphate reductase subunit beta | 2.69E-32 | 73.95% | 222 |
| L2522 | JR355065.1 |  |  |  | 184 |
| L2523 | JT925724.1 | insulinase (peptidase family m16) protein isoform 1 | 4.56E-10 | 89.05% | 284 |
| L2524 |  | chaperone protein 1-like | 2.88E-33 | 95.85% | 505 |
| L2525 | JR347852.1 | hypothetical protein JCGZ_14682 | 1.38E-57 | 69.90% | 627 |
| L2526 | JT919075.1 | nucleobase-ascorbate transporter 11 isoform x1 | 1.60E-67 | 64.45% | 653 |
| L2527 | JT948191.1 | late embryogenesis abundant hydroxyproline-rich glycoprotein | 9.32E-26 | 77.60% | 485 |
| L2528 | JR366306.1 | caffeic acid methyltransferase | 6.30E-90 | 85.95% | 486 |
| L2529 | JT947084.1 | sulfate adenylyltransferase subunit 2 | 2.86E-68 | 80.80% | 692 |
| L2530 | JR349804.1 | acidic endochitinase | 1.90E-57 | 91.70% | 486 |
| L2531 | JT937656.1 | farnesyl diphosphate synthase | 3.00E-145 | 99.00% | 606 |
| L2532 | JT954607.1 | Serine protease chloroplastic-like | 5.47E-105 | 92.45% | 284 |
| L2533 | JR349563.1 | peptide deformylase 1a | 2.63E-163 | 78.80% | 286 |
| L2534 | JT947980.1 | pectate lyase | 9.91E-29 | 94.10% | 168 |
| L2535 | JR363194.1 | axonemal dynein light | 3.18E-27 | 56.20% | 459 |
| L2536 | JT942644.1 | uncharacterized loc101210357 | 7.37E-38 | 88.35% | 368 |
| L2537 | JR360856.1 | conserved hypothetical protein* | 1.00E-25 | 77.00% | 329 |
| L2538 | JT928495.1 | monodehydroascorbate cytoplasmic isoform 2 | 3.12E-93 | 86.25% | 517 |
| L2539 | JT960460.1 | Xyloglucan galactosyltransferase KATAMARI1* | 1.00E-06 | 78.00% | 448 |
| L2540 | JT936257.1 | tryptophanyl-tRNA synthetase | 4.29E-17 | 95.25% | 233 |
| L2541 | JT966313.1 | cysteine-rich repeat secretory protein 12-like | 6.22E-47 | 85.85% | 283 |
| L2542 | JT930112.1 | Serine arginine repetitive matrix protein 2 isoform 2 | 1.47E-18 | 85.30% | 422 |
| L2543 | JT969377.1 |  |  |  | 468 |
| L2544 | JT933044.1 | isopentenyl pyrophosphate isomerase | 1.00E-116 | 99.00% | 569 |
| L2545 | JT921993.1 | 29 kDa ribonucleoprotein, chloroplast like | 3.88E-53 | 76.70% | 433 |
| L2546 | JT951476.1 | acyl carrier protein chloroplastic-like | 1.75E-48 | 80.30% | 424 |
| L2547 | JT957705.1 | hypothetical protein JCGZ_19481 | 1.68E-93 | 82.80% | 688 |
| L2548 | JR364926.1 | small nuclear ribonucleoprotein sm d1-like | 8.66E-59 | 99.35% | 611 |
| L2549 | JT932578.1 | heat shock protein 90-1 | 2.73E-129 | 97.05% | 640 |
| L2550 | JT927385.1 | chaperone protein DNAj 10-like | 2.18E-24 | 69.75% | 359 |
| L2551 | JR349172.1 | MYBR domain class transcription factor (MYBR4) | 7.57E-31 | 67.00% | 648 |
| L2552 | JT943442.1 | hypothetical protein POPTR_0001s16710g | 1.31E-40 | 83.60% | 340 |
| L2553 | JT964363.1 | transmembrane protein 97 | 2.44E-88 | 82.50% | 581 |
| L2554 | JT945784.1 | hypothetical protein POPTR_0007s13740g | 1.16E-90 | 77.10% | 614 |
| L2555 | JR365058.1 | phospholipid hydroperoxide glutathione peroxidase | 1.09E-63 | 75.50% | 699 |
| L2556 | JT955363.1 | histone H2A family protein | 8.10E-73 | 91.15% | 546 |
| L2557 | JT951143.1 | conserved hypothetical protein | 4.58E-10 | 75.92% | 507 |
| L2558 | JT922991.1 | phagocytic receptor 1b | 3.96E-99 | 93.15% | 578 |
| L2559 | JR365231.1 | cytochrome b5 isoform Cb5-D | 1.96E-68 | 85.75% | 593 |
| L2560 | JT920993.1 | peptidyl-prolyl cis-trans isomerase pin1 | 3.79E-74 | 95.45% | 580 |
| L2561 | JT961782.1 | zinc finger protein* | 8.00E-23 | 74.00% | 468 |
| L2562 | JT962689.1 | hypothetical protein JCGZ_04010 | 1.63E-23 | 62.00% | 319 |
| L2563 | JR363181.1 | zinc finger protein 706-like | 3.81E-28 | 90.05% | 414 |
| L2564 | JT934417.1 | conserved hypothetical protein | 1.14E-05 | 61.00% | 636 |
| L2565 | JT969039.1 | cytochrome c oxidase subunit 5c | 9.61E-28 | 90.60% | 487 |
| L2566 | JT929852.1 | ethylene response sensor 1 | 7.72E-15 | 93.80% | 136 |
| L2567 | JR347859.1 | DNA-binding protein escarola | 2.20E-75 | 80.20% | 109 |
| L2568 | JR346807.1 | stress enhanced protein chloroplastic | 2.39E-69 | 89.35% | 621 |
| L2569 | JR363180.1 |  |  |  | 316 |
| L2570 | JT958979.1 | heavy metal transport detoxification superfamily isoform 1 | 7.24E-17 | 87.70% | 666 |
| L2571 | JT942162.1 | general transcription factor IIf subunit 1 | 9.52E-29 | 58.55% | 616 |
| L2572 | JR348403.1 | sequence-specific DNA binding transcription factors isoform 2 | 2.84E-83 | 75.80% | 641 |
| L2573 | JT976044.1 |  |  |  | 491 |
| L2574 | JT976682.1 | vesicle-associated membrane protein 726 | 1.21E-31 | 94.30% | 406 |
| L2575 | JT964312.1 | methyltransferase-like protein | 7.44E-27 | 73.20% | 504 |
| L2576 | JT939584.1 | hypothetical protein JCGZ_23391 | 1.11E-13 | 89.33% | 610 |
| L2577 | JT944161.1 | protein lsm12 homolog | 9.69E-95 | 90.20% | 502 |
| L2578 | JT953296.1 | coiled-coil domain-containing protein 174-like | 2.03E-48 | 77.60% | 604 |
| L2579 | JT927604.1 | cilia- and flagella-associated protein 20 | 4.27E-49 | 98.35% | 528 |
| L2580 | JR362413.1 | stress-induced hydrophobic peptide | 1.64E-19 | 93.80% | 604 |
| L2581 | JT957065.1 | homeobox-leucine zipper protein ATHB-6-like (LOC105130321), transcript variant X2* | 7.00E-37 | 77.00% | 507 |
| L2582 | JR345748.1 | polypeptide n-acetylgalactosaminyltransferase 35a | 1.20E-25 | 92.15% | 538 |
| L2583 | JT920660.1 | phosphatidylcholine transfer protein | 4.52E-08 | 82.40% | 204 |
| L2584 | JT931896.1 | probable receptor-like protein kinase at5g15080-like | 1.94E-77 | 90.30% | 548 |
| L2585 | JT975586.1 |  |  |  | 479 |
| L2586 | JT966582.1 | uncharacterized loc101215930 | 5.25E-47 | 78.70% | 607 |
| L2587 | JR365186.1 | casp-like protein | 1.70E-92 | 82.30% | 616 |
| L2588 | JT954786.1 | hypothetical protein JCGZ_18648 | 4.26E-81 | 56.90% | 624 |
| L2589 | JT957865.1 | cst complex subunit ten1-like | 1.30E-68 | 87.25% | 641 |
| L2590 | JT958893.1 | G patch domain-containing protein 8-like | 2.79E-21 | 62.00% | 630 |
| L2591 | JT926352.1 | calcium-binding protein 39-like | 3.82E-20 | 93.90% | 598 |
| L2592 |  | hypothetical protein* | 2.00E-05 | 64.00% | 504 |
| L2593 | JT939454.1 | 40S ribosomal protein S25-2 | 1.17E-43 | 98.65% | 565 |
| L2594 | JT962047.1 | excitatory amino acid transporter 1 | 1.47E-49 | 94.85% | 494 |
| L2595 | JT916665.1 | auxin response factor 8-like | 2.88E-12 | 86.00% | 499 |
| L2596 | JT940467.1 | U1 small nuclear ribonucleoprotein 70 kDa-like | 6.01E-108 | 90.80% | 706 |
| L2597 | JR362084.1 | conserved hypothetical protein* | 4.00E-40 | 80.00% | 506 |
| L2598 | JR365086.1 | heat shock factor protein hsf24 | 2.55E-95 | 84.25% | 636 |
| L2599 |  | pentatricopeptide repeat-containing protein at1g19720 | 8.34E-40 | 71.10% | 642 |
| L2600 | JR349663.1 | E3 ubiquitin-protein ligase ring 1-like | 3.71E-142 | 73.85% | 422 |
| L2601 | JT963434.1 | phytosulfokines 4-like | 1.13E-10 | 62.45% | 557 |
| L2602 | JT950698.1 | LOB domain-containing protein 1 | 7.31E-92 | 90.20% | 697 |
| L2603 | JT928422.1 | probable E3 ubiquitin-protein ligase xbos32 | 9.96E-69 | 76.65% | 534 |
| L2604 | JT942319.1 | probable tRNA n6-adenosine threonylcarbamoyltransferase | 4.37E-58 | 96.70% | 480 |
| L2605 | JR364721.1 | hypothetical protein JCGZ_16048 | 1.40E-44 | 96.65% | 554 |
| L2606 | JT944226.1 | myosin-3-like isoform x2 | 2.79E-08 | 88.00% | 140 |
| L2607 | JT956575.1 | PREDICTED: uncharacterized protein LOC102631260 | 2.13E-08 | 74.40% | 447 |
| L2608 | JT943071.1 | Ubiquitin-associated domain-containing family protein | 1.83E-110 | 86.05% | 696 |
| L2609 | JT947585.1 | 60S ribosomal protein L10 | 2.41E-99 | 96.95% | 647 |
| L2610 | JT960690.1 | 40S ribosomal protein S12 | 5.51E-57 | 85.15% | 531 |
| L2611 | JT954113.1 |  |  |  | 670 |
| L2612 | JT935191.1 | mediator-associated protein 1-like | 3.58E-86 | 83.20% | 643 |
| L2613 | JT955904.1 | nucleic acid-binding | 1.05E-86 | 89.40% | 591 |
| L2614 | JT963983.1 | hypothetical protein JCGZ_17427 | 2.41E-38 | 70.90% | 646 |
| L2615 | JR366931.1 | polyadenylate-binding protein 2 | 2.52E-55 | 89.00% | 688 |
| L2616 | JT972363.1 | probable protein phosphatase 2c 60 | 1.40E-28 | 93.05% | 657 |
| L2617 | JR348166.1 | glutamate receptor 2 | 2.81E-10 | 86.00% | 517 |
| L2618 | JR345472.1 | Serine threonine protein phosphatase 2a 55 kDa regulatory subunit b beta isoform-like isoform x1 | 1.32E-17 | 96.10% | 695 |
| L2619 | JR345565.1 | 60S ribosomal protein L22-2 | 1.03E-49 | 96.10% | 661 |
| L2620 | JR366819.1 | 12-oxophytodienoate reductase 3 | 3.15E-135 | 91.85% | 720 |
| L2621 | JT928783.1 | pti1-like tyrosine-protein kinase at3g15890-like | 7.47E-22 | 88.85% | 655 |
| L2622 | JT951488.1 | PREDICTED: uncharacterized protein LOC104596457 | 1.94E-19 | 78.67% | 552 |
| L2623 | JT918732.1 | zinc finger CCCH domain-containing protein 66-like | 2.30E-108 | 81.50% | 680 |
| L2624 | JT952077.1 | 60S ribosomal protein L9 | 4.91E-79 | 98.40% | 605 |
| L2625 |  | histone H4 | 1.14E-15 | 100.00% | 269 |
| L2626 | JT944602.1 | 60S ribosomal protein L11 | 3.61E-110 | 98.00% | 344 |
| L2627 | JT939403.1 | cw-type zinc finger-like protein | 7.14E-88 | 65.80% | 689 |
| L2628 |  | zinc-finger homeodomain protein 11-like | 1.64E-61 | 67.65% | 640 |
| L2629 | JT942848.1 | Serine-threonine protein plant-type | 2.12E-67 | 94.20% | 587 |
| L2630 | JT949146.1 | golgi snap receptor complex member 1-2 | 1.65E-96 | 95.60% | 662 |
| L2631 | JR345469.1 | brain acid soluble protein 1 homolog | 8.44E-49 | 82.50% | 504 |
| L2632 | JT924050.1 | cytochrome P450 | 3.51E-100 | 87.65% | 596 |
| L2633 | JT940443.1 | Radiation sensitive | 7.84E-88 | 68.55% | 736 |
| L2634 | JT935799.1 | Ribosomal protein L4/L1 family isoform 1 | 5.18E-86 | 76.90% | 704 |
| L2635 | JT941282.1 | Ribosomal RNA-processing protein 8-like | 3.38E-115 | 91.50% | 577 |
| L2636 | JT935595.1 | hypothetical protein JCGZ_02187 | 3.12E-45 | 64.20% | 629 |
| L2637 | JT942536.1 | adaptin ear-binding coat-associated | 3.42E-55 | 82.45% | 666 |
| L2638 | JT929085.1 | eukaryotic initiation factor 4a-1 | 4.64E-47 | 98.25% | 573 |
| L2639 | JR366847.1 | IAA-amino acid hydrolase ilr1-like 5 | 1.68E-101 | 87.45% | 692 |
| L2640 | JR349584.1 | 50S ribosomal protein L25 | 1.81E-56 | 85.65% | 614 |
| L2641 | JR363256.1 | zinc finger protein 6-like | 1.04E-55 | 69.80% | 568 |
| L2642 | JT958746.1 | hypothetical protein JCGZ_10108 | 8.13E-39 | 96.15% | 457 |
| L2643 | JT949730.1 | 60S ribosomal protein L10a-2 | 9.10E-109 | 95.20% | 713 |
| L2644 | JT920788.1 | kelch domain-containing protein 4 | 8.56E-99 | 97.45% | 671 |
| L2645 | JT960496.1 | protein canopy homolog 1-like | 2.72E-09 | 84.11% | 525 |
| L2646 | JR365470.1 | thioredoxin h-type | 3.19E-78 | 90.85% | 633 |
| L2647 | JT943376.1 | ATP-NAD kinase family protein | 5.59E-27 | 74.25% | 648 |
| L2648 | JT962967.1 | hypothetical protein JCGZ_10888 | 6.77E-06 | 62.00% | 573 |
| L2649 | JT959274.1 | profilin 5 | 3.50E-77 | 96.05% | 622 |
| L2650 | JT936340.1 | pyridine nucleotide-disulfide oxidoreductase family protein | 2.13E-07 | 84.15% | 109 |
| L2651 | JT920590.1 | Vacuolar sorting receptor isoform 1 | 4.64E-145 | 94.05% | 675 |
| L2652 | JT952928.1 | oxidoreductase transition metal ion-binding protein | 1.90E-51 | 78.15% | 589 |
| L2653 | JT945486.1 | uncharacterized loc101212458 | 2.42E-64 | 72.90% | 565 |
| L2654 | JT961158.1 | dentin sialophosphoprotein-like | 2.11E-21 | 57.75% | 601 |
| L2655 | JR366120.1 | proactivator polypeptide-like 1 | 8.69E-81 | 83.60% | 605 |
| L2656 | JR349421.1 | NADH dehydrogenase | 5.93E-60 | 89.90% | 625 |
| L2657 | JT961337.1 | small acidic protein 1 | 6.12E-20 | 88.84% | 374 |
| L2658 | JT937027.1 | malate dehydrogenase | 5.57E-75 | 95.65% | 637 |
| L2659 | JT959907.1 | tbp-associated factor 7 isoform 1 | 3.54E-46 | 92.70% | 516 |
| L2660 | JT937576.1 | peroxisomal membrane protein 13-like | 2.49E-77 | 87.20% | 571 |
| L2661 |  | potassium channel KAT3 | 1.80E-22 | 87.10% | 497 |
| L2662 |  | eukaryotic initiation factor 4a-10 | 1.35E-76 | 97.20% | 524 |
| L2663 | JR366622.1 | stromal 70 kDa heat shock-related protein, chloroplastic-like | 3.10E-30 | 84.95% | 420 |
| L2664 | JT919453.1 | ABC transporter F family member 1 | 1.72E-43 | 97.70% | 594 |
| L2665 | JT965500.1 | was wasl-interacting protein family member isoform 2 | 3.91E-47 | 68.65% | 558 |
| L2666 | JT927498.1 | low psII accumulation 3 isoform partial | 6.00E-23 | 91.10% | 436 |
| L2667 | JT937862.1 | sphingolipid delta 4 desaturase/C-4 hydroxylase protein des2 | 2.69E-97 | 92.15% | 717 |
| L2668 | JT932932.1 | elongation factor 1-alpha | 4.55E-25 | 90.20% | 549 |
| L2669 | JT942101.1 | embryo defective | 4.71E-79 | 66.70% | 657 |
| L2670 | JT944491.1 | stromal cell-derived factor 2-like protein precursor | 4.39E-110 | 95.40% | 600 |
| L2671 | JT945400.1 | 40S ribosomal protein S6 isoform x1 | 3.26E-74 | 96.90% | 665 |
| L2672 | JT929525.1 | G-protein coupled receptor 1-like | 1.42E-74 | 89.30% | 637 |
| L2673 | JT965872.1 | benzoyl- reductase subunit c | 1.14E-34 | 89.35% | 570 |
| L2674 | JT972051.1 | seed maturation protein pm36 | 7.51E-53 | 82.25% | 482 |
| L2675 | JT917491.1 | bromodomain-containing protein | 9.33E-16 | 88.10% | 151 |
| L2676 | JR365757.1 | nucleolar protein 16 | 7.56E-112 | 83.70% | 674 |
| L2677 | JR359565.1 | 23.6 kDa heat shock, mitochondrial -like protein | 2.55E-77 | 79.20% | 628 |
| L2678 |  | hypothetical protein* | 3.00E-09 | 92.00% | 425 |
| L2679 | JT957499.1 | PREDICTED: uncharacterized protein LOC105110611 | 1.21E-54 | 84.75% | 524 |
| L2680 | JT958786.1 | mitochondrial acidic protein mitochondrial | 2.48E-37 | 89.70% | 494 |
| L2681 | JT960755.1 | elmo domain-containing protein a | 1.17E-29 | 74.70% | 649 |
| L2682 | JT957840.1 | f-box/lrr-repeat protein at3g26922-like | 1.05E-88 | 71.55% | 681 |
| L2683 | JT954539.1 | universal stress protein a-like protein | 1.91E-50 | 91.80% | 439 |
| L2684 | JT930356.1 | transmembrane protein 53 | 1.91E-113 | 80.20% | 657 |
| L2685 | JR364235.1 | LIGULELESS1 protein | 1.49E-75 | 73.15% | 665 |
| L2686 | JT971589.1 | vesicle-associated membrane protein 714-like | 7.48E-36 | 97.10% | 618 |
| L2687 | JT920567.1 | flocculation protein flo11-like | 1.96E-24 | 72.60% | 553 |
| L2688 | JR347353.1 | uncharacterized loc101222625 | 3.23E-76 | 91.45% | 676 |
| L2689 | JT919768.1 | GTP-binding protein | 1.36E-05 | 65.00% | 671 |
| L2690 | JR365540.1 | high mobility group beta isoform 1 | 1.74E-51 | 88.80% | 607 |
| L2691 | JT928560.1 | C2 domain-containing family protein | 1.08E-17 | 81.65% | 454 |
| L2692 | JT947635.1 | ormdl family protein | 1.95E-24 | 97.95% | 495 |
| L2693 | JT942370.1 | Vacuolar protein sorting-associated protein 32 homolog 2 | 2.01E-71 | 94.10% | 612 |
| L2694 | JT943432.1 | disease resistance protein RGA2-like | 4.69E-36 | 62.50% | 446 |
| L2695 | JT969509.1 | adenine phosphoribosyltransferase | 2.87E-91 | 77.20% | 708 |
| L2696 | JT956265.1 | hypothetical protein CICLE_v10010135mg | 1.68E-05 | 93.75% | 545 |
| L2697 | JT924844.1 | WD-40 repeat protein msi4 | 1.31E-49 | 83.10% | 628 |
| L2698 | JT968896.1 |  |  |  | 575 |
| L2699 | JT954671.1 | H/ACA ribonucleoprotein complex subunit 1-like protein 1 | 4.74E-62 | 96.00% | 632 |
| L2700 | JT971248.1 | hypothetical protein JCGZ_06092 | 3.24E-10 | 79.00% | 626 |
| L2701 | JT950589.1 | U-box domain-containing protein 8 | 1.45E-70 | 82.15% | 672 |
| L2702 | JT933067.1 | proline dehydrogenase mitochondrial | 1.42E-36 | 92.20% | 401 |
| L2703 | JT923367.1 | anthranilate phosphoribosyltransferase-like | 1.03E-62 | 92.90% | 668 |
| L2704 | JT919614.1 | membrane protein of ER body-like protein | 5.22E-08 | 47.20% | 637 |
| L2705 | JT948759.1 | peptide methionine sulfoxide reductase b5-like | 2.25E-90 | 75.25% | 569 |
| L2706 | JT949189.1 | dirigent protein 17-like | 2.67E-10 | 68.47% | 560 |
| L2707 | JT940045.1 | glycine-rich RNA-binding protein mitochondrial | 2.58E-61 | 84.40% | 584 |
| L2708 | JR349444.1 | DNA-directed RNA polymerases and III subunit rpabc5 | 3.64E-45 | 97.15% | 486 |
| L2709 | JR348668.1 | ethylene-insensitive 3f isoform 1 | 4.08E-26 | 58.55% | 474 |
| L2710 | JT950097.1 | protein odr-4 homolog | 2.62E-14 | 67.55% | 658 |
| L2711 | JT926360.1 | phosphoglycerate kinase | 3.03E-120 | 97.00% | 562 |
| L2712 | JT963599.1 | mlp-like protein 329 | 2.28E-59 | 72.75% | 564 |
| L2713 | JT969170.1 | dolichyl-diphosphooligosaccharide--protein glycosyltransferase subunit 4a | 4.05E-19 | 97.20% | 378 |
| L2714 | JT935318.1 | U-box domain-containing protein 4-like | 8.90E-53 | 93.15% | 397 |
| L2715 |  | fiber protein fb34 | 5.63E-78 | 92.50% | 624 |
| L2716 | JT952000.1 | conserved hypothetical protein | 1.29E-35 | 85.70% | 624 |
| L2717 | JT939758.1 | Ubiquitin receptor rad23b-like | 1.14E-70 | 80.25% | 581 |
| L2718 | JT922767.1 | PREDICTED: uncharacterized protein LOC103963856 | 1.80E-10 | 68.50% | 608 |
| L2719 | JT968336.1 | mitochondrial import receptor subunit TOM7-1-like | 2.45E-24 | 92.90% | 506 |
| L2720 | JT945306.1 | 3-isopropylmalate dehydratase small subunit 3-like | 6.60E-94 | 84.80% | 676 |
| L2721 |  | SAUR family protein | 2.51E-39 | 79.35% | 573 |
| L2722 | JT963368.1 | 18.5 kDa class i heat shock | 8.37E-32 | 64.35% | 358 |
| L2723 | JT951227.1 | AP2 domain transcription factor family protein | 5.62E-22 | 80.85% | 624 |
| L2724 | JT942002.1 | map7 domain-containing protein 1-like isoform x4 | 7.57E-48 | 73.15% | 642 |
| L2725 | JR346952.1 |  |  |  | 539 |
| L2726 | JT940583.1 | mitochondrial outer membrane protein porin of 36 kDa | 1.72E-64 | 96.90% | 638 |
| L2727 | JT950775.1 | 26S proteasome non-ATPase regulatory | 2.15E-111 | 79.80% | 628 |
| L2728 | JT934477.1 | acetyl-CoA C-acetyltransferas | 1.40E-81 | 95.85% | 662 |
| L2729 | JT978507.1 | V-type proton ATPase subunit b2 | 2.59E-28 | 92.70% | 238 |
| L2730 | JT962666.1 | protein early responsive to dehydration 15-like | 2.80E-15 | 63.80% | 606 |
| L2731 | JT957122.1 |  |  |  | 583 |
| L2732 | JT952037.1 | swib complex baf60b domain-containing family protein | 3.41E-58 | 79.80% | 627 |
| L2733 | JR366824.1 | DNA-binding protein escarola | 3.73E-22 | 68.00% | 619 |
| L2734 | JT954040.1 | ap-2 complex subunit sigma | 2.31E-94 | 96.25% | 484 |
| L2735 | JT928745.1 | Ribosomal protein L16 | 1.67E-81 | 98.70% | 623 |
| L2736 | JT952106.1 | upf0133 protein | 1.14E-110 | 82.55% | 583 |
| L2737 | JT960059.1 | 40S ribosomal protein S23 | 2.72E-97 | 99.35% | 582 |
| L2738 | JR365277.1 | multiprotein-bridging factor 1a | 8.19E-71 | 94.35% | 613 |
| L2739 | JR349797.1 | cyclin-dependent protein kinase | 2.99E-105 | 89.65% | 589 |
| L2740 |  | CAP-Gly domain-containing linker protein 1 | 4.34E-86 | 80.15% | 586 |
| L2741 | JT919583.1 | 26S ribosomal RNA gene | 8.94E-114 | 84.70% | 656 |
| L2742 | JT925145.1 | yth domain-containing protein | 7.44E-65 | 67.60% | 633 |
| L2743 | JT919907.1 | coronatine-insensitive protein 1-like | 1.11E-74 | 86.45% | 576 |
| L2744 | JR365447.1 | pleckstrin homology domain-containing protein 1-like | 7.20E-92 | 87.05% | 702 |
| L2745 | JR364002.1 | hypothetical protein JCGZ_06884 | 6.63E-35 | 87.10% | 486 |
| L2746 | JT957971.1 | BET1P/SFT1P-like protein 14A isoform 1 | 3.71E-74 | 94.45% | 684 |
| L2747 | JT937260.1 | dhha1 domain protein | 6.49E-125 | 81.05% | 666 |
| L2748 | JT939055.1 | U6 snrna-associated sm-like protein lsm1 | 6.94E-67 | 97.65% | 652 |
| L2749 | JT917839.1 | protein EIN4 | 3.76E-11 | 88.05% | 496 |
| L2750 |  | Auxin response factor* | 1.00E-25 | 84.00% | 337 |
| L2751 | JT914542.1 | transcription elongation factor spt6 | 2.36E-24 | 88.20% | 665 |
| L2752 | JT938017.1 | enhancer of mRNA-decapping protein 4-like | 4.65E-35 | 63.25% | 485 |
| L2753 | JT952968.1 | eg964 | 2.77E-60 | 92.80% | 543 |
| L2754 | JR360080.1 | gamma-glutamylcyclotransferase at3g02910 | 5.10E-58 | 81.85% | 454 |
| L2755 | JR366235.1 | RNA recognition motif-containing family protein | 1.58E-61 | 77.30% | 630 |
| L2756 | JT915831.1 | protein transport protein sec31a-like | 1.08E-14 | 96.60% | 349 |
| L2757 |  | tubulin beta-1 chain | 8.68E-43 | 98.10% | 251 |
| L2758 | JT941609.1 | floral homeotic protein apetala 2 | 1.89E-21 | 65.90% | 666 |
| L2759 | JR349544.1 | conserved hypothetical protein 12 | 6.26E-77 | 78.10% | 590 |
| L2760 | JT935810.1 | hypothetical protein JCGZ_18332 | 1.00E-51 | 89.00% | 662 |
| L2761 | JT933177.1 | Serine-threonine kinase receptor-associated protein | 1.36E-75 | 97.35% | 683 |
| L2762 | JT976255.1 | actin cytoskeleton-regulatory complex protein pan-1-like | 1.38E-110 | 80.25% | 626 |
| L2763 | JT945393.1 | structural constituent of ribosome | 7.72E-11 | 76.33% | 260 |
| L2764 | JT946238.1 | E3 ubiquitin-protein ligase rha2b | 3.89E-61 | 82.10% | 632 |
| L2765 | JR364734.1 | mitochondrial import receptor subunit TOM9-2-like | 1.51E-38 | 81.30% | 256 |
| L2766 | JT940643.1 | beta-1,3-glucanase (HGN1) | 4.62E-131 | 94.80% | 660 |
| L2767 | JT918613.1 | dynamin-related protein 3a-like | 3.17E-41 | 89.15% | 646 |
| L2768 | JT919210.1 | poly-a binding protein | 1.11E-15 | 84.30% | 638 |
| L2769 | JT935682.1 | alpha-soluble nsf attachment protein | 4.75E-133 | 91.45% | 626 |
| L2770 | JT918056.1 | probable linoleate 9s-lipoxygenase 5 | 1.27E-30 | 68.80% | 556 |
| L2771 | JT915676.1 | protein smg7 | 3.01E-119 | 86.35% | 669 |
| L2772 | JT967235.1 | conserved hypothetical protein | 4.71E-13 | 73.88% | 587 |
| L2773 | JR365113.1 | gata zinc finger domain-containing protein isoform 2 | 4.53E-55 | 76.35% | 463 |
| L2774 | JT962082.1 | 60S ribosomal protein L11 | 3.22E-85 | 97.70% | 299 |
| L2775 | JT959683.1 | thioredoxin-like protein cxxs1 | 2.96E-45 | 59.15% | 404 |
| L2776 | JT935675.1 | 14-3-3-like protein a | 3.98E-56 | 97.75% | 505 |
| L2777 | JT960141.1 | ASR-like protein 1 | 7.45E-07 | 85.00% | 555 |
| L2778 | JT976265.1 |  |  |  | 612 |
| L2779 | JT943093.1 | trafficking protein particle complex subunit 5 | 3.67E-72 | 98.50% | 695 |
| L2780 | JT944952.1 | transmembrane protein 208 homolog | 4.11E-87 | 90.45% | 690 |
| L2781 | JR364566.1 | adenine nucleotide alpha hydrolases-like protein | 2.59E-52 | 83.20% | 382 |
| L2782 | JT935061.1 | disease resistance protein RPP8 | 5.95E-55 | 70.50% | 717 |
| L2783 | JT926562.1 | 26S protease regulatory subunit | 3.35E-94 | 69.05% | 674 |
| L2784 | JT940134.1 | dcd domain protein isoform 1 | 4.35E-77 | 96.15% | 698 |
| L2785 | JT945027.1 | U3 small nucleolar RNA-associated protein 11 | 1.21E-43 | 85.15% | 534 |
| L2786 | JT964193.1 |  |  |  | 478 |
| L2787 | JT953155.1 | disease resistance protein | 9.77E-07 | 72.83% | 559 |
| L2788 | JT919311.1 | LRR receptor-like Serine threonine-protein kinase hsl2 | 1.83E-93 | 77.30% | 600 |
| L2789 | JT927677.1 | uncharacterized endoplasmic reticulum membrane | 2.10E-73 | 86.75% | 653 |
| L2790 | JT917444.1 | protein fam135b-like | 5.37E-15 | 80.95% | 611 |
| L2791 | JT934071.1 | plectin-related isoform 1 | 1.72E-64 | 94.55% | 594 |
| L2792 | JT946373.1 | dihydroxypolyprenylbenzoate methyltransferase | 1.29E-106 | 93.10% | 647 |
| L2793 | JR365080.1 | Ribosomal family protein | 1.52E-64 | 84.20% | 588 |
| L2794 | JT978185.1 | histone H2A | 2.24E-17 | 97.30% | 256 |
| L2795 | JT934988.1 | upf0415 protein c7orf25 like | 6.14E-20 | 73.80% | 189 |
| L2796 | JT964157.1 | Serine threonine-protein kinase yrzf | 1.98E-27 | 84.85% | 664 |
| L2797 | JT975649.1 | cullin-like protein1 | 1.19E-82 | 67.30% | 572 |
| L2798 |  | Ribosomal protein S27 | 9.88E-18 | 68.50% | 532 |
| L2799 | JT948566.1 | 60S ribosomal protein L34 | 4.60E-78 | 86.40% | 583 |
| L2800 | JR349212.1 | copper-transporting ATPase ran1 | 3.75E-30 | 95.80% | 491 |
| L2801 | JT957571.1 | acyl-protein thioesterase 2-like | 5.61E-108 | 96.25% | 615 |
| L2802 | JT934540.1 | pfkb-type carbohydrate kinase family protein | 1.34E-37 | 93.05% | 591 |
| L2803 | JT972388.1 | Protein kinase APK1B, chloroplast precursor | 3.51E-62 | 92.65% | 595 |
| L2804 | JT952582.1 | RNA exonuclease 3 | 8.33E-29 | 80.50% | 265 |
| L2805 | JT948434.1 | f-box wd-40 repeat-containing protein at5g21040-like | 1.20E-12 | 78.55% | 288 |
| L2806 |  | major facilitator superfamily protein | 5.08E-49 | 84.60% | 493 |
| L2807 | JT954545.1 | 50S ribosomal protein L29 | 5.68E-84 | 81.30% | 575 |
| L2808 | JT970595.1 | Integral to membrane, endoplasmic reticulum | 3.94E-85 | 97.95% | 588 |
| L2809 | JT968730.1 | uncharacterized loc101221008 | 2.95E-20 | 88.80% | 637 |
| L2810 | JT929183.1 | Serine decarboxylase-like | 1.20E-37 | 91.35% | 453 |
| L2811 | JT949354.1 | hydroxyproline-rich glycoprotein family isoform 2 | 2.48E-71 | 80.65% | 617 |
| L2812 | JT936508.1 | high affinity nitrate transporter -like | 1.52E-77 | 86.10% | 485 |
| L2813 | JT968080.1 |  |  |  | 567 |
| L2814 | JT924282.1 | 3-ketoacyl- synthase 1 | 1.46E-30 | 88.70% | 205 |
| L2815 | JR360660.1 | lyr family of fe s cluster biogenesis protein | 4.31E-30 | 76.95% | 272 |
| L2816 | JT963670.1 | hypothetical protein JCGZ_18841 | 3.03E-58 | 77.20% | 618 |
| L2817 | JT956946.1 | peroxiredoxin family protein | 9.06E-109 | 93.60% | 633 |
| L2818 | JT922326.1 | potassium transporter 11 family protein | 9.55E-38 | 91.05% | 524 |
| L2819 | JT918804.1 | Ubiquitin-associated ts-n domain-containing protein octicosapeptide phox bemp1 domain-containing | 2.36E-36 | 87.45% | 551 |
| L2820 | JT971070.1 |  |  |  | 711 |
| L2821 | JT918717.1 | WD-repeat protein | 2.30E-113 | 92.90% | 679 |
| L2822 | JT956719.1 | 40S ribosomal protein S12A (RPS12A) | 1.18E-92 | 91.55% | 430 |
| L2823 | JT957564.1 | small nuclear ribonucleoprotein sm d2 isoform x1 | 2.86E-66 | 95.20% | 588 |
| L2824 | JT916029.1 | tocopherol chloroplastic-like | 9.37E-35 | 82.10% | 541 |
| L2825 | JT956468.1 | hypoxia-responsive family protein | 9.91E-48 | 90.75% | 541 |
| L2826 | JT939879.1 | BTB/POZ domain-containing family protein | 7.35E-97 | 93.55% | 458 |
| L2827 | JT958520.1 | hypothetical protein JCGZ_00565 | 5.85E-39 | 80.15% | 578 |
| L2828 | JT944944.1 | hypothetical protein JCGZ_10524 | 1.08E-60 | 79.30% | 657 |
| L2829 | JR345342.1 | nhl domain-containing protein | 3.71E-85 | 81.60% | 677 |
| L2830 | JT935510.1 | casein kinase II subunit alpha-1 | 1.23E-120 | 96.25% | 617 |
| L2831 | JT939804.1 | pentatricopeptide repeat-containing protein at2g15690-like | 1.91E-25 | 53.13% | 530 |
| L2832 | JT921242.1 | ankyrin repeat family protein | 2.24E-87 | 93.95% | 574 |
| L2833 | JR366054.1 | RING-box protein 1a-like | 7.52E-63 | 100.00% | 631 |
| L2834 | JT952857.1 | nucleoside diphosphate kinase | 1.55E-84 | 95.05% | 727 |
| L2835 | JR365431.1 | homeobox-leucine zipper protein hat22-like | 7.67E-60 | 88.05% | 732 |
| L2836 | JT942175.1 | U-box domain-containing protein 30 | 4.22E-31 | 90.00% | 620 |
| L2837 | JT939842.1 | agamous-like MADS-box protein agl11 isoform x1 | 2.09E-51 | 70.45% | 592 |
| L2838 |  | exocyst complex component exo70b1-like | 3.42E-52 | 72.65% | 395 |
| L2839 | JT958121.1 | 40S ribosomal protein S23 | 2.11E-64 | 96.65% | 512 |
| L2840 | JR347629.1 | nucleosome assembly family protein | 1.18E-14 | 92.40% | 185 |
| L2841 | JT917276.1 | phosphoenolpyruvate carboxylase | 2.92E-62 | 93.90% | 564 |
| L2842 | JT927049.1 | dual specificity protein phosphatase dsp8 | 1.65E-104 | 95.70% | 694 |
| L2843 | JR366063.1 | trafficking protein particle complex subunit 6B | 2.90E-20 | 90.30% | 329 |
| L2844 | JT959969.1 | uncharacterized loc101205849 | 4.25E-46 | 95.50% | 568 |
| L2845 | JT951013.1 | copper transporter 1-like | 2.17E-45 | 78.25% | 625 |
| L2846 | JT945494.1 | Rac-like GTP-binding protein arac3 | 3.26E-22 | 98.35% | 587 |
| L2847 | JR364969.1 | ATP synthase subunit mitochondrial-like | 1.38E-40 | 93.90% | 629 |
| L2848 | JT915710.1 | pumilio -like protein | 7.90E-37 | 92.85% | 697 |
| L2849 | JT952676.1 | iron-sulfur cluster assembly protein 1-like | 1.20E-22 | 88.95% | 568 |
| L2850 | JT930825.1 | major facilitator superfamily protein | 2.31E-41 | 84.35% | 678 |
| L2851 | JT929777.1 | palmitoyl-acyl carrier protein chloroplastic-like | 1.75E-32 | 89.15% | 599 |
| L2852 | JT958968.1 | cyclin-dependent protein kinase inhibitor smr2 | 2.55E-30 | 66.95% | 711 |
| L2853 | JR349657.1 |  |  |  | 646 |
| L2854 | JT939246.1 | LIGULELESS1 protein | 5.28E-82 | 66.15% | 500 |
| L2855 | JT944178.1 | MADS-box protein svp-like isoform x1 | 5.53E-86 | 85.50% | 643 |
| L2856 | JT928863.1 | momilactone a synthase | 1.19E-64 | 67.95% | 724 |
| L2857 | JT956026.1 | 60S ribosomal protein L36-2-like | 3.25E-55 | 96.55% | 525 |
| L2858 | JT944825.1 | uncharacterized heme-binding protein | 1.10E-33 | 65.50% | 471 |
| L2859 | JT957119.1 | sec14 cytosolic factor family protein phosphoglyceride transfer family isoform 1 | 4.65E-45 | 65.25% | 637 |
| L2860 | JR365129.1 | light stress-regulated 1 family protein | 6.39E-63 | 75.35% | 648 |
| L2861 | JT944993.1 | endonuclease V isoform x1 | 2.03E-65 | 80.25% | 593 |
| L2862 | JT947784.1 | senescence-associated family protein | 5.34E-77 | 78.80% | 668 |
| L2863 | JT914879.1 | bah helical bundle-like domain isoform 1 | 3.83E-78 | 72.55% | 670 |
| L2864 | JT961678.1 | NADH dehydrogenase | 4.08E-23 | 86.00% | 530 |
| L2865 | JT941106.1 | zinc finger CCHC domain-containing protein 10-like | 1.19E-61 | 93.45% | 683 |
| L2866 | JT944521.1 | Ras-related protein Rabe1a-like | 1.10E-115 | 97.10% | 644 |
| L2867 | JT939910.1 | DNA-directed RNA polymerases IV and V subunit 4 | 7.94E-43 | 83.60% | 571 |
| L2868 | JT945608.1 | tetratricopeptide repeat protein 38 | 1.46E-34 | 91.00% | 288 |
| L2869 | JR344443.1 | transmembrane protein 147 | 2.79E-32 | 97.40% | 579 |
| L2870 | JT945568.1 | mRNA turnover protein 4 homolog | 1.27E-113 | 93.55% | 586 |
| L2871 |  | zinc C3HC4 type (RING finger) protein | 8.63E-11 | 66.71% | 603 |
| L2872 | JT946627.1 | protein tri1-like | 1.94E-22 | 86.35% | 192 |
| L2873 | JT955723.1 | NEDD8-conjugating enzyme ubc12 | 4.53E-98 | 98.45% | 652 |
| L2874 | JT958613.1 | profilin 1 isoform 1 | 8.80E-77 | 95.25% | 733 |
| L2875 | JT959532.1 | hydrophobic protein lti6a | 6.56E-22 | 95.50% | 572 |
| L2876 | JT932058.1 | Exostosin 2 | 6.10E-24 | 65.00% | 620 |
| L2877 | JT924666.1 | lupus la ribonucleoprotein | 3.37E-79 | 78.90% | 617 |
| L2878 | JT945783.1 | methylesterase chloroplastic | 3.84E-51 | 91.40% | 667 |
| L2879 | JT923772.1 | della protein | 5.07E-75 | 73.55% | 589 |
| L2880 | JR352290.1 | 2-nonaprenyl-3-methyl-6-methoxy-1,4-benzoquinol hydroxylase | 4.09E-33 | 80.10% | 574 |
| L2881 | JT947420.1 | kynurenine formamidase-like | 1.96E-59 | 87.55% | 645 |
| L2882 | JT921905.1 | mediator of RNA polymerase II transcription subunit 12 | 8.64E-59 | 56.15% | 614 |
| L2883 | JT962201.1 | conserved hypothetical protein | 5.49E-55 | 88.45% | 523 |
| L2884 | JT968511.1 | NBS-LRR resistance protein RGH1 gene | 5.00E-21 | 83.00% | 685 |
| L2885 | JT953611.1 | enhancer of polycomb-like protein 1 | 3.10E-52 | 89.90% | 525 |
| L2886 | JT949414.1 | basic pentacysteine 7 | 3.99E-49 | 91.35% | 495 |
| L2887 | JT978333.1 | mitochondrial substrate carrier family protein | 1.21E-38 | 86.70% | 707 |
| L2888 | JT968738.1 |  |  |  | 586 |
| L2889 | JT954993.1 | dnl-type zinc finger protein | 3.98E-93 | 77.85% | 673 |
| L2890 | JT935616.1 | latex allergen hev b 7.02 | 8.09E-144 | 78.95% | 685 |
| L2891 | JT971751.1 | rho GTPase-activating protein gacv-like | 9.89E-15 | 66.15% | 687 |
| L2892 | JR365202.1 | CYSTM1 family protein A-like (LOC103335081)* | 1.00E-59 | 78.00% | 690 |
| L2893 | JT942560.1 | hcf106 family protein | 5.68E-78 | 79.75% | 669 |
| L2894 | JR349959.1 | probable protein phosphatase 2c 49 | 3.91E-68 | 77.80% | 631 |
| L2895 | JT936846.1 | 60S ribosomal protein L5 | 7.35E-111 | 96.65% | 660 |
| L2896 | JT949683.1 | Hevea brasiliensis* | 0.00E+00 | 97.00% | 478 |
| L2897 | JT934604.1 | syntaxin-32-like | 7.06E-48 | 97.10% | 483 |
| L2898 | JT915342.1 | hypothetical protein POPTR_0002s08690g | 4.69E-09 | 77.00% | 625 |
| L2899 | JT956668.1 | DNA-binding protein ddb_g0278111-like isoform x2 | 6.58E-75 | 92.35% | 623 |
| L2900 | JT915002.1 | 1-aminocyclopropane-1-carboxylate deaminase | 9.96E-106 | 82.15% | 718 |
| L2901 | JT948689.1 | 60S ribosomal protein L18 | 4.55E-118 | 97.90% | 638 |
| L2902 | JT960872.1 | coatomer subunit alpha-1-like | 7.20E-25 | 81.95% | 604 |
| L2903 | JT937370.1 | Serine hydroxymethyltransferase 7-like | 5.01E-100 | 91.70% | 735 |
| L2904 | JT964168.1 | conserved hypothetical protein | 9.49E-39 | 87.40% | 638 |
| L2905 | JT944945.1 | Vacuolar protein sorting-associated protein 2 homolog 1 | 1.75E-100 | 98.00% | 646 |
| L2906 | JT948232.1 | octicosapeptide phox bem1p family protein isoform 1 | 1.30E-78 | 74.15% | 699 |
| L2907 |  | DNA cross-link repair protein pso2/snm1 | 5.62E-51 | 81.80% | 304 |
| L2908 | JT932054.1 | casein kinase II subunit beta-like isoform x2 | 2.14E-84 | 95.60% | 281 |
| L2909 | JR365582.1 | conserved hypothetical protein | 3.11E-26 | 52.09% | 673 |
| L2910 | JT937298.1 | PREDICTED: uncharacterized protein LOC101307120 | 2.45E-06 | 78.33% | 586 |
| L2911 | JT917120.1 | endonuclease or glycosyl hydrolase with C2H2-type zinc finger isoform 1 | 1.74E-48 | 89.45% | 555 |
| L2912 | JT932296.1 | cyclin-dependent kinases regulatory subunit | 3.89E-45 | 98.00% | 624 |
| L2913 | JT923230.1 | Speckle-type POZ protein | 7.32E-15 | 89.00% | 601 |
| L2914 | JT952575.1 | probable ribose-5-phosphate isomerase 2 | 1.62E-64 | 87.60% | 517 |
| L2915 | JT963310.1 | cytochrome b-c1 complex subunit 8 | 2.49E-40 | 92.55% | 578 |
| L2916 | JT963601.1 | conserved hypothetical protein* | 5.00E-89 | 80.00% | 515 |
| L2917 | JT954559.1 | TATA-box binding family protein | 2.60E-09 | 86.05% | 659 |
| L2918 | JT916383.1 | FYVE domain-containing protein 26 isoform 3 | 6.68E-102 | 90.80% | 585 |
| L2919 | JT962887.1 | f1f0-ATPase inhibitor protein | 1.62E-19 | 83.35% | 626 |
| L2920 | JT942571.1 | protein flx-like 3 isoform x1 | 4.41E-36 | 82.15% | 389 |
| L2921 | JR366604.1 | annexin-like protein rj4 | 4.08E-83 | 84.55% | 659 |
| L2922 | JT954120.1 | 14 kDa zinc-binding protein | 4.29E-87 | 84.10% | 655 |
| L2923 | JT952494.1 | Ubiquitin-60S ribosomal protein l40 | 4.02E-77 | 99.55% | 623 |
| L2924 | JR366554.1 | fasciclin-like arabinogalactan protein 8 | 3.03E-59 | 96.25% | 598 |
| L2925 | JR349772.1 | hypothetical protein JCGZ_05890 | 4.95E-69 | 71.30% | 590 |
| L2926 | JT971394.1 | E3 ubiquitin-protein ligase sina-like 2 | 7.52E-89 | 87.05% | 702 |
| L2927 | JT938949.1 | mediator of RNA polymerase II transcription subunit 22a-like isoform x1 | 3.62E-53 | 92.75% | 621 |
| L2928 | JT971152.1 | SERF-like protein-like (LOC102614321)* | 3.00E-36 | 78.00% | 566 |
| L2929 | JT930385.1 | general transcription factor IIe subunit 1 | 2.51E-39 | 76.70% | 704 |
| L2930 | JT936060.1 | Ribosome maturation protein sbds | 2.71E-125 | 85.60% | 642 |
| L2931 | JT933726.1 | ABC transporter I family member 20 | 2.33E-62 | 93.55% | 609 |
| L2932 | JT951706.1 | DNA polymerase epsilon catalytic subunit | 4.63E-40 | 69.85% | 553 |
| L2933 | JT959621.1 | 40S ribosomal protein S16-like | 8.70E-77 | 99.85% | 655 |
| L2934 | JR349551.1 | NC domain-containing family protein | 1.05E-47 | 74.30% | 483 |
| L2935 | JR360279.1 | cysteine-rich and transmembrane domain-containing protein a-like | 2.80E-13 | 67.90% | 566 |
| L2936 | JT938874.1 | lipid binding protein | 8.55E-55 | 64.65% | 720 |
| L2937 | JT917736.1 | Eukaryotic initiation factor iso-4F subunit p82-34 | 1.36E-77 | 88.80% | 676 |
| L2938 | JT923429.1 | Rop guanine nucleotide exchange factor 1-like | 3.89E-73 | 83.90% | 620 |
| L2939 | JT950864.1 | HSP90 co-chaperone | 4.71E-88 | 89.60% | 717 |
| L2940 | JT928593.1 | protein fam135b-like isoform x1 | 2.34E-32 | 76.95% | 658 |
| L2941 | JT925768.1 | conserved hypothetical protein | 7.49E-89 | 84.30% | 615 |
| L2942 | JT961228.1 | dolichol-phosphate mannosyltransferase subunit 3 | 2.01E-48 | 93.70% | 579 |
| L2943 | JT957295.1 | calcium binding family protein | 2.92E-74 | 81.80% | 649 |
| L2944 | JT916549.1 | golgin candidate 6 | 8.82E-68 | 91.75% | 396 |
| L2945 | JR348929.1 | hypothetical protein JCGZ_15657 | 4.33E-36 | 67.65% | 563 |
| L2946 | JT918681.1 | poly polymerase type 3-like isoform x1 | 2.35E-129 | 96.65% | 611 |
| L2947 | JT956176.1 | 60S ribosomal protein L6-like | 8.37E-49 | 92.75% | 643 |
| L2948 | JT956012.1 | E3 ubiquitin-protein ligase march10 | 4.86E-58 | 75.25% | 675 |
| L2949 | JT978935.1 | protein-tyrosine-phosphatase mkp1-like | 1.25E-33 | 81.15% | 679 |
| L2950 | JT951787.1 |  |  |  | 689 |
| L2951 | JT959043.1 | probable small nuclear ribonucleoprotein G | 3.19E-38 | 88.60% | 589 |
| L2952 | JT929967.1 | elongation factor 1-alpha | 6.77E-143 | 99.05% | 640 |
| L2953 | JT929413.1 | alpha beta-hydrolases superfamily protein | 4.76E-98 | 91.75% | 522 |
| L2954 |  | putative methyltransferase DDB_G0268948 (LOC104816676)* | 2.00E-26 | 75.00% | 227 |
| L2955 | JT916010.1 | dead-box ATP-dependent RNA helicase chloroplastic | 3.60E-45 | 74.15% | 579 |
| L2956 | JT936531.1 | zinc transporter 6 family protein | 1.14E-55 | 76.40% | 547 |
| L2957 | JT949583.1 | protein lurp-one-related 15-like | 2.20E-102 | 75.80% | 612 |
| L2958 | JT949501.1 | ankyrin repeat domain-containing protein 30a-like | 3.22E-47 | 94.75% | 660 |
| L2959 | JT944963.1 | probable glutathione peroxidase 2 | 1.00E-103 | 94.05% | 692 |
| L2960 | JT931383.1 | ABC transporter C family member 5-like | 3.00E-90 | 100.00% | 505 |
| L2961 | JT938607.1 | glucuronoxylan 4-o-methyltransferase 3-like | 1.82E-42 | 88.70% | 660 |
| L2962 | JT963889.1 | cytochrome b-c1 complex subunit 6-like | 3.56E-38 | 94.20% | 504 |
| L2963 | JT958622.1 | lipoate-protein ligase | 4.59E-05 | 69.00% | 592 |
| L2964 | JT924221.1 | TME3 chloroplast | 1.88E-09 | 78.00% | 376 |
| L2965 | JT974356.1 | 30S ribosomal protein S31, mitochondrial precursor | 6.26E-05 | 80.00% | 201 |
| L2966 | JT939084.1 | hypothetical protein JCGZ_12334 | 5.34E-92 | 83.25% | 731 |
| L2967 | JT948740.1 | protein low psII accumulation chloroplastic | 3.09E-20 | 94.70% | 505 |
| L2968 | JT926375.1 | transducin family protein | 2.69E-178 | 95.60% | 608 |
| L2969 | JT960538.1 | DNA-directed RNA polymerases and III subunit rpabc1 | 5.25E-77 | 92.10% | 642 |
| L2970 | JT945897.1 | f-box protein pp2-b10-like | 4.24E-99 | 75.60% | 646 |
| L2971 | JT920361.1 | zinc finger CCCH domain-containing protein 18-like isoform x3 | 1.20E-71 | 82.30% | 636 |
| L2972 | JT930936.1 | cyclin-L1-1 | 4.45E-97 | 78.95% | 615 |
| L2973 | JT944898.1 | protein Iojap, chloroplastic | 6.17E-49 | 94.05% | 576 |

* The unigene was annotated by comparison with the NCBI Non-redundant protein sequences (nr) database (http://www.ncbi.nlm.nih.gov/) with the BlastN algorithm using an E-value cut-off of 10^−5^.
