# Supplementary material for: Profiling Ethylene-Responsive Genes Expressed in the Latex of the Mature Virgin Rubber Trees Using cDNA Microarray
Source: PLoS One. 2016 Mar 17;11(3):e0152039. doi: 10.1371/journal.pone.0152039 (PMC4795647; doi:10.1371/journal.pone.0152039)
Supplement: S3 Table — (DOC) [file pone.0152039.s004.doc]

**S3 Table.** Probe sequences of the *H. brasiliensis* latex cDNA microarray.

>L0073

ATTCTTTTTTTTAATAATAACAATATAAAGTTGCCAGTAAAAACTGGGGGAAAACGAGGGCCGTCAGTAAAATTTGTCCAAGACTCGTGCAAGTATAACCTCTAAGAAATTCAAGGAAAAACTGGAGAAAATACAAGGTCACCAAACAGACGACATACTAACATAAAAAAAACCAAATAAAACATAACCAGACCACCAGATATAGTGCATCTCTTCCTAATTTTTGAATGTCCAGTTATGGGCACTCCGTGGCCCACTTGAAATGCCTTCATAGTATCGAGAGTCAAGCTTTTGATTGTTCCAGTCCTTGACAAAGTCTGTAAACAAATCGCGTGCAGTCTCTGATGAAAGTTCAGAGAAAAACATCTTCTCCTCTTCCCTCAGCCAGGTTGCAAATTCGTTGTTCTTAGAGAAGTAATCATCATTGGAAAGCTCTTGGAATTTCTTGCCATGATCACCTTTGCGATGTTTGCCACTATGCTTATCCTTTGATTTCTTATCTTTATCAGAGCGGTGCTTGTGAGACTTCTTGTACCTTCGCTTCTCTTTCTTATCACTCTTGCTGCTTCTCCTTTCCCGTTCTTCTTCCCTTCTATG

>L0074

TCGAATTCCCCCCCTCCATAAATCCCAGAGATTTCTATAAACAAAATACAGGAAAATAAAACTGTTTCAACTGTCGCCGTTGGATTTCCTCTTCAATCCATCTCCGCCGTTTCTTCTATCTTCTTTTTTCTTCTTTTCTCACCTGCTAATCGCTTGGAAATTTGCTTCCATGATTGGTCGCGGTGGTCGACTAATCTCGATTGCTCGATCTGATGCTCAAGTATCGATCCGATTGCCTTGATTTCCGTGCCTTCGAGCGCTTGAATCCCCATCGAGCTGGGTCTTGATATCGGTGTTTTTTTTCAATTTATTCTTGCTAGTCAGAAACGTTTCTTCAGGCGATTATTTGATATTTCCTTTCT

>L0075

ATTCCATCATAATTTATATTACAAATTACAGAGAGTCTACTACATCATTCGCAAATACCTACCCTTTCTGAACTCCCTCTTCCTCTTTCTCTCAATCACCCACGATGAACGATGCAGATGTCTCCAAACAGATCCAGCAGATGGTCAGATTCATCCGTCAGGAAGCTGAAGAGAAGGCCAACGAGATCTCAGTTTCTGCTGAAGAAGAATTCAATATTGAAAAGTTGCAGCTGGTAGAGGCAGAGAAGAAGAAGATCAGGCAAGAGTATGAGCGTAAAGAGAAGCAAGTCGATGTTCGAAAGAAGATTGAGTACTCCATGCAGCTTAATGCTTCTCGAATTAAAGTTCTTCAAGCCCAAGATGATGTGGTTAATGCCATGAAAGAGGCAGCAGGAAATGATCTTCTGAATGTGAGCCATGATCATCATGTTTACAAAAAGCTTCTGAAAGATCTGATAGTTCAGAGTTTGCTCAGACTGAAGGAGCCTTCTGTCCTGCTGCGCTGCCGAAAAGATGACCTGCATTGGGTGCAGTCTGTCCT

>L2735

AATAAATTAAAAAATTCGATTGATAATTTGATATAATTGCTATGCTTAGTCCCAAAAGAACCCGATTCCGTAAACAACATAGAGGAAGAATGAAAGGAATAGCTTTTCGAGGTAATCGTATTTGTTTCGGCAGATATGCTCTTCAGGCACTTGAACCCGCTTGGATTACATCTAGACAAATAGAAGCGGGGCGACGAGCAATGACACGAAATGCACGCCGCGGTGGAAAAATATGGGTACGCATATTTCCCGACAAACCGGTTACTTTAAGACCTACGGAAACACGTATGGGTTCGGGGAAAGGATCTCCCGAATATTGGGTAGCTGTCGTTAAACCAGGTAGAATACTTTATGAAATGGGCGGAGTAGCAGAAAATATAGCGAGAAAAGCTATTTCAATAGCAGCATCAAAAATGCCTATACGAACTCAATTCATTATTTCGGGATAGGAATATAGAACCAAAGGAAAAAGACCTTTTGGATGCAAAAAAAAATCACAAGTTCCTTTTTTTTTTTTTGGACAAACAATATATCTTTTTTTCCTTTGCATTAAAATAACAGATTAAAAAAAAATGATATGATCCAATCTCAGACCCATTTGAATGTAGCGGATAACAGCGGAG

>L0076

ATTCCAAATACGGATCCACCGCCTTCGATCCGAACCCTGAAACCCTATTCCCTTCTCTCTAGATTTTGTTCTTATCTACACAAACTATGCCAATGAGGAAGGGAGCCAAGAGGAAGAGAGTCCAGAAAGACAAAGAAGATGAAGCAAAAGCTTCTTCTTCTTCTCAAGAAAATCACAAGCAGGAATCCACCAAAGCTCCTACGCGAGCTAAAAGCGTCAAGGCCTCTAAACCCCAACCTGAGCCTGAATACTTCGAGGATAAGCGAAACTTGGAAGATCTATGGAAGGAAGCCTTTCCTGTTGGGACAGAGTGGGATCAATTGGATTCAGTGTATCAATTCAACTGGAATTTTTCCAACTTGGAAGATGCATTTGAAGAGGGAGGCATTCTACATGGAAAGAAAGTTTATCTATTTGGTTGTACAGAGCCTCAATTGGTCCCTTACAAGGATGAACAGAAAGTAATTTGCATACCTGCAGTTGTGGCCATTACGTCTCCCTTCCCTCCTTCTGATAACATTGGAATTAACTCAGTT

>L0077

ATTCCCAACCCATCAACCAGCCTGAGCCCTCACATAGAGCAACACGACGCCGATCACGTCCTGCTCGACGTCGTCAACATCCAGTGCGTCCAGCCTCTCCTCCCGATGATGCTTTGCCCCCACTTGTTGCATTCCACCCTTACTGCATCACATCAGCGCCTGATCATTCTGATCCTATTCCCTCGGCTCCTGCACTATCTGATCCTGCTCATTACACTGGTCGGATGGCTACCCCATCAATGCCTGGCCCATCAGCACCATGGATGTCATATCAAACTCAAATGCAAAACAGAAGCACATTCGACTTTACTGAGAGTCAACAGCCGTAAACAGCATTCGGCGGCTTATTTGCTCCATTTGATAGACCATCAAGCATTGGGCCATCACACTATATGTTAGATCAATTTAGTGGATATGGGTCAGAACAATACTTTGCACCATATCATTCTGGTGCCTTAGGCCATATTCCATCTGCTGCGGGTCTATTTGGATACCATGAACAATTTGCAGTTTAGATTTGACTTAACAAAACCAGATTTCCTAGAATAA

>L0078

GCATCTAACCAAACCATTCAAGGGGATCAGAAATGCAACTATACCATCAGGCACACTAACATTACTAAGATATCCAACACATGTGAAAAACAATGCAAAACGTACACGTTGAACGGAAAATATTCAATCATGTATGAATTAAATCAAAACCTTGTCTCCTCTTTAAATCAAAAATCATGGATTGAGGCCTGAGTACTTCCTGCCTGTGCAAACCCTTGTTTCCCAA

>L0079

ATTCTTTTTTTTTTTTTTTTCAGAGCAGAAAATATGTTCTCCAATTATACTTGTCAATCCGAGACATTATAAAAAAAGAATCAAACGTTCCTACAGACAAGTGATTTCATATGGCAATTGCGAAATTGATAATGTAGAGAGGAGAAGTGAATTACAGTAGATCCAATTTTGAGGACGGAAGAAGGATCGTAGACCATATGAACCGATGTAGAGGGGCTAGACAATCTCCCAATCCTGCAAGCACTTCTCCAAAACCGGTTCCGCTGCCATTTCCACCTCCTTCCTAGCTGTCTCCACTTCTACAGCTACCAATGATTTGTGCACAAGAATTTGGTGAAGTATTTTCATCAGTCCTATCAGCGTGAAAGACTGGAATCTTGAAGAGAGGAGTAGAGGAAGCGACAACGAGAAGAACAGTTAAAAGGAATGGATAAAGAGGCGACGGGGAGGATGCCGTCAAGGCGGGGAAACAGTGATAGAGAGAAAGGTG

>L0080

AAACGCGAATTCCACAGAGAGAGATAGATAGGCGGAAAGAGATGGAGAGAGAGAGAGAGAGAATTCGTAACACTAAATTATAGTTTTGCAGAATAAATTATTCGCAGAAGGATTTCAACCTAGTCCTTTTCGTTTTTGTTTATTTATTTGCTGTTCTTTGATCATCTTCATCCCCATAATCCTCAAAGGAAAAATGCAGTGCGGGCTAAACCTCCATGGAACCTATGGCCTTCCTCTTTCTGTTTCTGGGATCACCCATCGTCATCTTCCTCTCTCTAAAGAGATGGTAGATCATGATCGCAGAAGGGAAGCTATGAAGAAACAAAGATCACAAGCTAGAGAGGGTTTGCGTGCAAATCGAGAACTGGGTTTTGAAAGAAACTTTGAGAGAGTTGTTAGAAACATCCAAGAAAGTGAGATGAATGAAGATCTAGCAAGTTTTTTGCAGGATCTTTCTTGTTCTCTGGCTCGTACTGCTAAAGCTGCATAGTTTGTTTTGGCAAATATGTTAAATAAATTACTTAATTATTGTATGCTTT

>L0081

ATTCGTTAAAATCCCCGACTCCTCGGGGTCTGAGAATCAATTCATCAACCACGAAAAAGAGAAGGAAAATTGTAGCGGAGTTGCGAGTATCAGCCGATCATGTAAAATCCCTAATAATTCCACTATCAAATCCTCACATCACCTCCAAATTATCTTCGTAAATCAACCGGGAAACCGCTCAAGACGTCAAGGAATCATGCTACGAGTGAAAAGCTTTGCTTATTTAGGTAGAAATTAAGCAGAACCAGAGAAAAAGCCCTCCACTGTGAAACCCTAGAGAGCGAATTCGACAAGGGAGTGCTTGTTCTCTGAAGGAGCAGAGTTGGGGTTTTGTTGTTTTCACTATCCCCCTCTCAAGTTTAGACTGCTTGGCGACGCCATGAGTCCTATTCAGAATTTTGAGCAACATTCTAGCCATTTAGCGGAGCTTGACCTCCGTACGCTCTATCCGTCTCTCTCATTTTGTCATTTTTTCTGTTTGTTTAACAAGAAATTGGAGGAAGTGAGAAGAGAATAAATGTAATGTACACTTCGCGTATTTTGATATTAGTTGAAGAAAAT

>L0082

ATTCTTTTTTTTTTTTTTTTTTCCCAAATCAAAAAGCAAGTTCATTGCAAATATGAAACCATCATATCATGGATTGATGCCAGTTTTCTAAAGTTAAACACACTTTTTAATCAGAACAATATGGATGACATTCAAAGCCCACCTCCTCCAGAAGAAGACTAGGACCGGCCAATCTCATGATGCCTTCTATGGCTCATCTAACTGGTCCTGGACATCTGAAGTCCAAAGAGTAAGGTTATCCCTAAGGAGTTGCATGATTAGAGTACTATCCTTGTATGATTCCTCTCCCAAAGTGTCTAGCTCAGCAATGGCTTCCTCGAATGCCTGTTTAGCCATGCTGCAAGCTTTGTCAGATTGATTGAGAATCTCGTAGTAGAACACAGAGAAATTGAGAGCCAACCCCAACCTTATTGGATGTGTTGGTGCAAGATCCGCAAGTGCTATATCCTGAGCGGCCTTATAAGCTAGCATAGTATCCTCAGCAGCAG

>L0083

GGGCTGCAGGAATTCGAATTCCCACCTAATGCTTGGCATCCAATGAGTACACAGAATATGGGGCCTTCCTCCGATGCTGGTGCACGTGGTTTTGGGAGTCGGTTGCCTCCTAGATCTGGTGACCTACCTTTAAATCAGGCTGGTCCCGTGGGTGGCCCTTCCGATGGTGCTCCTCCAGGACATGTTTCTTCTACTGCACAAGTATGTGAAGAACGTCTTTTGATGCCTGCTTGTGTTTTCATGGTCTTCCTAGAATGACTCAAGAATGATAAACCCCTCCAGTCTCCCCAGCATTTGCCTCCTCTTCAACTACACCCTCAAGTTACATCTTATTCACAGACACAAACATCCCATGCTGGTCATGCTCCACCTACTGCTGGTCAGACACCTTTCACTCAAGCACCACCATCACAGCAGTATCTTGGTATGAGTGGACAGTTGTCTGCTTCTCAACCTCAGCTGCAGCAGGGCGCCTCAGCTGGTACACCATTACAAGCACCTTTGAATATCACCTAAATCCCATTCTGGGTCTGCTGGGAAAATCACAACAGATCCTGCTCTGTCCACCGAAATG

>L0084

CTTGAGGGATCATTCTTGGATAAGTTTTTCAAATCCACTGCAAAGATGGATCCACTGGAGCGTGCTGCATATCTTGAGAATGACAGAGAAATGGAAGTTGCACATTCTGTAGCAGCAACAGCTGGTGATACAGAGGCTTCAGATAATGTGGACACTCACTTTATCTGCTTCGCGTGTGTGGAAGGAGAACTTTTTGAGCTTGATGGAAGAAAGTCGGGACCAATATCACATGGTGCATCCTCACCAAGCAACTTAATGCAGGATGCAGCTAAAGTCATACGGAACATGATCCAGAAAAACCCTGACTCCCTTAATTTTAATGTAATCGCCATTTCAAAGAAAGCTGAAGGTGCAATTTAAATTTGGCAGTTTCAGGCTGTACAAGTTTTGTCCAGCCATCCATGATGTGGTCGGTTGTAGGCTTTATAAAACCCTTTGTCATCAGTTAAAGACATGCAGGGAATGATTTTCTGTTAAATGTTCTGTGTAAAGCTAAATCTTTAGCATAAAAAAAAAAAAAGAGAG

>L0085

ATTCCGAGTTTTTTTTTTTTTTTTTAGCAGAATGCTATTTAACGACTCAATTAAAATATTTAAAAACAATTCCAGACATTTTACAGTTATTAAAACATAAAAGGAAACAATTACAGGTCTAAGAGTACATCTACTGGGAACTTCACTCGACCTATCAGATATAAGTGCACCATTAAAGTAGGCAACGCAATTACATATCATACAAGCGCACAAATCAATAATCACCAAGAAAGAGGTGCACATATAAGGATTACAGTAATTGCACCCATGAGATGCCTTCCCAAGGATAATTAACCTTCTAAC

>L0086

ATTCCCATAATCCATCTGGAATCAAAGTGCTCTCCGCTCCGACCATTTATACTAACCTACAAGAAACTGATTTCAAGATCCCAAATCGAAACTGCAAAAGAAAATGGAAGCTTGATTAGATTTGGAAGCAACCTAAAAAGATAGATGGAGAGGTCTTTTTTGTTGAGTCGACTACTGCTCGTTACTCTCTTTCTTGCAGCTACAAGAACAGCAGCTTCAAGACCTGGGTTTCTCTTCACAAGAGCCAAGGAAGATGCCTCCACAGTACTGGAGCAGCAGGAGAGAGGATTGGCCAAGGATGGTCCCACAGACGTCGACGGTATCGAACGTGTTTGGGTCGAGAACGTTTGAGCGGTACAGATCTGGATTGACGTTGTTAGAATCAACGGGTAGGAACGATGATGAAAATGCGTTCGCTGGGTTGTTAAAGCAGGGGAGTGCANCCCTTCTCAACTCTTATGCTACAAAGGGGTTCCCATATGCAGCTTGGG

>L0087

GAATTCCAGTCCACGGCCAACCACCGGACAAACCAACCCGGCTGTTTCTGGTGCTGCAGATTCTGTTAACTATGCAACTGCTGCATCCTTGGGGAATCTGCCTTCCATTAGGGTGTCTGAAGTGCGGGTAGTTCCTATTAGGACTGTGGTTGCTGCAATTCCTGCTTCAGTTAGGCAAACATTTTCTAGTTCGACTCATAGTTTGATGGGCTTATTCCATCCGGTCTTAGATGGAGTTCATGATGTGAATTCTGAAATTCTGGATAGCACAAGTGATTTTCAGTTTCCTAGTGACCATAACCGTAGTTCACCTATCAGTGTTGGTGGTAATGTTGACTTTCAGACTACGGCCACAAATTCTACCCCACATGTGGCTGCTGCTAATGCACAAGAGGCAACACCAACAGTGGGTATTGAAGGAGCTTTCATCTCTAATTTGCTTCATCAAATGATGCCCTTCATAGCTCAAAATGTAGCCTTGGGATCAGAAGTGGCATCTTCAGGGAGAGATGCTGGTAATACAGAAGCTTCAACACAGGCTTCAGACTCAGATGTTGGCACTTCCTCCAGGCCACTAGGTGACCCTTC

>L0088

ACTAGTGGATCCCCGGGCTGGAGGATTCGAATTTCGTTTTTTTTTTTTTTTTTTTTTTTTTTTGGATGCTTTGTCAATCTATGTTTGCTCATCTTGTTGAGAATACATAATAATTAACATCTTTAAGTCAATCACCTAATCACTTTGTTTAAGTCAATCACCTACCACCTGACAACTTCGTAAAACCCTACTCAGACAGAGAACAAGCTTCAAACACCAAACTTTCCAATTACAAAATTCACCATGTAAACACCCCTTTTCTCAGCAAGCAAGAAATCCAATCAAAATCCACAAACAAAAACACCATCAAACACTCCCAATTGAACCTTT

>L0090

GAGCTCAATTCTTTTTTTTTAATAAAATTGATTAACAACTATAGCTCCCAATACCAGAATTCAAAATATGCTTCCTAAACCAAGTCAAGAGCTTTTAAGTATAGTTCCGTACTTCCAAAGCAATCAAATGATTACAATTGCTTAAATAGAAGAGGAAAACACCAGAAGTTGCAGATGAGATGCATGTGCAACTGAGGCACTTTCAGTTCCATTAGGCCAAACTTCTAGGGTGACTCGCTGCCTCTTGGGGCGCGGCGGTTGTCCTCATAATCATCATCAACAGGGCTCCTGTCATCCCTAGGACTGGGACTGGGACTGGGACTGGGACTGCGGCTGCGGCCATTAGTTTGAGCTGGACTTCCATACCTCCCATCTCCAGGGCTCTGTCTTTCAGGGCTTCTGCTCCCCCTTAGGCTACCACTATATTCTGATCTCTCTTGCTGGTCATCCAGTTTCCGAGCTTTGGCAGAAGGGTTTGGGCTGCCTTCATCAGGCGNATGCTGGGTCCTACTCTTGGAGGGCAACATTCTTTGGCTCATGGTCAGGACCTGTATCATCTCA

>L0091

ATTCGTTGTATTTTACAGAGGGCAGCAGGCCCCTTCACTCTGTGGTGAAGAGTTCAGCAATTAATCATCTAAGAGATAGAGAGAGAGAGAGAGACTGGTCTTAGAGTGGGCGCTCTTTGTGTCTCTTGCTCATGTTTGTGGTGGTCTTAACACCATTTCCAGCTCAGGTATGCATGTTGGTTTTTGCTTTGGAAATGGAAGTTGTGCATATAAATACCAAGTTGTTAAAAAAAAAAAAAAAAAAAAAAAAAAAAAAAA

>L0092

TGCAGGAATTCGAATTCGTTTTTTTTTTTTTTTTTTTAATTAAAAATGTAGTATATCTCATTCAAAGTCGACAGCAGGAGGGGGAAAAAACAAACAAGATCACACATCAAAATCTAGTCGTGATTGTAGTATTTTGGACATCATAAACACTATATACAACACTCCCTACAATGGTAAGCTATGCAAAATACTTGAGAAGACAAGCAGCCAAAGCTGCAAGAACCAAATAACTGGAACCAGCATGGTTCGGGCAGCCTTCATTAGAACCACGGTTCCGGTGATTATTTTGGTGATGGTTCATAGCCCCAGCAGCATACAAGGGAACTACAGTTGCACCATGCCTGTACCCTGTATCACCATTACCATTAACTTCTCCGCCATGCGCTCTGCTTCCCATTGAATGGTTCCATGACTCCTCCACTCCTCCACCCTCCTGTCTACTGAGACCGCCGCTCTTGCCCTTCT

>L0093

CTCGAAACGATCGATGATTTATTAAGAAGAAGATAATGGATTCTACAAGATATTGGAGTTGAACAAGAACGCAACCAAAGAGGAAATCCAAGAAGCCTACAAGAGATTGGCTCTCAAATATCACCCTGACAAGCATTCCCAATCTCCTCAACTGGTCAGAGAAAACGCTACCCTCCGTTTCAAACAACTCTCCGAGGCCTACCACATCCTCGCTGATGACCGGAAACGTGCCGATTATAACATTCGCTCCTCCTCCTCCTCATTCTCCTCTGGTTATAATAATCATAGACATGCTCATGGTTATGGCTACGGTTATGGCTACAATTACCATTCTTATCAGAGCCAGAATAGAAAAGCCGATGGTGG

>L0094

GGGACATTTTTTTATTCCATCTATCATATCATAAAGCTTCTGAACCTGTCACGATAGCAATAATAATAATAATAAGCCTAACTTATCTAAACTATCCGGTGCGTGGCGCCCCCCCAAGTTACACATCACAAGCCCGCGATAGCTTCCAATCCAATGGGCTTTGAATGAATGATTGATCCAGTAAAGTGCGTCAGCAGNGAGACGTGTTGCGCTTAGCCGCATTACCTTTTGATGAATTCACGCGTGCCCTCTTTCTATCCGTTGGATCTCCATTAAGGTCAAAGCTCACTTCATCTC

>L0095

TTTTTTTTTGTTTGTTTGTTTGGAATCAAAGAAAGGAAAGGAAAAGAAAACAAAAAGAAAAGAAAGGGATTATTATTGTTATTATCATTAGGGCGGTCAGGTCGTATGATGTTTTGTGGTGTATTGAAAGAT

>L0096

AAACCAGTTGTATCTAGTCGGCAGTCAAACCTTTCAAGTCAGAAGACAGGTCCATCTAATTCTGATGGAATTAGTACTTCTCCTAGCAAGCTAGATGCATCAAACACAGTTTTTGCGAAGCCAAAGGTGAAGCCAGTTCTTGAAGATCTGGTTGAAGAAGCAAACTCAAGACCTGGCCTTGTACAAAGATCTCACAGCAACCCAAAAGAGAGGTTAATACAATCAGATAAACAGGCAAAGAAGAGGTTGTCTCTGCCTAATGGTGGGGGAGGTGCTGGGGGTCAAGCAGCGACGCATGGCAGAATTGCAGGTAAAGGGACAGGAGGTTCT

>L0097

TAAGAATAACAATCCTCCAATTCGAGCTAGGCATCAATGTGCTTATCTCGAAGCATACCACGACTGGGTGTTGAAGGGATTAACTCATACGGTTTTGACAAATTGATCCTAGTAGCTGCATCACAGCTCAGTGACCAATTCCTGAATCCAGGCAAGCCACTAGAATCTCCTCTTGGAGCTGCAAACTGTATACCAGAAGTCACATGACCCGACACCAGTATCTGAGCATTCAGAGTTGAGTTCTTGGGAGCATTTCTGCCGGTCAAAGTCGCCCCTTCAAGTGCACCAAGCTGTGCTTCGCGTGACCAATATTGGTCGGGAGGGTCTTGTTTTGGGTTTCTAACAGAAGTTATATATATATATATATATATATATATAGAGAGAGAGAT

>L0098

GGTTTTTTTGTATGTTAATTGCTCATGAAATCACATTCCAAACAGAAAAAACAAATTCTGTGCTAGTATATTTTACAGTGAAACATAATTGAATCTTAAATGGTTCAGACAAACTATTATACAAGCTAAAAGAAAGACCAGTGAATATATGACAACACACCACGCCAAATTTCATGAATAACTTTGTTACCTTTTAGCAAAGATACGTCATTGATGCCCTGTACATTTCTTGATCCAAGACATAATAAAGTGGCCATTGATTTTCTCAGGAAAATATACTTCATGGATTATGAAGTCATTCACCTTGACGATTATACCAATTCAAACTGTTTGTTTATGTTAAAAGTAGATGTCCGGCTAGGCTCAACAGCTCATTTTTCACAACAGAGGAAATGAAAGGATACATGCTGATCCTGTATTGATTTCATCCACTGTCAGAGTCATCAGTCTGCTCCTCCTCAATTTCTCTTATGCTCTGTACAACATCAGCAACTTTCTTAGAAACATG

>L0099

GAATTCATTTTCAGTTTGTCTTTCCTCTGACCCAAAACAGCCCTCACAAAGAAGGTATGTTCCTAGTGCTGGAGGAGCAGTAACTGATCAAAGCTATGCTGGCCACGGGATATGTGTCGGGAAAAGCAGTAAGCAAGGCAAAAGTGCATGGTGAATCTCATCAAGTTACTGCAACTGAGCAGCTAAGGTTGCTGAGCTGACTGATAGAGTTGGCCTTGCTGATAAAATTTTCGCTGGCATGGGAGCTGTCAAAGCTTTGGGTCAGAAGTACCATGCCTCTGGTATGACCAAATCAGCTGCATCAGCAACAGGAAGAACAGCTGCAGCAGCAGCAGCAAACACTGTGGTAAACAGTAGCTACTTCTCCAACTTAGATACAGCGTTTGATACTGTATATCATATGTAGTGTGTAACTAGTTGATAGTTTTNCTACTTTTGTATAGCACTTGCTGGTTTTTCACTTCCTTTGCCAGTATAGC

>L0100

TACATTTTTTTCTGTAACCATTTTCCTCAATGAATTTTTGTCACCATTTGCGATTAGAGTGCTTATCTCCTTGTACAAGTCAATGGCCTCTTTGTAGAACTCATGTTTCGAGTATCCAGATTTTCTTAACTTGGAAATGGCATAAGCACTTTTCATCTCTAGAATGATATCTTCTTTTGTTCGTCTCCAACCACTTTTTGTGAACCACCTTCGCCAAAATGGGATTGGTTCACGAGGTGCATAAGGTTCATAGATGAATCCAGGACTCGAAATGGACACTTTAAGAGCCCCCATCTGCCGTGCTTGTGCGGGAGCTCTTGCCTGTGTTGTCGCCAACCTCTTGTTGCTTAAGAAAATTGATAATTCCATGGCCATTGAAGAATGAAGGGTTGTCC

>L0101

AAACTGCTGATATCCCTGGGTTGCTTTGATTGGTCAGTCAGTTCAATAGCTTCTGCACAGTCTGGATCTGAGAGCTCATTGGAAACTGCATGGAAAGCAGAGAGTGAAAGTCTTAAATCGATACTGACTGGAGCCCCTCAAAGTAAGCTAGGCAGAATAGCTTTGGTGGATGTTCTGGCACATCAGATTCGCAAGCGTATGA

>L0102

TCACATGGAAAATTACCACCAGCTTTCATTTCAATCTTCCTCGCCGAATAGAATGCGAAGGCGAGATGCGAAGAGGCCGGTAGGGTCGAAAGAGGCTTCGTGGAGTAGGCCACGCTTGATGTTATTCACTCGATCGGTTAGGGTTTGCTGCTCTTGCTTAAGAGCTGAAGTGTCTTCCTGAAGGTCATGGAGACGGTACTTTTGACCTAAGGATCTGATACTCAAAAGCAGTATCCCTGTCATTGC

>L0103

GGCTGCAGGAATTCGAATTCCATGTTTTTCTGTGTGTTGAAGAACTTAGGCGGTTCAACTATCCTTCAGCAGATGCATCTTCCTCCCCATATTTCTCCCTTGACTGGTGTTGCCGCATCAGTGATGCATCTGAGCTGGTCATTGAGCGTCCGTTCGTAACCTTAATTCTTCAGCATGCTACAAAAAAATTCTGTTTCTCACCAAAGGGTCACAAGGACATTGCAACCATTGATAAGGAAAATACAGGCTGCCCTTTGACATGGAAGCTCAAGTGGTTCTCTGAAGACAGTCTATTCAATTTTGTAGCACTGTTGAATGCAATTCATTCAGGTATAAAGAGTTCTGCTCTGATCATAACACGTGTGTCATGAGACTCATCGTTTGTATAATATTAGTTGAGTTGTTTCATATTCATTTCATTCTTTTTTTTTTTTTTTTCCGATTTAATTGATTTTTAGTTTCGTATATTCTAGTGTGGCAATTCAATAGCTTTCTTTGCTTGTGATTCTTTTATTTGTATTCTTTTAGTATTGTGTACAAATTGTAGAGCACAAAACATTAAAGAAAAATTTAAGGTTAAAAAAAAAAA

>L0104

CATTTTTTTTTCTTTTTTTTCAAACACTTAAAAAACTATCATGACTTTAATTTCAATCCATTTTAGTACAAGTCTGAATTTATTTCAAAAAATATAAAAACAACTTTACCCCACTCCAAATAATTATGGAATTTATTATTTTTAAAATTATTTGGAACACAAGGAACCTACAGGCACACACACATGGTCAAAACACTAAGCAAAAGCAGCAGCAGCAGTTTCTTCCGGCAACGGTTTCACCTTAGGGATATAGTCACTCATGGCTGGTCCCTTGGATTGGCCTTCAAAAGCCTTCCAGGCTTCCCATTTGGCTCGGTCCCTCATGTAAAACATCCCAGGAGGGCGGGTGTTCACGGGTCCAACAGGGGCTGGCTTATAAAGCCCATATAAAATAAGTTGGCTCTCATTGGTGGTATTTTCGGGAAGGGTCTTACCTTTCCTCAGCATACTCCTCAAATCCCTCCTTCAAACCCATCTTTAA

>L0105

ATTCCCAACTTTCTACAAAACCCTAATCGTTTTGCCTCCCAAAACGATGCGTTTGTTTGCCAAATTCAATACAATTCCATAACCATGAAGCTCTAGTTTTCATTTCAATGGCCTCTTATCGACTCCTCTCCTGTTATCAGTATTATCAATAACGACAACAACTGAAGCTTTGGTGATTCATATTCACGCTCCATGGAAGCTCGTAGAGGTTGGCGTGCCGTTCCCAAGGTTCGCCAGGTCGGATTCTTCACTCCCAATGAGCCACCGCCGCCTGCTCGGACTCAGTCTGGGCCGCCTGACTCTAACTCGCCGCCGCTTTCCAATTCTCCTGCGGGCAACTCCTTATCTCCAGTTATGATCCCCCCGCCACGTCACCTCTCTGATAACCTTGCTCATCGCGCCACTTCGCCACTCCCTGTCCCAGAGACATCGGCCTTCCGCCGACCTATCCCCGGCGGCGACCGTGTGGCGGTGGTGGGCAGCTACAACCCTACCGATTCGATGCTTGGAGCGTCGCCGCCGCTGACCTCGCCTTCAAGTAGGATCGGCGATGGAT

>L0106

ATTCGTTTTTTTTTTTTTTTTTCAAAAAAAACTCCTTTTATTTCATTAAACAGTAAAACATACATCACAAACATCGTAATCTCTATCATCAACATACCTAAACTACAAGCATTACTATTATTATGGTTACAGAACTACAAGCATTATTTGGAATTTGCTTAACCACAAGCTGAAACAGAACAGAGAACAGAGAACAGAACAAACCCCATTTTTTTCAAGCCTCAATCCACCTCTTCAATCTTTGGCCCTGCTCCAGAACCACCAGAGCTAGAAGGGCCATACCCACTTCCGGGCTGAGCACCGCCGCCCATTGGCACATCTTCACCACCACCACCTTGATACATCTTTGAAATAATTGGATTGCACAAGCCCTCTAATTCCTTCAATTTGTCTTCAAATTCATCCACCTCTGCCAGTTGGTTCCCGTCAAGCCATTCAGTTGTCTCATCAATGGCCTTCTCAATCTTCTGCTTGTCTGCTGGATTCAGTTTACCTGCAATTTTCTCATCCTTCACCGTATTTCTCATATTATAAGCGTAATTCTCTAGCTTGTTCTTGGGAGAGAGAACTAGTGCGCGCTCAC

>L0107

ATTCCGCTCTCTTCTCTCTCTTGAATCTTCTCTTGTCCAATTCACGCAGCGAGATCCTTCTGCCGGAGCTGATCACTGGAGGCCATGGAGGACGATTTCGAGTTTGCCGATCACTCTCCTCCGTCGTTCCAGAACGTGGGGAATGTGGTCAAGGATGTTGAGGCTAGAGGATTCAACCCAGGATTGATAGTGCTGCTGGTTGTTGGCGGGCTAGTTCTGGCATTCCTAATTGGAAATTATGCTCTCTACATGTATGCGCAGAAGACACTTCCTCCCAAAAAGAAGAAGCCAATCTCCAAGAAGAAAATGAAGAAGGAAAGACTGAAGCAAGGCGTCTCTGCACCAGGAGAGTGAAATTTGAGATGTGTTTGCTATTCGTAACTTGTGTTAAACTCTTGTGTTCGAAATTTTCCCGCCTTTCATGTCAAGAGAGCTATATTATTTCACTTTTTAGCTTTCATATTTGATAGAAATTTTAATATTGAAGTTCACAATCTGTCGTATCGGGTAGAATATTTGACTTGAATGTAGCTTGCCTTTTTTCTTTCCACTGCGGTGGCCTATGATAATCGTATCGTAGTGCAACCTCTCCGTTCTTTATGGTTAGC

>L0108

GTCTCTTGCGGCCATTTCCACCCTCCTCCTCCTCCTCCTGCTCCTATCATCCCTTGCCGCCTCATCTCCCGCCGCCATCGCCGATGATACCCCGACGGCGTCTGAGGTCCTCGCGGGATACACTTTCCCAATTGGTATCCTCCCTAAGGGCGCGACAGGGTACGAATTCGACACTCGCACGGGTAAATTCCTCGCCTATTTGAACGGTTCGTGCAGCTTCGCTCTGGCAGGTTCC

>L0109

CCCGGGCTGCAGGATCGAATTCCTCTGCAAGAGATAAGGAGATTTTGGCTAGGGTTAGGGTTTCATTTTTGTTTCTGTTTCTCAATTTCAGAGCTTTAATGGAAGAGATTACAGAGGGAGTGAACAACATTAACTTCACCGGTGATTTGCACAAGAAAAATCGTATTCAAGTCTCCAACACCAAGAAGCCCTTATTCTTCTACGTCAATCTCGCCAAGAGGTACATGCAGCAGCACAACGAGGTGGAACTGTCTGCACTTGGAATGGCTATTGCCACCGTTGTCACTGTTGCAGAAATTCTGAAAAACAATGGATTGGCTGTTGAAAAGAAGATCATGACTTCAACGGTTGATATGAGAGATGAATCAAGAGGGCGGCCTGTCCAAAAAGCCAAGATCGAGATATTGCTGGGGAAGACTGAAAACTTTGATGAATTGATGGCAGCTGCTGCTGAAAAAAGAGACATTGTAGATGGCGAAGAGCAGAGTTGAAAATGGGATGCTCCTTATTATCTGAGGGTCTGTTTTCGATGAATTATTAGCCAAGAACACTTTTAATAAGTCAATTCTTAGATTCTAGGTGCCTGAGGTGATGT

>L0110

GGATATTCCTTGCAGTCAACTACAATTCCTTGCAAGCCATTAGCTTGGAAGGAAATCCAGCACAGAAAAATGTTGGAGATGAACAACTCAAGAAATATCTGCAAGGCCTTCTTCCCCATCTTGTTTACTTCAACAGGCAGCCAATTAGAGTCAGCACATTAAAAGATGCTGCAGACCGATCAGTAAGGCTGGGCATCAATGCCCATCAGTTTGATCGTGGCCTAAAGTCAGATAACAAAGCTACACGGAAGAGTAGCCATGGACTAGCTGGCACCAGGCCAACATCTTCATCAATTCACGGTCGTAAAAGTCAAGCTGTAGTCTCACCGAAGCGGTCCAGGGGTAGGCATGTTCGTCTGCCACCAACTGGGACTAAGGCAACAACCAATCTTCGGCATCATTATTTTGATATCAGTAACAAACTGAACTTTAGATCAGAGCTGTCAATGCACAGGAGTCGAAGTGAGGGAACTTTGAGAGTCCTGTGAGATCTGTTATGATTTCAAGAGTCTATTACGCTACTTGAAGT

>L0111

CCGGGCTGCAGGATTCGAATTCCACAAGGCTAAGGAAGCTAGATTGAGAGCAAATAAGATGGCCAAAAACCCAAGATCTATGCCATTGCCAAAGAACTCTACAAAGCCAGCTAAGCTTGCTACCTGTACAGCAAGCTTAAAACTAGAGGCAAAAGTTGAAGTGGAATCACTTCAAGTTGAGGAAAGTAATAATGACGTAGACGAGAAAACAGGGAATAAACAAGATCCAGTGAACCAAAATGACAAGTTACAATTGCTGAAAAATGTGCATGAAAAAATCTTGAAGAAAAATGGAGCAGCAAAATTGCAGAGAAAGGATAGGAAAGCAGAACTTAGGAAGGCAAAAAAATTTAAGTTCTGGTGTGAAATGTGTCAGATGGGTGCTTACTCTGCGGTGGTGATGGAGGCTCATAGAAAAGGGAAGAAAATCAAGGCAGGCTGGCAGAACTTATCAAAACAGCGAA

>L0112

TTCTGGTTCTTTTGAAATTCATCTAGTGCCTATATATACATGAATTTATGCAAGCAAGACTAGTCAACTTTGTTAGCCAATTAACTAAATTACTCTCTGCTTAGTTTCTCAAGAATCTGAGCAGTCTTTTTCCATTGGAAATAAGGAAGTCCTCAAGCCCCATGGCAAAGTACTGTTGCTCAGTGAGAGCGAATTTCTCATAATAAACTTTCTTGATTAGGAAACTCCTGAACATGTCACATTGATCAGTTGTGTGCAAATCTGTTAAGTGCCATCTACAGAAGCTCTCCAGTCCATCTTTGTATATAGGAAACTCCAGGACACCAGAGCTGGCCATGTCGTTGAACAGATCAATAGCAGCAGTAGTTTTCCAGGTACTATTTGCCAGATTTAACAACATTGGTTTATGCTCACGGATGTAGTCTCTTGATCTTACATCAGCCTGTCGGACAGGCTCTGGT

>L0113

GGCTGCAGGAATTCGAATTCTTTTTTTTTTTTTTGCCAGAACAAATCAGTGACAAGTTCCAATTCCAGTCCACTAATAAATATCATCCTGATTTTAACAATGAGTGAAAAATGCCTGGCATAAACCAGTCAATTCACTTGCCCTCAACAATCTTACATCATAACCAGCCATTTTGGGACTTAGATAGTTAATACCTTCGGAACATCGACTGGGAACTGGTGGATCTCATCCATCCAGGGGCTCTTTTCCTTAGCAGGGTTGATGGTCCTTAGAGCCTTCCAAACTGCACTATTACACTTAAACCCTGAACCAAATGCTATTTGCCATGTTCTATCTCCCTTCTTCATTCTTCCTTGGGCTTCCGAATATGCCAATTCATACCAAAGGGAACTGCTTGAGGTATTGCCAAACCGATAAAGTGTCATCCTTGATGGCTCCATATGCCAATCAGACAGCTGCAAGTTCTTCTCCAATTCATCCAACACTGCTCTTCCCCCAGCATGAATGCAGAAATGCTCAAAAG

>L0114

ATTCGTCTGAGTTTTTTTTTTTTTTTTTTTTTTTTTTTAGGACAGCCACCATCCACGTCATATTATTCACAAAATTGAAGGGTCCTCCATCATGGTATGAAACTTTAGTCACTAATTTGAAGTTTCAAGTTAAAAAGAAACGCTTATGGTAATCAATCTCATAGTTTCCCCTGCAGTTGTAATTCCAACGAATAATAAGAGATAACATCATACCAGCGAAGGAACAATGGAAGCCCAGAGAGGAGCTGATGCAATGAAATCCAGACTTTATTTATCAAACTCCCATATGACGCCTCCATCCTCTTTAACTTTCTTGTTTATCCAGATCTCAAGAAGCATACCAGCACCAATTCCACCTGCAATACATGCCCCATAGAATGCAATTGCAGAGGGTAAAGATCTTCGAGGAAGGAAATAGTAATCTGGAATTTGTCTAATCTTTTTTGGTAACCGTTTCTTTATGACTGGTTTAGCACCAGCACTAGGGTCCTTCTGCAATTCACCGCCTATGGCTTTCCAGTCAACCCCCTTGAATGGATCTTTTGGCTCTTCAGTTCCCATGGCCAGACGCCACTATACTTTACAGCAGAATCCTGTAAATACAAAGCTCTGGAATGACCGTTGCCGGAGTCACCATTCGCCAGCCGCAAAAGGGTCTCGAGGGGGGGCCCGGTACCC

>L0115

ATCGCCCATTACAGATACCTTCTCTATCGCTCTTCCTCTTCTGATATCTTTCTCTGTCTAGATTTGGCCACCGAAACCCCGCATTCCATGGGGTCTATGCTCGGTGACTGGCCTTCCTTTGACCCTCACAACTTTAGCCAACTTAGACCCACTGATCCTTCCAATCCATCGAAAATGACTCCTGCTACTTATCATCCTACTCACAACCGTACTCTTCCACCACCTGATCAAGTGATAACTACTGAAGCCAAAAATATTCTTCTGAGAAACTTCTATGAGCGAGCTGAAGAGAAGTTAAGACCAAAGAGAGCTGCCTCCGAAAATCTAATACCAGAGCATGGTTGCAAGCAGCCTAGGGCTTCTACTTCATGCTAAGCTTTGTTTACTGTTGGAAGTACAACATGCCGGTTGTCAATGTAAATACAAGTCAAGTCATGTCATGTCATGCCAAATGTGTCAATTTGTGTGAAAGGGATTTGCTTGCGCAATGCTCTGAACTTTAGAGCCATACATGTAAATATGTGTGTAGAAGTGAGATTTACAGCTGAGTACTCAAATATAAAAGATTGAGATGAATAGTTATGCTT

>L0116

ATCCGTTTTTTTTTTTTTTTTTGGGTAATTCAACTCACTAACAAAATTTGTACTATACAGGACCTAAATTCGACGAACCTGATTGGAGCGGGTGAGCAACGTGAGGGGCTGTACTTTCTCAAGGGAGTGACATCAGTACATACTTGCAAGGTAACTGATGTGGGGTCTTTTGAGCTTTGGCATAAGAGAATGGGTCATCCTTCTTATAAGGTGGTAGAGTTAATTCTAGAAGCTGGTAGCATAGTTAGAAAAGCTGATAAAACTTGTGAAGTTTGTTTTAGAGCAAAGCAAACAAGAGAGATTTTTTTTTCTAGTAATCCAACTTCTGAAATTACATCCAACGCATACTTACGTTGACACAAGAAAATACCATTCGAGTTTCTGGATATAAATAAGCATATTCAATTGAACAGTCCCAGCTCGAAAAGTGAACAAAGAGTAATCTGAATATGATTGCTGAAATCCATAAGCTTTCAGAGATGCAGCCAATTTCGCAAACCAACATCTAGGTGCTTGCCGCAAACCGTATCGCAAACCGTATCGCAAACCAACATTCAATGACTCTCTTTGCCTTATAGTTAGCAAAGCGCGCTCACTGGCC

>L0117

GGCTGCAGGAATTCGAATTCCTTATGTTCGGTTGCTGAGAAACCTAAGGAAAGTTCAAGATTTTTCTCGGCGTCACCATTTCCGGATCAGCGCTGAATCACTCCTTTAATTAGTTGAATCCCAGTGTTTGATCAATGGAAGCCAATGAAAGACCTGAAGAAACTGTCTCAAATGCAAGTCCAAAATTGGCTGGAACTGCTAACTGGGGCACAGCAACAGTCATTGGAGTGTTTGCGGGCATGCTATATGGAGGTAGCAAGGAGGCAGCTGCCTCAGTTAGCAAGGATGCAGAAGTAATGTTGAAGCTTGGGAGCACTCCAGACAAGCGTGAACAGTATCGATTAATGAGGGATGCAATGGAGAAAAGGTTTATCCGAGTCACTCGTGGTTCAATAGTTGGTGGAGTGCGCCTTGGAATGTTCACTGCTGCATTCTACGGTTTACAAAATTTGCTGGCTGAGAAACGGGGTTTACATGACGTTTTCAATGTTGTGGGAGCCGGTTCAGCCACTGCTGCTACATTTGGTTTAATTTTGCCTGGACCTTTCCGATGGCGTGCAAGGAATGTGCTGCTGGGATCAGTTCTGGGTGCAGCATTCTGCTTCCCTCTTGGGTGGATCACTTGAAGCTTATAGAGAAAGCAATGAAGGAAATCGGGCCTTAATCCTGAGCTTCCCTCTTGGGTGGATCACTTGAAGCTTATAGAGAAAGCAATGAAGGAAATCGGGCCTTAATCCTGA

>L0118

ATTCGTTTTTTTTTTTTTTTTTTTTTAGCCATCAATTATTTGGCAATGCATTAAAGGAAACCTAACGAGATCCATGATTCCAACAGATGACCAATCTTTGTCTACATTATCACCAAAAAGATACCGTCCCATCAGTTGCAAAAGCAACAGCCACAAAAGATTAAAAGACTAATTGAAAACAAACAAACAAGACATTCAGTTTCTGAACTTCAATGCTTCATCGTATCCTGGAAATTGTCTCATCATCTTCCTGAATTTTATTCCAAGAGGAAAACTGAAAAATGCTGTAATTAGTGATGATCTTCATCGTCTCCATGCCCATCAGGAGGTCCTCCAGGGCCCACTACTTCCAGCACAAAGTACTGCGAGCAAACTGGACATTCATGAGGCTTGCCTTTCTCCAGCCAAAACCAAACAACGTCATGCTCATCCTCTCCTTCACCTCCAGGGCAACCAACAATTCTCTTGTCATAGTATGACTTGACAACAGCCGGAGCTTCCTTTGTGCCAAAAGGACCAACAGGATGGTTAATTTCAAGAACATCCTTCCCCTGAAGCTCAGCTTCAAGCTCCTCGCGCTCATGT

>L0119

GGCTGCAGGAATTCGAATTCCCCTTCGTTAACAATGGTAGGCGGCTTGGAAGTAGGCAACGTTCCCTTCAACCCCGACGGCTGGGGCCCTCCAGATGCCACAACAGCCACCGCGTCCACCACTACCCTCCCTCTCAATGTTCCTTTCGCTCCCTTTTCCCGATCAGAAAAAATCGGCCGAATCGCCGACTGGACCCGCAATGTCAACAATCCCAACGCAAGTCGCCCGAACGCCAACAAAACCGCGTCGGATTCAGTTTTTGACTTCACCGCCGATGATTCCTTTCCCGCGGCAACTGCTGCCGGTGATGATTCATCATTCCGCCTTGTAGATGGAAAACCTCCTCCCCGCCCCAAATTCGGGCCCAAGTGGCGGTTCAATCAACACCGGCCGCAGCTTCCACAGCGACGTGACGAAGAGGTGGAAGCTCGTAAACGGGAGGCGGAAAAGGAACGGGCCCGCCGTGACCGGCTTTACAACCTCAACCGTTCCAACCAAAACCAGCCACGTCGCGAGGCTGCCGCTTTCAAGTCATCGGTGGACATCCAACCAGAGTGGAACATGCTGGATCAGATCCCCTTTTCACCTTTTCAAAGCTTTCATTCACAGTCCCAG

>L0120

GGCTGCAGGCGCGGCAACCACCTTGCCCCCTCACCTAGCCGCTACCAATACCTCCGCCGGAACCTACCCTGACTCCATTGATTCCTCTCCTCGCTCTCGCACGACTGATTCTTATTTTGATGACCCCCCTCCCCTCTCCTCAAAGCTCCGCCTCATGTGCTCTAACGGTGGCCACATCGTCCCTCGCCCCCACGACAAGTCCTTGTGTTACGTTGGCGGCGACACCCGCATCGTTGTTGTCGACCGTCACACTAGTCTCTCTCTCTCTCTCAATTAAGATAGGCCAAAATTTTATTTCAACTATGGAAAACATAGAAAGTAGCAGCAACACAATACAGGCTACAGTATTTGACAGTACAAGTATGAGCAAAGGCAGGGATAATTAAGCAGGAGAAGGCTGGTGAAGCTTAGAGGCTTCAAGATCAGCTGCTTGCTTGAGAAAGTTCTGGTTATCAT

>L0121

TTTTTTTTTTCGACAAATAACAACTATCAGCGTCTTTCATTTTCAATAATAATTGAGTTGTAAATAGCACAACGGGGGCACAGAAGTCAGGATAGGCATCACAACCACAGATTTTCTGCTCTATGATTCCAAAGAGACACCAAAGAAACAAGCTTTAATTGCTACACTAAACTAGGACCAAGGCACAGGCTCTAACACCAGCAATAAATTCTTAAGTGTTTTTCAGACCATGGTGATTCATGGTCTTAGGTTGATTTTGTGTAGATGCGGATGCCAAAAGCCAAACCCAATATAGCAAGGGGAACCAAGAATTGCAGGAGCTTGATAATGAACTCAGAGGTCTTATCCTGGTTGTAGTGAGGCTGATTTGGAGGTTTATATGCAGCCTTCTTAGGGATGGTTGAAGAGTCAATCTCTCCAACATAGTATTGATCCATCATTTCTCTAGCACTTGTGCTGTGGCCCACATCCTCGAAATCATCAGTTGCATCTTTCCCTGTGGCAGACAACAAGACCTCATCACCACCAGGATGGTCTTCCAAAAACTTTGTCACATCGTAAACCTTGCCGTCGATAATCAACCAACAGTCCTTGGGATTGTTGTGCTCAGACACCTGAGCCAAGGTGAAAACTTTCCCTTCACCACCCATCTGGTTTCTATTCCCTTTTCTTCGAAAACGTGAGATATAAA

>L0122

ATTCCAGCACATTTCTCATTTTCTTGCCTCAGTTTCTTCTTCAATTGTAACTCTTCAGCTCAAAATTGGCCATGAGGAGTGCTGCTATCTCTGCAATTCGCGCTTTCGCTTCATCTTCTAGGATTTCTGGCTCTCCAGCTATCGCTAGACACTTGCATGCCTCTGCGGGGAGCAAAAAGATTGTGGGTGTTTTCTACAAGGCCAATGAGTATGCTGCAATGAATCCCAATTTTTTGGGGTCTGCGGAGGGAGCTTTAGGCATACGTGAGTGGTTGGAATCACAAGGTCACCAATACATTGTCACTGATGACAAAGAAGGACCAAACTGTGAACTTGAAAAGCATATTCCTGATCTCCACGTCCTCATAAGCACACCCTTCCACCCTGCCTATGTTACGGCAGAAAGGATTAATAAGGCCAAAAACTTGCTACTGCTTCTCACAGCCGGAGTTGGCTCTGACCATATA

>L0123

ATTCGATTTTTCTTGCTTGCTTGTTGTTTAAAAGTGGCAATAATGGATGTAATCTCTCACAGATCATCACTGAATCCCAATGCTCCATTGTTCGTCCCTCTGGCGTATCGCGCAGTTGAGGACTTCTCTGACCAGTGGTGGAGCCTCGTCCACTCCTCCCCTTGGTTCCGCGACTACTGGCTCCAAGAATGCTTCCACGATCCCCAATTTGACCCTGTCATCAACGATATTTGCGACCTCGACTCTCTCTTCTTCGACGCCGGCGATGATACTTGTACCCACAAACAGGAGGAGGAGGAGGAGGAGAATGAGCATAGCAGGGATTTGGTATCATTGGGGGTGATGAAATGGCAAGTGGGTCGAGCTCAGCTTGTTCAGGCACCGAGGCATGCGGAGAAGGCACCCAAGATTGTGACTGTGAAAATGAGTCCACGGTTGATTCAGCAACCGAGGTAGATTACGTGATTCAACGAGTTTGAGGATGATCGAGATGGGTTGCTTTTGTAGTTGTACTGTACATAAGACCCTGACTTCCATTTACTCGTTTGTTTTCTGTATTCCATGCTCTGTGTATCATCCTGTAAGCTTTTAACTTCTACTCTATTTCATAATAAAAGGAAAA

>L0124

GGCTGCAGGAATTCGAATTCATACGTCTGCCGAAGAAGAAAAGGAAGCTTCTGTTCCCTGCTTGCGATAAGATCCTCGATTGAAGAAAAGAGAAATGGATATTAGGTTGTTTGGTCCCGAATCACCCCTCCTTTCCACCATCCAGCATCTGATGGACACGACCGATGAAGCTGAAAAGTCAATCAACGCGCCGACTCGTACTTATGTAAGGGACGCGAAGGCCATGGCTTCAACTCCGGCTGACGTCAAAGAGTATCCCAACTCCTATGTGTTTATCATCGACATGCCAGGGTTGAAATCTGGGGACATCAAGGTCCATGTGGAGGATGACAATATGCTGCTGATCAGCGGAGAGAGGAAGCGTGAAGAGGAGAAGGAAGGTGCTAAGTATGTGAGGATGGAAAGAAGGGTCGGCAAGTTTATGAGGAAGTTTGTGCTGCCTGAAAATGCTAATGCTGATGCCATTTCGGCGGTCTGCCAGGATGGGGTTTTGACTGTCACTGTGGAGAAGTTGCCACCGCCGGAGCCCAAAAAGCCCAAGACCATTGAGGTTAAGATTGCTTGAGGAGTATTATTGTGTACCATCTATGGTTGGGGTACTGCAAATAAAGTAGGTTCTGTTATAGCTTGGTGTGGACTACGACTATG

>L0125

GGCTGCAGGAATTCGAATTCCTCTTCTCCTATCATGGCCATCGCCGCCTGCTACTTCACCGCCTCCCTCTCCAACTTCAATCCCAGTCTCAATCTCCATCGACCATTTCTTTCCTCCCACTCATCACCTCTTCTCCACCTCCGCCTCTCCCTCTCCTCGCCCTCCATTTCTCCTCTTTCCCTCCGCCACCAACACACTAACCCTTTCCCCCTCTTCGTCCCTTCCGCTTCTTCTTCTGGCGGAGATGGAAGCTTCAATAATGGCCCCCCTTCCAGCGGCGGCGGTGGTGGAGGCAATGACGACTATAATAGTAATAACAGCGGCGGTGATGAGGATGGTGATAATGCTGCGAACAGGAACACGAAAGAGGCGATGATGGTGTTAGCGGAGTCGAAGCGGGCACTGGAGACTTTGCCCAAGGACTTGGCGGCAGGGATCCAGGCCGGCAGAATTCCTGGTGCGGTGGTTTCAAGGTTTTTAGAG

>L0126

GGCTGCAGGATTCGAATTCGTTCAAGAATTTAATCAGAAAATTACTTGTTCTATATCGACGATGAGTGTCAGGACAATTCCGTATCCATCTATAAATCATTAAAATTGCAGGGTGTTGATCGAAGTTGCAAGAGATCCTATTGTTTATTCTATCACATTCTTGCCTTGTCAAGGTCCGCTGCAACAGCTTTGGCTATATCGTCTGCTGGCAAACATCATCCAATTTTAGTTTTGGAACACTTGCCCTGATCGCTAGATAATTTGCATAAACTTCCATGCTTCATCTACTGAATTTCCCTGAAGAAGTCCATCACGAATCTGCGAGGCAATATCTTTCACTGCACCAGGGCCATGTGGGATGAAAACAGACGAGGATTTTGAGGACGCTCCAATTTCCTTCATTGTATCGAAGTATTGAGTCAACAGCACCATATCCATCACATCCTTAGCACTTGTTCCAGGCACATTCTCAGAGAAGCCAAGCACGCTATCCCTTAGTCCATCAACAATCGCCTGGCGCTGACGAGCTATGCCAACCCCTGAAAGCTGTGAAATCAAGATTCAGTTCTTAGACTATGCTNCAGAATAAAATCATAGAACACTAGAGTGTTAGCAGTGATAAATAGAGGCTGAATTATCAATCTGGTATTCTAAACTC

>L0127

GGCTGCAGGAATTCGAATTCTTTTTTTTTTTTTTTTTGGAACACCAAAAGAAGCATAAAGATTAAGAAGCTCAGGCAGAAAAAGGAATAATACAAGCACTTTGGAATGAGTGTATCAACAATATATAGTAAAAAAATGTAAATTACATGAAACTTCTTGTAAATTAGTGAAGGCACATTAACCTCTTAAGAAGTAACAGAGGAATTGGATAGCCTAAGGCACTCGCTTTGGCACCTCCGTAAACAGACATCATTCCAGTTGGGCACTTGATGTTGATTAACACAGACAGTTCTCGCGCAAGCATCAGCACAAGCATCAAGCTCAGAAACACCACAAACGGTGACACAGGTATCAATGGTAGGGTCTTTACATAATGCACCAACCTTTTTTAACATCCTCTTAAAAGTGCACACCCTCTCACAAACAAAAATTTCATCAGAGCTTTCCTCACGTCCTCTGGCAGTCTTAAACCTCTGCT

>L0128

GGCTGCAGGAATTCGAATTCTTTTTTTTTTTTTTTTCATTAAAAGCTTCAAGTGCTTCTTTGTATTCTCTCTCCTTTTTCTGACAGGTCTGCCCCAGTGGCTTCAACTCCCTGTTAACTGCATCTATCCTTTTTCGCACGATTGAAACTTCTTTCCGCAGTGGATCTGTGAGAGCTTCAAGCTCTTCTCGTATCCCAGCTAAGCGTTTGGTTGCTTCTTCGATTCGTCCCAACTGAGCCTGAACCCTATCTCTCACTTCCATTTTCTTCCTCTCAATCTCTTCTTCCTTGGCTCGAAACATAGCCAATGCTGATCTTGACATCTCCTCCTCCTTGTCATCACTTAATGGACTTTCATTGGTACTAATAGTACCTGAATGCTTCAATCTCTGCATTTGTGATTGTTGCTGCTCCAATATCTGTTTCCGGGTTGTCATTGCTCCAATATGTCTTTTCTCTTTTCTTGATCTTTCTCCGTGGTATGATAATTGTTCTCTCTCT

>L0129

GGCTGCAGGAATTCGAATTCCTTTTCAATACAGGACCCTATTTCTGTCTTTGAAATCTTGCCGAAGTATGGCCTTCCAAGCGGACTATTACCCAATTCAGTAACAAACTACACCCTCTCAGACGACGGCCGCTTCATTGTTCTTCTGGGGAAACCGTGTTATATTCAATTCGAGTACTTGGTCTATTACGATAAGCAAATTACTGGCAAGTTGAGTTATGGGTCTATCACAGATTTGAAAGGGATTGAGGTTCAAAGATTGTTTTTGTGGTTCAATGTTGATGAGATCAAGGTTGATTTGCCTCCTTCCGATTCTATTTATTTCCATGTGGGCATAATCAATAAGAAACTTGATGTTGATCAGTTTAAGACCGTCCACTCTTGCCGCAATAAGGTCTCTGGGTCCTGCGGCGGATTCTGGAATCAAATTCTTGAGCTTCCAACTCCCACAGATGATATTCAGATGCTAATTACGGAGTAGAGGCAACCAAGTGTTTCTGCTGTATTGTAAGGTAGCTGATAAACTTTGATATCCCAGTCATGGTCATTTTCGGACTCACAAACTAAGAGTCTGTAAGAGTCATGTATGAACTACTCATGAGAATTTGCCCCCTTATTGAGCTTAATC

>L0130

GGCTGCAGGTTTTTTTTTAAAACCCAAGAATATAAAATTTTATTGAAGTATTTGACCTGCCATGCAAGCGCACCTGCCATGGCAAAGGTCAGGCAACAAAAAGCAGCAAATAAACATCAATTAACCAATAATCTCCTCAGCCCTCCCAGCAACCTTCCATCATCCTACTTCTATAGTATTACCTCATTCTCCAGCCAGAAGAACACAAAACCATAATAACCGAACAGATCCGTAAAAAAGACAACCACAAACCCAACCTTTTGTCGCCTAATAATCTTCACGAAAGCCAGGTCCAGAATACCATCAACGCAGATCGTCAGATTAGAAGGCACCAAATTTTCCAGATACCCGGAAGCACAGCATGCCCAACAGTCAGATCCATCTCCCATGGAGAGGCCAATCACCATCGGTTCGAACATCTCCACTGCCGGTTATCTCCAACCAAACAAAACAAAAGCTCCGAGATGGACATATCAGCTAGGGAAAGAGATCTTCACCAGTCGTAAATCCATAGATCTCGCCAACTGAGCTCACGAAGCTCCAACGTTCGCAGATCTGAGCTGAAAAGGAGACGGCTACGGCTTGCTCGTAACAGAACTATCTGCGCTACGAAGAACCTGCAA

>L0131

GGACAACCTGCAGGAATAAAGACCGCATCTCCAAGCTTCTGGACAAATGTCCATGGCTCAATAGCATATTCCTCTTTCAGCTTCCTCTTATGCTCTAGAGTCAAATAAAAGGTCTGATCATGTATAGGATGAACAACCTTCTGCAATGGGCAACAGTGAATGTGCCTAAATTCTTTGAAGTGTTTGCTAAGATATTCCTGCAACTTAGGAACATCTTCTCTTCGGAAAATGTCCCAAACAGCACCTCCCTCATCTGACCGCAATGACACATTTCCAGAACTTGATGGCCCACTATTCTCAGTGCCTCTACCTTGAACAGCATCTGCCTCGCAACATTTCAGCTCTAATTTATCTTGAAATACTTCTGCCTGCTTGCTAATTTTCAACTCTGATTTACTTGAAAAGGCACAATCAGTAAACTGAGAATCCTCAGTTTGGTTGTCAACTTCCCCAGTTTCCTTATCAGTAGTCGGTAAGCTGCCACAAAAACTACCGTGCATTTTGCCATCAATATCCTCCTCTACAACCTGATTGTTTCCAAAAATTTCTCT

>L0132

ATTCCTCTCTTAGTTCTTCCAATGTCTTCTTGCCACTCTTCTTCATAACCTCCCTTCCTCTCATCGACCCATCATCTTCATCCTTCCCATTCACATTATCACTGCGCCTCTCAAGGTACCAGGGCAACTTAACTCCTTTTCCAGCAACCCCATAACCCAACCTATACTTCTCATCCTCAGCAGTCACAACTCTCACCTCCTCTTTCTTCATCTTCTTCTTTCTAAACCCATCCCTCTCAGCATCCCCTTCTTTCTCCAATCCCTTAACTGGGTCAAAAATCTTGATCCCTTCAAAGAGATTAATGTGGTTGGACTTGGGTTCTGATTGCTCTGGCTTTGGCTCTGGCTCTGGCTCCACTTCTGGTTTATTTAGCGGAAGCAAACCACGAGCAGTCCGTAGCTGCTCAAGGCGAAACTCAGCATCGCGCTTTCTTGACTGCTCACGCTTTAATTGCTCTTCTCTAGCGGCGGCTTCCTCATCTCGCCGGACTTTTTCTCTATTCTCATAGTTGTATACATTCCACCGCTTTTGAGGAAGGATGTTAAGACCTCCATGACCTCCCA

>L0133

GGCTGCAGGAATTCGAATTCCAAAAAAAAAAAAAAAAAAAAAAATGGGGACGCGATTTTCTAAAGCGGCTCCTTGTAAAGTTGCAACAGGCTACAAAATTCCTCAAGCTCCTAATAAACCCTCTCTAAAAAACCGTCAAACGCGCGTTTATAAATCCCAGCAGAAAAAAAAGGAATGGGGAAGAAGAAGAAAAGAGCAGAAGTAGCAGAGAGAAACGAGCTTGAGTTCGAGGAGCTAAGTAATGGCGATTGTCATAGGAAGATGAAGAAGAAAAAGAAAAAGGAAAGAATTGAGACCGAGAAAGAATTGACCGAAGCTAAGGGGAAACCTACAGTGAGCATAGCCGTCCCCGGTTCCATCATCAACAACGCTCAATCACTTGAACTCGCTACTCGATTGGCTGGTCAGATTGCCCGCGCCGCGACCATTTTTCGAATCGATGAGGTGGTGGTGTTTGACGATAAAAGTAATTCAGTGAATGAGCATCCCACTGTGAACACAGGCAGTAACTCTGATGATAGTGAAAGCGGTGCGGCTTTTCTCGTGAGAATCTTGCGCTATGCGCGCTCA

>L0134

ATTCCCCAGGTGAAGAACAACCCAGGTCCCCTCACACCTAGAAAAATCCCGTCCAAAACCATCGCCTCCTCCTCCTCCTCCTCCTTCCCCTCAAGTCCAGAAAAATCATCTTCAAAGCCTACGATTTCAGCCAACGCTTTTGTTCTTCTTCGACGGCGACGCTCCCTCCACTTCGCCGCTGTTCAAGTCGTATCGGTCCACTATTCAATCTCAGCCTCAACCTCGACGCTGCCCAACAACTCTTTGTTCCTTGCGGAAGCAATTCGGTCCGTTTTGCAGCCCAACAGCAGCGGGAGCAGAACCAACATCTCCTATTAACAACAGCAGCAGCAGCAGCAGGAGTCTACCACCGACACCACAGCAACAGTGGCAGCAGTGGGTGGCGTCGAGGAATCTTTCTGGCGATGGTGGTTCTCGGTGGATGGCGCCGAGTAATCTGGCTTCTATCGACGCCACCTGTTTCTTATCTACTGCTGCTTTTTGTATGTTAATTACAGCTTTTAAGCCCATGTGCTTTATTCTCTGATTGAGAATTTTTGGGAATTGTTCTTAAATTTATTGATTAGTGTTGTTAATTTCCAGATTATTGATGATGTTTTGGGTTGTGTTGTTGAATTTCTAATGTA

>L0135

GGCTGCAGGATTCGAATTCACTACATACTCCTCACATCCTCAACAACTGCCTCTTCTTCCTTGTTCCCACTTCTCCCATCTGCAACTATCCTCTTCTAAAAATGTCATGCAAGGCAAATAAGAGAGTGAGTTTCTGTCCTGATGTCCAGGAGAAGCCCATTTTCTCTTCTAAACATGGAGGTGGAATCAGAGTTGCTGCAAACAGGAAGAGGGTTGCCGGAATCTTCAGCTTTAGGCTCCTCAGAAGCTCCAAATGTTCACCTGCAAGAATACTCAGGCGACTCGGTGCTAAGGTAGCTAGAGTTCTACGTCTTGTGTCCATGAAAAGGAAGTCTTCACGCAAGGTTTCTTCAGCCAGTTTGACAAGATCAAGATCTCTTGCAGATGCAATAGACTCCCAGAGAGCTGAAGCTATAGAAGATTGTATTGAGTTCTTAAATTCTTCTTCCTCTTTGCAGAGGTCAAATTCAGTTTCTACAAATTCTTGCTAGAATTTTCAATCTCAGTTTCTACAAATTCTTGCTAGAAAATTCAATCACAATCATATGTAAATGAAAGAAAACAACAAATATATGATTCATTAATTGATCAAAAAAAAAAAAAAAAAAAAGAGAGAGAGAGAGAGAGAACTAGT

>L0136

GAAGGGACAATAGGGCTTGCAGCTCTAGTGTCTCAACCAAAAAAAGAAAAAATTTGGATCATTCTCTCCATCTCCACCCATTCCTCCTCACAGTAGTCTCTCACCATGGTGGAAACTTAGTTGTCTCTCTTTACCACAGCAAATCAGTTCATGAAAAGAAGTGAGAGAAGAATTGCAGAGGAGGAGAACGGAATCGCCCTTGTAGCTGTCAACTCTTGTTTCTAGTTTTTGGCTTCAACGAATTAACCATACCTTCCTTTATCTCCTCTAGAAACCTAATACTGCATACCCATCGATTCAGTTGAGAATTGAAAGGGGAGATATAGATTGAAGAGCTTAAGAGTGATGATTCACTCAACACTAGCAGAACTTATGCACTTGCAAATTGCCATGGAGATCACTTGAGTATACTTTACCAGCCACTAACTCCAACAGACTGCTTTGACACTAGAAAGCGGGGTGCACGCTTCCACAATTCCAATTATACACATGAGACGGGTAAATAGGTCCAAATCCTTCATATTTGCAGGTATTGATGCTTAGCCTTCAATTACATGTTGGGTAAGGT

>L0137

GGCTGCAGGAATTCAAATTCCAGTTTTTTTTTTTTTTTTACTCCTTTTCCTTTCCCTCTCCTCCTCCCCCTCTCGCCTCTTCCTCCTTTCCCTTCTCTTCCTCCTCCTCCTCTTCTTTTCCCTCTCACTCTCACTCTCACTCTCACTTTCCTCCGTACCAGACTATGACTCTGAATATTCCGACTCTTCACTGTCGAAATCTGAATCCGAATCGTAGCTTCTACTGCCTCTCTCTCTCTCTCTCTCTCTCTCTCTCTCTCTCTCTGTGCAGATCTTGATCGTCTCACTACCACACCCAGAACCTGAAAAATGGCCAAAGATCGTAATGGCTCTCTATCTAGTAAGGACAATAGAAAATCCAAAGGAAAAG

>L0138

GGCTGCAGGAATTCGAATTCCGTATCAGGATTCGGGGAGGGAGAGAGCCATCCAAGAAGGAGCAGCGCGGCAGCGCCATCGGGGACCAGTCACCTACCATCGCCGGCACGTTGCCCCCACCAGGCAGAATGGCTAATGAAGAATATAAGGTTACTGGGTCGAGTTCTCAGAATGAAGGGTGTCCAAAGTCCAGTGCAGAAAGGGCTACAAGTAGGAAGAATAAAAAGAAAAAGCAATCGCAGGAAAGTGTAAAAACAGAGCATGAACAGGAGATCAATGAACACAGCCCTCCAGCTGGAGGGAATTTACAAGCTGGTGATATGAGGGTGGAAAGTGGGAAAAATAAAATAAAGAAGAAATTGTTGGAGGAAGCTTCTTTAAGCAAGGAAGGTAGTAAAAAATCTAATGGTGAGAGGAAAAAGAAAGTAAAGGAAACAAAAAGGCAATTATTACAGAAAACTGCTGAGGATGAAGAGGAAGATGTTAATACAGTGGGTTTTCCACAGGAGGTGAATCCAGCAGCTGAAGGTGGAAAAATTGAAACTGCAAAAGGCAAGGAAGAAGAAGAGGTCATTGGAATAAATCCTATAGAAGAAGAGGTCATTGGAATAAATCCTATAGAAGAAGAGGATCCAAAATCCA

>L0139

TCAATTCTGATCGCCGTTACGGTTGGGTGTTTGATGAATGGAAAGAACCCTCAGAGGAAGCTCTAGCTGGTGGCCGGGGAATGTTCTGCATATTGCCTCTGGCAAAAGCTTTCCTGAATACTGCTACGAACTCGGTTAATCTTGCAGCAAATTTTGCTGTCAAAGTTTTTGAAAGGCCAGATCTTTTCTCCCCTCAGGCACTGCAGGCAAATCTTGATAAGCAGCTTAAAAAATTCGGTTCTTCCATGAAAACCCCAGAAATCAACATTTTTGCTCTCAATGTAAAGAATTCATCGAAAACCTCCACCTTCCTGTCACATCTACAAGTAGGAACTACAGAATCAGATATGACTTGAAGTATGTAAACTTCACTTCCTATAATTTCATGTGAATCTACTTTATATTATTTCAAGTCTTTGATGGATTTATATGGATTTTCATGTATGAAACACAATATTGTTGGCTTGTAACAACACTATATTTGATAGATGATGAATCTGTTCACGTACAAAAAAAAGAGAGAGAGAACTAGT

>L0140

GGCTGCAGGCTCCCCTAACATATTCATTTTCTCTCTTAAAGCCTGAGACTTTCCAGACACATCTCTAAATCCTCTCTACTCAACGCAGGTTTGTAATAAAAAAGAATGGGATTTGATGAAGAAGCAAGCTCATCCTCCCGTGTGCTTCATGTGCCATCCTTTCCAAGAGAAGACACCCCACTTCTAGGCAAGAAAACCCGTCTCTCCTCTACATTCAAGACCTTTTCCAACGTCTTCATAGCCATTGTTGGAGCAGGTGTTCTTGGCCTCCCTTAAACCTTCAAGAAAACAGGATGGTCATGGGTTCTCACGTGCTCTTCTCTGTTGCCTTTCCCACATACTACTTTATGATGCTCCTCGTTTACACTCGCCGCAAGCTTGAATCCCTTCAAGGCTTCTCCGAGATAGCTTCTTTTGGTGATCTGGGTCATACTGTTTGTGGTCCTGTTGGTCATTTTGCTGTGGATGCCATGATCGTCCTTGCGCATGCTGGGTTTTGTGTAAGCTATCTAATCTTTATAGAGAACACTTTAGCCTATGTTTTTAATCGTCAATCAAGTGAAAAAATTCTGGGTTTCTTAAGTCCCAAGTCTTTATTTGTATAAAAAA

>L0141

GGCTGCAGGAATTCGAATTCCTTTTTGAAGCAACGATGAACCAGGTGAAAGAGACCTCAAAGTCCTTCACATCGCTTCTCTTATCCCTCATGCGTGCTGCCCACTGGGACATTGCTGCTGCTGTCCGCTCCATTGAAGCTGCAACTGCCACCGCTGAAAATGTCGCCACCATCAACCACACTACTGCCATAGCATCCACCATTGTAACCCACCATGCCAAGTATGCTCTGGAGTCCTACATTTCTCGAAAAATCTTCCAGGGCTTCGACCACGAGACGTTCTACATGGATGGCAGCCTTTCCTCGCTTCTCAACCCAGACCAGTTCCGCCGTGACTGCTTTACTCAGTACCGAGACATGAAGGCCATGGACCCAATTGAGCTTCTAGGAATCTTGCCAACTTGTCACTTTGGGAAATTCTGCTTCAGAAAGTATGTTGCCATAGTTCATCCAAAAATGGAGGAGTCCTTGTTTGGAAACTTGGAGCAACGCCAGCAAGTATTGGCTGGTAGCCATCCGAGGAGTCAGTTTTATGGTGAGTTCTTGGGGCTGGCCAAAGCCATTTGGTTGCTTCACTTGCTGGCATTCTCGCTCGACCCTGCACCAAGTCAGTTTGAGGCAAGTAGGGGAGCTGAGTTTCATCCACAATATA

>L0142

ATTCGTTTTTTTTTTTTTTTTTTTTTTTTTTTTTTTTTTTTTTTTTTTTATTGGGAATGAGTGAATCCATTGCAAGTTGTCTATACTCTTGACACTTGACAATGACTCCTGGTAAATCCTTTATGGAGTGAGTTAAAACGAGGACTATAAAATCATTTAACTATTTAACTGTAAACTCTGAGCCTCAAAGGCAATGAACATTACATCAAGATTTTTTTTTTTTCCCTTTTTGCAATTAAAAGAAACAGAGATAATAAGTGTTCAGCCATTGCATGTAAATAGGCAGGTGAAGTTCCTGCCTATTTAACCAGTTCTAAATAGGCAGGTGAAGTTCCTGTCTGATATTTAAAGAAAACGGGCCAACGACGATTATAGAGTAATTATACAAACCGAGAACACATGCCATAACCCCTAAAGCTTCTATCCCATGACTCAGCTTCCCATTGGTTAATATATTGAAATATCTAAGCGTAACAATCACGCGCCTCCAACTTCCTGTGGCAGTTCCCCACCCTTTCTTGCACGTCCAGCTCGGGTTTTTAGCCACTCCATCATATCACCATGCACCAAATTCACGCTCTCCTCCGGT

>L0143

CTAAGGGAGGTTAGGGACTGTCAGGCTAAGGCAAGAGGTATTCCATACAAGAAGAAGAAGAAGAAGCCCAGTAGCAAAGGAGATGATGAATCAAGCTCCGCCATGCACTTCTAGTCTTACATGTTCATATCCTTTTCTGGATATTTTACTATATGGATCTTGTTGAAGTGCATGTGGTTGGAGAATCAAGAACTGGAAGAAAATTGTTAAAAGGGATAGTGAATATTTTTGAAGTTGCTGTTTATTTCTTTGCTGTCAATTGCGTATATGATGCATGGTATCGTCATAGGGATGGAGCTGATGATCTTTTGAAGTACTTGCTGTAACTTTTTCTTTGCTATCAATTGAGTATATGATGGTATGATCAAGGGATATGGAGCTGATGATCTTCAAGAACTCTTCTCTCGCAGGATGTTCTAATGATTTTTTTCTCTCTCTCCCTTTATTA

>L0144

GGCTGCAGGAATTCGAATTCCCTAACCCTAATTCCAGCTTCTTAACAGTTCACAGACACTCAAAATGGACACCATCGGAGTCCTAATGACCTGCCCAATGTACTCAT

>L0145

ATTCTTTTTTTTTTTTTTTGGGCACCATTATTATACTATTCTAAACAGAAAATGAGGTTCAAAAGAGTAAGCAAAAGAGTTTACTTTAAAAAAAATTGGCACCTGAAGCTACAGATATATACCAAAACATTTCTGTATTCCTTGGAATTCCATTTATCATTTGACAATGCTACTCAGTCACATAAGGGACCCTAGACAAATAAGATCACTTGTACTTAACAAAGAGTAAAAGACAACGCCTCTTACAAATTGCTTTATGTCAATTCTTGGCCTTAACAGAGTTATTTTCCTTGATTCTTGCACGGTTGTCCAACTTGACAAAGCGTTTGAGATCTTCATAGGGGAGAGTTTCAATTTTTTCCCTTCTTAGTGCACTCAGTGCAGGACGTTTATCAAGCAGATGCCACAGCCAATCAGGGTATTCAGAATCAGGCAAGATCTTGGGATCTGCTCCATCCTTGAGAATATTGGCACCGACTACAGTAGTAGATTTAATTCCTTCGTTAAGTGTAGATCTTGGAGCATCAGCTGCTGCTCCCCCTTTGGCACCCTTTTTTGCTTTGCC

>L0146

ATTCGTTGAGTCCGGATTACTGTCATTCTTGGTCTTGGTTGGGTGCTTAATTTTTTTTTATATTTGGATGGTCACAACTGTCTTCCTTTTTCTTGATACTTCATAATTTGTTTTCTGGTACTGGTTTTCTTTTCTGTGAGGATACTTGAGGTGAGAATTCTGTATTTTTTGAAGCTTTTTAATTCAGGAAACTGTTTTGATTTCTAACGCTTCTAGCCTATCGTAATCCTTCTAGCTTACACAAGCCAATTTCTATCAGTTTTATACTACTGAATTTTATTGTTATGAGTTCTTTGACACTTATCTGTAAGCCATAGTGTTTTCCCTCTTAAATGATTTCTGGGTGTCAACGAGTTGTTTATGATCTGCGGATATTTGTGT

>L0147

GGCTGCAGGAATTCGAATTCGTCCTGACGCTTCACGGTTGAAACGAAGTATAGTAGCTAAAAAGGCGATGGTTGTCCACGATGTCCAGAGTGGAAACAATGGAAATCAATAACAGCGATTCACATTCCCATGAAAATGGGCATTTGAGAGGTTCAAGTAACTCCAGCAAAGAGAATTCTGCCGAGAAGGATGCGTTTGTTAACCATGCTGAGGAAGCTTGGCATGAGAAACGAAAACAGTGGACGGGTGATCTGTCTCCAAAGTCACAGAGAATGCCTAGAGAGCCAATTATGAGCTGGACCACAACATACGAGGATCTGCTTTGCTCCTCTGAACCTTTTCAACAGCCAATACCCTTAGCTGAGATGGTGGACTTTTTAGTTGACATATGGCATGAAGAAGGCCTTTATGACTAGATTGTTGAAATGCACGGTTGTTTGTGCAAAATTGGTTGCATGGTATATAATTGATTCTCTGAACAAGTGTTAAAATGTAAAGTTTATGTATTTTACCTTTTTCACAGTTGTGATTTACTGACAGCCCTATATTACACTGAACTTTTGAAGTTCTGGATGAGTGATAATGCTCGAGGGGGGGC

>L0148

GGCTGCAGGATTCGAATTCCTCCTCTCCATTTCTAAATATGCAGGCCAGTGATAGGTTTAACATCAATTCCCAGCTTGAGCATCTCCAAGCTAAGTATGTTGGGACTGGTCACGCCGATTTAAATAGATTTGAGTGGGCGGTGAACATCCAGCGTGATAGCTATGCATCATATATTGGCCATTACCCGATACTGGCATACTTTGCTATTGCAGAAAATGAGTCTATTGGAAGAGAACGCTACAACTTTATGCAGAAAATGCTTTTGCCTTGTGGTCTACCCCCTGAAAGAGAAGATGATTGAGAATTCAGGCGATCAAGTTAGAACTGATTAATTCTATGGTCTTGGCTGTGAAGCTATCCGTTTGTGACATTGTTGTCTGTGTATGCTTGAAGGAATGGTAACTGTTATGGCAGCGATGAGTACTGAAATTATGGTCTCATTATATGTTTGTGTACTTGGGAAAATATAATATGATTTATGGTTTCACA

>L0149

CCAATGCAGCCACTTGGCCCTAGTATGTCACCTGCTCCAGGTCCTCCAATTTCACCAGGTGTCTTTATTCCACCATTTTCTTCTCCTGTAGTTTGGCCTGGGG

>L0150

ATTCCTGGCTGCAGCATATTCTTTTTTCCCAACGAATTAGGCGTCGTCCGAATAAAATGAGAAGCTGAAGCTGAATTCAAGTTTTTATTTTTGATATTCGATCAATCTATGGCTTCGCAATTGCTGGCAGCAGCGGCGGAGAAAATCGGACCTGCCGTGAGGCGGCAAGCCCTAAACTTAACCGACGCGGCAGCTTCTAGAATACGCCATCTATTGCAGCATCGCCAACGGCCTTACCTCAAGCTCGGCGTCAAGGCTCGCGGCTGCAATGGCTTATCCTACACGCTCAATTACGCTGATGAGAAAGGAAAGTTTGATGAATTGGTTGAGGAAAAGGGTGTAAAGATACTTATTGAACCAAAAGCTCTCATGCACTTTATTGGAACTAAGATGGATTTTGTTGATGACAAACTGAGGGCTGAGTTCATATTTATAAATCCAAACTCTAAAGGCCAATGTGGTTGTGGCGAATCTTTCATGACAACAACTAGTGCTGGAGCTGCTAAGCAGGTTGGCAGCACATAAGACTTGTGATGGCTTGAGATATCATAACTGACCCATTGTTTCATACTCACCATGCACTGATGGTATCTAGCAAAACACTTGTATGAGAGCACACCTAGTTCCCTTAGCAAAAGAAAGCAAAGCAGTTCTATTATATGAGATCTACTCCATATGGATTTATATG

>L0151

GGCTGCAGGAATTCGAATTCCAGGTTTTTTTTATTGGTTTTACCATTCCTATTAGGACTGTTACATCTTAAGTGCACTTAAATTTTGTTCCTTGTTTTAAAAGATTTGGATGTATATGTTGCTTCCAGATAAACTGTTTCTTCTACCGAATACTAAGAAAAGAAGAAAAAAAGAAAAAAAAAAGTATTCTAATTAAAGAAGAGAATTAAAGCGGAAGGATACACCTGAGCACGCAGCTATAGCAAACTGGTCCGATCTAGAGCTCGCAGGCCTCTAGGGTTTCACTGAAGGAATCTTTCAACAATGGCGATGGCTGCTTTGAGACGTGAAGGGAGACGATTCGCTGCCCCTCTCATCTCCTCCCAACCGATCACCGCCATCCGATCCTCTCTTATTGCTGAGGAGCAGGCGCCTCTCGGAATACGTTCTATTTCAACTCAAGTTGTCATAAATAGAATGAATAGTGTCAAAAATATTCAGAAAATTACTAAGGCCATGAAGATGGTTGCAGCCTCAAAGTTACGTGCAATTCAAACTCGAGGGGGGGCCCGG

>L0152

ATTCTTTTTTTTTTTTTTAAAAATAGCCTTATTCAGAAGCTAGAATTAACATTCAAACACCACAAGCATCCAAGTACACTGCAATTATAACCGTATATATTTAGCATATGAACTACCTTAGTGGCGACAATGATAAACATTCTTTGTCACAGAGTGACCATAAGAGATAAGGTAGAGAACATTTGCAATATCTAGCTAGGTCTTAACTTGAAGACAACACATCACCCAGTCACCCTGCCTTTCAATTGTCACTCCTTTTGGGCTTGTCTGAAGCCAAAGTGACCTGACACAAGAAGTTGAATTTTAGTTGAGCACAAAGTAAAAACTCTTGCTAATGAGTAATGAAGTAAGCAAGTGAGGGTACAGTATTGGCAGCTGCTGGAATCCATTCATTAGTTGCTGGGTTTCCTCTGCAAGAAGCATGGCAACAGGATACCGGATCTGGTGCTAAGCTGAAGGATTTTCCATTGATATTCTCCATTTTAATGAACCCTGTTCCTGTCGTGTGGTGCTTCTTGTCAATGCAGTTGAGATCCTTGTAGTACTCTGTCTCCACAAAATCCTCCCCAACTTGGCCATTTCCATTGCAAACTAGCTTCTGAGGATCATTTTCGAGACGTTGAAACTTAA

>L0153

GGCTGCAGGAATTCGAATTCCTTCGTCACTGTCGAATCAATCTCAGCGAAATGAAACCACAAGAAGTAATCCAATTTCGCATCCACCGTTAATTCGTACTCCAAAACTCCTTTTCCCGCAACCGCAGTCTGGAAAAGCTTCATTGGGAAGTAATTCGGGGCCTGACCCGTGCCGGTTATTTTTTCTCGGGTGGAGAAGGAATGGAATTTGTTTGAGGTGCTTGGAGATCGAAATTCCGAATCGGACTGCCAGGAGCGGCCGAAGTCATCGGTATCGTTGCTTAACCCTGGTCCCCATTGTACGGAGCCGCAGGATAAACGGCCATAGTTGACGAGGATGAAATTGTTGCCGATTTTGGCAGAATCATACGACAGCGGATCGATTTGACGGATTTCGAGAGAGCCGATCACAGGAGGATCAGTGGCAATGCTGTAAAAACAGATATCCACCTCTCCATCTTTAACAAACGCGAATAAATCAGAGTAAGCGCCGTCACGAGCGAGATTCTCGGGCCAGGGGGAGCGCCAGGAGAAAACAAGAGTACCTTCAACGGAGACTTCGAAACTCGGAGAATGAGACTTACCGTCGTAGTTATCATAGACGGTGAAAGTACGGATGT

>L0154

ATTCCAAAGAAAACAAACCTTTCAAAAAGTATTGAGAGAGACAAAAAAATGTCAGGGATAATACACAAGATTGGTGAGACCCTCAACATAGGAGGACACAAGGAAGAGCACAAGGATGACCACAAGGGCGACCACAAGAGCGACCACAAGGGCGATCACCATCACGGAGGGTCCAAGGGACAGACTCAACACAAGGGAGGCTTAGTGGACAAGATAAAGGACAAGATCCATGGAGGTGAGCATGCTGAGGGTGATAAGAAGAAGAAGAATAAGAAGAAGGAAAAGAAGAAGGATGGACATGGCCATGTCCATGGAAGCAGCAGTAGCAGCGACAGTGATTAGATCTCATTAATATACCAATATCTAGGTGGTATGGAGTGTGAAGATAACCGATCCTTTTGTGCCAGTTTATGACAAATACAGAAATTGGTGGAAAAAGGAAAATTAATAAAGTCTCTCTCTCTCTCTCTCTCTCTCTCTCTATATATATATATGTATNGCATGGTGTGGTGTGTGCACTCCTCATAGGCAGGAGTCAATGTAAATGGAAATATTATAATGATATATGATTTGT

>L0155

ATTACAAGCTTCCTCTCCCACTCTCTCTTTCTGGCAAAAAATTCATCTATAAAAACCTCGAAGCCTCCTCTCGTTCTATTTCTCAAGGAAAACTCTCTCTCTCTCTCTCTCTCTCTCTCTCTCTCTGAGAGCTAAATGGGATTTCCTGTTGGGTACACAGAAGTTTTCTTGCCGAAGATGTTCGTGCACGCACTATCCTTTCTGGGTTTTATCAGAAACCTCATTATATGCCTTTTTAATTTTCTGGGTCTCTCGGATTTCCTCGAAACCGATAACATATGGCCCGATAATACTAACCGAATCCATAGCCACACGCCCGTTTCCGCCGTCCTGATCCGGGAAATCCTACCCGTCATAAAGTTCGACGATTTGGTCGCCGGAGACAGCGAAGTTGAGCTGCCGGAGAGTTGTGTCGTTTGTTTGTATGAGTTCGAGGGCGATGCGGAGATCAGATGGCTAAAGAATTGCAAGCACATTTTCCACCGTGCGTGTTTGGACCGTTGGATGGATCATGATAGGAACACGTGTCCGCTTTGTAGAACCTCGTTCGTGCCTGAGGAGATGCAAGAAGAGTTTAGTCAACGGTTACTGGCGGCTTCTGACGAGGGTGATTTTACAGTGAGTACAGTTCGGTCCCGGTTTTGT

>L0156

ATTCGTTTTTTTTTTTTTTTTTTTTTTTTTTTTTTTTTTTTTTTTTTTGGGACCAAAACCAACCCGCAGCTGGGACTACTGGCAGGTTTATCGTTTATAGTACAACTATCCCGCACAACGTAAAAAATGCTTAACCTAAATAGGTTAAAAAATTCAAAATACAAATTAAAGGTAGATTTTTTTTTTCTTTTATTCCTTGGCCGATCAAGATGCCAGTATGTGAAAGTCGCAGTGTGTCGTATTCTTTTTGTTGCCCAAAATTTAATCGTCTGTTGATCCAAAAAAGTGATGAGGGAATTTCGACCATACATCATCCCATGTCCTCTGCCCATCAGTCACTGACTTCACAACTGATGCTGCTTCTTCCACATTAACACGGATTTGATCCTTGCTATCCCTCTCTCTGATTGTCACAGAGGACGTTGAATCAACTGTTATTGCAAAGGGTACACCCAGTTCATCGAGAGAGAGAGAGAGAGAACTATT

>L0157

TCAATTCAACGCAGAGGCCAGAGAGAGCCGGAGACGGAGAGAGGAATTCATGGCTGCTTCCAAATTACAAGCTCTGTGGAATCACCCAGCCGGACCCAAAACCATTCACTTTTGGGCACCGACTTTTAAATGGGGCATCAGCATAGCAAATATTGCCGACTTCTCTAAACCACCTGAAAAACTTTCATATCCTCAGCAAATAGCGGTTACCGCCACTGGACTTATATGGTCACGCTACAGCACGGTAATCACACCGAAAAACTGGAATCTATTTAGTGTAAACGTTGCAATGGCTGCAACAGGCCTCTACCAACTGTCACGCAAATTACAGCATGATTACTTTCCTGATGCAGAAGCTGCTGCTGCAAAAGAATGATGACGAAAATTTGCTTAACTTCTTTTTGCGCCCTGCAGTGTTGAAGCTACTTTGCCTTGCCTAAAAATTAGTTCAACATTTCATTATGTGATTGATGTTTTCATTCCTTTGGGGCTTTGGATGCTACAATACGCTATGATGAAATTGTAGGTGGTTGTTGACTATTGTGCTTGCAAGGATATTATGAACTGTCAAGTGATCAATTGACCAATAAAGTCTTATCCAGATGCCTCTATTT

>L0158

GGCTGCAGGAATTCGAATTCCGGAATTGCTTCCTTCTCCTTCTTTCAGCGTCTGCTTACCAGAGCTTTAAACGCTGGGTTTTTTTATTTCATTCTTTAACGTAAAGATCGATCATGTCTTACTACGGCCACGAGCCTCACTTCCTTGAAGCATGTTTTCTTTGCCGGAAACCGCTTGGGTGTAACACTGATATCTTCATGTACAGAGGGAATACACCGTTTTGTAGCAAAGAGTGCAGACAGGAGCAGATAGACATGGATGAGTGCAGAAAGAAAAAGAACTGGAAACTGTCGTCTTCCTCTAGTAGATCTCTGAGGAAATCAGAGGCAGAGGAGACTTCCCCAAATAAAACCGTACGGACGGGCACTGTTGCTGTTGCTTAATTAAGGAATGCAAAGCCAATTTTGAATTTCCATGATCCAATGTGAAAATTGAGATGTTTATTCTTCTTGAATCTTGATCTGTAAAGAACTGATGGATGAGAGCTAATTAAGGTTTGAGAAATGGGAATAGAGGAATTAAGAGC

>L0159

ATTAGTTTTCTTTGGCAATTTGTACAGCGTTTGGGACGTGCAATATACAAGGAAGCTTTGCATCAGATTTCTCCTCTCCTTCCTTTCTTTGTTTTACGTTTTATGCGCATGGTTTGATTGTGTTGTTGCCGGCAGTGTAGCTTGGTTGTGTTGCTTGAAGTGTTGGGCTGTTTTCAATCCCTTGTGGAGGATTGTTGCCGTGGGTTTTGGGACCTAAGAGTGTTGGCTGAACTTGCTTTGATGAGTGCAAGTGCCGCACGGTCCTTGTAGTTGGAGTGGAAACAAAAGTGCCGTGACACAGGCACGTCGGTCGTTCCGTTACCGATTGGACGTTTGGGCTGATGGAGGGAGTTGCCACCCCTCCGCCCGCATCACATTCCGCATCCGAGAGCACAGCGACATTCCGTGGGCCACGGCAGCCAGTGAGGCATGCTTGGAGCACGGATGCCGCTACGGGGTTCACCTCGCACCCTAAACAAGGCGCAAAGCGAAAACGGGACAGCATAGCTTTCGAATTCCGCTTTCGTGGGTATGCAACACAGGAACCGAATCATGTCACCGAGGCACATTCCGCTCTCGGGCAACAACTGAAGGGGATCGAGAGCCAACAGTTCAATG

>L0160

GGCTGCAGGAATTCGAATTCGATTTGACAAAAAGAACAAACCATGAAACCGAACTTGCATCTCATGGAAGGAAGAATGCCATCGATAAGGGATACAATCTGCCTTGCAGCGGTGAAGATGAGGTGTTTTCTCTCCACAGGAGAGATAGCAGTGATCTCGATAGCCTGCAAGCTACTTCAAGTTCAGCAGGAAGTGTTTCAGAGGATGAAGTGGATAGCAAGAAGCCCTTCTACAGATTCGTTAATCCCCCTTACGTCAAACCAAAAGTGGAAAAAGAAGAAAGCAAGATTGAAGAACCCCCAAAACTAACTGGCAATGTTGTTGCTGAAGATGATTTGGTTAGTGAAGGTAACCCAAAAACAAGATCAGTCCGAAGGAGACCATTGAAGCCACCTCCAGGTCATGAAGATGTTGGCATTGTTGAAAGACCATTGAAGGCACCAACTGGCCTTGAAAAATTTGTCAGTCCTGGAAATGGTGGGCTTGCAAAGGCTAATTCAAGTGCAGTACTGAAAGAAGATGGGGCTAAGAGGGGTTCAAGAATCTCGCAAGCAGATGAGGTTGATCAAAAGGATGAAGAGGAAGAGATGATTGATGGACTTCTGATGCATTACAGCAAGGATTCAGTGAAACCATATCTAA

>L0161

ATTCGTTTTTTTTTTTTTTTTTGCAGGTATCAATTCCTTGCCACTGCTTTTTACTATATATCAATTGCAAGTACATAAGCAAGTCCAAACAATTTGTTGGTGCTTATTTTCAGCAAACAATATGATAAAGGAATAATAGTTGCAGTACATAAATAAAACAATTATTACATCTTTATTTACAGTAGATTCATTATAAACACTTACGCGAGTGGGAGATCAGAAGGGTATTCTGAAAATGGAAGAACGTTATTCAGTTTAGGATGGAAGTACTGATCTAATGTGAGAGGTTTAGGACCACCAAACCAATCAATCAAAAATATATCTTCAATGTCTAATTTGAAGAATTGGAAGTTGTGACCTTTAGGCCAGCCCTCCATCTCAGGATGTTTGGTAAATAGGGCACTTCGAGCAAATTCTATTTCCTCAGAGTCTTCCTTGAGTAACTTTAACTTTCCAGTGAGGGTAATCTTTGCACAACTTGGGTTCTCGGGGTCTTTCTTGCCACAAGTTCCTATTGGGTATTCACTGATTGTGAGGGAAGACCTCTGATCTCTCAATGCATTTCTTGCAGTTGGATCAAGCG

>L0162

ATTCGTTTTTTTTTTTTTTTTTAGAGCAGAACATCCCACATTTTTTTCAATGGTAATGCCTAAAAATGGCATTAATATGGACCCTCCACATAAGCATTCCAAAAATTCATTTAATTCATCCACAAGTCAAGTAAGACATTCAAATCCTCTTTGTCTTAAAGCCAACAAGTTATTGTTTCCTTCCTCTTCCTTTCTTCCCTGGCTGTGCTTCTGTCTGTTCTTTCCCTGTAACTTCTGACACATCAGCATCAGGGCCCCGAGACATCAAAGTGAAAAAAATATTATTCAGCTTCTCCATCTTCTTAGCATCCTGTGCCTGGTCCGTCTTGAACTTCAGACACTCTTTGTCATCAGTGACCTTGAGAACCAGCTTGCCGTCGCAGTGTCGATACTTCATAACATACCGCGTTGAATCAGGGTCCGCGCGAAACAACTGTGCCGATCGCTCAACGAACTCGTCCCACGAAGTTATGTAAACCATTTCAGTCGCAAATATAGCAGAATAAATATAGAAAAGAATTGGGGATTTCAGGAATCTGAAGGGCCGTCGATGGTCGATCAGAGATTCTGATTTGTTTTCTATCGGAGATTTTAGCCTCTTTAGGCTCTGTTTGCATGAGGCG

>L0163

GGCTGCAGGAATTCGATTCGTTTTTTTTTTTTTTTTTCAAAATTCAGAAGAATTCTTCTACAATTACAAATTTACAATGTGTTTTACGCCTTCAGAAAAGACGTATTGAACTGACAAGAGAAAAAGCAATACTATCAACAGACCTCTTCCAGGTCCCATCAGAGCAGACAAGGCCAAACCACCCTTCACATACGAATTGTGCAGCACCTCCCAAAATGAACCAAAGTCCACGCAAACGCCACGCGATTCCTACTCTTGACAAGCAACTCTTCTTTTTTCTGAACCATCTCTCGCCACTTTTTCACATTCTCTTTCACTCCGAGACCCGAAACCCTCCTCTTCGACTCGAATCCGCAATCAGTCAATCTCCGACCAAGACTATCCGCAATATCCGCCGAGGTTTCACTCTCTAAAGCCTCCGGTTTCAACTTTAACGCAGCGGTCTCGGTCAACATGTTGACCACGACAGATTCAATTCGATCATCAGCGGAAAGCAAGGACTTGACACGGGAGACACACCCACCGCACATCATTCCAGAGACGTCAAGGAGAACCTGCAAGTCTTTGGGCCGTTCAGGGACTTGAAACGGCGTGTCTTGTATTTGAGGTTTGACGTTGGAGAGAGTGAGGCGAGGAATGGCGCATGGCCGGGGGATG

>L0164

GGCTGCAGGATTCGAATTCTGAAAAGTCAGGGAAAAAAAAATGGCGTGGAGAGGCCAACTTTCTCGGAATCTTAAGGAGCTTCGGATTCTTTTGTGCCAATCTTCGCCTTCAAGTTCATCGGCTAGAGCATTCGTCGAGAAAAATTACAAGGATCTCAAGACTCTGAACCCTAAACTCCCGATCCTGATCCGTGAGGCTAGTGGAATCGAGCCTCAGTTATGGGCTAGATATGATATGGGTGTTGAGAGGTGCGTTCGATTAGAAGGTTTGTCTGAGGCGCAGATCTTTAAGGCCCTTGAAGACCTTGCAAAAGTTGGGGCATCGCCCAAAGCTTGATATCAATGCTGTTCAACTGTATGGAATGAAATAAATTGGTACTCTGGGAAGATCTTCCTATGACATATTTCCTGAGTTTTGCTATGTTTGCTTTGACAGATGCTCACCGTTATCATTTATTTTGATCACAACCAACATTATCATGTTTCTTTTTCTGTGGCCAGTGCTACTTTCCAACCCATTTAATAAAAAAAAAAAAAAAAAA

>L0165

ATTCTTTTTTTTTTGCTTTGCCTGCAAGTTGCAAGTTGCAAGTTGCAAGCTGCAAGCCATGGCGCACATTTTATTCAAGAGAATTTAGGTAACGAGGGAGTGACACAAAGCATACAACTTCATTCCTATTGAAACATATATATAAATCATAGAGCATAAAGGAAATACCAGGTTGAAGCATTTAGTGCTAAAAATAGAAAAGTTAACGAAAGAAAAAACTGGAAACAGAAGCTATGGAGATGGAGGCCAACCATATAATCCAGCATCCTCAATTCCTTCATAGAACCTGAGCTCAAGCTCTTCCAGCCTTTGCTCATCATCCCACATTTCATCGAAACTATCATCGTCATCATAATCTCCCATGTCAACAGAAAAGATATCCCAAGCAAAGTAGTCATCTGAATATTCAGAGCAATAATCTCCCCAGTCATTCATATCGTAATACTCTAGTAGATGAGGCCCCAGTACTCTCACTTTTGGGAACTTCTCCTTGAGGAAGTTACCATCAAGTTTCACATCCCAGCATCCTGTCAAATTCATGAACTTAAGCTCAGGCCAGCTTGAAAGGATCTTCAATACGCTTTCTGTGCTGATGAGG

>L0166

TTTTTTTTTTTTTTTTTGGGAGAATATAAATTTAACACTTCTCATGCAATTTGGTTTTTTTCTTTGCTAGAGTAATATGGATTTTAACCACTAAAGATTCTCTTGCATTGCACATTCGGCCCTCATTCATCAAATGAGCTTACGCACATCCCTCGGCTGGCCCATTTCTGTTATGGTCCCAACCCACAGATGGTAGACGCTATCTCTTCCAGCATAGCTGCCCAGCGAACGAGCATAGAGGAGACCAGTACCGGCCATTTCCATTACGGCCACCGCCATGCCTCCAGTCCTATCTTCACTTCATATCCTAAACCCACGCCTCAGCCTCTACGTGAAGCCACTCTTCCTTCGCAAATTCCCTCTGCCCAGGCGATCAGTCCCGATAAACCCTAGAACTCTTTCCTCTGTAGCCTCCGCACAGTCTCCAACTGAAAATGGCGATAGCCGTGGCAGGTCCGGCTCCCTATCGGCTCCGCCGCCGCTTCTAGAGGAAGCTGTTCAAAAAATCGACGTCAATCCTCCAAAAGGGACTAGGGATTTTCCTCCTGAAGAAATGCGCCTCCGTAACTGGCTGTCCACAATTTCAGAGAGGTTAGAGCTCTACCTCCCTCACTCTACATATATGTACCCAAAAACCCCGATATGTAT

>L0167

ATTCCCAAATTTAGTCCTGCCATTGAACCCTAATCAACAGAGAATTTGTGATGGCTTCAAAGCGGATTAACAAGGAATTGAAGGACCTGCAGCGTGACCCTCCAGCTTCATGCAGTGCTGGACCAGTTGCTGATGACATGTTCCACTGGCAAGCAACAATCATGGGTCCAGCTGATAGCCCATTTGCTGGTGGTGTGTTTCTTGTATCAATTCACTTTCCTCCTGATTATCCTTTCAAGCCACCCAAGGTTTCCTTCCGTACAAAGGTTTTCCATCCCAACATCAACAGTAATGGAAGCATTTGTCTTGACATTCTCAAAGAGCAATGGAGCCCTGCCCTTACTATTTCCAAGGTTCTTTTGTCAATCTGCTCTCTCCTGACTGATCCCAACCCAGATGATCCATTGGTGCCTGAGATTGCTCACATGTACAAGACGGACAGAGCCAAATATGAGACTACTGCTCGATCCTGGACCCAGAAATATGCAATGGGTTAGGGCTCCACACTTGCCTGATGAGCGTAATATGCTTTGTCCTATTAGTAAATAATGGAAACTTCTGGAGCAACGTTGTCTTTGTATTCGAATATGATATTGGAGCTCTTTTCATAGTGAGACATCATATTGAATAGTGGAATATTATGTGAAGTTTCATG

>L0168

GCTGCAGGAATTCGATTCCACCTTAGGAATCCAGATATATCCTTATTTGTATGTACGATTTAGCTTTTAGGGGTTTTATTTTCCCTCTTTCTGCCGGATTTTGCGGTATCCCCTACCCCTTAAGGGCAGGGGACGACTCCTTTGCCCCCTGCCCGAAAGTGGGTTAGCACCTCCCCGCATCTGGGTGATTTGTACATATTTGTTTTGTGTTGTTCCTTATAGTACAGATTGGTGGTTTATAGAGAGGGTTAAGGTTAAGGATTTGCTATTTGGTGTTTCTGAGTTGTTTTTTTGTTGCTCTTTAGTGCAGTGCTTCACATATGAAATGTAGGAGAAGGGTTTATTCTCAGGGTCCTTCTATTGACACCCATGGTGTTTTTCCGACCTTAAAGCTAAATGAGAATGAGAGGCTGGGAGCTCTGAATCTCTTCTCCTTAAAGCGTTGAAGGGAAAGGTTGCTACAACATCCGGAGATTGGGCTTCTCTTGCTATGATGGGTCTTCTTTTTGGTTCAATCCTTGCCATGTATGACTG

>L0169

GGCTGCAGGAATTCGAATTCGAATTCTTTTTTTTTTTGTTCAAAAGAGCTGTAAGTTTATATTTTATTACCAGTAATTGTTGACCGTATGTAATACAATCTTTAATGCTTTTAATAGAGAAGAGAGAGAGAGAGAAAGAGAGAGAGAAAGATGGCAAAACCTAGCTTCACATGGAAGCCTATGTTGTGATGAATAGTATTAACTCAGCATCAGTAAAATTTTCTGTGGTCATTTTTATTTTCAATCTGTACATTAATTAATTTACACAAACCTCAATCCAGAAATGGAAATGGACTAGGCTCCTCTTCAGTTTCAGAAGCTTCACAGGGCCTTCTGATCTAGCCTCTCATTCCAGTTCAGGCAGCGAATCAAGCTGTGAAACCAATCACCTGTTTGGTCAGATTTGTTGACTGTTGGGAGTGGGTGCTGGCTCATAGATATCTGGACAGAATCCCCTCTTGAAAGTTGCTGCCTTCTCTTCCCATCAAAAGAAACCCATACATTGCTTCGAGCATCATCTGGAATCTTTAATTCCAGCCTAGCAGAATCCGGAAGTATGACCGGCCTAAATGAGAGAGAGTGTGGACAGATTGGGGTAAACAGCATACAAGGAACATTTGGATGCACCATGGAACCTCCTGCAGCTGTAGAATAAGCAGTACTTCCAGTAGGTGTGGCTATTATGAGAGAGAGAGAGAGACT

>L0170

GGCTGCAGGAATTCGAATTCTTTTTTTTTTTTGGGGAATAAAAATTTAGTTCAAAGAAAACTCTAATATACTCAACAATTTAATAAACAACAAAGGTTTAAAAATTCCTGAGTGAGATTAATAAGGCAGCAAAAAATAATAATGTTCATCCAGCTTCTAAACAAACAATAATAACTCTCTCAAAACTAAAAAGCACATAACAAGTTTAAACTTGGCTTACAAAACAAGTAACACAGATCGCTCCAAGTCAACAATGAACTCAATCTGCCATCTTGTGACACTCATATGTTGCTGGGGTACATGAAGACTCTAACCCTATCTCCCATTGATTTTGGGGGCAGATTGGACTTGAACTTAGCTCGTACAACACCACTGTTACCATGAGGCCTGGTGACCTTGCCCCAAATGCAGCGATAGTGTGATCCATTCTTCTTTACCTTAGCCTTGTATATATAAGCCATGCGCTTTCCAGCATACCAGGCAACCTCTTCCTTGGTATTCACTCCTTCAATCTGGATTAATGACGTGTTTGGATACTGGTTTGACTTGGACCTCTTGTATCCGAGGATGGTCCCTCTGACGTAGAGTCTGACTCGCTCTCCTTGGCGTCCCTTCACCATTTTCGGCCAAGCCCTCTTCTCTCTCGCTGAAAGTGGCTA

>L0171

GGCTGCAGGAATTCGAATTTGCCAACTTGCTACTCCTACAGTTCCATCTGCACCTTCAAGGAGCCCAAACAATACTAAGCTCTCCTCTGTGGAACACAAGTCAGCGAACCTGAATTTGATTTCAGGGTTCAATGTGGAAAAGAGACCTTTGTCTCAAACCCAGAGTCGAAATGACTTTTTTAACCTCTTGAAAAAGAAAACCTCAACAAACACTTCTGCAGCTCTCCCAGATTCAGCCTCTGCTGTTTCATCTCCTGCAAGTGAGAAATCTTGTGATGCGAGTAAGGAAGTAGTCAGTGCTTCTACAAGTTCTCAGGCTATTAAGAATGGTGCTGAGTTGACTAGCAATGGTGATACATGTGAAGAGGTTCAGAGATTTTCTGAGGAAGAAGCTGCATTTCTTCGTTCTCTTGGGTGGGAAGAAAATTCTGGGGAGGACGAGGGCCTTACAGAAGATGAGATTAATGCTTTCTATCAGCAGTGCATGAAATTGAGGCCGACCTTGAAACTCTGTCCAGGCATGCAGCAAAAGCTGCTTGAATCTCATGCAGCTGGTACGGGTGGAGCTTCCTCTGAACTGAGCTCTTCTGACTCTGGATTGGAAACTTGATGCTGTTCCC

>L0172

GGCTGCAGGAATTCGAATTCCATGTATTAAGCTTTGCTCTTCAAGAAAGAAACATACAGATATATACATGGCTCCTATCACTACCCTCTTCTTCTTCCTTTTCTCTTCTTTTCATCTTCTCACAAGCCCTCAACTTTTAGCATTCCATGTTTCAACTTTCCAGGAAGCTTCATCAAGATTTGGTTTTGCTCTCATGGATGTTCCTACCTTCTTGTGATCTGATGGCTTTGTGTGCTGGGACTTCAAGCACTGTACCCCTCATCTCAGTCTTGCTGCTTCTGAATGTCATGTATCTCTCTCTATGTTATTTCGTGCATCCGTTGTATGTATGCCAAATAGATTTTGATTATGGTGAGATTTATGAGCTTCACTATGATCAATGGTTATGATAATGGTTATAATGAAATTTTGTGCTCTAAATTTTTTTTTTGTCATGGTTTTATTTTTTGCCAAATTTTATTTTTATATTTCTTTTCCTTGAATTATACCTTCAACTTAATTTTTCAGTTTTGGATTCTGTAATTACATAATTAAAAAAAAAAAAAAAAAGCGCGCTCACTGGCCGTCGTT

>L0173

ATTCCTTTACAACGTTGAAGGGATCAGGATATTGTGGCCTTCTTCTGGGAAGCCCATTTATTCTTTATGAGATTATAGCCTTTGTTCTTCCAGGTTTGACAAGAGCGGAAAGAAGGTTCTTGGGGCCCATTGTTTTGGGCTCCTCAGTACTGTTTTATGCTGGAATTTTCTTCTCCTATTCGGTTCTAACTCCAGCAGCTTTAAATTTTTTTGTTAGTTATGCAGAAGGGGCTGTGGAATCTTTATGGTCTATTGACCAATATTTTGAGTTTGTGCTTGTGCTAATGTTCAGCACAGGTCTGTCTTTTCAGGTTCCTGTTATACAATTTCTACTCGGACAAGTTGGTCTAGTGACAGGGGACCAAATGCTATCAATTTGGAGATACGTGGTTGTTGGGGCGGTTGTTGCAGCTGCTGTACTCACACCATCAACTGATCCTCTTACTCAGGTGCTTCTAGCTGCACCGCTTCTCGGTCTTTATTTGGGTGGTGCATGGATGGTGAAGCTCACAGGCCGATGATAAAGTTTTTAACATTCGTCAAGCGCAGGAGATTTACTCTTAGTGGTCAGTGCATTTAGAGGGCAGAATGGCCCCTGTATAGTTGCCCAAAGGTCGTAACATATTAGTAAATTGGATATCAGGACGAATCATGGATATTTATTCTTGAAATCTCT

>L0174

GGCTGCAGGAATTCGAATTCCTCAAAAGCCTTATCTTTGCTTCTATCTCCACCATTTTATCTCTCATCTTCCTCGCTTTTTCTGACTCCAGCTGCTGCTCTAGTCTGCTCAGCCTCCATATCGCTTCCTTCTTGTGCTGCACCCAGTTTTGCCTACCTTCCTCCATCACCTTGCAGTACTGTATCAGAGTACCCATATTCTGACCCCGAGTGAACCTCGTCAGCTCGGGGTCCATACCCGGATCCGACGGTGACAGAGAAAGACTTACGGACAGGGACGGCGTTAGTGATGCTGTTGTCGTGGTTGAAAATGACAAGGACGATGCCGCGTTCATCCAAACCGATCCAAGGGAAAGAACAGGATCCGGATCGGACATGAAAGAAACACTATTTAAACCAGCATCACCAGGCAATGTTGCAGGATAACAGAGAGATGATTGAAACTTCAGAATATTAGATTGAACGTACTTCTCAGCAAAAGTATCCAAAATATGATCGTATCTGCCCTGACCGAGTTTATCACCGATGACCGGAATATTCCCTTGTGGGTTCAACTTGGATTGCTTTTCTTTGAAAACTTCCCACCACTTGCCGAGCCGCTTGGCAGTGCGGCCAGGGAGTTGGGCGGCGATCTTTTTCCACCT

>L0175

AAATCTGCCTCAGGAAATGTCTCATCTTCAAGGATTGCGGGGATCTTACATCAGGTCTAAAGGTTATCATACTCTGATCCTGCGCCCTGTGCATATCATAAAGATCTGTCTGACAATGACAAATGCCATCTCAA

>L0176

GGCTGCAGGAATTCGAATTCCACCGCCATACAATGCGGTAACTCATCCTCCATCCCAATTGCGGGATCCGCCAGCTTATACGGTTTTATATCCTTAAAATCCGCCACACAAAATAACTTCCCGGCCTCCGTCAAGCACACTACACCGTTCCCTCAAAACACGCAGTCTGCTCAAAGCACTCTTTCCCCATAGAATAATTGGGTTCGAGACACTCGGCATTGATATTGTACCGATAAATCGTACCATCTTGGACTATGCAGATTTGGGTTTGATCATCGGTCCATGACATACCGATTAGGCGGCCTTCGGGGTGCTTCCAGACGGTTTCGGCGATGAGGATGCCAGCGGAGTTGAAGATGCGAAGCTTGCGGAGGGCAGATTCGGAATAGAGCTGGACGATCTTGGAGTCGTGAGGGATAATGGCGATTGGGCCGCCGAATGGAGCGCAGGCCACCTTGTTGCGGCTCAAGTCCACGTGTTTCCACAACATTTGGTATATCTCCAGTTTCCGGTAGAAGCGATTGCCGAGCAGCTGCCACTCCGCCGCGACGGAGAAGTTCGCCATTGCATAGAGAGATAAAACGGAACTTTACTTGATGAATCAAATGCTCTACCTACGATTTTGAG

>L0177

GGCTGCAGGAATTCGAATTCATTCAAAAACTAACACAGAAGAAAAAAAAAAAAAAAAAACTTTTGACAATAACATTAAACCCGATATGATATCAGCATTCTGTAAATAATTTCTCCATCCGAGTATGGGTTGCTCCACTATTTATGCAAACGCTTTGATTCATATGCAATTTTATTGGTAACCTAACAACTGGGACACTTGTTAATCCACCCCTCACCTCTTTAAATGTTTGCAGTTATTTGGTGTATCCGCCACAGCTCCATAATAAATCCACACACCCGAATTGCAAAACCCCCCAAGAAAAAAAAAAAAAAAAACCAAGCCCAACCCTTTCTTTTCTTTTATATCCTCAGAAACTGTACGGTCCTTCTATGGATGTGGGCGTCTTCTCAAAAAGGAAAATTCCCAGAAAAATGGATCTTCATCAAACACCTTGCTCTGTCGTCGTTTCTCCTCAAAAAGCTTACAAAAATAAGCATAAAAAAAATAAAGGGGGTGAG

>L0178

GGAGGTGGTCATGGAGGTGGACATGCAAGCCATTCCACTGGAAGCAGCGCACATGGCGGAAAGATTAATGGTCATGAGGGATACAGGCATGGTTCAACTGTCGTTCCTCTCTATGCTGCTGGGGCTATGAGCCATCACCAAAATAATAATCACCAGCGCCATGGTTCTAATGAAGGCTCCCAGAGCTACGCTGGTTCCAGTTATTTGGTTCTCGCAGCCTTGGCCGTTATCTTCTCAAGTATTTTCCGTTAGCTTACAATTGTAGTTTTTGTACATCATGTCTTTAAATATTACGAAATGCATAAATAATGCCTCGCTTGGCTTGCTCTAAAAAAAAAAAAAAAAAAAAAAAAAAAAAAAAAAAAAAAAAAAAAAAAAAAAAAAAAAAAAAAAAAAAAAGAAAAAAAAAAAAAAAAAAAAAAAAAAA

>L0179

GGCTGCAGGAATTCGAATTCCTAGCTTCAAAGAGAAAGAAAGCAGCAATGAATGCCCCTGACCGCTACGAGCGATTCGTCGTTCCTGAGGGCACTAAAAAGGTTTCCTATGAGAGAGACACGAAGATCATAAATGCGGCATCGTTCACTGTAGAGAGGGAGGACCACACCATCGGCAACATTCTTCGCATGCAGTTGCACAGAGACGAAAATGTTCTCTTTGCTGGTTACAAGCTCCCTCATCCTCTCCAGTATAAAATCATTGTTAGGATTCATACGACTAGCCAGTCTTCACCAATGCAGGCATATAACCAGGCTATCAATGATCTAGACAAGGAACTTGACCATTTGAAGAATGCATTTGAGGCTGAGATAGCAAAATTTTCTCAGGATTACTAGCACTTGACGCAATTAAGAACCAAAGTGAACAGACATTCAGTTGATTGTGCTTAATGGAATTTAGCGGAAAAGGAGCGTGATTTCAATGAGACTTTGATAACTTTGGTGTTGTATTTAATTAATGAATCATGGCATATCATCAAATATTAAATGTATGACAGATGTCTATCAAGTAATGGCATGCAGATCTGTTTTGT

>L0180

ATTCATTTCTTCAAGTACGTGGCTTCGTCGCCTTCGAATAGTAGCAGCAACTCTTCTTCTCTTAACACGTCGAAGAGGCATTGCAATGACGGTGAAGGAATTGTCAAATGCGACCAACCTTGCTTAAAGAAGATGCGAGAAGCACCTGAAGATCAAGCCAAAGCTGCTAATAAGAATGAGGCAGAGAATAGTGGACCAGTAATTTGTGAAAAGGACATTCGTAAATTTCGTGTTCCTAATGATAATTTGCCATTGACCTTCCGGCTTTTGAAAGTGCAAGGGCTGCCAGCTTGGGCAAATACCTCTTGCGTTTCTATAACCGATGTGATTCAGGGGGATATTCTTGTTGCTATCCTTTCAAACTACATGGTGGACGTTGATTGGTTGATGTCTGCATGCCCTACACTTGCGAAAATTCGTCATGTCTTGGTTATTCATGGAGAGGGTGACGGTACAGTTGAACATATGAAGAGAAGCAAGCCTGCAAGTTGGATTCTGCATAAACCCCCACTACCCATTTCATTTGGGACACACCATTCAAAGGCCATGCTTCTAGTCTAT

>L0181

GGCTGCAGGAATTCGAATTCTTTTCATACTAATTCGAAAATTTTTTATTTCAAAAATAAAGTAAGTCAAAACTTGCACTACATCCATGAAGAATTTATCAGTTAACCATCCATAGATTGCTTTTTCTTTTTTTTTTTTTAATATAATTTTCTATCCTTAAGAGACTCCAAAGCACTAAGAGGTACACTGGGGCAGTGAAAAATGAAAGTGAGAGAATTACAGAGTCATTCCTCTAGATTCTGCTTCAAGATATTTGCAGATCCTCTCCATAACCTCTCTACCCTTTGCACTATTATGCAAGAACTTCTCAACACAACGTTGTTCTATCTTCTCTGCCATTGATGCCAACGCAGATAATGGCTTGATCCGTATACTTGTCTCCTGCTGACAAAGTGTCCACTCACTAGGATTTTCAGGGTGAGGGTTGTAGCGAATCTTCTCCTCAACTTCTATGAACTTCTGGAGACTGATGTTACGACTGGTAAGCTGCATTGACTGAGTTTGAGCATCAACAACTGTTGATTCAACACAATGACAAATGTCCTGACCAATAATTTTACGAATGAACCATGGTCCAGGAGCATGCACTGTAACAGCACGAGTTGTGTAAAGTTTCCCAGATTCCCGATCAAGCTTCCAGTTCAGAGTGTCGACTTCAAGGATATGGGAT

>L0182

ATTCCCATTTTTGCCTCTGTAAATCCAGAGGAACCAGCTTTAACAGAAACCATGCCATATTATACCAGAAACAAAGACGACATTAATTGTTTCAATGAGTATAATCCGAGACCATATATAGGTGGATATGACATGGCCTTGACGTACGGACGTCCCATTTCAGCATCTGAGGAGACCTGCTACCCAAGGAGCTCCTCCGCCAATGAGATCGACTATGATTATCCCGACTTCACCTGGTACGTTGAGCCATCCGCCTACAACGATGATCATCTGCAGGAGGAGTACACTAGCTATGCTCGTCCCAAGCCCCGACCTGCTCCTAACAGTCTTGGAAGTTGCCCTATTCTAGGGGGAAACGATGAAGAGAAGAAACATCACCACTGCCACCACCAACACGAGAAGCACTGTGATGACGAGTAATAGGGATTCATGCCATTATTTGCTACTAAATAAAGGATTGAGGCATGTTTCCTTTCGTGTAAGAGCTCTTTGTGAACTTGTGTGTTTCCTGCAGTGCAATGGATCATCTGTTACTAGTATTCATAACACCAGTAGCTAGTATCTATTAGGCATGTTTCGTCTTTAATGGTGGTTCCGTTGTGCAATCTATGTGAACATTGTCTACTATAAGTTAATATAAACAAAAAGTTTAGTGCTCA

>L0183

GGCTGCAGGAATTCGAATTCCTCCCAACGATTCAGTCCATAAATTTAACGAAGAAGATGAATCCACATTATCTTCACCCACTTTTTCCTCTTCTATAAAGTTTGGCATGCCGGAGGCTTTGGATCACGTGCGCAATCTAACCGATGTCGGCGCCATGACGCGGCTGCTCCATGAGTGCATCGCTTACCAGCGGGCTCTCGATCTCGACCTTGACAACCTCCTCGCCCAACGCACAGATCTCGACAAGTACCTCCTCCACCTCCAGAAATCCGCGGAGGTGTTAAACATCGTCAAGGCGGATTCCGACCACATGCTTTCCAATGTCCGCTCCACATGTGACCTCGCCGACCATGTCAGCGCCAAGGTTCGGGAGCTTGACCTTGCCCAATCGCGGGTTAATACTACGCTTCTACGCATTGATGCGATTGTGGAAAGAGGGAATTGCATCGAGGGGGTGAAAAACGCTCTCGAAGCAGAGGATTACGAGGCGGCCTCGAAGTGTGTGCAGACATTTCTGCAGATTGATGCGAAATATAAGGATTCCGGATCGGACCAGAGAGATCAGTTGCTAGCGTCGAAGAAGCAGCTGGAAGGAATAGTGAGGAAGCGGCTTTCAGCTGCAGTGGATCAGCGGGACCATCCAACAATTCTAAGGGTTGTTAGGTTGT

>L0184

GGCTGCAGGAATTCGAATTCCTAAATTAACAAGTTTTTCAATTAACAAAATCTCTTTAAATTCAAAGACACACTGTAAATTAAATCATTTCAAAACCTAAGCTTGGCTCCTCAAACCTCTTCTTGATTACTTCGGCAGCCGTAACCTTCATGTCCTTGGTCACTAGCGCTTCAGGACTCACCACATCCTTCAAATCCACATCCAGAGTGTATCTCGTAGGCATCACATGGCTGTAGTTCAACACCTTGATAAGCGCCTTCACGCGCGACTCCTTCGCTGTCTTCTTCGCTGAGTCTTTATTGATCACCTTCGATGGGTACTTCGATATTCCAGCGACCAGGCAGTGGCCGTATGGGCGATCACGGGTCCCGTCATCGAAGAATCGGAAAATCACCGCCTTCCGGCGGGCGGAGCGACCCTGGAGGAGGATAACCGCCTAAATGGGTTTCAAGAACTTGACCATCCTCGTTTCAGGCGGAGGCGAGCGAAAGGTTTCGAGATTTCTCTCTCTCTCT

>L0185

GGCTGCAGGAATTCGAATTCCTTTTTACTTTAGATCCCTTGCACTCCGACACAAAAACCATCTCCTGCTCCTCCACCTCTTCTTCTTCAGTCCCTGCTGCTTCTTATTTAAACAAGCCACTTCTCAACCACAGTACTACCACAACACTCGCAAATCGCTTGCATCGTCACCAGGTATGGAATCCCCTGTGTTTCTCTTTCTTTTTCCTTCTCTTCGTCTCTTTTACCTTAACCTGTTTTGTTCGCTGAAATCATATTGTTTGGCATATTATTGAGCAAATTCTTCTGGGTTTTCTTTTTCTCTTAAATATTTAAGCATATTTTAGTGGTGGGGCATTGATGAATTTGACGGTAATTGCTTGGCATATCTTGAATTCATAATTTCCTTAAATTCTGCAAAAAGTGTTTGGGAGTCCACTCTTTTCATAGGATGGGTTGAATGGGTTAGTGATGTATTCATTGTTGAGGGCCCAATTTTAACCATACTGGTGAATTGTAGCATTCAAGGTGGTACAAAAACCTCAAAAATGGCTTTGGATTGGCGCGCTCACTGG

>L0186

GGCTGCAGGAATTCGAATTCCAAAACGATGGCTTCAGATCCCAAAATTCACAAATTCGATGAGGTTGCAAAGCACAATAAAACTAAGGATTGTTGGCTCATTATCTCTGGCAAGGTGTATGATGTCACCCCCTTCATGGATGACCATCCTGGAGGAGATGAAGTTCTGCTGTCATCAACTGGGAAAGATGCAACAAATGATTTTGAAGATGTGGGTCACAGTGATTCTGCTAGAGAAATGATGGCAAAATACTACATTGGTGAAATAGATGCATCAACTGTTCCAGCAAAACGTACCCACATTCCTCCACAGCAAACTTCCTACAATCACAATAATAGATCAGAGTTTTTTATTAAGATCTTGCAGTTTCTCGTGCCTCTTTTGATCTTAGGCTTAGCCTTTGCAGTTCGACACTTCACCAAGAAAGAGTAGGCTAATTACGTCATATTAGTTCAACATTTTAGGTTTCTTCTTCTTCTTCTTCCTTTTTTTTCTTTTTTTCTTTTTTTCTGCAATGTCTGTTGAAAAATTAAGCAGCGGTTCAAACTTGGTCCCCTGAAGGCCTGTCTGTGACTGTATTTGGAACATCTGTTTCATTGATAATAGGACAATATTTGCTTTTTCAAAAA

>L0187

ATTCCTTTTTTTTTTTTTTTTTCCGAAGAAAACTAGTAAATATCTTCCATATGAACAAATCAAGAAAATCTTATACAAAATGTGACAGAAAAAAAAAAGAGCGAATGTGAAATGAAACAAAGGACGAAAGAGAAAATCTCATAAGAGCAAATCACTCTTGTAAAATTATGCAGCATCTTTTTTATTTTTATTTTTTTTTTAATACATACTAGCTACAGATAAGGAAAACATCAAAGCTAATCCAAAAGAAGGAATTTTTAACTCTGATGAGCTCATCTCCCACCAGATAGCTCAATAGCAAGCTGGTTATATGATGAATCATCTCCCCACTCAAATCTTCTCTGCAATCTTTCATATTCTTGCCTGCGTGTATCCTCAACATGCTGCCATTCTTCCCATCTCTCGGCCAGACCCTCTCCTTCCAGCATCCGTACAACCTCTGACATTGCTGGACGGTCTTCCGGTGATGCTTGGGTACAAAGTAATGCAACTTGGATCATCATTTCCACTTCCTGGATATTGTAATGTTCATTAAGGTTGCAATCTACGATATCATCCAGTCTTTTCTCCCT

>L0188

GGCTGCAGGAATTCGAATTCATCTGTTTCTTTACTGACTGCAGAGCAACTCGGACCGTTAACAAAATCTGTAAGATTCTCATAGCCCAGAAAAATTCCTGAGGGTAGTGTTTTGTAGTCTGCAGACACACGTGGTAAGAATAAGGGCTTAGGGAAAGAACCAGGCCTTGATGAAGATTGCCAGGGCTAGCTGCAAAAAGAAGGCTGCAAACACCACTATTGTAAGATCATTCCCCTCAACACAAGACTTGAGGTTCACAGCAACAAAGCATGCTGAGTCAATCCCAGCAGCAAGTATAATGATCCAGTGGAGTATCTCAACTGGAATAACCAGCAGAATAGAGCTCAGAAAAAAAAAAAAAAAAA

>L0189

GGCTGCAGGTTTTTTTTTCATAAACTCAAAAAGGAAAAATCATTTGTTGAATTTCCATAATGTTACTAATCTACTTGACAGAAACGTTGATGTTTCTCTCACCGAAATGATTACCCAGCTAGGCTAACATAAACACTATTATGTCAACATACCTTAACCAGAGTCAGCCATGTGCTTGGTCTTCCTGCGTTGTGACTTCTTCTAGATGTACATTTTCTAACCCTTTTATGCCACTGTCAATTGCTGGTGTATTGTGCTCAGGGGAAGCAATTTCAGTATTTATCCCATGAAGCTGAGAACTGTCTGTTTCATCATTTTCTGAAGCATCAGAATGTGAGTCCTTGCTGGCATCGAAGGAAGCAGTAGAAGTCAGGGCTGGTTGTGGCGCTACTGAGGGATGACTCCCTCCTGTCCAAAGTGAAGGCATCCTCTTGTCATCCTCCTTCATCCTCTCAGCATATTTACTGATATCAAAACCAGAATTATCTTTGCCCCCAAATGCAGA

>L0190

ATTCCTATAGATAAATTAATAGGAACCACATAACTCAATTGAAACTAACAAATTCGAATGATTTTAGATCCCAAAACGCAAAAGTAGTCAAAAGTTTTTTTTTTCCTTTAACTTTTGGATCGAACCTTCTTTTTCGAATATCATGACAGAGTAAGCAAACACTCACCTCTTGATGTGGTTGACAAATTCTGCATCGGATACTGCAGATAGCTCAATTTGGCTAAGATCATCAACATCAATTGGAGCTGGAAGATTGAGATCTATTAAATTCATGTTGTCTTCCATCTTTGAAGAACTCCTAGCAGGAGTAGTTGCTAAACCAATTATATGAGTTCTCTTGTGACCTCCCAGAGCTTGCCCAGATGAAAATACTCTGAAACAATATGGACATTCATGAACTTTCTTCTCTGCAGTGGATGTTGAATTTCCTGCATTCTCTGGCTCCAATTTTAGTTCCTTCCCGGGTGTATAAACTTTGAGTTTCTTGTGACTTGCCCTGTGTCCACCCAGAGCTTGATAAGATTTAAATACCTTCTTACATGTTTGGCACTTATACTTG

>L0191

GGCTGCAGGAATTCGAATTCCTTTTTTTTTTTTTTTTTTTTTTTTTCACTCCATTAATATCAGCAAATTCATTAATTCATCAAGACATATAAACATACTAACAACACAGCTAGAGGCTGAACATGAAAATTACAAAAAGAAAATCACAACTAGCAATACGTCAGATTCTATTATCTCACCAAGCATACTATTAATAACATCTAAACACGGCCATCCCACAGGCAATCTGAGTTTACCTTTCCATCCTTCATCAAACCCTCAATTTCTATGGGTGGGTTAAGACCTAAACGTTGAGCACGCTCCCATCGTGCCAGCCGGATCATCCCAAGGCACGGTCCATATGCCATGCTCATGTCAAATTGCCTAAACAGTTCCTCATGTTTGTCATGATCATCTTGAAGATCCGGGGCGCCATGAGAAATGAGCGTCCTTGTCTGGGTGATATCACAGCCGATAGCTGATGCAAGTTTGGGCGATTTCTTCTAGGACTTTGTGATGCCAACTCTATTGCTCATCCTTTGCCTGTAAAAACTCTTCATGTTGCTGGATGCTGTTTCCAGAGAGAGAGAGAGAGAACTAGTGCGCGCTCACTGGCCGTCG

>L0192

ATTCTTTTTTTTTTTTTTGGACTCTAAAGGCAGATCATCTTGATGCAGCACTCAAACATAGTAGCCAGTAAATGTGAATCCTCGACCTACAATTTCTCCAGCACAAAACCATGCAAAGCACTCCAATCCAAATAAAGCAGCAATGCTAGCATCCTCAACCCTGAGCTCCTGCCTATTTTTCCACATACGCTTAACATAATCAAGCTCCTTCCAGAATGCTTCATACCGCCCAGGAATACTTGCAAGACGAGTATAAAACAACTGTTTTGACAAAAGATTGCATTTCTCAACAGTGGGTGGTTCCTTGATGTACTGCTTGTTCTGCTCTAATAGCTGCTTGTAGTAGGCAGTACCATGCTTGGCCACGAACTGCGAGGCCTGAGCAGCCTTTGATTGCAACTGAGCTAGCTTTGACGCCATTGCTTCCCTGAACTATATCTCTGTTGGCTAGT

>L0193

ATTGGCGTTCACTTTCCACTATAAATTTGCTTTCCAACTGTACTTCTAAAGTTCATGCCATGTCAAACGATTGCGCGAAGGGATCTCATCCCATCAGCAATTATAATTCTAAAGCTTAGTGATTGATGTTGGTCCTTCCTACTTCCTCATTGCACCTTAGAAATCATTGGTAAACTTATGGGGTGTCAAGTCAAGGGAACAATTATAACAGACAAGTTATCATGGCTGCCTTTCTCATATGCAGTATTTATAATACACTCTGCCAATGAGGTTGAAGACAGGCATGAGAATGGCATCTTTGAATCTTCACTTCCTTGGAAATGTGAATTCCATTGAAAAATCAGATTACCAATATCCTGTGGCGTCAAGCTCTCAAATATGCCATCAGATGCAACCACCAAATGCGTATCATTAGTAGTTAAAGGCCTCCAACCTGTATACTCGGGCTCAGCTATAACGCCATACCTTTTCAGATATACATCACCAATGGAACGAGACATGGCAAGCACTCCACTGA

>L0194

GGCTGCAGGATTCGAATTCAATTCCTATAGCTAGTTCCCTTGTCTCTTTTCTTGTACTAATTTCTTGAAATCCCACCATGTCTGATGAAGAGAAATACCCCGACCGCCCACACCACCACAAGGAAGAGGGAAAACCCGTTGAGATTGCTGTCTACACTAAGGTCTCTGAGTTCTTTGATGAAGAGAAGTACCCCGAACTAGGGGCTACTGGCGCTTATGCCTCGTATGAGAAACACGAGGTAAAGAAAGACTCAGAACATGCTTATTTATAGTCACAAGATAGAAGAGGAAGGTGCTGAGTTGCCGTCGATGAGAAATATGAGAAGAAAGAGGCCAAGAAGTAGGATGAAGAAGCTTAATTATGAAAGGAAGCAACACAATCTTTTTTAATAAAGTATGTGTGTTTATATATATAAGTAAAATATCATGACCTAAATAATTAATCTCTCTTTACAAGAGTGATCCTACTGGTTCCTTTAATTACTTTATAAGCCTTATGTGCATGTTTATAATAATAATAATAATAATAATAATAATAATAATAATAATAATAATAATAATAATAATAATTACTTAGATGTTAAAAAAAAAAAAAAAAA

>L0195

GGCTGCAGGAATTCGAATTCCTTCAAATGACCAGACTGCCCTTCCTTCCTCCCTTTTATTACTTGCAGAATCGTTGCTTTCCTTGCATTCGTTGCTGGAATCGGTGAAACCCAAGAGAATTTTGAGCATTGAAAGGAATTCGAATCGGGAATGTGGGCCTTTGATGAGCAAAAGCTTGTGGGGGGTATCGTTACTAGTTTGGTTTGAGTTTTCCTCTCCTCTTTTCCCTATTAAGGCGCTCTCCCTCCTTGTAGTGAAGTTTATGAGGAATTCAATTTCTGTTCTGGGTGCTTTTTTTTTTTTTTGTTCCAGTAATATTCATTCATTTTATCATTTTTTTTTCTTTCTGGTAAATAATAATAATCCCCCACGTCGTTTATTTCATTTATTTTGGGTCACTGATTGTGTAATCAGTTTGCTATGAGATTGATACGAGTGTTTAGGAGTAGCTTAGAGCTGAACTTCTTTGCTTAGATTCTTTAGAGAATTGAATTTCATTTTTGGGTTTTGAATTTGGAGAGCATTGGTGGAGTGGAGGAGGTTATATTGATTGATTTCTGGCTTAGAGATTTTTTCATTTTAGAATTGGAAGTGTCATTGGGTTGGCTGTCCTTCTGCGTGTGAGCATGTCTGGTGGTTCGCCTGTTGGAGGTGGATATATGAGGCAGAGACATAGCCAAGGCTATGCCTCTGGTGGTGATGATCTCGAGGGGG

>L0196

GGCTGCAGGAATTCGAATTCCCTTTTTTTTTTTTTTTATAATGAACTTACATGTTCTTTTACATTACATAAATAGCTACAATGAAACTATTAATGTTGGACAAGGGAAATCCAAAGCCTAAAACATATGATTTAAGATTAGTACTGCTTAGAAGGTTAAGCAAAAACCCAAGAAAATCAAGATTTTATGCATTTTTTATTGGTACTTGTGATCCAAAATCAATACATATCATACATGAACAAATCAGTTACTTGAACTTAGAGACTAGCATGAGATTGTGTTTCCCTTTATGCTTTGAATAATAAATGCCGGCCGATGCATCCTCTTCTTTCATCCAACCATTCGGAAGCTCAGCTATTGGATCACTGCCAAGTGGACTGAATTTGTATTAAGCACATCTTCTGATGTGTTAGCAGAACAAGCAGCAGCAAATGCTATGAAGCAGTAGCAGCGCCGTTAACTCCCGGTGCCAACTTTGGTTTCTTTTTGTGCTTGCGTTTCTTCTGTTTTTGTTTCATTCTCATTTGATCTTTAATACTCTTCTCCCATTGTTTTTTGTTTTTCTGGTACAGCAGCATTGCTG

>L0197

ATTCCTCTTACGCAACATCTCCGATCGTGAATCGGTTGGCTCGTTTTCTCTGCCCGTAGATTTCCGTAATCGCTCCCGGCGTTTTGACTCGGTTTTGGATATTAAAGATGGAACCGATGGATATTGTTGGTAAATCGAAAGAGGATGCGTCGCTTCCTAAAGCAACCATGACCAAAATTATTAAAGAGATGTTGCCCCCAGATGTTCGTGTTGCAAGAGATGCTCAAGATCTTCTGATAGAGTGTTGTGTAGAGTCCAATGAAGTATGCAGCAAAGAGGAGAAGCGGACAATTGCGCCTGAGCATGTACTCAAGGCTTTAGAGGTTCTTGGGTTTGGAGAGTACATTGAGGAGGTTTATGCTGCATATGAGCAACACAAGGTTGAGACTACGCAGGACTCTTTAAAAGGTGGTAAATGGAGCAATGGGGCAGAGATGACTGAGGAAGAAGCAGTAGCAGAGCAACAAAGGATGTTTGCTGAGGCACGTGCAAGAATGAATGGAGGCGCCATTGCTCCTAAGCAACCAGAGACTGACCGAAGTTTAGAGAGCTAACCTTAGGTGCTTTTATTTCATTAAAACCATAGGCAAACATCCTTGACTCTTCATTCTCTCTCTCTCTCTCTCTCTCTCATATAGTGTACAAAAAGTCATTTGGCAAGTGCACT

>L0198

GGCTGCAGGAATTCGAATTCAACTCCTCATTCAAAAATCAAAACCCATAGATAATAATTCGATCTTCTTCAATTAGGGACGTTTTCTTCTTCAATTCCTCCTCCAATAATTTAGCTTAGCAACCCGCTCCACCAATCTGTATTCACAATGCCCCAAAACGAGAGTATTCGCTTCACTTCATCTTCGCTCAAAATTCCTTCCCTTCCTCTACGACAGCTGCAAACACAGCCACCACCTCCCTTCCCTTTCCTTTCTCATATCTTCACCTCTCAATTAGCTAAAACAGCAATCTCTTTCACTAATTTCATCGACTCGCTCATAACTCGTTCTAGATTACACATACCTCGCCCCCCCGATTCCAAAACTCGGAGGTCTAACTTGTTTGCCAACTCACCTCTTATGTGTTTAGCTTCATTATCACTCAATCGGTCGACAGATTTAGCTCCTTCCGAGTCACATATAAAGTCGCCGATTCTCTGTTCGGCTTCATTGTCGGTGAGTCAACCTGCTGAAACGGCGTTGCCCAGCTCAGAAGCCTTGGTGACGCAGCATAAAGGGGGTGGCGGTGGCGGTGCTGGTGCTCACTCAGCTAGTCGACACGATGAGGAGAGAGTTTTGATAAGTGAAGTGTTGGTGAGGAACAAGGACGGCGAG

>L0199

GGCTGCAGGAATTCGAATTCCCAGATGGCCGCGGCGATGGAATCAGGGACGGAGGACTCGTAGTATCTCTCTAGATCTGGCAAAAGCGAAAGACCCCCTAACGTAATTCGAATAGGGGCTGACCGCTGAGCCCACTCTGGCCTCGCAGGGCGGGCCCCTGTGGTTGCGAGCTGGAGCTGCCATAGCTTATGGCTAGAGCAATAGGTGGCCCCAGCAGAGAGAACAGTAAGGATAACGAGCGCTCCGCCCGTCAAGCGGGCGGCAAGAGCAGCGGGCAAGTGCTTGGTAGGCCAACAGCCCAGTGAACCGGGCGGGGCACTCGACGAAAGGGGGGCACACTGAGCAAGTACGAGAAATTGGCCCCGCTCCACTTTATTAAAAGCAAGGACCACTACGGGAGGTCAAAGCAAGGACCTAGGGAAGTCGGGGCTCATCCCCGGTCAATATTGGATCAAACAATAGGGAGCCGTAGCACTGACCTCTTTTTTTATTGATTCAATACAATAGGGGAAAAGATCGTACAGTTCCCTACCGAGACAACAGAAACTCTCAACGGATCCTCCGCGCACTGGGCATACCTCTTCCGTGCGTCTTTCTCGTGGCGGAAACAGAACAAGAGGGAAAGGAAAGACCCGGCCGACCTGCC

>L0200

CCGGGCTGCAGGAATTCGAATTCCCAGTTTTTTTTTTTTTTTTTTTTTTTTTTTTTTTTTTTTTTTTTTTTTTTTTTAATAAAATATACTTTTTAAAAAAAGGGGAAAAAAAAAAAAAAAACATTTTTAAAGGGCCCACACACCAAATGTTTGATAATATGCACCACCCAAAATCCTGTCCAAATTTTCTACTCTATGCCCATTAAAAGGCAAATAACCCTCTCCCCGAAGTTCTGACAACTCCTGAAAAACCCCAAATGCCGAAACCCGAAAACTCAATTCCTGTAACCCCTTTTTCTAAAAAACCCCCCTCCTGAAGTGCCTAACCGCGGCCTCCCAATTTTGAAACGAAAAAAGGTCTGTCTTAAAATGATTTTCCGGGGGTTTCTTGGGAAAATGGAAATTCCCAAAAGGTCAACAAAGGCAAAAAAGGGC

>L0201

GGCTGCAGGAATTCGAATTCTTTTTTTATTACAAGCATTAAATCTTTTCAACTCTCACCAATCTAAAAAACATAGGCAGAACCAAGGGAAAATGTTGACAATAGAGAACAATTAAAAAGAAAATGACAAACAAAAACTGAAGATGCCAAAATGGCTAAACACTAGATCAGTAGAATATAACCATATTAAGCTTTAACTTTTGTTCTTATATTTTGTTGAAGGCAACAATGTCGCAGCTCTGAACATGTTCGCTGCAAGGCTCAACTGTCAGATGTTCCTCTATGAGGGAATCCACAGATACAAGGTCATCCACAATGGTGAGCATGATCTGCAATTTCTTGATGCCATAACCAACTGGAACCAGTTTTGATGCTCCCCACAGGAGGCCGGGCATCTCAACACTACGTACTGCCTCCTCCAACTTCTTCATATCAGTCTCATCATCCCAAGGCTTCACGTCCAAAAGAACAGAAGATTTTCCACTCTCTTTCTTCTTGGCGGGCTTTTTAGCTGCCTCCCTCTCTTCTGCTGCCTTCTTTTCTTCCTCCGTTTCATCACCAAAAAGATCCAAATCATCATCATCTCCATCAGCAGCAGCCTCCTTAACAGGGGCAGCAGCTTTGCCGCCAATTCTTACCCCAACAGCTTTCC

>L0202

ATTCCCAGCCAAATTTAACAGCAGATTTCTATTCTTTTTCTGTCCAAATGATTAAGATACAAATTTATTTCAATGCGTCCCTTCACACCTTACATAAAGCAAATACCTAAATTAAAGCTATCCTGATAAATGAGAAGTAAAAGAACATTCACAAATTGAGAAAAATAGCATAGAAAGTGTCACTCCTAAGCCAAGATTTCTGTAAGGACAATTCAAAGAAAGAAAGAAGGAATGAATTAATTAATTAATGAAAGAGAAAGATGAATTAATAATAAAACTACCGGGTCAATTTCAACTAATTCACGAGGTGCAACGTTGATGACATTGAGGATCTCACCTTTTGCAAGTTTGTATGTCTTATACTTCTCCATAAATCCAGGGATTTGCTCTCTCGTTTGCCTGCAAGCAGGAGTCTCCACCAAATAAACAGAAACATCTTCATTGGAATATGATTCTTCAATTCCCCTGAAAATGAGACCAACAAGAAAAACCAGCAGAAAATTAAGAAAATCCTGCTTCACAATTAGAAGCTAACCTGTAATAAAACTGAAGTTGCTTATCGAATTCGCGATCAGCTTAAGTCACATATTGGTAGCTCCAAAAACTCAGCTTAACACAAGAGGC

>L0203

GGCTGCAGGAATTCGAATTCCTTTTCGCCTTTTTGCGAGAACCTAATTAAGAAATTAAAGCTCTCCCCTTTGACTTAACATTTTTTTTCAGAAGAAATCTCTTGTATCTATCGAAATCCACTTGCTCATTTCCCTTAAGCTCTATATATATACCTTAGAAATTTCTTCTTCTTGATCTTCAGAGGTGTCTTATTCAATCCTAAAGCAAGATTCAAGAACCGGAGATGGCTACTGGGCTATTGGAAGTGCAGCTGGTGAATGCAAAAGGCCTCAGAGGCACTGATTTCTTAGGTAAGATTGATCCATATGTTATCCTGAAGTACAAAAACCAAGAGCGCGAGAGCAGTGTCGCCAGAGGTCAAGGTGGGAATCCAGTGTGGAATGAGAAACTCGCTTTCAAGGTGGAATATCCAGGGCAAGGTGAAGAGTACAAGCTCATTTTAAAAATCATGGACAAGGACACCTTCTCTGCTGATGATTTGCTTGGCCATGCTACGATATATGTGAAGGATTTGTTGGAATTAGGAGTGGAGAATGGAACTGCTGAACTTCATCCTCAAAAGTATAGCATTGTTCAATCTGATTCATGTTACAATGGAGAGATTCAAGTTGGAGTCACTTTCACCCT

>L0204

ATTCCATTCCCCTCTTCTCATGGCTCAGCAGAAGGCAGTGTTAAAGGTCTTGACCATGACCGATGATAAGACTAAGCAGAAGGCCATAGAAGCTGCTGCTGATATTTTTGGAATTGATTCGATTGCCGCGGATCTGAAGGAACAAAAGCTAACAGTGATAGGGATAATGGATCCAGTTGCAGTGGTCAAGAAGTTGAAGAAAGTAGGGAAAGTGGACATAATTTCAGTTGGCCCAGCCAAAGAAGAAAAGAAAGAAGAGAAAAAAGAAGAGAAGAAAGAGGAAAAGAAGGAAGAGAAGAAAGAAGAGAAGAAAGAAGAAAAGAAAGAGGAAAAGAAGTGACCCATTTTTCACTTGCTATAAATTATTCAAGTTTTTAAGAGAACTTTGTCCATTAAAGATCCCTTTCTTTATTTTCACATGGTTTTCAGGAATCTATGGGTACAAATATATTTCAGCAAATAGTACAAGGAGTGTTAGTTTAAGGTGAGCCTGCTTTCCTGCATGTTTTCACATGGTAGTAGTGCTAGCTCTCATGGGTTTTTGTCCATTTTCACTTTTTTTTTTTTTTTTTTGGCAATAAACTACCAAATGTAATTCCAATTTTAAAAAAAAAAAAAAAAACTCGAGGGGGGGC

>L0205

GGCTGCAGGAATTCGAATTCCTCCATAAGACAACTTGGTCCCTCTATGATCCAGCAAATGCAGCATCCTTGTAATGAATGTAAGGGTACTGGTGAGACCATTAATGATAAGGATCGTTGCCCTCAATGTAAAGGTGAAAAGGTTGTTCAGGAGAAGAAAGTGCTGGAAGTTATTGTTGAGAAGGGTATGCAAAATGGACAGAGGATTACTTTCCCTGGAGAAGCTGATGAAGCTCCTGATACTATTACAGGGGACATTGTTTTTGTCCTTCAGCAAAAGGAGCATCCTAAGTTCAAGCGAAAGGGTGATGACCTAATTGTTGATCACACTTTATCTCTTACAGAGGCACTTTGTGGCTTCCAGTTTATATTAACCCATTTAGATGGAAGACAACTCCTCATAAAATCCCAACCTGGGGAGGTAGTGAAGCCTGATCAATTCAAGGCCATAAATGATGAAGGGATGCCAATGTATCAGAGGCCATTCATGAGGGGGAAACTGTACATTCATTTCAGTGTTGATTTCCCAGACTCTCTGCCCCCTGATCAGTGCAAAGCCCTAGAGGCAGTTCTTCCCTCAAGAACATCAGTCCAGCTGTCTGACATGGAGCTGGATGAATGTGAGGAGACAACTTTACACGATGTGAACTTTGACGAGGAGATGCGAAGGAAGCAACACCAGGC

>L0206

GGCTGCAGGAATTCGAATTCCGAGTTTTTTTTTTTTTTTTGGGGTTCCTTTTCTTCAGGTTCATTAGCAGCACCCTCCTTATTAGAATCTACTGTTTCCCCCTCTCCAATGGGCTTCTCATCACCCAAGTTTTTCTCACCCTCATTGACAGCTTCCTCTGTCACCTGAGCAAGTTCATCAGCCTGGTTTCCCCAGTTTCCACGACCAGACCCCTCTCGTTTGATCTCATTTCCGCGTCCAGTCCCACTACGGCGTTCAAATTGCCGATGAGGGCGATCTCCATCGCCAACTTCCCCATTAGTGAAACCACCACCACGACCACCACTGCGGTAACCACCACCACGAGTTCCACCATATCCCCGCCTTTCAGAGGGCTTTGCAGCATCTCCATCTTCAGGGGCACCTTGACCAGCCGGTCCTCCAGCATTACTGAATGAATTCTCATTGTTGGCGTCCCGACTGTATCCACCACGACCGCGCCCATATCCGCGTCCCCCACCACGACCTCCTCCACGGCTAGCTTCATTCTTTGCCTCCCTCACAGCCTGAGCCGGAGGTACGGGCTTGGAGGGGAGCTTAAACGACTGCTTGGCTAGAGGCTGAGGCTG

>L0207

GGCTGCAGGAATTCGAATTCGGAGACTCTTCTCGCAAGCAACTTGAACTGATTCCCAATCGTCCATACCAAATCATGGGAGAAGTGAGTTTCTTGCTAGAGGAAGATATTCGGTTGGAGACTACACGAGCTAGATTTGCAAGTGTTCTTAAGTGGCATGGGGAATTGGTAGAGCGTATTTCTAGGGATCCTGACAAGATGATTTTTGAGCGCCACATAAAGAATTTGAAGCTGCACGGGCATCTCAAACTCAATCTACTTTTAGTAAATGTATCATGCAGAAGAATTGAAAAGTGTTGCATCTCAACCTCAAGTTTTACGTGTAGATGATCTCTCTCTGTCAAAAGCAAAGACTTCTCACTGCTCGCAAACCTGATCCCAATCCTCTTAAAAAATAAACTGATCTGAACCAAGCTCATTCCCATTACTGCCGCCATCTTCAAGCTTTATTTAGATGGTGAAGAGTGGAATGATGGGCTATTAGCTACAATAAGAGAGAGGGTTCACATGGAAACAGACAGAAAGCAAATTCCCAGAGACTCAAATATGATGCCAATTGAACATTTTGAGGAAAAAAAATACATATAGAGCTGGAAATAAGGTGATTTGTTGCTTAGAAGGGGCAAGGATAGGTATAAAATACGAGACATCTTTTGCAGAA

>L0208

GGCTGCAGGAATTCGAATTCCATTACTCCAGCAATGTATATGGGGTGCCTCAGACACTGAGAGTTCAAGACGAAACTTTGGGCCATCGTAATGGTGCAACACTGGCCTAAAAGCATCCTCTTCCAATGCCTTGCAGAGTTTTCTAACACAGATCCTCACCTGAGCAGGAAACCTCGACTCGCCTTTGCATTTTTTGTCTTCCCAAGCAGTTGGGTCAATGTTAGAACCCCCAAAACTTGCTGCCTCAAATATACCATGCAACTGGTGAGTAGCGTAGTTGTAAAGAAAGAGTGGCAAGCCAGGTGTTATTGCCCGAACAGAATCCCGATATCTTGGTGGTAAACCAAATAGCTGGCGCTTCAAGTCTTCCTGCATAGTGTCATTGTTACAGACAAAGATGTATCCTCCAAGGACCTCGTTTCGTGGAAGTGTCTCTGTCGCAGGCAGAGTCTTGAACCTTTTGTCAACTGCATTATTTGCACTGTTCTCATTGTTGTTATTGCTGTTATTGTTGTTGTTGTTGGGCTCTTTGATATTCTGACTGTTACCACTATACTTGCTACCAGTCAGAATTCCAATATTGTTTATGTTGTTTTTCTGGTACACTGCATTCATATTGTAAACACCATTTCTGAAACAACTTTGCCCACATTTTCTGTCATC

>L0209

GGCTGCAGGAATTCGAATTCGTTTTTTTTTTTTTTTTTAAATCCAACAAAACATAACTTAATTTCATCAAATCTCAAGAGTTTCTCTCATAATTCTCTTAAATACATCCTCATTATTGTAAATGAACATGGGGAGGCAAAGAAACTGATCAACATTCAGATATTCATCTCCTAATATATTCATGAAACTCTTTCTTAGTAGGGTATTCTAATCAAGCATCACAATATTGATTAACCTCACTTCGATTTCCAAGGTAAAAAGATGTCAATACCCATCTACAGCATACAGTTTTGAGCCCCTTGACTGCATTACATCAATTCTTTCTCCCTTCACATAAAATTATGTGATATCCATGAGTTGATTTAAACGGTGCACTGGTAGCTCCAACAACTGTGCTGAAGGCAACTTCCTGGAATGGACCAGCCATCTTTCCACGTGGAAACCATCCAAGGTCTCCACCCTTCTTTCCAGAAGGACACTCTGAATATTCTGCTGCCAGCTTTGCAAACTCAGCAGGTGGGACTTTGTCTCCATTGCTAAGCCAACCATCTTGCAGCTTCTTGTATGCTTCATTGATTTTACCTTGCTTCTCACATAAGATATGCCTTGCTTTTACATATGTGCAGGTGCCAAGTCCATCTGCTTTTCCAGCTTTCC

>L0210

GGCTGCAGGAATTCGAATTCCTCTCCCTCAGTTCATCCTTTGCCTTACCTAGCAAAACCATACTCGGCCATTAATTAATTTGATCATAGCCCATGTCGAGTGAAAGAGGAAAAGATTTAGCACAAGGATCCAATGAAGACCATCAACAGTCTCAGTCTTCTGCTGCTACTCCAAGCCGTTATGAATCTCAGAAAAGAAGAGATTGGAATACTTTTGGTCAGTACTTGAAGAATCAGAGGCCTCCTGTACCTCTTTCACAGTGCAATTGCAACCATGTCCTTGACTTCCTCAGATATCTAGATCAGTTTGGCAAGACTAAGGTCCATCTTCAAGGGTGCATGTTTTATGGGCAGCCTGAGCCTCCGGCTCCTTGTACCTGCCCTCTCAGGCAAGCTTGGGGCAGCCTCGATGCCCTCATCGGTAGGCTGCGAGCTGCTTATGAAGAAAATGGAGGTCCTCCTGAGACTAACCCTTTTGCCAGTGGTGCTATTCGTGTGTATCTAAGGGAGGTTAGGGACTGTCAAGCTAAGGCAAGAGGTATTCCTTACAAAAAGAAGAAAAAGAAGCCTAGTCCAAGCACAGGGAATGATGAATCAAGCTCTGCCATGCACTTCTAGTATCTTTTATGTTCATCCTTTGCGCTTTCTCTGGATATTTTACTATATGGTAAATTTTTGTTCATTATTATTGCTTTTCTCTTAATTTTCTTTTGTGTCT

>L0211

GGCTGCAGGAATTCGAATTCATCTTTGAGACCTCAATCTCCGCAACTCCTCCGTTAAACCCATAACCAAACCAACTAAACTCCATATTTTTCTCTGATGCCGGAATCCACCCTCCTACGCCGTTTCCTCCTCTTTCAATCGGCACTGTTATTCTGCAACCGCTGCTCTACAAGGACATCTATCTCAGCCGGCCACATACTTCGGTCTCTCTACACCACCACCACCCACTCCTCACTCAAAGACCAAAACTATAAAGACAACGTTAAAAAGAAAGGCAAATGGTTCACTTTGCCTCCGTATACTTCTACAATCAGTGGCTCCGTCTTGGGAAAAGCGCTATCGGCAAGCACTGCAGCGAAAGTGGCCACTGAAACGACGGCGCTTAAGTGGGTCCTTCATTGCTGCCCGGAACTCCCCAGAAGCCTTGTACAGAAACTATTTCGCTTAAGACAGGTTAGAAGAGAGTCCTCTAATCCAGGAGATCAAGCAGAGGAACAAAGACTTAAAAGGGTGGCAGCTAAGGACTTGCTGGATGTAGGAGATAGGATCGTTCTTCCTATTACGGTTCAAGCATTGCCCTTTGAGTCTGAGAAACAACAGTATTCTTACAATGAAGAAGAAATAAACTTTATTCGT

>L0212

ATTCGTTTTTTTTTTTTTTTTTACAAAAACGTTCACAATTGACATTAACCATGAAAACAAGAGTTATTATTGCAAAGCCAAAACTAGCAGATAAATATGAGCAAGTTAATCAGTGCAAAAGCTATTATTGACTCTCATGCTGACCACTCCTACCTTCTTTCTTGACGCTTCTGCATCGTTTCTGCCAGAGACTGATGTGCATCCCACTTGCTTCTATCACGATTTCTTTGATCCTTCCTGGTCCGGGCATTAGTGCTAGTTCGCTGTCCTCTGCATGGCAACAGGCAAACATGATTGAATCCTCTGTAGCACCGAACATCTACCAATTTCTGTATATCACTTCTAACACATCGCTTTAGGTCTTCTCCAATCAAGTACTTAGAGACTTCATCTCTAAGAGAGTGGAGCTCCAATCCAGTCAAGTCCTTGGCAAATTTGTTCCCTATGCTAAGGTCATTTAGGATTTGGTGAGCTCTTTGGCGGCCAATTCCCTGGATGTGCTGAAGAGCAAACCTCAAGCGCTTGTCATTTGGGATTTCAGCATTACCAATGCGAAGGCATTGGACCCTTTGGCAACGAAACGACAAATTTTGAAAGAGAGCTCGACTAATATTGTCGAGTAATCCAAAAGA

>L0213

ATTCCTTGATTCCCCTCTCTCCTCCTTCTCCTTCTTCTTCTTCTTCTTCTTCTTCTTCTTCTTCTTCTTCTTCTTTACACCTCCCCATTTTCAATCTCTTTTCTCATTCTTCTTTTTCTTTCATTCTCTTCACACGCATGTTTTCCTCTTTAAAATTTCCCTTCATCACATCTCTCTCTCTCTCTCTCTCCCTCTCTTGTTTTCTGGTTGGTGATTTATAGATTCTGTGTCAGAGAAAGAGGACAGAATTATAATTTTCCTTTTGCCTATCTGATTTCTGAGCAAAGCCCCTTTGTGTCAGAAACAAAATCTCTCATCTCCAACTGTTTACTTGCAAGTACTATCATATGCATATCTGCATCATCATCAATACCCAACTACACAAAAAACCTAACTTCACCCTGTAAAAGAAAAGCTCCATGAATCCTGATCACCACTCTTCTTTAAACCCTAGTTCTTCTTTAAAATCTAGGTTCACTAAAACCTTCCTTCGATCTTTGATTAAAATAAACAAGCAAAGACCTTTCCCATATTGTCCTAGAGAGATCTTCCAGCGATGTCATAGGGTTAAGACAGCTGCTGATAAATCCCTAGCTCGCGCTGCTGGGTCAAGAAGGGTTTGGAGTAGGGCAATGCTTTGCAAGATTCGAAACCGAT

>L0214

GGCTGCAGGAATTCGAATTCTTTTTTTTTTTTAAAGAGAATGAACTAAGTTTATAAGGAAAAGGATTGAAATTGTACAACCAAAAGTGCCAAATTCAAAACAATTCTCACAAATCAAACAAGCTCAAAATTTGAAGGAACCCCAAGAAAAGAAATCAGAGATACTACTAATCAATAGTTTTTATTTATAATATAATAATAAGTCAATCCATAAGCATCTATGCACCACCAAAATTATTACATATCTGTAAAATCTCACTCTCAGAATCCTACATAATCGTATATTTATATAAACATACCACCTCACAAAACCCTAGATCCGATCCACCAGTTCCTAACTGTAAACCTCAAAAATCAATGATACCCTGAATCGAGTCCTTAGATCTTCAACCCATAGCCAAAGAGGAGAACTGCAGCAGCTGAAACGCAAGCGAAGCCAAAAGAAGGCGAGATCAAGCCAGCTGCAGAGGTGGGAGTGGGAGCAGGAGAAGACCCAGGCGCGGCGTCTTGAGCAGAGACCGCAGAGAGTAGAAGAGCCAGCGCAGCAATGAGAAGAGTCCTTGAGAGGATCGCCATTGATTCTTTGAAGTACGGGTTAGGGGATGTTATGAACTTTGAAAGAGAAACCAAGACAAGAGAGAGATATTGGAAAACGATGGATCGGGAGAGAGAATAGTCTCGAGGGGGGGCCCGGTACC

>L0215

GGCTGCAGGATTCGAATTCCTGAAATTTTTCTCTCTTCACTTCGAGAGGAGCTACTGCTTCCTCTTCTGCTTGATTTCATTCTCCTCTCTTCCATTTTCAATTCCTACACTTGTTTGGTTCCAGTTTAGGAAGAGGGATGAATAACAATAACAACTCCTCCTCCGGCACGACATGTCCACAGCTGCTATTCCCTCAAAACTTGCCTGTTGGATGGAGATTCCACCCGTCTGATGAAGAACTGGTTGATCATTATTTGAAGCGGAAGAGGCTTGGCGAGCCTATACATGGCTTGGACATCGGTGAGGAACAAGTATGCGATTATTATCCTTGGGATCTACCAGGTCGTTCCAAGAATAAATCTCGGGATAAAGTGTGGTATTTCTTTTGCCCTCGCCAGTATCACAAGACTAGTGGCCTGCCTAAGAGGAAGGCAAACTCTGGATACTGGAAATCCACAGGTGACCTTCGCGTAGTCACGCCTGAAGACAGTGATGAGGAGATTGGAACAAAAAGAACCTTGGTTTTCCATAATCCTGACGTGACCCCATGGGTCATACATGAGTATGAGTACACTGCTGCA

>L0216

GGCTGCAGGAATTCGAATTCCTCACTGTCCTCTACTCTGTGCCTTCTCATTACTGTCCTCTGCCTTTAAATGGATCTGCGTGCCCTCTGCAAATTTTCCTTCTAATAGACTCCATTAAGTCCCGTTTCGTTTCCGATCTTATGTCATTTCCGAAACGTTTCGCTTCTTTGAGTTCCGATCGGCTCCTGCATTTCCTCTCTGACCTCAACTACGCCGAATCGGAGCTCCTGCAGCTCTCCTTGTGTGATCAGTGCTTGTTGTCTCGTGCATTTGCGTGATCGATGCTTTGTTAGTTAACGAGATGAATCCTCTCTGCTGCATTGCTCCGGTTTCCATCGATCGGGATCGGGCTAACCCAGTGGTGGAGAAATCTGATAACCAGTCTCAGTTAGGTCTTGAAATTGCTGATAAAACAGTCAACCAGTGCTCCAAACCTAGTTTCTCAGCTCAGGTATCGTCGGTCGGTACTGATTTGGATAGGTATGCCGCCGTTGGAAATCAAGACGTTGAAGAATTGGTTGTTGAAGCTAGGGATTCAAAGGCATATAGTGTGAATGGCGGCGTGAGTGGCGCTGTGGCGGGGATTCTCTACAAATGGGTGAATTACGGGAAGGGATGGAGGTCGAGATGGTTCGTG

>L0217

GGCTGCAGGAATTCGAATTCCTCTTTTCAATTTTTCTTTCCAAGAAACCAAACAAGCAATTTTCTCCGGGCGTTTACCAGAACCAAGTTACTACAATTCCAATTCCCCATCTCTCAAAATCAATGAAGAAATCAGGCCTCTTCGCTGCTTCTGTTGCTGCTGCTTCTGCTACTGCTATATCTGCTTCCTCTTCTTCTTCCACCTCAAATTTTAGCTGTAATTCTAATATGCAGCTTTCTCCTGAGGAAGCTAGCTCAAAGAAACATCAACAGAAATCACCTTCCACAGACAAATTTGCACCAAGGTTTGATGGGTTGAGGTTTATTGAAACTCTGGTCACTGCTCACAGATGAAGTTCTCCTTCTGGCTGCAGAGCTTCGCCATCACTAATTTTAACTATCCATCCTTTTCTTTTTCTCTTTTATTTAGAAGAAAAGAATGAGAAAAACAAAACAAAAAAACAAGAAAAAAAAAAAAAGAGATGATGTTGAAGAAAGTCTTGACTTTTATTTGTGTTTTTATTTATTCACCCTTCAGCAGATATGTATAGGAGGAGAAAACTGTAATATTTGAGTCAAGTTCTGTGACAACATATGTTCACACCAAATATATATATATATATATA

>L0218

GCTGCAGGAATTCGAATTCATCCTTCTTCACCTACAAGAGTTGTATGGTGAGCACAGCAGGAATGCTAGATATGAGATATCTAGGTAGCTGTTCCGCATGAGAATGTCTGAGGGACAGAATGTTGGGGATCATGTGCACAAGATGATTAAGCTAATTGAGCAGCTGGAACATCTTGACTTCAATATGGATTTCTAACTACAGACGGATTTGATCCTTCAGTCCCTTCCTGAGTCTTTTGGGAATTTTGTGACAAATTTTCATATGACTAAACAGGAATGCAGCTTAGCTGGTTTACTCAACATACTGGTTATTGCCCAAAAGAATATACCGGACAATAAAGGAAAAGAGGTAGCTTTGATTGCATCTTCTTCTACTGGAAAGTCCAACAAGAAGAAGGGCAATAAGAAAAAGAAATCTCAGATTCCTGGTCCTTCCAAGAAAATAGCTAAATAGAAAGGGAAGACTAAAGCTGATGGAGGCAAAGGAAAGTGTTTCCACTGTCAGAAGGATGGGCATTGGAAAAGGAATTGTCCAGAGTATCTTGTTTCTCTAAAGCACAAGAAGGATAAACTTTCGAAAGGAACTAGCAAATAGTAGCAGCTTGCATT

>L0219

ATTCTATTCATTCACAATTTGTTTGACATCACCATCGTTGTTGTTCTCCAGCACTGTTGCCGACGCCTTTATTTACCACTTTTGTAATTGAATTGAAGCAGGGAAAGAGCCCTAGCTAGCTGCCTACCCCTATCCCTTTTCCAAATTGCGTCCCCATTTAGTGTGGCTGAGGAGAAATGGTTTCTGGGCTTATAAATCCCAATCCAATAGTTTATGAAAGGAAAGAGTGTAGGGTTCGAAGTGTCTCAAGCAATACTGATGAGTACGCTGTTGAGCCAATTGATCAACAGCGTACTCATCAGTATTG

>L0220

GGCTGCAGGAATTCGAATTCCTGTTTTCTTTTTAGGTCTGTGTCAGATTTTGCCTTAGAAGCGTCTGAGCACCCAGCTTCATCATGTGACAATCCATTTTCTCTAAAAGATGTCCGACTCTGACCACTGGAGCCAGTGGCATGCACATCAGACGCTTCAGGCCGACAGAAGTTAGCTGCTTCTTCATTTAGTTTATCAACAGAAGGATTGAAACTTTGAAATGACATCCGTCCCATAGCTGCTCCAGGATGGGGATCCCCTTCCATTATTACCACACACTTCCTAATGATACCAGGGGAAAAAAAAG

>L0221

ATTTCGAGTTTTTTTTTTTTTTTTTTTTTTTTTTTTTTTTTTTCTTTTTTTTTTTTTTTAAAACTTAACATGATACATTTATTAAAATTACACAAACAAGCTTTCTGAATCTCAAAATTACACGTACAAGCTTTCTGATGTGTGGCTACTTCAAGGGCACTTCAAACACGCAATCGATACTTTGGCACCGCAACCAAATGGTTAGCTCTAGGCACCGAGAGGCCAAATATCTCACTCATGTCCAACTCATTAGGCAACACACCATCTGGGAGCTCCCAGTTAAAACAATGCACTAATTGAGCCAAAAAAAACC

>L0222

ATTCCTTTTTAGTTTGGTGAGGAAAATAGGGTGAAGGTACAAGTTTCTTTGGCCATGAGACTGTATTCTGTTGTTCTTAAGCATGGAAAACAAGGTGATGATGCTGTTACAATGTGAGTTGCATTTGTAGTAGAAAGTGTAAAAAGCTATAACTTCTATTATCTTAGGTAGATTGATATATATATAAGGTTTAGATCAACCATTATTTTCATCCTCAAACACACTAGTTTGTTTGAATAAATATCAAGGCATGCAAGGCTCCAGATTTCTCCCAGGGATTACATGTATATGTGAAGATCTAATCTTTGGGGAGAGTATGATTTTGCTTTCTTTGTGTAATGCAATCTCCTTGTTTTCTTTTGGTCATTGCAAATTAAGATGATTTTTTGTTATGATGTATAGCAAAAAAAAAAAAAAAAAAAAAAAAAAAAAAAAAAAAAAAAAAAAAACAAAAAAAAAAAAAAAA

>L0223

TCCATGGGATAACTGTTGAGAATGGGATACCATCCCTAGAAGATCACAAAGCAGTACCATCTTTGCTGTCAAAATCTGTAGCAATTGAAAATTCTAATGCACTTGACGCTTCCCAACCTTGTAGCCTTACCGTCAGGATTGGAAAAGAGAAAGGAAGAAGGCGAAGGAAGAGAAAGGGTGTTACTGCAGGTTTAACAGGACTTTTTGAAGTTTCAAGTAGTCAAAGTGGGAATTCCACACCTTCATCTCCTCTATCTCCTGTAATGAATGTGACTCCTAATCGTACTTGGTCACCATCTCCTGACATGGACCCTATTGAAGCTAGAAATCCATTCACTCTAGTGGCTGACCAACAGGGTGAGGTTCGAGTGGCTGAGTCTACTGCCAAGGCAACTGTATTGGAGCCCAGGGTTTATTTAGAATGCTACAGTAGTAATTGTTTCTCTGCTACTGCAAAGCAACTTTCAGTGCCAGAAGAAACTACTGGAAAGCCCGTTTTGTTGCCTTCTGCCACTATTCCTTCTGGTGGTAGGGCTGTCCCCAATCTGATGCACACTTCTCCAGCTTCAAAATCTACAATTGCTCCTCATG

>L0224

GGCTGCAGGAATTCGAATTCCTCCACAAATTCCGGTATGCTTATTGCGGCTCAAATCGAAAAGTGGGAGAGAGACAATTGAAGAGAGACAACCTGCCCAGTCGGATCACAACCTTCTTTTCACCTCTGGCCATTGCTAGTCTACGAAGAAGCTACTGATAGAATTGAAGCAATTTCTCGAAAATTTCTGGTAGAAGAAATGATAAGAACAGGTGGCGTTGCGTTGCGATCCATTATCAGTTACTTGTCTGTCCGAAGACTCACTCCCCATTTCGTTAAACCTCTCTCTACAAATGCTACTTCAGGTGATAGAATTGATGATAAAGAAAGTCCTAATTCTTTTGAGTCACGTGATGAGTTTGAGCACCGGATATTTGGTGGCATTTCTGGGAGCAGCTCAGCTACTGACTCCTTTTTCCAAAAGCTTGACAGACTTGCGAACACTCGCAACAGGACTGGTTCCATACGGAATGAAGGAGATAGTTGGGAAGGTGATGGTTTGGAGGGCATAGATGAGAGTTCTGACTCGTTATCCGATAGAATGGATGAGAAGCTGAGGAAATGGGCAGAATATTTTGGATTTGATCCTGAGGAAACAGAACAAGATGGCTATGAGTATAGACCAGAT

>L0225

GGCTGCAGGGATTTTTTTTCTTTTTTTTTGCCCAGAAACACATACAAACATTGTTCAAGTAATTTAGAATCAAACCTGTGCTCAATCGCATTTCATTCTCAAATAATAAAAATGAACACCTCTTAGCTGGAGCAAAAAACCATCTATTGAACTCACGGTGAAGTGGTTATTGTTGAGCAGACGGGGCAAAGTGCGCTGAATAACAATACTGAATGACAACTAATATGCAGATGCACCTAGATCACATATGCTAAGTATTGATATGAATATATAGATATATGTACAAGGAGGAAGGTTACCATTACCATTGTATAGTTAGCTTAGTCTTCATATTATAGTGAGATGTACAAGCCTCAAGTGCCCATAGCACTCATCACTACTGCTAAAAGATTCTGAGCAGACCCAGCAGATGTGTTTGCCACATCTACACTCAATGTGGTTGCAGCCATCCACCTTCTCAATTATATACCCGCAAACTGGACAGCTCTTAATATTCTCTTTTCCCTTGCACCAGTCCTTCAAGGACAAATCAGGATCCTCCTTGAACTCTTTGTATCTTTCACATGACACACATGGATGATACTCCAAGTGACACCTCGTACATGTCTCTGCATAGCATGCTCCACACACAAATGGACCTCCAACCATCTCAGTGCCTGATACCCGGTAAACAGAAGGACAG

>L0226

GGCTGCAGGAATTCGAACTCCCTACCATACCACCAAGATACACTAATGGCCGTCTCCGTCTCCATTTTAGCTATCGTCACTTCTCTCCACCTCATAGCCTTTGTCCTTGCCATCGGCGCTGAGCGCCGTCGGAGCCACGCTAAGGTGGTGCCTGATCAATACGATGAGAGGACTTACTGTGTATACACGACGGACGCGTCGACGGTGTATGCACAGTAAGTTCTCTCTCTCTCTCT

>L0227

GGCTGCAGGAATTCGAACTCGTTTTTTTTTTTTTTTTTTTTTTGACCAATATGGATTTGCAGACCATAAATTGTATAAAAGTGGAACCCGTAATTTCACTTACACCAATAAATCTGAGAATAGTATGACCGCAGTTCTGCTAAATTTTAAGAGATACATACCATGAGGTAAAAAAGTACAAGAGAGAGACCAGAACAAATCTCAGGCCACTCCGTTTTTCCAGATATTTTGAACAGACCAATGAAATCAGCTATCCAGTGAAACAGACTGGGGACCTCTATAGGAAGCTGATAGACATAAAGCAGGATAATGCTAAAGCTAGCATACAGCTGAAGAGGTCTCCACCACCTGAAAAGTCCTAAAAAATTGCTTGTCAGCGACCAATCCACAAGACCTGCACAGTTACCAATAAAAAAAGGTAGAGAAAGCCACGAGGGATGGCTAATCCCCACACCCAGCTGAATGGCAGGCAACAGCAAACAGGAAGCAACCCTAAAATAAGAGCCTATCTG

>L0228

GGCTGCAGGAATTCGAACCCGTTTTTTTTTTTTTTTTTTTTCCACTAATAAATTGTATGCCATTCCATTACGTTAGTTTTGGAAGCATACATTAATATATAAACCTTATGGCTTATCCAGTTGTCTATTTCTAATGGCGAAACGCCAAAAAAAAACATAAAACAAGTCCAAACTCTAGTTTTTCTCCCATGGATCTGCGACTCTTTGAAGCTCTCTTTCTTGAGCAAAGGCCCAACAAGCGAAAACCCATTTTGAACAAACTAACAAGTTCAAAAAAGAGCTCACAGCTGCCTCCAACCGAAGGCAATTGGGCTTGTCACTTGGTGCTGTGCATTTTTCCATGCTATTGAACCAAATTCATAGCTAGCAGCCTGGTTTACACCTTTCTTTGCCACGAAAGTCACTGTGTATCTAACTTTGTCTCCTACATTTCTGAACACAAGTTTGGTGGGATTCACTTTCACCACCACCGCAGGTGGCGCAGTCACAGCCACTTCATATACAGACCCTGCCTCCCCCACATTGGTCAATTCTCGGGTATACCTTACAACCCTTTTGTTCGCAAACACCACCGAGAATGATGGGTAATTGAGCTCACCTGGGTCGTTGAATTTCCTCGAACAAGTAACGTTAGGACGCTTAACAATGGCTTGAACACGATCAATGGAATAAACAA

>L0229

GGCTGCAGGAATTCGAATTCCTATCTCCCTCTCTTTCTCAATTCCTCGTTAATGTCCCTTGCCCTTCTCTCTCTCTCTCTCTCTGTTTATATATAAATATTATTATTACGTAGGGAACGTATTTGTTTTTCTTTTTTTTATCGGAAAAGAAGGAAAAGTGATGTAGGGATGAGTCGTATTTGAGATTCAAATGGTATTGGCTGGAGGACGAGCTGGGGATTCTAATGCTTGGATCTGCTGATGGCCTCCAGCTTATACAAGTTGGAACAGAAGAGGTTTCTAGTACGGTACTTAGTTGTCTAAGAATTATTTCTCTTTGAGAAGATTTTTTATGGCGTGAGGATAGGAGGCCGAAATGGTTTTGCATCCCAAATATTTAAGAACATGGAAGAAATGCAAGATGACATGGGGCCAACAGAACAAGGGCCTTCTGTGTGGTGGGGTTCTGATTTTATGGAGAAATTTGAATCTGTTTCTTTGCTTTCACAAGAAGATAGTTTGAATCATAAGGAATCGCCTAGAAATTACGAGGAAGATGGTTTATCTTCACAGACTGCATCACAAATTCTTTGGAATACTGGGATGCTTTCTGAACGAATTCCAAATGGATTCTACTCTGTCACACCGGATAAAAGGCT

>L0230

GGCTGCAGGTTTTTTTTTTTGAAATAAACACTAAAGGATTTAATTAGTTAGGCCAAAGGCATGCTCACTATCACTGATACACAGAAGCTCATACGCTAAAATACAAACAGAAAGACCCCAGATAGAAAGAATAGGAATTGCAGGAAAATTCTACATGAGTTCCCTTGCTAGCCTTACAGGTTTTTCCTTTACAAATGCAACATCACCATTGGGTATAGTCAGCAGATGATGATCTGAATCCTCTCCTTCACTCTCTGCTGACACAGATGATACAACAGATTCTAACCCATCATCATCAGAGTAAGCTGGATGATCAGGAAGATGTCTAACTGCTGTTATCTGAATGCTTTCCGGCAGTAGACAATTACAGAAAGAACCTGAAACTACTCGTGCCAGACGATTTATCCATCCAGGAATAGGCTTTCCTGTTAATTGCAAACAGACTTCATCAGTAAAGTGGTTGCAGTTCTTAGCAATCAAATGATAAGTGTCCCCATGATATTTTGCAGAAAGATGTTCCATGAATGACCGAACTTCCGAAGGGGACAAGTTGGTGCTGCCCAGTAACACTGATCGTCGGAAGATGAAGCCTGGACAACTTCTTGGTTCCACCTCAAATACCCCACTGGTAGGGTACTC

>L0231

GGCTGCAGGAATTCGAACTCGTTTTTTTTTTTTTTTTGGATCCAAAAACTACTTTATCAAAATAAAATAGTCATTAGTTGAAAGAAAGTTGAGAAAGCCAAGCCAGTCATGGTATAAATGAAGCAAACAGGGAAGAAGACAAAAAAAAAAAAATTTATATCCCAGATTTGGTCAACTTCTCCATGGCACAAATAGGGGTCCAGAACTATAGATCTTTCATGTACTGCCAGGCTTGGATCCCCGGAAAATTTATTCCAAGCACCCTCTCAGAAGTTTATTCCTTGCAAGCAGAGTGGAACTGCCAAGAATGTTGTCAGATGACTAAAACTCTCGTCCCTTATATGGCATTTCAGGTTGGTAGCTTGGCATGTACAAGACCTGCCTAGGGTGACCGAGAAGCATATTTTGTCCATACTGGGGTCCACCAGGATGAAAATATGCTTGTGGTGTTTGCAATGCCCCAACCTGTGGATTAAATATAACTGGCTGATGGCCAGTAAATGATGGTCCAATTCCAATTCCCATTGGCATGCCATGCATATGTGGTAGAGAAGGAACATTAGGTTGAAAATAGAAGGAACCATCTGGCGACGGAGGCCTAACAGGAGTTTGGGAAGGGGTGAAACTCTTGGCATTGGGATTTAGTTTGAATTCCTTAGCATGGGGAT

>L0232

GGCTGCAGGAATTCGAATTAAAAAAAAAGAAGCTATTTTGCTTACCATTAAAACGAGTAAATTAAACTACATAAGCCTTCAAAAATTGGGAAACAGACCAACATGGAGCAAGCAACAACCATTTGTAATGCCCCACCATGTACAATAACAGGAAATAGGTGCTATACCACTTCATATTGTCTCTTAAATTGCCCATGATATTGCGCCAAATGATTTCAGGAAGTTGCGGCTTGACTTGCAATTCAAGATAATTGAAAAGCATGCCCAGTATCACCAAATGAATATGGCCCGGCAGAGGGTGATTAAAAACAAAAGAAAAGAATATATCTTTTATTATCGGATCTTAATTTACTTGCGGGGATTTCATGGGGCACCTTAACTGCACACACCAGCACTTATCCGCTATATGCCCCCAAAAAAATTGGAAAAGAATTGATGGCTGTTGAACTTTAATCAAGCAGCTGATCAGGAGGGACTTGGCCAAATATATCAGGACATGACTCCCAATTTCCCTGTTGCCTGGCTTCCCAATATCCGCCAACATAACGATATGAATCACTTCCTTTATCCTTTACAAACCACCGCGGCTTCCATCCACTCTCTTGCATTTTCCGGGCCTGGCGTTGGCGCTG

>L0233

GGCTGCAGGAATTCGAATTGTTTTTTTTTTTTTTTTTAATTTTAATTTAGTGGTATTAGAATCATCTACCAAAAAAAAAAAGTTTTAGGTTCTACATGGACATGTTCATACACCCATGAACAATTTGAAATTTTCAAACTTTCAAAGTTCAAGACTATGATTTTGCACAAAATTTAGCAAATTATTTGCGCCGAGTTATGTCGTAGGTTGAGAAATTTTGGCTGCATGATCCAACACTTCAAGAACCTCTGTTCTGCTCCTTACAATTCTATGAAACACCTCTCTAATCTGCGCCTGCAAAGGCACCAAACCTTCTCCCATTTTCCGGAAAATTTCTGCAAGCTCCGCCACCTGAGCTGCCACCTCCTCCATCTTCTCTGTCTCCCCTGGAAACTGAAACCCATCAGCAAATTCGATCAAATTCAGAGCCAATTTCTCCATCCTCTGCATCTCTTCCAACAGCCCCACCGACCCTTTTTTCTCCTTCTTCTTCCACTCCTCCCCGATCCTCTCTTGCAGCCCAATCATGCTATGTGCCCACGTTAATTGCCTCGGAATCGGAAAATGCGTTGCCAATCCACTCCTCTCTTGACAAGGCAAAGCAGCCACCAGAGCCCACATCACAAACACCATAACAGAGCTC

>L0234

ACTCTTTTTTTTTTAATAATTATTATTTATTTTAATTTATTATCAATGTATCAAACACAACGACTTCATTTCATCCCAAATCCACCATAGTTACAGTAGAAACTAGAAACCCACAAGCAGACCAGACCCATTAGATTGCTTTACAATAATAGCAAGCATAAGAACACCTAACTACCCAATTTCTTGATAACAAGACTAACAATCCAAAATCAAAAGCCCTAACCACCAAACCCATAAAGAGTCCTGCCCTGCCTTTTCAGTGCATACACCACATCCATGGCTGTTACAGTCTTGCGGCGGGCGTGTTCCGTGTAGGTCACAGCGTCTCTGATCACATTCTCAAGGAAGATCTTCAGTACTCCACGGGTCTCCTCGTAGATCAGACCACTGATTCGTTTCACGCCACCTCTGCGAGCCAAACGGCGAATTGCTGGCTTTGTGATGCCTTGGATGTTATCGCGAAGGACCTTGCGGTGACGCTTGGCTCCTCCCTTTCCCAGCCCCTTGCCTCCCTTGCCACGCCCAGACATTTTTGTTCAGAAACTGATATGAGAAAATTTGAGTAGTGAATCGAATCAGAGAGAATTAAGAGATTGC

>L0235

ATTCCGCTCGCTTTCTCAGACCCGCTATTCCTTCCCTTCTCCTCTCCCCTCCAAAATCCTCCACTGACAATGGTGCTGGAATTTCAGCCTCTGTAGAAGAACTAAAAGCGGAGGAAAAGGTTGCTCAGCCTTCTGGGTCTGCACCGGGGGCCGAAGAGAGCACTTTGGGGTCCAATGGCACGGTGGCGGATGCTGAGGTGAAGCCGGAAAGTAAATTTATAGAACCTAGATGGGTTGGAGGGACTTGGGACTTGAAGCAGTTTCAGAAAGATGGGAAAACTGATTGGGATGCTGTAATTGATGCCGAGGTTAGGAGGAGAAAATGGCTTGAGGGAAATCCAGAATCATCCCGCAATGATGACCCTGTAGTTTTTGACACCTCAATCATTCCATGGTGGGCATGGATAAAAAGATTCCATCTACCTGAAGCGGAACTACTTAATGGCCGTGCTGCGATGATCGGATTCTTTATGGCTTACTTTGTGGATAGTTTGACTGGTGTTGGTCTAGTTGATCAAATGAGCAACTTCTTCTGCAAGACTTTATTGTTCGTAGCTGTGGCAGGAGTAATTCTAATCCGAAAGAATGAGGATATAGAAACTCTAAAAAAGTTGCTGGACGAGACAACCTTTTATGACAAGCAATGGCAGGCAAC

>L0236

GGCTGCAGGAATTCGAATTCTGTTTAAAGAACTGACAAGAGGCTACATAATTGAGGCACAGTGGGCAAAAAATGGGTATGTGCCAGCATTCGATGATTATGTACAGAATGGATTAATCGTAAGCACTTGTGATGTCCTTGCTTCAGCATTCCTCCTTGGAATGAAGGATGTAGGGATTAAGGAAATGGTATGGATACGAAGTAACCCAAAAATTGTTAACTCTTCCAAGTTACTTGGAACTTTGAAGAATGACATAGCAGGCCACGAGGAGGAGCAAAAGCGGGGAGACTCTCCTTCAAGTGTGGAGTGCTACATGAAGGAATTTGGGGTGTCAAAAGAGAAGGCTGTTAAAGAGATTGATAAAATATTTGTAAATGCATGGAAGGACATAAATGAGGAACTACTTCTTCAGCCAAGACGGGTTTCAAAGATTATTCTCAAGTATTTTCTCAACTTTGGACGCATGTCAGAATTTCTTTACAAGTTCCTTGATTTCTACACCAACCCATCAAGTATGAAAGATTATGTCACAAAATTATTGGTTGATCCATTTTCAATATAAGAATGGATTGAGGAACTATATTTGTTGTGTTGTGGAGAAAACATCAAAATAAACTGAGTGAGCTGTCCCTTAGTCTGGGTATTGTGGGTTAGTCTATC

>L0237

GGCTGCAGGAATTCGAATTCGAATTCTTTTTTTTTTTTCTTTAACTGAATTATTATTTGATAAAAATAATTTAAGCATGAAACTTTACACATGGTTATATATGTATAGAGTGATCATTCTTGCAACACAAAAGAACCCTCTATTCCTTTCTCCATCAATTGTGGAGGGGGGAAATTCAAAGGAGAAAACATGTTATTAATACTAGAGGCTGGTGCAGGCTTGAACTCATAAGGTCCTTCCTTCGCTCCCCACACCTGATTTCCATCATCATCGTAACCCCTATCCCAGGTATGTATTTCACTGTTCTTTAAAATTGCAAGTTCTGAAGTGCAGTAGGTTGCTCCATTCCATGAATTTGGGAATCCTCCAGGGGGTGTAGAACCTTCATACAAGCAGCGTTTTCCACGGTCACAGCGTTTAAGATGTATTGTTGTCAGATGTTCTGCTATGTCACCAATGACTTCTTCTGGCAGTGGTCGCTGGTCCTTTGGGCGATCACAGAAATTTTTGTACTCCTCTGCATCTCTGATGGCATATGAGCTCACCTCAACATCACATTTCAACTCCTTTGGACAAGGCTTCACCATATAGAACCTCTGCCGAAATGGCTTTTGAGGCGTCCTCCAGAATGCCTGTTCGAAATATAAACCTTGGAACCATCCACCATCTCCG

>L0238

GGCTGCAGGAATTCGAACTCCCATTTCTTCTCATATACAGATACAAGAATAATTTGATGACTACAACTTGGATGGATTTTGCATTATGTCAAAAAAGTCCTACACGTCTAGTCTTTTGGCTCAATAGATCACTACTCGAAGCAGCCCTGGCTGCGGTTCAGTGTAGTCAAGGCCCTCTTCCTCAAGTAGGTATTGCTGTACAGCAATTGGAAGTCTTGCTGGAGTGCGAGAACCCCTGGTGATATGACCTGTTCCAACACATATATAAACCTGCAAATGCTGTTCTGCTGCCCGGGCTGTGCTCCTCAGAACACTTACTTCATGCTTTAGCACATGAATGGCTTCACTTACATGCAGACCATGAAGGTCTATCATCCGCTCATGTCCCCTCCCCTCTGCCTGCATCTCTGAACCAACTGGGTTCCTCAGGCGATAAATAGATCCCTGTGCCTTCCCATGGGCTGCTTTCATATGCATATTGTGCAGCTGCCCTTTCACACTCAATTCCTTTGCTAGAGCCTTATTTCCAATGATGTACGCTTGCTGTGCCCGTTCAAAGTAGGCATTCCGTAATCGCGCATGATCACGAGCTTCTTCCCGCAACTCAGAATACATATNTGCCACGGCCTCTCCAGTTTCAAGCCAGACAGGAGCTGCTCGAGGGGGGGCCCGGTA

>L0239

GGCTGCAGGAATTCGAATTCATGTACTGGAACTTGATGATCATTTTGTCTTTTGAGGCTTCTGAGTTGGCCAAGTATCTTAGCAGGTGTCCTTCTGTATTGTAGTGTATGGGAAAAGCAAGGCAGCATATGACATCTCTCTGCAAAATGAAGGACTCTATATCTTGTGATTGGCGAGAGTTTATATGTTGCCTCCAGTGTTATTAAGTCTAAATCTGTGCTGGTTTGATAGACTTTGTCATGGTTAGGTTTGTACTGTGTCATCTTGTTTCATATAGCATTTCGCAGGTAGTACGTCAAAATGGCTTGTGCAATTTCTAGAAGATATATGGAAGTCATCCATGGACGCTCATGTGCGTAGCTTCGTAAATTTAAATTATGGCTTCCATGGCATTAAATTTGCTATAATTGAATCCCTTTTATGGTTTGCAATTACTACTATCTAATTTATTTCTAAAAAAAAAAAAAAAAA

>L0240

GGCTGCAGGAATTCGAATTCAACAGAACAAATGACTATGCTTTAGAAAAGCTTTCAAGCAAATAAGAACAATGCTCAAAGATAGTCAAGAAAAAACCCTAAAAGAAAGTTGGCCATCAAAACCAAAAAAGTATCCACACATGAGAGACTCAAAGTATCCATACATGAAATCATGCAAACAGAAAATTTCAACCTGTTTATGCTACTTCATAGATAAGAAATGAACAGATTTGACAAAATCATCGCTTTCCACCACCACCACCAAGCTTGGGGCCCTTTGGTGCAGCACCCTTAGGCACATTGCCCTTGGTCTGTGTTTTTTGAGTTTTGGCCATCAACTCAGCTTTCTTTGCCTTTTTTTCATCTTTTGTTTTCTTGATCCTCTCCTTGATTTCACGTAAAGCAGCTTCACGAGCAGCATCTCGGACCTCTGGTTTCTCAGTCCTCCTCTTCTGTATGACCTCCAAGGTGGCACCAACTATAGACCTTGAATATGGCTTCTTGGTGGTCCTGCGCTTCTTCTTAACAGCCTCAGCAGCAATGTCCTTCTTGTGTTGTTTCCTGTACATGGCTGTCCAGGTAAGCTTTGAAGGCTTTAGGCGGTTGTGGAAGTACCTCTTGCATTTTGAATTGGCGAAGAGGAAAACCTGAGAATCAGATCGAACAA

>L0241

GGCTGCAGGAATTCGAATTCAGCAAGTCTCTTTGGCTTTTTGGCTCGCAAGAAGCCAGAGTGATACTGGGCGTTGAAAATACAGTGACCGAGCATGGAAGCATCGAGAAAAATGGCTTGGCACTGGCATTTCTTGGGTTTCTTCTTTCTCAGTTTGCACGTATTCTCTGCAAAAGCCAAAGCTTCGCAGTGCTCAATCAAGGGGTTGCCACTTGTGAGGAATATTAGTGAGCTTCCTCAGGATGATTATGGAATTCGAGGCTTGTCCCATATTACCGTTGCTGGCTCTGTCTTGCATGGGATGAAAGAGGTGGAGGTATGGCTTCAAACGTTTTCTCCAGGGTCATGCACGCCAATCCACAGACACTCATGTGAAGAAGTTTTTATTGTCCTAAAAGGAAGTGGCACTCTCTATCTTGCCTCAAGTTCACATCAGAAGCATCCTGGAAAGCCACAAGAATACTTCATCTTTCCCAATAGTACATTTCATATCCCTGTGAACGATGCTCACCAGGTTCATATATGATGACTGGTTCATGCCCCATACTGCAGCAAAATTGAGGTTTCCCATCTTTTGGGACGAACAGTGCCTACAAGTTCAAGCACCTCCAAAAGACGAGCTTTAATAACAAGCTATATCTGTGTGTATTGCTTACACG

>L0242

GGCTGCAGGCGGAGGTTTGAGATTGTAAGCGGGAAGAGTTTTGCCGCCGGTGGTAACCAGATCTACTCGACTCGTCCTCACTCGCCCGATCGGCCACCGGTTCCGCCCCGCACGACCAGGCAGAGTCGGACCACATCAACGCCCTGGGGTTTTAGTGATCCAGAGATGAAGCGAAAGAAGAGGATTGCCAAGTACAAGGTTTACGCAGTGGAAGGTAAGTTCAAGGCTTCCTTCAAGAACGGGTTGCGTTGGATCAAGAACAAGTGCTCTGAGATCATCCATGGATACTAGGAGAATTGATTTCGGCTCTGATGATTACATGTGGGATGGGTCCCATGACAAGTGGTACCCGTTGCGTATGTGGCAGGATCTTAGTTGCGGGGTAATGGTAAGTTAAAACTAGACTCGTTTGTTGATTTGTGTGTGTTCAAATGGCGAAACACCTGCGGCTGCGAGCATTGGACATTTGGATTTTTTTTTTTTTTTTAATCCGTCAATAGAATTTTTGAGTTGGAAAAAAAAAAAAAAAAAAAAAAAAAAAAAAAAAAAAAAAAAAAAAAAAAAAAAAAAAAAAAAAAAAAAAAAAAAAAAAAAAAAA

>L0243

GGCTGCAGGAATTCGAATTCGTTTTTTTTTTTTTTTTTGCAATCACAAACAGATGAGATAAAATGATAAAAAATCAGTGCCAACATAGAAAATTTAATAAAACGTCATTGATCTTTTGATAATGATAATTGATACCTCAACTGGTTGAGTAAAAATTTAACTAGTCCTTAAAAATCAGATAGGGGTAAAATGTCCTTGACTAAAAAACTCTCATCTCCGTGCAATTCTTTGCCCTGCACCAAGTAACAAGGCCTTCCCAATTGCACCAGAATGAAGTCCACACACCAGAGCTACTGTTCCTCCTATGACTATCCATTTCCAGTCAATGCCATGATCCATGTGTCTTTTTATGTCTACCACTTGACCTTTATCCTCTTCTCGGCTAACAGGCTCTGCAGAATCTGTGCTTTCATCAAAAGAAATTTTCGTTGTGATCCTTGCCATGAGTGCGCTTCTCGACAATGAAGCCTCTGTTTTCCTAGAATTCTGATGCTCTCATGCCAGAATGCAACTTCTTGACAGTTCCCCACATACCATGACGCACTCCCAACTTAGCCACATCTTTGGGTATTCCCATGTCCTCATAATGCAAAAGGATAACTTCACATGCAGACATCTGTC

>L0244

GGCTGCAGGATTCGAATTCCAGAATCGATTCTTGGTTGCTCCGTCCTGCTCCATCCTTTCTAGCTACTGCTGCTCCTCTCTACCATTCCCTTCAACGAAAATCCAACATTGCGGACTGAAACTGTGTACCAGGTTTGACCTGTGAGGTTTTTGCAGTGCTTCAAACTCTTAAGATTGCTGATACAAGATGGTCAATGCTACTAAAGGATTGTTCATATCCTGTGACATACCCATGGCGCAATTCATTATCAATTTGAATGCTTCTTTACCTGCATCGCAGAAGTTCATTATTCATGTTATTGATAGCACTCATCTCTTTGTACAACCCCATGTTTCTGAAATGATACGAAGTGCTATTTCCGACTTCAGAGATCAGAATTCCTATGAGAAGCCGAATTGATTTAATTTTCTTTCCTTTGCTTTGTATCATGGAGGATTTATGTATATTTTTCATAAGCTCCTGTTTTAACTTGTATATTTGGCAATTTCTTTGTTTTCATATGTCAGAGTACTTTTGCTGCTGTTCTCAATGCTTTTTTAAAGTAGAGCTCTTGAAATGGCCAGTTGAACTTCCGCAAAAAAAAAAAAAAAAAAGCGCGCTCACTGGCCGTCGTT

>L0245

GGCTGCAGGAATTCGAATTCCAAGAAGCAAACAAACCAGCTGATAAGGTACTGAGACGTTTGGCACAAAATCGTGAAGCTGCTCGCAGAAGTCGTTTGCGGAAAAAGGCCTATGTTCAACAACTAGAATCTAGCCGTTTGAAGTTGGTGCAGTTGGAGCAGGAACTTGAGAGAGTTAGGCAACAGGGTGTATACATTAGCATTGCACCAGATTCTAGTCATTTAGGACTGCCTGGAACTCTAAATTCAGGAATCACTACATTTGAAATAGAATATGGCCATTGGATTGAAGAGCAACATAAACAGGTTTCTGAACTTAGGAATGCGTTGCAAGCTCGCATCACTGACATAGAGCTTCGGATACTTGTAGAAAATGGCTTGAACCACTACAACAACCTTTTCCGCATGAAAGCAGATGCTGCAAAGGCTGATGTCTTCTATTTGATTTCTGGCAAGTGGAGAACATCAGTAGAACGATTTTTTCAATGGATTGGAGGATTCCGCCCATCAGAGCTTCTCAGTGTTCTCATATCACAACTTGAGCCCTTGACTGATCAGCAACTTGTGGATGTTTGTAACCTACGACAATCTTCTCAGCAAGCTGAAGATGCTCTCTCACAAGGAATTGATAAACTCCAGCAGACCCTGGCCCAGAGTATAGCAGCAGATATAACAAGTGGTGGA

>L0246

GGCTGCAGGAATTCGAATTCCGCAGAAAAGTGCACACTGATGAAGATGGGAGCGAGACCAGATGGCACAAAACAGGCAAAACAAGGCCAGTTTTCTCCGGAGGGACGGTGAAAGGATTCAAAAAGATTTTGGTGCTTTACACAAACTATGGAAGGCAAAGAAAACCTGAAAAGACAAATTGGGTTATGCACCAATACCATCTTGGCCAAAACGAAGAAGAAAAAGAAGGAGAGCTAGTGGTTTCAAAGGTTTTCTACCAAACTCAACCTAGACAATGTGGTTCTAGCATTAAAGATTCGCTTGACCAAAACTTGAGAAATCGAATTGACCATGAAAACGCTCCTTTAGCTAAGAACCCAGCTCTTGTTGATTACTATAATAATCCACCTTTCTTCTCTTATGATCATGGGAGCCATAATAGAGAAAGCCCATCTCAGTTAATCCCAAATATGGTTATTCAAGGTGATGGATCTTCCTTTTTTCGATTAGCTGCTGATACAAGCAAGGGCAAGCTTCAGAGAAGATAGAGATATTCTGGTAGAGAGCAGCAATGACAAGGACTATTGTTTTGGTGGTTGTTGTTGTTGTGGTGTANCAGTCATACATATAATTTATTACAGTGAGATTTTAGTTTGGTG

>L0247

GGCTGCAGGAATTCGAATTCTTTTTTTTTTTTGTCATGAATACATGTATCGTTTGCTTGCCTTCAGAACACAAACAATTTGAGAGAGTATTTTGCCTTGGCAAGATAACACAAACAAGCTGCTTGCCTCCTATATAAATACTTGCTGTGATTCCCCTGGTTCAACTGAAAGGAAAAATATACACCACTTAATGTAACAATTATTCTCCTTCCCAACCGAGTTGGCGAGCCCGTTGCTCCCATAAGGAAGTAATTTTCCTCTCCCTGATCAGCAATGCCTCGGCCTGCTCCCTAGCTTCTTCACATGTTTCGGTGGCAGCATTACATTTTTCTGCTTCTCTTTGGTATTGGGAAGCCACCCGCTTTGCTTCAGCAAAAGTGACGTTCATGTGATGAATATGTTCTTCAGCAACAGCCTCTTGCAATTTCAACTCTTCCGTTAGAAGGTCGACAAACTGCTTCTCCATCTCCCTTTTGAGATCAGGGTCATCACTTCCACAATCTGTGACTGAGAGATTGGCTAACCCAGGAGCAATCTTGAGAAGGGACAAAGGAGGAGGGCAATCACAGATGCAAGGAGGACAAGAAATTTTAGAACCAGAGAGTCGAATGGTTTTCTTAAATCTCCAATAAAGAGCAGGGCCACACACAGCCAAAGCTGAAATTACTGCAAATA

>L0248

GGCTGCAGGAATTCGAATTGTTTTTTTTTTTTTTTTTTTGTTAGCAGAAGCAGCAACTGCTCTATATTCCCCTCTTGTGGCTTTGTCAATTGTATTATAACAAGTAATTACATTGACACCCATGACCTAACTATCTACCAGAATATCATAATGAATTTAGGTTCTTGCCTTCTCAGAATTCTGCTGTGACCTGGCACTCTCCCATTGCTTAACAAGTTCTGCATATCCTTCAGCAGAATTAATAGCAAATTCTGTGAGAGAACTCAATGCAACAGACATTCCAGCACAGAGTACCCTTACGGCAACTTCAGCCATTTTCTCTGCAGTTACAACCTCTTCTATTTCACCCACATGCACTAGTTCCATATTTGATCCTGGTTCACTCACCAACCCAGCACCATATCTCTTTGAGCCACTCCTGTGAGGATCATCCCTTAGCTGCTGTGCATAAAGAGACCCCATACCAGCAGCAAAAAAATCCATTCCATCAAGAACTGGCATCTCCTGAATAGCATCAAGAAACCTTAACCATTGAATACAAAGTCCAAATATTGGAAATGTCCCACTGGACCGACGAGGAGAGAACGGTAGTTTTGATGTGTCAGGATCTGATCTAACACACCTGAGGAGCCAGCCAGTTAGTGCATGCATATAGGATCGTTGAGAAGTGAT

>L0249

ATTCCTACTTCTGTGTTTGGTACCAATATGTTTCGCCGTCCTTGTTTTATCCTGTCCTCCATGCTTTCTGTCTATCTTGAAGTTGTGGCAATGATAATGAATGAGCCCATCCATCAAAATCATTCTGGTTTTGTTCTCTCAATTACCTTCACAGAAAATTAACTGGAGAGTTAGCTGGACCAAGGCAGGCTTGTATACTTTCTACTAGGGTTCATCTAGCTTAGATGTCATATAGCGTTTTTGAGACTTAATACTTTTCGTGAATAAAGGAGGAGCTTCTTTGTGTTCCACAAGGAGAATGGAGAGGTACAAGTTAATCAAGGAAGTTGGTGATGGAACATTTGGGAGCGTGTGGCGAGCGATTAATAAGCAATCTGGTGAAGTTGTTGCGATTAAAAAGATGAAGAAAAAATATTATTCATGGGAAGAGTGTGTTAATCTGAGAGAAGTTAAGTCATTGCGGAGAATGAATCATCCAAATATTGTGAAACTCAAGGAAGTCATACGGGAAAATGGCATATTGTACTTTGTTTTTGAGTACATGGAATGCAACCTTTATCAACTTATTAAGGACAGAGAAAAGCTATTATCAGAAGCTGAAGTCAGGAATTGGTGTTTCCAAG

>L0250

GGCTGCAGGAATTCGAACTCTCCTTCTTCCATTCTCAGCCAGGTGGTTATGTCCTCTTTTGCTAGCCATTTCACTGGGAGCAGAACCCATTTACAACTTCTTGCTCTGGGAGACTAATATTTCTTTACCTGTATTCTATATAGCATCCACACTAATACCCATTTCGTAAATTACCTTAAAGATTATTTCTTTCCCCTTTTCATTTCATATTTACGGATCTGCAGCTCAGCTTACTCACTGATTGGAATAAGCAATTGCTGCAGATAATTGAAAGGGGTACACCCATTGTAAGAGTCGAGTATGTTGAAGCAATCACCTAGTAGAAACCAGAGGTCTAAAGGCTTTAAAGTGAAGCATTTTCTTCAGATATGTTTGTTACTTGCCATTTGCATTTGGCTGCTTAACCAACTCAAGCACTCCTATGATAAGAAAAAGGCATATGATGATAGCACTGTAAACATACTAGAAAAGGTGCAAAGTGAGCATGAAATTATAAAACTTGGGCGGAAGGACCTCCTT

>L0251

ATTCCTCTGCAACCCTTCACGACGGCGTCATCGATGCTCCGCCAGCGCCTAGCATCCTCCCTCCGTACACGCGGAGGGGCCGCGGCAGGTCCCAGCCGGTGGACATCGCCAGGCCACGAGGAGAGGCCCAAGGGTTACCTATTCAACCGGACCCCGCCTCCACCAGGGCAGTCCCGTAAGTGGGAGGACTGGGAGCTCCCCTGCTACATCACCAGCTTCCTAACCATTGTGATTCTCGGTGTTGGGTTGAACGCCAAGCCCGATTTGTCCATCGAGACCTGGGCGCACCAGAAAGCCCTCGAACG

>L0252

GGCTGCAGGAATTCGAATTCTATTATTATTATTATTATTATTATTATTATTATTATTATTATTATTATAACGCATAGAAAACTAATTTATAAAAAAATAAATAAAGTGATCTTTTAATATTTTCAGTCCTTTCTTTCCTCCCATACTTTCATTTCTAAAATAAAAATTCAACAAATTTGAAACTTCTGCTATGATCAACAAATTTGTTCCATTTCTCTTCTTTCTTATTGACCTTGTCTTAAACTGCTCTCTCTTACAATCTCGTTTTCCTAAACGTCGCTCTCCTTTGTCCTCTATGAACACTCCTCCTCAAGCCAATACTGACCCACCTCAGTCTACCTCCTCAACAAAAACGGTGAGGGCCGTGATCAAGGGGAGGGTGCAAAGAGTGTTCTATAGGAACTGGACAGTGGAGAATGCCACCCAGTTGGGGTTGAAAGGTTGGGTTAGGAATAGGAGGGATGGTTCTGTGGAAGCACTCTTCGCTGGGGATTCTGATAAGGTGCAAGAGATGGAGAAGAGGTGCCGCCGTGGTCCACCTGCTGCTAAGGTTACTGCCTTCCAAGTGTTTCCTTGCGATGATGACCCTGGAACTGTGTTCCAGCGCAAACCAACCGTTTGATGTGTGAGGGATTGTTGAGTGTT

>L0253

GGCTGCAGGAATTCGAATTCCAAAAACCATGGCCTTCTCTGCTACATTCTTCATCTCTTCCACACCGTCTCTCACTCTTTCTTGCAGAACTTACAGATCATCTCTCTTTCCAAAGCCCATTTCGGTTAAAGCTTCTTCAACTTCTCTGGATTACTCCACAGCCTCCGTGGCCGATAAACCGACAATCCCATTAAAGTCAAATAATTGGGAATGGAAATTTAAGGACAACTCTATAAACATCTATTATGAAGAACATGCAAAGGAGAGCTCTGATCCTCCCAAAAATATCCTTATGGTACCTACCATTTCTGATGTTAGCACAGTTGAAGAATGGAGATCAGTGGCCAGAGATATTGTTCAACGAGTTGGTAAAATTAATTGGCAGGCTACAATTGTTGATTGGCCTGGTTTGGGATATTCTGACAGGCCAAAAATAGATTACAATGCCGATATCATGGAGAAATTTGTAGTTGACTTCATAAATGCACCAGATAGTCCATTGCGGTACTCAGATGAAAAGGATTTGGTGGTCTTTTGTGGAGGGCATGCTGCCACAATACTACTTCGTGCTGCAAAGAAGGGTTTGGTTAATCCAACTGCCATAGCAGCCGTTGCACCAACCTGGGCTGGTCCTCTTCCTA

>L0254

ATTCCTTCAACGATACCTCTCATTTCTCCAACGCCTCCCATGCAATCCTCCAAACCCTAGCAATTTTCTTTTCCCCGAATTCATTTTCCTACTTTTTCTCCCACTTTTCCCTCTCTGTTCCTATCCCCTTCTCTTTTCTCTTTTCATCTCTTCCGGTGCGATTGGATCTGAGGGACTTCTTTTACAATTCCCATTCTGTTCATTCAATCCGGAAGAGAAATCTCCAATTTCTACTCTTTACGCTTTTTGGATCTATACAAGATTACCCAATTTACCGCTTTCTTGTTTTTTCCTTTCTCGGTGATTGTAGGAGTAGTTAGGGTTTGTAACCTGAATTTTTGGAGGTTTTCTTTAGCAATTTGGATTTCTTTGGGATTGGAGCAGTTAGGTTTTGTGGTTTGACTTTTGGTTTGATCGAGAGATTAGGGTTTTTCTATCTTTTGGGTTGATTGTTATTCTTTGTTTGGAGGTTTTAGATTGAAGTGGTAGCGTGGTGAGGTGGCTAAAGGAGGAATTGTGAGTAGCCAATGCCATTTTTTTGGGTTGGTGTTCGGTGATTTTTGCAGTACCGAATAAGATAGAGCTTGAGCTTATCGATGTCTTGGGCAGGACCGGAAGATATCTACCTTTCTACTTCTCTTGCT

>L0255

GGCTGCAGGAATTCGAATTCCTCTTCTGCTGCATCTCCAACAACCCCTTTAATGTCCATGAAAAGCTTCTCTTGAATATGTGGGTACTTGACCAAATTCGCCATGATCCATTGCAAGGCCGTGGCGGTAGTGTCTGTGCCTGCGTTAAGAAATTCATTGCACAAAGTAACAATTTCATCTTCGCTGAGCTTCCTTTTCTCATCAGGAAGTTCAAGATCAAAAAGCGTATCAACATATGATAAGATATAGTCATCATTTTCGTTGTTGGATTTGCTCAATTTCTCTTCTTTTACCTTCTTTCTTGCTCTTATTAAAGGAATTAATACGTCTTCTTGGTTCTTTCGAAGCTGCAAGAAATTTGACCAGCTTCTGTGAAGGACCATTTTGGAAAATCTTGGCCAGAAATTGAGTATACTGAATCTGGCATTAGTCAAAAGCATAGCTCGCTCTACTCTCTCAATTTCTTCAATTTGTTTCTGATCCAGCTTGTCACCAAAACACATGAGGACCAGCAGGCAAAAAATTGCGTATTGAAGACCATCCACAACACGAACAGGATGTCCAGATTTCGACTCTGATTCAAAGCGATTGAGGAGGATTTGCAGCATCCACTTTCGAGCGTGAGAATATGACTTCACCCTTGAAGGATGGAG

>L0256

GGCTGCAGGAATTCGAATTCAGAGAGAGAGAGAGGAGAACTAGTGGTGGAGGCGGTTGGCTCTTATCAGCACCTCCTTGGTTCCAACTGCCTCCACCACTAGTTTTCCATCCTTCGCCAACATTGTTTGTCCCATTCTTCCAGTCGGCCTTGTCATCACCTCTTAGCTTCCAGCTGCCTCCACTAGTATTCCATCCTCCTCCATCATTACCATCTCCCTTGTTTCCCCAAGGAGAGAGAACTAGT

>L0257

GGCTGCAGGAATTCGAATTCCTCTTTTATATTCTTCTGCTTTTCGGGTCATTCCATTTCTATCTTCATTTATCCCTTTGCCCCCAACTTATATATTATTGTCAATTTTGTGAGTGCGATATAGTGCTTCTGATTTTGTTTCTGTTAGTTTTGATGGGCTGTTGATGTCATTGTTGGTGTGGCGTCAAGTACCAAAATCATGGGTTTTGATGAGCATGGCAATGAGGAAGTTTCAGAGACGAAAACAACACCAAAAACTTCTCCTGATGGAGAAGAATATCAACTTGATGCACATAAAAGTGGTGGGATGAGCAGAGAAATGAGTGAGAGCTCTATTCATGCAACTGAGGAAGAGGAAGAGGATGAAGAAAGGAAGATTGAGTTGGGTCCTCAGTACACTCTCAAAGAACAACTTGAAAAGGATAAGGATGATGAGAGCTTGAGAAGATGGAAGGAACAGCTTCTTGGAAGTGTGGATTTTAATTCTGTTGGAGAAACTCTAGACCCAGAAGTGAAGATCCTGAGCCTTGCAATCAAATCTCCTGGTAGACCTGAGATTGTTCTTTCTATTCCTGAGGATGGAAATCTTACTGGCATATGGTTCACCTTAAAAGAAGGCAGCCGCTATAGCCTGCAGTTCACTTTTCAAG

>L0258

ATTCCTTCCCCATTTCTAGTTTCCTTTGATTTACAGCCTCTTCAATTTTTTCTGTTTATTAATGTAGCTTTCAATATCCTTTCCATCGACCATCACTCTCCTCTCGTCTGCTTGTCCTTTCTCTCCTAATTGGCCAGCGCTCTCCTCTGTGATTTTGGGAGTTCTTATGGTCTCAGTATCAGCAACCATTCTCCTTTCGCCTAAGCACGTCACAGGTAGCAGAGAGTATCGAGTTGTAAGAAATAATTCCGATGCTCCATCTCCTTCAGCACAACAAAATACCCATCTCATTCCCAGTATATGTTTGAGAATAGCCTTTGCTATTGAATCCATAATCAAAGCTTGTAAATCAAATTGTGTTGATCTTA

>L0259

GGATCCCCGGGCTGCAGGAATTCAATTGAGATGTTCTTCCCGGTCAATGGATTATTTTAACATGGAGGGTGGGTCTCTCATGATATTCCAACCACATTGCACCGGAGTAAAGCTGATTGTCCAGTGCCAGAGGAAATGGTTACTGTTAGTGTTGATGGATCTGTGCTGGATCGAATTGCTAAAATTATGTCATATCTTCGCCTTGGATCTTCTGGGAAGGTTCTCAAGAAGAAAAAGAAGGAAAAAGATGCAAAAGGAAAAGTTCAGCTGCTAGCAATGAATATGATGAAGAGGGTAAGCATGCAAAACCAAATGGGGGGATTTTGAACAATAAAACTGAAAGAGAGATTTTGCCTCTGCCACCACCACCACCACCACCTCCCAAGAGAGTTCAGTTTGATTCTCAAGAGAAGCTGGGTCCTGCAGTTGCTAGGGAAGAAGAGAATGACATATTTGTGGGAGATGGTGTTGACTATCATGTTCCTGGAAAAGACATGTAT

>L0260

GAATTCGAATTCCTTCAAGCGCCCATGCTCATAAAGGAATACATCGATGAGGTCTCAACTCAGCTGAGAATGGTTTGTGATTCAGATTCGGAGGAGCTTAAACTTGGAAGAGAAGCTTT

>L0261

ATTTCGTTTTTTTTTTTTTTTTTGGACACAATGTCTATCAATATTATATATACACAAATTAGATTTCAATTATTGCTTCTTAAATGGCAAAATAAACTGTGTACTAAATCATATGGACAAATGAGAAATGGATACAACAATGTCAACATATTTCACATTGTCACAAACATTAAACTATAACCANGGATGTCAAATTTACCCTTCGCACAATCAGAGAAAAACTCAGCGGCATTCACTAATTGGTGGACCTCTGCTGGTGCTTGCATCCCCTATTGACACCGTAACAAGGGACTTGGACGCCCATGCCCTGGCATTCATCGCTCCTTCTTGTAAATTAGTTTTCCTGCAAGGCTCCTTCAGAGAATTGAATACTTGTATTATTCCATTCAGATAGGCCCGAGGATGCTCCAAGAAGCTCCCCAATCTCAAGTTCCGAATATGCCCTCTCCAAGCAAATTGAACAAATACAAGCAGAGTAGGGAAAAATATATATATCAATCTCAAAAACACAAACGCAG

>L0262

CACAGGAAATAATTATGCCAGTTAAACATATACCTATTCACAATCAATCCAATCCTTCTCAGATTAAACAAAAAAAGAATTGAATAAACAAGTTCATTATTCCCTTAAGCCAAACAGAAATGACACGAATTTATTCATTAGTAGTCGACTCACCTTGATTCATCGATTTAGCGCCGGAGAACTTCAAAAAGGCCACAGCAGCTCCTCCAAGATGTACGTTGCAGGGTGCGCTGACTAAGAGATGTAACATGTGGATTTCCTTTGCTCTCAGGGAAACCATCACTACTGCCAACAATAGTAGCATTGTTTGATGGCTTCAGTGAATCATCAGTATTTTCCTCGGATTCTTTATCAACAAAGGCAGAGGGCTCCCCAGTGTTCTCCTCCAAAGCTATCACTATTGCTTCCTCAACTCCATCTGACACCGTATCTGTTGCAATTGCAGATAAATCACGTTTATCATCCGAAGATGGCAGCCTTGTTTCCTTAGATCCATCTGGCGCCACTTCAGATACACCTCCATCAGTCTCATCCAGAGTTGTCTTCTCATTGGTCTCATTCGAAGACGGGAAGATTTTCACTTCAATTTCTTTTTGTTCCACTTCAGTCACATCAGGCTCCTGTGATTTCTCAATTGATGTTTCTGTAGTCGAAGAGAGTACTTGTGG

>L0263

ACGAATCCAACAGTCGAAAAAATCTCAAGAGTTTTTGTTAACCACAAATGCTAATTGTGCTTATCATGAAGATGCTACTTCTAGATATTCAACACAATCTGTTGATATCAATGATGAAAATAAGCAGTATGACAATGTGAAATTGGATTTGGTTGCATCACCAGAGGGCAAAGATGCAATTTTATCATCCTCTTACGATGGAAGCAAGAATTTGCAGAAAAGAGATGTTAAGGACAATGCCACATACAATTTCTACCAGGCTGTTGATGGTCAGCACCTCATTCTTCACCCTCTAAACATGAAGTGTCTTCTAAACCATTATGGGAGCTATGATATGCTTCCGCACAGAGTACGTGGAAAAATCTTGCAGTTGGAGACATTGA

>L0264

ACTACGAATTCCTCAAGAGGAGTCATTTTTATTATTGCTATGAGCCTGGTCATACACAATATAATTGTCTGCAACTTCAGAGGAAAAATCAGCGATCACAGATGGCAAATATGGCAGCAGAGGATTCTACAGTATCTTCCTCTGAGAAAACTATTTTGGTATCTGCAGATGATTTTGCACCAGTTTTCCCAGTATTAGGCACTCTCTAAAGCCTACCAGTTCCCCTGTCATTGCGATCGCTGAGTCAGGTTTAAAAGATTGACAGGTCGGAATGAGGTTGTTGTCTCTGGCGCGC

>L0265

CTCCGTTGCCTTTATAATTTTCAAATATTTCTTTTTCTATCAACAAACAAAGTCTTGTCACCCACCCAAGTAAGTAACGCAGCCCTCTGCCTGTTCCGGCTAAGCTACACTTCTTGACTTCGCCTTTCTCTTCACCATCATGTAAGATTGTTCCTTTCTGTTATTTTTTCCCCTTTCTGATTTGGTATTNTTCTTGCCCTCTTGTCTCAACATGTCTAAGTCTTGGTGCAACTTCACTTCAAGACCTCCCTAGCACTCCAAAGTGAATTTTCATTCCCAATTCCCATCGGTCATGACTCTTTATCTCATATAATCAGATACCAAAGATGGAGGACTATAATCAAATGAATGAG

>L0266

AATTTGTGTAAATAAATACGCATATCTGTATATATTTGATACTTTCGTTAGGCTTTTTGGTTGTGCAATTTGGGTGGCGTTGTGATTTGGTTTTGTTGTTTGAATATTGAGGATATT

>L0267

GGTTTTTTTTTTTTTTTTTTTGTCACAAATTTGTGTCTTTTACACTCTCTCAACTTAACTCGCATAAGGAATGCAAACATTATTGCCTTCTATGAAGATTTGCATACATCAAAGAAGCAAACCACTTCATACCATTGGCCAGGCTTTGAAATGTAAAGTTATTTGCACAAGTTGTTTATCTAGCAGCCAACTATGCATTCAAAAAATATGCCTTAAGAACATGCAAAAACCTTCTTGATTCATTGACACATATATGTTATTAATTTTCACTATAGAGACCCATTAATATAAATATTTTATTACATCACATCTCCAACCAAAAGCATTAGTGAAAAGCACCAGAGATGCCGCTGCAATTAACCGCAGCAGGAACCACAGGCAGGCAACGAACTATGGTCAATTCCTCGAACATTATCTAAGAACGCGCGCTCACTGGCCGTCGTTT

>L0268

GCAAAGTTGATTCCAATTTACAATGGTTTAAAATGCTTCGCAGAACACATTTCATTACTGCCATCTCACAAATCAAGGTCTCTATAGGGCTTTGTCATCTTTCATTCTAGTATTTTACTACTGACACAAAATTGCCTCAAATGTCTTACACCATAAGCTAGTCCACTCGCTCTGGAGGAGCTGTCATGACTGGAGTCCCAAACCCTGAAGAATAGATTTTCAGCTTCCATTGGCAGCTGGGATTGCTGCTGGTTTTGCATCCTCCGCTTT

>L0269

TCGGAAATAAATCATCATCAGAATTTACATTAACAGTGTTCCCCCATCCCCATGTACATATTAAACGATCTCAAACCAACACCTCAAAGTTAGTTTTCCCATAGCATAGCAATATCCACCACACGAAAGCTTTGCAGAATCATTTCCTTATTCTTGTAATAAACTCTTCGGTCTAATCAATAATTTATAAACCAAAAATCACATAGATGAAACTTGCGGACCCTTTGCATTGGCACCAACAACCTGAACCTTACCATCTTGATTCAATGCGCTATTATCTGCTGCAACTGCCTGATATGGGGTAGTAGTATAATAAACCTGCCTCCCTGCAGTGTCGTAACCAACCTGCACATATCCCTGCTCGGCTACTCCAACTTTAGCC

>L0270

TCCGAGCGCATGATTCGAGCTGAGAACCCGATTTAATCGTTTTCACTGGGGATAACATCGTTGGATTTGATGCCACGGACGCGGCCAAATCTCTGAGTGCTGCATTTGCACCTGCAATTTCGTCGAACATCCCATGGGTGGCTGTTTTGGGGAACCATGATCAAGAATCGACGTTGTCAAGGGAAGGGGTCATGAAGCATATTGTTGGCCTTCAGAACACTCTTTCTCAACTCAATCCTGCTGAGGCACACGTTATTGATGGTTTTGGAAACTATAACCTTGAGATAGGTGGAGTTGAGAATTCAAGATTTCAAAACAAATCAGTTCTCAATCTCTACTTCCTTGATAGCGGAGACTACTCCACGGTTCCATCAATTACTGGCTATGGTTGGATCAAACCTCTCAGCAGTTTTGGTTTCAACGCACTTCTGCAAACCTCCGG

>L0271

AATTCGAATTCCAATTCAAACAAAGTATTAAATATAACTTCCATAAGGTTTATGACCGCAAACTGACACTAGATAAAATTTCTCATACAATAACTATTAACATTAAAGTTCCCAGGGGAAAAAAAACCTTCAGATATTGGAAAAAGAAAAATGATGGAAAAAATAAAAANAAAGAAGCATCCCAATCACTCTCCANGCTACAAGCATCCTACTAAAAAAGACATTGGTTGAACCACAAAAGATTTGAGGAAAATAATGACTTGAGTACCAAAGTGCCTGCCCACCAATTCCAGAGGGACCCATTCAGATAAAGCCTCTCACTAGCAGCCATAACATGAAACGCAATAGATGGAACGCTATTTGATACATCAAGTTCAGAATATATAATCATCTTTCAATGACCCCATCTCAACATTTCTCCATATCAACATATTCAAGTTCAC

>L0272

GGAAAACAACCCAGAATCATGGACCTATCTCCAAGGATTCAAGTGGTGATAATGTTCGAACTAAGGAAAGTACAGGCAAAATAAATGATGTCAATGGGAGTGCAGGCTCTGGTGTGGTCACTGTTCCATCTGAAGAAAGAGGAACTGAAGGTCAAAGTTATACTGGAGCTTATGATTCTGTGGCAAATTTACCAATGGAATTCTACCACTATTACTACGGTAGCAATAATGATATGGGCACACTTATCGAGGTTAGAAGAACATGGGATGCATATATAAGACCAGGAGGGAGCCGCATTCCAGGGCATTGGGTTCAGCCACCTCCTCCAGCTGATGACATATGGGCGTCTTACCTTGTTAGGTCTAAATGATTCCTAACTATTAAAAAGACGGAGCTTTGGCTCCCACAACTGGCATATTGCAGAGTTTTGAAAGCATTTATGAGCTGAATNGCTCCGTAGTTGCATGGAAATTACACTGTGTTCTTGCATCTGCACCCTTGATTTGGAATGACTGGTGACTTGTAACTTGGCGGCA

>L0273

ATTCCCTGAGCAAAACCCCATGACTCCAGCTCACTGTTCTTAAGCCACAGCCTGCTTCACACCGTCACTGTCACCACCGCAACGCACGACATTTAACTGTTGGCCTGGTCAATCTCATCATCAATTTCTGTGATCGCACATGGGCGAGCAAACCCAACACCATCCACAACTCCAACCCCAACCCCAACCCCAACCC

>L0274

ATTCCGATGAGTGATACGAACAAAGGGGTTCCATAAGTAATTGGAGGGACCATCGATTCTTCGGACGTCAGCTGAGAGGCCTAGGAGATTAGACAGTAGGGAACCGAAGCCAAGAGCTAAACATATGCATGGGTGTACCCTCCAACTCCTGCGTTGGCGTGAGGGGATTTGGGAATCATAGAGGGCGTATTACCAACGGAACCCCAAACGTGAGGTGGTCAGTCCTACTCT

>L0275

AATTCGTTTTTTTTTTTTTTTTTGAAACATAACCATGCTTTTCTAATAATTATGAGCAAAGGAGAGAGCACAAGCATGCTCTTCTGACATACATGAGCTAAGGAAGACACCGTTACATGAGATGTGGCTTGCGGCTTCGCTGCCAGAGTAAAATACCTGCCACAGTGCCTAGCATAGCATTTGCAATCCCTAAAAAATTCCTTGATTTATATNTGAATAATTACAATAAATATCATCTGAGCCTATTTCCAGATTATCATCAGCCACAGCAACAAGACAATGCAAGTAACAATATCAATTATATGCTGAAGGGCGAGGTCTTGGCGGGAAGTAAAAAGAATTGCCAAGTTCTATTGAAG

>L0276

GCAGGAATTCGAATTCGTTTTTTTTTTTTTTTTTTTTTTTTTTTTTTTTTACTGGCACGCAAGAAAACTAATCATTAATCGACATAATTCGCAAAGGAGACTACCCAGACCCCAAAACAAGAAGAAAGAAACAATACATTCCCATGGATCATTTTCAAATCCCCAGGAAACAAACAACGAAAAGTGAATCAACTTTCTCAATAATCAACACTCGGCTGAGTTTGGCACGCAAGCGCCTCCGTGTAAGATAAGATTAAACATGGGCGCGTGGACTCGACGCCCATAAAAACGACGCTACCCATTTCAAAAAAGGGGTACGTGGTTCAGAAATGAAAGGCCCATAAAAAATAAAATAAAATACGCACGTGCAAGAGTCGCCAACGTTTCCTCCACACTGATGAAACGACATCACTGCCTGAACCTT

>L0277

TTTTTTTTTTTTTTTTTTGGGAAAACAAATAAATGCTTGTATTGAGATAAAACTGTCTTTATTCTAAGAGTCGTTCCAATCTATGCATCAAGCCATATCTACAGGTAGAACAAACTATCCACCGCTAAATAAATTAGTAAATCATTATTCAACAACTGTTATATAATAAACAAAAAGACAAAGATAACACCCAAGTTCCCCCTC

>L0278

ATTCTGATCTTTAATTATTAATTGGTAATATTACAACCAGGATACAGAAATACTACTAGCACTTTTACAACAACCCATTTATCGCATTTGCTATAAGAATTATAATCTTTAAAATACAATATTTTTTTTTTTAAAATTTTGGCTTGGTAAGAAAAAAATATTAACAGAAAATCTGACACACTATGAGCTTGGTTTTCCTCTTCCACCGTAAGCTTGCTTGCACATATAACTAGGCTTTTGTGAAGGCGTGCACAGCTGAGACGAACCTGGACATCCTTGCAAACAATTATCCCCCAAGCTACCGGTGACAGGCGCCTCTCCGGAGAAAAACCCAGACGGCGGCTTCCCAATCAAGAAATTACCGTCCAAGAACGCCTCTCAACTCTCTCTGACGTCT

>L0279

AGGAATTCGAATTCCTGAAACATTGGCTCTTTCCCTAGCCTGCTCGTTGTTCTCCTCCAGCCTTCCTCTCAAAGGGAACTACCCCGGGGCTAGAAGTTGGAACTCCTGAGCTTGCAGACAGATAGAAATGGTTATTCCACCACCAGTTAAGTGGCCACCTAGAGTCACAGAGTTTCTGAAACCTTATATCCTGAAGATGCACTTCACAAACAAATATGTGAGTGCCCAAGTTATCCACTCACCAACTGCCACAGTAGCATGTTCTGCAAGCTCACAAGAGAAGGCCTTGAGGTCGAGCATGGAAAATA

>L0280

ATTCCTCAAAAGCCGAAAAAATAATTTCCCTCGTCTCTCTGATCATGTCTGGCTATGCTCATCCTCCAGCCAGCTTCCGCTGCCGTGCTCCACCGCCACACCAACCACAAACTTGCGGAGCCGATCCTCCAAACGGTAAGCCGGAACCTTCTCAGCCCTGCGATGCACCCTGCAGCGCACCACCTCCGAGCGACAAGCCCCCAAAGGACAAACCCAACTCCTATGCATCTCCACCCGCACCTGGCTATTACCCACGGTCAGCTCCATATGGAAGCCCGTTCGCCTCACTAGTACCCTCCGCTTTTCCTCCAGGGACGGATCCCA

>L0281

GGAAAGTGGAGGAAATGGCAGGAGGAGATGGCCAGAACATCACCCGAGTTCTCTTTTGTGGGCCACATTTTCCTGCTTCGCATATTTACACTAAGGAATACTTGCAAAACTACCCTTTTATCCAGGTTGATGATGTACCTCTCAGTGATGTGCCTGATGTTATAGAGAAGTATGACATCTGTGTTCCCAAAACTATGCGGGTTGAT

>L0282

GGCTGCAGGAATTCGAATTCGATAACTGAGGGAAACGAAACCAAACATGCCCTTCTCCTCTTACATTGGCGGCAACACTCGCCGTGGAGGTGGAGGAGCCGGCACCACCTGGGCTCAATCACTCCTCCCCTCCTCCACCGCTAAATCAAAGATTCCTCCAAGAAGGTCTCGCAGGCGGACGGCTCTGATCAACTTCTTATTCACTAACTTCTTCACTATAGCTCTCTCGATCTCTCTCATCTTCCTTCTCATCACCATCCTCCACTTTGGCGTCCCTAAGCCTCTTTCCTCTCCCTTTAAATCTAGACCCAAGCCCACTTTTCGCCTTCCGAAGCCTCGCAAGACGATTCCTAGGAAGCCCCAAAACGATAACGATAACAAAGATGCCGTTTTTGGCGCTGTAGTGGATATAACCACAACGGGTTTGTATGATAAGATCGAATTCTTGGATGTGGATGGCGGGCCTTGGAAGCAAGGGTGGAGAGTGAGCTATAGAGGAAATGAGTGGGATACTGAGAAGTTGAAGGTA

>L0283

ATTCGGTTTAGATAGAGCTTACGGTGAAGCAACTGCTTGTTCATCTACACCTTCTGCTTCTATATCTGAAAATCATGGTTCAGCTGTTAATTCCTTGAAAAATTCATTGATGGTGGATTCATTTTTTAAGTTGAGATGTGAGGTGTTGGGTGCAAATATTGTGAAGAGTGCCTCTAGAACATTTGCTGTGTATTCCATTTCTGTTACAGATGTAAATAATAATAGTTGGTCAATCAAAAGAAGGTTTCGACATTTTGAGGAGTTGCACCGGCGTCTAAACGAGTATCCAGAGTATAATCTTCATTTGCCACCAACGCATTTTTTGTCAACAGGTTTAGATATGCCTGTCATTCAAGAACGGTGTAAATTGCTCGATATATATCTCAAGAAGCTCTTGCAACTTCCTACACATTCAGGATCTATTGAAGTTTGGGACTTTCTTATTGTTGATTCTCAGACATATAATTTCTCAAATTCCTTTTCCATCGTTGAGACATTGTCCGTTGACCTTGATGGAAGCCATCTGAACAG

>L0285

ATTCCTGTCTTTTCTGCGGTGCGTGATAAGATCATTAACAATCTTAATAGCCATGCTGCTCCTGAAACACGAGCTATAGCTGGACAGCCATGGGTAAGAGGGTGGGATGATATCAAGAAAAGTCCCGGCAGCCCTGTGGCTGGGAATCAGTAGCTGTTCTATTGTTAAGCTGGAAGATTTATTGGCTTGCAACATATGAAAAAGAATAATATATTTTGTCATATAATCTACATAGCGTGATTTGAACAAGAGCTACTTACTGTAACGAAGTCAAGAAATTTAGTGCCCTCTCCTTATAGGTTCATGACCCATTTGCAAGTTGTATCTTAGAGTGAGAAATTGTTCTGTGAAATGTGTTTTATTTTTGAAAAAA

>L0286

TGCAGGAATTCGAATTCATTTTTATTTATTTACTGCAACAACTTACAAGCTTACAAAAACAACAAAAAGAATTGTAAGAGTTTGGGGCAAAAACAACAGACAATTATTCCTATAACGTGCTACCTAAAATCAAAATTCAAATTAAAAAAGTGATTTTAATGAAAAAACAAACAATCTAAAGCTCATGAAGCTGTTCTCTCCACTGCATCTCAATTTGAATCTGACCATTCTTGGAGCCTATAAGATGGTACTTCTCATTGATCCTTCTGTTGCTAACCACATCCGCAAGACTGATGTCCACATAACCCAGAGATTCCTTAGGATGCAACAAGCCCATCCTCGATGTAGTGCTAACAACTTCTACACGTAGCTTATCGTTAGTGGGAGGCTCATCCAACATGAACTGGAACTCCTCCTCCCATCTAGGATCTCGGTTCTTCTTTAAATGCTTAGTTTTCCTCTCCTCCCCTCTAAAAATAAGCCGTACATGTGGATTAGTATGATACTTTCCTTCAACATCCTGAGCCTCATGAACAATCACTACTAGCAGACCTCCACCAGCAGGGATTGCATCTGGAGCCTTCTGCACTGTACTCGACTCTTCAAAACCACTGGGCAAATCCTCCTCTTTGAATGGTATATATGTCAATTCCACCTC

>L0288

AATTCCAGTTTTTTTTTCTTTTTTTATTTGATTCTCTTTGATTTTTCATATAGTCACATTTGATTTTTCACATCGCATAACACACTTGTTACACTCTTTCATTTCATCCATCCATCCGAATTTTGAATAGATTCAAACAGAAAACACAACAAAAGAAAAAAGAAACACAAGTATTTTTTACATAATTTTAATCCATTGTTCTTAATAAAACCTCCTCCAACTCAATCACGAAATTTATGTATCATTACATATTGGCCAGGATCGTTGCCCTCAATGTAAAGGTGAAAAGGTTGTTCAGGAGAAGAAAGTGCTGGAAGTTATTGTTGAGAAGGGTCTGCCAAATGGCACAGAGGATTACTTTCCCTGGAGAAGCTGATGAAGCTCCTGATACTATTACAGGGGACCATGTTTTTGTCCTTC

>L0289

TCCCCTCGTCTTCTGCCAACCAAAGAGTACTCCAAAGAGCTTCAAAACCCAACCCTAAACCCCAAAAAAACTAGAGACAACCAGCAAATCCAACCATGGTGTGCATAAGGAAGGCGACAATAGATGACCTACTAGCGATGCAGGCCTGTAACCTGCTATGCCTGCCGGAAAACTATCAGATGAAGTATTATTTCTACCATATTCTCTCCTGGCC

>L0290

ATTCGTTTTTTTTTTTTTTTTTGGCATAAAATTTTAATTATTTGTAAAGCAGCAATAAAACCTGAAGCTAAAGTTCAAGTGTAGACATCTATTCTAGGCAAAACATCAAAGTCTACATCAATATTGTTTCAGTTCTGAAAATTGAAAACCTTTCACTTCTCGAAATATGAATTGCATCCTCACTTCCAGCTCGACGGCCAGAAAGTATGAACCCCATAACCAGTCTTCCAATTGTCCAGGTAGATAACCTTATTCACAGCCCAAAATGAAACCACTAGTGAAACGACCATAATCGATCTCTGGACTGTCACATTCTTTACCATTACAAGCACATGAGTCACAAACGCTAACAATAGCCCCACAATGGTGTACAAGAACCCAATGGCGAAACCCTCTGCTCCCAGTTGCATCCCAGACCCCTGGTAAAAGAAAATCAGGTTGGTGGGGTCGTTTCGATCGGCCAAAAACATGGGCATCTTACGAATTATATTGTGCATTGCACCGGAA

>L0291

GGCTGCAGGAATTCGAATTCCTATCTATGCCTTTGCATCGATAGTCGCATATTTCACTCCTAACTCATAGCAATAAGTTAGTAGTCGAAATATGCGACTATCTATGCCTTTAGCATCGATAATTTTCGAAGGAAACCTCATGAGGTTCAGTACGTAATGGATCTAATGCTGGAGAAGATTGATGGGATGATCAAGGAAGAACGTATCATCAATGCTTATGATATTTGCGTACGTTTTGTGGGTAACCTGAAGCTTTTAAGTGAGCCAGTCACGACCCGCAGCAGATAAGATTATGAGGGCTACTGCCAACAATTCCAAATGTGTGCTTCTCCTTGCTGTGTGCTATACTTCAACTGATGAGATCGTGCATGCTGTTGAAGAATCCTCTGAATTGAAATCCAATGAAGTTTGTA

>L0292

GCAGGAATTCGAATTCTAAAGACGTAAAAGGATCTCTGTTTGGCTTCAATTGAATAGTTATATAACAGTGGCTAACCCCAACCGTATACCGGCACTTTGCTATTCTAGCCGCCACATCATGCTTACTGAATAATCACAAACCACCGCCACTGCCGATTAATAAGCTGGTGTCTGCAAGTGGGTTTCCTTTGAAATCTTCAAATTCACTTGTTCTCCTTGGTGGGTAATCTTTGGTTCATCCATTCATGGCTTCCTCAATGCTTAGTGGAGCTGAGAACCTCACTCTCATGAGAGGCATAAGCCCAAAAGTGAAAGGGTTAGGTTTTTCGCGGTCAGATTTTCACGGGAACCACTTTCCCGGAGTGACAATCACTTGCTCTAGTATCTCCAGGACAAGAACATTGATAGCCAAGTGCAGTTTATCAGCCTCTAGGCCAGCTTCTCAGCCCAGGTTCATCCAACACAAGAAAGAGGCTTTTTGGTTCTACAGATTCCTCTCAGTTGTATATGACCATATTATAAATCCTGGTCACTGGAATGAGGACATGAGAGATGA

>L0293

CCCATTCTCGTCCCAAGTCGCACCAAAGCAAGCTCTGTACATTACATCTCAATGTCTTCTAGCATCTGGTAGCGGAAATCGAAGGATGGCGGCAATGCCAGTCAATTGTGCCAATTGCTCTCCTGCACACATGCATTGATGAAAAATATATGAACAGTGCCCCCTGAATCCTTCA

>L0294

CTAATAAAACTCTTTCTCCTGTTTTTGATTAATCAGTTACTTTGTCTCCCTACACCTTCTGCTGTACAACAATGGCGAATCCTTCTTTGGACGAAGGGACAGCGAAGGAAGTCCTTAGACAGGTGGAGTTTTACTTCAGTGATA

>L0295

TGCAGGAATTCGAATTCGCCATAGCTTCTGCAATTACAGAAAAATATTAGAAGAAAGGAAATGGCAGCAGATGTGAGTTCCATGGTGAAGGGTCGGAATTCAGAGATTCTGATCACAAGGGACTTGCTTGGAGGCTTATCTAAAGTTAGAAACACTTCCCCTTTGGATCTCGATTTACAAATCCCTAAATCCTGCTTGGAAATGCCTCTTCATTTGCAGACAGCAGCGATGGTGTATCAGCAGAGATGCAACTCTCCAAATTCACCATTGTCTTCATCGTTTGCAAAGAGGAACGATAATAAAGATACAGAAAAAACCATCCAAGACAATAAAGAATGTCCACCATTGAAATTCTTGGAAGAAAGCTGCCTGGAATTGAAGCTTCTTCCTTCCAGCCATTGCCAAAGTGTCTGTACACTTGACAAGGTTAAGTCTGCTCTACAGAGGGCGGAGAAAGAAACCATTAGCAAGAAACGATCATCACTTCCATCACACATTGAACAAGAAGAACAACAACCAGAAAAAGGTGGTAAAA

>L0296

CGGATTAAATAAAAGAGCACATGTCGAATCTTCAAAATTCTTCCTCTGCTACCAAACGATTTCTAGGATCCCCCAAAACTAATAGTTTTGGACCTGGAATTCAATTCCTAGGGTTTTGAGAAGAAAACCCCAAAAAACTCTAAACAAAACAGATTAACAAAGGATTTCGAAAGCTGTGATTTGGAATTCCACGCCATGGAATTTGAGGTCTCCAGTTTTTCCAATCTTTTATCAGCTTCCTCCGTCGATTTGGTGGTCGACACGAAGAATAAAGGAGAAGAAGAAAATGATCGGGTTCGCCTCAGGAGGAAGAACTTGAAGGCCATG

>L0297

ATTCCTCTCTCACACGCACACACACACAGAGACACGCCTAATGGCTTCTCTTTTCAGAGACCTCTCATTAGGCCATTCCAAGAGAGAGTCAACACCATCGCCTCTCCAACCGCCACCAACAATGCCTTCTAAACTAACTGCCTC

>L0298

ATTGTTTTTTTTTTTTTTTTTTGGAAAAACAAGTTATGCGAGACGGTTGCATATGAAAATACATTATCACACACAAGGAAAAATATCAGCTTGATATTTAGTTCCCTTTTAACAAACGTAAGTCCTAACATGAAAAGCAACTAAACCTCATCCAACGTCAATAAAACTCCAGAAAATAACCAAAAAAAATTTCAAAGCTCTTATGCAACAGTTTGGGCTGCTGCAAGCCTCTTCCTAGCCTCCTCAATGATAGACAACTTAATACCCTTACCCTTGGGAAGAGAGACCCATGGTTTAGTCCCCTTTCCTATGGTGAAGACATTGCCCAAACGAGTCGCAAACTCATGGCCAGTTGCGTCTTGGATATGAATAGTTTCAAAACTCCCCTTGTGCTTCTCCCGGTTTTAAATGACTCCAACTCGCCCTCTGTNCCTCCCTCCGGTGACCATTACAACATTTCCCACATCAAACTTGATGAATTCAGTAATTTTGTTGCTCTCAAGATCTAGCTTGATGGTATCATTAGCCTTGATTAGTGGATCTGGGTAGCGAATAGTGCGACCATCATAGGTGTTCAA

>L0299

GCAGGAATTCGAATTCAAAAACGCCCTGTTAATTCATTTTTTGAAAAATTGGTAGCTTAAAGTTCCCGAGGAAAGCGATCTCTCCACCGTCGACGAGAGAGAATTTATCAGAGGCTGAGAGGGATGACAGC

>L0300

ATTCCAAAACGCAAATGTAAAAAGATAGCGGAAAGGAGAGAGATTAGCTCTGGGTTTGCTGCGACCTACCCTTAGCTGGTTACATCACTTTCCGGATTCGAGAACGACCTCCGGCAACTGAAATATTCGCCGGAAAATCGCAGGCGGGAAGTAGATGCAAGAATCGACTCTGTTCCCATTCTTTCAGGCCAGTCCAGCCAGCATCTACCCATAGTTCCCTCAAACCCAACGACGAATGCGGACCGGTATTTTCGCCGGAAACCAAGCGAATACATATGAAGATGGGTCCTGCTCCTAAACAACCTTCGTTTTGTCTGAAATGGCCATGGGATGTTGAACAACATCCTAAAAATCCTAATGTATGCACCTTTGAGGTTCCTTGGCTATTCAAATCTTTTCATACTCTTGGGTCCATTGCTTTCACTTCACTGAACTCAATTGCCAAATCTTCAAATTCTTGGTTGCATACCTTTAATGCTGTCAAAATGGATGCTGGAACAAGCCAAACTAACAGTTTGAAGTTGCAAAGACAGCACTTAACTCTTGAAGAGCAAGGAGAGGCAGAGCAAAGAGCATTTGCCTCCGCACTAGCAACTGGGAAAGAGGCTACTGTGCCTGGAGTTCTACTCACCC

>L0301

ATTCCAACTTTATTTGCATGCTTCTCGGTCTATGATAGTTGCTCTGATGCAAGGGTGATGTGGGATCATAAAAGTGGCCGCACAAAAGGATATGGCTTTGTTTCCTTCCGTAATCAACAGGATGCACAGAGTGCTATCAATGACTTGACTGGTAAATGGCTAGGGAATAGGCAAATAAGATGCAACTGGGCAACTAAAGGCTCTGGGTCCAGTGAAGACAAGCAAATTGGTGATAACCAAAATGCAGTTGTACTTACCAGTGGATCTTCAGAAGGAGGTCAAGAAAATACTAATGAGGATGCTCCTGAAAACAATCCTGCATACACTACTGTCTATGTTGGTAATCTTTCCCATGAGGTAACTCAAGCTGAGCTTCATTGTCAATTTCATGCCCTTGGGACTGGGGTAATTGAAGAGGTGCGAGTACAGAGAGATAAAGGTTTTGGATTTATAAGATATAATGCTCATGAAGAAGCAGCCTCTGCCATTCAGATGGCAAATGGGAAAATAATTCGCGGGAAGCCCATGAAGTGTTCAT

>L0302

GGCTGCAGGAATTCGAATTCGTCTTTTTGCATGGCCTAATCAAAACGAGCGAGGGAACTCTGACGGAGACGACGAGAATTTGCAGCCATGGCAGAAGATCTGGTGCTGGATACGGCGATAAGAGACTGGGTACTGATCCCTCTATCCGTAGTTATGGTTCTTATCGGCGTACTCCGCTACTTCGTCTCCAAGCTCATGCGCTCGTATCAAGTTCCCGATGCCAAGATCGTCAAAGAAGGCCAAGTAATTGTCAGGGCTCGGAATTTGCGCTCTGGCGCTAATTTTATACCTTCTAAGGCATTTCGTGCTCGTAGGGTTTATTTCAGCAACGAGGAAAATGGATTGTTATTTGTTCCGAAAGGCCAGGCACAAAATGCACAAGCACAAATGTTCTCTGATCCAAATATGGCCATGGATATGATGAAGAAAAACCTTTCTATGATTATACCGCAGACTCTTACTTTTGCTTGGGTCAACTTTTTCTTCTCTGGATTTGTTGCAGCTAAGATACCCT

>L0303

ATTCGTTAATGATGATTCTTAGATTCCTCTGTCCTCTCCTGCTCGAACGATTCGCCGACTGGTGCTTCAAGAATAAGATTCGGAATTACCCCCGATAGTCGAGGAAGAACTGGCGGATATTCATCCGTTGGTGGGTGTTACTGGTGGCGAAGAGGAGGTGGGTTCAGATATCCATGCACAAGCTGAGAATGAGGATACCTCTTTGCCTTCATAATTCCAGGAAACTTGGAAGAACAGGTTTAGAAAATGGGAATGAAAAACCACATAGCAGAGACCAAAAGGAAGTTCTTAAGGTTTCTCCTTATTTTCGTGCGGCCACTGGGCAGCAATTGGGGATGGATGAGATAGAGGCTCCTAAACCATGCCAAAGAGCTGGTCTTGCTGTTCGAAATGTCTCCCTCTGCTTCCAGAAGGTACCCAGGGAAGAAGAAGCTGCTGATAGCAGCATGACTAAGAGCAAAGATGGGCAGAATAAGTCGCTAAGGA

>L0304

CTTGCTGCTGTTAACACTAATGACACCAGCCACATTGTTCATGTTCCTCCTGGTCCAAATGCTCTTTCTGATGTGCTTATCAGTACACCTATCTTCACTGGTGATGGGGAAGGTGGAAGTGGTTTTGCTGCTGCAGCAGCAGCAGCTGCAGCTGGTGGTGTTTCTGGTTTTGAATTTGGTGTGGATCCCAATCTTGATCCTGAACTGGCCCTTGCTCTTAGAGTTTCAATGGAGGAGGAGAGGGCCAGGCAAGAAGCAGCTGCCAAAAAGGCTGCAGAGGAGGGCTCTAAGCAGGAAAAAGGAGGGGAACAACCATCTACCTCACAGGACGCAACCATGACTGAAAGTGCCAGTGTTGCAGCTTCTGAAGCTGACAACAAGAGAAACGATTTAACGGATGAAGAGAATGCTCTCCTACAGCAAGCTCTTGCAATGTCAATGGATGAACCTGCCTCTANCCATGAATTGCGAGATACTGATATGTCAGAGGCTGCTGCAGATGAT

>L0305

TGTTAGATTGGGAGCTAACAAATATAGGGAGACGCANCCCATTGGTACGTCAGCTCAAAGCCAAAATGACAAGGACTACACTGAACCACCANCAGCGCCGCTGTTCGACCAATGGAGCTCACTTCTTGGTCCTTTTACAGGGCTGGTATTGCANAGTTCATANCCACTTTCTTGTTCTTATACATCTCTGTTTTGACTGTCATGGGTGTAGTTAAGGCACCCACCAAGTGTTCGACTGTTGGGATTCAGGGGATAGCTTGGTCCTTTGGTGGCATGATCTTCGCTCTTGTCTATTGTACTGCTGGCATTTCAGGAGGTCACATAAACCCGGCGGTGACGTTTGGGCTGTTTTTGGCAAGGAAACTGTCCTTGACAAGGGCTTTGTACTACATGGTGATGCAGTGCCTTGGAGCCATATGTGGTGCTGGTGTAGTGAAAGGATTAGAAGGGCGTCACCAGTATACTTTGTTGGGTGGTGGTGCCAATAGTGTGAACCCAGGTTACACCAAAGGTGATGGGCTTGGTGC

>L0306

GGATCCCCCGGGCTGAGGAATTCAATTCGTTGAAATTGGTACTCGACACCCCGAGGCCGCCCTCCTGGTTCCAAAAACAAACCAAACAACCATTTTTGTCACCCGTGCACAGCCTAATGCCCTCCGCCACCATGTTTTGGAAGTTGT

>L0307

ATTCCTCTTCTACAAGGTGCTTGTAGCCATGGATGATCATAACGATGAAACAAATCGTCAGGGATGTGGCTTCTCCATGAAGCATGGCTCCTCCTATCGCCTTCTTAGCCCGGCTTGATTGATCGCCATGAATCAGTTCTAAAAGCCGATCATACATATAGTCTATACTGTTGTTAATAAAACTTTTTTCTTTTTTTTACCCTTAATTATGAAGAATAAGGCCTATTACTTTGTGCCTTTCGTACTTCATTAAAGCTTTGTTAATTATGTGTGGTCAAATCATGTACAGTTGGAAAGGATGAGCAAAAGCCATAAAATCCAAGTCATGTAAGATCTAAAAAAAAGAGAGAGAGAGAACTAGT

>L0308

GATATTCCAGAAACTGGGCTGTTCGGACAATGGCAAAAATATGCATAGAAGCGGAAAGCACACTTGATTATGGTGACGTCTTCAAAAGAATCATGCAACACTCACCTGTGCCCATGAGCCCATTGGAGAGTCTGGCATCTTCTGCTGTTAGAACAGCCAATTCAGCAAAAGCATCCCTTATATTGGTCTTAACCAGGGGAGGAAGTACTGCAAAACTGGTGGCTAAATACCGACCTGGCATGCCTATTTTATCTGTGGTTGTACCTGAGATTAAGACCGATTCCTTTGATTGGTCCTGCAGTGATGAAGCTCCTGCAAGGCATAGCCTTATTTTTCGTGGGTTGGTACCTGTTTTATATGCAGGATCTGCTAGGGCTTCTCATGCTGAGACAACAGAAGAAGCGTTGGACTTTGCCATT

>L0309

TGGATCCCCGGGCTGCAGGAATCGAATTCATTGATTTTGGTAATATTACAGGATGAGTTGTTGTGGGCTGCTGCCTGGTTGTATCAGGCAACTAATAATCAGTACTACTTGAGCTACCTTGGGAACAATGGTGACTCAATGGGTGGAACTGGATGGGGCATGACTGAATTTAGCTGGGATGTCAAGTATGCTGGGGTTCAAACTCTTGTTGCCAAGTTCCTGATGCAAGGCAAAGCTGGACATTATGCACCAGTCTTTGAGAGGTACCAACAGAAGGCTGAGTCCTTCATGTGTTCATGCACTGGAAAGGGTACCAGGAGTATTCAGAAAACTCCTGGTGGTCTAATTTTCCGCCAGAGGTGGAACAACATGCAGTTCGTCACAAGCGCCTCCTTCTTGGCTACTGTCTATTCTGATTATCTTGCTTCTGCTGGTAGAAGCATCAAGTGTGCTTCTGCCATTGTTACCTCATCCGAGCTTCTTTCTTTCGCGAAATCTCAGGTGGATTACATTCTTGGAGA

>L0310

ATTCATGAACTTACTGCCGAAGAAGAAGTAAAAGCAGTTATGGAAAAAGAGATCGGTGAACTTGCTATCAAAGAAGAACAAACAGAAATAATAAGGGAACTAAGGGATCTTCCACCAGCTCACTACATATTCAAGATTGAAAACTTCTCTTACCTTTCTAATGCAAAGGTAGAAAGCTATGAATCCAGTGATTTTGAAGTTGGTGGATACAAATGGAGGTTGTCTCTTTACCCTAAAGGAAACAAGAAAGTAAATGACAAGAATGAACATGTTTCTCTGGTATTTAGTACTAGCCGAATCAAATGCAGTCCCCATGCATAANAGAGATAAATGTGCACTTCAAGTTGTTTGTNTATAATCAAATTCAGGATAAGTATTNTGGTTGTACANAGATGCAAAGGACAAGGAGCTTGCTAACAGCTATACCTACACTTGGAAAATTCAAAAGTTTTCAAATTGCAACCAAGCAGGATATACATCTCCGGTGTTTGTTATTGGTGGATACAAATGGAGTTTGACGCTTTA

>L0311

ATTCGTTTTTTTTTTTTTTTTTTTTTGGGCAGGAGGGGAGGGTGGTTGGTGCGGTGTTCACAGCATCTAATATGGACTTATAGTTACTTGCTGATTCTGTTGTCTCTGCTGTACAAAATTGGAGTGATTTTGTGATGCATTCTGAAGTGCCAACATACTGGACTGTAAATTCTGTGCATTTGGTGGCATAT

>L0312

ATTCGCCCTTCTTATTTACTTCATCTTCACATTTATCCTCCAAAATGGGTTCTCGCTATGAGGTCGAAGTAACCATCACGTCAGCCAAGGACCTAAAGAACGTCAACTGGCGCCACGGCCCACTGAGGCCGTACGTAGTCGTTTGGGTTGATCCAAACAAGAAATGCTCCACCAGAGTCGACGAGGAAGGTGATGCATGTCCTCTATGGGATCAGACCCTTGTTATCTCCTTGCCGCCTGGCCCAATTCAGGATCACACTCTCTACATCGACATCGTCAACGCCGGTGCTGAGGAAGACACCAAGCCGCTGATCGGCTCCGCCCGGCTAAAACTCGTCGACGTTCTCGATGACGTCGGAGTTGGCGAACGAGCTAAGCGCACTCTCCAGCTCAAGCGTCCTTCAGGTAGACCCCAGGGGAAAGTCGATGTTAAAGTTTCGATCAGAGAACCACGGTATCGTGCGCCT

>L0313

ATTCCGAAATGTGGGGAAGGGTTTTTATATAGGTTTCTCAAATTTTTCCAAATTGAATTACTTTCAAGGTAAAATTAGCGTGCCATTTACATGGCTCAGATATTACCTGCTTTGGTTACATGAAGTTCGGCAGCTAATGAAGTTAGAAGAAAATTGCAGTGTGGAAGATTTTGAAAAACCTTTCCCTCAGAGACGCATCCTTCCATTTTTGGAGCTCCCTGACTTCTATTTATGCTTACCAGGGAGTGGAATTTGCCATCAGAGACCTTGCATTTCATTTTTGGAGCTCCCTGAGTTCTATATATGCTTACCCGGAAGTGGAATTCCTGACTGGTTCACGTATCAGGGAGAGGAGAGTCAGTTGAGAATAGAACTACCTGCAAATGATGAGTGGTGGAATATTGCAGGTTTTGCAGTTTGTGCTGTTGTTGGGGAGAGAAAT

>L0314

TCGAATTCTTTTTTTTTTTTTTTTTTTACAGAAAGAACATATATGATAAATTGTATAAATTACCATAAAATAGGTTCGTTTAAAACACAACTGGTTAACTATTCTATGAATTCAACAATAAAACTAGTAGGTAATAAAAGCTTAGTTGCTCATCTCATAAAAATTACATTGTCCAAATATAAGGGAAATAATAAGTTGGGAATAAAATCTGAATATTAGATTCAGATTACAAAATTTTAGGCAGCACCAGCTCCCTTCCCCTTCTTCCTCTGGATTTTCTTCATGTAGAACTCCACCTCTTTACCCTCCAAGATGTATCCATCAGTACGGCCACACTGACCAGGGCGGGATGAGATACAAGCTAATAGACGACCACCACCAAATTGGTCTTCAATATGAGGATCAAGCTTGCGAGTCTGCTGTCTCTTCTCCAGCTTTCTCAAGACATGGTTA

>L0315

GAATTCGCATGTCGGATCCCTATCATCCGCGCACTTCATACAATTAGGGTTCAATCTTTCTAAATTCTTTTCCCAACAACGAACTTCATTCCATTATTGAAATTATCAGCCCTTCACTGAGATCATAAACAATGGAAAACGACAACAAGCCACACAAACACCACCGGCATCGTATCCATGGAGAACCTCAATCAGGTTGCCAACTGGGTTAGCGCCACCGTGATCTCCGCCTTCTTCTCGTC

>L0316

ATTCCCCTGTTGTTACACCAGTCCCACAAGAAGTAACAACAGGGCTTTCCAGAGAGATGCCTTCTTGATCAAACTGTTTCTTAAGTTCATCTGCTGGTAAGAGTGTCTGTGAAGCATCTAACATATGAGGAAAAGGAATGCACTTGCTGCCAGGTACATGACCGCTTCTTATTCCCTTTCGAGGCTCTGGTGCTGCTCCGTCAAATCTGGCCTTTGAACGAGCATCTATCTGTTGGTGAGACTTGTCCTCAATGTTTTTCTTAACCTGCTCAAGAGTCCAAACAAGATGTGGCTGAAACTTTGTCTGAAATGTTATTGGTCCAACTGCCTGGCCTTGATATACTTTCTCTATTGCCTCACTGGCAGCACTAGCTTTCAAGAATGGATCACCAGAAGCACTAGATTCAACATCATAACCTGATGCACGCCATCTTGGCAGGCCTCCATCCAACACCCAAACTTTGTCATGCCCAAAGAATCGGAACATCCACCAGAAACGAGCTGGACTGAAAA

>L0317

ATAAAAATGAACCATATATTAAAATCTTGGATGGCTCTCTTTGCTCATCCTTGCTGTCTATTTTAGCAATTTTAGCATAAAGAAACAAAGAGAAAAATTACAACATGAGCTCATATCTTTCTACAACAACCCTTTCTCCTTTATGAAGCACGATAAAAGCACTGACACAGGGATGAGGGGGAAAAATTAGATTAATCTTACACAGCAACTGGTGTGGCTTCATCAAGAGCAAGAACACCTGGAAGTTCTTTGCCTTCCAACAACTCCAAACTGGCACCACCACCAGTTGATATGTGGCTCATAACATCAGCAACTCCCTACTTTTTCTACAGCTGCAACAGAATCTCCAACTCCAATAATTGTTGTGACTCCCTTGCCCACTAAGCTCTGCNTAGCTTCTTTGCAATAGCCTCTGTTCCCACTGCGAACTTGTCAAATTCAAACACTCCCATTGGTCCATTCCAGATGAACAGTTTGGTAGTTTCCAAAGCATCACTGAATGTCTT

>L0318

ATTCCAACAAGGGGGAAAAACTGTACTAAGACACCCAAGAAGAAGAACTGCAGCTAGAATCAGGACAACCCTACAGCAAAAACAAATCTATTAGCAGGATCCTGATAAAGACAGCAGGAAAACCTAATCCTATCTATATCATCCTGTTTACATGGATTTGGCATCCTCTTCAGCAAACCCAACCTTTTCAAGCCCAATAATGGGTGGCTCGATCACGCCTTCCAGGACCTCCACCACTTCCACCACACCAAACGCAACCTCTTCCCCATCTTCCACTCCAAGTCGCTATGAGAACCAGAAGCGGAGAGACTGGAACACTTTCTGCCAGTATCTTAGGAATCACAGGCCCCCTCTTTCGCTTCCCATGTGCAGTGGTGCCCATGTTCTTGAATTCCTTACGTATCTTGATCAATTTGGCAAGACTAAAGTCCACACTCAAACTTGTCCATTTTTCGGCCTCCCTAACCCACCAGC

>L0319

ATTCTATTTATTTTTTTAAGGGCAAATTAGGGGAAATTTTTTAGTAATTGAACAAATTTGATAAATAAAATCAATCGGTGGAATCGGGAATTGGTAGCTATGGCGAAGAAGAGGAAATCAATAGCCACAAGCCTGGACGAGGTGGATCGGACCATGTATGCTTCATTTTGCAGCGCTGCTAACTCTCTCTCTCAGCTATATACCCAGGCTATGAACCACCAGAAACTTTCCTTCCAAGCCGGTGAACGTCACGGCCTTGAACAACTTTATCAGTGGATTTGGAGACAACAAGAGGGAGGATCAAGAGTAGCAACAGTTGATATACTCATTACATTCAGAATGAGCTGGACTATTGTGGAGAAGAGCTGCCATTGTCCCCCCGAGCGCCACAACATCAGAATTCCCAACCAATTCAGTTCTCAAATTCCAGCTTCCTTGTTTCATCAGGTTCGTCTGGTCCAATAACTGCTGGCCAAGGTATCGCACTGAGCATGTGATCAACAACCCAAGAATCAGTTTTCTCAAATGTTTGTCAGCCTGTCCGGAGG

>L0320

ATTCAATTTTGGTGTTCTCCAATCCGAAATATTAGATTTTGTTTCTGATCCATCCTTAATGGCCACTGAAGATCCAGCCGCTGCTGTTGAGGCCAATACTGAGCCGGCCACCACTGAGCCTGCAGAGGAAACACCGGCCACCAAGACCGCTAAGGCCAAAAAGGCTAAGGAACCCAAAGCCAAAAAAGCTGCTGCACCTAAGAAACGTAGTCCACCATCCCATCCTCCATACGAAGAGATGATTAAGGATGCCATTGTGACTTTGAAGGAAAAGAATGGTTCAAGTCAGTATGCGATTACTAAGTTCATTGAAGAGAAGCAGAAGCATCTGCCTCCTAACTTCAAGAAGCTGTTACTATTCCATTTAAAGAAGCTTGCAGCTGCTGGTAAGATTGTGAAGGTGAAAGGATCATTCAAGCTCCCACCAGCAAGGTCATCGGCGCCAAAGCCTACTTCTCCTGCTGCTGCTCCGGCCAAGGCT

>L0321

ATTCGTAAAAAAATTTTCAGGATGATCCTCATGTGAATTCTTCACTTCTCTCACATTCTCTTCCAACTTATCGTTGGTCCTCTGATTAGATATTTGCTGTGAGATCGTTGAACCTCCCTTGATGGGTGCTCCTAAGCAAAAGTGGACAGCAGAAGAAGAGGCAGCTCTTAAAGCTGGAGTGCTTAAGCATGGGACAGGCAAATGGCGTACAATACTTGTGGATCCTGAGTTTAGTGCCATCTTGCGGCTAAGGTCAAATGTTGATTTGAAGGATAAATGGAGAAATATAAATGTAACTGCAATATGGGGGTCCAGGCAGAAGGCTAAGCTTGCACTTAAAAAGAGTGCACAGACCCCTAAACGTGATGATAATCCTAAGGCTTTGAGCGTTATAGTTCAAGGAAATGAAGAAATTGTTGATGCTAAGCCCTATAGCAATTTCTAGTGGAACACCGAGGAATGTTGGTTCAAAAGAACTACTTGCAAGGTTGGATAATCTAATATTAGAGGCTATTACAA

>L0322

TTTCTCTTTCAAAATAACCAATCCCCTCTTTCTTTTTCTTTTTCCTTACCAGAGACTTCATAATCACCAATTCAACATGGCATATGAAGATCAATACCAGCAATCCCTGCTGCTAAAATATGACACTCTTCTCTTTGACGTGGATGATACCCTGTATCCCTTAAGCACTGGTTTCTCGAAGGAATGCGCCAAGAACATTGAAGAATATATGGTTCAGAAGCTTGGTATTCCAGAAAATAAAGCCTCTCAATTGAATCAAGAATTGTACAAGAACTATGGAACCTCAATGGCGGGTCTCAAGGCTATTGGNTATGATTTTGACAACGATGAATATCACAGCTTTGTTCATGGAAGATTACCTTATGAGAAGCTGAAGCCTGACCATGTTCTAAGGAGTCTTCTGCTTAGCTTGCCTGTCCGTAAAGTTATATTCTCAAATGCTGATAAAGCCCATGTGGCTAAAACTCTAAGCAAGCTTGGATTGGAGGACTGTTTTGAGAGGATTATATGCTTCG

>L0323

AGCTGTGGTTGCTAATCACAAAGGAGACCAACAAGGTTGCGACCTGTTTTTGAACCTTTTCCTTNAGGTAGCGCAAGAGAGAGCCCTCACAATCGGACCTGAGGAAGGTGGTTTCGGAATGTCATATGACCTTATGTATGGACGAGCTGGGTTTTTATGGGCTGCTTTGTTTATAAATAATCATCTTGGAGAAGGGACATTGCCAAATGATCTTCTTCTGCCTGTTGTTGATGCTGTATTAGCTGGGGGCAGGGCAGGAGCATCTGATAATCCAGCCTGCCCCTTGATGTACAGATGGCAAGGGACAAGGTATTGGGGTGCAGCCAATGGCCTTGCTGGAATCTTGCAAGTGCTACTTCACTTTCCTCTCTCCAAAGAGGATGCTGAGGATGTTAAGGGGACTTTAAGGTACATGATGAATAACAGGTTCCTTCATAGTGGAAA

>L0324

ATTCCCGCGAACCGCCTCTCTTTCCATTGGCTTTCCGTCTATTGTCTGGCCAATTCTCCGATCAGATGGATAGTGAAACTGGTAATCAACACAAGCAAGAACGATCAAGAACAAGATGGACAGCTTCCCTTGATAAGATATTTGCAGACTTGGTGGTGAAACATATTCAACTTGGAAATAGACCAAACAATGTTTTTGATAAGAAAACTTGGAATCACATACGTGATGAGTTCAATAAGCAGACAGATCTAAATTTCAATAATAATCAATTGAGAAAACACCTAGACGTACTGCGGACCCGCTTCTATAATTTAAAGTCGGCTATTGTTCAAAATGACTTTGCAATGGAGGATTCTTGCTGCATTGGATTTGATCTGTGTGATGACGTTGGGTCACAATCA

>L0325

ATTCATCCCTTCTCCACCCTCTCAAACCCTAGCGGACTGTCACTGCCCAACGATCAATCAACAGCCCTTTACCACCGCCGTCTCTTGATTCTTCTTCTTCTTCTTCTTCTTCTTCTTCTTCTTCTTCTTCTCGAACCAACACTTCNTGATTGCAAGTTTTGATTCTGAAACCGATAAGTTCGTAGATGGAGGACTGGGAGGATGAACAAATTCCACCTCTCCTTGCAAAGGAGCAACCTAAAAGTAAATGGGATGATGAGGATGTGGATGAAAATGAGGTGAAGGAATCATGGGAGGATG

>L0326

TGCAGGAATTCGAATTCAGTTTTTTTTTTTTTTTTTATTTATTTATTTTTCCTTCCTGTTTGAGGACCTTGTTGTTCTTGTTGGTATGGTTGTTGAGGTTGATCTTTGTTCAGCTCGGCTCTTCTACCCCTNCGTCGCAGCAGTACTCTGTACCGCTGTCTGTTCTGCCATGGATGCGTGCATCATGAAAAAGGCAACAAAGTGCCCCCTCTCTCTCTCTCTCTCTCTCTCTCTCACCCCCCTATAT

>L0327

TCGTTTTTTTTTTTTTTTTTACACAAGTAAAACTTACTGAGCTTTGTCATTAGAAAAATAGGTCAACCACACAGCAAAATACAATAATAACCACCCTGAAACAATCTCTTTAACATTTTCATCATCATCTTAATTTTCATTTGAGGAACCTCCCCTGATAAGGGATCCACGGCGATGGCCACCCACAATCACATCCTTAAGACGTGATATAGGCCCCAGCATCCTGCCACTTTTCTCATTAATGCTAGCCTGCCACTTGAGTTGCAAAACCCGCCACCTCTCCTGTTGCCAGCGCTGTCCAAGAATTACAAGGCCCATAACTGCTGCTGCAACAAAAATGGACCAAAATGCAGAGAGAGAGAGAT

>L0328

ATTCCTGTGATATTTTTCTATAACTTGGTGTCTCTGTGTCTCTACGATCACGATCCAACGGTCCAAAATGAACGGAGCTCCTTACGCTTATTTCTCCAGGTTTTANGCCCCAAACCTCCAGATCTGTTCTCTCTGTGCTCAGATCTTCAATTTCGGTTTAATATTTGTATTTCTTTTTTGACGCACGCAAATGGAGGCTAGCTACGGCTTAGTGACAGTTGCGCTAATTAACGGTGCACTAACGTGCGCCGTTGAGGCAATAGCTAGTTTGCTAGTTTCGGCAGCATTGGTCAAAGCAGCCCAGGATCCCGTGGAATTCTTTCTGACTAACAAACCTAGAATGAAAGGAGATGACTCACATGGCCAAGCCAAGCCAGTTGATGCAGGTGGAGATGATGGCAATGAAGATGATGACGATGATGATGAAGGGGATGGGGAATTTGGAGAAGGCCCAGAGGAATTATCATCTGAAGACGGAGGGGACAATGGCAACAAC

>L0329

ATCCCACCTTCCCCTCCTAAAATCCCATAACCCCTTTTCTCTCTCAACTGATCTCTCTGTTTCTCTCTCTTTCTCTCTTAACAATCATCACCACCTCCTCCAAGTTGTAATTGATCTTGCCTTCTGTGATCCAAGNAGCAAGGTGATAAATGCTAAAGGCTGTCGTCTAAGCCATTGTGGACTGAGACAAAGACTGTAATCCTCAATGGTTAGCTTACGAAGGCGTAAACTCTTGGGACTGTGTGCTGGGAGAAGTTCTTTCTTGACTCCACTTCCTCGATTTTTTGATCATGGAACAGCTCCTTTAAGCTCTACTCAGAATGCCAGGTTTGTCAGTGTACATCCTCTGCCGTCAGATGATGTCAAGCAGCCAGGGGAGGTAAAGAAAAGCATTTTCA

>L0330

CAAGTCGGAGTCCACGATTCCAAACTGCTGCCGGATCCTTTCATGGACCGACACGTCACCTGGCAATCGATTGCGTACATAATGACAATCGAGGCCGAGGAGAGGAAAGCAGAGGAAATGGAGAACCCCCTCTCGGCCCTTATCTATTTCGGAGACTAATTAATAAATGAATTAGGTACAAAAAAACCCTAATTCCATCCGAATTTATAAAATTTCCTAAATTCTTAGGTTCAGAGAAAACGAATCAATCAGGACAAGTATCCAAGGAGATTTTTTCTTTTTCTTTTTCTTTGGGGATTTTGTTGAATTTGGGTAGATTGATCGATCCGATCGGTGCTGCTTGAACACGAAACTGTGGTTGAAGGAGGAAATTAGGGTAGAGGATAGGTAGATAGATAGGAGAGAGAGAGAGAGAGAGAGAGAGAAGAGAAGAAGAAGAGACGAGGAGAAATAGAAACTAT

>L0331

ATTCCGGATATCTCGGCTCTCGCATCGATGAAGAACGCAGCAAAATGCGATACTTGGTGTGAATTGCAGAATCCCGCGAACCATCGAGTCTTTGAACGCAAGTTGCGCCCGAAGCCATCCGGCCGAGGGCACGTCTGCCTGGGTGTCACGCAAAAAAAAAAAAAAAAAAAAAAAAAACAAAAAAAAAAAAAAAAAAAAAAAAAAAA

>L0332

ATTCATCATACCAGCGCTTCCCAAGGGTGAAAATCCGAGAGCTCAAAGACGACTATGTCAAATTCGAGCTCCGTGATACCGACGCCTCCATAGCCAACGCTCTTCGCCGTGTTATGATCGCCGAGGTCCCCACTATCGCCATTGACCTCGTCGAGATCGAGGTCAACTCCTCCGTCCTCAACGACGAGTTCATCGCTCACCGCCTTGGCCTCATTCCTCTCACCAGCGAACGCGCCATGAGCATGCGCTTCTCTCGCGACTGTGACGCTTGCGATGGTGACGGCCAGTGTGAGTATTGCTCAGTCGAGTTTCACCTACGTGCTAAATGTATCACTGACCAAACTCTAGACGTCACGAGCAAGGACTTGTACAGCTCTGATCACACTGTGGTTCCTGTCGATTTCTCTGATTCGGCGGGCTATGATTCCTCTGAGCAGAAGGGAATCATCATTGTGAAGCTTCGTCGTGGGCAGGAACTGAGACTGAGGG

>L0333

ATTCCTCTTCACTCAAGCTCCTCATTTCCTCCCTNGAACCGCACACCACCGGGCACCGGCGAGGAAGGACGTGAAACGGAGCATGAGGTGAATGATTTGTTGTTCTATTGTCTTTCTTAAATCGTCTGCTGTCTCGTTTCCGGACTGGATCGGCTTGTCGCTGAGTTGATTGCTTGTAGCAAAGAAGAAGCAACACTGGCTGTGATGAGAGGAAGCCTCTTGGTTAACCGTCGCGGGATGCAGAGATTTCGGCAACTCGCGATCACTGCCGCTGGATCGATTAAGATTAAGCTCTTACTCTGCTGTTGCATAGCTTTTACGCTGCTCGCGCTCCTGGACGCGCATCGGATTCATGAATGGACCAAACAGTTGTTTGTGCTGATCAGCTCTC

>L0334 GGCTGCAGGAATTCGAATTCTTCTCAATCGCATCGAAAATGGCAGGAGAAGCGACTATGTTTAAGTTCCTTAAGCCTAGGCTTCGTCCTCAATCCACTGATATCCAGGCCGCTGCCTTTTGGGGCGTCGCCGCCTCCTCCACTGCTCTTTATCTCATCCAGTGCTTGTGTGAAATTCAAGTTCTTCTGCCCACCTTGTGAGAACTATGGAAGGATGTTTACAAGATACGGAACTTTTGCCATTTACTGGAAATTTTTTGCCATTTGATTGGATTAAGAAGACCTTTTTCGAGAAGGGTGAAGCCGAAGGAAAGTGAATGGGGATCAGATTTCTGAATAGCT

>L0335 GGAATTCAATCGTGAAATCTCTTAGTTAAGCCGGAGTCAGATTATTTCACTTCGCTTCGATTTTTTCTTCTTTTCTTTTCTTTTCAAGCGCCAGGAGGATTTGAGATGTTGGGTGTGTTTAGCGGCGCCATCGTTTCTCCGCCGGACGAGCTGGTGGCGGCAGGATGCCGGACGCCATCTCCAAAGATAACGTCGGATGCGCTGGTGAAGCGCTTCGTCGACACCAACCCCTCCTCTGTATCCTTACAGATCGGAGATAACGCGCAGTTGGCCTACACCCACCACAGCGAGTCCATGCTGCAGCCCAGATCATTTGCA

>L0336 TGCAGGATTCGAATTCCTCAAAGAACGTGTTCTTCGATGACAGCATCCGTAATTTACAGACTGGGAAACAGCTGGGCCTCAACACTGTGTGGGTTAGGCTCTTCCCATCGCACTGAAGGTGTAGACTATGCACTTGAGAGCATCCACAATATCAAGGAAGCACTGCCGGAGCTCTGGGAGGCTAATGAGAAGTCTGAAGATGTGAGGTATTCAGGGAAGGTTGCCATTGAGACTTCAGTTAAGGCATAGCATATTTGTAGGTGGGCATTTTCTCAACTCTATCTAATGCGGTCACACGATGACATCTTATTTGTTTGAAATTAAACACGCTTTTTTTTCCCCTCAACTTTTGTAACATTTGAGCCATTGAGCTCTCACATTAGCTTCTGTTGTTCTTTGTTTCTCTGATTTGTAGTTTAAGTGATCTTAGCTTCCT

>L0337 ATTCATAAAAAGAAAAATCGAAAGCGTTTGGATTGGTGGGTTTCGTTGGACGAGGAGAAGGAAGAGAAGGTTTTGAAGAAGGAGAAGAGAAGGCCGCCTAGGGAGTGGTGGAAGGAGGAGTATTGCGAAGAATTAGAGAGAAAGAAGAGAAAGAAAAAGAAGAAACGAAATATGGGGATGACCAGCGATGACAATGGAGGCCAAGAAGTGTGGTGGCCAAGGGATGAGGAATTGCAAGTTGAGAGAAAGAAGAAGAAGAAGAAGAGGAGTAGGAGTAGAAGTAGTCTAGGCAGTATTGACTGGTTTAGCGGTGAGCTATTTAGAGGTATCTACAACAGCCATGATTCTTTAAGCGGAGAGATAGCAA

>L0338 ATTCCCAAAAGAAAATAACCCGAGAGATATCAAGGAGACATCGAAACTACAGACTCTCTCTGCGATCTCTGATTTAAACGACATTGTTCTTTTATCCGTTAAACAACCATGTCTTCTGGTTTCAGTGGCGATGAAACTGCACCTTTCTTTGGCTTCCTTGGCGCCGCTGCCGCTCTCGTCTTCTCCTGTATGGGAGCGGCATATGGAACAGCTAAGAGTGGTGTTGGTGTGGCATCGATGGGAGTGATGAGGCCTGAACTTGTGATGAAGTCAATTGTGCCAGTTGTTATGGCTGGTGTGTTGGGTATATATGGGTTGATTATTGCAATTATTATCAGTACTGGAATTAACCCTAAGGCCAAGTCGTATTACCTGTTTGATGGGTATGCACATCTTTCATCTGGTCTTGCTTGTGGCCTTGCTGGGCTTTCTGCTGGAATGGCCATTGGAATTGTTGGTGATGCTGGTGTCAGAGCTAATGCCCAACAACCAAAGCTTTTTGTTGGAATGATTCTCATCCTCATT

>L0339 ATTCCTCAGTTTTTTTTTTTTTTTTTAGATTAGCCAACAATCCAGCCTTGCAGGAATCGCAATTGTAGCAAAGTTGGTTTTGGTCATTGTTCCAATTCAGGCAGTCCATATCTGCAGCATTGTTGATGGGACTAATCCAATACGTTGGGTTCACAAAGGTGTACCCACATTGCGTAGGAGGCTTGCAGCATCCTGACTGCAAGGGGGTAATACGAGCATTAAAGAAATCTTGAGCCATGCGATAACTCTGGTTCAACTCAGAACACATATCGGTTGCACTAAGGCAGCTTCTTATTCGATCCCACTTGTAAGAACTTTGAACCCTTCGGCGAAGCCATCCTGAAAAATCATCGACGTGATACTCCAAGTAGGCTCTACTTGGTTCAAGATGACCTGACCCCCTGACGGTGACCATATAGATGAAAACCACTAAACATGCAAGCAATATTATAAGGATGAGCATTGCTATGAGATAGAAGATAAGGAGCCATGGGATTCTCCAAAAACCTCCAACAAACCCTGCC

>L0340 GGAGCTCAATTCCACACCTTCCCTCGTTCTCTCTTTGACACAGTCACCAAAAGGTCAAACTTCTCTACCCGGCCAAAAACTCCTCCGGAACCTAACCGGAAAATGACCACATATGGCACGATCCCAACCGCATCGTCGCCGGGAGCCACCACCAACATCGAGTACATAT

>L0341 TGCAGGAATTCGAATTCTTTTTTTTTTTTTTGTCAAAGCACACAGAATTATGGTGTGAACTATTTTTTATTTCCCCTCTTTGATTAACCAGCAAGTACCATACAAAAGTAAAAAACTATCAACTAGTTACACACTACATACGATATACAGTATCAAAAGCTGCACCTAAGTTGGACACCAAAGCCTCATTGCTGAACACCCCGACTACCCAAATCAGCAGCAACTTTGGCTGCCCGATTCAAAGCATCCGACATCCAAAGAGCTCCCTTAGAGAAGTAGCTACTGTTTACCACAGTGTCTGCTGCTGCAGCAGCTGTTCTTCCTGTTGCTGATACAGCTGATTTGGTCATTTCAGAGACATGGTACTTCTGATCCACAGCTTTAACAGCTTCCATGCCAGCAAAAATTTTATCAGCAAGGCCAATTCTCTCAGTCAGCTCGGCAACCTTAGCTGCTGCAGTAGCAGTAACTTGATGAGATTCATCATAAGCTTTTGCCTTGCTTAATGCATCTTTTCCCAACACATATCC

>L0342 ATTCCTGCACAATCCCTACAATTTCATCCCTATCCCGCCAGACCGTTTTTCTCTACCACGTCCTGCCTTATGACCTGTTTGCCTATACCCTTACAGTCGCTCCCCCACTCTCTCTCAAATTCACATTCTCACTCTCGTCTCCTTAATTTCTCACAATTAACCCTAATTTATTTTCCATTCAGAACCCTAATTTCCCCCTCTTGTATGATTACTTGACTAGCCTCCAAAATCTCCGTAAAGCCATAGTTTTTTTTTTTTTTTTTGCCTTTGTAGAAAATTTCCCGACGAAAAGAATTCTTCAATTTCTGCTCAAGAATTGTCTATTCCCCTATTTGTGCTCCCTCATTTTGAACGAATTCAAACCCTAGGTAGATTTTCTTGGGGGAGTTTAGCAAATTCTGCGCAGGCTCCATAGAAACCCGCATGATAAAGCTCGGGTATCTGAGGCCGTTTCTGTATTTCTCATGGAGTCGAATCCCGTGATTTTCGATATCAGTCTGTGAGGACCGGCTTAATGAGCC

>L0343 ATCAGAAGTCCTGTTCCAGCCATCACTCATTGGAATGGAAGCTGCAGGAATCCATGAGACTACTTACAACTCCATCATGAAGTGTGATGTGGATATCAGGAAGGACCTTTATGGTAACATTGTGCTCAGTGGTGGTTCCACTATGTTCCCTGGCATTGCAGACCGTATGAGTAAGGAGATCACTGCCCTTGCTCCAAGC

>L0344 ATTCGTTTTTTTTTTTTTTTTTTTTTTTTTTTTTTTTTTTTTTTTTTTTTTTTTTTTTTTTTTTTTTTTTTTAACAATAATCACGGGTATAACGATTTGTTATTAAAAAAACCCCAAAAAATCCCACAAACAAAACACAAATTGTTGAACACTTGGGGGCCTTGTGGGTTTTGGAAAGGGGATAAGCAGGGAAGCGATACCACAAATAGCGAGGGGAATTTTTGATTGGATAATCAAACTGGGAGGTCAAAAACATCTGTCGCGTTGATGGGGGTTGGTGGTGAAAGCGAAAGGACTGTCCTTGGCCTCATCTTCATTATCATCGTCGTCGGGGTCGGTGGTAGCAACATTAACGCAAAAAAAACG

>L0345 AATTCCAGATATTTATTTTCTTTTAAAGGAAAATTTATATATATATTGGCATGCGAGTCTGGTTCACCGTTTTCCTCGTCTCTGTTTCTCTCTTACCATTTTTCATTTTTCTCAAGAAATAAAAACTTTTGAAGTAGAAGATGGGCACGACTGAACCGAGCTTGGAACAATTTTTTGAGGAGAAGAAACGGGTCAGGAACCCTCTGGTACCCGTTGGTGCGCTTATGACAGCTGGAGTGCTTACAGCTGGCTTAATCAGTTTCAGGCAAGGCAATTCTCAATTGGGTCAGCTTTTAATGAGAGCTAGGGTGGTGGTCCAAGGAGCTACTGTTGCACTTATGGTTGGAACTGCTTTCTACTATGGGGAGAACCCCTGGAAAAAGTCAAGTTAGAGTCCATAAACCACATTCCGACTCCTGTAACTTGGTATGGATCTACCACAAATGGGTTCTAGTGGTGGATGCCATCTTCATCGTTAACAAATGGTAGCATTGGTTCTTAAATTCCATACTGCATCCATTATATT

>L0346 ATTCCTGAAACCCCCATTCTCTCTCTCCCTTTCAACTCTACGAACCCTAACCCACGATGATTGCTACTTGCTGTTCTCGCTCCGACATTTGAAATTCTTCGAGCAACATCTTCGAATCTCTAGATTTTTCGGTTTGCTTGACGGAGATTTCACGTTCATTTTATTTTTCGCTGAAAAGGAGGGTCTACAAGTTCGCGATATATCTCTTTGTCTATCGAGATTTATTCAATCAGCGATCGAGGATTTTCGCTGGCAGGTGTTGGATTGTCGGATTGGTTGTTGAAGATTTGGATCGGTGGAGTTTTATCTGATTTCCTTATTTTGGCCCCTCTAAACTGAAGGTTGGGGTTTTTTCCTTCTGTTGATGCGATCTGCGAATTGTCATTGAAAACCTCAGATTTGCCCCGAGTGGAAGCAATATTTCTTGGATGCGATGAATTTCAAGCAGCTATAGCTGGCAAAAGGACCTTTTTGGCCTTCATTCT

>L0347 CGAATTCAAAATCTTCCATGTCAATTCTGTCATTGGAGAGCGAGTGGTTAATGTGAATTCCTTCTCGTCAGAGGTGTCAAGACCTGTAATGGTTTCACGGACCCGGTCTGGATTCTCATTGAACATCATAGACACCCCGGGCCTTGTGGAGGGTGGCTATGTTAGTTACCAAGCTTTGGAATTGATTAAACGGTTTCTTTTGAACAAGACTATAGATGTTCTTTTGTATGTTGACCGCTTGGATGCATATAGAGTGGATGACTTGGATAAGCAGATAATAAGTGCTATCTCTGATAATTTTGGGAAAGAAATATGGCGTAAAAGTTTGCTTGTCCTCACCCATGCACAGCTCTGCCCACCCGATGATCTCAGTTATAATGTTTTTTCAGCTAGAAGATCAGAGGCAGCT

>L0348 ATTGATACTTTTTAATCAGCATATCCATACCAACAACAACAGCATCAAGATAATCGCCAGCAACAGTTCCTCGTGGAAGTTCTTGTAATGCTTCAACAATATCTCCATCAACAACCTTGATATTTCGCAAAACCACAACATGCTCATATCCACCAACTTCTATTGTGAGCTCATTGTCAGTGTCTTCAGCTCCAAATATGACAATTCCAACTTCATCAAATTTTCTGTAAATTAGCTTTTTCTGTATGAGCATGGAACAAATTTTTTGAACCTCAGGAAGAACTTTGTGCATTGATGGACCAACATCGAGTAACAATACCAATCCTTCCCGGTTTCGAGCCATTGCAGAGGCAGAGAGAACAAAGCTTGTGGTAGAAGCTGAAAATGGAGGTGCAGGAAGAGGGCTTTATTTGAGTTTAAAATGGGTCAAGTGCTATACTCCGCTCTTCGATTCTTCCCGCCTCGGGTTACCAAGTCAGCCAAATTACACGCAGCTCTCAGCTGGAAGTCTATTTATTTTTTCTGAATTAGCAAAAAAAAAAAAAAAAA

>L0349 GATTCGAATTCCTATGGGTGGTAATGGCAAGCACAGATGGAAAATCTCCTTCTACCGTCGCTCCAACTCCAACCACAAATCTACTCATCCTCCAAAAGAGTTCCTTTGCCCCATTTCTGGATCCTTAATGTACGACCCTGTTGTTGTCTCCTCCGGTCAGACCTTCGAGCGCGTCTCTGTCCAGGTTTGTCGAGACCTGAATTTCGCTCCTTCCCTCGATGATGGTTCCATTCCGGATTTAACTACTGTAATCCCCAATTTAGCCATCAAATCGACGATTCTCAACTGGTGTGATACTAATGGCGCTGAACGTCCTCTCCCGCCAGATTACAGCTCTGTGGAAAAAGCCGTCCGTAAGAAGATGGTTGAATCGAAAACTTTGAACCCCGAGAATAGGGTCTCCGAGAGCGAGTTGCTGAAGGCTGTGGCAGAAAATCCTCCTGTTCTATTCTCCCACGCGCAGACCGAGTTGACTCGCCGAGTCAATCATTTCTA

>L0350 ATTCCACCAATCCTCGCCTCCAGACCGCTGGACAGAACAGAATCAACTCGCATCGCGTTGTGTTCAAAATCCCGCCAAGTCTCCAAGACTCCAACCTCGCACGCAACGAAATCAGAAACCCGTCGAAAAATTACAATAGTGATCGTTTCAATAGTGATCGTTTCCTTGCAGAGTTGCAGTTTCGATTAAGGAAATGAGAATTCTGAATCCGATCACAACACTGATAAAAACGAAGGCGATGGGTTTCGATTAACGAATTTCTCTTAAATATTGGATTCTTGGGGTTTATTAGGGTTTTGATTTTGTCCATTCCAGAAGAAGAAAGAGATGAATTTGGGGGATTTACATAAAGTATGGGAAATCGAAGCCCTAAAGAAACCAGGAGAAGAAGAAGCTAAGAAGATG

>L0351 ATTGTACCATTCGTCACGAGATCTTTGTTTTGACTAGAGATGGCGTCGAGTTCGAGATTGACAAGATCCCTGGAAGTCAGGGAGGTCATGTTAAAGCTAAGGGAATAATCTACTTGTCAAATGTACGTATGGTTTTTGTTGCAAATAAGACTGTTGGAAACTCCACTGGTGTTGATATGCCCCTGCTTTATGTTCATGGAGAAAAATTCAATCAACCAATATTCTTTTGCAACAATGTTTCAGGTCTTGTGGAACCAGTAGTTCCAGAAAATGAGAATGCAGCTCTCTACTCCACTCACTCATTCAAGATTTTGTTCAAGGAAGGGGGCTGTGGAACTTTCATTCCACTTTTTTTTTTAACTTGATCTCTTCTGTAAGGCAACACAATCAACAATCAATCCTGAGAACGGGCCTCAGATGAATCCTTTAGTAGCAGCACAGACTCCAGTTGATGAAATGATGAGACATGCATATGTTGATCCTAATGATCCAACAAGAATTTTTTTGCAGCAGCCCACTCCAGAGTCCGAGTTAAGGAGGCGCACATACCAGTTGTCTCCAGCTGAACG

>L0352 ATTCGTTTTTTTTTTTTTTTTTTAAGGAAATCTTGCTTATTACGGCTATCCATTTCATATTTCAGTGTCTTCTAAACAACCAAATCAAGAAGAAAAACTAAAGAAATTTATTATTACACGCTTGCAATGGACTTTAAAACCAGAACAAGCAAAACAATGCAAGCAAATACCTCCAAAGTTGTCTTCCTCTCATGAGTCAGTCATGGCCTAGCTAGGTGACTAGAGACCAGAACAAGCAAAACAATGTAAACATTACCTACAAGGGAGCATTGTGTATATATCTCCTCTCATAAAGACCAGTAATTTGGTCAGCCACGGTCCACATTATGGCCTGGCCAGGTGGGATTCTCATCAGCCGGGGCAACAGTCCCTTCCACAAGGCAAGAAGCCCTTCCTCAGCATATATTGTTCTGATAGCATGGATCATGCCTTTGTACTTCAGATTGCCCCCCTCTCGACTCTGAGCCATTAGCCTTGTTTTGACCACATCAAATGGCCCAGTGCACACTGGACCTGCTGTTCCAGCTAGAAATCCTGATATCATG

>L0353 CACATTCATCACTTCCACAGTCCCCCCAAATCCATGGCACATCATCCCCATCTGCACATGTGCTTGGTGGAGGCAACATGCAATCATCTCCTGGCTCATGTTGGTTTGTGTCCCAAATTCATGTGCAATTTGCCACTTTTTTCTGCTTCCAGCTCTTTCCAAGC

>L0354 ATTCATTTCAATTGACAAAAACTCATCCATTTCAGCTGATAAAACCTAGGCTGTGCTTCCTTTCAAAACTTCAGCCTCTAGAATCCTCAAGCAATGGGGCAAGCCTAAGCTTCTCTTTGATTTGCTAGATTTCTCTCCCAAATTGAACAGGGGAGGCAAGAAGGGACAGAAACAAATTAAAGCGGGTAGGACCAATCGAGAAGGTGGAAGTAGTTGTACTGATGTCTCCCTCAAACCGAATGTGGAAATTGTCTGACTGAACAACTGGACAAGAGTGACATCTGTTGGGTTATTCCTAGACATGCAAAGGACCAAACTT

>L0355

ATTCCGCTAGTCAGTCACCCAGCACAAAACAGAGAGGTAAGGTCCAATTCACAAAACCAAAGAAGGGAAAAATGGGTTCTTCACTCTCATTTTCAGTTCAATCTGTATTGATTTCTACTGGTGTTTTGTTCTTAGCTTTGCTTCTCAAAGCCTCTGTTCCATTAGTGGTTGATTTCTCTGTTTATCAAGCTCCTCTTATATGGAGCTCACTCGCTTCATGGCTTAAACCTCCTTATCTTTATGTTATTCTCAACTGCATCATCATCACTATTGCTGCCTCCTCTCGATTCCACCATACCCATGGTGCCGACAATGAACAGCAGGAGCAAATACCTGCTGTTCGGAAAATCTCAGCTCGTGACCCGGATCAGTTCCACTACGATATGAAGATTTCGGCTCCGGAGTCGACGGTGGTGGTGGCGGAGGATGTATGTGAGCAAAGAGAGGAGAAGGCTAGTGCAATTCTTGTTTTTGAGGATAAGAGTACTGTGTTGGTGAACGGTTCTGTGGAGGTTGAGGATAAAGAGGATGAGGATGACTTTGTAATCTCAAGGTCCACGTGGATTCCTCCGAAGAGGATG

>L0356

ATTCGTTTTTTTTTTTTTTTTTTTTTTTAGGATAGCAAACAAGATTATCAGCACTAAAATTTTTGCTGAAGAGAGCAAACTCAGCAGAACCTAAACCAATAAGCGAAAAACTACTGGCATGTTACAAGATCAAGAGGAGGTGAAAAGGGAAAAAAATGATACAATGAGGAAGAGTACATGTATTCCATTGCTTGAAACCAGAAGAGCTACTAACATTGATTTTCACTTGAAGTTTCCAGCGGAGCATTGGAAGAGTAATGCAACTGTTTTAGCAGCTTTGGTTGTGGAAGAATTCTTGAGGTATTGAAGGAGGAGGGCAGTATTTCTTTGCTACGAATTTGTACTTATGTTGGCTGTATTTCTCCCGGATTGCAGGTAAGGTAAAATTATGGCTGTTACAGCACAGGCGGTGGAGATCCTTCGCACATTCACACAAATCTGAAGGCTGGTAAAGAGTGTAAT

>L0357

TTTTTTTTTTTTTTTTTGTTTTTTTTTTGTCAGAAATGACCCAAATGTTCATTTCAACGAACACTAACGGTTTTTCAGAAATACAATTTGCCTCAACAAGGAACAGATATCTATAGCTTCACTAGTATACAAAGATAATATCTAGTACAAATATCAAACTTTCCCCAAACTACAATGTTTAAACAATTATTTTTCCAGATATAGTCAATAAAACTAACAAACAAAGCCAAAGTAGCAATCACCACAAAATGTATGTCTTCCAATTGCACAATCAGTGAGTAGAGGATGGAAAGACCAAAAGAACTATAGGGAAAACAATCTGCATTACTTCAGATCTTTCCAAGAACAGAGTCAAGATCTGGAGACAGGCAGAAAGCTCACGTGTTGATGCCATGAAACGATGGCAAGACAGCCATAAGAGGGTCTCCATAACCAACAGCATCTCCATCATTGAAGAGGAGCTTTAAGACTTCTCCAGCCACATCTGACTTTACAGGAAGTTCAGTACCAAATTGATCCAAATATCCTATC

>L0358

ATTCTTTTTTTTTGTTCTAACAAGATACAATGAGCATCACTATCATCTAAATAAAATTTTCTAGTATCCTTGATCTGTTAGGATCTGGAGGCCGGAAATCCTCCAGATGCAAATTGGAGCTAATTCGAATCTCATTTAAGACACTCTCCCCTTTCTCTGCTTTTATCATCTCCAATGTACTTCCAAGAACATCCCCTGCAAGTCCAACCTCAAGAAGACGGCCTCGCTCCTCTCCACCTTCATGTAATAGTTGCCCAATTTCTGTCAAAGGGACTAAATTTTCTAATACAACTTTGGCAAACATGCGGCCAAGAAATTCTGCTGCTTTTGGGGCATCATTAACAGCATCCTCCAAAGAGGTGAGAACTGACTCAAACCCTTTGATGAGCTGGGGTGGAGTTAATATGCCATCTTGAGACCTTGCAAGATTGACAAGAAGTTTAGCCAAAAGATCTCTCTCCATGTCCTTCCTCTCAAATGAATCCGTCACCCAGAGAGAGATCATTGAAGGATGGAAACTTGACGAATTCAA

>L0359

TGGATCCCCCGGGCTGCAGGTTTTTTTTTTTTTTTTTTGTTTTCAGGCTATCTCACATTCTTTCCTGCATAACACATTATAATGCCATCTTCCACTACAAAAAACAAATTGAAAAACTGGGGAAAAGGTTTATGGAAATTTTGTTCTAGATGTGTTGTCATCATCATCGTCCAAAATAACTTTAACTCCACTATTTTTAAGGACATTAACAATAGCCCATATCTTGGTTCGGTTTTTAAAACCTTTTTTCTCAACCTCTTTGTTTACATTGACATGAATTCCTCTTCCGTGTCCTTGATCAAGACCCTCCACCTTGAGACGCATAGCCAAAACCTCTCCAACAACTGCTGCTGCTTTTGCATTGCAGGACCGACCACACTCTAGTGTGTTCTTGATGGAATGTTCAACTGTCGATGCTGTTACAACAATCCTCCCATTGTTTCTGTCCACAACATTCGCTGTAATGTACTTCAATGATATAAACAACCTCAACACGTACCTTTTCAAAACTGTCATTCCGAGAGAGAACTAGT

>L0360

ATTCTTTTTTTTTTTTTTAACAACAACCTAATAAAAAGATAATAATAATGATAAGAAAAAGGAAATAGTAATTTTGATGCATAAAACATACATTAAAAACAACGAAAACTAACTAGATGGTCACAATTTCATTTCTGTCCAGTCATTCACCACGGTTTCCATCCTTCACATCACCTTCTATGTCAATTTTAGCACATAGTAAATATGTATACACAAGGAGAGAAGTTTCAGCTACAGCCAACAAAATAAACAACAAAAGTTGGTTTTCATTAAGCTACCGGATAAGGTGAGGCTTGATTCAAAGGCATTGTAGCAATCGGTGACTGGGATTCAGTTCGAACTTTCTTGTCGGCT

>L0361

ATTCCCCCTGCGCGCGCGCGTCCTCCAGTTTCTCTGCGCCAAATCCGGCCGATCCGGCCACCGATTGGACCGGGTCTTGTGTCCAAAATCATCTACTCGGCGAGAGCTTTCCATAGACACCAAGAACGCCGAAATCCATCGAGCGGATTGTCCGATTTTTGCTCGGGAAGATTTTCGCCCATTTCGACTTTTGGGCTAGATTTCTCGAAAACCGTGAATCCCACGAGAAAACCGAGGCTACCAGCACGCTCCACTCGACGAGGGCTTCGCGGGATATTAATTTCAAAATTTTCCGACAACGCTTTTCGGTATCCTCGATTCGCGGAAATTCGACAGTTGACCGGGTCTGCAAATTTCGGGCCAGACAGACCCGTGACCGGAAAAGTCTCCGAATTGGGCCGAGGTTTTGGCTAGCCCCCCATTGTCAGACGTCCCGAGTGTGTTCCCGAAGTCGGAATCGGCAAAGGTGAGCCGGGACAGCCTTCTTCCTCCGCCCAGCCGCCACAGTAATTTGTCGTCAAGTCTGCCACAGGAGGATATTTTGTTCTGGGTTAACCTGCTTTTCTACCTCGCAGG

>L0362

ATCCACCACCTCATTGCTGTCGTCGCGATTCCTGTTCTCCAACTCCTCTCCATCGCTTCTTCTCAGAAGATCGGTGTCTTTTAGAGTTTCACGATCATCACTGAATCCCGATTAAATTGAGGTAAAATCACTTAATTAAACCTCAATGAATGGCTGGTTTTGATTTTGCTGTTTGGGTTTGATGAGTTTAAAATTATGGAAATGTAAATATGATGGGATCAATTTGTATGTGCATTCTGGTTGAACCATATTTACATAGAAACTGATAATCCTTCTTAGAAGATCGTTTGATTCCAAATTTAAGGTCAAGACGCTACACTAAAAGAAGGGAACAAAACAAACCAGTTGCCTAGTGGCCTAATTCCTGTGTTCCTGATCTCAATCATGGCAGNAGTAGCACCCACAGTTTCTCAGCTATCGTGCTTCTCTTCAATCAACCGTAATCTGCGACTCCATCTTCGATTCTCTCCTCTCTCTACTCGCTCAAAGGAG

>L0363

TGCAGGAATTCGAATTCGTTTTTTTTTTTTTTTTTTGAAATAAGTTTTCCAATTTCAAAGCAAACTACGCAACAATGATACAAGATGTCATGTCCAAGTTCATCTAAATAACTAACTGCAGCAGACACACATATCAAAGCAATTGAAAATCATCAAGAATACATCAACAACTTCCACCATTGAATCCCACAAAAATCATATTTTCACAAACCTACTTCATTTCCTCTCTAGGAAGATTACTTGTGATTCTCATCCTTCTTAGAATGAATTGCTTCAACAAGGGCCTCAACCTCTTCCTTCACTCGTTCAAATACACCTGGTCCTCTGACTTCACCGGTAGGGGTATTCTCATCAATGTCATCACTTGTTCCATGCGTTTCTTTATGATGGCGTGGAATGTGAATTATTGACTCAATCTCTTCCTTGGCTCTTTCAAATATATTTGGAGATTTCACATCTTTTTCTTCAATATCAAAAACAAAGGAAAAAAAAACTCAGGACTCAGTATNTCTGCTTCCAAAGTTCCATTTGAGGGATTGCTTACAAACTACTAGAGTGGATGTCACATCAACAAAATGGTTTTAAAATATCCTCATTTCTTCAGGGAACATAAAA

>L0364

GGCTGCAGGAATTCGAATTCCAGCCTTTTCACCCTCTAAAAGCTTTAGCTCCTGGCGCCAGCTTTCCACTTTTGCCAACTTCTCCAACTGCTCTGGCTTCATATCCTCTGGTGCAGTTTTCTGCTGCTGACCTTCTGCAATTCGAATCTTCTTTTTAAGCGCACGAATTCTTTTTTCAATATCTTGAGATGGAGCACTTGGATCTGAAGCCTCTGTCAAGTCTGCAGGAGGAGTACTAATGACAGGATTTGCAGAAACAGCCAGCTCATTCATTAGAGATGTCAATGAATTGACAGATTCTGAAGTGTGACCTACATCTTCAGCAGGTTCTTCATTTTCCATATTGCCATCTGCCATTGCTTCCACGTTCTTACCCTTTTCAAGTGCAGCCTGTAACCTCTTCTCCTTCTTTCGTTCATTCCTCTTTACTGATTTCGTCTTAGGCTTTACATCCAAATCTGGGTCATAACCTGGAGGCACTACCTGAGATTGCATCTCCTTTTTCAACAGAGCGCCTTTAGATTGGTAGATGGCGAATTCGTCTTGAGGTACATAACCAGCGCGAATCCGAATGGGTTTTCGT

>L0365

ATATTAGCATTTAATATATATATTTTATTTATTTGCAGTTTAAATGTTTCTGCGGTGCATAATAAATAAAAATAGTTCCAAGAATTAAATCGATGTTTCACATTTGATGCCACTACAATTTACGCTCTATCCCAGCGCGACAGTTTGAACCAAACACAAGCATAAGAGCCGCACCATTATTGCACCCCACTCCACCTAGCCCAGTCCACAAACTGCATTCCACCATAAACGTTGTTGGCAAATCGCACCCCAACTGTGAAAGCCATTGCAATAGGTGGAACCTGCTTGGCTAAGGGCGATGCTTCTACCACGCGTCCCAACCCATTAATAATTTGGTAGCGTGTGTTGGATGAAACTGCAAGAAAAACACCCCAAAGAGCT

>L0366

TGCAGGAATTCGAATTCCGATTCTAAATCCTCCTTCCGACACCGCAATCAACCAGAGATTATTGGACTACTAGCTGTTGAGAGAGACAGAAAGAGGGAGAGAGTTGTGGGGTTCGATGGCCGGCGGCTCTGATGTGGCGGAAGCAACTGCCCTATCGTGTGTTCAATGCGGGAAGCCTGCGCATCTTCAGTATGTGCCCAAAGTGCATGGAATTAAAGCTTCCTCGTGAAGGTGCTGCTTTCTGCACACAGGACTGCTTTAAGGCTTGTTGGAGCTCTCATAAATCAGTTCATGTGAAGGCAAAATTGTCCTCACTTGGGACTGGTTCTACAGGTGAACAAGATTTAGGTGTACTTAATGAAGGTTGGCAATATTGCTTAAGGAGAGGACAATCTCGAACTCCAAAACTTCCTCATTTTGATTGGACAGGGAAACTAAGACCTTATCCTATATCTCCCATGCGAGTAGTTCCATCTCACATTGATCAACCTGACTGGGCAGTTGATGGAATCCCAAAGATTGAACCCAACAGTGAATTGCAGCGTGTTGTGGAGATCACAACTCC

>L0367

GAGCTCACGAATCCAACTCCTTCTCTGCATCAAACTGAACTCCAATCACTTCCTCAATTCGATTTCCTCTTCGCCTACAATGAGTCGCAAGAGAGATAAACCATACTTCTCTCGCCACGACCGTGCTCCCTACCCGAAACGGCGTCGCCCTCTGCCTCCTCCACAGTCCGCCGAAGAGAGTGACGACAAGCCAGCCGTGAAGCCGCCTCCGCCACCTGCTGTGGTGGTTATGGGTCTGCTACCGAATTCCTCTGTTCTTGATCTGAAGTCGAGGTTCGAGATCTACGGTTCAATCTCCCGTATCCGCATCGACCGTGATGGCGTCGGGTACGTCACGTACCGGTCCAAGGACTCTGCCGAAGCTGCCATTGCTGCTGCCCTTGACGCCTCCTTTGGCATCACTATTGATTCGAAAAGAGTGCAGGTGTTATGGGCAACGGATCCTCTTGCGCAGTGGAGGGAAGGAGTNGGGGTTGGAGGTAACAAGGAAAATGGGTCATCCTCCAAACTTTTGCGGCCTGAAAGACCCTTAAGCAGACACGGCAGAGGTTATAAGCTCGCTTCTGCTATAGTAAATCCG

>L0368

ATTCTTTTTTTTTTTTATTACATGAACTGATGAACAACAAAGATCAGATAGGAAGACAGAACTCAAGATTGGAGAAAATCTAAACCCCACCCATAAATATCCGCAGAATCTATTATATCCTCTTTGATGTATATAGCATCTCTTAGTTAAACAACAACAACAAGTAAGTCTTAATCCCAACCTAGTTGGGGTCGACTACATGATCCTTTTTCGAGATTTAGTCTATCATCTCTTAGTTAAAGAAGAAAAAATTTAACTCCAATCTTGAATCTTATACCTGTCAACTTCATTATTCTTATTTTTACAACCTGATTGCCCATGTATACCTGTAGCTGCAAAATTCATGGGTATCAGAATCAGTTTAGTTACTGCTATACATCAACTTCTCCTGCATTTTGCAGCCTACCGGTTCAACTATGGCTCTCCGATTCGTCATCATCTTCCACTTCTATGCTGTTGCCTCCAGTACTAGAGATATCTTTGGCAGATCCAGATCCATGGAAGGGTCCTTTAGGAGAATTAGACTCTGAAATAGTTGATTTCGATGGATC

>L0369

GAGCTCAATTCATCTCTCTCTAAGGTTTAGAGAGAAAATTTTCTCTCGCTTAGGTCTCAGTTCAGGTTCTCTTCGGAAAAGCCGGAGTCGTCTTTGGTGGGTTTCCTTGCCCTTCGGGGTTGAGGGAACCCTTCCCTGGCTAGAGTATAAGCTTGGCTCCCTCCCTAATCTCTAGTTGTGGGTGTAGATATCCTTTTGATTTGGTTGATTTTGCAGATTTTGTGCTTATTTAAGGAGGGACTTTTGTTTCTTTGATCTGTGTTTGCTGTGGTGTGATAGCTTTGGTGCAGGCCTTCGTATTTGGGTGGATTAAAG

>L0370

GTTTTTTTTTTTTTTTTCAATTAAATCCTGAAATTTATAAGTAATTAGATATTGGCCAATAATTCTATTTGGCCATTTATTCTCATTATGAACGTTCATAACTCACGACTGAAAACATAGTCTGAATACATAGGACAAGTACACTGAAAAAGCTTACAATACATATATAATCAAATCCAACATGCATTCTTGAGCGCATCATCATGTTGCTCAGTTGGAAGCAATTGGCGCAGAATGTGGCACCTAAATTCCAAGCAACTTAATAATTTT

>L0371

GCGGTTTTTTTTTTTTTTTTTTTTTTTTTTTTTTTTTTTTTTTAAGGTAAACGCGATGTGCCATTCATAATTAATAACACCATAAGTAACTGAAGATATGCGAGTATAAGAATTC

>L0372

ATTCGTTTTTTTTTTTTTTTTTTATCTGCATCCATTCACTAATTCAAAATAGTGTCCAATACGATAAAATGATACATCCGCTAGAAAGTGGCAACGGTTATTGGTAATTAACAATACGAAACTCGTAAAATTTACAGGAACAAAAATGGAGTTTATAGTTCAATTTACAACAGAAGGGAATAGTGTAGAGTTGGAAGAATACCCAAAAACGCGAACAGCTGGCTAAAATGAAATATATATCTGGTCCCTAGTCGATGATGTTTACTGTGTCCGCATTCTGCTATTTTCTGCACTTCTGACTTGAAAGAAGTATGGATGATTCATTGCTTCTCTAGCTGTAAGTCTGTCCTGATGATCATAACGAAGAAGCTTATCAAGAAAATCAGTAGCCTCTGGAGAAACCAGATGCCGATTATCAGCATTGACAAATCTTGACCAGGGTTTCCTGCCGTGTCTGCCAACAAGAGCTTCAAGATGACGATCTAGCTCTAAGCGATACTTGTTCAAATATGCATTTAACTCATCTGTACCAAGAACCTTAGCAATTTTGACAAGCTGGTCTTGATTGTCACGACCAAAAAAAAATGGCTCCTTCCGAAAAATCATT

>L0373

GGCTGCAGGAATTCGAATTCCTCTTCTCTCTCCCTCTCTCCAAATCTCTCTCTTGTCTTCGTGCGAGTTGTATTTGTAATCCTGGACAGACAACAAACATACACCACTTCCACTTCCACTTCCACTTTCATTCCATGGCCAAGCCAAAGGCACCCAGACGCACTCTAGACTCCTACACACTCAAGCACATCAACAAAACCATTAAAGCTGGTGACTGTGTCTTTATGCGACCTTCCGATCCATCCAAGCCTTCCTACGTCGCCAGGATCGAGCGGATCGAATCCGACAGCCGTGGTGCCAACGTCAGGGTCTACATACGATGGTATTACCGCCCGGAGGAGTCGATCGGCGGCCGCCGGCAGTTTCATGGCTCTAAGGAGGTCTTCCTTTCTGATCATTATGATATACAGAGCGCTGATACCATCGAAGCCAAGTGTGTGGTTCACAGTTTCAAGAGTTACACGAAGCTTGACGCTGTTGGAAACGAGGATTTCTTTTGTCGTTTTGAGTACAATTCTTCTACTGGTGCTTTCAATCCCGATAGAGTCGCCGTGTATTGCAAATGCGAGATGCCTTACAACCCTGATGACTTGATGGTTCAATGTGAAGGCTGCAGTGACTGGT

>L0374

ATTCGTCATTTCCGCCTTCACTTCGACGGAGGAGGCTTCCTGTTCTGTTCTTCTGTCGATCCATATCTTAAAATCTTGGAAGTTACTTTTTAGTTTGATATAGAAAGCATGTCACCTACCGAATCTTCACGTGAGGAAAATGTCTACATGGCCAAGTTGGCTGAACAGGCTGAACGTTATGAGGAAATGGTGGAATTTATGGAAAAGGTTGCAAAGACCGTGGATGTTGAGGAGCTAACTGTGGAGGAAAGGAATCTCCTCTCTGTGGCTTATAAGAATGTCATTGGGGCTAGGAGAGCTTCATGGAGGATAATCTCTTCCATTGAGCAGAAGGAAGAGAGCAGAGGAAATGAGGATCATGTCACAATCATCAAGGAGTACAGGGGTAAAATTGAATCAGAGCTGAGCAAGATTGTGATGGGATATTGAGCCTCCTCGAGGGGGGGCCCGG

>L0375

ATTCTTTTTTTTTTTNTTTGAAAGGAAAGGAGTGGCCATAAAGTTCAGGCGCATAAAATCAAGAAAATAGTTGCAAATTCATACAAGCAGCAAATAGTTTATGTATATATGTATATTGAGAAAAAGAAAAGATAAACATTACTCAAACTTGTAGTGCTTGTCTACACCTACAGAAACATCCCAAGTGCAGCTCATTCCTTTGGGAAACTCAACCAAATCACCAGCACCAATTTCAACAGCCTCAGTTGATCCATCAGGATAAACTTTCACTTTTCCCTCCAGAAGATAGCATATTTCCTTGGCAGAATATGTCCATGGGAATTTGCTTGCAGGGCAACCCCACTTGGGCCATGAGCGGACTCCGAGCTCGGTGAGTTTGGATCGGGAGGATTCCTCANGATCTTGACACCAGATTCTCTATAGTATGGCTCTGTTTTCAGTTGTTGGGCCATCACCCCACCCTTGTTGTTCTTGATCTGGGTCTTGAC

>L0376

CGGCGTAGGATCGAATCTATATTCAAGCGAGACGCTTGAACATGACTTACCTGGTCATTTGCTCCGATATCCTGTTGGGATTGCTAGTGAAGGAGATGTGACAGAGCTTCCAGGAACCGAATTCTTTCCTGACACCAAAG

>L0377

ATTTCGTTTTTTTTTTTTTTTTTCATGAGCGAGTCTACATTGACCGCAAAAAGATTCATAGAAAGAAAACAAAACTCCTTAGCAGCGTATTTCCACCCCATAAATTTTTTCGGGAGATGAGTCCACAATTATAAATCATCCCGGTGGAACAAACCAAAGAGTACGAACCCAAAAAAAAAAAAAAAAAAAAAAAAAAAAAAAAAAAAAAAA

>L0378

GAGATTCTCTACATCTGCCACAGATCGGGATCAGATAGAATCATACATATCAACCTCCATAAAGAATGCATTTACGAGGTCTCTGCAAGCTGTTGAAAAATTAGACACAATGGATGAGCATTCTCTAGCATTACTTGCAGAAGAAACCAAGAAGCTTCTGAAAAAAGAATCAACCATATTCACGCCAATTTTGTCTCAGAGACATCCACAAGCAATCATAATTTCAGCATCGCTTCTTCACAGGCTTTATGGGATCAAATTGAAACCATTTCTCGATGGTGCTGAACATCTGACTGAGGATGTTGTATCAGTGTTTCCGGCTGCTGATAGTCTTGAGCAGTATATAATGTCACTTATCGCATCTGCCTGCGAAGAGGGGAATGTTGAAGTCAATTTCAGGAAATTAACTCCATACCAGGTTGAATCTATATCTGGAACACTGGTGATGCGATGGGTCAATTCACAACTTGGAAGAATTTTAGGTTGGGTTGAGCGCACCATTCAACAAGAGAGATGGAACCCAATATCTCCTCAACAGCGGCATGGGAGTTCAATTGTCGAAGTATATAGGATTGTGGAAG

>L0379

ATTCTTTTTTTTTTTTTTTTTTTGAAGCAATTTGATTAACAAAACCAAGTCATTACTGATTTCCCCAACAATCGAAAATATTTAAAAAAAAAAACTCGGAAAAAAAAATAATATTATACTTTTTTCCTTTCCACCAGCCCAAACAACAATTGCAATATACATCATCGTCACCCCCATTCTCTCCGCTGCACGTGCCCTCTTTCTTTCCTCTTTCTGTCTCTTTCCTTCCTTCCTTCCTTCCTTCCTTCCTCTGCTCTAATAGAATCGTCACGCATACAACTCCCTGGGAGACGGAGAGAGAGGAATCCACAGCAACGCAAGAGAGTAAAAAAGTCAGGCAAAAATAAGTTTAAACCCGAACGCAGGGACGCCGCCGAATTTTAAAACGTAAACGCTTATGCATGACGACGTCGTAGCTGAGGAAGCGAAAACGGAGACACAGAGATCCAAAAGTTCATTGTTAATAAATGCACGCATACAACAGGATACCGAGCAGCGGCCACACGACTCCGTCACCGCCACAATCGCCTCTTCGTTCCCCGAGGTATCGCCACGGGGGCAGCCGTTCCAAGCGCTTCACTCCGCCGTCCCACCAGCCCGGTCGAACCCTCGCTCATCGCCTCGC

>L0380

CAGGAATTCGAATTCCACAATATTGTTGCAACTTCTCAGAAAAGTTGGGCGACTTTTCCCATTCCACTGCGTGAGAAAATTCTTGGTAGTTAGCGGTACCGCAAATTATATCCATACCAATATCATTATCGCGCCTTTCCTTTCCTTCTTTTTNTTTTTTTTAATTTTACTTTAATTGAATTTTTCTTCTTAAAATTAAACGAATTGATCAAAATCGTTTCTTTCCTCTAGATTCTTAGCTCTTTGTTTGGCAACTTAGAAAGTGAATATGGATTTCCAATTTTAAATCAAATAGCAAAATTGAAA

>L0381

CATCACCAGCCTATCGTCACTTTCCCTATCAATGGTATCCTACCAGACCCGGGAATCAGTTGTTGACTCTTTTCCTCTCAAGAATCACAGGAAGGAGGACAAAGTTGAGGGCAGTGATGATGTTGAACAGTGTTACATCTGTCTAGCCGAGTACGAGGAAGGGGAGAAAATAAGAGTCCTTCCCTGCCACCATGAGTATCACATGTCTTGTGTCGACAAATGGCTTAAAGAGATACATGGCGTATGTCCACTATGTCGTGGAGATGTTCGTCAGGGTGCCAATGAGCCTTCTGCCAGCGTGTTTTCTGTTCCTAATCCAGAAATCCCTTATATTTGATAGAATTGTATATATGAACGTAAATAGTAGCCTTTTGTTTCACTTGCCCCCCTTTTTCTCCTCTCTTCCTCTCCTCTTCAGCTCTATTTACGGTTTTTAAATATCT

>L0382

ATTCCAAGATTTCTGGTGGAAAAACAACGTACAGTTGCAACGTTGTAAATGGGAAAAGAAAAATACTTTGTCAATAATGTTGTGTTTGCTAAGAACAGAACAGAACAGAAATTCTCTCCAAGACTAAGCCAGACATGAACAAGGCAGCTTGTCTTCCACGGGGCTTATTTTCTATTTTTGGCATCATCTACGCAGCAATTGAACTCATTTTTACCAGAGCAGTGAAATGGAGGACGAAATTCAGTGATGGAAAAGTAACCAACATAACTCCAGGGTCGGTGCCGGAGGTGAAAGTGACACTGCCTGCTAAGTTGATATTTGAACAAGATGGGTTTGCAAAAAAGTACTGTGATGGATCAGAGCTAGCTCAACTTCCAAAAGTTATGCTTGGTGAAGGTACCATGGGGACTTTGTTCAAGCTTATTCTTAATTGTGGCTTCATCGTCACAG

>L0383

AGCTATTCGAAAGTCGAGATGGGGTCACAACAATATTATCCTTCAAACTTTCAATTTGATACTGGTGCAGATGATCTTAACAATCCCAAGTGGTATATTGTTTGGTCAAGCAATATGAACAGACACATTATCCCCGAGTGTGTTGTGAGCTACAAATCTGCTCATCAAGTGCAAGGCCAAGTGAAAGGACCGACTTGTATGAAATATTCCCTTGAGAAATTGTTTTCGAAGATGAGGAATTTGCTCCCTCCCACCAAAATTCGGGAAGTGGTGACATTATATGATACTTACAGAGGGGGTAAGTTGGCCAGAGATATGTTCATAAAGCAGTTGCGATTGATTGCTGGGGATGAGGTGCTGCTATCCACTATTAGGGAGATTCGTATGCCAGAATGATGTTTAATGGGTTTTTTTTATCCCTCCTTTCATTTGGACGAATGAGTGCTAGGATTTCAATTAGCTTGGGAAGGCTATCATATGGAATAGCAAAATATC

>L0384

ATTCGTCAAGCTGTTAGGGTACTGTAATGATGAAGCGGACAGATTGTTTGTTTGTGAGTTCGTTCCCAACAACTCCTTGAAATCTCATTTGCACGGAAATAACATTGCAGGAAATGGAAACTCCATTGTCATTTGGTCAAACAGAATGAAAATCGCCTTAGGCACTGCAAAAGGATTGGCATATTTGCATGAAGGGTGTAATCCCAGGGTCATCCATCGGGATATCAAAAGTGAGAATATTCTTCTTGATGAAAATTTTGAACCAAAGATCGCAGATTTTGGACTTGCCAAAGACCCTTCAGATTCTACTAGTCACATTTCCACTGAACCAAAGGGAACTTTTGGTTATTTTGCTCCTGAGTGTTTTAACAATAGGCGGCTTACTGATAAGTCTGATGTCTTTTCCTTTGGAATTGTGCTTTTGGAGTTGATTACTGGGAAACCTGCTGTTGATGATGAAGGCAATGATCGCATTAACTTAGCTGATTGGGCAAGGCCTCGGCTCAAACAAGCTTTGTATAGAGGCAAATATAGT

>L0385

GTCTAGAATAGTGGATCCCCGGGCTGGAGGGAAGTTCTGGTGTTGAAAGAAAAATTGGAAAATGGTTCTCGTCGCTTCACTGTCTTTACACTACTTGCTTTGGTGATATTCTTCCTTTCGCAGGCTCGCGCTGTAAACGAGCTCGATTATCAGAGGATCAAAGTCAAACCCTCGGCATCGCCGTTTGAATCAGCTCTTGTACTGCTACAGCAACAAATCAACTATACTTTCCTCAAGATTGATCGTCTCCGCCGTGCAATGACCCACCCCTCATTCTCCGAAGAGAACAACAGGGCATTGAGCATTCTGGGTGTCAACGTCATCGACACGTTTGTCTCTATGCTTGCCCTTGGCAACGACATTGATATCTCCTCCAAGGAACTCAACCGCCTTATATCTGAGATATCCAAAGTGGAAACGTCCTGTGCGGCTGATGGGATGCGGCTGGGATTGCACAAGATTGTTAGAATTTCTGCCAAGACTAATTCAACGACTCCCTCAGTGGTTTGTGGAGCTTTCAGGGCAACATTTGGTGCTATTGCTATTGATTCTGGCAAGTCTGATGTTGCTGGAAGTGCTTTCTGGTATGTNCATACTGGTAAGGCTGAAAAAGCTCTTGCCTTGTAAGTTGAGCTTCCCGGTATCTACTGCTTATAGGGAATGCGTGCTAATGTGCTACTTAGGGCTTT

>L0386

GAGCTCAATTCGTTTTTTTTTTTTTTTTTATCAAGTCACACACTATCTTATTTTTCTAGCATGCATTCAGAACAAGATTAGGTTTCCTTTTAATATTTATTTATTCCATACAATGAACATCTATCAATTTTTCGAAGTTGGACTGGAGAAGGGCTGTCAAGGATCGTGCTTGATTAGAGAAGCTTTCTCAAAAGACAGACGCTAATCTTGCGCGGCTTATTGCAATTCAAACCACCCACGCCGCAGAAGTCATGTCAGATCGCCCTCCTGTCATCCTCTCAGCCTTCTCTCCACGCTCACCAGTGCGAACTCTTATTACATCTGAAACGGGCACCAAGAAAATCTTGCCATCTCCAATCTCTCCAGTCCTTGCCTCCTCAATGATCTTTTCTATCACGGCCTCAACCTGGTCTTTGCTCACCACAATCTCCATCTTAACTTTAGCAACAAACTTGTCTTCAGAAAATTCAGAGCCACCCTGCCTCTCTGTTGAACCACCTTGAGCACCAAAGCCTCGAACATCAGAAACAGTAACACCTCGGATACCAATTTTTAGCAAAGCCGAGGAAACTTGCGAG

>L0387

ATTCGATTTAATAACTAAGCATATTATAAATTTGGCCTCAAATAATTTNAGTTTCAAAGGAAGCCCAGGCTCAATACAGAAAATGCTCCATGGTAAGTAAAGAAAAATAGTCCTAACAGATCAGGCTCATCGCCTGTTACAACAAACTCAACCCAACACCAAGAACCTGACCCAGTTGAAATTCGATCCAGGACTTATCTTCACTGCATGAGCTTGGAACCCTAGCCATCTGCTATAGCAACCATTGCAGCAAACGACCCAACGCTGACCACCAAGCAAGCCTCGTACCTATAGATGAAGCACCAAATCGACACCCAGGCGAGGCCATAGAAGCAAACTCTTTCCAGAATCAACGTCTCCGAGCTCAATCTTAGCTTTACCAGGAATAAAATCTCAAATTCTAGGGGCGGTATGGTAAAGCTCTCTGGTATCATAACCATTAAGCGACAAGAAGGCTAACGAGTAGCAATGGAGGAGTCCCCAGAGGTGCCATAGCCACTAAACTGTAACCCCTAAAACAGAGCCTTTTACTAAACAACAAACAGTGGCGACAGGAAAGCCCACCGGCGTTGCATACACAACTCAATCCATCTCAGTTTCTTAAGGACCCTTTCAAGATACCAT

>L0388

ATTCCGTGTTCCTGGCATCACCGATCTCTATTCAAACTCGATCGAACTGGGAACGGCGGTGTGATTATCGATTCGGGCACGTCCGTGACTCGTTTGACGCGACCCGCTTACATAGCCCTTCGGGACGCTTTCCGCGTCCGAGCTACCAACTTAAAAAGGGCACCCGATTTCTCTCTATTCGACACGTGTTTTGATTTGTCCGGGAAGACGGAGGTGAAAGTTCCAACAGTGGTGTTGCACTTTAGCGGTGCTGACGTGTCTTTGCCGGCGTCTAATTATCTGATTCCTGTGGATAGTAACGGGAGCTTTTGCTTTGCGTTTGCGGGTACAATGAGCGGGTTGTCAATAGTAGGGAATATTCAGCAACAGGGGTTCAGGGTCGTCTACGACTTGGCGGGCTCAAGGATCGGGTTTGCTCCACGTGGGTGCGCCTAAACTCTGAAGGCGTAATCAGTGGGCGTTGCGGTAAGGGACAAAAGGTTTTTTCCCTTTTTTATTTCTCCCTCTAATTAATTTTTTTTTTTCCCGTTTGTGGGTTGTCAGATCGAGTGTAGGATTTGGGATGATAT

>L0389

ATTCCTATTGAGAATTATAGGGCTGATGAAGAATTAGCTCGTAAGCTGCATAGGGTTATGAACAGCTCTCCTAGAATTTCGAAGAACCCTTCCAGTTCTGATTTCAAGGGTCATAAAAATAAGAAGCCTAAGAGCTCACCGACTTCTGAGAGAACTAGGATTTCAAATGGAAGTGTAGCATTTGGAGGAAACCTATCTTCTATGTGCAACGGGCATGGTGTTGCAGGTGAATTGGATTCTGAAGGCTCCATTGGCGAGGTATGCACAAGTACAGCAAATGAGAAGACATCCAAATATGAAAAAGATACTCAATTAGAGATGGATAATGGGGAAGCAGAATCAAGTCACTCAAAGGAGAAAATATGGGGAGATGCAGGTTCGCCCGGTAAAAAGAGGGGAAGACTGAAACTAAAGAAGTTGCCGTTAAGCATTTGTAGCTCTAGGGATCAAGCAAAACCCAAGGATGATATTTTTCCCAGAAGTTGCCCATTGACTGACAAGAACATGGGCAATTCTACTACTCGCAATAAGCCTTTGTTTTCTAAGGAGCCTTCAGGTGATAATCTGATGCCAATTGACGTTGCCCCAATGCGGAAATGC

>L0390

ATTCATTGGATTGAAGACAAACCATGGCAACTTGTGTTGCTGATGGCAATATAATTACTGGGGCTACATATGTTGGCAATCCTGAGTTTATTCGGCTTTTTGTGAAGGCTTTAGGAGGTACCATAACTGGTTCAAATAAAAGGATTCTATTTCTCTGTGGGGATTACATGGAAGATTACGAGGTAACTGTTCCCTTTCAGTCTCTTCAAGCTCTTGGGTGCGATGTTGATGCAGTTTGTCCCAAGAAAAAGGCTGGTGATTTCTGTCCAACTGCTGTCCATGATTTTGAAGGGGACCAGACTTACAGTGAGAAGCCAGGGCATCGTTTCACTTTAACAGCATCCTATGACGAGTTAGATGCCTCAAGTTATGATGCTCTAGTCATTCCTGGAGGCCGAGCACCAGAATATTTGGCACTGGATGANACAGTGCTTGCCTTGGTGAAAAAATTCATGGAATCTANGAAGCCAGTGGCATCCATTTGCCATGGACAGCAAATTTTANCTGCTGCTGATGTTCTGAAGGGAAAAAAATGCACTGGCTATCCTGCTGTGA

>L0391

ATTCCAAGCAACGAGAGAGCGGAGCACTCCATAAAATCACTTCCCTCCTGCTCCAAAACACTCTAGAGTCTCCAGGGTTTCTCTGCGTACAACTACCAAGAAAAGGGGTAAAGGAACGGGAAGTTTTGGGAAAAGAAGGAACAAAACGCATAAGCTCTGCGTGAGGTGCCGGCCCGCTGCTTTCATCTGCACAAGAGCCGTTACGGTTCTTGCTGATAACTGGAGCGTGAAGGCAATCCGGAGGAAGACTACGGGAACGGGAAGGATGAGGTATCTGAAATACGTTGCTGTTAGATTCAAGAGCAACTTCAGAGAAGTATTCAGGCAACCCAAGGAGGAAGGGTACTTCACCATCCTCGTAAAATCAGTTGTAGACTTGTAGAATCTGTTTCATATTGATCAAACAGTAGCAGAATTAGGAACCACCAAATGCGTAGCTTGGTTTTCATGCTTGATGAATGATGATGCTTATTATAGCTTTACCATCATCAACTATAAGTCCTGAAAAAGGGCCAATGTGATTCCCAATAAAAGAAATTTA

>L0392

ATTCGCAGCACCGGACGGAGTCTCCGCTCCTCAACCTCGCCTCTGCTCATCCTTTCCGCTTCACAGTGAGTGGATCTCAAGCTGGCCTAATCATCCTTTCCTCGCAGTGGCCCCCATCTCACTGCTGTCGTCGTCATCGTCGCCAATGACCACTTTGAAGTCATTGCCACTGCTGCTTAGATTGAACTGTAGGAAGTTGCTGATTTAAGGAAGTCATTACAATAGAGATTCAGTAATTCCCCCAATTTGAATCCCCAAAAATGGCCACTGCCGCCATTCGCTCTGCTGTCTGTGCAGGGCGCCACTTCTTCTCTTCTATTGCCAGCAGCAGCCATAACAATACCCATAAAAACACCCATAAATTCTTGGATTCTAACTCCTTCATCGGTAGCTGGAAAGCGCCAAAGAACCCAAAAGAGGCGGAGGCAAAACTAGCACGATTGAGGAGGGTGTATGCGAAGCAAGTGAAGGAGGTGCGGAAGGAGTACATAAGGGAAATGGAATTGATCCGCATTGAGAAGCAGAGGCAGGACGAAGCTAGAAAAGAGGCCAT

>L0393

AAGAAAAAAAAAAAAAATTGTCCAAATCCCAAAGATGAGAACTTTTCTTTCACATGGGGGGATTGAGGGTATTTGATACTGATATGGTAGTGTATGTCCAGCCGTCGCACGGTTCAATGCTTTAGATAAACATGCAAGAATTCTCTCTGGAGTTCACCCATATTTTGGTGTGAGATTAAAAGCAAATCTGTTACTTTTCTCTCCAAAGCCAAATAACCTTTTAGAAGGGAAGGTGGTGAAACTTATGCAAGAATGGATTCATGTTATTGTTCTTGGTTTTTCACCTGCTGTTGTAGTAGATGAAGATATTCGTAGTGAATTTAGATATAAATTTAAGAGTGGTAAGGGTGCATATGTTAGCAGAGCTCGCAAGCGACATGTTATAAAGGTTGGAACTGTGGTACGCTTTTTAGTCTGGAGGTTGGATGAGGAAATACTCCGCATTTCCGATCCTTGATTCCAGTTTACACTGGAAGCGTTGGTTGGATGGAGATGCCAAAAATGCAACAACTGAAAGAAGAGAACACAGCCTGATGGAGAAATTGA

>L0394

ATTCTTTTTTTTTTTTTTTGTGAATTCAAAGCTACTCATTTCCAATTGATACTTTTTTACTCCTATGAGGCAGGTGAACTGAAGTTCATTATCAGAACAGAATACAGGCATACTCTTGTAAGATGAACTAATACTAATACAACACAGCACAATGAAGCCTGAGCTAGAAGAACTCGAAGTCCAGATATTTACCCCCAGACTTCATGTCTGTCCCCACCATTCTTTTACTGCCGCTGCCCACCCCAATTGGATCAACAGCCCCAAATTTCCCAGGCAGCTCAGTCTCCATGGGTCTTGGCACTTCTGGAGGTGTGCTGCACCTTATTAGAGCCCAATTTACACCTTCAAAGAAAGGGTGCTGCTTGATCTCAGTTGCACCCCTCTTCACTCCCAACCTGTGCTGTGGTTCTTTCACCAGCAACCCTCGAATCAAATCTCGGCTAGCATAGCTGGTTGCTGGAGAGTCTGGGAATCTC

>L0395

GCGATTCCTGAAAAATAAAAAAAAAAAAAAAAAACGAAAAAATGTAAAGAAAAAGAAGGGAGAAGAGTTTGATTCTGAAAGGGGAGGACAAAACAAACCAACTCTAGTTTTTCAACTGAAGAATTCGGGGGAGAAGGCGAGTTGTATCTCTTGAATAGCGAAGCTTGGAAAAATGGCAGTGAGCTTGGCAGGAGAGAAGGTAATTCTTGTACCATACATGGCGGCTCACGTCCCCAAGTACCACCACTGGATGCAAGACCCGGCTCTGCTCCAAGCCACTGGGTCGGAGCCCCTCTCTCTCGAAGAGGAATACCAGATGCAGCAGTCCTGGACCCAGGACCCCCTCAAGCGAACTTTTATTGTACTGGATAAGGACTTGATAGAGGGAGATTTTGTTCATGGAGATTCCCATGAGGAAGCCATGGCAGGTGATGTAAATATATATATGAATGACTTGGATGACCCACAACTGGCTGAGATTGAGATAATGATAGCTGAACCCAAAAGCATAGCCGTGGTAAAGGCCTTGGGAAGGAATCAGTGCTAATGATGATGGCTTATGCTATCCAGAACCTTGGGATCCACATT

>L0396

ATATTCTCCTCAGTCTGTTTACAGAGATCACGTGAACAACATTGATAAAGCTCTTGATGGCTTCAGAAGCAACTGTATCTGGCACACGGTCAAGAGCATGCTTCCACTCATCACAGAATGCATATGCATCGGAGTGTTCCACATTACCATTTGCATTGTCATTGCTCACAGGAACAAGGGTGAGCTTGAACCAACCATGGACAGATTGTATAAAGTCCCGCTGGAACTTTATTAGGTGGCAGAAACTGGAGTGCCAGGCAGAGACAGCTGACTCAAGATCACGAGTTGCCTGTCTGTGCAGTTCAGATGTTGAGTCACCATTGGCTGATCGGTTCACAAGGCCACGGACTTGTTGAACAATGTTGTTCTGAACTTCATGGTACTTGGTGCATTGATTTCCACATGTACATGAATCCATGACACAGTTCAACCAGCTGAGGGACAAGATCAGTGTCCCTAAGACCAATGATGGCCGTAGAAGTGGTGGAAACAGCCTGGGATGTGACAAAAAT

>L0397

ATTTCCCAGACCTGAACTGCGTGACTGGACGAATTTCTGGCGCTTGTTTTGATGCAGCTCGTGGTATTATTCCTGGCTTGAAGGTTAAAGGAACAGATTACAGGACACATGATGGCACTTGCATTCGCGATTATATTGATGTTACTGATCTTGTTGATGCCCATGTGAAAGCCCTTGAAAAGGCAATGCCTGGAAAAGTTGGGATCTACAATGTTGGCACTGGAAAAGGTAGATCCGTCAAGGAATTTGTGGAGGCATGTAAGAAAGCAACTAGTGTGAACATCAAAGTTGACTATTTACCTCGCCGACCTGGTGACTATGCTGAAGTTTTCAGTGACCCAACAAAGATTAGGCTTGAGCTGAACTGGACAGCACAACATACTGATCTCCAGCACAGCTTACAGACTGCATGGAGATGGCAAAAGGCACATAGAAATGGATATGGATCCCCTTTGGTGATGGCTTCTTGAAACATTGAGAGCAGTCCATAGCAAAAATTTAACGTAAACCCACCAATGCAGCACCTGCTTG

>L0398

ATTCCGTGCATTTGCCATATTTTGGATACTGATCAGTACAGTTGGTTTAGGTCAGTTTTTCTTCAACGTTGCTGAGATGTTCACTGAAAGCAGACAAAGGGCACTGGTCAATTGGGTCCTTACTAGAAAGATGACCAACCTTGATCTGGAGGCAGCTGATATTGATAATGATGGAGTTGTTGGGGCTGCTGAGTTTGTCATTTATAAGCTTAAAGAGATGGGTAAGATTACAGAAGAAGATATCTCTCTTGTAATGGAAGAGTTTGAGGATCTTGATGTTGATCAATCCGGAACCTTATCAGCATCTGATTTAGTGCTTGCTCAAACAACACAGACAAAGAGGTGATCTGAAGCATCATAATGGGGTCATCTACTTGTAAACTATATAAAATTGTTATCGTGTGAGGATTACTTCCTGAGTTTTCAATATCTATTTCAGGTTGTACGCTATATTTTCGATTTATCTTAAGATTTGCCTGAGTTGAAGTGCTCTCAAA

>L0399

ATTCTTTTTTTTTTTTTTTTGGAATAGAAAATACTCTCCAGCATAAGATTGCTGAAGATGTAGAATCAGCTGATATTAAAAGTTTCAGAGTCACCAAATCATAAAAACAGAAATAAATAATAACCTAGCAAAAACCTAGCTACTATGATATAAACCCGACCAAGAAAGCCACAATTTATACCTTCACAAAGCCTAATCCTACTCAGTTTTATATGCCGNCAACATCAGCTTTGGCTGCTCCCTTATCAACTCATTCCTCTTCAGAAACAGCTCCAACTCAGCAACATGCAACTCCCTCCATTTCTCTTCCATCTCAGCCTTTTTAGCACCCTCCAACATATCCAGTCCCCTCTTAACCACATACTCCTCCATTCCTCCTACACTCACGCTCTTATCAAACTGGACAATCTGGTTCAGACCAGAATGGCATCCAACTCAACCTCCATCTTATCAACCTTCTCAGTCTCCTTCTCCACATCCACACCTAGTTCTGCCTTCAATGTAGCCAAACTCTTTCCTCTCAGACTATTAATGGCACTTTTCTT

>L0400

ATTCCTGCTCTTTCTTCAACCTTATCTGGAGGTGTTCTGCAATGTCCTTCTCATCGACATTACAGATTATCTTTTCCTTGTCACTTTCANCGTATATATACAAGCATGTAGGCATTTGAATATTTTGTAAACTTAAAGGGAGAGTTGTTGAAGCCGGGATTTGCTTATGGTAACTCTTCCTCACCACCATACTGCTCCTCTAGCGCCCTTTTCATATCTTCCTTTGTTACCCGCTCATCATCAAACTTAAAA

>L0401

ATTCATTTGAATCTACATTTTTGCTTCATACGCTATGACTAAGCAACAAGCTAACTGGTCTCCATATGATAACAATGGAGGATCTTGTGTTGCGATTGCCGGCGCAGATTATTGCGTCATCGCCGCCGACACTCGAATGTCCACTGGCTACAGTATTCTCACCCGGGAGTACTCCAAAATCTGTAAACTAGCTGACAAAAGTGTAATGGCGTCTTCTGGTTTTCAAGCTGATGTCAAAGCCCTACAAAAGCATTTGGCAGCCAGGCACTTGATCTATCAACACCAACACAACAAGCAAATGAGCTGTCCTGCCATGGCTCAACTACTCTCGAACACTCTCTACTATAAACGCTTCTTTCCGTACTATGCTTTTAATGTTCTAGGTGGTCTTGATAGTGAAGGAAAGGGCTGTGTCTACACTTATGATGCTGTTGGTTCCTATGAGAGGGTGGATATAGTGCTCAAGGTTCTGGTTCCACTCTTATCATGCCTT

>L0402

GAGCTCACGCGGTGGCGGCGCTCTAGAACAGTGGATCCCCGGGCTGAGGAATCGAATCGAACGAGAGTATCTATTTCAAAACAGCTTTTTCACTTCCTTACCCATGTCGAATATAGTTCTTTTCGAGGATATTTTTGTGGTTGATAAACTAGATCCAGATGGCAAAAAGTTTGATAAAGTTTCGCGTATTGAAGCACACAGCCAGAACTGTGACATGTTCATGCACTTAGATGTGAATACAGAAATATACCCAATGGCTGTAGGTGATAAATTCACCATGGCGTTGGCACACACTCTAAATTTGGATGGAACACCCGACACTGGCTATTATATTCAGGGAGCAAGGAAAACCCTTGCAGACAAATATGAATACATAATGCATGGGAAATTATACAAGATTTCTGAGGAAGGTTCAGGAAAAGCAGTTAAAGCGGAGATATATGTTTCATATGGTGGGCTTCTAATGATGCTGAAAGGAGATCCTTCTCATGTCTCTCATTTTGAGCTTGATCAGCGGCTACTTCTTCTTATAAGGAAGTTGTGAAGTCCTTATCATTCTAGCTTGGCTCTCTAGTGGTTG

>L0403

TTTTTTTTTCCCATAAGATAGATTTCATTCATTAAAAGAAGACTTCTAAGCCCAGTATCGAAGAAAATCTATGGCCGAAGCTTGAACTTAACCAACCCCCAACGTACAAGTAAGCCCAACCCAAAAGAAAACCCTAACTCTGTATCTCCATATCCGAACCCATACCCGGAAGAGAAACCAACACCTCCTACAGGACGAGGCTCAAGCGCCACATACGGACAAACCACAACGCCGGCGGGGAGAAAGATCCCCTCATGAAGCACCAATGGGGATGAGATATGGCTACATCGCCTGAAAAAAGAAACCTCACAAACTACACCTCAAGGAATTGCCATCAAGCTAGGACACAATCAAAACCATGACGCACTGGGTTGAGCACCAAAACCAGAGGAGTTGTAGACCCTTCTGTTGGGAAGAAACAAGCACCAAGCTTGGTGCACCGCAAAGGACTAGACTTGGCCAAATCTCCTGAAGGATGT

>L0404

ATTCCACTTCCGCATGCCGCCATACTACACACTTGTTCTACGATCTCTTGCATCCCTGGAAGGTCTGGCGATGGCTGCAGATCCAAACTTTAAGACATTTGAAGCTGCATACCCATATGTTGTCAGAAAACTACTCACTGAAAATTCAGCTGAAACAAGGAAAATTTTACATTCGGTGGTTTTAAATAGAAGGAAAGAATTCCGGTGGGACAGGCTTGCCCTTTTATTGAAAGTAGGATCAACTAGTAAAGTTTTGAATAGAGAAGCAGCATCAAAGCATGAAAATTCCCTTGATTATTTAACAAATAGGTCATTCTCTGGTGTTTTTGATGTTGCACACTTGGTCTTGATGCTTTTACCGGCCAGAGATGGTGTTGTTCTTAGGAAGCTTCTAATGACTGCAGATGGAGCTTCGTTAGTCCGAGCATTGGTTTCCAAGGAGGCAGTTTTCTTTAGACAGCAACTTTGCAGGACCATTGCTGACCTATTATATCAATGGACAGTTCAAACTCTTGGA

>L0405

GATTCGCTCAATACCCTTCAAATATCCTCCTTCTAAACAGAAACGGGCAAGAGGGTCATGATTACTCATTGCAACCCCAACCAGGAGGATTTCTTGATCAATCCCATATGCTATTCACCAATGGAGGGACTAATAATCCACGAAAGAGAGGAAGAGAAGTGGCATCAGCAGCGGCGGCGGCGGCAGCAATTACAACGACCACTGGTGGCATCACAACAGGGCCAACAATCCATCCATTCTCTATGCAATCTCAAGCTCCTCAGCTAATAGACCTCTCCCATCTCCACAACCAGCAGCACCTATCCCAACCAAATGTTGTCTCCACGGGCCTCCGTTTATCTTTCGGCGACCAACAACAAATGCAACAAAATCACCATTATCAGCAGCAGCAGCAGCAGCAACAACAGCAACAGAGTTTTGTATCCCAATCATCTCCTTTCTTGTGCTTATTATCAGAAGATTTTGCCACCCAAATTAAACGCCAAAGAGATGAGATGA

>L0406

GGAATTCGATTCCTGTCCTTCAACAGAAAAAAATCCATTTCTTTCCTCTTTTCTCTCTTGTAATAACCTTCAAAAAAGAACCAATTTGCAATATTTTTCTGTTTTCTTTCACTCTTTGAGAGAGCTAGTAATCTTTGATCAGAATACTAACACTCTGCTCTCCAATTTGATCTAAATTTCAAAGTTTTTTGCCTAAGAAGCCTTGAATTTCTATTCAATTATTGATGGATGCAGCTGGGTTTTGAGCAATCACGGGCCTTTATCATATGTAATCTTCGTTTCTTAATTGTTATATCACGATCTGTAACGATCCTAATGTACAGATGACAAAGCTGTTAAAGCATGTGTTTTGTGTTCTTTTCTCTTTGTCTTGCGATTCTCTTGTGCTTCTTGTGGGTGGCATGGTCAAGTGTACTCAGTCTATTATCTGATGATG

>L0407

ATTCGTACAAAAACAGAAGGAACAAATAAAATCACTCAAAATAGCAGTAAATCAACAAGTATTGGACATGAAGTTTTGGATGACAACAGTTTCAACTAGTGTTGCAGTGAATGGTAAGAAAAATGAAATAAGTTGAACTTCCAAAAACTCAGTGCTCTGATAGCCAACAAAAGTTTTCCTGAGAATGT

>L0408

ATTCAATCGAAGGTGTTATTATAAGATGAAAGGAGATTATCATAGGTACTTGGCGGAGTTTAAAGTTGGGGATGAGAGGAAAGCTGCTGCTGAGGATACTATGCTTGCTTACAAGGCTGCTCAGGATATAGCTCTTGCTGATCTTGCACCAACGCACCCTATAAGGTTGGGGTTGGCACTCAACTTCTCAGTGTTTTACTATGAGATTCTCAATTCATCGGAGAAAGCTTGTAGCATGGCGAAACAGGCCTTTGAGGAAGCCATTGCGGAGCTGGACACATTGGGTGAGGAGTCCTACAAGGATAGTACTCTCATCATGCAACTGCTGAGGGATAACCTTACTCTCTGGACTTCAGATATGCAGGAGCAGATAGATGAGGCATAATTGGATGGGATTGTTTTGGCTCTTCCAAGGGGGTGGGCTAAAATGTCATGAACTTTGGTCTAAGTACAACAAAGCTGTGTAAAATATGCTCTGGTTTTAGCTTCCTGAC

>L0409

ATTCCTCATCTTCAAGAATTGGAAGCTCTCACCAAAGCTCTCTCAGGACTTGGAATTGATGAGCAGTCATTAATATCAATTCTGGGAAAATCAGATCCTGCGCGTAGGACAACATTCAGACAGAGAAGTACTCACTTCTTCATTGAAGATGAACGTTCTTTTGAACGCTGGGATGATCACCGTATCAATCTCCTCAGGCTTGAGTTTGTTCGATTTGAGAATGCTCTGGTGCTCTGGGCCATGCATCCTTGGGAAAGAGATGCTCGTTTGGTGCATGAGGCCTTGACTCTTGTTCCACAATCTTATCGTGTGATTTTGGAGATTGCATGCACCAGATCATCAGAAGAGCTATTGGGAGCAAGGAGAGCCTACCATTCCCTCTATGACCACTCCATTGAAGAAGATGTTGCCATCCATGTCACTGGCAGTGAGCGTAAGCTCCTGGTAGCGCTAGTGAGTGCCTATAGGTAT

>L0410

ATTCCACAAGAACGATATCCAAATCTATTGCTCGTCATAAATGATCTTTCCAGGTTCCAGCTACATATCCGTGACAAATAGGATGTGAAGTCACAAACATGTTCATCAACAGCTACCTCTCTGTGATCACTAGTGATCACAAACATGATCACTACTATTACTGCTACTGCTACAACTACAGAAAATATAACATATTGTACCCAATAATATAATATAATAAAATATAAATCCGTGTTCATTACCTAACAATCTAAAGAGTTCCTCACAACCCAACAAGAGATATCGCACACATGATGCAACAAGATAAGAGAACTAGCTCATAGTCATGCGTATGGCAAATAAAATTATGAAAGAGTAGTTTCTGATGAGTTTTATCCTCATTCTTGTTCTTTAATTGAAATGGGTGGTAACTTTAATACTTACTCATGATCATCACTTGTATTAGTGGTCGTGGTGCGGTGTGGTG

>L0411

CGATTCGTCTCCTCGTCCTTGTCCCTCACCATCGGCGATGCCGTGATCTCCTCGTCCGATTAATTTTTTTTTTTAATTTTCAGCTGCCTAATTGTCCATCGCAATACCAGATCTCCTCTCAGTACCAGATCGCCAGACGAAGAGCAACCTGCCCACTGAACCCTTTTTTTATCCGGGTTTGTTGATTGGTTTTTAATTTCTGAGAAAATTTGTTGAAGATGTACACTGCTGTCTGTACATTCTTTTTTGGTCATTAATTGGGTACGAATGCTGCAGGGTGAATATTGATTCAGAGAAGATTTGTTGAGGTTTCTTTCAGATCTGAGAACACCTCTCAGAAACGGTTTGATAACTCAAAAACCCTAACCGCAGAATTTTTGGTTGCTGGTATTGTTTTCTTCAATATTTGAGATGGAGAAGTTGATTTATATTCACGTTTTGAGGTTGGGAGAATCTTTGTATGAATCAGATGATATAGATAGTGGCTATAGGGACGCAATAAACGATTGGTATTTGTTGAAACAAACCGATGGTAATAATGGTGTTGAAATCAATT

>L0412

ATTCGCCATTGATTACAGTACGTACGTTTCATTAACGCATCCGGTACTATATACCTTTGTCTGTTTTATGCGGACAAGCTGCAGAAAATTTCACGGCAATGGATTTGCTAGATGTCATCGTAATAACCTTCAGTTACCTAGGTTTCGTTTCTCTCTGCAAAAATATTGTGAGATTTTGCAGGTGGGCATGGGTCATGTTCTTGAGGCCACCAAAGAATTTGAAGGAATCCTATGGTTCATGGGCAGTAGTCACTGGTTCCACTGATGGAATTGGCAAGGCTCTTGCATTCGAGCTCGCATCAAAGGGTCTGAATCTAGTTCTGGTGGGTCGAAACCCTTCGAAACTTGAAGCTACAGCTAAAGAAATACGAGCAAGAAATGGCAAGAATAATGATCAACTGCAGATAAAAACAAGCGTCATAGATCTTGCAAGGTCTAATGGAGAGAAGATATCAAGGGCAATAGAGGAGTGTATTGAAGGGTTAGATATTGGTGTTGTGATAAATAATGCAGGATTGGCCTACCCTTATTCCAGGTTCTTTCATGAGGTTGATTTGGAGCTGATGGAAAGCA

>L0413

ATTCCTCTTTTATTGAACTCTACTCCGTTCCACCTTTCAAGTCTCATTTTTATTTAGTTTAACAGTTTGTTGAGTACATATCTCACCGGGTCTAATGGCTGCTAATACTGATCTACCCCCAGGCGTGGACAAAGAGCAGGTTTTTGGCATGCTAGCTATGGAGATGGAGTATAAGGTAGAGCTGTTTAACAGGCTTGCTATGGCATGTTTCAACAAGTGTGTTGATAAGAGGTACAAGGAACCTGAGCTAACTATGGGTGAAAATAGTTGCATTGATCGCTGTGTTTCAAAATATTTTCTGGTGAATGGTATAATTGGCCAGATGCTCAGCGCAGGTCAGCGTCCAATGTGAAATTCGTATTTGTTGTAATTTGGAAATTT

>L0414

ATTTGTCTTCATTTGGTTACTGATGTTGAATGGTTAATATAAATATACAAGCTTTGTTATTTTTTTCTCATTTTTTTGGGTAATTTAGTTCCTTATCTGATCTCTTTCCCTTGCATGTAACCTACTAATATCATTGTCGATTTTTTTTCTCTTATGTTTGATAATTAAAAGATATTTTGTTACTGAAAATTAATTGCGTGTATCTCAGATCTTTTGTGTTTTTCTTTTAGTTTTGGATTCTTGAATTCTGCAGATTTCCCTTCAAAGTTCTCTTGTAGGATTAACCCTTTCTGATGATATGCATAAATGAGATTCAAGAGGTCTGTGTCATGCTTGGATTAACTCTGGATTTCCCCTACATTTGTGTTATTTGATTCTACAGGCAAAATATATGGAC

>L0415

TTTTTTTTTTTTTTTTTTTTTTTTTTTACATCTGAACCAAAACTTTGTACCATATTTAAAAGCTGTCTCTTANTCAAACAGTTCACAAACAACAAACAAAAATTATAATGTCTTCAATAAATTTCAATATTCACTTCCTAAAGATACTAATCATCCCTGNAACAGTAACCAGTTCATACTCAACCAAAGAAAGATTGTTATTTTCTTGAACTACAAAATTTGCAGGTTCAGTAACTTCAATGCGTCCCCACTTGTCCACTGCAAGCCTCATAGACCCCTTGAACATGTCAATCTTTGCATTTCGCAGAATAACAGTGGTCCCAG

>L0416

ATTCCCCAACTTGCTTCATGGCTTATTAGGGCTAATTGGGTTTGCATGCATAAGGCACATCCACATGTCGCCTGCCTTTTGGTGATCCAGATGCTCTGCTGCTAGCTTCCATCACCTCTTGTCTTAGGCGCGCCTTTCATAACTCTGAAATGAAATTCAAGAAGGTGATCTAATTAGAGGTTCAGATGATCTGGAGAAACTAGAAGAGTGAACTGCTGCTTTTGGATTGGTGGATCCTGCTTTGCTGTTGCAATGCTGTTTTTCGTAATCCTCATTTTGGTGTCTGATCTCCTTCAGCATTGCTGTTACATCTTTCATTGTGGGTCGTTCCCCTGGGGAAGGGTTCACACAGAGTAGCGCCACCCCAAGCACCTGAAGCATTTCTTGAAGCTGAGTGCCTGATCGAAGAAGTAGCTGCTGGTCTAGAATTGTTGTGAATTCTCTTTTTCTTTCCCGGAGTTCTTTGTTGACCCAAGCTAAAATGTGGGCACCTTCAGGAATCCTGTTGTCAGTTGGTT

>L0417

ATTCCAATGCACGAGTGTTCTCAGAAGAGGGAAACAAATAATAGCAATCGCAGAGAAGAATTGAAATCTAGGGATTCGAGGAGTTGTCTGAGAGATGTCGTATCTGCTTCCACACTTGCACTCAGGATGGGCTGTTGATCAGGCAATCTTGGCGGAGGAAGAGCGCCTCGTCGTCATCCGATTCGGCCACGACTGGGACGAGACTTGCATGCAGATGGATGAAGTGCTCGCATCAGTTGCTGAGACAATTAAGAACTTTGCTGTCATATATCTTGTGGACATCACTGAGGTGCCTGATTTCAACACAATGTATGAGTTGTATGATCCATCCACTGTCATGTTCTTCTTCAGAAACAAGCACATTATGATTGATCTTGGAACTGGAAATAACAACAAAATTAATTGGGCTCTCAAGGACAAGCAAGAGTTCATTGACATTATTGAGACTGTCTACCGTGGGGCAAGGAAGGGTCGTGGTTTGGTTATTGCTCCAAAAGAT

>L0418

GGCAAATAGAAAAATTTAGCAAAATTATAATACTATATTCTTAATTAAAAAAAATTAAAATTATATATCAATAGCAAAAGCCTCTCTCTCTCTCTCTCTTTTTGTTCTCGAAGAAGCTAAGGCAGAGCCAACCTCTGTTCCTCCACTCATGTATATAACATAACACAAACCTCCACGGAGCTTCCATCAAACCCAAGAGGGGGAGAGAGAGGGAGAGGGAGAGTGCACAGAGAAGGAAAGAATCTCTGATGGCTAAGAAGGTTTGGTTCTTGAAGAAGGTGGGGATCAACAAGGATCATCTATGGCCATGGAAAATCTCACTCTCTTCTTTCAAGTGGAAACATGTTGATTTTCAGTTGAAAATTATTGATAATTTGGTGTTCAAGATTCTTTATGTTGTTGAAGCTATTGTTCTTGTTTCTACTCTCTGCTTCTTCTACCTCTGCTGTGGCTGTCACTTTTGATCCCTCT

>L0419

ATTCCAACAGCTTATAGATGCAGTTGCTCATTGTCATAGTAAGGGTGTTACCATAGAGACCTGAAGCCTGAAAACCTACTTCTTGATTCTGTTGGAAATTTGAAGGTTTCTGACTTTGGATTGAGTGCATTGCCCCAGAAAGGGGTTGGACTTCTTCATACCACATGTGGAACCCCAAATTATGTTGCCCCTGAGGTGCTTAGCCACCAGGGTTATGATGGCGCTGCTGCTGATGTATGGTCATGTGGAGTCATTCTCTATGTTCTGATGGCTGGATATCTTCCATTTGATGAGGCAGACCTACCGACCTTGTACAAGAAGATAAATGCTGCAGAATATACTTGTCCATTTTGGTTTTCTCCGGGGGCAAAGGCATTGATAGACAAGATACTCGATCCCAATCCTAAAACTCGTATACGGATTGAAGGAATTAAAAAGAATCCATGGTTTGAAAAAAAATATGTGCCTGTCAAATATAGTGAAGAAAGGGAAGTGAATCTGGATGATGTTCGTGCAGTTT

>L0420

ATTCCTCCTCGAAGAATTATAAGCTCTTCCTCTCTCAGCCTCCTCAATCCATCTGGAGTCTCTGATGCTTCCCGTGGATGATGAATACTTTTATTCGATTTCAGTGGTCCGGATCTAAAATATCATGCTTTTGGGCAAGTAGAACCGATAAGGAGCTTCAGTGAAAGGTTTTATTTTATGGAGCTGACGGGCACAAGAAAAGTTGTTTCTACCCATTAAGTGGATTGGATAAAGAATTACAAACTCTTAGTCTTATGCATGACATTTCAGAACCCAACCTTAAGAGCGTTGTCTCTTTTTCATAAAAGGTGCTAGAGATGTTGCTGTTCTAGATGCAACGGTTGAATCTGGAATGACAGGTAAAGAAGTTATAGGGAAGAGGCAAGAAAACAACAGAAATATGGTTGACGCTGTTAATTTGAAATAATCCATTGGTTCTGAAACCAAATGTAATTGACCTAAGTTGTAATAGATCGTGGAGATTGATCTTGTAAAGGATACTCTGATGAGGTTCAGAGATTTATGGAAGAGTATTTGAATCTCTAACAAAAAAAGAGAGAGAGAACTAG

>L0421

GGCTGCAGGAATTCGAATTCAGAGAGAGAGCAGCTGAAGTGAAACCAGGGAAAGTTGCCTACTTGGCACCCTATCGTAACACTGAAATCCTCTACGTCTACTCCTCCCTCACTGGTCTCCGCCGCACTCTATCACCTCCCCCGGACTCTCCCGACCAGAGAGCAATTGTGGAAGTAATTGGACACAAGGCTCAGGGTGCTGTTCCAGAGCCTGGTTATGTTGTCATTGCCCGAGTTACTAAAGTGATGGCTAAAATGGCTTCAGCTGACATTATGTGTCTTGGTCCCAAGTCTGTGTGAGAAAAGTTTACTGGCATAATAAGCTGTCCCTTGGAGATGCACGGGCTTATCACCTTTCTACTGCTAAGAATGAGTTAGGTGTTGTATCTGCCGAGAGCACAGCAGGTGCAACAATGG

>L0422

TTTATATATATATATATATATATATATATATATATTTATTTATTCAGCTCGCATGAAGTAGTTGAACAAAATAGTCTTACAAAATGAAAGAAATCTGTTCATGCATATATTCTAACATGTACATGAAATTCAAATATAACACCCATGCGAAACACCAAATGGATCATGGAACTGCACCAGCAGTTGTGTGCCCAATGATAACCAGAAAGCTTCAATCTTGAAGAATAAATGGGATTTGACATACTGATTCAAGTCTTCCCTTGAATGCCCAAATCCACTCAACCAAGCCTTGAAGAACCAGCATTGATATTCCTATGAAGAATTCGAGCACCACCAGAATCCCCACCATCAATCAACTCTCTCAACCCCAAATTCCCCATTTTCTCCTCTTCCTTCTCCTCCTCTTCCTCTTCCTTGTCCACCTTACTAACCCCGCCAGATCTGGCCACCTTACCCTGCAGCCTCTCAAAGTAATTT

>L0423

TATTCCTGTCTGTAGCTTTTGTGGTAGTGGGAAAAGGCAGTTCCTGGATGGCTATAACAATAACCTTAATGGGTGCACCAATTCTTGTTGGGACACTTGCAAGCATGTGCTACTTTGTTTTCCGACAACATTTCGGAGTGTCTAGAGATTCACAGAGGCGCATAAAAAGGGCTAGTGGCAGTAAGTCTTTCTCATGGTCTGTGTACTCGGCAAACATATCAGATATTGATGACTACAACTCTGATATGGAGAAGATATATGCTTTGTAAATACAATAAGGCAGTACGTTTGCAATCAACTCTGCTTTTTTGCTAGCAAAAAAAAAAATGTTGTTGGTTGGTTCCATTTCTGGATTTTAGGT

>L0424

AGCTATTCCTCATTTCATACCTCTGTAGAGAATAGAGATCACTGTTCTGCTCTGTGGAACAACCTCCTTAATCATGGCGCTCCGTGGATCTACCTTCTGCTACAAAGTAAATTTTATTGATAGGCGGCTGCCATGTTCTTGCATTAATCGTTTCAAGAGGCTTCCCCATCACAAGGACAATCACTTGTTCTTGCCTGCTACTTCTGCAACGCATTATTTTCCTCTCAAGCTGCACATTGAAGGAAGAACTCCACTTTTTAGAAAAACATCCCGGTTGTTGTCACTAAGAGCATGTCAAGTAACAAGCGAAGACTCGGAGGAAATTATAAGTGGTGAAAGCATCATATGGGATGAGCAAGCCTTAATGCGGGATCTACAGATTGCCATTGAAGAAGAGAATTATGCCCAAGCTGCAAAACTCAGGGATAGCCTTAGAATACTCCAGGAGGATAGTAAGGCTTCAGTACTGGCAGCAAATGCTCGTTTTTATAATGCATTCAGGAATGGGGACTTGGCTTCCATGCAGTCCCTCTGGGCAAAAGGAGATAACGTTTGTTGTGTACACCCAGGAGCCAGTGGGGTTACTGGTTAT

>L0425

ATTTCCGTGGTTTTGGGAGAAGAACAGTAAGAGAAGATGTTGGAGGTGGTGTTGAACGATCGCTTAGGGAAGAAGGTGAGAGTTAAGTGCAACGATGATGACACCATCGGCGACCTTAAGAAACTCGTAGCTGCGCAGACCGGTACCCGAGCCGAGAAGATCCGAATCCAGAAGTGGTACACTGTTTACAAAGATCATATCACTCTCAAGGACTACGAGATTCATGACGGAATGGGT

>L0426

ATTCCACAGAGCGCTTAGACTTGTCTGATTTTAGGGTTTCTTTCTAATTTATTATCTTCTTTGCGAAACCCTAATCCTCGGATCAGGTCTGAGCATCTGGTTGTTAGGCTTCTCTGGTCAGCCAAGTTTCATGTCTGAGCTCGAAACCCAGATTCCTTCTACCTTTGATCCGTTTGCTGATGCAAATGCTGAAGACTCAGGTGCTGGTGCAAAAGAGTATGTGCATATTCGTATACAACAACGGAATGGTAGAAAGAGCTTGACTACTGTGCAGGGATTGAAGAAGGAATTCAGCTACAATAAGATACTCAAGGATCTCAAGAAGGAATTCTGCTGCAATGGTACAGTGGTGCAAGACCCTGAATTAGGGCAGGTTATTCAGCTTCAAGGTGATCAGCGGAAGAATGTGTCTACATTCCTTGTTCAGGCTGGCATTGTGAAAAAGGAAAAAATAAAAATTCATGGTTTCTAATCTGCATCAACTCCCTGCATCGTGCAGTTCCCTTGTATGTG

>L0427

TGCTGAAGAGCTCTACCATACCGCTCATAGCGTGACTGAGATTAGTCCTAGTTGAGGTACAAGAGATGGCTGATGCCATCGTCGATGTCGGAGATATAGTCTGTAGCCTCGCCCGAGCGTCGGTTTGCCTTTTATCTGTCGGTGCAAGGATTTCTCTAGGTTGGGTTCTATGGATCCCATCATTAAATTGTCTGCTATACTTGCAAAGAAGGTAATTTCTATCTACTATGCTTGTGAAGATGGTAACGTCTAGGGCTCCAAAACTTTGCAAAGAAGATGAGACCCGGCTTGTTTCTCATAGGTCGATTATTTTGAATTTTGGGGCTCCACTTTTAAGCAATCGGTAGAGTTTTCTTTNACTTCTTGGGTCTCTTTGTGATGAGCCTGACTGAGCTTTAATTAGCTTTACATGTAT

>L0428

TGGATCCCCGGGCTGCGGAATTCGAATCCCAGAACCATGTTCTGATGGAGGATTTCTCCTACAATTGCAGATGAAGCTGTGGGTTCANTCCGAAGAAGAAGGGAGTGGAGGAGGGGTAGAGGTGAAGGATTGTGTTCGAGCAAATGAAGATCGAGAACTTAAGGACAAACTGTTACGTAAATACAGTGGCTACATAAGTACCCTAAAGCAGGAGTTTTCAAAGAAGAAGAAAAATGGAAAGCTGCCAAAAGAAGCAACGCAAATCCTACTTAATTGGTGGAATATTCATTACAAATGGCCATACCCGACGGAGGCAGATAAGATAGCTCTAGCAGATGCAACTGGGTTAGATCAGAAGCAGATTAGTAACTGGTTTATAAATCAGAGGAAGCGGCACTGGAAACCATCGGAGAACATGCAATTTACTGTTGCCGACAGTACAT

>L0429

TGCAGGAATTCGATTCAAAATTCGAATCGGCAACGCTAAAAACCATCTCAACTAAAACCCGTCAAATTCTCTCTCATTTTCCTCGATTCTATTTCTCATCGTTTCGTATCTCCGATTGGGATTTCCGTGTTCTAGAATTTTCCTCCGCGTCTACGAAGGGAAAGCCAAGGAGCTTCTCTTTCTTGAGTGTTTTTGCTCGATCTCCGAGGTTTTTGAAGAATTATTAAGATGGATACTACAAAGACACAGAATACTGTGCTAGCTTTTACAGATGATACCGTTCTTCCTGTAAGTATGGTGATAAATGCAGTTAGGGAACTTGCTAATGAAAGAGCCGAGCAGTGTGATCCTCAAGTTATCACTCAAGCATCATCTCTAAGTAAGCTGCCAGTGGATTCTTCATCTGTGGATATAGTCATTTCCATAAGTAGGTCACTCCAATTTCCCAG

>L0430

ATTCCGACGCCTCTCTGTTTTCTATCGAAAAGGCATGGCACTTTCCCTCTCATCCAAAACCAAAACCTACCTAACCACCTTTATGGCGAGATCAATCCCCCGCTTCTTAGCCCTGCGCGCCCATTCTAATACTCCTTCCCACCCCAACCACACCGACATCTCAGATGACTCCTCCTCCACCGACTCTTTGCTACGGAAA

>L0431

ATTCGGCAAATATTACCAGCTCTAAGAACAAATTCTGTATCTGTAGCTCTGTATGGAATACAAAGGCAGGCTATAATACACATAAAATTACTTTCAGTTTGAAATAAAGCTCCACAAGTGTCTTCAAAGAATCGACCATGCTCATAAAGGAGTTAAAAATTTTAGGTCAGCAAAATATCAGGAATTGTACCTTCCCAAATATAGAAATTATTACATCCAGCTGACCTTCAGACATCACTTTGAGAATTGTGGGGGGTTGGTGATTCTGTGTGGATCATACCCAAGTTCATAAAGTAAGCTTTTGTGGTGTCTCTACAAATTCAGGCCCCAGTACAGGTCTCCTCCAACATATCTATTGGCAGCATGGACTAAAGAGGCTCCAGAAGCATCAATACCCAATGTAGCTGGGATCCCCAACATACTCTGCAATTTGTTCAAGTTTGACATTGAGCAATTCATGGGTATCAGAATCTTG

>L0432

TGCTCCTCCTCTAGCCCCTGTCCAGAGTAGTGGTAGTGGTGGATCATTGCTTGGAAACATAGTTCAGGGCATGGCTTATGGCGCTGGAAATGCTATGGCACAGAGAACTATAGATGCTGTGTTGGGTCCTCGTACAATTCAACATGAAACTGTGGACCCAGCACCCGCAGCAAGCAATATGGATACAGTTTTAAAAATCCTGGGTTATGGCATTGAGGCATTGAAATATGTAATGAGTGAAACAGAAACCCCAAACTCAAAAAAACAGTTCAATTCTAGTCACAATAATGAAAGAGGCGCGCATCAGGGGCGAGGTAAAGGCAGAAGCGATGGTACCTCAAATGGCCAAAAGTGGGCAACAAATGGGAGGCGCAGCAAGCCGCCCTCGGGCAAACCAAGCAACAAGGGCACGTCTTAACCATGATAGCCCTCTGAGCCAGAGACTGAAAAAAAAGAGTGAAGAAGGGTTCTTCCCTTGAAGGCTATATTTGGATGTTTACTACATTTACGTAAGACAAGCATGTGAGTG

>L0433

ATTCTTTTTTTTTTGCTTGTAATTAAGGAACTTTATTTATTAATTGCATTTCTAAAATTCACAGCACTAGTTAATATCCTTAGCAGAGGAAAGATTTGATATTTGATACGAAATGCACAAAACCTAAGATCTAGACACACAGATTAAAAGAACCAAACTTTCATTCAGCAATCACCACCACGAAGACGGAGCACAAGGTGGAGAGTGGACTCCTTCTGGATGTTATAATCCGCGAGGGTCCTCCCATCCTCTAGCTGCTTCCCAGCAAAGATAAGTCTCTGCTGATCCGGTGGAATTCCCTCCTTGTCCTGGATTTTGGCCTTCACATTGTCAATTGTA

>L0434

TGTTTCAACTTTTCAATACCAATCATCAAGTTCATCTTTANCTCCTTCCATGCATTCTGCACTGAATTCTTGGGCCAAACTATCAGGTCCTCTTTTGCTTCTACATCTACGCATAACTTAATCAAGAAGCCGCAGCAACCACCAAGCAAGACCCCGAGATTTGCATCGGCGCTTTATGGACTCAACCTTTTCAAGACCCTCGTTAAGTACTGATTCTTAATTCTGCTGTTGGGTGTCTATCTTAACATTCATACTTGTACAAATCAGGAAACTTACTAGTGAGATTGCTATGAAATTCTTTCGTTGATTCAATCGAAGTTGTTTTAAGCTTAAAAAAAAAAAAAAAAAAAAAAAAAAAAAAAA

>L0435

AATCCTCTCTATAACTCTCGCTCTCTTTTCGTTAGCCGCCTCCGTAACACACCGACCATGCCTCGCCGAAGCTCTGGAGGAAGACCTGCTCCTCGGGCTGCTGCTCGCCCTGCACCAGCACGCAGCCCACCACCCCAACCAGTTAACCATGCTCCTCCTCCAGCTCCTGTCCAGAGTGGTGGTGGTGGATCATTGCTTGGAGGCATTGGTTCAACCATAGCTCAGGGTATGGCTTTTGGAACTGGAAGTGCTGTGGCACACAGAGCTGTAAT

>L0436

TGCAGGAATTCGAATTCTGGAATTCTGGTGGTGCATGTGACAGCGAGGTTGAGCCGATCAAGAATGCCACATATCTAAGGGAATATCCACCCAAGATGTTGGTGCTGGAGAAGGTGCTAAGAGGGATGAAGACCCATGTCACTTACTTAAATGTCACGCAAATGACAGATTATCGAAAGGATGGTCATCCTTCTATCTACAGGAAGCAGAATCTATCACCGGAAGAAAGGAGATCACCACTCCTATATCAAGACTGCAGCCATTGGTGTCTTCCCGGTGTGCCTGATACCTGGAATGAGATTCTTTACTCCGAGCTCTTAATATACGAGTACCAAAATCG

>L0437

GATTCCTCTCTCTCTCTCTCTGAAACGCTCTGCTTTAGCTTTGGAAAGAAAATGGGTGCTCTTGATTCTCTTTCAGATTATCTTTCTGACTTATTTACCGTCGCAAGAAAAAAGAGGAAGCGCAAGCCAATGCAGACAGTTGATATCAAAGTGAAAATGGACTGTGATGGCTGTGAAAGGAGAGTTAAGAATTCTGTTTCCAACATGAAAGGTGTAAGGTCGGTGGAGGTAAACAGAAAGCAAAGCCGGGTAACAGTAAGTGGATACGTTGAGCCAAACAAGGTCTTAAAGAAAGTGAGAAGCACAGGAAAGAGAGCTGAGTTTTGGCCCTATATACCCTACAACTTGGTGGCATCCTTACGTTGCTCAAGCCTACGACAAAAGGGCACCTTCTGGCTATGTTAGGAATGTGGTTCAAGCACTTCCTAGCCCCAACGCAGCAGACGAGAAATTCACAACCTTCTTTAGCGATGAAAACCCAAATGCATGTTCCATTATGTAGGAATTAATTTCTAAGCCAAAAAATAGAAAATCACATATATACAGAATATCAGCAGTTGGAAATTTGGAACTTTTAGTTAATTAGAGGAGTTTT

>L0438

AGCTCATTTTTTTTTTTTGAAGTGTCATCTTACAGTGAATTGACTGTCGAAAATACACACTTGTATGCTCAAAGTTGAATTAAATAGGGTTTTATCACAATACAAATTACAAGAAATGAATATTCAGAACCGTGTCCTCACATCTGCAAATCTATTCCTTGAAAACTAATCAACCCAAGAAGCCACAATGATGCTTAACCCAATAATTAGTTAGGCTGACACTGGCAGCTTGCTCTTGCGAAAGCTCCAATAGTTAACTCGCAGTGGATCGAAATGCTCCAAGACAGAACATACTGCTTTAGCTAAGTCAGTCTCTGGTGGATCTGCATTTGATGTCCTCAGAGCATTAACAGCATCTCTAAACTCTTGGTTTGCTTGAAAACCATGGCAGAGAAGATCCAAGAGCATGCTCAGAGCAAATACATGGTTGGTTTTGCCATTCAAAACCTTCAAACATACAGAAGAGACTTGAAGATCATTACTCAAAGATTGGGTGTCCCCTTTGTACAGGCCTCGAAGATATCTCCATGAGCTCTCATTCTCAGGGTTAGCTAAAATGGCCT

>L0439

GCGAGTTTTTTTTTTTTTTTTTCTCATTAAAAATTGTTTTGTACTGTACATTCTCATTTCTTAGAGCCTTCACTTGTCCCAGCAATTCCGCACGCTCTGACCTCTTTGCTTTCAATTCTTCAGTAATCTGGAAACGGGACTGATTGCTCTTCTGAATCTCCCTGTCATACTGGTCAATTTTGGCTTTTATTTTAGGATCATCAAATGGTCGGTATCTTACAAAATAAAAGGTATGGATCTGCTTTGGAGCAGGCCACTCATCAGCTGCATCCGTTGGGAACTTAGAATCTGCAACCTCATCCCCTTCTCCTTTAGCTGGCTCCTCCTCATGCGAACCAAATTTTATAGGTTTAAAACCAGATTCCTTATCCAGCTTTCCATTTTCCTTGTCATGCAAAAATGAATTATCTACTTCAGTAACAGTCTCAACAGGTACTTGAGCCATCTCAGATCCACCAACCTCCACACCCATTCTTCTGAGCAGCGATCAACCTTGAAAAATGGAAAACCAAAACGGTAGCTAAACAGAATGGGGCAGGGAAGAAGTGAAAGAAAGACGGAATTGGATAC

>L0440

ATTCCGTTGCTAGGCGTCAAGGTTGTTCTGCACAAGTTGCTTCGGAGAAAGGGCTTTTCTCTCTGGTTTTAATCTGCAAGTACCTGGTTCAACACATTATAGTATGGTATTTTACTTTGTGACAAAGGAATTAATACCTGGTTCTCTCTTGCAACGGTTTGTTGATGGTGATGATGAATTCCGCAATAGCAGGCTTAAACTTATTCCATCAGTTCCCAAGGGCTCATGGATTGTGCGCCAGAGTGTTGGAAGCACTCCTTGTTTATTGGGAAAAGCAGTCGATTGCAACTATATCCGTGGTCCCACATATTTAGAAGTTGATGTTGATATTGGTTCTTCAACTGTTGCTAATGGAGTTCTGGGGATTGTAATTGGAGTAATTACGACATTGGTGGTTGATATGGCTTTCCTTGTACAGGCAAATACCACAGATGAATTGCCAGAGCGGTTGATCGGTGCAGTTCGTGTTTCACACATAGAGCTCTCATCAGCTATAGTACCAAAACTGGATCAAGATCCGTCGTGACTAACACCAACTATTGACCTCTAATGATTTTACACAGCATTTGTTTA

>L0441

GAGCTCAATTCGTTTTTTTTTTTTTTTTGCGCAAACAATAGCTGATAATTATTTGTCTGCAGTAGCTTCCGTTCACATTCCTGCTTCAATACCTAAATTGTGCAAATGTTTTCATGATTCCAGGTTGATCTTCATAAATAAATTGCATAATTAAATGATAAATAAAGCAGTGACTAAACCAAAGTCCCTACACCAGACATTATAGCACGAAGCTCCTCCCCGACTTTCTTCCAGACCCCATTTATCACCAGACAGCTTGTGATATCATCTGGTAATAAAGTAT

>L0442

ATTCATCAACAACTACTCTCTTAATCTCTCTCTCTCTCTCTCTACAAATCTATCCATTTCTGTCTAGATATAATGTTCCTCTGTTAGCAGCCCTAGCCCTGCTTCATCTATGCTAGGGCTGCAGGAGAAGAAGAAAATTAAACAAAAACCCATTAATCAATCAAACCCTAGACTCATTTACTTTTAAAGGATTAAACAGATAAATATATACCAATGGTCTTCAAGAACAAACTCTTCTTCTCTTCTAAGAAATCCGATACTTCTAGTCCAGATGGATCCAACAGCCCTCGATCTATCGGCTCCAATTCACCGATCCGATCTGACAAGAAAAAGCCCAAATCTTCATCTAAGGATGAAAGTCCCACTGCCCATAATACTGGTTTTGCTGCTGCAGCTTGTAGACAAACCCAAGTCAAGGATGGAGTCAAGAAGAAGGACTCATTCAGGGTAGGAAGTGTGCTCATCGCTGGGA

>L0443

CAATTCGAGAAATTATTGTTACCAAATGGCAGGGCAGTCAAGAAAATGGATGATACTAGTGGCTACAACATGGATACAGGCATTTACAGGGACAAACTTTGATTTCTCATCCTACTCTTCCAATTTAAAATCTGTTCTTGGGATATCACAGGTCCAGCTAAACTACTTGGCTGTGGCATCTGATATGGGAAAGGCTTTTGGGTGGTGTTCTGGGTGGTCTCTTTTGCATTTTCCTCTGTGGGTTGT

>L0444

ATTCCACTCTCTCCTCTTCCCTCAAGACGGGTTGCGCTCAATCTCTCCCCTTTGTATCTCTGTTGAGATCAAAGTTTAGCACCCAATCGCAAGCTAATACTATCAAAATGGAAGCAAGTGGCAACACTGTACCCAGTATCGTTGTGTACGTTACTGTCCCTAACCGAGATGCAGGAAAAAAGTTGGCTCAAAGCATAATCAAAGAGAAACTAGCAGCATGTGTGAACATAGTACCAGGTATTGAATCAGTATATGAGTGGAAGGGAGAGGTCCAGACAGATTCAGAGGAGCTACTTATAATCAAGACTAGGCAATCTCTGTTGGAAGCACTAACAGAGCATGTCAAGGCAAATCATGAATATGAGGTACCAGAAGTAATCTCCTTGCCTATAACTGGTGGCAGCATTCCATACCTAGAATGGCTCAAGAACAGCACAAGGGATTGACTTCTCACGTGTAGGGCAAAAATTTGAGCACAAAGATTGG

>L0445

TTTTTTTTTTTTAATGCAACGAACAAATAATATATATTTTCATCAGATAAGAAAAAATAATTGGAAGATCATGAGCAAACTGATCTCAAATTCTTTGATAGATATATAAGAACATTTCCGCATTATTCTACTTCAAAAGCATCGCAACAATTTCTAGTAAAGAACTTTAAACCACATGTACTAGACATATAACTCCAACATAAGGAAATAGTCCATTAGAACCTTGCTTAAGCACCAGCTATAGAGCCTTCCAATGTCGCATTGATTTGTTACCAAGATGCTTTCTTTACACCATTCAACATACCTTGGGATGCCAACTCACGGAAAACAATACGAGACATTCGAAAGACCTGATAAACAGCACGAGGGCGACCCGTAAAAATACACCTATTTCTCACTCGTGTAAAAGAACTGTTTCTCGGCAACTTAGACAGCTTGTATTGGAATTTCTCACGAATTTCCGTTGGAAGCTGAGGATCTCTAACAAGCGCTTTGAAAAGATTTCGTTTCAATTCAAATTTGGAAGCAAGC

>L0446

ATTCCTCAAATCAGGAAAGGAGACGGGATGGAATTGGATATCTCTTTCATAATGCCTCCTATTTGAGAGAGAGAAATGCTTGCATAAATCCTTTTGATTTAGGTGTGCTTGCGGAAGCCTTTCATATGAGAAAAACTACTCCTGGTTGACCTGCCTTCTGCTGAGAGAGGGATCCCTACTGCAATAATGAAGTCAAGGAGCAAATTCACAAAAGACAAGGAAATGACGCACAAAAAGCACAAGGACAAGGATCAGAATAAGCATAAACATTGCAAAGAGGATGGAAGTAATAATAAGGATAGAAAAAGAAGTGGAACGGTACCTGGTCTTGAGAACTTGAAGAAACAACATGACAAGAGGAGACGATGCAATGGATTGTAAATGTCTTTCCTACAACCTCAACATGAGAAAACCTGAAATCAAGAATATTGAAGTTTGGCAAGCCACGGGC

>L0447

ATTCTTTTTTTTTTTTTTTGGAAAGGGGAAGTGTGGTGCTGCTCCATATATCATTCAGGAATAAACACGAGCCAACCAGAGAGTCACAATGTGAAAAAAAGAAAAGGAGCATGGTCATTCTTTAAATAAAATTCATACAATTATAATTTCAA

>L0448

GGCTGCAGGAATTCGAATTCAAAAAAACCGAAGAAATAAACTCTGCCGATCAGTGGCTGCCATGGATAGCGGCGGCGGCAGCACGACCTGAAGTCTTGTATTATCTGGAAAATCATCTGCAGAGAAGGAAATCGTTCAATCTCTGAAGAAAAATAGTGCTATTAAACTCCCTAGTAATACGGAAGTTTCCATACTCTTGGAATCGGAGATTCCTAAACTGGACAAAGAAGAGCCTTTCAATGTGGAGCTGTTCTTCAATTCTCTTTCCACTATTCGATTCGTTTTGACTTCTTCAAAGGACTGGTCAGAAGGAAACTTTTATGTAGAAGATTCCTTCAGCACCATCAACTGGAAGCAACATGAACTGTCATGTTGTAACATGTGCGCAACAGAGGACATTTTATGAACTTCCTCTTTGGTCTTTTGTAGACACTTAGTTTAATCAAATTATTCATGTTATATATACAAGTCCATTTTTAAAATGAGATCTGGTATCACTTATACAAATTTTCTCATCTTTTGTCCTCATTTTCTAATCTTAAATCTTTCTTTAGTTAATTAGGTCGGATTTGTAAACTATGACAATGGTACAACGTTTTTGTACTGTTGCAACCAGAAATCG

>L0449

GGCTGCAGGAATTCGAATTCGTATCTTCCATAATCTCCCCTGGGTTTTTCTTGGTATTAGACCTTGAGCCAACAAAAGCCGAACTACTTCTCCTTTCTCCAAACGAAAATGGTGGGAAAGAATATAGATATAATCAAAGCAGGATTTGAGATGATGAGGAAGGTTTTCATAAATAGATTTGGCAGCAGCTAAAGAAGTCTGACCTTCGTCTTGTTGAATTATGGTTGGAATTGGTGCGTCTGATAAAGCTTTTCCTGTGGGCATCTCCCCGTAATACCACTCACCAATTAGGTCACCATCGGAATTACGATTGAAGGAGAAGAGAGGCATATTGATTATGAGATGAAAATGAAGAGAAATAGTAAGAACGTTGAAATGAGGTAAACTTTTTTGGTAGTGTGGGGGGTCTTTGTTTNCTAGTGTGGATGGAGCCAATCGTTTTTGTACGAGCTAGCTGGACTCCACCAGAGGGGGGAAATTTATAGTCGTCTATAATTTAGGCCATTAATTTTTAGAAAATTATTAATTAATTTTTTCTTTAAAATTTACTTATTAAATATACTAATTAATCCAAAAAAAAAAAAAAAAA

>L0450

ATTCTTCACTTCAGGCGGTGTCTCCAAAAATGTCGTTGAGGGCTCTCCTTCTACCCTCACAAAATACCCTTACAAGTTGTCCAGTGGGCGAAACTGGAGCAAATTCTAGATGGGCACCACCCAAATTCTCAACATCGCGCGGAGGTCTCTGCAAAACCAAGCATTCATCCAAAAGTTTGTGCTTGACAACGGGAAATGGCGCAATTACAAAGAACCCACATGGGATTGGAGAGGAAGATGGCTTGATCATTGTCGACCACGGTTCGCGTCGTAAGGAATCAAATCTCATGCTAAACGAGTTTGTTGCCCATGTTAAGAGAGAAAACTGGGTACCCAATTGTAGAGCCTGCTCATATGGAGCTGGCAGAGCCCTCAATAAGAGATGCATTTGGTTTATGTGTTCATCAAGGGGCAAACCGGGTGATTGTAAGTCCATTCTTTCTCTTTCCTGGACGTCATTGGCACCAGGATATTCCTTCCTTAACTGCTGAAGCTGCTAAGGAG

>L0451

AATTCGTTTTTTTTTTTTTTTTTTTAAGACAAACATATATACATTATTTGGTTAAATCTCAAACAAGTTGTAGTAATTTAGCATTCAATCAACAGTGATGACAAGGAAGTAACCAAGAATCAAGTACAATCAATAACGTGCATAAACTATCATACATACGAGGAAGAGAAAGAGAACCTATGAGAGATATCAGGAGAGACTTGAAGAAGACGTGGAAAACGGAGAAGCAACTAAACCATGAATTAACTCTTCTTATCATATCTTCATTTTTTTTTTCCCTTCTCTATTTTTAGACCAAGATTAAACCCGATAAATACACAATACTTTTCCATTTTCTTTTTCTCTCTATCTTATGTCCTATTATTATTATTATATTTCTTTCACCTTCTGGAATTCAGTCCAGTGTCATTGGCAAGGTCATGCAACATCTGAAACGAGCCTTGCGGACACTGGTCAAGATCTCGGTCTCTGCATT

>L0452

ATTCGAAATCCCTCCTTTTTGCCTCACTCCCAGACCGTCAGAGTCCAGACGCCGTCCGCTTCCGCCATCGTCTCTCTTCCCCTGGTGGACTCATCTCGTGAGTCGTGTCTCCTTTCTTCTCTGCAAACCGAAAAAATCTTAGTCTCCTTCTCCTCGTGTCTCCGCCGTTGGTGGTCTGTCATAAATCGTGTAGAACAAGAAACCTTTGATTTCAAATATTCGATCGATTGTTGCTCTTCGTTGGAGGCTTGGATGGACCCCAATTTAATTGGGATTACCTGCCTGACCCTCCCCTATAAGTTGAGGGGTCAAATTGGCCTTTTGGCTGAAAGAAGTGAAAACGAAGATATGGCAATAATACGACAGAGAGTGGTGGCTGCACTGCCGAAGCTGATACAAAGCCTGCGAAAGCCCGCAATAGAGAGAAGGAGTGCATTGCCATCGCTGAGACGTGCTTTCTCTCTCTACGATCAGATCAATCTCATTGACAACGCTCCTCAGGACCAACTCCGCTTTCAGGGGTATACTGACCCGGGATTCACAGTAAATGGGGTTGAGTATGAAGGCAGTTGCTTTGCG

>L0453

ATTCCAAACTCAGAAACCTTGACCTTGCGGAGTTGGCTGAATTTCTCAAGCCATTGAATGAAGTTATCATGAAAGCAAATGCAATGACAGAGGGAAGGAGATCTGATTTCTTTAACCACTTGAAGTCTGCTGCTGACAGTCTGACAGCTTTGGCATGGATTGCCTATACAGGAAAAGATTGTGGTATGAGCATGCCTATTGCACATGTTGAAGAGAGTTGGCAAATGGCTGAATTTTACAACAACAAGATTCTTGTAGAGTACAAAAGCAAGGACCCAAATCATGTTGAGTGGGCCAAAGCCATGAAGGAGTTATATCTTCCAGGTTTGAGGGATTATGTTAAGAGTCATTATCCCTTGGGCCCAACATGGGGTGTTTCTGGCAAAGCACCAGCTTCTGCTCCATCTAAAGCTCCTGCTCCTCCACCACCTCCACCTGCCTCTCTCTTCAGTGCTGAATCTTCTCAGCCTTCGTCATCAAAGCCAAAAGAAGGGATGGCTGCTGTTTTCCAAGAAATCAACACAAGCAAGTCTGT

>L0454

ATTCGTTTTTTTTTTTTTTTTTTAACAATACAAGGACAACAATCAATAACTAGTCACTTGGGCTTTGGAGGCAAAGGGATCCCCCAGTTCAGTGCAATATACAATGACAATTTGACAAAAAATGGCTTCTGTACAGGATATATACTAAACACAACCGCCAAATACCAATGTCAATTTTCAGGAAGAAAACCCAGCTTCCATTGCTCACAGGACCCCAGTCCAGCAGCGTCAAATCTAAAATTTCTTAAGCTTTTGCCACGGCCACCATCAGGAGGATGAGCATCAGTTCCAGGCATAATTGATTGGTTCTCCTCTATTGTTGTGCCAATGCTGCTTCCTACAGAAGTGTTCTTCGCTACTCACACACACATGCCTCCATGTCTTTAACTGAAGCATCATTTGCAATAATTGGTGCTTCGTCTTGTGGAGACATGTCCAAAGAGTCTTCAGTAGACAACTTGATATTGCACATTAGAAGTTCTAGCTCCTTCGCGACTGAGAGTGCGCGGCTTTTCTCCTTCGCGGAGAGAGAGAGAGAGAACTAGTCTCGAGGGGGGGCCCGGTA

>L0455

ATTCGTAAGCTGTGCTATACAGATGGGAAAATTGTGCTCGAAGCACTAATTCTAACCTCGGCTGTGGTTTGCTCGCTAACTGGATACACTTTCTGGGCTTCTAAGAAGGGGCAAGACTTCAGCTTTTTGGGACCAATTTTATTCACCAGCCTCATCATACTCATCCTAACCAGTTTTATGCAGATGTTCTTCCCTCTTGGCTCAACATCTACAGCTATCTATGGTGGAATTAGTGCTCTGGTTTCTGTGGGTATATTATTTATGACACTGACAACTTGATCAAGCGTTTCTCATATGATGAGTATATTTTGGCCTCTGCTGCTCTCTACCTGGACATTCTCAATCTGTTCCTATCCATTTTGCGGGTTCTGAGTCAAAGAAACAATTAGTTTGTACAGGATACTTTTATCAGCCTGGTTGGCATGCAAATTACATCTTCATAAAAGCTAATTCCAGTTAAGATTGTGGATATTTGGTATTGTGGTGAACGTACTCTTCTATTAGTGACGAGTGTTGTTGATTCATGCAGAATCCTAGTGACCTGCATTTACCTCAATCAACTCTGCTATAGTAAATTGATTTGCTGTCAAAAAAAAAAAAAAAGAGAGAGAGAACTAGTCTCGAGGGGGGGCCGGTA

>L0456

ATGGTAAGCCCATTTGTGAATCTCTCATCATCGTTGAGTACATTGATGAGGTTTGGTCATCTGGTCCATCTATTCTTCCTTCTGATCCTTATGATCGTGCCATTGCAAGATTTTGGGGTGCCTATGTTGATGAGAAGTGGTTCCCAAACCTGAGAATCCTTTCAACTGCTGAAGGAAAGGCACGAGCTCAGGTTATAGGGACATTGGTTGAAGGGCTTGTAATGCTGGAGGATGCATTTG

>L0457

GAATTCCCATAATTAAAAACTTGAGAATTGAAAATAAACAATGTTTCTAAGATTACGGATTAAGCAATAAAATATAAATCCAGATGATGTCTCACAAGTTGAAAGGGCTAAATATTCAGCCAAAGCTAAAAGCAAGAGTAGTATAGTATAGGGGTACGCATATCCCCCACCAAATGTAATGTGATTCAATAGCTAATTAGCTATTACTCTGATCGATTGGCGTACCTTTCAACCTTATCATCATTCAGATTAATTCCAACCATAGCCTCGCCCAATCCACAGCTCACCTCCGCAAGCACATCAGGATCACTATAATGCGTCACTGCCTGCACAATCGCGCGGGCTCTCCTCGCCGGATCCCCACTCTTAAACACTCCAGAACCCACAAACACCCCATCGCATCCCAGCTGCATCATCATGGCTGCATCCGCTGGGGTCGCTACCCCACCCGCCGCAAAATGCACCACCGGAAGCCGTCCTAACTGCTTGGTCTGCATCACCAAATCGTAAGGAGCCGNAATTTTCTTGGCGAAAGTAAAAACCTCATCGTCATCCATG

>L0458

ATTCTTCTTAGCCATACAGCACGCTAACGTAATGTAGCCGGTCCTAGATTTAGATCTCTGTAGCCAATTGGAAAAAGTCGGGTTGCAGCTACCTGTGTGAAAATGGAGAAGAAACGATCTTGGACCTGTACTTTGATTGCGCAGGTTTCTCTCTGTATTGCCTTTTACCTCTCTCTGAACTTGGGTCAGCATCAGAATTCCATTTATCATACCAGAGATGGCACTGCCAATACCAGACCTCTTGATGTTTATTTTCTGAGTGTTGGAGGAGGCTTTAGACCCCTGAACCAACAGATCCATCTTCTTAAACTGATGGAGAATGTGGCAAAAGCTTACAAAGCAAAATTTGTGGTGAACATTAGTGAGCTGGGGGAAGATGATCCTCTAACACAGAATGCAAGCAGACTCTTCTCGTCAATGAACATTCCCTGGTATACTACTAGAGCTTCAAAGGGTTGGAAAGTTGGTTGTTTCATGGAACAGATCAACTTAACACATGGGAAAATGTNAACCATTGCTGGTGTGGATACTGAGTCATTACAGGATTCAATG

>L0459

CTAGAACCGTGCGAGGGCCTGGTGCCGACAATGCTCACCGATTCTTCCTTCTGAACCATCACATGATAATACTTCATACCCACCGTTTCTAGTCGAATCCTTCGCTCTTTAACCCGGAACTGGTTCCAAATCTAGCTTTTGCAATCATGGAAACCTGCAGGAACGCTAAGC

>L0460

TGCAGGAATTCGAATTCGTTCGTTAACGGAATTAACCAGACAAATCGCTCCACCAACTAAGAACGGCCATGCACCACCACCCATAGAATCAAGAAAGAGCTCTCAGTCTGTCAATCCTTACTATGTCTGGACCTGGTAAGAGAGAGAGAGAGAACTAGT

>L0461

GATCCCCCGTGGCTGAAGGAATTCGAATTCCTAAGAGTGGAAACAAAATTCAAAGTTATATCAGAAGGTCATTGAAGCTGTTCCTTTANAAATTCAGACAGTCATACCGCAGGTTTCAGGAATACCAGTTGTATTTATTTCAGCATT

>L0462

AAAAAAAAATGCTTCTACTTTCTCTCTCTGAAGTAAAAGCCGTTTCTGTTTAACTTTCTCTTCATCAATATCTGTGATGGTCAGACTGGTGGTCTTGGCTGTGGCTGTGGTGGTGGTTCTTTAGACGAACAAAGAAAAGATGGGTTCTTGCGTTTCTTGTCCACAATAGCGCCCCAGATTCGGCCATGAAACTAGGAGTATCATTTGGGTCCAAAACGGACAATCTTGTAATTCCAGAATCACCCATCAAGGATAAACACGTCAATGTTGACCCTCCAATCAA

>L0463

ACAAGAAAAACCTATCTTTAATTTATTGCAAGTCATCAAGGTCTGTACAAATACGATAGGAGTTTATGAAAACTCACTATATATTGGTTAAAGACATACTGCATAAGAACATATGAAATAAATAACACACCCTAATAAGAACGGAAATTAAGTCAGCATTGAATCAGCAGTCAATTTCGGTCTCCACCTGAATCAACAAACGGAAAACTGGAGCAATCCAGTAACCTTCAGCTTAATCCCATTTCCTTCCTAGATAGAAGACATCAGATGCTTGACTTTCATCCATCATGAGATGAATCTCAAATTTGTCTCA

>L0464

ATTCGACACTATACTCTACTCTCTCTCTCTCTCTCTCTCTCTCTCTCTCTCTTTCTCTCTCTCTCTTGTTCAGGTAATTACATTTCATTGATGGAGATGATGACTGATGAGATACCGAAACGCCCCTATTTTCGCCAACAAGTAAAATAATAAAGAAATTAAGAAAACAACTTCGCAACAGAACTCTAGAAAGCTGCAAGCTTGCTTCTTTTTCTTCTTTTCATCGTCGTCTGTATTTCTCTTCTGCGAATTTCACTATTTCTTATCAATATTTGTTAGAGGCAGGGAGGAAATTGCGGACACTAACTTTCTAATACATTTCTGGAACGAATGGATCAGGGGCACTCAACTAATGCTGTCGACGATAAGCAAGGCGAGTATATTCTGATTACTGATGCTGAGGAACCTCAGTTGGGACTGTTTGACAAACCCCTTCCTTGTTTCGGCTGCGGAATTGGATGGTTTTCTCTCTTGCTTGGATTTGTGTGCCCATTGATTTGGTACTTAGCA

>L0465

ATTCTTTTTTTTTTTTTTGGACAAAGCTCGGCTTTTATAATGAATAGGGTGAATTCTGTAACCAAAATAGATGCACAAAATTGATATTTCTCTGGAAACAGCGTAACATACACTACTTTCACTACAGTTGCTTCATCCTTTTCATATAATATGAAAAAGGGAGGGTGAAAAAGGAAAAAAGAAAAAAAGGAAGAATCAAGAGAGCTCTGTAATTACACGTTCACACTTGTCTTCCAAAATCTTCACAGCTTCAGTAAATAACACTTCAGGGGGCATTGCTCCGGTTGATTCAATAGTAAATATGAAATGATCTTTAACACGGCGCAGTGCCACACGTTTCTCCCATTCTTCTCCCCCTCTAATGCATTCCCTACACAGTGTGCAAGCTCGTGGTCGAGCAACGGTTGCCCTCTTCTTACCTTTCCCAATGTCTTCAATATCAAATACTTTAACAG

>L0466

TTTTTAATGCTATTCATCACTTTCTTAAGCACCAAGATAAAATACATAATAATCTTGACCAAAGAGCCATACATCATTTTCTAAGTAAATTACAGGAAAACAAGACGACAATAATCCAGATGTGAAGAATCCTACAGCACCACAAATAGCAGAAGCGTATAAAATAGGAACCCAGTAGACTCCTTCTGACCATCAAATTCTTCTCAGTAACCAACAAGCAATAGATATATTACAGTAGATCCAAATTCCTCTAAGGTGGGAAGACAAAACTCGTTGTGCAAGAAGATACAAGAAAAAGGAGTCAAAACCTTGAACAGAGCCCATCTCTTTCTCAACCTTAGCTTGCATCTCGCGAAGAGCCGCTGCTTCCTCCTCCATTTCCTTCAACCTCTTCTTCATCTCATCCAGCTCCTTGACGGCGTCTTCATCGGCGGCGGACATGTCTACGTCGGTG

>L1530

ATTCCACATACAACGGAGGAACAAGACGAAGAAGAAGATGGGCTTACAGAACCGGCTAAACGACTTATCGTCTGATTCAATCCCCTTATTGCTGCTAGCTCTCATCGCTAAATGCGTCGATCATCTCCGTTCTTTACTCTTCACCCTCTTCCACTCCTTGGGTCTTTCCCGATTCGGCCCTTCACGCGGCGTAGTCCACGATGACCTCTTGGGTTCAATGGGCTCTGGCCTCGCTGGCCTCATCGTCCTTGCCGAGCAGCTTAATCTCAACCGCGTTTTCTCTTACAGGTACTGTTGTGGTAGAGGCGATGGTACCAATAACGGAGAATCGGATTGTGTGGTGTGCTTGTGCACCCTAAGAGATGGGGACCAGGTCAGGAAGCTTGATTGCCGCCATGTCTTTCACAAAGAGTGCTTTGATGGCTGGCTTGATCATCTCAATTTCAACTGCCCTCTCTGCCGGTCGCCGCTTGTCTCTGATGAGCGCGTCGATTTCGCGCGTAGGCGCGTGGGTGGGGATTTAGTGAATTGGTTTTCTATGCGATGAAGCGGCGTCCGGCTGATGGTGGTGAGAT

>L0467

ATTCCGCTCTCTGTCTCTCACCGCCACTGGTTTTTCNGGGTACGGATATAAACTAGAACGCTTTCATTTTTGGCTGTATACGCACCTGTTTTTCCGGTTCAGTCCCTGCCGGGAAAATCAAGTTCTAAACTGAATCTTCCCTTTCCTGATGCTTTCCATTTCCAGTCCATCCTCGCCGGAACCCTATATCAGAAAACCCAAAAGCCAGCCGTACGAAGTCCTTCCTCGCAAGAGCTCGCTTAACTCCCTTAATTCTCTCCTTGCGAATTCTTACTTTAATCAATCCCGCGCATGGCTACTCCTCTCCATCCTCTCCATCCAGATCATTCTCCTCCTTGGTCTCCGCTCAATTCCTTTCTCTTTTCCCCACCGCCAACATTTCCCCTCCCCGTATACTGACCATCTCAACATCTCTGCCAAAACCATCATCAATATCAGNCCCTCTGCAGCAGAAACTACTGATGAAGATGAATGTGGATCAGGAAGAGTCTTTGTCTACGATCTCCCCAGCAGGTTCAACACTGA

>L0468

ATTCTTCTTTTTATTGATGAAGTTCATACACTTGTTGGGACTGGCACAGTTGGACGGGGAAACAAGGGATCTGGACTTGACATTGCTAATTTACTGAAGCCATCACTTGGGAGGGGTGAATTACAGTGCATGGCATCCACTACGATAGATGAATACAGGACACATTTTGAAAGTGATAAAGCATTAGCTCGAAGATTCCAGCCTGTGTCAATAAATGAACCAAACCAGGAGGATGCTGTTAAAATACTGTTAGGTCTTCGTCAGAAATATGAGGCCCATCACAACTGCATTTTCACACCTGAAGCCATAAATGCTGCTGTCTATCTGTCAGCAAGATATATTGCTGATAGATATCTTCCGGATAAAGCTATTGATCTTATTGATGAGGCAGGAAGCAGAGCACGAATTGAGGCCCATAGGAAGAAAAAAGAACAACAGACCTGTATCCTTTCGAAGTCACCTGATGACTATTGGCAAGAAATTAGAACTGTCCAGGCCATGCATGAAGTGGTTCT

>L0469

ATTCCAACCGGATGAGAATCGCAATGAGTGCAGCAAGAGGCCTAGCTCATCTACATGTGGTTGGAAACGTGGTCCACGGCAACATCAAATCCTCCAATATCCTCCTCCGACCTGATCAGGACGCTTGCGTCTCCGACTTCGGACTCAACCCGCTCTTTGGCACCTCTACGCCGCCCAGCCGCGTCGCTGGCTATCGTGCCCCGGAAGTCGTTGAAACCCGTAAAGTTACCTTTAAATCAGACGTGTATAGTTTCGGGGTATTGCTTCTCGAACTGTTAACGGGGAAAGCACCCAATCAAGCCTCCTTGGGCGAAGAAGGCATTGACTTGCCCAGGTGGGTGCAGTCCGTGGTTCGAGAGGAATGGACGGCTGAGGTATTTGATGTGGAGTTGATGAGATACCATAACATTGAGGAGGAAATGGTGCAGCTGTTGCAAGTAGCAATGGCGTGTGTTTCGACAGTGCCTGATCAGAGACCGGCAATGCAGGAGGTGGTGAGGATGATTGAGGATATCAATAGGGGAGAGCCGATGATGGGTTACGGAAATCTTCCGATGATCCA

>L0470

ATTCCCTTGCTTTGATTGAGGGAGCTGGGATCATGCTCAATAAAGTAATGAGTGCTCAGCAGAGCATGCCTGTAATGATTGATGAATCAGTACCTGCTATGGCTGGTGGTCCTGGATTCCCCAT

>L0471

ATTCAGCAAAACATAGCTTGTAGAATTTCAATTCAGACGCGTAGGGATACGAAGGATGAAAATGTGGTGCGCTGGTAGGTGCGATTTAGGTAACGCTGGCCTAAACGAAACCCAACCGCAAACAGGCTGTAACACCAATTCTTTTTGCCTAGCAGCCGACCCATCCTCTTTCTGACTGCGTACACATCTCCTTCCTCATCCACCGTCGACGGCTTTGAGCTGCCGGACGATCANGTGCAATTCCCTAACAAACCTTAGGCACATTGATTTCAGTTCCCCCACCCTCCCGAACGGACTCTCATTAAAGATCAAGAAGGCGACCGACGATCTAACCGCACTGGGCGC

>L0472

TTTTTTTTTTTTTTTTTAAATAAAAAGTTCGTGTATTCCATTTGAAAATGTTGTATTATACAAGACAATAACAAGCAATCATCAGTTGCTCTGCAATAAAAATCAAAGTATCAACCTGTCATTTCCTTTTCCTAGAGACCAGTAAATAATTACCAAGCATGATGCCGATCAGTGTCATGATAACATACCACACCTAAAGAAAGGCATCCTCCTCTTGAAGACAGAACGACATAAAAGACTAGTTCTCTCTCTCTCTCTTCCTCCCATACTGACAAATTAGCTAACAAAAGACTGTCAGAGTCTAGAGCTCTGGTTGCAGTTCCACCTGCGAGTGTTGAGGCACTTGCTATTGTTCCTGTTAACCAGAAAGCTAGGCGACCTGAACTTGTACAGCGTCGAACCAGGAGACCGTTCTCTGTGTCAGAAGTAGAAGCACTTGTGCGGGCAGTTGAGGAACTTGGAACTGG

>L0473

CGGCGAATTCGTTTTTTTTTTTTTTTACACAAATATCACCAAGATATTATTAGATTGTGAGTTCTCCAAGTAGATTCAAGAAAAACCAGCTGTATCCGCTAGAAGGTACAACAGTATCACAACAATAACAGCGGATCAGGCAGCCAATGAAAGAAGAGTAACAATTGATTTTTGTTTTGGTTAACTGATTTTAAATAGTGTACTGTTCCTATATGTGCATGTTGTTTTGTAAAATGTATAGAATAAAACATCAAGGGGCTAATGAAAATTGTTACCTCCTTGCTCATGGTGATTATTTACAGGTGGGCGAACAACAGGAATATCCAAGCTGCTTCCTTCTGTCTCAGGAGCAACCTCTCTTACATCAGCTATTCTCGACTCTGTTGGCTGAATATCACTCCATTCAGCATCTCCAATTGGCCGGAGGTTAGTTTGGTCTGCTCCCATGTGCACCACATTCTCCCCAGAAAATGGCAAAACTCTAGAAACACTAAGACAACTTTATCCACATTCTCCTCCGACAAGCCCACGTGTTTAGTCAGCATAGAAGTGCTGCCAACAACATTTGAAGTCTCGGTCTTCTGAAA

>L0474

GATTCGTTTTTTATTTTTTTTTAGATGATTCAATTAAAATATCACTTCATGTAAAGAGTGCCAGTAAGAAAAAGAAAATACTGCACACTAACATAAGACTGAGAGAGGAACTTCATATCTAGAGGGATGGGCAAAATACTGCACCGTCTTCCACTCCCGAAAGCCCCATTCAAACCACACACAGAAAAGGGAAAAAACAAACACCCATCAAAACTACAAACAGAAAATCCAACGCGACTTCTTGCTCAAACTGCAGCTTAATCAACTTCTTCAATTCTAGGGCCAGCACCACTGCCACCAGCTGATGGGACATCCTCATCCATGCCGCCACCCATGTCAGGACCAGCACCACCCTGGTACATCTTGGCGATAATAGGGTTGCAGATGCTCTCTAGTTCCTTCATCTTGTCCTCAAATTCGTCTGCCTCAAGCAAGCTGGTTAGCATCCCACCACTGGATAGCCTGTTCAATAACATCCTCGATCTTCTT

>L0475

TTCTCAAAGCAAAGAAATTCTGTGCTTCATGCCTACTTCACTCTCAAATTCTTTCATTAGCTCACAAACACCAAAAACAGCCCCATACGCATCCAAATCGAGAAAAGCCCTAAGTAGAATGGGGTACCCGATTCCGACTTTCGTTTGATCTGTGCTAGTATTGACAAATTGGGACCGAAAAACCCCAATTGTGTTCACCTTACCGGAAAAGTTTAACTTCCGCCGGAAACCAACATAAAGCTGGAATTTTGACTAAGCGTGATGGGAAAATGAGGCGG

>L0476

ATTCGTTTTTTTTTTTTTTTTTTTTTTCCAAAAGCCACCATAAAACTTGAAATAGGGCCCCATTGGCATAGCTCTTCTGGCCGGAAACCACTAACAATGATTATTGTTGTAACCATTAGATATTTAGATTCCACTGCTCATGAACCAAGAAACACAAACCTATTTAACTAACAATGTCAGCCATATCATCTGACTTGTTTATCAAAAATCAATTGGTTCAACAATAAGAGCAAAACTTCCATTGGTTCGACAATAAGACCCTAAACATAAATACCAGAACCAAGCAGCAGGAACAAGGACCCGAAACCCAGAAAGAAAGATGCCAATGCTCCTGTTGTAAGCTCCTTGGCAAGACTGCGATTTCGCTTAGATGATGTTGCTTCATAGATGAAGAAGGAAGCAGTGATCACGAGGCCAATCGCAAGCATAAAGACGGATAGGGTTGGATACCATGAGTCCGGCACGGGGCTCGTGATCGGTTTTGTAGCCATGG

>L0477

GGGCTGCAGGAATTCGAATTCCGAGAGCTCTATTCTACATTCCAAGCCGCTTGAAGCTGAACGAGGAAGAGGGATTCCCTGCATTGACTTTTCAGGTGAGTCATGGTGAAGCATAACAATGTTGTGCCTAATGGGCACTTTAAAAAACACTGGCAAAATTATGTCAAGACATGGTTTAACCAACCTGCTCGAAAAACAAGGAGACGAATTGCTCGCCAAAAGAAAGCTGTCAAGATCTTTCCTCGGCCCACAGCAGGATCTCTCCGACCAATTGTCCATGGACAGACTTTAAAGTATAATATGAAATTGAGGGCTGGTAGAGGGTTTTCTCTTGAAGAGCTCAAGGCTGCTGGTATTCCAAAGAAGCTTGCCCCTACAATTGGAATAGCTGTTGATCATCGTAGAAAGAACCGTTCTCTGGAGGGCTTACAAGCCAATGTTCAAAGGCTGAAGACTTACAAGGCCAAGTTGGTTGTCTTCCCAAGACATGCCCGCAAGTTCAAGGCTGGTGATTCTGCTCCTGAGGAGTAGGCAACCGCAACCCAAGTCCAAGGACCGCTCATGCGTATTGTACGTGAGAAGACATCTGTGGAGCT

>L0478

ATTCCACAGCTTCTAATTCCAAATAAATGTCGATACTAGGCGATTCTATGGTTAATTCCAAGATATCACCGCCAAATACGTACGGAGCGGTGGTTCTCGGCGGCACCTTCGATCGTTTGCACGACGGCCATCGCCTTTTTCTTAAGGCGTCGGCGGAGCTGGCGAGGGAGCGGATTGTTATTGGAGTTTGTGACGGCCCTATGTTAACTAACAAGCAGTTTGCTGACTTGATACAGTCAATTGAGGAAAGGATGCGGAGTGTTGAAAATTACATCAAGTCTATTAAACCAGAACTTGTAGTGCAAGTTGAACCTATTATTGATCCATATGGACCTTCACTTGTTGATGAAAACTTGGAGGCT

>L0479

CCGGGCTGCAGGAATTCGAATTCTAGAATCGCTAACAAGATTTATGAAACTGAAGATGACAAACACTTGAAAATATGACCACTGCCACCGCCGCTGCAGCTACCACCAAAGCTGAAGTGACCATGACCTCTCCGACTAACTCTCAAAATCAAGCCCCAAGCTCTCCCTCACAAGATCCCAATCACAATCAACAACAAAACCCCCCACAATCAAATGATCCCGAATCCCCTAAAACCCTAACCCTAGAAATTCCGGAAGCAGAACATCCTAACCCACAAGACGATGATCCCAACCAAGATGACCCAGAAGATATCACCCCTATTTCTCCAACAATCTGCGATCCCCACGTCTCCTCCACCTCTGTTGCGACTAATTTCACCGCCTTTCGCCGCGGCGGAGGCGGCGGGAAACGTATTAAGCGAAATCATCACCAGGAAAGGAAGTATCAGAAAAGGCTCAAAATTCTTGTTGGAACCCTTAACCCCGTCCCGTATGTGCCTGTC

>L0480

CTTCCCTTTATATACTTAGTTGACCCAGCCTATGCAACTGTTTCAGAGAACTTCCATTCACAGGCAAAAGACAGGCTTCGTCCGCCTGATACCAGGATCCCCTTTGAATATTGTTATGATATGAGTCCTGATGCAAATGCCAGTCTGATACCGAGCATGAGATTAACCATGAAAGGAGGAAGCCATTTTGCTGTATCTGATCCAATAGTTGTCATATCCACTCTGAGTGAACTTGTATATTGCTTGGCTGTTGTCAAGAGTACT

>L0481

AAAGTGATGATGGTAGGTAGATTCCAAAACTAGCTACAGAAATCGCAGAAAGACGATGGAGGAAGCCAAGGGACTGGTAAAGCACATCTTCCTAGCGAAGTTCAAAGAAGGAATCCCATCCGACCAAATTGAGAAACTCATTAAGGGATATGCCAATCTCGTCAATCTCATCGAACCCATGAAGGCTTTTCAATGGGGTACAAATGTGAGCATTGAGAACCTGCACCAAGGTTTCACTCACGTCTTTGAATCTACCTTTGAAAGTACAGAGGGAGTTGCAGAGTATGTATCTCGTGCGCGCTCATGTTGAATTTGCCAATTTGTTCTTAGCTGCTGTGGAGAAA

>L0482

CCGGGGCTGCAGGAATTCGAATTCCTCAACAGAAGCCTGTTGGGTATTAACAGTGTCATGACCATCTGCAGCGAATTCTTCTTTACCTGTTGTTTCAGGAACCCCCTCAGACGGAGGAAGAGCAGCATCCTCTGCTTTCTCATTCTCAACACCTGGCTCTTCAACGCTCTCCTTTGCGTGTTCCTCTGACTTCGCTCCTTCAAAACCATTTTCGCTAGCTAGACCATCGGGTTTACTCTCATCGACCCTTAGCCGCTTGGCATCTGGCGAATCTCCATCAACCACATCATCAGCTTCCTTATCTGCATCGGGCTCAGCGCTCAGGTCAGCTTCCTGTTCGGGATTGGTCTCAGTCGGATGAGGAGCTTCAGGCTCATAATCTTCAAGCTTGCGCTTGTGATCAGAGGGTAAGGGACTAGCCGCCGG

>L0483

CCGGGCTGCAGGAATTCGACTTCTGAAATACCGAGTTTCTTGCCCTAACATATATTCACCGTACGCGCCCATGTCAAGAACCTCAAAAATAGAAAAAGAAGGCGCTTCTTCTTTCTTCTGATCCAAAATCCCTATATTCTCTACTCTGAGATTCATCAAATCTAACATAGATTCCGGCTAAATTCTCTCCAATCTTTGGATCCAAACCCAAAGATCTGCAACTTCACGCTTGCCAGTACAATTTATATGG

>L0484

CCGGGCTGCAGGGTGCATTTTCGCTGCTCACTTTCAAAATTCCTCCCTTGAGCCTTTCTCTTCTCTCTTCCTCTCTTTAAACTCCTCCGATGCCAACTCTCTGATACGATTAGTTACCCGATTCCTCGTTTCCCAAAACCAATCCACTCTAAAATGTTCTGAATCGGTTGCATTTCGCTTCCTTCAGGTTCTGAAAAATGGTGTTGAAGTAAAGCTGCAAAGGAATGCATTAAGCGTGTTGGAACATCCTACTGGGAACGAAGTGGATG

>L0485

ATTTGCTAGAGATGACACATATTTAGATCTATAACCGGTCTAAATATGCTTGGAAGAGGCAATTTTATTAGGAGATGATCTTCTTTTCTCCAGCTTTTTGAACCCTAGACGTCTTCTGTGAGT

>L0486

ATTCCATTTTCTTCCTTTCACATTCATAACCTATCATCCAACCCCCTCATCTCTCTCTCTCTCTCATTCACTTTGAGTTTGACCCGTTGCCAGCCACCGCAAACTCTCTACTCCAATGGAGACCCCATCCTGCACTCTATCTAGAGGATCTTCTCAGTGGAAATATTTTTTCAGTCTTCTCTTTTGCAGCTAAAAGCTTAAATAACCTACCTTCTTCTTTCATTTCTCTTTTTCTTTCCTCAAAAACCCTAATCGAAGCCATACACTGGCTCTAAAGGAAGAAGAAGATAGACACAGAGAGAGAAGGGGCTCACATCTCACATATGCCCTTAGCCTTTTACGGTTCAACCTGCTTTTGTTTGGTTTTCTGCTTTTCTTTCTAGACATCATAATCAAGATCTGTGCGCAAATTTTCTTTTCTTTTCACAGAAACTTTTGTTGATAGTGCAAGGCAAGTAAGAGATTGTCTCACGTTCAGATCAGGCTTTAAAAGATATGGGAACTTTCTTTAAAGCTGATTTAGAAGACATATGCAAGACTAATTCTTCAGCCTTCATTTACTTATTGATGGGTTCTTCTGCAGCGTCCCCATAATTGCTCTTTCGAATTATCTTTTACGTCTGTG

>L0487

ATTCGCTTGAACAAGGATGGCTCCAAGAGCCGGATCGAATAGCAGCTCCAAACCCAACTCCGCTACCAAGGAGATCCGTTACAGAGGCGTTAGGAAGCGCCCATGGGGCCGTTACGCGGCCGAGATCCGTGACCCCGGCAAGAAAACCCGTGTCTGGCTTGGCACTTTCGACACTGCTGAAGAGGCTGCGCGTGCTTACGATGCTGCTGCTCGTGAGTTCCGCGGCTCCAAGGCCAAGACTAATTTTCCGTCGGTTACCGAACTTAACAACGCGGCCGCTGCGGCACCTGTTGTCACCATCGCGCGCAGCCCAAGCCAAACCAGCACCGTCGAGTCTTCCTCTCCCCCGCCTCCACGCGCTGCTTCTCCTCCAGCAACTCTAGACCTCACTCTCAACATTCCCAGCTATCATCGCCACAGACATTTCTCCAATGGTGGTAGCTATCCTGGGGGCGCGTGTGTCTC

>L0488

ATTCCCCATATTGGCTGTGTTTTGTATTGGCGAGACGGAAGAGGACTACTGCTAAAAGCAAGTAGAAGCAACTATTTCGTCTCCATTTCGGCTCGAATCAGGCTCTTGAAAATAGCTTCGGCGATTATGCGATCCGTTCTTCTTCGAAATACTCTTCGCGGCGGCTCCCGGGCCCCTGCTCCTCCCAAGAGACGCTTCTCTTCCTCTGATCATAACGACGATGCTTTTGAGGTGGCTGAGGCATCAAAGTGGGAGAAGATAACATATTTGGGCATTGCAACATGCACTATCTTAGCTATTTATAATCTATCAAAGGGCCATCGCCACCATGAAGAGCCTCCTCCGTTCCCATATTTGCACATTCGTAACAAGGAGTTCCCATGGGGACCAGATGGGCTTTTTGAGAAGAAGCACCATTAATAAGTGACAAGGGCCCATTGCAAGAAGATATCTGAAGTACCCTCTTTTAGCACGTTCTTCTCTTATGCCAGCGATTGGGCAACTTTTTATGTTCTATGAATTGCTGTTGATTTTGTGACTATGCACATGGCTANGTTGTTAGTGTTTTTTTTTCTTTTACCTATGCTCTGCTTGCTAATGAATAAAATCTGAGGAAAGATGTGAGATTTCTCAGTC

>L0489

ATTCGCTAGATCCTTCAATTCTCTCCTGGGAAACACCACCATCTCAACCACCGACAGCCATGACATTCAAGCGTAGGAATGGCGGCCGTAACAAGCACGGCCGAGGACACGTCAAGTTCATCCGCTGTTCCAACTGCGGCAAGTGCTGCCCCAAGGATAAAGCAATCAAGAGATTTCTTGTGAGAAACATCGTCGAGCAAGCTGCTGTGAGGGACGTTCAGGATTCCTGCGTTTATGATGGATATGTACTTCCCAAGCTGTATGTGAAGATGCAGTACTGTGTTTCATGTGCCATTCACTCCAGAGTTGTGAGGGTTCGCTCTCGCTCTGAGCGCAGGAACCGTGAGCCGCCACAGCGCTTCCTCAGGCGCAGGGATGACTTGCCAAA

>L0490

ATTCACCCTTATTTTGCCACTCCTCTTTTATTTTCCTCTCCTCATTGTTCACCTCATGTTCTCGTGCTTAATTCATTGTTGTCCTCAATTTCCTATCTCGCTTGGTGCAAATTTTTTTTTCCCTAATCTCGGCAATCTATTGTAAAATGGTGGTACAAGATGTTGTAATCCGTGTCGATTA

>L0491

ATCCTTTTTTTTTTTTACAAAGAAAATATACCACTCTTTCTTCTATCAGCCGGACAAGTTTCATAATTTAAAGGGATATAACGTCCCCATTGAGCTCGGGCAACTAAAACCACATGTCACCATGCCTGGATATTGCGAAGAAGACTAACTAGCATCCAGTGGCTGTTACTATATCCAAAGCATTACAAGCAACTGACCTGCTAAAATTTATGACCCTCAACCCACTATTTTACAAGCACAGACTATATAATTGAACAAGTTTGATCCAGATCCAAACAGAACAGCACTATAACAAAAACATTTTCAACTTGTGCTCTCTCATAAGAGAGATTTTAATGGCTAGATTAGGCATCTACCATCTACCGATGCAGCACCAACTGGTTTACCTTCCACATATCAATAAGACCAGACAGACACCTTGTTTACCTTCGACATATCAATAAAACAAGACGGACACCTTCATTCACCTTAACTTGGGCCTTTTGGGAAGACTACTGCTAGCATTCCTTCTATGAGGTG

>L0492

ATTCATTCTCTTCTTTTGCAGGAAAGCTGGAACACCAAGACCTCTTTGGAATGCAGCATCTCAAATTCTAATGGCTGTTGGGTATATTCTTATGGCTTTGGCAATGCCTGGTTCTCTATATATTGGTTCAGTTGTTGTTGGCATCTGCTATGGAGTTCGTCTGGCTGTCACGGTCCCAACTGCCTCCGAGTTGTTTGGTCTCAAATATTATGGTCTAATTTACAACATCTTAATCCTCAACCTTCCACTTGGTTCCCTTCTCTTCTCTGGTCTGCTTGCCGGACTTTTATATGACGCTGAAGCTACTCCAACGCCAGGAGGTGGCAACACTTGTGTTGGTGCTCATTGCTATAGGCTTGTGTTCATTATTATGGCGATTGCTTGTTGCATTGGATTTGGTTTGGATGTTCTATTGGCAACTAGAACAAAGAAGCTCTATTCTAAGATACAGGCGAACAAGAGATCAAAGAAATTTTCTGCAGAATCTAACAACCAATGACAAGGTTCAAAGTGGGGGTAAGTGGATGAGAATAGCAGGATTGAAAGGCTTCTGCCTAGTGATATCCTGAAATGATCTTTTTTTTTCACCTCACAATTC

>L0493

TGGATCCCCCGGGCTGCAGGTTTTTTTCCTTTTTTTTTTTTTTAACCAAAAAATGATTGTTAAATTAAATGTAGATACAACTGAGAGTAATCACCATTCATTTGTTCTAACATTTTCTTTAGTTATCGTCTCCTATTAACCCGATATCCATATCGATAATAGGAAGTAAATCTCAATTCCTAATTCGTTCTATTGCATTAATTAGCTCCAAAATTAGCATAAACAAAAGGAATTTCTCTTTCAAGGGGAGAAAAAAAAAATCAAAAATCACCTAGCTTTCTCATTTACCACTTATCGAATCTTTGGAATCCACAACCTTCTCTGTTTTCTTCTCGGCCACATTTGATTCGGATGCCTTTTCCTTCTCCAATTCCTTCATGACCCTCTCTTCTGCATCCTTCTCCGCCTGCAAAATAGGCTCGTAATAATCAGCATGAGCCTGCATACACTTCCTTAAGGCCGCAGTCGCGTCAAAGCACTTGTCCACAACATTCTCCTCGTTAGTTTCGGCCTCTTTGACGCAATTCTCCCAATCTATAAAAGCATCTCTGCACCCACCCCCTTTCATGAACAAACAGAACCCACACTCTCCCTCCTCTTCTTCTTCCTCAGTAGGGTTCGTTGCTTGATCTGATTCTTCTGGGG

>L0494

ATTCTTTTTTTTTTTTTGAAATAGCACTTCATATAAGAATCCCCGATGCAGATCCAGGTGATTCAAATGTAACATTTTTACAAGGTTGTCTTCAGTAATAAGCCATTCAGCTCAAAAAGTTTCCAATACAAACATAAAATGGAAAGACTGCTCTAATTTTTTGCTCTTATACTTCCTAATCATAAGGGATTTGGATGTATGCTGCATCACAGGCAAAAGGGACATCAGCATACATACCAGCAAGAGCACACCGTATGCACTCCAAGACTGTAAAAAGATACGCAAATGCAATGGCTGTCCAAAAATGCATACCTACTTTACCCCAGTAGACAGCGAGTGGCATCCAACGGCTCACAGTCCCTATCACTTGGAGAGCAATCTCCAACAACATGCCCATCACTACATGAAATCTGAAAAAATGTGGCCATTCCTTCCTCCTCACAACTCCAAGATATGCAACAAAGAAATATGCCATCAGGAACCAGCTTGGCAAACTGCCAATTGCTCCAAGGAATGGGTATGTCAAAAATTCAAAGTCTTCAAAGAATGGGTGTAGGTGATATGCTGTCTC

>L0495

ATTAGAATAAAATTATAAACAAAGGACTTGCGTCTGGGGGAATCGTCGCTGAGAAAGAAAACGCGCATTTTTGTAGTGAAGGAGAGAAGATGCAGAGATCATCGAGCAGTTCTTGGGTGCCTGATGAGTCCTTTAAGGACACCAACACAAAGTACACAGACAAAGACCAGCAACAACTGCCCACTTACGATCCTCTTTCCCACGTCGCCAAGAAGGAGAGGTCTCGTCTTAGATCTGCAGAGAACGCAATCCATCTCATACCTCTTGTTCTTGTTCTCTGTGCCATCATCCTATGGATCTTCTCCAGCCCAGAGTCAAGGGTTTGAAGTTTGATGGAAAACCTATTTTAGCATCAGTTCCTAGAGTGGTTCGCCATAGAGCATGAAGCTAGAACACTTCAATTCAACAAAAGCAACTGGTTTATTACATCTCGCCAAAGGGATTAGCATACAATACAAGCAAAAGCACTACTGCTGGCCTCTTCTGTTAGACTGATTTAGTTTGTTAATATGGAAAAGCAGTTAAATTTCTTTTTGCACATTTGACCCATTGTATGTAGAAGTTTTATGATATAAAGGGATTTTTTTAAACAACCAAAAAAAAAAA

>L0496

ACTCGTTTTTTTTTTTTTTTTTTAAACATGAATAAATAGATTTATCATTATCAACTTATGCCCATAAACACAAATGCAAATTCCCTACCATGCTCTAAACTAGCACCATTCATATAAACCTAAAATTGAACCAAAACATTTAAAATGTAACACATTCAAATTATAAATCTAAACACATTATAACAAGACCATAAAAGACCTCATCATTCTACTGAATTGCCAAGGACAAATGAGAATAATTCACGAACTGGTGAGAACTCCACCAACCCAATATCCATTGCATACCCAGTAACCGGCAACTTCAAGTCTCTCAAAACACAAAATGCCTCTCCTATCCTTGCCTTTGGCACATCTTGAGCCACTGCACAATAAGGAATCCAGGAGTCAGGGCGGTACTCTTCACTGATCTCAATTCCCTCCTTCTTCAATGCATCGTACAACTGAGAATGAAATTGAAGAAGTGAAAGTGACGGCGTAGGGGCTAAAAAGAGGAAAT

>L0497

CCCGGGCTGCAGGATTCGAATTCAAGAAGAGAAAAATTCATTTCACACTCGTTTGAGAGCATCTTCAACCGGGAGAGAAAGTACATAAAACAAAAACCCTCACCACCACCAAGCCCATCGCACAACTTCAATAACACATAAAATCAATGGCCGAGAACCTCGACGTCAACGCCAAATGGGACGCCTGTCTCGATATTACGGTCCGTCGCTTCGTTTACTCTTCCTTGGCTGGGGCCTTTAGCGGTCTCCTTTTCTTCAGGACTCCGGTGACTCGTTGGGCATCAGTGGCATTTGGTGCTGGAGTGGGCATTGGATCTGCTTACACTGAGTGTACTCGTATTTTTTATGGATATCCTACGAAGTTGGCATCTCCTAAGCCGTCAAATGCTCCAGCGCATGAGACATGAGGATGACAAGGAGCAACTTGCGATTTGAAGTTACAATCAAGATTTTCTTGAAATTGAGTATGCTATTGGCTTTTTGACAATGTAGTTCAAATATTGGTGTTGATGGAAAATTAACTTTAACCTGA

>L0498

GGAACTAATAACAAGCTCAAAGAAACAACTCAATCCAAAATCTAGTTAAAATCCATCTAATAAAGCAGAACTTTCCCCAACCTAGAAACACTAAGATCACAGGAGAGCTCTAACTCAGAACTACACGTTGCCGACCACTAGGAGGCTTGCTATCAAAATAAGATGAACAAAGATCGAAGCTCACAGACATTGCTAACATAGCAAGGAGTAACCCAACGTCGCCCATCATCGTGCAGCAACTCAAAGCAGCATTAGTCTCCCACCATCCTTGACATACTCAAAGCACAGCTATTGCAGAGCTTTCTGGCACTCGATGCCATCGGGAAGACTTTCCCAACCGTAGTGTAGAAGCAAAACCCGGAGATGCGGAACAGCCAATGCAAGTGAGCCCTCTCATCACTCAAAGCTATCTTTTAGTTACAAACTAGTTGGCAGCCATTGGAAATCAAACTGTCTTCGTAAACACAAACCCCTAAATACGACAACCA

>L0499

CCGGGCTGCAGGATTCGAATTCGCCAGATTGCAACAATTGCTCTGGCAACCCACAAAAATGCAATTGGTGTAGCTGTGGGGGCTACATTAGGACACACTATCTGTACTTCCCTTGCTGTGGTGGGAGGAAGCATGCTAGCATCTAAGATCTCGCAAGGGACAGTTGCTACAATTGGAGGCTTGCTCTTCCTTGGATTTTCTTTGTCTTCTTATTTCTATCCTCCTCTATAAGGATGAAATTGTAGGCTAACCTGATTCTTTTAATAGCGTGTATTCACTTCAATGCAGTTTTCATTTTTCCTTCCTAAAGTTTTTTTTTTGTGGAAAGGTTTTCTACTAACTGGTTTTCACCAGAAAGTTCTACTTATTCTTTTGTATAATTAGCATTTATCGTCTT

>L0500

CCGGGCTGCAGGAATTCGAATTCCTCATACGGAACTCAGCTATTTCATTTCGCCTTCTGCCTAACGTTAAAATAAAAAGAAAGAAAGAAAGAAAGATAGAAAGAGAAGCAGTCTCTCCTCTCTCTCTCTTCTATCCTCTTTGTTAGGGTCTTCCGGGAAGAGCTTCAAGTGAAATATTGCAATGGCTTTCTCTACTCTCATCAGATCTACAGCTACCGCTCCTTTGATCGAGGCTTCTTGTGTCGACTTCTTCCACTCACCCTCCGATCCATTTAAGGTTTCACTTTCCTGTGATCGCCAGCTTCTTTTTCGAACGTGTCTTCCTTGTTTGTAATTTCGAAAGATTGCTTTGGTTTTCGGATTCTAGTGATGTTTTTTTTTCTAAATTTATTTTAGGACTCTCTTGCTCTCTTTGGATGGTCGAGAGAAGTAGGCTACCAGGTACTAAAATGGAAATATGTGTTTCGTAGCTTTGCTTTTTTTTTTTCTCCCCCTTTCGTTTTTTGGAAGAAGGA

>L0501

GGATCCCCCGGGCTGCAGGGTAATAATCTGTCAACGATTTGCCATCTGCATCTTTTAAAAGCAACCTTTTCATGAAGTCTGCGAGAGTGAACTGCTTGTAAAGAGCTGGTTCCTCTGGCGATAAAAGCCCTGGGAAGGGTCCATACCAGTTGTCCATGCCACTTGGGTTGAAGAAAGCAGCAACAGAAATTCATGATTCCTGCAATGAATTAGCCAACACCCTGTGCTCTACGCTTTGGTACTCTCCATTGGACATCATCTGAGAAACAACAGGAAATTAATTCAAAAAACAGCAAAGAATTTCTCTACTTTAATCCATTTGCTCAAATGGGTTTCATCTAATTACCTGAAAACATTGATAACAAGAGCACCAGGAAGAGGTTGAACATCTACCCATTGACGATCACCACCACATTTAACTTGTAAACCACCAACATGATCCCGTAGCAACACCGTCAAAACACCAGGATCTATGTGGCACTCATTCCCAAATGTGCGATTGGGTTGGGGGCAGCAGGGATAATAATTTTC

>L0502

GGGCTGCAGGTTTTTTTTTTTTTTTTTAGCAAGGTTAAAAAAGGCCAGAGCATGAGTGGGAATGCAAACACAAAGAAGAATATTGAAATACAAATCTTAGTAATAACAGTGGAAGCACTTTAATACATGTCCTATCAATCTCACACTCCCGGTGAACATGGCCTACAGAAAACCTGAACCACTTCTTGAGATATTCCAGGTGTTCCTTGGCCACCATCACCATCTACCTCAGATTCCCTTGCTATCCTTAATTTTTGCCTCCATATAAGAATCTAAGAAGTTCTTTGTTCACAATCACACTTCATACCTCTGGCAAGTATTTCTTTACCTTCCTCTGTAAGACAGATTTGTTATACCAGTTCTGACTGGAGAATACACACTGACGCTATAAACTCTACCAAAGAGTAGCTCACTAACTGTCTAACTGAAGGAGTAGCTACCCAACCATCTACCCCATAAATCACTCATTGCATTCGAAATATAAGTGCAATCAATGGAAGAGAACACGAGTAGAAAA

>L0503

ATTCAGTTTTTTTTTTTTTTTTTTTTTTTTTTACCAAACAGGTGTTAATTCATTCAGTGAACCTACTGCATGACATAACAAATTTCCACTACCAAAAAAATTTGAAGCACAGAGCGTGTTCAATAATCCATCTTTTAATCAATAATGTATTGAGATGCTGTTTAAAACTTCTAAAATCTGCTTTTCTATAACCAAATTACCCTAAATCCATTATGACTGTAACATCACCCTGAAACCGTCATTGTCAAACTCACATTCCAGTGAAGGCCTTCTTGCCCCCGCCAGAAGATCCAGCAGGAATCACCTTAAGAACATTGAATCCGACGGTCTTTGAAAGTGGTCTGCATTGCCCAATAATAACATGATCTCCTTCTTTAACACGGAAACATGGAGATATGTGTGCCGAAATATTTGAATGTCTCTTCTCATATCTTTAGTATTTCTTGATAAAATGAAGATAATTTCGGCAAACAATAATGGTCCTCATCATCTTTGCACTGTGGCAAGTCCCCGCTAAAATACGACCCCTGATAAACCAAGTTCCAGTGAAAGGGA

>L0504

GGATACCCGGGGCTGCAGGAATTCGAATTCAGCAGAGATAATTGTACATTAATTCATCATTAAAAAGATATACAATCAACGAAGAAAAGAAACAAGCACAACTACATTTATCATATCCACACCAAAAAAGAAAACGTCTTCACTTTGCCTTCTTCCTCATCGGTAAGGTTCATGCTCTCCCTGTCCATCCACAATAGCAAACAATTCACTATGTTAAAACAATCAAACTTCCTAATTGATTAATGAAAGGGTTTAATTTCCTTCTTCAAGAGCTGTTGGCATGAATTATACATGTATGCGTTTATTGATTCTTTTCACAAAGTTGAAGGTTAATTAAACCTCAAAAGTTGTGGTTGTGGCTGTTTGCCTCTTTCGTAAGGCATGAACTAATTATTAATTAAAGCAAGCATGCTTAGATTTGATAACTTACTTCCTTTGTGGTTATGAATCGAACAAATTTTTTTTTGCCCTTGCCACTGCCGTTGGGGAAACTGTGATGGGAATGGTGCTGATCTCTCGCTCCTGCATATCTCTTCACATTTTCCCTTACGAATTTGTCGTATATGAAAG

>L0505

ATTCGCTTCTTCTCTTATTTTCTCCCATTTCCTTCTCTCCTACTCTCGCGCCCTCGTTCTCTCTGTGATTTCTCTCTCGGATCCCTCTGTGAATCTCTCCAGAAAGGGATCAATAGCTGAGAAGCAGAAATGGGGTTGACATTCACGAAGCTTTTCAGCCGGCTTTTTGCCAAGAAGGAAATGCGAATTCTGATGGTGGGTCTTGATGCTGCTGGTAAGACCACAATCCTGTACAAGCTCAAGCTTGGAGAGATTGTCACCACTATTCCCACTATTGGATTTAATGTGGAGACTGTGGAATACAAAAACATCAGTTTCACTGTGTGGGATGTTGGTGGTCAGGACAAGATTCGTCCCTTGTGGAGGCACTACTTCCAAAACACCCAGGGTCTCATATTTGTGGTGGACAGCAATGACAGAGACCGTATTGTGGAGGCAAGGGATGAGTTGCACAGAATGTTGAATGAGGATGAGCTGCGAGATGCAGTGTTGCTTGTATTTGCTAATAAGCAGGATCTTCCCAATGCAATGAATGCTGCTGAAATCACTGACAAGCTTGGGCTGCACTCCCT

>L0506

ATTCCTCCATCCCTTCCTGGCGTAGAGCATGTTGGTGGAGACATGTTTGTTAGCGTTCCTAAAGGAGATGCCATTTTCATGAAGTGGATATGCCATGACTGGAGCGATGAACACTGCTTAAAATTCTTGAAGAATAGTTACAAAGCGCTGCCACCAAATGGGAAGGTGATCATAGCAGAATGCATTCTTCCGGTGGCCCCAGACAGCAGTCTTGCCACCAGGACNACTGTTCATATCGACTGCATCATGTTGGCTCATAACCCCGGTGGGAAAGAGAGAACTGAACAGGAATTTGAGGCGTTAGCCAAGGGAGCTGGATTTCAGGGTTTTCGAGTGGTATGTGAAGCGTTCAATACCCATGTGATGGAATTCTTGAAGAGCGCCTAA

>L0507

CGAATTCGTTTTTTTTTTTTTTTTTTTTTTATCCTTTGCCTTCCCTGAAATACGTAATAATTTTTCTCTCCACAGTTTTGGGTATTTGTATTCTTTGCCAGTGACAGCCTATTCTATACACAAAAACATCAGAGAAAATCAAGATTCAAGAATCATTCTGCCTGTGCTAACTAATTCATCCATAGAGAGAGCGAGAGAAAGGAATAACTGAGAGATGCTGTTGGTGTTTTGTATGCTCGAAGTTTCCTCTTCTTCTTCTTCTTCTTCCATTGGAGGATTGGAAGCGTGGATAGGAGAGGGTCCTTTCAGCTTTGCATTTGATGAGAGATTTGGGGTTTTGAGATGGTAGAACTTTGGGGAATTCATAAGCTTCTTTTTTGCGGGCTTTAACAATAGCTGGTGATGAGAGGAGTATGAGTAGCAGAGCTTAGGAGCTCACCTTCCCATGGCTGCCAATGCCGCTGCCATTAACTGCTTTCCTCGAGGGGGGGCCCGG

>L0508

AATTCCTCAATCCTTGGTGACGCAAACGCCACCAAAGAAAAATATTCAAAACCTGAAAAATCTCTTAGCAGTAAATAGCACATTCTTTAATGAAGGATTCGATTCAGCCAAGGCTATACAGCTGCTATAGATGCAGAAACCTGGTGTCCTGTCATGATGATATTATCTCCAAGAGTTTCCAGGCAAGCAATGGCAGAGCCTTTCTTTTCTCGCATGCAATGAACATAATTGTAGGACATAAAGAGGATCGGCAGATGATTACTGGTCTCCATACGGTTGCTGATGTCTACTGTTCTGACTGCGGAGAGTTGCTGGGTTGGAAGTACGAGAGAGCTTACGAGGAATCGCAGAAGTACAAGGAAGGAAAATTCGTACTCGAAAAATTCAAGATTGTCNAGGAAAACTGGTAGCAATTTGTTTCATCTTATATCAGATGGAGATTCTATTTTGATTGATTGTTTTACGTCTCATTGTTTGTACGTACAAATATCTTTCATATCGCAACTCAATAACTCGCATTGTACTGGTGGCGGCTTGTAATCCAATTATAACAAGTGAATGTTTGTCTTTTTAGTA

>L0509

ATTCGTTTTTTTTTTTTTTTTTACTATCCTCATCACCACTAACCTCCTCATTGTCATTTCGTAGTTTAGCCATGTGCTCCGCAAGCAATTTATCATCTCGTTCACTCACAGCTCTCGGTGGCTCCTTCACAACGAGCTTCCCCTTATGCTGCTCTATTGCCTCGGTGCAAGCAGCAATTGCCTTGTTAAGAACTGATATGCCTTGCTCCTTGTCTAGGGTTTGAGTGGTAAGGACATAAAGTGGAGGAGCAACCAGTTTAATTTTCACAGGACAATCTTTATTGCCTGCAGCTTCGGCTTTCCTCATTGCATCCTTAATGTGAAGAACCCCATCAAACTGGAAACATTTCATTTCAATATCAGCCCGAATCTTCAGTGGTTGTGGGGTCATTCTTCTCCTAATATTCTTCACCAAAGCTTCTTTGACTTCCTCTGACACAGCAGGCACTACCTTGGTTAC

>L0510

AATTCAATGATGACAACTACAGAAAAACCAAGAAAATAAACCCTAAGCTTTCCCTTCTTTAGTTTAATTTGGAACGAGGTTCCATGTTGATCAACTCGCTCGCAATTTTATCAACTTCTTCTTTCCCCAAACATTCAACCTTAAAGATACCCAATTCAATTATCATCGCAAACCCTTTATTCCCATTACGCTGGGCCAAAGAAATGGCAGCATCTGCGATCTTCGGCTTCAAGAAATTAAGCACATGGGTATCAAGATTTAAGGTCTTCAACACGTCCGGCAGATAAAGTTCGCGCCACATGGATAAAGGGAAGACGATTGGAACTCTGATCGAGCTACGTTGTTCTGGGGTCACAAGATCTTCAATCACGATTGCAGCGCCTTCTTCCGGTGGGAGTTTTAGTTGATATCTATATAGAATGCGAAATTCGATATTACAGGTTGGGAGGGAGGAGGATGCTGGGTTTGT

>L0511

GGGCTGCAGGAATTCGAACTTCGCTCTCTCCTTCTCGCCGGAAAATCTTTCTCCTTCCTTACTTCCTTTCCTACTCTTTCTCTATTTCTCTTTAACATTCAGTCACGGACCTCTGGAGATTGATTCCTGGAAAAAGCTTTTTCACCGTCGTTTTACGTTAGAAACCGTTTCGAAACACTATAGATTTTTTTTTATGTTTCCTTTTTTTTTTTTAGAAAAAGATGGAGGTGATTGAGCTCTGGTTGTCGTTTCTTTAGCAATAAGAAACTTTGGTTTTGGTGTTTTCTTTCATTTTGTTTTCATTTGATTTGGAGAAGAGAAACTGAATGGCGGTGGAGTATAAGTGCTGTGGAGCGGATTTCTTTATAGACGTTTTGATAATAGTACTTCTGGTGATGTTCGCTGGATTGATGTCTGGGCTTACTTTGGGGCTTATGTCTATGAGTATCGTCGACCTTGAAGTTCTTGCCCAGTCTGGGACACCGAAAGATCGCAAACACGCAAGTACTCGCTCTTTTTTTTTTTTTCTTTTTTTTTTTCGGTGAATCTCGCTTTTACCTTTTAAATTTTCTTG

>L0512

ATTCCTCCTGCCCAATTTTATTTCCCTTGCTCTCTTTTACACAACCCTCTCGATTCTAAATCCTCCTTTCGACAACCCGCAATCAACCAGAGATTATTGGACTGCAAGCTGTTGGGAGAGACAAAAAGAGGGCGAGAGTTGTAGGGTTCGATGGCCGGTGGCTCTGATGCGGCGGAAGCAACTACCTTATCCTGTGTTCAATGCGGGAAGCCTGTGCATCTTCAGTGCCCAAAGTGCATGGAATTAAAGCTTCCTCAAGAAGGTGCCGCTTTTAGCAAAGATCTGCTCTTGTGCATTGAGAAAAGAAATTCAGGGAGACATTGGCAAAGATTGATCTAGGAAGTGTCTCTCAATAATTGATAATATGTTGTCTTTCTTTTGTGTTAGTTCATGTCATACGCAGCAAGTTTAAGGTGTTTGATCAAAGCTCCAATGTTGTATTTGTACAGGTTCCCTTCAATATTAAAGAGAGAGAGAGAGAGATCNGTTTATGATTAAGAGGGAGTATTTGTCTTTCGCTGCTATTGGTATTGGTCTAAGATTTGAGTA

>L0513

ATTGTTGTTTTTACAAGCATACTGTCAAGCCAGGCTCATACACATGGAAATATCACAGACCTCCTCCACGGAGACCACGTCCTCGCAGAACCAGTTTTGTGGATAATATGACATTGTTTGATGTTTTTGAACACTTATTTGAAGAAGATGAACTGGACATCTTTGATGCTGAGGATTATTTTGATGAAGATGATATGGCTTTTTTATTAATGCAGATGGATATTGGATCAGGCGACACCTCCAGTGATGATGAAAACTATCTCTTTTAAGCTATTAGCTTTAGCCATATTGCAAACTTTTCATTCTTCTGCTGGAATTTTGAAGTTGGTGGCTTTGGACGTCTTAGTATTTTAAGCATTTTGGAATATATGAAGCTTGTAGTTTGTCTCTTATAAGCCCATGTTCTGTGTTATGTTTAGACAATGACTATTCAAAGTAAATCTATGGTGCCTGCCAGGACATTGGTTCCATCTATTTATATGTTTGTAGCATTATGGTGTGGAAGATAAACAGCCTG

>L0514

GCAGGATTCGAATTCCGGGAATTTTCCAGCGCACCCGCCAGGAAAAAAGAACCTTCTCTTTCTCTGATTTTAGGGTTTCCGGTGTATCGTCCTTTATGTTTCCTTCAAACTCTCTCCATATCAGACCTGGATTTCTCTAATCACTTCAGTTTTGTGTAATCGGTTCCTTATATACTCTAATCATCGACGAAATTAGGGTTTGTTTTTTTGAAAAATAAGAAATTATGTTCATAATCTCTTCATCCTCCCCTGTCGTTTCCGTCGTCATCTTTCAAAATCTCCGTGTTTTTGGACCCGGTTTGAATCCATTTGCTCCTTATTGCATTGCCAATCATTCCTGTGCTTCTCGCTAATTTCGCATCTTCAATAAATAAAACATGTAAATTTTTGGTTTTTTCTCCATCGTCACCTTCTAAACCTTCTCAAAATTTTAGGGTTTTTAATTG

>L0515

CCGGGCTGCAGGAATTCGAATTAACTAGTCCAGTTTTTTTTTTTTTTTTTGAGCAATCAAATCTACCATAGGCAGAAATTTGGAAGGTATGATCAGCTACATAGTAATTCAATGACAACTCGATACCTCGTGAGATTCATTGATAGCAAGTTATTTCTACCATTCATCACAGTTTGAACAAAATTTTAAAATTTAATCAATAGCAGACATTACACTTTTTGCCCATCATCCGCCTAGAAGTGTTAAAGGGCACCACAGACATCTAGTGAAAGTAACCAAGAACTTGACCTCCCACATTCCGACCAATTGCTTCTTCGTGGTCCAACACACCATCTGGAGTAGTTATCACAACATACCCATACTGACGAGTGGGAAGTATTCGCAATCTGTATGCTTCTAGATCCTTTGCCTTGATGTCTTGCCTGTACGTGAGTGCCCGACAATCTTTTATCCTACCTTG

>L0516

ATTCGTTTTTTTTTTTTTTTTTTAGCCTTGGCTTCTGCAACTGCCTTAGGATCCAAAAATTCCCCTGCATGGTATGTTGAAGCCCAGAGCAGTGGATTTTGCTTCTCATCAACAAAACTCCTCATCATGTGCCGAATAGAGTCAGTAGAAATTACTGTTGTAACTCCTAGTCTACTACCCAGCAATGCAGACAAAGTAGATTTGCCACAGCCACTAGTACCACACAATAGCACAGTTACAGATTCCTTCCTTTCTCGAATTCTGCATGCCAAAACTAAGTCTGCCCTCTGGTTAGGACCCACATACTTATATTCAGTCAGAGAATCGCAAACAACATTTAAGAAAGCCTCTCTTCTAACCATTACAGTTGTACGCCTTTTGTACAATTCAAATGAACTCCTGCTTCCATATTCCTTCTGCCCATTTCCTTTTGAAACATCAACCCCACTTACTTCCACTTCTTCAGGAAGTAGAGCATCACCTGAAGGTTCATTTCTCATCAGCTCAAAAACCCTTTGGCTAATCTTGAAAGCGTGACGCGC

>L0517

CGTTTTTTTTTTTTTTTTTACAGACACAACAAGTCGGGCAAAATTATAATTAGGTAGTAGAGGAAACGATTATAACACTGAGAGACATCATATATTCTCACATTGTCACCTG

>L0518

GGATCCCCGGGCTGCAGGTTTTTTTTTTTTTTTTTTTTTTTTTTTTTTAAAGGATATTAGATGTATTGAGAGAAGTAAATTCCCACAATGAAAGGCAGGATAACAAAAAATAATAACATAAAATATGTTCTATAGATCCACAAGCACACTGTATGAATGTACATATATACAAACACATCTGTGTAACTTGCATAAACATACATGCATTATACAAATGCCCAAGTATCTTAAGGCAGCTAGATAGCACATTAAACCACTGAACACTAGCATTACAAGGCAGGCACTCATCACATTGAATTTTGTTTCCTAATCAGCTTGTTGTGCTATAGATTTCTGATCAGAATCCAAAGCAAGACTGAGAAGAAAATTCTAATGTCTCGCTTTCATACCAGAGCAAAGAAAGCCGTAGATAGAAATGACTTCAGCATAAAAAGTGAAATGGCCAGTGCTACAACTGTTACTGAAATCCTTGCAATTGGTCTTGGTACAGAGTCAGGCACCAAATTAGTTCCTGCTTCAACACAATCCTTCCCAACCTTCCATGCTGTTTGAATAACTTTAA

>L0519

CCGGGCTGCAGGATTCGAATTCGTTTTAGATGATCCGTAGCAACTTCAGCTTCTAGTTCTTGCATCATTGAAGCATCCGATTACTGATTCTCTGGCATAGGAAAGACGCCTTTTGAGGTTTGGTGAATTGTTGAATCATCCATTTAAGTCCTATGGCGGATGAGGAACCTGTTGATCAAAAGAAGTATCTTGAGGATTCCTGCAAGCCTAAATGTGTGAAGCCTCTGATTGAATATGAGGCATGTATTAAGAGGATTGAAGGTGATGAACCTGGGCAAAAACACTGTACAGGGCAGTACTTTGACTACTTATTTTGTATTGATAGATGTGTTGCTCCGGTGCTATTCTCAAAACTGAAGTAACTGGAAGTCCCTTCGGACATCTCTTTCTCATTCTGTCAGTATGCTTTACGAACAGCTGGTTTTAGTCCAATCTTTAGTTGGCTTATTTGAAGCAGCTTTTGATTGACTTGGAATAAAGGAAGATGTCTTGAATTTTGATGTGCAACATGTTGCAGTTCATTTCATAAAAAAGGGATAATGTAGAAGAATGATACATGATAAACGA

>L0520

ATTCGTTTTTTTTTTTTTTTTTGAATAAATTTCAAACACATGTTTAGAGAAAAAGTTAAGAACTCCAAATCACTGAAAACTGAATCACTACTGGCCTTAAGCCGCACAAATAGTCATTCTGAAATTCTCTACCCAGAAACGTGACATTGACAATATCAACTAGTAGCAAAAGGGAAGGGTCAGCTTGAAGAAAAGGCGAAAGGTAAAAAAACAAGGTCGAGTTCTTTGAGCGCATATTCCCACAAAATAATTTATCCTGTGTACATCATCTAGAAAACTGGTTAAATTGAGCTTGAAAGCTGGCTAGAGTACCTTGTACTACTATCTAATTTAGATGTTGGTCGGCGACCCTTGTCAGGACCGCCAATAATTCCCCAAGTCTCAAACTGCCTCTGTCGAGCTCTTGCCCTTTCTTTTTCTTCATCAGATTTTAATGAATAACAGTATGGACAAGAAACTCCATACTCCCATTCTGGGGCTTCCATGTCAGCATCGCTCACTGGTTGCTTACATCCA

>L0521

ACTCCGGTCACTGCTTCTGTTCACCTATCCTTCCCAACAAAAAATGGGGAAGATGGTGTTACTGAGATCCACATGGCGCGCATCCTGTTTCAGGTCCTCCCGTTTCGCCGCTTTTACCCACCACCAGCCCCGTCACTACTTGCCTTCTCGATTCATCTTCACTTCCGTTGGAGCACCTTCCTATCGCAACCCTTCTGATTTCAGATCTTCTCTTACAATGGGCATTGGAAGCATACGATATTTTAGTCAGGATGTAGCTCGCATGCCTGTCATACAAGATCCTGAAATTCAAAATGTTTTCAAGGACTTGATGGCTGCAAGTTGGGACGAGCTTCATGATGCTGTCATCCACGATGTAAAGATTGTGTTGTCTAAAGACACTGATGACAAAGCTGGCCAAGAAATTTTGAAGAATGTTTTCTGTGCAGCTGAAGCTGTTGAGGAATTTGGTGGCAGGCTTATTTCTATGAAAATGGAACTTGATGACAGCATTGGATTGAGTGGTGAGGA

>L0522

CCGGGCTGCAGGTTTTTTTTGTTTTTTTTTTTTTGGCCAAAAAGTATGTATAAATTGGAATGCAGAGAGACTGTTTGATACCAAAACAGGTAACAAGTTGACCATACATTGAGCTTTGAGCAGTTTCTAACACTAACCATTCATCATTTTCATTTAAATAGACAACACCATTTCAACTCCCTAGTTGTCACCACTTTTTAGATCCAAAATCCAAAATTGCTTCATAGAGATCAAGAAAAAGAGGGAAATTGCATTGGGTAATTACTAACTCACATATTCTTCTTGCCATCAGATTGTTAACAAGTCTGCTCTATGGACTCCAATGGGCTCCACAGGCTTTGATTGTCATCATACTCTAAAAGGTAAAAACTTCAGATTACTTTCGTTTGGCATCCAGTTCAGCTTTCGCAGCATTTGCCTCCTTCGTTTTTGCCTCGCATTGAGATTCTAGATTCTTGATCTTAGTCTTCAAGTTAACAATCTCTTCCCCCAGTGATTTTATCTCCCCCGAACTAGCATTTTTCCCATCATCAAGGCTTCGGCTTTGTTTC

>L0523

CCGGGCTGCAGGATTCGAATTCCAATTGTGAAGCTGTCTTGCTGCTTTAAAGCGTCAATATTGTAATAGTAGTGCTAAGTCTTGAGATTCTTCATTTGTGATAGCTTGGGTGGGCTGCCTCAAATTGAAGTATTTCTATGACGCTTCTTCCGTGCATTCGTTGGTTCCTGACAGGTTTTCATTCCCTAGAAGTATGGGTTTAGGGTTCTTGTTTTATTGGCTTTTGTAATCTTATATGACTGGTTTCTAGATTTCTTTTCCTATAGTTTCTGTTTTACTTGCATGTCCATTTCCTGCCTTTTGAAGGTCTAGATATTAGCTTATCAAGATCCGTTTATCCATATTTGTAGAGAGCTTTAAGAAAGGTGGGAAAGCATTTCAATACAAAGAAGCTTTTCATTTTCGCACT

>L0524

CCGGGCTGCAGGAATTCGAATTCGTTTTTTTTTTTTTTTTTTTTTTTTTTTTTTTTTTTTTTTTTTTTTTTGAAAAAATTGTATTAATTTTCCAATTCCAATTATAAGGGATAAACAGGGGAAACCCAAACCCCTATTACAAAGCTCAAATCAACCCACATGATTTATAAAGCTATTCCCAACAATAAAACTTTCCTTACCACAATTATTCCCAAAACACCTCTTCTGCATTCATACTAAACTGCATACTAACCCGGAAAGGAGGAGGTCCACCTCATTTGAAATGTCCTAAATATTGACCTGCTTATTGGGT

>L0525

ATTTTCAAGCTCAGTTTGTTCATTTCACCGAACACAAAAGGAATCTGATTTTTCTTTTAAAACTGCATTTTTTTTTCCCTCCTCTACCGCAATGTCAAACGCGTCCTAACACCCACTGTTCCCCGCCTCTTCCACCATGAAATTCCGACCAACCTCTGCACCCATTTCGCTCTCTCTTTTTCTCTTCCCGATAAATTACCGCTGTCGTTCGATATCAACAGCTAACCGGTTGGCGTGCTTATGGATTCTAGAAACATCCGACGGTGAATGAACTGAAATTTGCAGACAAACTGCCCTGTTGACTTCTGACTTCACTAATGGCATCCACTGGTTCAACGCCTACTGATCCTTCTTCTTCTGCACCCGAGGACACGGCAGCCAAAGCCGTCAACAAGCGATACGAAGGCTTGGTTGCGATTCGAACTAAGGCGATAAAGGGCAAAGGAGCATGGTACTGGGCACATTTAGAGCCTATTCTGATCAGAAACCCAGACACAAGTCTCCCCAAAGCTGTCAAACTCAAGTGTACTTTATGCGACGCAGTCTTTTCAGCCTCAAACCCGTCGAGAAACGCCTCGGAGCATCTTAAA

>L0526

ATTCTTTTTTTTTAAAGAAAATATTTTGTTATTAATGAACAGGACATTAGGAAATTGTCCGTACATAAATTAGTCCCACATTGTAGGAGTTTTTAATACACACACACACACACACACACACACACACACGAGTTAAAGCTCCCCATCCCATAGCTCTTCAAGGGACTTGAATGGTCCATCTGCTGAAACCAACAGTTCTTTACGCTGTAATCTTTGTTGAGGGATCTGATTGATCTTGTTATAATCATCCTTTGACAGTGCCCAGTCAAATATCTCCAAATTTTCCTTCAGTCTCTCCTTTTTATGGCTCTTAACTACAAGAGTAACTCCCTGTTCATACACCCATCTCAGACAAACCTGAGCAACAGTCTTTCCATGAGCCTTTGCAATCTCATTTAGCACTTCATTGTCCATTACAGCATTAGTGCCCCAGCGTGTCCCTTTTGCTCCCAGGGGAGAATAAGCTGTCAAAACTATACCTTTGGCCTTGCAGAGCTCTCTGAGCTTCTTTTGTTGCCAGACAGGACTCATCTCCACTTGATTTACTGAAGGAGGGATTTTAGCC

>L0527

ATTCAACTCCACTTATGAATGCAGTCGATTACCATAACAAAGACGTTGCTCTACTTCTTATAAGACATGGAGCAGACGTGGATGTGGAAGACAAGGAAGGGTACACTGTGCTTGGTCGAGCATCTGATGATTTCAGGCCAATGCTGATTGATGCTGCCAAGGCCATGCTTGAAGGATGAGTTATGAACTAGTTTTACAAAGTTAGATGGGGAATTTAGGTAAGTAGCTGGTTTATCAATGAGGTTTTGGCAGTTGCATTCTGATGTATTTTATCTGCATGTTGTGTGTGATAGAGGAGAGTTGCAATGCAGTTGTTGGTATGTGCAGCAATGATTTTCCTGTTGCTGGTTTAATTGTCCTTGTGTTTTTTACTTGGCTGTTCCTTTAGGCCATGTTCAGTAGCTTTGGCGTTTTCT

>L0528

CCGGGCTGCAGGAATTCGAATTCGAATTCTTTTTTTTTTTTTTTTTAGAAACTCAAAAAGATACTATTATGAAGTATAAAATAAATCCAAAATATTCAACCTTTAGATATCCAAACTTAATTCAAGCATAAAAGAGGGCAGCATGAATACACATAAATTAAGAAAGCATGGCGGGAGTTGAAAAGATAAAAAGGCAATGATCCTACAATCAACAACAAAAGGTGACCAAAAAAAAATATCATTGTTTCTGTCCTCACAATGTTATTTCCCAGCAAAATCACTAATGCCAGTTCATAGCCAGCTGCAAGCCCAATCCTGAAATCAGCATTTGACAACCATGAAGCTAAGACGAGGTATTGAAAGCCTACAGGCCTTGATCAAGGAGGTAATCACCCAGCCTCTCTACGATCATGTTGCACTGTCCTGGAGTTCATGGTTCATCGTAAATACCAATAATGAGAGCTTGGCCGGTTTTCTTCACAGTGATGCCACCAGAACCCTTCTTTCCACGAATCAAAGCTCCGGGCTCTCCCTGGATCACAATGTAATTTGTGCCA

>L0529

ATTCTTTTTTTTTTTTTTTTATTAAAATAACTATTAATTTTATTCATAACAAACGTTCTTTCATTTATGGTAGAAAATTACTAAATCTTATTAAAAAGGTAGAAAAATTAAAATAAGGGTGTATCTGATTCATCCGCCCATTTATATTAATTGAAAAGACGTCTACGAATAATGAAAAATCCAGGCGAAGCCAATGCCTTGAGCCTTAAAGTTGGCACGATCCATGAATGCTTCCTTTTTCATTATTGAAACAGACTCCTCTATTCTTCATTTTGCAGTAACAAACTCTCAGATTGCTAAAAAATTGGAGATCCAAGCAGCGGTTCGCGCAATCAGAACTCTGATTCCTCCTCTCTCAGCTTTCTTTCTCACTAGTTAC

>L0530

AATTCCTCCTCTCTTTAAACGAGATCAGCCCTGTCACTGAGTAGATTGATGGGCGTTTTGGACATTGAAAGTTTCAGTCTCAATTTCTTCGGCAGTGGCTTGTGTTAGGAATGCAAAGGCTGAAGAGGAACCCTTGATAGGAAGCCTTGTTTTGTAGGAGAAGTGGGAAAAAGATAACCCGTCTTCAGTCACCTTTTCTTCTCTTTCTGATGCTGTAAGTTCTTGTATTTAAAACCAAGGGAAATATTTGTTACTTTCTTCCCATCAATTGATCAAAACCAAGGTTCATGTCTGAACTCGACGTCCAAATTCCTAAGGCCTTGGATCCATATTCACTGAGGCAAATGCTACGGATTCAGATGGTGGGGCCATGGAATATGTGCATATAGGCAAACAAACAGAGAAATGGAAGGAAAAGCCTGTCTAAACTGTCAAAGAAGGAATTAAGCTATAATAAGATTGTGAAGGACCTTAAGAAAGAGTTGTGCTGTGCTGCAACAGTAATGTTGTCCAGGATCCAGAGCTTGGCTATTCAGCTTCAAGGTGAAAAAAAGGAAAAATATTTCCACCTTCCTTGACTTATTTAGGTGGTTATGATTTTACATGCCTGA

>L0531

CAATTTCTCTCTCTCTCTCTCTCTCTCTCTCTCTCTCTCTCTCTCTCTCTCTCTCTCTCTCTCTCTCTCTCTCTCTCTCTCTCTCTCTCTCTCTGTGTGTGTGTCTATCACTGTCAATCACTATCACTCTCTCAAGAGAGCAAAATGGGTGTGAAAGAGGAATTAGAGGAGTATGCAGAGAAAGCTAAGACCCTTCCAGAAAATAAAAGAAATGAGAGCAAACTTATTATATATGGGCGTAAAA

>L0532

AATTGTTTTTTTTTTTTTTTTTGATTTCCCAACTTCATGGCATTCAAATTCTATAAAAGAACACGAGAATTAAAATAAATATAGAAATGCAGTTAACTTGAGTACAATTCCCAGCATTAGTGACAAGCAGTTGTGAGTTCACTTGAGAAAAGTACATGCCACAAACATGAAAAGATATCCTACCAATGAAGTCAGGAGTTCGAGTGTCCAAGTTGGGTGTATTAGAACTGTTGGGATCACGGGAACAAATATTGAGCCGCATGCCCACCCAGCTGCAAGAGATCCAAGGCCAATAATGAAAGCTCTGCCCAAGTTCTTTGTCTTCTCATTGAGGAAATATATGCAAGCTGCCAAAGACACTGCCACCTTCATAAGGTGCATCCACTGCACACTTAACGCTTGTAAGGGTTCTAGTGCTTCTTGAAATAGGGCTATAAAGTGTGGTCGAATTTCTAGTATTTTGCTCATGATTAGTGAGTTTCTGGGGGAGGAAAGGAGAGGAAATAGTAGGGTTAGAGAGGATAAAGGAAGCCATAAATAACAAAATTTTCAGATAAAAACCCTAATTGCAATTTTCTGACTTGGTAACAATCGTAGCTCTCCCTTTCTATGAAC

>L0533

CAATTCTTTTTTTTTTTTTTTAATCCAAAAATCCAATAGATTGCATTGAATCCTCCAAGGTAAGTTCAAGTTCATAGAGACTAGAAAAACAAAACAAACTGCCAAAGGCAATCGAGTTTAGAAAGACATTAGAAACATCACATTAAAAGTAACACCCAACATTCAACTACCATGGATAGATCACAACTACCTTTTAAGAGGTTGATTCAAAATCATCAAACAAAAATCCAAAAAAAATTAAAATAAAAAAGAAAATAGGAAACGTTTCCATTTTAGTTTTGGTGGCAAAAGGATGATCACTTTGCCATCATGACTTTCACAAATTCCTCATAATTTATCTGGCCATCACCATCAACATCTGCCTCCCTTATCATTTCATCAACTTCTTCATCAGTAAGCTTCTCGCCAAGGTTTGTCATCACATGGCGCAATTCAGCAGCAGAAATAAAACCATTCTGATCCTTGTCAAAAACTCTAAAGGCTTCTTTCAGCTCCTCCTCGGAGTCGGTGTCTTTCATCTTCCTAGCCATTAGATTTAGAAACTCAGGAAAATCAATAGTTCCATTCCCATCAGCATCAACCTCATTAATCATGTCCTGAAGCTCTGCCTCGGTAGGATTCTGTCCCAGTGATCTCATCACAGT

>L0534

ATTCTTTTTTTTTTTTTTTTTAAATAATAAATTTTCCTAAAAAAATATATAATCAAAATTAACATATAATAATTCAACTCATCTGTGCTAAGAATATATTCCTGCGTTTACTCGTCAATCCCCCAGCATTTTCACTTGCTTATAAAAATATTCTCCCATTCCATCAAAATACACGATATAAGACTAAATGAAACAGGAAAAGACTGAAAGAAGTGGAGATTATAGATACTCCCAATAAGTTACTCTAAAGAAGAGAGCCTCCCACCAAGACTCCAACGAATATGGATCTTGATTTCATTCTCGTCCTTGATGCTTTGCATCTCCTACTGTTCACAGAAGATTGTATTACCAAGGATGTAAACATCTTACTTCCTGCTCCTCTTTCTGGTACTTTCTTTTCCGCGTTTTCCTGTGAAACTGACACCGGAATTGACTCTGGAAGTGTGGGCAGAGTGAACAGGCACATGATGCCCAGGAACCAAAGCAGGGAACCCAACAGCAGGGATTCCAACAAGAGCACAGCAGACTCCTGCTTGGCTGAGGTAACACCAAATGCATTGCCCTTCTGCAAACTAAGACGTAGAGCTTGATTCTCTGCCATAAAACACTGAAGCAACTGCCCCAGTCTTCTGCATTCCCCTTCCAAATACCTACTCTTAATTTCC

>L0535

AATTCTTTTTTTTTTTTTTCAACAAAATTCATACTATCTCATATCACCATGTTTCTACAAGGTCCAAATGTTAATTCGAAATCAGCAAAACCACGAACATAAACTACAAAAAAATGTCAACTTGAAAGAACTTGTTCCAGCTGACCTAAAAAGTACTGAACATAACCAATTAATAAAAGCACCACCACCAAACTGGAATTGAACTCCATGGGAAACACTTTACAGCCTTCTTCCCCTTCTACCACCCTTTCTACGGGTGCTGTCCGTTGGAATTGGTGTCACATCCTCTATGCGACCAATTTTCATTCCAGAGCGAGCAAGTGCCCTTAGGGCAGACTGCGCACCAGGACCAGGTGTCTTGGTCTTGTNGCCTCCAGTTGCACGGAGCTTGATATGAAGAGCAGTAATGCCAAGTTCCTTGCATCTCTGAGAAACATCTTGCGCTGCAAGCATAGCAGCATATGGTGAAGATTCATCCCTGTCAGCTTTAACCTTCATCCCACCTGTGATGCGAACCCAGGTTTCTCTCCC

>L0536

ATTTTTTTTTTTTTTTTTGTTATAAGCGTATAAATACCATTCATCTCAAACATTATCTTACAACACATCACGAGAAAGATTATCTCTTAATTTTACAATCTTATGCAAAGGTAAAAGATTAGCCCAAACTTCCTGCAGGTAGAGTAAAAAAGATATACATCTCAACGCCGCTTGGGATCAACCCCAACAGACTTGGGAGTCTCAGCGCGGGGCTGCTCGTGTGCAAAAGCGTCTCTACGAATAGGATCAGGCGTATATCGAAGACCGCTATCAAACTCTTGAACATGCGCAATTTTCCTGAAGAAAGACTTCTTCAAGAATCTATCAGCCTCATTCACCACTATCTCAGGCTCCTCCACCTCTGGTATCGTTTCCCTTGTCTTCTTCTTCACGCGTACTGTCGGGGAGTGCATTATCTGTTGCTTCGCGGTGTACAGACCCAGAGATACCGATAGAGCTATCATTCCTATCACCACATAAACGGGCACAAACTCTCCCTTTATTGTCTTTTTAGTACGTTTTTCTCGCTGAGGATTGCCGAAATCAGCTGTTGGAGCATACGCCATCATTTTTGGAGAGCTTGAAGTTGCATACGTTGCAGTTCCACTCAACCGACTCACCATTGATTTCCAACAACTCATTGATCTCAAAGCCATGATTGGA

>L0537

TGGAGCCAATTCCTCTTGCCCCTCCGAGGGCGAGACAACCCTCCTTCCTCATTCGGCGTGACTTCCAAGTTTGAGGATAACGATGAGGGTTTGGTAGGATTTCCCTCTGTGGCTGTGTACAAGTGCATGGGTTGGCGTGAATCTGGGACTCTTGGAGGCGTTACCGATGTCATTCACTTGGGTATCCAATCTCTCAACAATCGGTGCAAAAGAGCGCTGCCCTTGGTGTTCCCCTGGCTCCAGATCTAATCCTCTTCATGGCTCTGTGTTTGTTTCTTTCTCAGGGTGATATTCATATTGTTTTTCGGTCCAAGTTTGAGGATAACGATGAGGGTTTGGTAGGATTTCCCTCTGTGGCTGTGTACAAGTGCATGGGTTGGCGTGAATCTGGGACTCTTGGAGGCGTTACCGATGTCATTCACTTGGGTATCCAATCTCTCAACAATCGGTGCAAAAGAGCGCTGCCCTTGGTGTTCCCCTGGCTCCAGATCTAATCCTCTTCATGGCTCTGTGTTTGTTTCTTTCTCAGGGTGATATTCATATTGTTTTTCGGTCCTATGAGTTTCTTT

>L0538

AGAAAAAAGAGAACTCATCTTTGGCTGGTGAATTGCCGCACCTCTTCCTTTTCTCTCCGTGACGCCATCTCCATTTGAGGTCTGAAGTTGCTTGCATGAAGTATTTTTATTGTAGCCATTCACTAAGATAGCATAGCATTGTAGATCTGTTTCAGTGCATCAGAGACTGAAATTGACTCACAAAGTTTGACAAAAATGACAGAGATATTCGGTTACACAACCTGCAGACAGTTATCTCAGATGTTTTTGGCCATTATCTTCTTTCATGGTTCTGAATACATTCTAGCAGTTGCCTTTCATGGGAAATCAAATGTTACACTCAAGTCACTTCTGATCAGCAAGAATTATCTTCTGGCGATGATATTATCATTGCTTGAATACTTCATTGAAATTTCTTTATTTCCTGCATTGAAGGAATATTGGTGGGTGACTAACTTAGGCCTTGCACTTGTCTTAATTGGGGAACTCATTCGGAAAATAGCAATTATAACAGCTGGGTGGGCCTTCACACATGTTATCAAGATTTATCATGAGGAGCATCACAAGCTAATTACTCATGGAGTCTATAGTTTTGTTCGTCATC

>L0539

ATTCCAAACCATCTCCTATGCCAGCTTTCATCGATATTTTCAGATGTCCACATATTCATCATATTTCTTCGTCTCTAATGTCCACCTTACTTTGAGAATTAAGAGACACATAGCCCAGTCCTTCCCTTGAATTCCTTCCAATCTTCTCGCGGAATTCGATTTTGCCTTCTTCCTTGGCGTTTCTGCCAATTCCTCTGCCTTTATACCATCCATATCCTGCAAGCAACGCCGCGCCGAAACCCTCCACTGGTGCATCCTCGAACTCCTCAAGGCCGCAATCCTTTGATTGGATTATGAGATCGAGGTTCTTCATTCTCCTATGCGGCCATTCGTTCTCTTTGGGAGATATGTTGGTAAAATTTGATGTTTGTTGCTGAATCATAATATGAATAAGTTATTGGATTTTGGAAGGAAAGCCCTTTTCTATGTGAGGGTTCTTTCTGGGTATGAGGAGCGTAGAATCCGAAATTATAGATTACAG

>L0540

ATCGTCCTCTCTTTTTTACGAATGGAGTAAGTATAGCTAGGCCTCTCCTCTCATAATTATTTATATCTGCGTCATGCTAGCTAAAACCAAAGGAGGAGTAGAAGAAGACAACTATCGAGAAGCGTTAATGGGAAGAAGAAGAAGAATACAGAGTGATTAAGATTAATTAATTACCTTGAGAAGAGAGTGCCACAGTCACATCTGTACTCCCTAGTGCCGCAGGTCTTAGAATGAGCTTTCCAATCAGATTGAACAGCGTACCTCTTAGAGCACTTCTCACACTTCCATTTCTTCTCTCCATGTTTTCTAGAGTAGTGCTTTTTGATGCCGGTAAGGTCACCAAGAGCTCTAGAAGGGTCATGGTGGACGCAAGTGGGCTCAGGGCACAAATAGACCTTCCTCTTCACTTCTTTTGTAGTCTTCTGCTTTAGCTTCCAAGGCAGGTTGGTTTCTCTTTTTCTTCTGTGGCGGCGGAGCCGGAGCTGAAGATGAAGTTGGTGTTGAGGAATGGTGT

>L0541

CTGGAGCCAATTCCAAACTTTCCTTTCACTTGCCAATGCTGTGGGAACCGGTGCAACTTTCTTGATTCTTGCTGGTGTGGCTGTCGCTGCAATTGTGTTTGTGCTTCTGTTTGTACCAGAGACCAAAGGGCTCACATTCGTTGAAGTAGAGCAGATATGGAAGGAAAGAGCTTGGGGCAGTAGTTATAACACCGAGAGCCTTCTTGAGCAAGGAAGTGAAACGTCGTAAGCTTGTGCATAGCTCAATAATGTCAGCATCATATATAAAGGTTTTGAAGACAGTTTGTAAGTATCATAGTTTTCTGTGCCTATAAATGTGATGTGCTTGGATATATTTTTGAAATTTGTCCAGCACAGTCTTCAATATTCTAGGGTCCAAAACTCTGGACATAGAAAGTTATGACCGTTAAATGTGTGCAAGTGAGAGAGCCAATTTGACCTGTATTTTAGTGGTCTTTTTCCTTTAGCACCCTAGGAAAGGCTCTGTTCTCTCTATTTAGTATGTGGGAAGTTTGTATTGATTTCAAGAACATGACTAGTGGGGTGGTAATGTAAGATAATGTGTGATG

>L0542

TGGAGCCATTTTTTTTTTTTTTAAGTAAGGATTCTCTGTTTTTCCATATGAAAATTGTATTTTTCATCTGTAAAATTTTACACACGAAAGAAGCACATCACATGCTTTCGGTATCCCTATTTGATTTTCTACTCTCTATACACTAGAAAAAGAAACCAAAATTGACAATTCCTTCTTTTTCCTTTTCCCTGATTATGAACGGAACCGCAAAGGAGGAACCCAAGATGAATAGCTTAGAGACCGAGGGGCATTAAAGCAAGTGCAGCCCAATTCATTTCTTCCAAAATCAGGCAACAGCTTGAAGGCAATTTCGATTCTCATCTCTGTCACTAAGTCTTTCTTTGGTGTATGAAAGAAGACGTTGAAGGAAAAGGAAAAGAGGAAAATTTCCAATCAATCGCGTATAGCGTGGTCATCCCAGCAGAGAAGCATGGAGATGCAACGTCCAAAGATGTAGAAGCGGGTCTTCTGCTGTTTTACCATTAGAAAGCACTTCCTTCGAAGACTGTTTTTCCTGTGAGCTTTTCTCTTGCCCGCGGCGGCGTGCGGCGGTGGAAGGTGGTANGAAATTGCCATGGTGGGTTGGCGGACAATGGAAGGAAAACTGTAAACTAGAGAACTAGT

>L0543

CAATTCTTTTTTTTTTTCTAACACAACTTTACGAATGTATGCTACAATTATATGATCCATACAAGAAAATGCCCGTCTCTTATGTCCAAGAGATTCACAACAAAATGCCTGAATAATATGTCAAATCACAATATAACAGCTTAATTCAACAGACTACAAATGAACTGGCCATCTGATACGGTAATCTTCTCTGCTTTGACAACTGCCTCGAAAGGGAGAGGAATGAAACTTCGTTTCGGCCCATAAAAATGGGGGAAGCTATTGGTACCATCTACAACTTCATATATCAATCCATCCCCAAGCTTCTTAGCGTCAATTTGAAGCAAATACAACTCCAAATTAGTGTTCAAGAAGAAGTTTTGCAATGTTGACTTAACCTGGTGGAGCTTGCTGAGGTGGATAAAACCGGACGATTTATCAAGATCTCCACCAAAAATAGATCTATCTTTCTGCAAGACTTCCCACTCCTTAGCCGTACTAATCCTGTACACGTACTCTTCGCCTTCACTCCCACTCGCTTCCTCTTTTGAATCCATGTCCTCGGCTTTGTGAAAACGCCGCTGCCCGCGGGCCACCGCAACTACCAAATCT

>L0544

CAATTCCGTCTTTTCACTATGATAAATGATCTCCCAACAATATTTGAGGTTGTGACGGGAACTGTAAAGAAGCAGGTGAAGGAGAAGTCATCAGTCTCAAATCATAGCAGTACCAAGTCCAAGTCAAGCTCAAAAGGGCGTGGATCTGAATCTGGCAAATATTCAAAGGGGCAGCCAAGGGAGGACGATGAAGGACTGGAAGAAGAAGATGAGGAAGAGCATGGGGATACTTTGTGTGGGGCATGTGGAGAGAACTATGCTGCAGATGAGTTCTGGATTTGTTGTGATATATGTGAAAAGTGGTTCCATGGCAAGTGTGTGAAGATCACCCCGGCTAGGGCCGAGCACATTAAGCAATACAAATGCCCATCTTGCAGCAACAAGAGAGCACGACCTTGATTTTTGTGGGTCGGGTGAGCCCTCACCTCTGTGCTTCACTCGTGTTCATGTCATCAAACTATTTAGATAGTCAGGTTCTCTATGTTGTGCTATG

>L0545

GAGCCAATTCCAGCCGCCTCTTTCTCGCCCCCTCCCGTGCTGCAATCTACCTCTCGTCGAAGTCGAGTCGACCACGACGGTCAGTCTTCCTTGTCGTTCAAAAAGATGAGATTTCTACAACTGCTCAGCCAGTGCCAATACCTTGCCTACACTAGGAGAGAAGCCTTGGTCTATGCGAATGGTGGAGGAACTCTGAGTCTATGGAGTATAGGGTTTAAGCAGTTATTCTCTTCGTACAGAGAGGAGCCAGAGCTCAAGGACTTCATTGATTACTTGGATTCTCTCAAGAACTATGAGAAATCAGGCGTTCCCAAGGACGCGGGTACAGATTCAGACGATGGCTTTGATCTTGGTCGCATGCGGCGGCTTATGGACCGTCTGGGGAATCCGCAGTCGAAGTATAAGGCCATCCACATTGCCGGGACCAAAGGAAAAGGATCAACTGCCGCCTTTCTCTCTAACATCTTACGGGCAGAAGGCTATTCAGTTGGTTGTTACTCTAGCCCCCATATTATAACTATAAGGGAGCGGATTGCAATGG

>L0546

ATTCCTCCCATCAAAGACAATTGTTTGGACTATAGTTATGGGTTCAACAACAATCTTCAGTGCTTCCTCTACTGTCCCTGCAATGAAGGTTTTAGACTGGAAGAACTACGTTGGTTTAAAGCCAACCGTTAATGATTTGAGGTTCAGTGGTGTTAAGACCTCAGTCAAAGTGTGTCCCAAGCGTGTTTTGATCGTCAGGGCTAGTGAAACGCAGGGTGGGCCAATGAAGAAGTTGGGGCTCAGTGATGCAGAATGTGAAGCCGCCGTTGTGGCGGGAAATGTGCCTGAGGCGCCTCCTGTGCCTCCTAAGCCCGCTGCACCAGCTGGAACTCCTGTGGTTCCTTTGCTTCCACTCAGCAGGCGTCCTCGTCGTAATCGCAAGTCACCCACATTGAGAGCATCATTCCGGGAAACTAATTTATCACCTTCAAATTTTGTCTATCCACTTTTTATTCATGAAGGTGAGGAGGACACACCTATTGGAGCAATGCCTGGATGTTACAGGCTTGGATGGAGACATGGACTTGTGGAAGAGGTGGCAAAGGCTCGGGATGTTGGTGTGAAAACCATTGTG

>L0547

GGATCCCCCGGGCTGCAGGTATTTTTTAAATCAAACTGTTATAATTTCATTTCNGGGCCAACTTAGCCCACAAATTTTCAGAAACTAAAATCAGGGGAGCCCCGCTCAACCCTGTTACCTCACTTACAAACTGTTTAAATACCATACAAATCTTCATTTATTAATCTTAAAAATTTAAATTAACACAACCTCTACCAAGGTCCACACTATTTCTAACACATGCGGAGTTCTAGATTTTAAATTTAGAAAAAGATAGTAAAACAATTAAATAATTGACGTTAAACCTGCGAGGAAGAAAACAGGTTGTTCTGTAAAATAACTCCTCCTGTGGCAGTACCGTCGACTATTGTGGCTGCTAGATGCAGGAAAATAGTTGATCTCGATCACCCGAAGTTTATCTGTGTCTCAAAAATTCCAAAAATTTATGAGGAAGCCTCTGGAAGTTGAACCATCTCCATAGCTCACCGAGCCACCGCCGGCAACACCGCGCACGGTGGCCGCCCGCCGGTCGCCGGCAACATTTTCCGAATCTGATCATACCCCCATGCTCCTCTCCTTTTCCTCAGTCCATATGTGGTCTCGGATCGTCGATCCAACGGTCGGATAGTC

>L0548

ATCCCTGAAATTGTTTCCATCTCAAGCTTTTGGCAATGGAGAACATTCTTGGTCTTCTTAGGATTCGGGTGAGAAGAGGGGTAAATCTAGCAGTTCGTGATCATGGTGGTAGCGATCCTTATGTGGTCGTCACCTCGGGACACCAGAAACTGAAGACTCGAACGGAGAAAAATAACTCCAACCCTGAATGGAATGATGAATTAACTCTTTCCATCACAGATTTGAATGTTCCAATAAAATTAGAAGTTTTTGACAAAGACACATTCACTGAGGATGACAAGATGGGCGATGCAGAAATTGACATCAAACCATATATAGAGATTCTTAAGATGGGCTTGCAAAATCTCCCAAATGGCTGTGTAGTAAAAAAAGTTCCGCCAAAAGTGGACAACTGCCTTGCTGACGAGAGCCATATCATTTGGAACAATGGCGAAATCACCCAGGACATGCATCTCAGATTGAGAAATGTAGAGTGTGGTGAAGTGGAGGTTCAAATTAGGTGGA

>L0549

TGGAGCCATTTTTTTTTGATTATCTGACACATTTCATTATGTCCTAACATAAAAGAAACATCACAACAAAAATAATGCTTCCAGTTCATCATATATCATAAGGTGACAAACAAACTATGAAAAAAAATAGGTTGAAATCCACTCTCAGACTATAGCATTGGCAGCACTGGCAATTCTTGGCCACAAATCAGCACACTCTTTCCCAATGGGCCCAGTAATAGCAGATCCTTTCATCTCCCCCTTGGGATTCACAATGACACCAGCATTGTCTTCAAAATACATGTAGACTCCATCCTTTCTGCGCCACGGTTTGCGCTGTCTGACAATGACGGCTGGCATGACCTTCTTCCTGAGATCGGGCTTCCCCTTCTTCACGGTGGCCATCACCATGTCTCCAACACAAGCTGACGGCAAACGGTTGAGGCGGCCCTTGATTCCTTTCACCGAAATGATGT

>L0550

TGAGTTTTTTTTTTTTTTTTTGAGCATGCTTGAAAACATAATGTAAAAATAACTTTCCATATTCCTGAGATGGTATCATTTGTTAATTTGTTAATCAGTCAACAGCAATTGAACAGATTGTCTAAGGATATTTCCAAACTCGAAAACTCACATCGAAATGGCAGCCAGATACCAGTTTCATGTTCATTAAATAGTACAAATAGCATTCAAGCCGTTCTACCTTATCCACACGATAAATTAGTATTATATACTCTTAGAGATTGTGATCACTTCACAACTTCAAACTTGGAACGAAGGTTGGAAATCATTACTGGGCACTCCCCCTTTTCATCATATTCAGCAAATTCACCTCCAGACAAATCAATATCTTCGTATTTAGTCCCTGCAGCAGACTCAGCTTTCCACCCACTACCAAATACAAAACCCTCAGGTTCATAGCCTCTGCAGTCAAACATCATCAATGGGGCATATTTCCCTGTATCACTGATTTCCTGGGTAAGCAGCTTACCCTTGCCGGGCACCAT

>L0551

GGGCTGCAGGAATTCGAATTCCAAGAATTAAGAAAAGAAAAAAAAAATGAAAAGATAGATGGAAAAGAAATTGCAAGAACCAGAAAAGCAATCCCAAACCGGCTATATCAATCTCAAGAATCTTCCTCCTCTAGATCCGCGCACCACCTCCTACTCCCAGCAGGTTAGGAAAATCAAAACAGTTGCCAGGTATGTTCCTAAAGCTGTTAAGCCAAGCCCCCAAGAAGAGGGAACCAAGGCAGGGAAATTGTTTGATCATGAGGCAAACACAGAAGCAGATTTGTTAGCTGATAGAAATCTAGTATATAATAAGAATAATCCCAGAGATGATGAAGATGATGCTTGTAATTGCTGTATCAAGCACATGATAGACTCTAATAACACCATCTCCAAGACCCAAACGCAAAGCAAGGTGGTTTCTGAGACCAGCATGGATGATAAAGCCTTCCATAATAACTCAGGCAAACAAAAAAATGCATCAGCTAAATCACAATTGCATGAGATTTGTGC

>L0552

ATTCAAAATATTCCCACATCTCTCCCATAAAATACAGAACTGCAACCTCCTATTGCGACACTTCTCCCCAGTCTCCATGGATACCCTCTCCAGCTCTGTTTCAACCCTCAAAGTCCCTGCCCTTTACTCAGCACCTCGTGAATTTTATCACTTCAAAACTCTCTCAAAGTCTCATAAACTTTCCCCTCATCTCACTACCAAACACAACTCCATCTCCAACAAAACTACCGTCCAGTCCTTTTATCTCAAGACTCTTAATTCTCCACAATCCTACACTTCTGAAAGTTCATCTCTCAAGTCATCTCCCAATATCCACCTAAACCCAGCAAGCGGCTATGCTGCGGCACTTCTAGACGTAGCCCAATGCAACAATTCCCTTGAAACCGTACAAAGGGATGTCGAAAGATTGTTGAAGCTGCTCCANAGTGAGCAAGCACAGGCTGTTTTGGCCAATCCGCTTGTGGGTGAGAAAAAATAAGGACAATTGGTGAATGAATTGGCAAAGAAAGGGAAAATGAATAGGCTTTTGGTG

>L0553

ATTCCCCATCACCTGAGTCTACTGTCTCAAAATCTAGCTGGGGAAAAAACCCAGAAGAAGAGTAGAAAACAGGAAACGAATATCCCCATCTCTACGCACTTCTTCTTAGAAATGGCGTACGTAGACCACGCCTTCTCCATAACAGACGACGACATTATGATGGAGACATCATACGTTGTCAACAATCGACCACCAATCAAGGAGATTGCTCTTGCCGTCTCTCTTCTCGTTTTCGGCGTCGTCGGTATCGTTTTGGGCATTTTTATGGCTTCGAACAAAATCGGCGGTGACCGTGCTCATGGGCTTTTCTTTGCGATTCTGGGAGTGGTGCTGTTTATACCTGGATTTTATTATACTCGGATTGCTTACTATGCTTATAAGGGATACAAAGGCTTCTCTTTCGCCAACATACCTCCCGTCTAGTTGATGATAAGCTGAAGCTTCAATTTGCCCCAATTTCTTGTGTACATATCTTTCTCAAGTTGTGGTTCTTTCTTTAGTGTACTTGCGGTT

>L0554

ATTCCTTGTAAAAAATATGATTCCTTCTCATAAAGAGAATAGACACAGTACGCTGTCGTATATAGATTCCTTTCTTCCCCTGCAAATAAAATCCTAATAAGCCCAATTCCAGAATTGTCCACCGTATTCCCGGACTGCGAGGTTAGGGTTTCGCTCATATTTCCTTTAAACTAGTAGATCTTGTAAATTCGGATCGACAGGACCTTGTTTTGAGCACTGAAGATGGCGACTGAACAGAAAGCTGCAACCGAGGACGTGAAGATGGACCTCTTCGAGGATGACGATGAATTTGAAGAGTTTGAGATCAATGAAGAGTGGGAGGTCAAGGTGGAAGGAAAAGAGGTCACACAGCAGTGGGAGGATGATTGGGATGATGATGATGTCAATGATGACTTCTCGCTGCAGCTGAGGAGGGAACTTGAGAAGAACACTGAAAAAACCTAATTTCCTAATTTGTTGTTGTCTTTCAGTGCCATCTTCTGTAATGGAGTATCAATTAGTTGTTTGAGCTTAATATGGTTTTACCATATGCTTAGTATGTTAGACGTNTTAATTTTTGTACAACTTTGATTTTATGTGCTAGGGTTTGGTACTTGAATGGATTGCCTCCATGGACCTTCCATTTGTGTAGTATG

>L0555

AATTCCTCTCCCTCACGTCTTCCTTCCTCTCCCTTTCTTTTTGCTCTGGTGCCAGCGAGGTTCCAAGTTAGTACTCTTCCCTCTAGCGACCACCAGCAATGCCTTGAGTTGGTGAATGGCAAGCTACAACGGCGTGAGGAAGGAAGAACCGCAAGTTGGAGCAGAAACTTCAAATGGTTCCTTTGGCCGTTGTCGGCGGCACTAGGTTACTAGGTGAATGGGCTTTCTTTTCCAACAAGAATTGCAGAAAATGGAGCTTTATAAAGAAATAAATGAACAGAGAGAGAGAGATGGGTTTGGTAGCAGCTTTCCCACCAATTTCCCGGTGATCCAACCATCAGATCAGAGATCCAAGACCAATGTTGTTTTCAAATGAAACTTTCGAGATGGGATTCATCCTGTTCCGATTAGACACCATTTGAAAAAGAAGATTATCAATAATTCAGATCAGTAGGTGAGATTTTCGAGTTTTTTTCATGTTCGTAAATTAAATATAATTATATAATAATAATTAATAATTCACCATTTTGGAGTAAAAAAAAAAAAAAAA

>L0556

AATTCCTCAGATTCAGCCGCCCCAAATCGGCACTGGTTATGGTGGTGGCGGTGGGCTGCTCATAGAGTGCTCTTCAAGGCCTTAGAAGAAGCAACAACTCATCAAGACAAGGCCAAGAAAGAGTCAGCTATGGGACGTAAGGCGCAAACTCACTGTTTATCATCCTTTATCTCCTCTTCCTCCTGATTAGACTCTCCTTTTCTCCGATGACGCTGATGGTAACGGTGGTTCTGCTACTGAGGCTGCTTTGCCTTCTGCTTCTTCCTTCCAGGCATCAATTACCAGTGGGTAGTTGGCAATTTGTTGTTTTATCATGTTGTTCGCTTCTATTTGAGATCTTGAGTTGGATATTTTCACTTTATGTGCTTTTAGTTCTGTATTTTTATTGTTAAAACTTAAAATTGAACTATAGATGTACCGAGGTGGAGCTTTTGTTTATGCCCATTATCTGTTTGATGATAGA

>L0557

AATTCTAATTAGTAAGCAAAGACCGAAATTGCATTGGTCGGCAATCGAGTACTCTCAGTTACGCTGATCTACTCTCACGCTCTCAGTGCTTCCATCCAGAAACAAAGTGGATTAGTATAGAATTCTATACAGCTAAGCATATGGCGGTGTGTCACCGACAGATCACTTCAAGCACCAAACGAGCTGTATAAGAGTTGATGACATCTGCCGCAAAAACCCAAAAGAGAGATTTCAATGGAGAAATTTGCTGAAGATCTGAATAATCGATTTCTCAGCATGCCTGAAGCCATCAAAGGAAGCAAATCATCATCTGCAGGAAATGAAGATGGAGAAGAAGCCAAGTCTGAATCCATGGAGAAAGTAAGTGTAGAAGAAAAGGCCATCAACTACGCCAGAGGTCCGACACGCCCGACTCCACCCAAAGGTCCACCTCCGCAGACTGCTTGAACAATTTTATTTTGTTGTTCTTTATTTTTAATTTTCAGAAAACTATTTCGTTGAATTTAAATTTTTATTTCACTGGAGAGAGAGAGAGAGAGAGAGAGAGAGCGACATATATAATAATTATAATTGTCTGC

>L0558

AATTCCCCGATGTGTACAAAACTGCTAGGTTAGGTCTCTTGGCCTTGGATTTACGTTGCACAGTTAATTCCTTACTGTCACAACACTCCTTTCTGCAATGGCCGATGAGTCCAATAGAACTGCGTTTTTAGAGATTCAGAGTCGCATGATCGAAACTACTGCCAAATTGAAGCAGGTGCAAAACCAGATGCGAACCAAGGAGGGAGAGAAGAAGCGTGCTTACTTAACATTGGAGGAGTTGCGCCAACTGCCTGACGATACAAATACTTACAAATCTATAGGAAGAACGTTTGTCTTGGAGCCAAAGTCTGTTTTAATGAGTGAACAAGAACAGAAGCTAAAGGATAGTGAGAATGCAATAGCCTCACTGCAGACCTCAAAGGAATACTTGGAAAAGCAAATGGCCGAGGTAGAGAACAACTTGAGGGAGCTTTTGCACCAAGATCCAGGTCTTGCTCGTCAGATAATGTCCATGAGTGTAATGTAAGTTTGCTGCTTTCCCCAACTTAGTACAATGTGTTTTACATTTGAAGGGAAGTTGTAAGTTATGATAAGAAATGTGTTGAAATTTCTGGAATGAATGCTCATGTAATTGCTTTTCTGGATAGTTTAAATTGTCTGTAATTT

>L0559

CAATTCATAAATTCTTCTCTTAAAAGAGAAATACTTGTCCATATTGTATAGCTGCCCACACAGTACCAAGCAAAAAGGTTCAACAGGCTTAACAGAAGCCCTAAATTATCAAATCAAACTAACATTTCTCATATCTCATAACAAAAATGGAAAGGAAAACAATACCTATTTATCTAGGACTCCTCATGCTGCTCCATGGAAACTTGGCCTTCTCCACCGTGTAGTCACGAACACCACCACGACCTCTCCCAGTTCCCTTCTCATACCCAAGCCTTTCTTCCTTCTTCCCGTCAAGCCAATGCTTTTTCCCTTCGCCATCTTCCATAACACTAGACCTCCTCTGCTTGTTCTTTCTCTCCTTCTCCTCCTCAATAACCCTCAATGGAAACAGATCCGGAGATATCGAAAGCGGGTTTCTCAAAGTCACATAGGCCTTTTTGTAGTCAGGTTTGGCGATTAGTAATCCGCGTATTTTCTTCTTCTTGCCTTCCATGTTGAGAGTACGGACCTTGTCGACTTCAAACCCGTAAAGGGA

>L0560

GGAGCCATTTTTTTTTTCAAATAAAATTCCAGAAAAATATTTATTTAAACGAGGATCATCCCACATCAGCAATATTTACATATCATAAAGAGTAGAAAGTTTGATGGTCATTACTACAATCACCATGATCGTAATTAGACGATCTCGGACTTGGCCTCGGCCTCTTCTGGGTCTTCGAAGTTGGGATCCTTCACGTCCACGGCTTCCCTCTGGATTCCGATCTCAGCCTGAGAATACCCATCGCCAGCCCAAGTGTACTTGCCGCCGTGACCTCCCTTCTTGGGAGATCCATTCATGCCAGTGGCCGATTTCTTATCACTCCTGACATTGGACTTGGCATTATGATTGGTTTTGAAACTCTTACCAGCGTTCTTCATCCAAACCCATAGTAGTTAAAACTTTGGGTGTTCAATTCAGTGCACTTCTTTTGAATTACAGCACTACTCAGATTTAGTGCGACTGATTATTTTTCTTCTTCTGATGTTCTTGGATAAAGCACTTAATTACAAAGGTTTTCTAATGCCAAAAAAAAAAAAAAAAAAAAAAAAAAAAAAAAAAAAAAAAAAAAAAAAAAAAAAAAAAAAA

>L0561

AATTCGTTTTTTTTTTTTTTTTTCCACAGCAGCAATTATCCTGCTTCCTTAATAAAACACCATTGTCAATACAAAACAATAAAACAAAGACAATACCAAACACCTATACGTCCCATAATATATTAGAACTTAAAAGACAGTAATTGCAAAACCTTTTTTTTGTTTTTTTGTTTTTTTCTTGGACACTACTTGCCTCGGGTGAACAAACATCAATAATCTTGCACTAACAAGATCATCACAACCCATAGATTTGCAGGAAGGCTGCTGTANAAACTAATGTTGTTACCAGAAAGGAAACTGGTTGGTGCATTGTAACATTTCAGAGGAGTCCTCTTCTTCTCTTGTAGTCACTAACAGTCAAAGTCAAAGCATGCCTCCTCCCATCATTCTCAGGGACCCGGCATCCAACAGTTGCCTCTATCCGTTGTTTCCTTGCCATGAACCCTGCCTGAGCTCCCTGCGCTCCACCAAATTTCTGAAGTGCAGTTAATGAGAATGGAAGCTCAAAATCATGAAGTGTATTTGACTCTGTGCCTTGATCCACAGGAGCAGCTGTATCAACCAACTTCCCTGCATCAGCTGCTGCTTCCAGGGCTGCCGCTGTAGCAGCCATGCCAGTCTCGGCTATAACCCCAGCTGCTGC

>L0562

AATTCCGAAAGAGCGAGAGAAAACAAAGAAATTGAAAAATTTGAAAAACGGCACAGAAGACTGCTGCTATCATTTCACTCTCTCTAGCTTCGGCTCAGTCTCTGTCTCGAAGCGGACACCGTAACGGTTCGCCTTCCCATTTCGGCGACACCGGAGATCTTAGCGAATCATCGGCGGTTTCTTGATCTCTAGACCTTTTTCTCCCTGTTTGGCTGTTTCAGTGCTCTGGACTAACTCTCTTTTTTGTTTTCCTGTTTTTTCCATATCAATAATATTATCATCAAGAGGTACTAATGTGTTCTTCATTTTCTTCTATTCATCGTTTCAGTAGTGAAATTGATTGGATTGTTGCTTAGTGTTTAACATTTTCCCTGCCATTAGGTAGTTAATGGCATTCTATTTTCTATTTGAGAATAATTAGTTCTATTTTCGTGTTTTTTAGCCTAATTGTCAAGGTTTTGATGTGGTAGTCTGTGTATTTTGTGCCATTTGTGTTTGGTTAAATGCGATAACGGCGCGCTCACTGGCCGTCG

>L0563

TGGAGCCAATTCTTTTTTTTTTTTTTTTGTTAAACTCGTAGAGATTCAATCTTCAAGAGAAAAATGATGTACATAACAATCCAGCAAAAACATGTTTCTAAAGGACTAGCAGATAATCTGAAAACCTTACATTCATTAGCCATACTTATATCACCACAAGTCTCTCTCTATTTTTCATACATGGGAACCGATGACAAGAGAGAAGCAAAAGAAGTATTTCTATTTTGCAGATGGTCCAACAAAACAACGCCATGTCTCTCTTAGCATGCCACACCTAATCTCTGCTGATGCATAATTGGATCTGCTATGAAGAGGAGCTAGCCATGGATTCCAAAGCTCAGTCTATGCACCTTTGCGATTGGCGTCCTCCTCGTCTTCCTCCTCAATAGGGACGTTAACGTTAACTACGGCCTCGCCTTCACCATTTCCACCAGCTTCCTTATCATCTGGATATCTAACCACCACCACCGGACACACGCAATGATGCACACAGTAATCACTCACACTACCCAACCTCCCATCGCTTCCTCGTTTGGCTGCTCCAAACCCTCTGCTCCCCATAATCACTGCACTCAACCCTAACCTCTCCACCTCCAAACACAGTCTCTCCTTCATGTCATGGTCCTTTACAATGTGGATCTTATAAGGGATCTGTGCTTCCCTCAGCGGCTTCGCGAGATCCCCCGCTTTGGACGCCGCAAAGGCGTCGAAATCATCCTCGAGGGGGGGCC

>L0564

AATTCAACACAATTACAATCTCATTTCTTTGCAAATCCTACCTTCCAACAGATAATACACCCCATGAAATAATACAAATACATACCCTCTTAAAAGGGTTGTAACCTCCAATATTATACAAAAAAAAAAAAAATCACTATTCTGAAACCTTGGTAAGGCTTACTTCAGAAAACTTGCATAATTTCAATGGCAAGCTACTACCTATACATGTTTGGTCTAATATCATCATCAATAAAGGAGAAGTGATTTGAATTAGGCATTCCAATGGTTCTACCCGGAACAGATGCTGACAATGCGTCTCCAACGGGCATCCGTGGCATCAGGTCAGGATAGGAGGTATTGATAGGATAATTGGGATAGGCGTATCTACTTGAAGAAGGTAAACCTGTGTCCAAGTAAGGATCAAAATAAGGCTTCTGAGTTGGGATTCTTGATCCACCACCAACATACTGGCTGGCAAAAACTTGATCCTCCGTGGACAAGCTTGACCTATTCTCCAAGTAAGGGCTTGAGACAATTTGATTGCTGGAAGCTGTTGGAACTGCTGTATTGGGAAAATAGTTTCCCAGACTTAGCCGAGGACTTGAACTAAGGAACTCTG

>L0565

AACTGGAGCCAATTCCTAAGTATTTTATTCTCTTCAGTTCTGGCTTTAATCTCTCTCTCTCTCTATCAAAAGATAAGTTTTTTCCGATAATTTCTTCGAAACCCACTGTTTGAAATCCTCATCTTTGAGCAAATCATTGATGTTTTGGGGTGAATTTTGTTCCTAGGAGTGAAAATCCATGGATTTTAAGGCCTTGAGAATCCAGATCATGGGTTGGCCAATTGCCAGGCGCTTGCTTTTACGTGTATTTATGCTTGCCTCGGCTCTTTCTGTTATTCCTTTCATCCAAATCCTTTCTGGGTCTGATCCTGTATTGCTCGATTCTGTGAACTTCCATGAGTGTGATTTGCCGTTTATGATTACGGGCACGAATTTGTTTCAGAATCGGTTTTTGAAACCCATTTGGAACTCATTTGAGTGCAAGGAAGATGTGAATTTGACTACTGATGTTGTTAGACAGCTTATGGTTAAGCAACTGTTGGATTATAGTGCAAAAGCTCTATATGTCGGTGAAGGTTCGGCATCAGCGGTTTATGCACTGCGAGAGT

>L0566

TGGAGCCAATTCTTTTTTTTTTTTTTACTTGCAAAACCATAATCTTGATTCACACACCAAGGGCACATGTAAACGCTATTCAAGCTGTGAACAATAGGTAGTTAGGTAAGGTGCAAATACACATTACACTATTGATTGATTTCAGAAAGTAGCTTTTCAAGTGAATCATTTGTAAGAGGGATATATAAACAATCTTGGCCATCACTACGCCTTCTGGTCTTCACCAACTCGTGATCCTTGAATTCTGTCAAATGGGAGTTAAGGGTAACCTGGCTACTCACAAGGAAGCGCTCTCTGGAGATTGTATATAGATTATCAAAAGGCATGCCTTCTTCTTCAGGGTGCGACCGTTGATATTAGAGAGGTGCATTGACATGGTCAACAGTGGCAATGGTACGGATATTCGAACAAGAAGCAATTCGAGCAAGATACTGTTGGCTTTCAGAATCTCTTAACCCAGGCCCATCAATGTTATGAATGACAACACATATTAAAGAATCATTTTCCTCTGCCTCTGATTTATCCAAAAATG

>L0567

CTGGAGCCATTTTTTTTTTTTTTTTTAACAGAATTCAAAAATCATACCATACAATCCGAACAATTTGAACAAACAACTCTTAGAAAACTGGATTAACAAAGAAAGACATCTCCAACCACAAACTGCAAATCTCTCTTTTGTATGTACAAATTGCATGCACAAACCTTCCCAAAACTCAGAAAGAAATTAAATTGCCATTTCTTGAATATAACAAAAAGGAGAACAGGAGCAGCATCAGGGTGGTTTGGCAGGTGATGACGAGGGCATGATGGCTTCCCAGAAGACAAAAAGGTAACCAAACTGAAACAGAGAACTTGCCATTTCTTGAAGAAACCTGCGGCTTTCATGGAAGAAGACTACCAAAAGGAACAGGAGCTGCAAAAGAGCGGTTTGGGAGGTGATGAGAGTTGTTCTGCTCAATCACAATTCCAATTGCTTCAGCTACATCCATGACAAACTCCAAGGCCAGCCTCGTTAGTTCTATGCAAATCTCCAATCTATGGATCACCATTTCTGCAACTCAACTCAAGCTTATTAGCCCTTACTTTTTTTCTTTTTTATTTTTAAACTCTCTCTTCTTATGTTCTCCACGAGAGAGAGAGAGAACTAGTCTCGAGGGGGGGCCCGGTAC

>L0568

ATCGTGCAGGTTTGTTCGAGTGGCAAATCTTCAGAACCATCGACTTTGAGTGGCAAATCATCCATAAAGCACATCTTCAGAATCATCGACTTTGGGTGGCAAATCGCCCCAATTTTGTTTCGATTTGTTTGATTGCTGTAGAATTAGAAGAACTATACGCCTGCAAAAAATTCAGGGGAGGATGAGGAGATCAAAATAAATTTCAAATTTGTTATAGCTTTGGAGCATCTTGGAGCAAATGAGCAATGGAGCTTTTGGATCACTGCTCACATGGAGGATTGCTCACTTGTGAAAGTGCTATATTTCTCTGGTAGCAGAAATGAGTGGCCGCTTTTCTCGCACAATCTATGTTGGCAACTTGCCATCAGATATAAGAGAAGGGGAAGTTGAAGATCTGTTTTACAAGTATGGCCGTATACTAGATATCGAGTTGAAGATTCCACCTCGTCCTCCTTGTTATTGTTTTGTGGAGTTTGAGAATGCTCGGGATGCAGAAGATGCAATCAAGGGTCGAGATGGCTATAACTTTGATGGCTGTCGTTTGAGGGTTGAGCTTGCCCATGGCGGT

>L0569

AATTATTCATTTTGCACGCACAGGACATCTGCTGATTTTGCAGAAAGGGTCCTGGGAAGCTTCTCAAAATTTAGAAGGCTCCTTATGCAAGACGTAGGGGTGTGTTAATGAGCTACCCCTTTTCTATCCTCCCGATATTCTAGATGGCTTAACCTATTTCTGTTCTCTCTTTGTTAGCTTATACCTTTATTATTATTGTTATTATTATTTTCATGTTCCTTTAATTCAGAAGTTCAAAACTTGAAGCCACTGTAAATTGGGCGTTGTATTCGTATTGCTCTTTTAATTTTCAAAGTTTTAGCAGTCTTTGAAGGTTGAAGAAAAATTGTGGGCAGCGTGGTGGAATTTCTTGTCTAGGCTTTCCAACTTCGGTTGTAGCCTCAAACATGCTTTTAATATTGTACTGTGGATTTCATCTAAGCTTTGAGGTCTCTCTCTAAAAAAGAGAGAGAGAGAGAACTAGT

>L0570

TGGAGCCAATTCTTTTTTTTTTTAAAATAGCAAAATCATTTTTTTTTAAATAGTATTTAAAAGTAGCATTTACTACCTTCAATTTCTAACCGTCTCTAAATCTTCCGTTGATGGGTCACTAAATCATAAAAAGAAGCTGAGATAAGATAATTTTACAGGAACATTTCAGTAAAAGTCGCTTATATATTTGATTTGAGATCTTCTGAATACACAGCAGAATTCCACGACGGGTTCCGCGGAAGAAATGTGTCAGAAACGTAGGAGCAGGTGGGGATGATCGACATGGAGTGGGACTTGGCGTGATTAAGAGCAGCAACACAAAGGTGAGAAGCCATACCCAAGCCTCTCTTTGAACGGGGGACATAGGTGTGCACTAAATCCATCACTTTCCCATTGTTTCTGAGCACGTACTCAATGTAAGCCTTCTTGTCCTCCGTTTCAAACCTGCGTTCGTTTTCGTTCCACACAATCTTTGGCGATTCTGTTTCTGCTGTTGCTGTTGTCGATGCCATCTTTCTTCCTCTCCGATCTCTATTTTATTTTTGTTTGTCTATTAAAGACCATTGCACCTAGT

>L0571

AATTATTCATTGAAGATCGTCAAGGTGAAAAGGTTAATTAGATTGGATATGCAGTTGATGAGTGATCAAATCCTGATTAAGATAAGGAGATAACATCTCCATGATTGTATCTTTGTTATCAAAACACCACCACTCACTGATGTAATCTGCCGTCTCATCCGGATTGTCTGTTCATGAAAATCTAGATCTCTATGTCCATCTCAGTTTCTGGCAACTTATACTACATTTCTGCCGTTATCTTTTCCAATGATATGAGGCTTTAGTCTCTCCGGTGGATGGTTTCATCGCTCCTCCGTCTCTGGTTGGGAAAATTTCAGGCTGTGTTTCCGTGGAAGTGGAGCCATAGGTGATTATTGATGTCTTCGGATTGCTAACTTGAAACCAAAGTGATTGCTGTCTGTACTCCAAAATAGTTGGGGCTCTTTTGCAGTAATCAAGATCTTGGCCTCAAAAAAAAAAAAAAAAACTAGT

>L0572

CAATTCGTTTTTTTTTTTTTTTTTTTTTTTTTCAAAACAATAATCATTAAACATATTCGAACAGAACCACTGTACTTCATATAAATGTGCACGCACATGTACATTGATTGTAAACCAAGTAACAACCAACAAATCAATCAGTTACCTTACCAGTTTTTTCAGATACATGCTTCACTGAACGCATGTCTTCACCATCAACCAATTTGTCACTCTCCCCCTCCTTACCCCTCCTATCTCTCTCCTTTTCCCTTTCCCTCTCTCTATCCCTCTCCCTATCAGTTCCACCTTCTCTCCCCTCCTTCCGACTACTTGATTCCCTATGCTTTCCCTCACTCTTTCCGCTGCTCTTTTTCACCAACTCCTCACTCTCACCATATGCTCCAGCACTCTCATCTCTCCTCTTACTCCTGGATTCACCCGACTTCTCCTTCAACTTCTTCGTCCCCTCCCCTCTATCATCATCCCCGCCATTCCACCTATCATTCACGCCATCCTCAACTCTCTCCTTAACCCGCTTTGATGACGAGTATTCCTCTAGGTACTTCCCATTTCCAGAGCCAAACGAGTCTTTATTGTCCTT

>L0573

AGCTGGAGCCAATTCTTTTTTTTTTTTTTAAAGCACAAACTTAACAAACAGCACAAACTTAACAAACAGGTTACAACCAGACATCAGAGTTGGTGATTGTTTTGGGTAGGGAACAAGGCAAGTAAAATAACAATTGTTTGCAAAGTAGTAATCCGGAACACCCATGTCCAACATAAAAGGGGAATTAGGAATACACTTTCCTGGCCTTTCACTCAAAGGCCCATTGGTGTTCTCTCTCTCT

>L0574

ATTCATAAAGAAAAGATAAAAAAGAACAAATATATGAAGCAAACTTAAGTAGTGTAGATATCTGTACCTGAGGCGTTGGTGGGTAAGCGAGGGCAGGCGAGAGAGGAAGAGCTTGGATTCTCTCTAAGTCTTGTGGGATTCTCTCCCCGAAGTTGCAGTTGCTGTTGCTGCTGCTGGTGCGGCAAGGCATCGAGTTGTTGTTGCCTCTCTCGGCGTTTAGAAGCAGGAACCCATGTGCTTTTAACATGGGTTTGGCACTCAAATCCTCGGCTCTTGCAACAAGTCCTACACCGCATGTATATACAATCTTTCTTGGCTTGGTTGCCGCAATCTTGGCAGCTCATGCCTCCACTGCTGGACCTCATCATCACGAAGGCTGATCTCGACGAAGGCTCATCAGAGACGTTGATTGAGCTCCTGCTGGTGCCTACCCCTAACCCAGCAGCTGAAGAGTAAAGATCTTGCTGTGGATTTTGGTGGCGTTGGTGGAGAAATTCTTGCTGTTGCCATAGCTCAAAACCCCTGTAAGGAATATCTTCATTTTTGTACAAGAACCAGCTTTCTTGTGGGATTTCAGTTGGTGGGTTGTTATTTTGGTCGTCTTGGTTAAT

>L0575

CGGCATCAATAAACCAATAAAACCCGTAGCCGAAAAAAAAAAACTGAGAGAAAATTCAGGTACAATGAATCAAGCAGCGGAAAGAATAAAATCGTAGAAGTTTCTGGAAAAAGCCAAAGAACAAACCCTAATCGAGATTCTCTAAGGAGAGAGGGGGGCGGGGGAAAGAATACACTTATGTACCTGAGTGGTGGTAGTGGGAAGCTTGTAAGGTTCAGGTAAGTCAGGAACCTTGGAGGAAGGGAATACCTGCACGTGTACAGAGAGCAGAGCCATCATCGTTATTGCGCATATGGCACACGTTAGCAACTTTCGCAAAACCACCACCGCCCTCCATCTCCGCTTCTGCATTTTCCACTTTCCGCCGCTCTCAAAACAAGACACTACAAAAAAAAAAAAAAAA

>L0576

AATTCAATGGCTTCTTCTTCTTCTTCTTCTTCTTCTTCTATCAATCCTCAACGGAAGAAGTATGATGCGTTTATAAGTTTTAGAGGTGCAGACATCCGAGATGGTTTTCTTAGTCATTTGTACGAAGCTTTGAATCGAAAACAAATTTGTACCTTCAAGGATGAAAACCTTGACAGAGGAGAAGAGATCTCACCAGCCCTCTTGAAAACAATTGAAAACTGGATGGTTTCCATAGTTATTTTCTCTGAAAACTATGCCTTTTCTCCATGGTGTTTGGATGAGCTTGTGAAGATTCTTGAATGCCAGGAAACTACAGGACAAATAGTATTACCAGTCTTTTACCAAGTAGATCCTACAGACGTTCAAGAATTGACAGGAAGATTTGGGGATGCACTTGCTCAATATAAAGAAAAATTCAAAGAAAACGTGGAGAGTTGGAGCCGTGCTTTGAAGGGAACGGCCAACATTTCAGGGTGGGATTCAAGAGCTATTAAGTAAGACACTTATTTTTCAATGTCCTTTTTAACTTATATAATTGATTTTTTCTTTTCACTT

>L0577

AATTCGAGAGAACTAGTCTCGGTTTTTTTTTTTTTTTTTGCAATATTGTAATTGCTGCATAAACTGCATAGATATTACTGATCCTTAATACAATGATGAAGAGGGGAAATACAAGAGCACGATTCATACAGGTTGCTAATCTACAATACAAAAAGCAAATATGTCTTAGAGCGAGGATGCAGAGTACTGAATAAATTAAAGATCCGTGCTAGAGGAAAAGTTTTGTAGCTCAATCATGTGGAGTTTATTCAGTTCCAGCAATCTTCTTCTTCAATGCTTCAATATCTGTTGCCAGTTCTTTTCTGCTTGACTTGAAGAGAAGGTATCTGTAAACAAACCATCCAGTATATCCAAGCCCAACCAACTCCATGATCTTGGGCAGCAAAGGTACTGAGTTGATGGCTCCCACAAGGATCGATGATAGCCAAACAGCAACTATGGCCCCACCTCCATAGAGAATTACCGTAGACTTGTTTTCAAGTGCATCCCACTTTGCCTTCAAGTCTGTGAAAAGCTCACCAGCATCAAGAGAAGTGGATTCTTCTGATGAAGAGGCCCTAATCTGAATGAGAGGGAACCTGCGGGACTCTGACACTTGTTTTATGGAAGGGCGGAATGATTGGGATGATATG

>L0578

AATTCCTTCTTTCGATATACAGTTTTTTTTTCTCCCCTTTCTCTTTCTGGAATGGAAGATTCTTTCTCTCTCTAGATTCTCTCTTCTTTTGTTCTCCTCCATTCATACTCTAGTTTTTGATCGATTCTCATAATTGTAATTCAATCACATCTCAATTCAAAACCCTAGCTGCACCTGCCTCTCTTTCTCTGTCCTCGCATCTACTTTAACAGGTAGTGGCAATGTCAACTCCAGCAAGAAAGCGATTGATGAGAGATTTTAAGAGGTTGCAGCAAGATCCGCCTGCTGGGATAAGTGGAGCACCGCAAGACAACAACATAATGCTTTGGAATGCTGTGATTTTTGGTCCTGATGATACTCCTTGGGATGGAGGCACGTTCAAGTTGACTTTACAGTTTACTGAGGATTATCCAAACAAACCACCAACAGTGCGATTTGTATCTCGAATGTTCCATCCAAATATTTACGCAGATGGAAGTATCTGTTTGGATATTTTACAAAATCAGTGGAGTCCTATATATGATGTGGCTGCTATACTTACATCTATACAGTCATTGCTCTGTGACCCCAACCCCAATTCTCCTGCAAATTCAGAAGCAGCTCGCATGTTTAGTGAAAACAAGCGTGAGTACAACCGAAGAGTTAGGGAAATTGTGGAGCAGAGCTGGACGGC

>L0579

AATTCAGAGTGTTGGTTATTATTCGATTGATTTGCACCTGGTTGCTGGTGATGCGACTCCCATCAGTGCGAATATCTTTCCGATTCAAGCTTGAAGGCTTCAACCTTCCAGCAGCAAGCCGTGCAACCAATCTTCGACGGACAATTAAGCTCGCTCACAAGACAATCGACTGTCGACATCCTTCAATCTCCGGCGACCCTCAGCGCCGGCTGCAACCATATTGATTTTTAAGGAACGACAATTTGAGTTTGAGGTGATTTCGATCATAAGCTCATATAAATTTCACCAAATTTATCGGCAAATAAGATTTGGCTTTGAATTGATTTGTTCTTTTATGTTAATTCTGAGTGAAGTCTTATCACCAGGTATTGGAATGGAATTGAATTACTTGGATTATAATTAGAATTCAGAAGCTTTTCATTTGGTAAATAAATCCCTTTAGAAATGGTGAATTTGTGGAATTGAGTAATGATAAGCCAACATTTCCAAAAAAAAAAAAAAAAAAAAAAAAAAAAAAAAAAAAAAAAAAAAAAAAAAAAAAAAA

>L0580

TGGATCCCCCGGGCTGCAGGTAACTGTATGAACTCAGACAAGGAGTCGCACCAAAACGGCAACACCACATCCATTTTCTCTCCCATCCAATTCTTCTGTTGTCTCCCACCCTTTTTGGGTGATCCGCTGCATCGCCGGTCCATCCAAAACAAATGGATTCGTCGCCGATCAACAGATTGCCCCAGGATACGCTCCACCAGATCTTCTCCTCTCTTCCGCTTCGGCAAATCATGATCTGCCGGTCAGTCTGCAAGTTCTTTAATCAGATTTTGACTTCGCCTTCTTTCTTGGACCTTATCTCCACCCAACCCCCTCTTCGTTTCCTTGCTCTACGGCCCCCTCACCACCATCACCACCACCACCACCACCACCGTCATCATAGCCACCTGTCGCTTCTGCCCTCCCTTCACGTTTACGACCCTGACCAAAGCCAGTGGCTCAGATTTAATCTAAGCTTTTTACCTTTCAGGTCTCCTCACCCTGTGGCATCAGCTGGTGGACTCGTTTATTTATGGGGTGACTCGCCCAACTCGACCGAGTCAAGCAAGTCGCTCGTGGTTTTGCACCCGTTGACTGGCCGATTCAAGGTGTTACCTC

>L0581

ATTCCCACTATGCAGCTTCTAATCGTTCTCCAATAGTCAGGATCATTAATCCTATCTCTCAACCAAGGAGAATAATCTTGAAGCCTATACTCCTTGTAAACCCTACCAGGAACAGCCACACCACCACCTTGGCTTGTGACAACAAACCCAAAAATAGTCAATCCCATTAAGGTTGCAATAAGGAACAACATGACCACGAGGTAGACCCAAAGTGCCCATGCTACATGAAAACAAGCTCCAATGAAGCCAGCTAGTGAAACTATGAGCACAACAAAACCCACAACCAGAAGTGGGGTTTGGAGGAAACTTTCACAGGTTGTGCTGCTTCTTGCAATCCATAAGCCTCCACCAATGATAGGTATGGAAGCTAAGAGAGTGAAAAGATTCAAGAACCCAATAACTGTGTTGCTAAATCTATACATTTCTTTAATCTAATTTCTGTTTGTTGCACTTGCACCTAGCTAGTTCAATTCCCTCTCTCCTTCTAAACACTTGTATGAGTATCTCTCCTTTTCTCTTTGTATTCCTGCAGCTTCTTTTCTTGTTATGGTGTCTTGTTCAGCTTTTCTCTTTTAATGGGTGGCAATGCTTTTCTTTATTATATATGTTTCTAAAAG

>L0582

ATTCCTTCAACTTTGGCATAAGCAATGGGTTGTTTCTCATCCACAGAGAACCAACTTTCTGGCTCTGCTGAAGCAAATTTCTCCACATCCACAACTAGCATGTTGGGCTGAACGAGTGTCTGCTCAAAAGCAACTGACAGAAGCTTGTCTAGCCATCCAGCAATTCCTGGAAGTTCAGTAACATCAAGCCCATGAGTGAAGATGGGTTTGACAGTCATTTGGAAATAAGGTGGCTCAGCAAAGCATACCCTCAAACGGCCAAGGAAAGGCCAGTGGCGAAGGAACTTCACTCCAATCAAGACCTTCCCTTCAACATGCATGGCTGTCATATGCAACTTTGCCCACATTCCAAATCCCAGCCTTTTCCTTAACTTGACAGCAAGTACTGCACTCATATCATCTGCCGTGCAAAAGTTCATTCCCAATTCCAATGCCAAGTGGTCATCACTGGAACATTGGCGAAGAACCCTCATTTCTGTGAACATTGGCGGGTTTCTTCCCAAATACATGTGTTGAACCACAGCCTTTTTAGCGGTCCATGGCTTATACTTGTCCAAGAACCAAGGTATAATTGGGAGTAGAATCTTCTGCGATACAATATNCTCCATACAAATGGGCCATATCTTTCCACAGCATGGTTCAACCAACGAACAGTTTCAGAATCAGAGAGCACT

>L0583

ATTCAGAGAAAGCAAGAGTGAGAGCAAGAGAATCAGAATTTGAAACCCTAAATTGCTTCGAAAGAAGTTACTCTATTTTGCACAAGAAAGAGAGGGAGACAAATTCCGTTTTCCTTCGACTTTGTGGCGTGAGGTTTTCTGGATTTCTGTCTTTGAAGCCATAGCTTTGTTCTTGGTAGCGCTAGAAGGGGAAGAACCTTTTAAGCGACGACGTTTCAAAGATGATAATGAATGAAAAAATGGTTGCGGAAGCAGAGATTATCTGTCGACCTAGCATTCCTCCGGTTCTTGACGTGCAGTATCACCTGCAGAACTTGAACGTAGATGTGACAGTTCCTTCGACTCCCATCTTTGAGTCAATCTCCACTGATATCTCGCGATTTGAATCAGTGCGTTTTTTTTTTTTGTTCCTTAATCTTCCTTGATTTCTTGAATTTTCTCGGTTTATTTTATTTTTATGTTGTTTATGGCGATTTACTGATTCGTGAGTAAAGATTGCTCGACTGATTACTGGGTTTGATGATT

>L0584

AATTCAATTATTAAATTTTGCACTCAGAACCCATCAAAGACCACCAACGCCAAAGGCCTGTCTCCCCGAGGGAACAAGACCGTTTCTTGCCCATTGCAAACGTTAGCAGGATCATGAAAAAGGCTCTCCCTGAAAACGCTACAATCTCCAAAGACGCTAAATAGACTGTTCAGGAATGCGTCTCTGAGTTCATCAGCTTCGTTACAGGTGATGCTTCCGATAAGTCCCGTAGAGAAGAGGAAGACCATCAGCGGCGATGATTTCCTCTGGGCCTTGATCACTATGGGCTTTCAAGACTAGGTCGACCCTTTGAAGGTTTGCTTGCACAGATTTAGAGAAATGGACGGGGAGGAGACTGCTGTAGCGCGTAAGATAGACGCTCGTCGAAATGGCAGTGGCTTTACTGTCGAGGGCTATGGCGGTGGGTACCACAGCCACGTATACAGGACCGGCTCTGGGTTTAATAATCAGATGGGAGGAGTCGGGTTGTGGTAAGGTAGTTGGTCGGGCTTTAGTGGG

>L0585

ATTCGTTTTTTTTTTTTTTTTTTTCTAATACTTTTACATGTTAAATTAGACCATTGACATGGCCAAACGTCCTAATACAATTGATGGTTACATAATTGAATGCACAACAATTGCGTCGAAACTGGATTCTAATATTTTCATGGGTGCCAATAACTACATGATGCCCCCTTCTTGCAATACCCGCTTTCATAAAATTTACATACTCGCTGCCCTTTACCCTGAGGCTTGGATGAACCTCCATTTCCAAAACCATATGTTGGCTGCCTGTTCCATGAACATCTATCCCTACCAAAACCTGATTCTCGACCTTGAAAATAGTAACTACGGTGGTCTCTTGGACTAAAATATCTGTTGTCCCCGCCATTTCTTGGTTGGCTTCCCCAACTGCCTACACCCACATTACCCGTAGAAACAGCTGGAGTTGAATTAGTTCGTTGCTGAACTGACCCATGACCAGTTCCCCATGCCATGCTTGCATTTCCTTGAGGTGCTCCCCTATTCAAGTTTGCACGTCGAGTGTTGGAACCAGTTTCCATTTGGCTAACTGTTGATGAAGTAGGAGCCCGGATATCTGAACCTTCATCTCCTTGGTTAACAGTTGAAGATGCCAGAGGTTCTATTTCTTGACCGTGTTTTCCG

>L0586

ATTCCCAATGTTGTGCGTAAGAGCAGTTTGTTTGGAGATGCTGTTGGGTTTTCTACTCCTAGTTCTAATTTGAGGGATTTACATGGTCGATCAACACCAAGCAGTTTGGTAGATTATGCAGATGACAACCAATATACTAACCCTACCCAGCTTGGGCTTTCAAATGATCACCAACCAAACAACTCAGAGCGATTAACTCTTGACTCTCTTGTTGTTCAGTACCTCAAGCACCAGCATCGCCAGTGCCCAGCTCCTATAACAACTCTTCCCCCACTCTCTCTCTTGCACCCACATGTTTGTCCTGAACCAAAAAGGAGCCTTGACGCTCCATCAAATGTAACTGCTAGGCTTGGTACACGTGAGTTCAGAAGTATATATGGTGGTGTTCATGGAAATCGCAGGGATCGCCAATTTGTATACAGCAGATTCAGGCTGTTGAGAACTTGCAGGGATGATGCTGATGCTCTTTTGACATGCATAACTTTTCTTGGTGATTCATCTCATATTGCAGTTGGCAGTCATACTGGAGAGCTTAAGATTTTTGACTCTAAAAGCAACAGTGTG

>L0587

AATTCCTCAGTTTTTTTTTTTTTTTTTTTTTTTAGACAATGAAATATTATTAATTCCATATGAACAAACAAGCTCCTCTAATCCCTCCTCATCTTCATAGAGCATTACACATGTCGTTTTGATCATTTAATAAAAAATCAAGAGGTTGAGGGCCGACACATGTGGATTCCCTATCAAGGGACAACTCCACCACTGCCATCACATTACCCGAAACCTATCTTTCCCCCAAAAATCACCATCACTGTTCCTCTGCGGTCGGGGACACCTCGGTCCCATCCTCCACATAATCATCACCATTGATCTGATCAAGCACCAGAACTAATCCCATGGCAAACGCCCCATCAAAGCCAGGCCTTAAGCGGAGAGAAAAAACATCCTTGCCAAGCACCACGTTAGTGGAGGCATCCACTTTGCGTTTGATGTCAGCCACAGATTCTTTTTTTGTATTCAAGATTGTGCAGCATCTATTCGCGAAGGACCCTTCTATCTGGTACTCTTCCCCTGGATTCTCATACACTTCTACGGCCACACTGCACCGTCCGATTATTGATGATCTTCTTACGCTGAAGATCGGTTTCTGTCCGTCTGTTCTTTCCCCTATATAGCCTTCCCATCGGTGATGTAGGCTTGGTCTCTTTT

>L0588

ATTCCTGGAAACTGAAGAACCTGTGGTTGAAAATGAAAAGGACATTGGTTCTGAGAAGCAGCCAGGAGAGGAGGATGCTGCAGATGCCAACAAGGACAGTCCTGTAGATCAGCCTGAAGAGAAGGAACCCGAGAACAAGGAGATGACTCTGGAAGAGTATGAGAAAATTCGCGAAGAAAAGAGGAAAGCTTTGCTTTCTACAAAGCCAGAGGAAAGAAAGGTTGATGTGGACAAAGAGTTTGGCTGCATGCTTCAGCTTTCAAATAAGAAGGGCAGTGATGACATCTTTATCAAACTGGGTTCTGAAAAGGACAAACGAAAGGATGCTGACAAAGATGACAGAGCAAAAAAGTCTGTCAGCATAAATGAGTTCCTGAAGCCTGCTGATGGGGAGAAATACTATAACCCTGGTGGCCGTGGTCGTGGACGCGGCCGTGGTTTAAGAGGTGGATATGGTGGAGGTAACATGCGTGATGCAGCAGCCCCCTCAATTGAAGATCCTGGTCAGTTCCCCACCCTGGGTGGCAACTGAGATCTTACATCCATATTTCAAAGTCGTAATGGTTGGAATTTCTTTGCAATCTGCTTAAGAGTAAAAGTTGAAGGTCATCAACCGGAAATGTTATTTCCTGCATTAAAAA

>L0589

ATTCCCCAGCTACGCGATCCACCTAGCTCGCCAGAGATTGAGGAAGGCTTGTTCGCTGGAAAATGACGTCAGACGGGGCCACTTCGACGTCAGCGGCTCCGAGGAGGAAGCCGTCCTGGCGAGAGCGAGAGAACAATAGGAGGAGGGAGAGGAGGAGAAGAGCCATAGCTGCTAAGATTTTTACTGGGTTAAGGGCTCAAGGGAATTACAATTTACCTAAACACTGTGATAATAATGAAGTCTTGAAGGCTCTTTGTACCGAGGCTGGTTGGGTCGTTGAAGATGATGGCACCACTTACCGTAAGGGATGCAAGCCACCCCCAATTGATTTTGTAGGCACCTCTGCAAGAATTACTCCATACTCTTCACAAAATCCAAGTCCACTATCTTCATCATTTCCTAGCCCAATTCCTTCCTACCAAGTTAGTCCCTCTTCCTCTTCATTCCCTAGCCCAT

>L0590

ATTCGTCTCAGTTTTTTTTTTTTTTTTTTTTTTTTTTGGTCATTGCAAAACCTCTTGGTTCTTCGCTGAGAGCCGTTTCAGAAACTCGTAGGTAGTGATCATTGTTGTTGCAGACATAGACATTGAAGCCCAGCGAGGCCCCAATCCCCTGTAACAAGCCGTCCAACCACCTTCCCTAACCAGATTTCTAATAGTCTGCCCAATAGTTGGTCCACGCCTCCCATTCTCCTCCCCATCCAAAACTTGCAATCTGGTCTTAATTGTATCAAGTGGCATTGTAATTAAAGCTGAAAAACCACCTGCCATCGCTGCACTGACTCCCTGAACTGCCATTACAGTTTTTGAATCTGGTCTCAATGCATTGAACCCATTCTCATTCCCATCTTCATCTTTCTTGTAAATGTAACAACCCACACCACCCCAAACCATCCTCTGAGCAACAGAGTATGAAGCCCACCAAACCGCATTTGATGGGGCATATGTCAAAATGGATATCCCAAAC

>L0591

TGGATCCCCCGGGCTGCAGGTTTTTTTTTTTTTTTTTTAACAGCAATAAGGTTGCAATTTAAAGTGTGAACGAGACCTCAAAGATTCAGTAAGAAGCTGAGACAGCAAGCCGATACAAAAGTGTCCTAAGCTTTTGCGGCTGAAACACTTTGATGAAGCCATCCAAACCAAGTTTCTAACAAACCTCATTGTCATGACATGAACATAACTAACATCAACTGATCCTATAAGAACTACAGTATAGCAGATATTGCCTCTAATGTCCTTCCACTCTTGCTTCAGTTCAGCTTCCAGCCCTAATCTCCATTAGCATAAGGTGAATCACGACGGGCATGACGGTATGGAGGGGATCTGCTGTATCTGCGACCACGGTGAGGTGAT

>L0592

ATTGTTTTTTTTTTTTTTTTTGACAGGAGAAAAAAAAAATTTTATTTCATTATCTAAGTTTCTTTCATTCCTTGGGCCTGTCCACGTCGCTGATGTTATTTGGATGTCAATAAACAAAAGGATCATAAGATCCAGAGTAATTTCTGGCTCTGCACATGGCAGGAAAGACAAGTAAAAAAACACTGGATAATACCAATCCAGATTAAGCAAACACAATGACAGCAATGCGTTCAACTAATAAATCCCAAGCTTCCCCCCACTTCCGAGTAAATCTCCTTCTAATTACGTCACCTTAATATAACCCTTCACTCAGACGCAGCTGCCTCCGCCTTAGGACCATGGAGAGAGGGAGGGCGCTGCTCCTTGCCCCAGCCAATGCCACGAGTGGCGTCAAGATATTGGTGAACGAGGTGGCGGCACTTCTGGAGGTGGTTGACGCCTTCGATGCGATAGCACCATTTGAGCTTCTCCCTCAAAATCTTGGCCTTCTCTATGTCGATCCACTTCTCCCTCACAATGTGCTCCCGCAT

>L0593

GGGCTGCAGGAATTCGAATTCCACATGTAAAGTATACAAAAGATGCAAAATTGCGGTTTTTTATGGACTCTTCCATTTGTACAATGCGTATTGGTACTATCTCCTTAGCTCTATGTAGCAAAAGCAAGCTGGTATCAACAGTCTCACGCATTCCTGTGGTGCTACTCGTATCTTTCTGCCGTGAACTTACCACAGCAATAATAATAACAAGATAATCCCAGTGCTTCTCATCTACAACTTGAACAGCAAGGCTGTCACTTCCATCCTCAACCTTTCCCATTTTCCACTTCACAAATCCACCAAACAAACTACGACAAGCACTGCCTGAACCTTGTCTTGCAATAGCAGAAAGCTCGCTATTATCTTCTTTAGCATTCATCAGCTTTGCAAGGGCAAAAACAAGACAAGCAAAACCAGCAGCTGAAGAAGCCAATCCAGCAGCAGTAGGGAAATTGTAATATGAAGCTATATGTACATGCAATTTCTCCCAATCCTTCTTTGAAATCTTGATACGCCTTTCTTTATCCTCAACATCACAGGGCGCGCTCACTGGCCGT

>L0594

TGCAGGATTCGAATTCGAATTCGCTATGATATTCAAGAATCTATGTCGTCGACCACCATCTCCGATGATCAAGACAAAGTCAACAGCGCCACCATCCAGATCGCTATGGCCAGGCTTGAAGATGAGTTCCGCAACATACTTCTCAACCACACTACTCCCGTCGAGTTGGACTCACTCGCCGCCGCCGATCCCAGCTCTTCAGTCCACTCCTCCGCCGCAGGCGAGTACGAAGATGACGATCATGTGGGTGATGACGATATACAAGACCAAATCCAACGAGCAGATTCATCTACAAGTAACAGCAGCACGAGCTATCGATCCACTAGCAGTATTCGCGAGATCGATCTGATTCCACAGGAAGCAGTGTGTGATCTCCAGTCCATTGCCAAGCGCATGATCTCTGCTGGGTACTTGCGCGAGTGTATCCAGGTGTACGGGGGTGTACGAAAATCTGTTGTGGACGCAAATTTTA

>L0595

CTGCAGGAATTCGATTCCAAGTAAAGAAGCGGAGCGGAAATGGAAGATATATGGAAAAGAGCGAAGCTCTACGCAGAAGAAGCGGCAAAAAAGTCCCAAACCCTAACCTCAACCAACAAAATCGCTGATCTAGTCGCCGAGACCGCTAAGAAATCCAAAGAACTCGCATTGGAAGCTTCGAAGAAAGCCGATCAACTAAAAGTCGCCGCTCTGAAGCAAGCCGATCAGATCCAAATCAAGTCCATTTCAGATATCATCCCTCCTCAGCTCTCTTCTCTCTCGATTGTGAATTCTTCTTCTTCTTCTTCGGTGTCTGCGGCTTCGGATCTCGAAGAGCTTCAGAAGTTTGGAATTACAGATGATTTGAGAGATTTTGTTAAGGGACTGACTTCCAATACATTCCAAAACTTTCCAATTCAAGATGAAGCTGAGGCGTCTGATGTGCAGACTACGGCGTCGAATGTACGGAAAGATCTTAATGAGTGGCAGGAGAGGCATGCCACTCTTGTTCTTACTAATGTANAGCAAATCTCGAAGCTAAGATATGAATTATGCCCTCGTGTGATGAAAGAAAGGAGATTCTGGAGGATTTATTTTACGCTTCTGAGCACTCATGTGGCACCGTATGAGAAGCAGTAT

>L0596

CTGGAGCCAATTCCACTGCAGTGAGAAAGTAGAAGCGTAAGCTCTGAAAGACTCTCTTTGGTTTCGGGTGCTTTTTATTTGGAAGAGAATGGCTTCTTCTTCATCTTCAACAACAGAGAGAAGAGGAATACCGGGGGCTCAATTTGTACAAGATGTCGAAACCTATCTCACCCAATCTGGTCTCGATGTCAACTCTGCTCTTTCCTTCCTCCAAGAAAGGCTTCAGCAATATAAGTTAGTCGAGATGAAACTTCTTGCCCAGCAGAGGGATCTTCAGGCAAAGATCCCTGATATTGAGAAATGTTTAGATGTGGTTGGAACTTTGCAGGCCAAGAAGGGTACTGGTGAGGCACTTATTACTGATTTTGAAGTCTCTGAAGGCATATATTCACGGGCTCACATTGAGGATGCTGATTCAGTGTGTTTGTGGCTGGGAGCAAATGTCATGTTGGAATATTCATGTGAAGAGGCCACTGCCCTTTTGCAAAAGAATTTAGACAATGCTAAAGCTAGTTTAGAAGTTCTTGTAGCTGATTTACAATTCTTGAGGGATCAAGTCACAATAACACAGGTCACTATTG

>L0598

ATTCGTTTTTTTTTTTTTTTTTGATAAATGTTGAGTATATATATATATATATATATTTGACCATAAGATAAAATTGCAATGTACCACACACATGCTGCCACAACAATGTCATCATGCAGAACTCATAATTACAAATAATTCATCCTTGAAATGCTGATCTTTCAGGTAATAATTGCTACATCATGTGAAATGGGATAAAAAAAAACAGCGAACAGGCCAAAATATAACGTACAATAAACAGTGGGAAGTCACGTCCCACGAAAAATCTTGCACTGTCATCAAAGCCCAGCCTCAACTTCAATGAGGTCTGTTCATCCCAGGTGCTGAAATTGTGGCTTGCCCAGCCTACTCTTCTTTCTTCAGAGATTGTGAAGAATCCTCTGCTTTACCTGTAAGCTGACTAACACTAGCTTTGGCCTCAGAAACAAATTTTGCATCTGGCTCATGCTTATCTGAAGGTTCCTTCATTGCCATATAAATGTAGAATGCAATAACTATGTTGACTGATATAACAGCAACAAACCCACTCACCAGTGTCAAGGAATGGGGAGATAAATGGCTTACACCAG

>L0599

GCAGGAATTCGAATTCTTTTTTTTTTTTCTACAGAAAAGACCAAGTATATAACATTAAGTTAAACAACTCCACATTACAGCCAACTTAATTTTATCGAACACTTTCCACATAACACATAAACCTCCTAATTCAAAAGCTTAGCCAACTTAATTTCATCAAACACTTTTCACATACCACAAAAACCTCATCTTATCATTCATCACATATATCGTAAATACGTGTCGCAGGAGAGAGATGACACTTAACTTGAAATCTTCAGCCAGAGATTTCAATAGCCTTAACATCAGGTTTCTTGACCTCCGCCTTTGGCACAGTCACAGTAAGAACCCCATTTTCCATGGAAGCCTTAATCTGATCCATCTTAGTATTCTCCGGCAGCCTAAAACTCCTGGAAAACTTCCCGCTGCTGCGTTCCACGCGATGCCAAGTATCGTTCTTGTCTTCCTTCTCCACATGCCTCTCTCCGCTAATTTGCAGCACCCGATCATCTTCAATCTCAACCTTCACCTCTTCTTTTCTAAGGCTTGGAAGATCAGCCTTGAAGACATGGGCTTCAGGGG

>L0600

ATTCCAACTTAACATCGGAGTCGGACACTGTCTCGCGCGTTGTTTCGTCTCTCTCTCTCTCGTTTTCACTTGGATCCCTTCCTCTCTCCGTCGCTTTCTCGCCGACCGCAACGGAATGAGTGCCGGCAACCTTCTTAGCGTCGAGCCTCAGGAGCTCCAATTTCCATTTGAATTGAGGGAGCAGATCTCTTGTTCCCTACAGCTGTTAAATAAGAGCGACAATTATGTGGCTTTCAAGTTACAATGCAAGCACAAAAGGAGGCGCCTCCTGATATGCAATGCAAGGACAAGTTTTTACTTCAGAGTGTAGTTGCAAGTCCTGGAGCTACCGCAAAGGATGTCAATGCAGAGATGTTCAATAAAGAGGCAGGACATCATGTTGAAGAGTGCAAATTGATGGTTGTTTATGTTGCTCCTCCTAGACCTCCATCACCGGTTCGAGAAGGATCGGAGGAAGGTTCTTCACCCAAAGCTTCTGTGTCTGACGATGGGAGTCTGAGCACCTCTGAACGAACAGT

>L0601

AATTAACAACAATTTCGACCTCAATCTCAACCACATACCCAGCCTCAACAACAATTCCGACCTGAATCTCAACTTCAGTTTCAGCCACAATTGCAGTCACAGTATTCTCAATACTCATCTGGCTATCCCCCTCCGCCCTGGGCTGCTACACCTGGTTATGCCAATGGCCAAAGTCGTATGTCTACAGCTAATAATATGTTCTCAACCCCACAAGCCAATGCAGCTACATCTTATACACCTACACAAGCAGCTAGGCCCATGCAGCATTATAACTCCTTTCCTACAAGAGGAATCAATGGAGTAGCCATGAATGGTGATCCTATAGTGAATTCTGGACCTAGGAACCCTGCACCTCCAGGACAGAAGCCCTTTTTCCATCATACAGATTATTTGAAGATCTAAATGTTCTTGGCAATGCAGATGGAAGGTTTAAGATGACTGGTAGCACATCACCAAGCTTGTCAGGACCTTCTAGCCAAGGGATGGTGGGTGGAAGGAAG

>L0602

GGGCTGCAGGAATTCGAATTCCTCGCAGATCGTCTCTCTCTCTCTCATTGAGCCAGTTTGTAATAATTTCTGTAAAATCCCCAAAACCCAGGCCCAAAACCCCCATATTTACTGTTTTCATTCACCCTCTCTGTGATTTCACCTGGCCACTGATTTATTAATATTGGGTTTGGGGTTTATTTTTGGAATGAAATTAAAAGAACAGGGGGAACACCAAGTTTGAAATCTTCGACTCCGTGCCTGCGTAGTAAGTGGAAGAGAAAGAAGTGGGGGGTGGCGCGTGCGAGTTGCTGTTATGGCGAAGCGCGTTTATGAAGTGTGGAAAGGAAGTAATAAGTTCATCCTTGGTGGGAGGTTGATATTTGGACCTGATGCTCGGTCACTGATTGTCACATTATTGCTGATCATTGTTCCAGTTGTAATCTTTTGTGTATTTGTTGCGAGGCACCTTCGCCATGAATTTTCACCGTACAATGCTGGATATGCGATTTTGGTGGTGGCAATCGTCTTCACTATCTATGTGTTGATTCTTCTTTTTCTCAAAATCAGCTCGAGGGGGGG

>L0603

CCGGGCTGCAGGATTCGAATTGGTGGAGTGGGCACTCTTTGTTTTCATGATTGAATCATATCTGTGTTAGTTGCCTGGAGGAAGATGTGTTAGATGAGCTGCCTTCACTGGATTTCTTCCATAATGGTTTTGATCTGCCAAGTTTGCAAAAGAAGCCTTGATTATTTTGGCTTTCACCCTTTTTTCCACAAGCACCACTTAAGAATCTGTAAATAACATTCCTGCAAGAATATGTCTTGAAAGACTTGTATTCATGGAGCTGTAAAATAATGAAGAGATGTATTAGGTCCTTCAGTTTTTTTATTTCATGTAAATGGATACATGTATTAGGTCCTTTAGTTTCTTCTTGTTCTACATCAGGTCAACATCATGTAATGGTCTCCTTCATTATTTATTTTGATCATTGATCTTTTTTCTGTTT

>L0604

ATTCTTTTTTTTTTTGATAAAGAAAATTCCATATATATATTAAATCACTCCTCTTGCGAAGCTTACAGTAACAGGATCAATTCAACAAAATTCTAGCCTCGCATATCTGGTAATGCAATTACACTTGCTAAGCAGTTATCCACTTGCATCGGGTTTAAACTCCATATTTCAATTTTAATAAAGTGTAATCACTTGCAACTCATTTATCAAATCAGTAACAGTAACACTCCAGCTTCACGGCTGTACAGCTTCCAGAAGTTTCTCATACACCTCTCTAGCAGACAAATCCTCCACAACAAGGAGTTTTGATTTGTTATTCCATCCCCTTTGAAGAGTCATCTGGCTCAGTCTTAGACCCAACACTTTGCCCATGAATTCTAAAAGTTCATTGTTAGCTTCCCCACGAGCAGCAGGCGCAGCTACCGTAACTCGAACATCATCAGCATTCACTCTTGTTATTGCTGAGCGTTGTGCACGGTCTTCAACTTCAATAGCTACTTGAACGAGTCCTCCTTCTAACTGAGATATACATGGCGGCACTGGAGCATCCTGCGGATTT

>L0605

CCGGGCTGCAGGAATTCGAATTGAATAATGAACATGACTTTTCTTTTTCATAACTAGAAAGGTATTAATATTGAATAACAGATGCAATGGCTCGATTGCTAAGCTTGCAGACACAAATGCATCAATATTGCCATTTCTAACACGTGGATGGGAAATTAGGATGAAATTTTTGTTCATTTTATATCTTTGTCTACATCATTCCTATCTAGCTATATACAATACCGCATTTGAGAAGAAGGAAGCTACTGTTCTCCTTGGTGGGTGCTTCCCATTGGAGACTCTAATCAGCCTTCTGATGTTGAACCAACGTTGCCCTGTTCCTCAAAGCCCTTCACTGGGTCAGCAAATGATTGAGTAGAAGGCCACCTGCTTGACATTATAGTCTCATCACCCATTCTCAATCTCTCATCAAATACGTCTCCGCCATAAAGATTCTTCTTAGAGAAGTATTTTGCCATCAAACGATGGGTGTAGTCAGCTTCATATTTCCTGTATTTGTCCATGATTTCTGCACTGTCAAGTATCTGGAAATGAGCTACGTCGGGCTTTTCCTGGGGGA

>L0606

ATTCTTTTTTTTTTTTTTTTGATCTTTCTCTTCTCGTCCCTGATACTTCTGTTCAAGCTTCTCAAACAGATCGAACTTTTGCAGGTCTGAGTCTGGGTTCCATCGAACTTTTTTGGGATTTGGCTGCTCTGTCTTTACACCTTGAATAAGTTCTGCAGGAAAATGCATAGGTGTGAGCAATAGAAAATTGTTAAGTTTATCACGTTTTGATGTGGACTTTGACTTTCCCCAATCAGAAAATCTTTCTATGTCAATGCTCTGGCTCTTCTTTGAAACAGTCTCATCAAGATAATAAGCAGAGCTTTTCCAAAAATTTTGTAACTTTGCATTCCCAACAACTAAGGCCCTTTCCTCCTTCACAGCTTTGTGATCTGGTAATTCAATTTCAGGAAACTCCACAAAAGGCTCTTGCTTTATATAGCCGAATCCACCACCTCCTCCTCCAAATCGCCCTCTCCCTCGACCTCGCCCCCGCCAAGCCATCTTTAGAGATGTTTCTTTCCTTTCTTTCGCTGCCTTCTCTGCTAAAGACACCGCTCGCTCGAGGGGG

>L0607

ATTCCAAAAACCCTAGCCTCCTCAATTTACATTTGCAAAAATGACAGGGGAGGCAGTGAACCCAAAAGCATACCCGCTTGCTGATGCTCAGCTCACCATAACAATACTCGATCTTGTTCAACAAGCAGCTAACTACAAGCAACTCAAAAAGGGAGCCAATGAAGCTACTAAGACCCTTAATAGGGGTATATCTGAGTTCATAGTGATGGCTGCCGATACTGAGCCTCTTGAGATTCTTCTCCATCTTCCCTTGCTTGCTGAAGATAAGAATGTGCCATATGTATTTGTCCCATCAAAGCAAGCACTTGGCCGAGCATGTGGTGTCACAAGACCTGTTATTGCTTGTTCTGTGACATCAAATGAGGGAAGCCAATTGAAGTTACAAATACAACAGCTCAAAGATGCCATTGAAAAGCTCTTGATATGAAATATTGTGGAATATGCGTATTCGGTGTGATGGGCCTCTTGGATTGTTAGTGGCCGCTCCATTGGAGGCTTGGACTGATGAAGTTGTATCACTAGGGACCAAAGCTTTTTACAATGACCTTTATTAAGATTCTTAGTTCTAAGTTTTCTGATGCATTACGACCATATTAGTATGATA

>L0608

TGGAGCCAATTGTTTTTTTTTTTTTTTTTAAGAAAAATGTACTTTTTCAAGGGAAGGGGAAAAAATAGTCAAATACCGATACATGAAAGGAAAACTCTGGCCTCGCCTCACCTAACATTAACAGCTCTCCACATACCGACATTTGTACAGTCATTGTCACGTTTAGACTATTTCAAACATATGATCATTTTTGTTTTTACACCCTTAAACACTTGTATAGTCATTTTATCTCGTCTTGCAGGGGCATCAGGCGTATTCCACATCTGAACCTCGGCTTCCTCAACTCTATGTATAAACCTTAAAACTACAAAATGTACAGTTTCTGGTACAAGCAATATTGTTTATTTTCAATCAGCTCATACCTGTCTAAAAGATCAAGTACAGACATATCTCTGGACACGTGAAGGAAGAAAGAGATGAAGCTCACACTAGCCTCAGTTAGCTGTACAATCTTTCTTTCCTATGCCACCTAAGCAGAGTGCAGAGACTGTGCGGCAATTCTCCGTTCCCCAGCAGCAACAAGTTTCTCTACACGAGCAACAATATGCTTTCCATAAGTATACTTCTTCAATGCGTTTAAATGAACTTTTATTCGTGAAAGAATCAACTCGCGTTGTTGGTCATCACAAGT

>L0609

ATTCCTGACATTAACATCTCTATTTATAAGTCACTTTTCTCACCCCGTTCCTTCGCCGCCTCCAGCACCTGCTCCACTTCACCCAACTCTCTCTATCTTTCCTTCCTCAAACAAGGTTTATCGGCGGAAGATTATACATGCATTTATTTGTCGACTTCTCTGAGCTGGTTATGGAGATGGCTGGTTGTGCATCGAGTTTCAGATTATCAAACGCTGTGAATTTGCAAAACAATGTCGTTTTGGGTTGTTGGAACTTGATGATTCGCAATAATTTTAGACTCAAAACATTAGCGAAGGGGGCGGTAACTTTTCATGTGCAGGTCAGACATGTCTCTATTCAGAAACAGAGGCTTGTGGTCAGGAACTCACAGACACCTTCTGAACCGCAATCGGGTGTGGGCACCACCACGGTTGTTAAACACGAAGGCGTGGAAAATGTAGTAGGGAAGGATGTTAAGATTTTGGAGAACGGAAATAAATTAAAAACTGATGTTGGCGGCAACGGTGGGGGTATATTTGATGGCAGTGGAGGAAATGGGAAATATCCAAGTGGTGGAGGTGGAGGTGGAGGTGGCGGCAGTGGCGGTAGTGAGGATGGTGAAGGAGAGGACAAAGGAGAAGAAGAAGAAGAGTTTGGACCCATCATGAAGTTTGATGAGGTGAT

>L0610

AATTCCTCGTATCGCGATGGGGAACAGATCCAAACTCACTTGGTTGTTATGCATACGATGTGGTCGGAAAGCCAGAAGATGTCTATGAGAGGCTTCGTGCACCCTTGGGAAATCTTTTCTTTGGAGGGGAAGCCGTTAGCATGGAGCACCAGGGTTCTGTCCATGGAGCTTACTCAGCTGGTGTCATGGCCGCTGAGAACTGTCAGAGGCATGTATTAGAGGGACTTGGTAACCTAGAGAAACTCCAGCTAGTCCCTTTAAGGTGTGAAATTCATGAATCTGCATTTCCCCTCCAAATCTCAAGGATGTAATTTTTTTCATTCAGTGAAGTATGAATTGGATTTT

>L0611

TGGAGCCAATTCTTTTTTTTTTTGTCAGAATTTCATCCTTAATCTACTACAAGATTGATAAATGATACAAATTTGGTGAAGATTTAAGAACTAAAGAGAAACACAAGGTTAGAGATTTAACTTCATAGTATTTGAGTCATCTTGGGGACTGAGCTCTCCAATTAGAGTTGTAGGTGAATTAAAGAAAGAAAAAAGCAGCAGAAGGTAAGCTAGCCTACGAATGGAGCATAGAATGTGAAAATTTGACATCAAATACCACAAATGTCCGAGACTGTCTAGACAATGATGACCAAGAGAAGCTCAACAATCAAATAAAATAGCAAACCACAACTGAAACATTACATGCACCATTTCATTTAGCCTAACAAGCACTACCTTTTAGGGTATAGAAAGCTAATAATTTGGAAAGATGTCAGATCATGTGTTATTCAACCTTTGACATCTTGATGTAAGATGAGCAATGGGG

>L0612

CTGGAGCCAATTCCGAGTTTTTTTTTTTTTTTTTCACTTATCAGAGAAATGCAAGCGTTGAGAAAATCGAAAAAGTGGGGTTCGTGATTTTTGTTCCAGAGCAAGAGAGAATCAATTTAGTTGGTTGATATCTCTCCGCTACTCCCTCTATATCTCTCTCTGAGTTTGGAGAGTGGAAAGCTAACGGAGGATATCTCTCTCTCTCTCTCTCTCCCCAGTAAAGAGGTGAAGGTGAAGGGATAAAGCAGAGACAAAAACTAGAAAAGAAAACCAAAGAGATTGAGTGGAAGAAAAATAAAAAAGTGTTAAGAGAATCAGGTTCTTCGTGAAATAAAGCACATACAAAGAGAGGAAAAAGAGGAGAAAGAGGGGGGGAAAATGCAAGCAAAGACACTCAAGAGAGCACGCAAAAGGAGAGAAGGTAAGGAAAGGAAACACTCTCTCTGTTACACCCGTGAATCATCTTGGATTTTTACAAGAACAGATGCAGATCCCACTCATTAGAAAAAATCTTTTGAATTAAAAAGAAACCCCATTAATGAGCACGAATCTTCCTGAACCTAAATCAATTACACTCTGCTTTACGGAGACAGATACACTATGTTTACTTCTCATCCGCAGATCTTAATATCACCCATCACGT

>L0613

AATTCTTTTTTTTTTTTTTAAGCGAATCTCAAAATTAACAATTATATCTGAAAAGGCTGATCTCCGTTATCTAACTCAAAAATTCCAGAATGCAAACAAGTTCCAAATATCATATCCAACTACAGACCAAAAAACTTATAATTTTTAACTAAAACGCTAGCACGAAGGCCTTGATACAAATGAAAGCAGCAACCAAATCTCCTAGTCAAAGAGGCTGAAACCCATATCCTCATCAGACTCCTCTTTCTCCTCAACCTTCTCTTCTTTTTTTGGCCTCAGCAGCAGGAGCTGCAGCACCACCTCCAGCAGCAGGGGCAGCAGCAGCAGCAACAGCAACACCACCACCAGAAGGAACTGAGGCCAACTTCTCCCTACCAGCAGCAATCAACTCTGTAACATCCTTTCCATCAACTTCAGACAGCAACAAATTAATCCTATCATCGTCAGCTTTTTTTTTTTTTTATTCTCATTATGAACATTCATAACTCACGACTGAAAACATAGTCTGAATACATAGGACAAGTACACTGAAAAAGCTCACAATACATATATAATNCAATCCAACATGCATTCTTGAGCGCATCATCATCTTGCTCAGTTGGAAGCAATTGGTGCAGAGTGTGGCACCTACATTCCAAAGAACTTAATAATTTGCTCGAGGGGGGGCCCGG

>L0614

AATTCTTTTTTTTTTTTTTGAATCATGATCTCAACTAATTTTGGATCTAGAAATTTTTCACAAGCATCAATACTCACTTCAGATTTACAGCCTTCATGAGGACACCTAGCCTCCATCCCATTAAGTAGCTTCACTTCAACATGTTGTTTCATACAAGAAAAGCAATATCTATGCAGACAACCATCAACACAAAACATCTGACCCACATCTGTATCCTCAAAGCAAATTACACAAGTCTCCTTCGAACTCTTTTTGCCTTTGCCAGATTCAGTAGACCAGGTTATCTGAGAAACTATAGCATCTCTTGCCAGTTTAAAGGCAAACTTTACATCGTTACGTGCAACAAGGGATGGCTTACAATCTGTAAACTTTTTCTGAAGAAGAGACACCTGATTGACTAAGGTTGAAATTCTATTTTGAATTGGTTGAACTCTTCCTGTCACATATTGATATACCATAAAGTCATCACAAAAGAAGGTGACGTTTTTCAAATCCAAACTCAAAGCCGCATTCAACCCTTCAATCAAAGCCTCAATCTGAACCGCCTCTTCACTACTCACGTCTTTCCCTTTACCACAAGAATCCTCCTCCAAACCCTTACTCACCTCAAAAATCACATGGTCCCTCGAATCACAAATTGCCACACCAACCCCACCTACTACAACATTCATTTCCCTAATCATCTCTTTACTCTTCAG

>L0615

AATTCGGTTTCCTTTCTCCGAGAATCTAAATCGCCCGTATCTATCAGGTGGACATGGAGACCGGAGGGAAAGTGAAAAAGGGGGCTGGAGGGAGGAAAGGTGGAGGTCCGAAGAAGAAGCCCGTTTCAAGGTCGGTCAAAGCGGGTCTGCAGTTTCCCGTTGGGAGAATCGGGCGTTACTTGAAGAAGGGGAGATATTCCGAGCGGGTTGGAACGGGCGCTCCAGTTTACTTGGCTGCAGTG

>L0616

ATTCTAGTCTCGATTTTTTTTTTTTTTTTTTTCTAGCCTAATTTAATTCCTATCTGAGCAAAATAGGAAAAAAAATTCAGAGAAAAAGTAAAGAGAATTATTAAAAACCGATGGGCATCATGGGCTCATGTCCTGCCAAATAAAAACAGTCAGAGGATTTCTGGTTTCGGAGGGAGATTGAGATCAAAATCAAGGGATTTCCGTGGTGGGTTGATCTCATGTTGGTTCAAATCAACGACGGATGACGAATCAGAATCACTCTGGACCCCCCCGCCACCACCATTTCCAGCAGTGGACATCGCCTGATCATGAAGATGTTGTCGCTTATTCTGATGATCAAACAACAGTCTTTGATACTGACTCTTCACCATCGCATCGAAATAAAACACGCGTTGATGATTCCCCACCGGTGCTGTAAAAACACCGCCCACAGCCGGCACAGGCTGAAAGGGAAACCTCACAGCAGCGGATCGGCCGAGATTAAGATCCAACGGCGAGGAGGATTCCATCATCAGAGCAGGCGTCTCCCGGCTCGATGACTCCACGGTGCTACTGGGACTCGGGCTGTGATTATT

>L0617

ATTTTTTTTTTTTTTAAAACCATAATCATAATCATAGATTTATTGAATCCCACATTACACAACAACCGAACCCACAATCACTTCTCCTTCTCCTTCAGCTAAAACGCACACTGGAAACTAAACGACAATCACTTCTTCTCCTCCTCCTCTAGCTCAAAGGCAGACACTGGAAAAGTCCTCGATATCCCTGTTGGATTACCGAAAGTTATTTTCTTAGTATCGCCAGAGTCCACATAGATATCAGAGATCGTAACCCAAATCAGAAACTCCTTGCTCTTCACCCCAGTCAGCCTCCGCATCCGCCGGTCCTCCACGAACGCCGTCACCTCCTTATCGTACGACACGTTGCGTCCAATTGCACGGAATTTGTTCTCTCTCTCTCTCT

>L0618

GAATTGTCTTCTTGTCTTGTTGCAACTCCTACCAGCTTTTACTGAATTTTTGAGCTGTTTCCTTTTTGTCCGAACCAGAACATCTCGGATTTCCCGGAAGTCGGTTTCTCCATTACGGCTTCTAATTTCTACAATTCTTTTTTTCTATTGGGGTTTCTAGAAAAACAAACTCCTTTTTCTAAACATATAAACAATCCCAATTTCACTTTTCACAACCCACCAACTACTGCTTCTAATTTTATGCAGTTGGTGTCTGCAGAGCTGAGAGCTTCAAGAAAATGGCTGCTTTGAGGTCTTCTTCTTCTTCTTCTTCTTCTTCTTCTTCTTCTGTCGAAGAAACTGAAAATCATCATCATAAAGAAGAGACTACCGGCAATCAGAAGGTTTATGAGCTGTTGATTCCTGGTTTGCCTGATGAAATATCTGAGCTTTGTCTTCTTCATCTTCCTTACCCATACCAAGCATTGGCGCGTTCTGTTTCTTC

>L0619

CAATTCCCCAAATTTATCTAAAAATTTCGAAGGAAATGGCGCTGAGGAAGTTCTACAGCGAGATAAAGGGGTTGAAAGTGAAGGAAGTGCCCAACTACGTAAAGCCGATGCTATCGATCGATTACGTGAAGAAATCGGTGCAGAAAGGTTTGGACAACTACCACGCCAAGTACATTGAGACCAGCTCCATTGATCCTCTCTACCATATCTGCTTCGGTGGCATGATTTTTTCCTACCTTGTTGCTCTTCCGGAGGAGCGCCGCC

>L0620

AATTCCTCCAGGTCCCGTTGTTGAGGCTGATGCCGCCAAGCCTGAAGTAGCCGAGCTGATCTCTTCCACTGAGGAGGAGACCCAGCAAAAGCTTAAGCTCGACGATGTGCCCGTTGTAGAGGACGTGAAGGAGGAGGATGATGAGGACGACGAAGACGACGATGACGATGAGGACGATGATAAGGAAGATGGAACCCCAGGGGCAAATGGGAGTTCTAAGCAGAGCAGAAGTGAAAAGAAGAGTCGCAAGGCAATGTTGAAGCTGGGTATGAAACCTGTTACTGGTGTTAGTAGGGTAACCATCAAGAGAACAAAAAATATACTATTTTTCATATCCAAACCAGATGTTTTTAAGAGCCCAAACTCTGAGACATATGTCATATTTGGGGAGGCTAAGATAGAGGATTTGAGTTCTCAGCTGCAGACACAGGCTGCCCAACAGTTTAGGGTGCCAGACATGGCATCTGTACTTCCGAAATCAGATATTTCTGGTGCAGCTGCTGCTGCACAAGCAGATGAAGAAGAGGAAGAAGTTGATGAGACTGGGGTTGAG

>L0621

ATTTTTTTTTTTTTTTTTTTTTTTTTTTTTTTTTTTTTTTTCCACATGGAAATATGTAAAATCAAATTAGAAAAACATTTTTTTAAGGAAAAAACCCACATAAAAACCATTGCTAGTTATTCCAGGACTTCAAGCATTACTACATATGCATTGAAAGACTACTTAGATGGTCTCAAAAGAAGCTTTAACAAGAAAGCATTGCAGCTAGTAACTACAGGCGTTTGAACCACCAGTACAAAGCAGAACTAAAAACAAATGGTAAAGAAGGCATAAGCTGGTATTTTCCGCAGATCCCATTTGTTCACCAAAATTCATCAGGAGAGATTGTGAAATAGAAAGCTATGGGAAGTAGTAAGGCCAGTTGAAATAATAAGAGTCACGCGTTTCTTTGTCCATCTCATTCCACTTAAATTTATATGGCTGGTTCTTCTTCATTGCTTTCTTCCAATGGTATTGCTCAAATAGAAATGCCTTGTCATGCCAATTGAACTGATTAGAGAGCTGCCACATGTAGGGATTGAAAAAGGTCAGGACAGAGAAAGCGGCCGACCCAATCCATATGTTGACGAAGCGGTCTGGATCCGCGTGCGGTACACCTGGGTCTCCTCTGTATGCATTTCCAAAGTACTTCTCCATTATCGCCTCGCCTCCTCTCC

>L0622

AATTCTAACTTCTCTGGCTCTTAGCTAATACGCCGCCGCGTAGCGAAGAGAGTTTCTCCTTATCTGGAACCCTAGCCATGTCGCTGGTCGCAAATGAAGATTTCCAACACATTCTTCGTGTTCTGAACACGAACGTCGATGGGAAACAGAAGATTATGTTTGCCCTAACCTCCATCAAAGGTATCGGTCGACGTTTTGCTAACATCGTCTGCAAGAAGGCCGACGTCGACATGAACAAGAGAGCTGGTGAACTATCAGCTGAAGAGCTTGACAAACTTATGGTGATTGTTGCAAATCCCCGCCAATTCAAGATTCCAGATTGGTTCCTGAACAGACAGAAGGATTACAAGGATGGAAAGTATTCTCAGGTTGTATCCAATGCATTGGACATGAAGCTGAGAGACGATCTTGAGCGGTTGAAGAAGATCAGAAACCATCGTGGTCTTCGTCACTATTGGGGTCTTCGAGTGCGTGGCCAGCACAC

>L0623

ACGCGGTGGCGGCGCTCTAAAACTATTGGATCACCCGGGCTGCAGGCAAATTTCCATGCTTCATGGATGTCATCTTGTTGTTGAGAGAAGGATATCTGCTCAGTGATCACCTAATATAACCTCCTAATCCGCCATTGTGCTTGTATTAATAAAAATACCAAAAATTTATCCTTTAATTTGCCTCCTCCCAAGCAAACAGAGTATAACTTTCTCACTTGACCCATTTCGATTCTTTGCCGTACTTATCGATGCATCCATTTGCGTTATGTTCGTACCCGATTTTATTAGCTGGGTAGATCCAAGATGTGCAGAACGACGAGGTTCGGGATGCGGGCTGATTGCGTGAAGAAGAAGAGAAAAGAGGAAGATGAACAAGCACAAGCACAGGAAAGCTGAGGAAAAGGCTCAGAAGACAGACTTGGATTTCTTTGCTGTTTTCTTTTTCTTGTGATGATGATGATGATGATGCTTCTACAGCTAGCTACNATCTGTTTTCAGATGAAGAAATCTGCTGGTTCGCTTCCATGTCTTACGTAGTTGTTGGTTCAAGTATACTCTGTATTTTGCAGCTCTCTCTCTATCCCTTATTTTCCTACTTTTTTTTTTAAAGGTTAATTGGCATTGGCAACATCATAGGTGGGT

>L0624

ATCCGTTTTTTTTTTTTTTTTTACTAAAACTTTCGTCATCATCAAATGGACTTGAAGAACCAACATTCCAATAGAAATCTAAAACAAATTAAAATATGCAAAAATATATGGACAGAAACTTCAAGTATCCAAAAATATATGAACAGAAGATAAGTTTACTACAAGTGCGGGTTACTTATCCTATAGACAGACGCACCCCATAAATATCTTAAAAACTATCCTTTGCTTGACAAATGATGTGTAAGGCTTCTGCATCATTTGCCATCGCTGGAATCTTTAATGACTGCCGGATTTCTGTACTTGAAGTGCGAAGGAATGGATTGCAAGCCTTCTCAACCTTCAGCACAGTTGGAATTGTTGGCAAATTCTTGCTGCGAAGATGGGCCACATGGGCTGCATAGGAATGGAGTGCCTTATTATTGGGTTCTATAGACAATGCAAACTTTGAATTACCCAATGTATATTCATGGCCGCAATATATATTTGTATCATCTGGCAAAGACGTGATTTTTCTCAGGGAAGAATGCATCTGCTCAGGT

>L0625

CCGGGCTGCAGGAATTCGAATCCCTCGAGTTTTTTTTTTTTTTTTTTAAGTCATAATAACCCAACTAATATACATTTATCAATTATACAAGGCCAAAATTTCCTTACCCAAAATTATAAAAATAAAAAAAGAAGGGGAGAAACCCCAAAAAGAAAAATCTAAAGATGAAACTTGCTAAATACCAATAATCCACATAATTTTTTTTTTTTTTTAAATTAAGGCTGCATCATTTCATCTGCCATAACCTTAACATACCTCACTGGCGCATGTTAGCAGCTACTATAATCATAGCCGCATCCACTCTAGGAGAAGAAGAAAAAAAGGAAGAGACTTTGTTTTGTTCCTCTAATTCCTTTCCCACTCAACATCTGAATCTTCCGCTTCTGGTAGCTTGTTCAAGTCGACCCTCTCCACAAAGGACTTGAATTCTGAGCAATCAATCGCCGATTTGTTGCTGCTATTGTTATTAGTGCTATTGCTACTGCTACTGCTACTTCGATGGTGGTCATGGTGGTGGTGGTGGTGGTGGTGGTGGT

>L0626

CCGGGCTGCAGGAATTCGAATCCCTTCTTTCTTGTGATACAAAATCCCTATTTCTCTTCCGTCGGACTAGTCAAGTCTAACATAGATTCCAGCTAAATTCTCTCCATTCTTTGGATCGAAACGCCAAAGATCTGCAATTGCACCCTTGCTTGTACAAAATATATGGCAACAACAACAACACACTGACCCAAGCTTCTCCAATGGCAAACACCCGCAAAGGCAAGTTGCTTTCTTTTCTCTCTCTGCTTTTCATTTTAGCTGCAATTTCGGGGAGCTGCTCTAGCTCGGTCCCTGACGATCACCACTCTCCTGACCATGTACCTGAGACTGAAACTACCTCTCAATCCTACCAGCAACAAGTTTTATTACATAAACTTGAAGAATTAGTGAGGAACCTCAGTGAAGTAGTTGCTAGATTAGAAACAAAATTATCGGAGTCTGCCAAAGTAGATGTATCTTTAAAGGGTACTCATCAAAATCGGGAACAAGATAGGGTTAATCAG

>L0627

GGATCCCCCGGGCTGCAGGTTTTTTTTTTTTTTTTTGATGTGGGTAGTAAAATCAAAGAAAACAAATGGAAGTGATGTTTATCCGCATAACAGGTACTGCACATCTTGATCAGAAAAAGGGTTCCCAGATCCACTCTCACTTAGGTCCATACTTTAATTTTATATTTCATCCCAAAAGCATGGACCAGGAAAGTTAAAATTTATTCTAGTAAATAAGCTCAGAGAGCCATTTCGTCATCTAATTCGTCCTCTTCATTCCAATCATACTCAACAACTACACCTTCTCCCCGCAGCAAGTGCAAGATCTGCTCTAGATCTCTCGCAGGCCCCTCAAAATATGCATTAGGACCTGGACAGGATCCATTTTCAGCTAAGAGTGGTGGTGTGTAGCTCGATGCTTTTAAGACAAACTTTCCAGAGGGTTCTAGAATTTCNACACTAAATGGAGGTGATAATGGTCGGCCCACAACTTGAACGTGAGAGACACATATATAGAATAAACCATCCATTTCTTGTATCTGAACCCTAACCAACAGCTCAACTTGTAGAATTCCACCTA

>L0628

ATTCCAATCGCCTCCAGAAAGAACTGGTAGAGTGGCAGGTCAACCCTCCTGCTGGCTTCAAACACAAAGCCACTGATAATCTCCAAAGATGGGTAATTGAAGTAAACGGAGCTCCGGGAACACTCTACGCTAATGAGACCTACCAACTTCAAGTTGACTTTCCAGAGCATTACCCTATGGAAGCTCCTCAGGTTATTTTTCTTCATCCGGCTCCTCTGCACCCACATATTTATAGCAACGGCCATATTTGTTTAGATATATTATATGATTCATGGTCCCCTGCCATGACTGTTAGTTCTGTCTGTATCAGCATTCTCTCAATGCTTTCAAGCTCAACTGTGAAGCAACGCCCTGAAGACNATGATCGCTATGTAAAGAATTGTAGAAATGGCAGATCTCCGAAGGAGACTAGGTGGTGGTTCCATGATGATAAAGTGTAATAACAGAGGTGTCTCCAAGTGTAATAACGGAGGTGT

>L0629

AATTCCCTTATCAGAGGAGCAGGAGTCAGTCACCTTATTATTCATCTGAACGCAGTAGGAGCAGGTCATATTCTCCTCACTACAGCAGGCGCCGATCCAACTCTCCTTATTACCGTCGACGCAGATCACATTCTCCCTACTACTATAGCAGGCGCAGATCGTACTCCAGGTCTCCTTACAGTAGGTACCCTGTAAGTAGGCATGATCATTCACACTCTCCTTATGATTCTAGATATTATTCACCAGATGATCGTTACTATAGAAGACATCGTTATCGCTCGATTTCCTGGAGTCCTAGCCCACAGAGGACAAGGAGAAGCTCAAGGAGGAGCTACTCAAGCACCATTTCACCTAGCCGAAGAAGGGGCTCAAGGAGGAGCTACTCACACAGCATTTCTCCTGCATCAAAAAGAAGCTCAAGGAGGAGTTACTCTGGAAGCCTTTCCCCAAGAGTGGGGAAGAGCACAAAGAGGAGTTACTCTAGTAGCCTTTCCCCAAGAGTGGGGAAGAGCACAAAGAGGAGTTACTCTAGTAGCCTTTCTCC

>L0630

ATTCTTTTTTTTTTTTTTTTTTAATATAATTTTATCAAACACTTAACATAAACTAACAATAATTACATCAAACAGTTAAAACAAACATACCCTTCATCTTCAATGGCATAACTATTGGCAAACAATTCCTTTAATCTTCACCCTACTATTTCATGTTCTTCTCAACTTTCAAACACCCATAAAACTTGCCAAGCCTAATTCAGATTTTAATGCCTCAAAAGATTGAGAATAACACATGCTAAATTTGAAAAAAATTAAAAAAAAAAAAATTCCAGCACAAAAAGCTGGGATGGAAAATATATAGAGGGAACGATGGAGTGGGTAAGGCAAGGGAAAAGGGACTTGATTAGTATCGTACAACATCTTGAAGGCAATCGTAAAAATGAGCAATCTTGATCTTTTGTTTGCCAAAGTAAATTGCTTCTGCAGCCCCTC

>L0631

CCGGGCTGCAGGAATTCGAATTCCTCTCTCTCTCTCTCTCTCTCTCTCTCTCTCTCTCTCTCTCTCTCTCTCTTTTTTTTTTTTTGGGGAATAAAAAAAGAGCAAATTCATATCCATAATTGAAGTGTTAAATTATGCAAGGATTCAAAGCACTAATTACTAATTAGTGATTACAAAAGCTAGCTGCAAGATGCAAGAGAGAGAGAGGAAACCATTTGGGTTTAGCATTCCACAAAAAGGGCAGCTGCTTGCTTCTGCTTTGCTCTATTACATCGTTTTTGTGTATATTACTGTATTCTTTTGTGTTACTAACAACTTCACATTTGACCTTAACCACCACCGCCACCACCAACCAAAAACCCTAATAAAAATAAATAATTGAGTATTCACGTTTACTCAATGGTCAGTCAATAAATCCTTCTCCTAGAA

>L0632

CCGGGCTGCAGGAATTCGAACTCCAGAAGATAAGAAGATACAATAATCTCATGGAGAGATCCAGAATATTCTACAGAATTAGCACTGAGCTTTCAGGAGACAACAGGGTGTTCTTATATATGGGACCATATCTGTAATGTGCAAAGAAGTCTTCAGTTTAGTACTCTTAACAATGAGACATTTCACAGCATGAGCAGCGAGTTGAGGGAGTTACCTGCAGTTGAACTTTCCACACTTCCTTTGATACTTAAGACTGTGTCTGAGTGTGGAATTACTGATCAGATGAGGTTGACTGAACTTATATTGAATGATCGAGGTTTCTTCCACAAGCTGATGGACCTTTTTAGAATCTGTGAA

>L0633

CCGGGCTGCAGGAATTCGAACTCCAAGATTCCTTCCGAATCAACTCTGCTTTAAAACCCAACCCCAGCCTCACCCACCCCCAGCGTAGCCGGACGATGCGGCTCCAGCTTCCTATTCTTCCAAAAACAAGCCTCTGGTGCGAGCTTAGCCGCCGACCTCATCGGCTCCGACTTGCAGCGAGTTAGCACAAACGGCTCAATACCCTTGGTTCCCACCAGCTTCTGCTCTATCACTGTAGACATAGACTCTGCGCCAGCGGCACGAGCCGGCGGCTTGGCCGGATATGAACAAGAAGACCTAGGTGGTTGCTGCAAGTTGTTGTGCAAAGGCGGAGGATTGATGTCAATGCTGATCCTTTTCTTCTTGGC

>L0634

ACTCCGATCATGGCAGAAGAAGGAGAAGATTCCAAATTCAATTCTCCCCAACCTCCTCCTTTTCTGGAGGTTGCCTGCAAAAGTTTGGGTAAAATAAGCCGCTTTGCGGCTGGTACCAGGGCAGGATTTGCAGTATCTTTGATAAACAGGAAGTTAGAAGCTGGAGCCCCTCTTGTTTCGCATATTGAAGCAGTTAAAGATGGGGAGGAACCCATAAGTTTTGGGCCTGATGCGATTCTTGTGGATTATAGCAATGGCTGGAAGTTGCAGACTGTTACTGAGTTGGATTTTGGTGGTGTTAGAAAAGCAGAAAGCATTCGTCTGATTCCAACACAAGCCCCTAATTTAATTTTTGAAGGCAGACGTCCAGCAGAGATAGTTCCTAAATCAGGAGTCAGTTTTTTGTACATTGCAAAGATATTACTTGCCTTTG

>L0635

AATTCGAGAGAGAGAAAGATTGTCGATCGGCGTGCCTCGAAGAGTCGGAAGATCAGGTACAATGTCCATGAGAAGATTGTCAATTTCATGGCTCCACAGGCCATGAACATTCCTCCTATGGCTCCCAAGTTGTTTGCAAATTTGTTTGGACTTAAGAGCCGCCAACCTGCATCAGCTGTTTAGTATCGCGGGTAGATACATCATGTGAGCTCAATTTTGTGGAGTTGAATCGAGGTTAAAATTCATGTTGTTTTTGTTCTTATGATAGAAACTTTTAATAGTATTGTAAGTTTGTAACTGAAAATGTCCTTTGCAAGCTACTTCTTGCTGAGAGATAGGGTTCATGAGTGAAACCGATCCTCAATGCTTAATTTTTTGGATTCATTTCTTGAACTTGAAATCTTACTATTCTAGTATTGTAGTAC

>L0636

AATTCGTTTTTTTTTTTTTTTTNTGGAGAAAACAAGGTAATATCATTGATTTTACTTGATGTATCAAAAACTTAAATTCACCAACAAAAATATGGGTCAAGTATTCATTCCCACTAGTAAAGATGCATGTCCCATTAATAAAGAAAAGCTTCATGATCTAAAATTACACTCATGTGATTTACAGGCCCAAAACGTCAGATACAGACTTGTTGATGATCTGAAATAGCTCTTCCCGCTCAGGTTTGAATAGGAGATATCGATATACAAACCACGAAGAGTATAGAATGCCAATAAGTTCTAGCACAGTTGGGATAAGCGTCAACTTGTCAATAGCGGATAGGAGATTTGCTGATGCCCATAGAGCTACTATAGCTGCAAACCCCAAGCCAACAATGGCCAGCCGATCTTCGGATTTATCCCAAATATTCTGTACGGACTTAACAATGCTAAGAGAGGTTGATGACTCGGAGCTTTCTCCAGCAGCCTTCACAACAACAGCCACACAATTCTGTCTCTCTCTAATGGGAGAAACTAGAAGTTTTAGAGGATTCCTGAAAAGGATGCTTCTACCAG

>L0637

ATTCTTTTTTTTTTTTTGCTCAGAAACATATATATATTGATTGTTTTTACAGGAAACTATATATATATATATATAAACTGTTATCAACATATGGAAATTGATTTATCAGGCTTCTTTTCTCCCTTTTGATGTCCTCCTAGAAAAAAAACAGAATTAAAAAAAAAAGAAAAAGAAATTCAGAGCACTGTCCCTTCATAAATGGTGCTTAATCCGAGCTTAAAGGACCGAACAGCCAGTTTCGCTCGTCTGGAAGTATACTTTTGTAATCTTTTAACAGACCCGGATCTTGATAACCCGACAGGAGATTGATGCTGTGCCCGTGGCTTCTCAAACGACTTAATAACATCGGCATCATCAGAGGGGGACAGATTCCCTAACGAAACAGGAGAAATCCTGCCCTTCTTACGCCGATGACCGCCTTTCTTGGAGGCATTTTGGATGGCTACGCTAACTTTGACGGCC

>L0638

ATTCCCCTCCCCTCTCTCTCTCTCTCTCTCTCTGAGACAAAACGCCGGTAATTGCTATGGCAGTGGCCACCGTCCCGGACAATCTAACCAGAGACCAGTATGTCTACCTGGCCAAGCTTGCCGAGCAAGCTGAGCGATATGAGGAGATGGTCCAGTTTATGCAAAAGCTCGTTCTCGGATCTACTCCGTCCGGCGAGCTCACTGTGGAGGAGCGCAACCTTCTCTCTGTCGCTTATAAAAACGTGATCGGCTCTCTCCGGGCGGCTTGGCGGATTGTGTCGTCCATCGAGCAGAAAGAGGAAGGGAGGA

>L0639

CCGGGCTGCAGGAATTCGAATTCGTTTTTTTTTTTTTTTTTTAATATTATATTTCTATCTTTTATTTTTGAATAATGATTTGCTTCAAATTAGTACTAGAAACTCAAATTATACTATATAATGTATAGTTGCATGAAATATGCAAATTACTTACGTATATCATCATCGCTAAACGGCTTTGAGTCCGCGATCTCGTCCGGCGGCACCGTGAAGCCGGTGAAGGAGAAAGACAAGCCCAAGCTGGCACTAGAGGCTCGGCTTAAAGCTGCACCGCTGCTTACGTCATCCAAGTCAAACTTCATCTGTGCACTCCATGACTTCCTTATGTGTGTGTTCTTGCCACCTCCACCGCTCCGCCCTGGTGATGCCGCCATAATCTGCCGCTTGCTGGACTTCCTCGACAATGTCCCACTTCCTTCCGACACTCCTCCACCCACCTCATTGGTCGGGGGAGATTTAAACTCCGCCGCCACCGTCTCCACTGGTTGGTCCGATTTTGTTCTCACTAA

>L0640

ATTCGTTTTTTTTTTTTTTTTNTGGAACAAGTTGCATGATATAAATGAATTATCTCATCAATTTTTCCTTGATCAATCAAATACAAATTCCAGAATTTTAAACATTCTGGGAGCAAAACTTTGTTTACCAATACATCAATAGGTATAACTGTTAGTGTTAGTCAAATTGGTGTTAGGACAGCATCATGAGAAATAACCTCTTGCTTCACTGATATCAAAAATGGAACTGAAAACCACCATCAAATCCGCCAAAGCCCCCTGGAAAGCCTCCTTCAAACTGAAATGTGAATTGTTGCCCTCCANCACCAAAGGGGTTGAAACCGGCACCCCCACCACCCATTCCCATTTCTTCTAAGTCTTCTCCTCTATCATATCTTGCACGCTTATCTTCATCACTAAAAACCTCATATGCAGCAGCAACTTCTTGGAACTTGGCTTCTGCTTCTTCTCTATTATCAACATTCTTATCTGGGTGCCATTGCAAAGCAAGTTTTTTATAGGCACGCTTGATCTCGCGCGCTCACTGGCAGTCGTTT

>L0641

CCGGGCTGCAGGGTTTTTTTTCTTTTTTTTAGTGTTAGTCAGAAAGAGAGAGAAAGAGAGAGAGAGAGAGAGAGAAATTTATACCTGCAGCATCAAATTCAACTTCTAATTTCATTCAACTTCTACTCTGTCTCTCTCTCTCTCTCCCTCTCTTCTCCTTCTCTCTCTTTTTCCACTTCGATCATCACTTCCCAGTTCCGGAAGCAAGGTTCGATTTCTAAAGTTCTTTACTTTTTTTTTAATCTCAATTTTAGTTAAAAAATATAACCCTTTCTCTCAGGGCAATTCTCTCTTTTCCCTTCTTTTATGCCTTTTATCTCCTTCCTTCAACTTAGTTACTCAGTGATTTACTCTCTCCCTCATCTCCTCTATCTCTCTCTCTCTCTCTCTTTTTTTAGGGTTTTATCGTTTGAAGAAGATTAATCATCAACCCAACGAGGACAGGTAAGTGGTCGCATAAACAACTTCTCCAAAGCCAGCCAACACTGTTATGGATCAGCGAGGGCATGGGCAGCATTCTGCAGCATGTGAGGTTGGAAGTACGGCTCCCGTCNCATATGGCATATCTTCGTATCAACCTAACCAGATGATGGGGCCTTCTGCCACTGGATCACTTCAATCTGCTGCTCAGCCATCATGTGTCACTGCATCTCAAGCTCAGCTTGCTCAG

>L0642

ATTTTTTTTTTTTTTTTTATTGACTTGCAAGCCATATAAAATTTCTGAAACGACAGTGTCTACTGTCTAGGGCCTCTAGGCTTGGGGTTCTTTCATCACCAAGAACAACCATAGAGATTGCGACAATTTTCCCAACCACAAAAACTCAATATTTAGTGATGGTAACAGAAAAAAAATTCCAAGGGTAATAACCCTCATTGCTGCTATGTTATTGTCAATATTCAATACATATGTTTCCCCAAAATCCATCCGGAGAGTTATAAAAATGGTGAGGGCAACAAACTGGGTTTGTTCCTTGATTTCTCAACTAGCTAATGCTTTTACTGCTTTCTCCGCAGCATCATCCAAGTCTTCCGCTGTGATTAGCGCCATTCCACTTTCCTTGAGAATTCTCTTTCCTTGGTCAACATTAGTACGTTCTAGACGAACCACCACTGGTACTTTCAGTGCAACCTGTTTAGCTGCATTAACAATTCCACTTGCTATCACATCACACTTCATTAATCCTCCAAATATGTTCACTAAAATTGCTTTAACCTTATCATC

>L0643

CCGGGCTGCAGGTATTTTTTTCTTTTTTTTTTTATGGGGAAAGCCATACTAAATAACTGACACATTGAACTTATAAATAGCCTTACAAATGAGAGTTTTCATCAGGATCATGGTACCCAAAACATACATCAAGAAATTTCAATGCAGGGCTCTTGTTCATCTAGATGCATACATCTAAGAAGCACATGATTGTCATTTCTACAAGCAATTTAGGAACAAGGATCATTTGGCATGTGTTTCAAGAATACCAATACAGCAACAGATTGCAGTTGCTCATCTTCCAAGTCCCTGTAGCATTCGTTCACCAAGAAATACAGCAAAGGTTTTTTGCTCAAAATTGACGTCTTGGCCTAGCTTCTGCAACAGAGACCCGAATTGCTCTGCCATCCAATTCAACGCCATTCAAGGACTCAATGGCATTTTCAACCTCTTCAGCAGAACTGTAAGTAACAAACCCAAAGCCCCTTGATCTACCACTCTCTCTATCATAATCACCTTAGCTTCCACAACTTTCCCTTGCTCACTGAACAAGGTCTCAAGTGCCAAGTTATCAACACCCCAAAAAAGGTTACCAACATACAAC

>L0644

ATTCCTCAAAACCACACCCTCCCTTCATCAACCTTCTTCCCCATTCAAGGGAAACTTGGCAAATTTGAAGTTTACGACCGGCAACTTCATTCCTCTTAAACTCAACTTCAAGAGCAATTCAAATGGAAGACCGTCACTACTAGTTACCAATCAGGCAGCTAGTGCTGCTGTTGCATCTCCAACTCCTAATGTGAGGTTCAGGTTGGACAATTTAGGCCCACAACCGGGGTCGAGAAAGAAGGGAAAGAGAAAGGGAAGAGGTATTTCTGCTGGACAAGGCAACAGTTGTGGGTTCGGTATGAGAGGTCAGAAGTCGCGCTCCGGTCCTGGTGTTAGAAAAGGGTTTGAAGGTGGCCAGATGCCCCTCTACCGCCGCATCCCCAAATTGCGAGGAATTGCTGGAGGTATGCATGCGGGTCGTCCTAAATATGTCCCTGTTAATTTAAAAGAC

>L0645

CAATTGTTTTTTTTTTTTTTTTTAGAAACAAAAGAAATTCATCAATCATATATTTGAGTTGGAAATTTCTTCAAACTCCAAAAGAGTCAAGTTAAATGCAGGGTAATAATTATATTTTGTCACCTTGCCGAAACAACATAGACCACACCACAAGCAAGCAGCAAATAGCTTGGTATATTTTGTAGAAGAATAAGATCCTTCTGGCTTGCTGAATAGTCAGGGAGAGCTCTTGTCATGACTGCAACACTGAACAGAGTAAAATTGTTCACAAATCTCTCTCACTCTCCATTGTCTCTATGAGAATGTACTTGTTTGTATCCAAAATAAAGAAAAAAAATAAATAAATAAAAGGAAGTTCTTCCTAACACATGCAAGATCTGCAAATGCCCCTACCAGCCCAATATTCCAACACATCGAATTCTTCCTTGA

>L0646

TCTCAAAATTTACTCGGGGCAGAACCAGCGTTAGCCGCCATGGTGAAGTACTCAAGAGAGCCTGAAAATGCCACCAAGTCCTGCAAGGCCAGAGGCTCTGACCTCAGAGTACATTTCAAGAATACAAGGGAAACAGCCCATGCTTTAAGGAAATTGCCTTTGGCAAAGGCTAAGAGGTATTTGGAAGATGTTATGGCTCACAAACAAGCTATACCTTTCCGACGTTTCTGTGGTGGGGTGGGACGTACTGCTCAGGCAAAAAACAGGCACTCAAATGGGCAAGGAAGGTGGCCTGTTAAATCTGCCAAGTTCATACTAGATTTGCTCAAAAATGCTGAGAGTAATGCTGAGGTCAAAGGCTTGGATGTTGATACCCTTGTCATTTCTCACATTCAAGTGAATCAGGCACAGAAGCAGAGGCGCCGAACCTACAGGGCTCATGGAAGAATAAATCCTTACATGTCCAGCCCTTGCCACATTGAGTTGATTTTGTCCGAGAAGGAAGAACCAGTCAAGAAGGAGCCTGAGACACAGCTCGCTG

>L0647

GGGCTGCAGGAATTCGAATTCGTTTTTTTTTTTTTTTTTGGGCTGTAAAGCAGAGCCAACATTAAGCAGCCACTGCTCATGATATTATAAACAGAAATGGTTACATTACAATCATGTTTACAGAAACATTAGTTAAATACAAAGACAACTGCAGTGATATGTGAAACAAAAATGACTAATTTAAAAGGCCTTCATATCAACATATTTGCCTGTGAGAAATACAATATAGAAGAGCACAAGCAAGAAAGCTGTAAAATATAAGTTAGATTGTGTGAAATGCTGCATTTTGGTAACACATAGCTGCCTCATTTTGTATAAGCATTAGCTATAGAGCCGATTTGGGGGCATCTTGCCAGACATAGCCTTAGTGGATGGGTCATCCAAAACTTTGTCCAGTGAACGATACTGCAATTTTACACTCTCTGCATTATCCAATGTCTTCACCACATACCATCCATTTAAGCATTGTGTTGTCACGACAGCCTCACGCCCAACAAACTGCTGAGGTGTCCTCT

>L0648

CAATTCTTTTTTTTTTTCTTAAATTTCCATCCGCATTATGTAAACAGATAAATGATCCTGTTTGAACAATTTATTACATCCCTGGTCAGTATGGTAAGTGATCTCTGTTTCTCACTTTATGTTGCAGGCCTCGGTAAAAATTCACATACCATTGCATGTAATGAATGGAGTCTCAAAAGGCATGAAATTCATCAAAAATTCACCGAAAGCAAACTAAACTGCATATTGATTACAAAGCCATGTAAGCTACCAAATGAACATACGCTGAAAATTGCAAGAAACCCCTGAACTCTGCAAGAATCAAAAGAACAATTGCAAATTAAGCACCTTGTATATTTCCTTCAGGCCAAGAAAGATACTCCAAAGCTGCTCTCATAGACCCCTGACTTTCTTCATGAGCAGGGCTTTAAGCTTACGACAATATTCCTGGGATTTAATCTTGCGCTGGAGATTTTTGCGTGCCAAAACGCGCTTCTCAGAATTCTTGATGTGGTGAAGCTGCTCGCGCTTTATCAACCGTTCGATTCCGCTCGATTGCATCTTCCTCTGCATCACGGTCAAAGCATGCTC

>L0649

CCGGGCTGCAGGAATTCGAATTCCATCTGTGTTTGAGCAGAACAGCCCTCTCTGCCCCTCCAGAAGAAAACCCAAGAAGAGAATTAGTCCTGTGGCAAGATTATTTGGGCCATCAATTTTTGAGGCTTCAAAGTTGAAGGTGTTGTTTGTAGGGGTTGATGAGGAGAAGCATCCGGCGAAGCTGCCGAGGACTTATACACTAACACATAGTGATATTACTGCTAGGCTTACTTTAGCCATCTCACAGACAATAAATAATTCTCAGTTGCAGGGATGGTCCAACAAATTGTACAGAGATGAAGTTGTGGCAGAATGGAAGAAAGTGAAGGGAAAGATGTCTCTTCATGTTCATTGTCATATAAGTGGAGGCCATTTTTTATTAGATTTATGTGCAAGGCTCAAATATTTCATCTTCTGCAAAGAACTCCCTGTGGTGTTGAAGGCCTTTGTCCACGGAGATGGGAATTTGTTTAACAATTACCCAGAAT

>L0650

CAATTCCCATGTCCGACGAGGAGCATCACTTCGAGTCCAAAGCCGACGCTGGAGCGTCCAAGACCTACCCGCAGCAAGCCGGTACCATCCGCAAAAACGGTTACATCGTCATCAAGAATCGCCCTTGCAAGGTTGTTGAAGTTTCCACCTCCAAGACTGGAAAACACGGTCATGCTAAGTGCCATTTCGTTGGAATAGACATCTTCAATGCCAAAAAGCTTGAAGATATTGTCCCCTCCTCCCATAACTGTGATGTTCCCCATGTCACTCGTACCGACTACCAGCTAATTGATATTTCTGAGGATGGATTTGTGAGTTTACTGACTGAAAATGGGAACACTAAGGATGACCTGAGGCTTCCAACTGATGAGAATCTTTTGTCTCAGATCAAAGATGGCTTTGCTGAGGGAAAAGACCTTGTTGTGACAGTCATGTCTTCAATGGGAGAGGAGCAGATCTGTGCTCTCAAGGACATTGGCCCAAAGTAATTTATCATTGGTTTGCAGATGGTTG

>L0651

ATTCCTCGAACAATCTACATACAGAACAATCACCTCGTCGTTGCTCAGCAGAGTCTGGTTTCCTGAAACATCGATCTCTTTCGCTACAGATTCACTCCGCTCTCAGCTTCTTAATCTCAAGCGATGTCGGAACAACCTTTTCGACCACGGGAGAAGCTTCTAGAGAAGCAGAAGTATTTCCAAAGCATCCACAAACACACATATTTGAAAGGGCCTTATGATAAGATCACCTCTGTTGCCATTCCTGTGGCTTTGGCAGCCAGCGCAATATTCCTTATCGGACGAGGGATCTATAATATGTCTCATGGGATTGGGAAGAAAGAATGAGGAGGCATCTTTTTTAATGACAGGCATCATGTTGATTTCTGCTCATATTTTAAAGGATTATTTGCTCTTGCACATGGACTATTTGTGTTTGAAGTATTTCTTTTGTTTAGTACCTCTAATAATTGCATACTTGTAGACTTCTTGCTACCGATGGAAGTTGACACTTCACTGTTGATGGATTTATTTTCTGAGTCTTTTAATGCTGCATAATTGAATTG

>L0652

TGCAGGTTTTTTTTCCTTTTTTTTGAAATAAACCATGCATCTTTGGATATGCATAAATCTCCAAATACCTCTTCAAAACTGACAGTAGTTGAGGCTCATATGAATAAACTAATCCAGTCAACAAAAGTTGGCAACACAAACCAAACAGCATGAAAAAAACCTAAAAATTGTAGACCTAGTAACCACCCTTGAGATCGTTAACAACATCATATGGGATGGGCGTAACAGTTTCCAACTGTGTACCCTCAACCATAAAACCAGGTTTAGGGCCTTCTGGCTGCCTCTTTGTGCTAATGACCTCACCTCTTGCCTTTGCCTGGGCCTTCAGTTCATCATTCTTCTTCTTCCTTAGACGGAATTCCTCTGTGCACCTTGAAGGCTGCAAATGCTCCACCCGCACATGAATCCTCTTTCTTATGATTCGGTTACCTACCTGCTTGTTGACCTCAACGCCAAAAGCACGCTTGGTAACGTTCCAGACACGACCGGTGCGACAATGATAGAACTTGTGAGGCATACCCTTGTGGACGGCGCC

>L0653

AATTCCATAAATGAAAATTTAAAAAGTACACACATTCGCCAAGTAAAACCCTACTCCGCTTCTGTCATATAATCCCAGCCAATACAGAGCTTCTCGCTGCTCTCTGGTTCGCACTTCGTCATACGGCTTCTCCTAAACCTTTCCTTCGCTATCTCCTTCGCAATTTCATATCATCGCAGCCATGTCTAAAAAGAAGACTAGAGAGCCAAAGGAAGAGAATGTTACACTTGGACCTGCAGTAAGGGAGGGTGAGCATGTTTTTGGAGTGGCCCATATCTTTGCATCATTCAATGACACATTCATTCATGTGACTGATCTCTCTGGAAGAGAAACCCTTGTTCGAATAACAGGAGGCATGAAGGTGAAAGCTGACAGAGATGAGTCTTCACCGTATGCTGCCATGCTTGCAGCACAGGATGTCTCACAGAGATGCNAGGAACTTGGTATTACTGCTCTTCATATTAAGCTCCGTGCTACTGGCGGGAATAAAACAAAAACTCCCGGTCCAGGTGCACAGTCTGCTCTGCGTGCGCTTGCT

>L0654

TCCGTCAACGTCAACACTTTCCTCCACCACCACACAAGCTTATTCTTAACCCTTTTCTACCCTCTATGCGTTCCTTTCTTGGTTTTTTCCCAATACCTAATTCTTAACGCAGTTACATCATACCTTCAGCTTCTTCAACTCTCCGAATTTGGTTTGACTGAATGCCCAATCTGCGTATAGTTGAAGAAGGGGACCATTGTTTGATTGCAGAATTCATAGAAGCATAAAGGAGATGGAGAGAAAAGGGAGCCCAAAATTGAATATCGCAATCATACACCCAGATCTTGGTATAGGTGGAGCTGAAAGATTGATTGTTGATGCGGCTGTTGAACTTGGCTCCCATGGCCACAATGTTCATATTTTTACAGCTCACCATGATAAAAACCGATGCTTTGAGGAAACTGTTTCTGATCAGGTTTCTGTTGTCATCCCACTGTTGAAACTTAAAAAATCAGCAAAGGTGGTATTTTATTGTCATTTTCCGGATCTGTTACTAGCTCAACACACAACTTTTCTTCGGAGGTTATATAGAAAACCTATTGACTTTGTAAAAGAAATTACAACTGGAATGGCAGATATGATACTT

>L0655

GGATCCCCGGCGCTGCAGGAATTCGAACTCTTCCCACTTTATTTCTCATTTTCCCCTTACGATGCCAATCAACAAGGACCCATCTACTCCCCCTCCTATGATCGGGAAAATCGGGCCCTACACTGTCTTCGTGACCCCTCCCTCCACCCCTAAACCGGCGGGGCCTGTTTTTGATTCCCCTAAAAAGGTGGTTTCACCGCCTCCCGTCCTGCCGCCGCCCCAGCAAATCGATAAGTCTGTTTCTGCTCAGCCTTTGTCTGATGTGTCCGTTTCTGGGTTCTTCACAAATGCTTTCTACAAAGTGC

>L0656

ATTCCCCCCTCCCTTTTTTTTTTTTTTTTTTTTTTTTTTTTTTAAATCCAAAACATATATACGAGTCTCACCTATTTTATATATAAATTCCACCATATTTAGTGTCTTGAGTTCATAATAGATTAAGTCTAACAAAGAATTTCTCAAGTGCATAAACCTACAGGCTACAGGTTTCATTGTGCCAATCAACATAAAACAAACATTTAATAGCGCTCAAAGTGCCCTCCCCTGCCAGGCCTGTCATATGGACCTGGCCTATTCCTATAGCTCCTATCATTTCGAGCAGGCCCTCCCCCACTTCCATACCTATCACTGCCTCCTCTTCCAACACCATCCCTATCGTAGGCTCTGTCTTTGCCATAACCACTGTATGCATATCGATCTGAACCACCATATCTATCGCTTGCAATGCGATCCCCACTTGGAACGTACCTGTCACCAGCATAGCGATCGCGGCTCCCATATTTGTCTCTGCCAT

>L0657

AATTCCGATGGTTCAATATATATGTACAAGCCCCAAACCTGCGTCCCCCCCCCCCTCTGCTGGCTGCTCCTCATCCCTCCCTAGTTCCTAGGCCTCGCTTTTCTTCATCCTTATCCTTATGTAACTTTCTACGTCGACGGATTATCGAGGACAATCACAGAACACGAAACCCAAACACCACCACATCTCACAGATCTCTCGCCATTCTCCACACCCAGAATTATCAATATCACCATGGCCCATCCTCCACTCAAGGCCGTCACTCTCACCCACGTCCGGTACCAAAAGGGCGACCACTTAGGCCATTTATTAGCTTGGATCTCGCTAGTCCCGGTCTTCATAAGTATAGGTGGATTCATTTGCCATTTCTTCTTCCGTCGCGAACTCCAGGCATGTTCTTCGCTCTTGGCCTCCTCATTTCACATTTCATCAGCGACTTTCATCAAG

>L0658

ATTGGAGCTTTCCTCGCTTGAATCACTGCTATACTTCACCTGTGTTTGAATCAAAGAAACTTTCAAATAGCTAAAACCATTGATTCCGACCCAGAGAAATCAACAACTAACCATTATCTAGAAGAACAAGAAGTGAATATTTTGATTATCTCATCAAAAGATGCAGCACGATGAGGTTATATGGCAGGTTATCAGGCACAACCACTGTAGTTTCATGGCCAAAATCACGACTGGGAATTTCTGTANAAACCCCTACAACGTGACGGGGATTTGTAATAGAAGCTCTTGTCCTCTCGCTAACAGTAGATATGCCACCATTCGTGATCATGATGGAGTTTTCTATCTGTATATGAAGACTATAGAAAGGGCTCACAAACCAAATGAATTGTGGGAAAGAGTTAAGCTTGCCAGAAATTATGAGAAGGCTCTTGAGATAATTGACAAACATCTGACTTATTGGCCTAAATTGTTGGTACACAAAGTAAAGCAACGGTTGACGAAAATGACTCAGATG

>L0659

AAAAATTCGTCTCTTTTTTTCAAACTTCAAGTACTGTGTTTTGAGTTTTTACCCTCCGTTTCTGCAAAAAAGAACTTTTCAATTATGGCAAGTGATCCCTTAAAATCTGACACAGATATAGCTGAAGAAGCCGTAACTGCAACAAGTGCTGTAAAAGGCGTGTTGGGCAGTAAAGGGGATATTGCAAGGAAAGCTGCCGACCGCTTACTTGGTAATAAGGAGGAAGACAGTCTGAAGTATTTGCTATTTGTGCAACTGTCTGCAAGTGTTGCCGTGTTTTCTCTCCCAAAGCTCAACGATTATGTCAAGAACGATTCTGGTCCACTGAAGCATGCTATCCAGACTATTGAGTCTACGGTTAAGACCGTGG

>L0660

CCGGGCTGCAGGAATTCGAATTCCCCAATCTCATTTCTCCTTCGAAGAACTCGCTTCAACCAATGGCTGCTACTGCTTTGGATAAGAGCGCGCCAGAGAAATGGCCTCCAGTGCTGGAACCCACTGCTGAGCAACCTCCACTCTTCGATGGAACCATAAGGCTGTATACCGCCTACATATGTCCATTTGCACAGCGAGTGTGGATCACCAGGAACTATAAGGGATTACAAGACAAGATCAAGCTAATTCCTCTCAACCTTCAAAGTAGGCCTGCTTGGTACGGGGAGAAAGTTTACCCCGCCAATAAGGTGCCATCTTTGGAACACAACGGCAAAATCATTGGGGAGAGTCTTGATTTGATTAAATACCTTGATAGCAACTTTGAAGGGCCATCTCTGCTCCCTGATGATCCTGCTAAAAAAGAGTTTGCTGAAGAGTTATTTGCCTACACTGATACTTTCAACAAAATTGTGTATACTTCATTTAAGGGAGATCCAGCAAAAGACGCTGGTCCTGCTTTTGATTACTTGGAAAATGCTCTTCATAAATTTGATGGTGGGCCATTCTTGCTTGG

>L0661

ACTTTTTTTTTCCACCAAACCATCATCTAATTACCTCAAAATAGTAAATAACCTCAGAAAAATCCTTAAATATGCAAAAACACAAATTAGATCTAGCACAGTTTCTCTTTGTAAAGGGAGTGAAAAAAAAAAAGGAAGCAGCTCACTGTAGCTTGTGATCTCTCAGTCAAGCGGCACCTCAACTTCACCATCAGTAATCTCTTGTTGCAAATCCTTGGGGTTCTTCCCATCGACCGTACATCCAACCGAGACGCAAGTCCCCAAAATCTCCTTGACGGTTCCACTTAGATCCTTCGCCATCGATCTTGGCCTCATAACCTTGGCGATCTCAATCACATCATCAAGCGAGATGTTCCCGCTGTGCTTGATATTCTTAGTCTTCTTCCTGTCTCTCTCTGGCTCCTTAAGTGCCTTGATGACTAGAGCAGCCGCCGATGGAACAACCGTCACCTTAGCCTGACGGTTCTGCACCGTAAGCTGGACAGTGACGCGAAGACCCTTCCAGT

>L0662

CTGCAGGAATTCGAATTCCATGGCCTCAAGAGCAGGAAGTTCTCAAGATTGAGTTGATCAAGCGAGGGGAAAGAAGGTAGCTGATGCACTCACGAATACCATATCTTCACTAAACAAATTTGAGCTGGGTGTGAAGGACTGCCTTTAAGTTTGGTCAATTACTTTATGTAATCCCAGAATCAGAGTAAAACTTTCAGGCGTATGCTGTGCTTGAATATCTTGCAAAAGCTTCTTCCCCCCG

>L0663

AATTCTCTCATCTTGCGTGTTGCCGGTGAAGTAACGAATCAACAAGACTGCAAGAACAAGGAAAGCAACAGCCAGACCGACCTTGCCAATTGATGAGGTGAGCTTATTGAGCCTAGCCTGCAAAGGTGTCTGTTCACTGGTGTCGCGGCTGATATGGCTCATCATTTCTCCCCATGTTGTGTTCATTCCTACTGATGTAACAAGCATTCTGCCATAGCCATCAGCAACCTTGGTGCCAGAGAACAAGAATGGATTTTGGTCGTGATTAACTTCAACGTGGTCGCTTTCCCCTGTCATGCTGGATTCGTCAATTTGCAAGGAATGCCCATCTACGAACAATCCGTCAGCAGGGATTTGGTCTCCTATGTTGAGACAAACAACATCTCCGACAACAAGTTCAAATATAGAAACCTGTTGTCGCCGACCTTGTCTTACAACATCAATTTGGATATTGTTGCTGACTCTGGACAACTTATCAAATTGTCTATTTTGCCTGTAATTGC

>L0664

CCGGGCTGCAGGAATTCGAATTCGCTCTAATAGACGAGGAAGATAACGAAGAAGAAAATGCTCTCTTCGAAGAGGACGGATTCGTTGTTCTCGATTCCGATATTCCTCCTCACCTCCGGGACCTTGCCTTCGCTGCTCAAGTAGGCGATGTCAACGGCCTTCGCGGAGCTCTAGATAACTTGAACGGAAGCATTGATGAACCTGTAGAGGATGGGGACACAGCTCTTCATCTGGTCTGTCTCTATGGTTATTTACCATGTGTCCAGCTACTTCTGGAAAGGGGAGCAAACTTGGAGGCAAAGGATGAAGATGGGGCAATACCTTTGCGTGATGCTTGTGCAGGGGGTTTTGCTGAAATA

>L0665

CCGGGCTGCAGGAATTCGAATTCCAGGTATGGAGGCTGTTGGAGAAGTGATAGCTGTGGGCCCTGGACTTACTGGTAGGAACATTGGAGATATTGTAGCCTATGCTGGCAATCCTATGGGCTCATATGCTGAAGAGCAGATCCTTCCTGCAAACAAGGTTGTGCCCGTTCCTTCCTCCATCAGTCCTGTTATTGCAGCATCTGTTATGCTTAAGGGAATGACTACTCAGTTCCTAGTCCGCCGTTGCTTCAAGGTTGAACCTGAGCATACCATCCTTGTTCATGCTGCAGCTGGTGGAGTTGGCTCTCTCTTATGCCAGTGGGCAAATGCCCTGAGTGCCACTGTCATTGGAACCGTCTCATCCAAGGAGAAGGCGGCACAAGCCAAGGATGATGGATGTCACCATGTAATGAACTATAAAGAAGAAAATTTTGTTCTCCGTGTC

>L0666

AATTCAGTCTTCCTAATCTCTATCTCTTCTATTCAATGTTTACAAAGAACTTAGGGTTTTGAAAACTGAGATGGGTTTGACCAATTTCATCATCACGGTGGCCGGTGTCAGCGCCGTCATTCTTCTATTAAGGAGCGATGTGAAGCAATCGGCTACCATTTTTAGGCGCAACGTCAAGCACATCCGTCAATGGCTCGAAGAAGAATCCGCCGCTGCCTCCAAGGCGTCAGAGAATGCAACACCCAAGGAACTGGAATCAAAGGTTCAGAAAAAAGATACCCCCAAGGAGGACAAGCACTAGTGTCTGTATGAAAGGCTTGCATCTTAAGAGAGTTTAGAGTCCTTTCTTTCCATTTGGTGGACTTCAGTTAAAATAAGTATTGTTTAATGTCCAGGGATGTCCGTAGCTAAAGTTTCTTAAATTCGTTTAGCCTTTTTGCAGTCACTTAAAATGTTGATTTGATCTAATTAATTTGTACTTATAAAATTTAAAGTGTGGCTATTGTAAGTTTAACTTTATACAAGA

>L0667

GGGCTGCAGGAATTCGAATTCGGGTAACGTATCGATTACGACACGAAATACACTTGCTCTCTTTCGCTACGCTATGCCTGCTAAAGGCGGGAACACAAAAATTTTCACCCCAAGCCTCTCCCTTGTCCTCCTCTTGTATTGTCTTTGTTACGGCTTCTCTGATATTTTGCTTTCAATCTTTGTTCATACCAAACCAACATGAATCTGCGCAAGGGCTCTAATGTTTGGGTAGAAGATAAGAATTTGGCTTGGGTTGCTGCTGAAGTAACTGGTTTTGCTGGAAGGCATGTCCAACTCCTCCTCACTAATTCTTCCAAGAAGGTTTTGGCTTCTCCGGAGAAGCTGTTTCTCAGGGATGATGATGAGGAGGAGCATGGAGGAGTAGATGACATGACTAAGTTGACTTACTTGCATGAGCCTGGAGTGCTTTACAATCTCGAACGCAGATATGCTCTTAATGATATTTATACATACACAGGAAGCATTTTGATAGCTGTAAATCCATTCACCAAGCTTCCTCATCTTTACAATGTGCATATGATGGAG

>L0668

ATTCTTTTTTTTTTTCACCAGAAATCTATATGCTTTCAAGACATAAAAATATAATTAATTGTGTAATGTAATAAGGAAAGAAACTTTTAGTTGGCACAATTCCGCACAGTTACAATAATTATAAACCAATTAACATGTTCTCTAGTATTATGGAGGGCACTAACTACCAAATTCTCTGGGTACTATAGAGGGTACTGCTCGTTTGAAATGACATTTTTCAGGAAAATCATTTCCGTGCTTCAGCCTTCTTCTGCTCTTTTGCAGCCTTTTCTTCTTCTCGCTTTCTGGCAGCTTCTGCCCTTTGCTGACGTATGAAAGTCCAACGCTCTAAGTCTTTTCTTGCTTGTTCCGTTTTTCCTTGTTCTTGAAACCTCATG

>L0669

ATTCCACCTCCAAGATATGAAGATATTTCAGAATCACCTGATGATGTGTGGCTTACTGTGTCGGAAGTACCACTTTTTACTCAACCCACAAGTGCTCCACCCCCTTCAAGACCCCCAAGGCCTCTACCACCTCGGAT

>L0670

CCGGGCTGCAGGATTCGAATTCGTTTTTTTTTTTTTTTTTGGGGCTTCCTACCACGCTTCTTCCCCAACCTACTACCTCCGTCCTTCCCAGAATCACAATTTTTCACCGCTTCCCACTACTTTCTTTCCATTTCTCCTCAATTTCCACCGCCTTCTTTTCCCCAACCTCAATAATTAATTTATTATTTATAACCCCACCTCCAATTTCCTCCACATAACCTTAAATTTCCGTCCCACTCTCTTCAATTCCTTATCGTTTATCGCGGTTTACGTCGCCGATTACACTGTAGAGTTCTTGATTTTCCGAAAATTGCGTCCTGTGGCAAAACATCGCCGCCTCATGCTCCGCCTTGG

>L0671

GTTCGAAAATGTTGCACCTAATCACAATAGAGTAAATACTGTATCGATTCGCTTAACCATCACCATGAAATAGTAGGTTATTAAATCACTAAGGAAATAGTCAAACTAACCACCTCAAATTGGTGTAACAAAGCAAAAGTTCCCAAAATATTTTACTGTGACGCCACCGCCTGTCAAGCACCCAATTCTGGGTGCTGCAAATAGAAAGTGGACACAAAAAAGTCCAAACGAAAATACAATTAAAACATCTCAAAAAT

>L0672

CCGGGCTGCAGGTTTTTTTTTTTTTTTAGCAAAGATAAAGAGGCAATAAGTAGCATTATCTAAAATTTTTAATCACTTCCTAAAGCATTGGAGCCACCAGAATTCCCAGAAAAGTCAACAGAGCGTCGGAAAAAGGAATAGGGGGTTTGGATAAGATTCCGTTAGAACTAAGGACCAACAGCACACAAATGCTCTTATGTTTGCCGGTACACATCATTAAGCATTCAGCCAAAACTAAGAACACCAAATGTGATCTTTCTAATTTAACTCTACAATACATAGTTCGCAAGGACCAGGAATTTTACACAAATTAAAGACTGGTCATTGTTGATCTGCCGCAGCTGCCAGCGGCTGGAATGCCTTGAGCGGATTTGTGATGACACCATCAATAAGCCCATAGTCTCTGGCTTCTTTTGCGCTCATGAAGTAGTCACGGTCTGTGTCCTGGTTGATCTTCTCAAGACTTTGAC

>L0673

CCTGACAGAAAGGAAAAATGTCCAAAGTCCCTCAGGATATCATCAACGACATCCTCCTTCAGGCTCATATCAAGGCTCTTCTCCGCTTCCGATGCTTATAAGAACCTCTCTGCTCATTAATTAATGGTCCA

>L0674

AATTCTTTTTTTTTCTCTAACAGCTGTTTTGCTTGTTAATAAAACAAACTATCACTCCAACAACCAAACTAATAAGAGGCTATTAAATATCACCAAAATAGCAGCAAGATCACATCCCTTAAAAGGGATAAGCTGGCAAATTTAAGATGTAACACTAATATTAAACTATCAATGTCTACTATATTTACATAACGCATCAAACTCTGCATTCCATTCTCAATATGCTGCTACTAGCAAATGGAGACTTGGCAAATTTAATAGTGCTGCCATGTATGAAT

>L0675

ATTCGTTTTTTTTTTTTTTTTTTTTTCAAAAATCATATATTATGGCAACTCAAAATTGAAAGTAATTCCACTGAATCAAAACATTCAACACAAATCAACTCTTTAACACTTCAAATATTCAAACTGAAGAACCAGCTCATAGTTGTCCTAAGCAAAAATTGCATAATAATCATAAACAACTAATTTTACTATGATAACTGAAATCTGATATTGATATGCATAATTATGCCTTCAGACGTCCAAAGAACTTCTGTTTCTCCTGTGTTGTCTGGAAGCGGCCATGTCCAAACTTTGATGAGGTGTCAATGAACTTGAGTTTAATTTCCTCGAGAGCAACACGAGATGTTTGGGTGAGCAGAAACAGGC

>L0676

CCGGGCTGCAGGAATTCGAATTCAGTTTTTTTTTTTTTTTTTTGTTTTTTTGAAAAGAAAATTGATGGTTGCTTTATTGGGATTTTTATTTTATAAAAATTGAGTTCTCATGTAGAGTTTTATCTAATATATGTACATCTTTTTCACGACATCCAGTATGTATTTTCTCATGACCAAACCTAGTGCCTAAACTCGACTGGCTCTCGCAATAAAGCTCCATCCATCGCTCTTCTTTAGTTTTGTAATTTCCTGTACATGGATTCTGTCCGTTTAGCACAAGAACAGCGACTCCTTCCACTGCTTGCTTTAGTACATCTTTGTCATACCATATTGAACTGAACCAACTTTTCTGTTAGAAGCCGGGGCAGTTACAGCAGTGCTGATCCCACCGTGCTGAGCCTTGGCATGCAGCAAATTTGTATTGATTGAAATTTGGTCATCGACCTTATTCATTCCTGCCCGGGTC

>L0677

CCGGGCTGCAGGAATTCGAATTCCTTCTTTTTCCTCTCATAATAACTCAAAAATCAGATCTTCTCTCTCTCTCTCTCTCTCTCTCTCTCTGTTTCTGAAGCTTAATTACTTGCGGCATTCCATTTCTGTATTCTTCATTTCTTTCTTCTTCCCTATCTCTCTCTGTAAGATCATGCCTGTTTTTGTAGAGAGTTCATCCACTATAGCGGAGCAAATCAAGCTGAGAAAACCCAGAAACCAATTCCATCACCAACATCAACAAGAAGAGAATCCCATTTCTACACAAGAAACAGATCCACCCCCACATATCTGTCGTCCCTCCAAATCCACCATCTCCTCTATCTTCCTTTCCCCCTTCTCCACAAACAACACTGAGGCAACTACCAAGAAAAAGAGCACCACA

>L0678

CCGGGCTGCAGGAATTCGAATTGTTAGTTTTTGTGTAATGGGTAGCAAACTAGTTTGGGGAGTTGATGCTTGTCTCTTAAACTCTTTGATGATGGTTCCGGTGGGGCAGTGCTTCCCTTTTCTTTCATGGACTTTTAAATCATGGTCAAGCATAACTGGTGGCGGATTGGAAAATGACAAATAATACCTTTCGGATCGGGCATGCCATTACATCTCCAGGCTCTCTTGCTGTTTTGCACATCATGGTTTGTGGTGGAGATTATAGGATTATGTTCTTTTGGAATGTCTGGTTGGAACCTTTTAAACTTGCTGAGATGGTTTTTGAGAATAAGTGGACAAATTTGTAGCTGGTTCATTTTCAAATTGTTTGGCTACTGTGAAACTGTGGTGGGTCTTCTGTAGATGGTTTGTCCTGCATATGATTTCTATTGTTAGTTTCATTGATCCT

>L0679

CCGGGCTGCAGGAATTCGAATTCTTTTTTTTTTTTTTTTTAATACAAGACTCTTTTCAATTAGCTGAATTTTCAAATCAGAATATTATTCTTGCTACTTTCACAAATATGCACATAAAAATATATAATAAAAAGAAAAGGTAAAACTACGTGATTATGCTCATATACATGACCCAACAATTTTACAGCCATGCTGTCTGTGTGTCCAGTAGGTATACCTATCTTCACTTGATCTTCACTTGATGGCTGCCCAAAACTTCAAGTAAAAGATCCATTAATACAATTCCCATATGCTAAATACCTCTTTGCTGCATTTGGGTTTCTTCCATAAACCCTCAATTTGTCAATTTATCCAAGAATTACCACCTTGATTGAAACCATGCTTTCCCCTCCTTTCAAAACCCATTTTTAATTGTAATATTCTTCTTCCTTTTAAGGTCCTTAATCTTCTTAGGGATGCTACGAATGGATTT

>L0680

ATTCCCAAAATGGAGGTGCTGAATGCAATGTATAATCTGGCGGTGCTGTGCTAAGTTCTAACTTATCGAGTTGTCTTGACGGAGGAGATGACTGTTTGCTGCCCAATCCTAAAATTGCCACTTTCCTCTCGCTTCACAGAGTATCAATCTGAGAGTCTGCAGGTTTCTAAGATTTACCCATCAAATTCACCAACACCATTTCTTTCTCTAGCAAACAAATCCTCTCTGCAAGTTCCTCGATTTTCACGCCTGTGGAAAAACACAACAAGGGCCATGGAGGTCCAGCAAAGTGGAACTGCAGTCTTTAAAGCTGGCAGCTCAAACGCACCAATGAAGCTCTTGTTCGTGGAGATGGGTGTTGGCTATGATCAACATGGCCAAGATATTACAGCTGCAGCAATGCGAGCATGCAGGGATGCCATCTCTTCAAATTCGAGTCCTGCATTTCGCAGAGGGTCCATACCTGGTGTTTCGTTT

>L0681

CTGCAGGAATTCGAATCCTTTTTTTTTTTTTTTTTTTTTTTTTTTTTAGCTGCCTGCCACAGAATTATTGATTCTACTATTCCCAGGTGATCTGCATGGTCCCAATATACAACATCAACTTATGTACTAAACTGCCATATATATGACTATGATAGGGAATTCAATTGAACATCCTTACATATAAATCTCAGCCAATACACACATTAACTCTCATTTACTTTCAGAAAGGAAACAGGAAATATGATTATCTACTCGATTGAAGCCGCATCTACTTCTCCAAGCTCTCCAGCCTCCGCGCAAGAATATTCAGAGACAGGGCTAATTTACATTCTCGAAAGATGA

>L0682

ATCAGATTCACAAGGATAAGGATGCTGAACCCACTGAATTTGAGGAGTCTGTCGCACAGGCTTTCTTTGATCTGGAAAATACCAACCAGGAGCTGAAAAGTGACATGAAGGATCTCTACATAAATTCAGCGATCCAAATTGATGTTGCCGGGAACCGGAAGGCTGTTGTTATCCATGTTCCTTTCAGATTGAGGAAAGCTTACAGAAAGATTCATGTTAGGCTTGTGAGAGAACTTGAGAAGAAGTTCAGTGGGAAGGACGTAATCCTGATTGCTACTAGGAGGATATTAAGGCCTCCAAAGAAAGGTTCAGCTGCTCAAAGGCCTCGCAGCCGCACGCTTACTACTGTGCATGAGGCAATGCTGGAGGATATTGTACTCCCTGCTGAAATTGTTGGGAAACGCACCAGATATCGAATTGATGGGTCTAAGATAATGAAGGTTTTCTTGGACCCAAAGGAGCGAAATAACACAGAATACAAGTTAAATACTTTTGCTGCTGTTTACCG

>L0683

CCGGGCTGCAGGAATTCGAATTCAGAACTAGTCTCGAGTTTTTTTTTTTTTTTTTTTTTTTTTTTTTTTTACAGTACAATTAAAGATTTTATTCGAGAGATAAGGATCTGTTATGGCACACGTTATGAAGACATGTGATTTTTCATAGATGAACAACCCATTGATTGCACCATGGTTGTGCATACAGATTCATGCCTCGTTTTGTTGTCCGCGACTTCTCTTCTCCTCGATACCCGATTGTGAAAATCTATGTCGAAAAACATCTGGCACATCTTCTAGTTTCACATCTTTAAGTGCCCGCAGAACCATAGCATGGTATAGCACATCTCCCATCCCTGATGCGGTACGTGTGCCGTGCTCATCCTCCCCTATTGTTCT

>L0684

CCGGGCTGCAGGAATTCGAATTCGTTTTTTTTTTTTTTTTAGTTCATAAACAAATTTATTGTCGTAAAAAGAACCAACAGCATCACACAAGACAACATTCTCATCTCTGTTTAATCCAGACAGCAGCATCAATAAAATGATTCCCTCCTATTACCGCCCACACCTAAACCACAACCCTCTTAATTAATCCTGCAGATGTTTATTAAGTAGCATTAGCCTATGAACTAATTGAATTGGGCACCTCATGCCTCGTCGTCACCAATATTAATAGCAAAATGCCCAACATTTCTATCCTTAGACAACATCCGCTGCTTGTTCAGATATTGGGTCCTGGCTTTGTTGAGGACTGAATTTGGAGACTTGTACAACCTCTTTTCACTAGTAGGACATTTGATGTTCG

>L0685

ATTCCTTTTATTTTACTTTCTTTTCAAAAACCTTAGTTAGCTCTATCCAAATCCATTGCACCAAAAGAAAAAATTCATTTTTTGATAAGTCAGTCACCATTTGAAAGCTACCCTTTTTTTTATTTTTTTCTTATTTGAAAGCTAACGTTTAGTTACCGAGGATGCTTCGTTGGAGATTAGGCCCTAATCAAACGCTTTGTTTTCTTCTTCATTAAGAATCTGTCCCTGTAGATCTATGCAATATATATAAGGATCAAACTGTTAGATTACCGTTGAAACGGAAAAAATGCCTACTCGCTGTGAGATCTGCTGCGAAATCCTTATTGCAATCTTGCTTCCTCCTTTGGGCGTTTGCTTCAGGCATGGCTGTTGCAGCGTAGAGTTTTGTATTTGTTTGTTGCTGACTATCCT

>L0686

ATCGTTTTTTTTTTTTTTTTTGCACTTGTACGCATTGGTTCATCATAGTGCTTGCCAAGAATATATCCAAAATAATTTGTTCATAAGGATAATGACCTTGACAGTATAACAACAACAAAACTACAAACAGATGCATGCATGGAAAATCAACAGAACAGAATCCGAAGGTTTCCATTTTAGAGTCATGTTAGCTTGGTTGACTTCCAACTCCACAAAAAACTGCCTCTGCAGTCAAAATACTCCTGCACTCCAATGGCTCATCATTTGATCTTGTCAATTATATCTGCGATGACTTTGTCCATGTCAAGATCCTTCATTGGTTTAAATGGCCGTTTCATGTCCAGAAGACTGATGAACTTCTCTCCACCTTCCTCCCGTATTTCAAAGCAACCCCTTCTAAGCTTATCGGGATTA

>L0687

CCGGGCTGCAGGAATTCGAATTCATTTCCTTCTTTATTTTTTTATGCCTTCTCTATTTATTTTCCAATCATAGAAACATAACACAGCATTTATTGGCAGTTGGGAGGCAGGAGGGAGATTTTGTTTCAGGGAGAGAGAGAAAGAGAGAGAGAGAGAGAGGCTCTTTGCTATTTTTGTAAAGTTCATGTTACTAATTTTCTTTTGTTTCTGTTTTTTCAATTCATAACTTACTAGATTATCTTTTGGTTTTCTGGGTTTTGTTCCTGTTGTTTTTTCTTTCTTTTTTATTTTTGGGTTTTTCCCTTCTCCTTGTCTCTCTTTCTCTCTCAAAATCAATTGGGGGCTGGCACATAAAAGCATATTAGGGTTTAT

>L0688

GGGCTGCAGGAATTCGAATTCCACTATCCTTCTTTCGCATCCTTTCTCTTTCTTTCTTGCTCTCTAACCCCCACCTTTTTTTTCCCATTTCTTGCTAAAAATACCCAAGAATTTCTCCAAAAGAAAAAAAAAAAATTTAATCATGTCAGGTGACAGACCTAAAGATTCTGCGGAAGGGTCCTCGCGATCCGGTGGTGATCATCAGCAGCAGCAGCTAATTCCTCAACCGGCTCCGTTGAGCCGTTACGAGTCACAGAAACGGCGGGATTGGAACACTTTCGGGCAGTATTTGAAGAACCAGAGACCGCCAGTGTCACTGTCGCAATGTAACTGC

>L0689

ACTGGAGCCAATTCCTGAACTCTTTTTTTTTTTTTTTAATAATTTAAGGAAAAAAAAATTCCAAATACAAAGTCGGCAAATATCAACAGAATATATCTGGTGGCCCGTTTAATCGTCATTTGCCATTTCAGCAGCACAGCTCTTAACAGAAGCCAAAAAAAAAAAATTTATCCCTAGCTTAAACACTACTGCTCTTGTTTTAGAGGATTAGGGTTTTTCCATTTCTGTTTCATCAACAATGGAGGCCTCAAAGGAGCAAGAATCCACTGGATTACTCCACAAGATCCTCCCTCCACGCCTCGAAGACGCGGGCCTTGAGGACTGTGCTCTCCCTGATGAGTCCATTAAAGAAGCTTTCTTCAAAGCTGCCTCTGCCATCAAATCAGGCGCCGCCTCTTTATTCTCTGACGATGAAAAAGACGATTGTGTTCAGGACCCTTGGCCAGAGGCCAAAGGCTTGACTGATGAGGTCATCGGTGGGTCACAGGACTCGGCTATGCTGGACACTTTGGTGGG

>L0690

ATTCATAACAGAACAGAGCCATTACCTATTCACCTTCTTGTTTTGCGGGAGTCACTTCAAACCACCGACATCGTTCTTCTCTTCTTTGGACAAAGACTCGCACCTGAAACCCAGCTTCTCCTAAAGTCTTGCTGTCATCACAGCGTGTTTCATCGAAACCAACAGATTCGGTTAACACGCAGACCCATTTACCCAGCAAACAAACCTATTCTACTTTCTCTCGCTATCCCTTTCTCTTTCAATTCACAGCGCCAATACATCTCCTATCAATAACCTTGAGAGCCGAATTTCTTTTGGAATCAATATCTAGTCATATTTGTCCTCCATTAAATTTGGGTAAAAGTTATTTGCATTTATTTATTTATTTATTATTATTATTGTTGATTTGTTGAACATTTTAAGCTTTGAAGTATTTATCTTTTAACTATTGTATTTTCTTTGATATTTATTCTGATTAATTTTACCTTGGGATTA

>L0691

AATTCTTTTTTTTTTTTTTTTATTGAATGAATTATGTCTTATTTGATGTTCTTTTCCTTCAAATACATAAACTGAATGAGGCTGACCCTAAATTTTTGTTGTTATATCAATTAAAGCTAAACTAGAACTAGAAGAAGCTAAACTAGAACTAGAATAAGCATAAATGACTGTAAATGATACATCAAACTTGTAGTAATCCGATGATCAACGGGTGTAGCGAGCTTTCTTCTGTGTATCTCTGAGATTGTAAGGTTTTTTGACATTGGTAGAAGCCTCCACAGAGCATGAATGACGTTTACTTCTTCTCAGCTTTATCGGTTGAGACTGTTTTTCACTCAATTCCAGTTCTTTTAATTTTTTTTCAAATTCCTCATCTTTCCTTTCAAGTTGTTTGGATTTCTCTTCTAATTGCTTCTCCTTCAATTCAAGTTCTTCCGAACGTTTTTGGAAAATACCCTCTTTCAGTTCTAGTTCTTTGGACTTGTCTTTCAACCGTTCCCTTTCCAAATTGAATCCCTCAACACAAAACTTGAATGTCAGAGATTTGACTTGAAAATCTTTTATTTCTTC

>L0692

CCGGGCTGCAGGAATTCGAATTCCTGATGATCAAATATTGAAATAAAAACTACAAAAGTAAAGCTAGAAGAGACGAGTAGCAATTCATCTAGCAAAACAACCTAACTCTTAGTACCTCTGAAATTTTGCTCAAGGCAGTTAGAAAATAAAGAAGCTAGGTATTGATGTTACCAAATTTGAGCCCATAACAAGGAAACTCAAGTAAGTTTTGTTTTCTTTGACTTAACGTGAGACTTCTCAGGGATTTCTTCTTCATCTTCAGAAGAGCTTTCGACCAGGTCATCAGGGACATCATTAGGAACATTATGTGTGGTGATGAATTCCTTCAATATGCTCATGCTTTTCTCTTTCATAGAAACCAATTCCTCTGAGATGGTACTACCGTTGCTTGCATCAACAGTGTATATTTCCGACCCAAGTTTAAGTGTGGCTTTAACATGAGGCCCACATGTAATTGCATCATGTGCTTGAGCCTCCATCACCTTTCCAAATCACAACTTGAAAGAACTGTACAATTGAGATCATAATAGTCTTCTCAAATGAAGGATGTAACAACAGGGATATTTCGGGGAACCAAACTCGAGGGGGGGCC

>L0693

GGCTGCAGGAATTCGAATTCCAGGATGTTCTCATAGGTGATAAGCTTCTCTCCGACTCATTCCCATACAAGGAAATCGAGAAAGGGATGTTGTGGGAAGTGGAGGGAAAGTGGGTGGTTCAGGGAGCAATTGATGTAGACATTGGTGCGAACCCTTCTGTAGAAGGTGGAGAAGAAGATGAAGGTGTTGATGACCAAGCTGTCAAGGTTGTCGATATTGTTGACACATTTAGGCTTCAGGAGCATCCTGCTTTTGACAAGAAGCAGTTTGTTACCTACATGAAGAGGTACATCAAATTGCTGACACCCAAATTGGAGCCAGAGAAGCAAGAGTTGTTCAACAAGCACATTTAAGGAGCTACCAAGTTCCTGCTTTCAAAGCTTAGTGACCTCCGATTGTATCTTTATCTTTATACTTCTCAAAATAACTAAATAAGAAAAGAAAAGCGAAGTCTAACTGGAGGTTTTTTGAAAATTCTCATGATTTAATATTCAATTTCCAA

>L0694

CCGGGCTGCAGGTATTTTTTTCTTTTTTTTTTTTTTTTTTTTTTTTTTTTTTTTTTTTTTTAAACACCGAGCCAACATAAAAAGCAATCACAATAAATAACAGATTAGTTGTTGCACAAAATAAATTAGCGTAATCAAATTTTTGAACATGGTGACAGTCTTGCAATTTGCTCTATCAATTTGACAAACTTGTATCTTAGTAGAGTAATTTTCAAGTACCCCACTTGCCACTTGTCTCAGCTGTGCATTCATGCCCTGATTTCTACTAAACAGTGGCAATCTAGGCTACAAATCCAAGCTCATTTACAAAAAGCAGAAGGCACGAGGATTGACCATTACAGATGTCTAGTTTCAG

>L0695

ATTCTTTTTTTACATAAACAGGAAACTGGAAAATTCTTCAACAAAAAATTAAAATCATCCAATCCATTATACAAGTAACAATTTCCAACTGAATTAGAAAGAAACTATTATTACAGACAGATAATGCAATTGTTATGGCCTATAACAATTAAAGAGAAGAATTATGAACAATTATAGAGCCTCAAAAGCAAGCATCCAAAAGACAACAACAGCAGAGTGCTGCTAAGCATCCTTCAAGGAAACCAGTCTCTTGCCTGGGAGGCTGGGCGTANCCTGAAGCATACCCTTGAGGAGGGTAACCCTGGGGAGGATACCCTTGTGGATAACCTTGAACAGGATACCCAGGCGGTGGATATGCATCTTTTGGATACCCTTGTGGTGGAGGAACACCCACCGGATGCTGCTGGTCGTAATAACTCATTTTTCTTTCCTATCTGTGGAGCCCTGTAGCTTTTTCTTTCAAATGACTAACATGGGTAATTAAATTGGCGT

>L0696

GGGCTGCAGGTATTTTTTTTTAAATAATAATAATAATAATATTATTATTATTATTAAAACAAAAATCCAACATTAAAGAGCTATGATACATTGTTCATTCAAGTGAGCATGTACCAAATGAAACATTGCAATAATATTCACAAAGTTCACAGATGCTAAATGTTGGAAGCGTAACTTCTAGCACAAAGAACTTCCCACTACACAGACCAAAGTCTCATCCGGTGGTAATAATTTGCGCAACAATCAACTCACACCGCTGTACACTTCTATTTATATAACTTATGCACAGAAGCAACTTCACAAAAGTTCTCTATATGATCAATCTGTTTCATGGTCAGGAGTAGGAGTGTACCAAGCAGGAAAGCTTCCTTTA

>L0697

CCGGGCTGCAGGCTTGGTTCACAATTCTCTTTTGTTGATCACAAAGAAAAGCTAGCTTATTTGCTCTGCTGCAATCTGGGTTCTTGTAACCAGAAGATCCAGATGATTATTTTGATGATTCTGTTGTGATTCTCCACTGTGCCTACAGATTGTTTTCTTGCTCTCTCTCCCTATTTCAGAGTTTGTGTTGGTGTACTGATCTCAAAGGAGACTGGTGTGTGTGTGGGAAAAAGCTCTGGTGCCTCTCTGTAAGACCTCTGTTTTGAAACTTCTTTTTTACTGGAATTCTTAGCCCTTTCTGTTGGAAACTTTTACCAGT

>L0698

ATTCGAATTCTTTTTTTTTTATAAAATTTACATATACTATTATTTAAGGCTCTTTTCCAAGACCCAACATTCATATTGTCAACCTTCATACTACGAGATGTGAAGGTATTGTACTAGTTTGACAACATAACAAGCCACATTACTGTTCTGATATTTACATTCTGGCAAGTCAAGGTCGGCCACGAAACACTTTCATGTAAAAAGCATACAGATGAACGAGGGATAATTATTTATGCAGCTTCCGCAAATCCAACTCTCTTATTACCATAGTCGAATACTGTATGGAAGCGACCCATGAAAACATCTCCCAATATCCAGAGGGGACCACGAGGTGGGGGCACATCTAAAGCTGTAAATCCACTAATGCATTGGGCGGCTTCTCCCTCACCCACTTTGAGGACATACTGCTCAGGGGAGAGGTCAAAAACCTTCCCACCAATTGTAAATGAAACATTAGGCATGGT

>L0699

AATTCGCCATTCAGGAAGCTGTCAAAGATGTAAATGCAAATTCTAGCATTCTGCATGGAACCAAACTTGCTGTCACAATGCACAGCTCCAACTGCAGTGGGTTTACGGGCATGGTTGAAGCTCTGCGATTCATGGAGAATGATATCGTTGCTATTATAGGCCCACAATCTTCTGTAGTTGCTCATATCATATCCCATGTTGTAAATGAACTCCAAGTTCCTCTGTTGTCCTTTGCCGCAACAGATCCCACTCTTAACTCACTCCAATTCCCCTTTTTTGTTAGGACAACACAAAGTGATTCATACCAAATGGCTGCAATAGCTGAAATAGTTGATTATTATGGTTGGAAGCAGGTAATTGCCATTTTTATTGATGACGATTACGGACGAAATGGCATATTAGCTCTAAGTGATAAACTTGCTGAGAGGAGGTG

>L0700

CCGGGCTGCAGGAATTCGAATTAGAGAGAGAGAGAACTAGTCTGAGTGTTATAGAAGGATATCAAGAAGGTGTCCAGCAGGTCAGGGAGAAGAAAGAAGAATCTAAAACTCAACAAGAGGAAGATACACCCGAGAAGTAGACTACTGATTACTGTAACTGAGGCAATGCAAGTATTTTTATTGCACATGCGAGTAATAGTCCTTTTTAATTTTGTCAAGTTACTGCCCTAGCAAAAAGAGTGCAAGAAGTTACTAGGTTGATTCATCCTCTTATGGGGATTCCATTGTACATTAAGTCTAGTCTGAGAACTATGGATACACTGAAAACCTTTTGGTATANAAAAAAAAAAAAAAAAAAAAAAAAAAAAAAAAAAAAAAAAAAAAAAAAAAAAAAAAAAAAAA

>L0701

ATTCTTTTTTTTTCAATCTAAAATAATCACCTTTATTGACTAGAAAAATAACTAAAAAATTAAGAAAAATAAAATCTTTAGCCAAGCCCTTTGTCCTACCCACACCTACCTTTTTAACCAGCATAGATATTTCATTGATAAAATAAACTTACAGAGTCGAGTCCACCAAAAAATATAGAATCACATTAATTTCAACCCAAGCTCAGAAGCCTAAATCCAATGGAACATGAATCCTTAGACTAACCGGGTTAACCCAGAGACCATTTTCATCAGCAACCTTCTTGATTCATCAATCATAGTCGCCGACTTTTCACCAGCAACACAAGCTTTTGAAGGAGAAACAGTAAGGTAACTTGAAAAAATTACATGCAGGGAGATACATATCAACATCAAATGGAGTGCATGAGCACCCACTTGACCCAGAAGCCTTTGCTCGCCCTTTCAAACCCATATGGAGAACGGGGAAAT

>L0702

CAATTCCCCCCCTCCTTTTTTTTTTTTTTTTTTTTTTTTTTTTTTTTACAAAACAAAAGGAAAAAGTTTAAATATAGATGAGTATATATTTAGTAGCAGCGTGCGATCATAGTAGCCAAGTATATAACCCACAATGTAATGCTAGCTAGAATGTCAAACTTATAAAAAGCATAAATAACAAAACACTCTCAATGTTTGATAAAAGGGGAATGCACAGCCACCCGGGTAAATTATGACACTACCAAAACCTTTAGTGGTGAGAAATTCACCAAAACTTAACTGCCAGTCTATGCAACAACAGATGGTTTGGAAGCAGCAGAAAGCTTAAATCTCCTCTTTGCCAAGCTCTCGC

>L0703

ATTCGTTTTTTTTTTTTTTTTTTTTTTTTTTTTTTTTTTTTTTTTTTTTTTTTTTTTTTTTTTTTTTTTTTTTTTTAAAACAGAAATACTTGAAATTTTATACCAATCAAATTACCACATCCATCAAATGAAATCCCCTGTATTTTAAACAGTATTTCCCCCGGCCATCATCCAATACCATAAACAAACTATTACAACAACAAAATGATCCCCATATCAATTCACTTTAACAACCCCCAAACCTTTTCTTTCCACACAGTGTTTTCCTTGACTTTTAATATTTCAAAACATTATTTCCCCGCCTTTACCCGTTCTGACAGCTGGTAACCAAATGCCACCTTGTATGGAACCCAAAAAAATTGCCACAAATAAAAGGACTTATATAAATATTAAAAACCCTGATTAATGAAATAATCCCCACCCCTTTCAAAAATCATGTTGCCCTGCCCTGAAATCATTGGCC

>L0704

ATTCATGAAGAAGAGTATAAGCAATTATTTGAGAAATATATATACATGCAATACACAACAAAATTCTTAGATAAGTTCCAGCATCTGGTGAGAAGAAATATATTATAATTGCATCACCAAGCAATTTATGTATCACCCAAAAAGGAGAAAAAAAAAAATAAAGCAGAAAATACAGCAGATAATCGAGAAGCTATGATGAGGAAGCTTTACTAGAAGAGGAATTGCCATAGATGTAAGAACCAAATCCCCAGAATGTTGTGATCAAAGAGAGAATCTTGAAACCGCTCATGGGGTCGTGCAGCAAAATAACAGCGGCAATGCTTGTGAGAGGTACCCTCACTGCATTGAGAACACCAGCCAGTACAGTAGAAGCCAGATAAAGCACAGCAGTGCCTC

>L0705

ATTCAGACAATTAAGCTGCAGATTTGGGATACTGCTGGACAGGAGCGTTTCCGGACCATAACAAGTAGTTATTACCGAGGTGCACATGGGATAATTATAGTCTATGATGTTACTGAGATGGAGAGCTTCAACAATGTCAAGCAGTGGTTGAATGAGATTGATAGATATGCAAATGATAGTGTGTGCAAGCTTTTAGTTGGAAATAAATGTGATCTAGTTGATAACAAGGTTGTGGACACACAAACTGCAAAGGCATTTGCAGATGAGCTAGGAATTCCTTTCCTAGAGACAAGTGCTAAAGATTCAATTAATGTGGAGCAGGCTTTCTTAACCATGGCTGGAGAAATTAAGAAAAAAATGGGTAGCCAGCCAGCTGCAAGCAAGTCAACAGGAACAACTGTTCAAATGAAGGGGCAGCCAATCCAGCAGAAGAACCACTGTTGTGGTTA

>L0706

AGTGGATCCCCGGGCCTGCAGGATTCGAATTCCATCACTCTCCTGCTGCACTACTGCATTATTCGTTAGGGTTTTGGAAAGTCACTTTCTTTTTTTTTTTTTAATTCTTTTTTAATTGACATGTCATCAGATTCTTCCCAACCTGTTCGTCCATCAGATCCGAACCCAATCACCGCCCACAATTCCCTAGATGAAGCGGCCACCACCGCATGTTGCTCCTCTTCCTCCACCATCACCACTTCTTCACCGCCTGTTGATATTCCTCTCTCTTGGCCTCCCGATGCCGAGCTCAGTTTCGATTGGATTCGACATTTCTCTTCTGCCTTGGACTGGTCTTCTAGAAATCTCCCTCCCTCAGATTTTCCTTCCGTCTTGCCTGTTTCCGTCTTTGACTCTCTCGTCCTCACCGCTTCCAAGATCCTTCATAAGGAGTCCAATTGCGTCACAATTGACGATTGTCTCTCTCTTCCTGATTCCACTGTTGTCGTTGTAGGAGATGTTCATGGCCAGCTCCATGACCTCCTTTTCCTCTTGCAGGATGCTGGATTCCCTTCTGACAATCGCTTCTTCGTCTTCAAAGGTGATTATGTTGATCGAGGCGCATGGGGTCTCGAGGGG

>L0707

AATTCTTTTTTTTTTTTTTTTANGGGGAAAGAAATTTGAACTTAAAATTTGATAAGCATAATCTAAAAATCATATGAGAAGCTTGGCCTTTTCTACATCTGATCATGAATCTGATAGCTCAATTAAGTCCCCTATAAACAAGACTACCCCAAAACCCATCTCCCCCTTTGCCTTACACCTCTCTTTTCTCTTCAGTACTTGGGTCTCCTGCCAGACCTTGGTTTCTTATCTCTTCACTTATTTTATGGCTGCCATGATGACCTGCTGTAAATGTAGGGATTCGGTCGCCAGAGCCAACTCCTTGAGTCGTATTAACTGCAGCAGTGCCTGAAAACCTCCCACGGAATTCACGTTGGAATGCTGTGTTAGCAGCCACTCCTACTTCATTTTCTCCACTGTTCATGTAACTTTCAGAACCTACCTTCACAGTTGGTTTTGGCGAGTACGACGTCGACCATGATCCTCCATTTGATCCCGCTAAGGCCATTATAACATCTGACTTGTGTGGGTGCACATCATCAAAAA

>L0708

AATTCCGAATGTCCCTCATTCTTTTAACTTATCGATTCCGGTCACCGTTATCGGTACCGGGTTGGGTAATGAATCGGTATGCGGGGTGGTAGGTCTTGCTTGTGCTCCAAATTATATGAACCATGTGAAGTTTTACAATAAGTGCAAAGCTCCTGCTAGTCATTTTGTGACTACAGATTATGGTCACATGGACATGTTAGATGATAATCCAACTGGTATATTGGCGATTATAGCAAATTCTATATGCAAGAATAGCAAGGATCCTAGGGATCAGATGAGGAGGACTGTTGGTGGCCTTATTGTGGCATTTTTGAAGGCTTATTTCCAAGCTGACAGTGGAGACTTCATGACCATTTTGAACGAACCTTCTGTTGCTCCTGCAAAGCTGGATACTGTCCAATTTAAGGAAGAACAAAATCACGCTCAAGTATAACGCCT

>L0709

ATTCCCTACATTTCTCTATAGAGCTTTGCATTTTCATCTCTCTACCTCCAAGAATGCTTCATCCTTAAGGAGCTTCTTGTGTTGGACTTGAGTTTCAATGACTTATCTGGTCCATTTCCAAGCAAGATTGCAGAGACTACAGATAAATCAGGGTTGGTTCTCCTTGACCTATCTCACAATCGCTTCTCAGGTGGTATCCCATTGAAGATAACAGAGCTGAAAAGTCTGCAGGCATTGTTTCTTTCTCACAATTTTCTTAGTGGGGAAATCCCAGCAAGGATTGGAAATTTGACTTATCTTCAAGTGATAGATCTTTCTCACAACTCATTGTCGGGTTCGATTCCTTTAAATATTGTTGGTTGCTTTCAGCTACTTGCATTGGTACTCAATAACAATAATCTTTCTGGTGAAATTCAACCAGAACTTGATGCTTTGGATAGCTTGAAGATATTGGATATAAGTAACAACAAGATTTCTGGTGAGATACCTCTGACTTTGG

>L0710

CTGGAGCCAATTCGCATCACTCGACTGCTCGGACCAAACCCTCTTTACCAGTGAAGAGGAAGGCGAGGGTCAGTGCACGGGCTCTGGAGAGATGAAAGCATCATGGTTGAATCGAATGGGGATTAGGTTCTTTTCAAGTAATACTTCATTCAAAGGGGCTTCTGTAAAGTGTAGGCAGCTGTAGTTTTCTTTTTTCTTTTTCCCATGTTATTATTTTTTTTTTCTTTAATTTTGGCTTGTAATTTTACAAGGGCACCATTGTAGGAAGAGGTGGCATGTAGTGCAAGTGTCCTAGATGTAGGTGGTTCTCATCCCAAATGCTATTTAAGAAATTTGGATGAGAGGCTTTCAAAGATGAAAGCTGCATGTAGAGAATTGGGAAATGTATTCAAAGGAAAAGAACCANNAAAAAGAAAAAAAAAAAAAAAAGAAAAGAAGAAAGCTTTGTTGATGAGTGAAAGAGGTGTGGCCGAAGGCCTTTGAAAAATGAAAAGCCTCGTCTTTTCTTGTTTCTCCAAAAAAA

>L0711

CTGGAGCCAATTCGTTTTTTTTTTTTTTTTTGTTGAAAGATTGTAACAGTATTAATTATCCTAATAATGTTAACAATAATATCTGCTTACAGCATATAGAAAGGGTGACCTTAGATCTAAGGTGGTCATGCCAGTTGCCGAGAATTGCCGGCCAAATAGATACACATATCTCAAAAACCCCCAAAAAATATTGGAGAAATATAGAAACAAAACAACAGATTTACATCATCCAATGAACTGAAATGTGATGCAGCTCCCATCCTACGTCCACCATCATCCACTCTTCATCATTTTATATCCCAATTCTCCTTTACCTTCACTGTTAATTACAAATCCGACTGCCATAGCCCAAAACAATCATACTCAAGTCATTTTGTCTGCCCAAAGGCAGTAATCCTTCAAGGTCCTCGATACTTGCGGAAGCACAGACAACCACCCTGTTGATCAGGAGGGATCATACCCCCACCTTTTGATATGTCAATGGCCTCCAACATGGAGACAACCTCGTCCATTTCAGGGCGCTTGTCA

>L0712

GGCTGCAGGAATTCGAACTCGAATTCGTATTTTTTTTTTTTTTTGGCTAACAGGATTTATATCTCGGTTGAGATTGTAGTTTTTACACTTCTTCATCGTACTATTAGTAGATTATTTTAGCATTCTAAAATCATAGTTGCAAAATTTATTGTTCGTGGAATACTAAAACAATTGCTATAACACAGCTCTGAATCCACTAACATTGTAATAGCCCAAGCCAAGCCCAGAGTCGAACACAGTTTAGCATGGCTGAGCATAAACTAGTACTCAATGCAAAGAAAAAATTGTCCCATCGTAAAATGATAGAGATTCGAGTAAGTATGAAAGCAAAATCTTCTTATATGGTGCATTCTTCTGGAGAACTTTCTACTCAATTCAGTGTGGTGCATTGAAAAGAGCTCTGCTAATGATGCCAGTAGCCTCTATGTAACGGTCCACACACCT

>L0713

CCGGGCTGCAGGAATTCGAATTCGTTTTTTTTTTTTTTTTTACTATAGATATCTGGTCCCAGATATGCTTCTGAAAAGTGACCAAGATAATCACAAAAGTGCATATATCTTTCAACTCACTCGCAAAAATGCACAAAATGCAAAAACTATATAAGCATTATATCCACAAGCAAGTGGTAAGCAACTTACAGCAGACTTCATGGATTTTACACATGCATTGAAAGACACAACACAAAAAGAACTAAAAGATCCAAAAAGTTAAGAGTTGGGAGACACTGCTGCCGTTTATGAAAGAGCAAATTAATAGAAGGAGAATGACTGAAAGCTATCTGTATCATCTGATTTGCGCTCGACGCT

>L0714

CCGGGCTGCAGGTTTTTTTTGCTGAAAGCAATTCATACATGTCCAAGTTATAATAAGTTTTTCAGGATTTGCAGCCACCAAAAGGCAAAAGGCAAAAGGCTTTATTAAATACCAAAAAGGATTCGGATTTAAAAATGAAAAACAGAAAGAGTCTCTATCCACACAGCACTTAAGCAGCAGAGAGATACATAATTGTATTTATTTAGATCATAAAGATGTTGCGAACTCTGCAAGTAACAAATTAAAAAGCACTCATTTGGTCCACCAATGGAGTAAATCCTTGCGTCTGTTGATCTTTTTCTGCAGCTGGAGTAGCCCATACAGCAAGGCCTCCGCAGTGGGAGGGCATCCTGGAACATAAATGTC

>L0715

TTTTTTGACCAAGTAAGAGACTGGATCTATCATGTCTCTTTGAAATTGCTTCTACACATGGAAGCTGCTCATAGAATACAAAGATTTTGGAGTCTCTGTACAAATTAGGTTAGCAGTAAAGAAAAGAAAGTTAAAAAACAACCTAGGTGAAAAGATACAGCTATAATTTAAAAGCTGAAGCAAACGGCCGCCAATGATGCTACATTAAAATTACAGCGCACAACTTGAGCTTCTAAGGAGTAAATTTACCTGGGATCCATTACATTTTCTCAGGCAAAACCTTGTT

>L0716

CCGGGCTGCAGGAATTCGAATTCTTTTTTTTTTTTTTGAAAGAGATATTTTGAGAATCTAGTTCAATAATTTAGTTGAGTTCGGAACCTTAGAGGTCGAACAAAAGCTATTAAAAAAAGGCTAGATTACCACCAAGAATATCACTGATACGGTATATTGCGTTTCTGTAATATACAGTTGGCACCCCATAAAAAGACTCCTAATGCCTACTAAGATAGGCAATTCATTCTATTTTTCCTATTAAGCTATTATTCATCGTTCTCGAACACTGGCTGACTTAGGCGTCGAATTTGCTGCCTTTAAGTCA

>L0717

GGGCTGCAGGAATTCGAATTCATTACAAATCCCATTTATATATTTTTAGCTAAGCTTTACCAAGAAAGCAATGGCTGGCAGAGAAATCTTAAACAAGATGAAGGAAAAAGTGGGATTAGGTTCATCTGCAGACTCTGGAAAGGGCAAGAGTAAGATATCAAAGAATGTAACCCATGGCTATCACTTGGTGAAGGGAAAGTCACATCAGGCCATGGAAGATTATATTGTTGCACAATTTAAGCAAGTTGATGACTATGAGCTTGGGTTGTTTGCAATATTTGATGGTCATCTGAGCCATGTTATTCCTGATTATTTGCGATTGAATTTGTTTGACAATATCTTAAAAGAGCCAGACTTCTGGGCAGAGCCAGAAAATGCCATGAGGAAAGCATACCACATAACCGATACTACTATTTTAGACAAAGCAGCTGATTTGGGTAAAGGGGGTTCAACTGCTGTCAGGGCAA

>L0718

ATTCTTTTTTTTTTTTAGTTAAAATCATTACTCTCTTACTAAGGTGGTTCAATGTGAATAAATTCTACGATACCAAATGAAAACGCAGCAGGAATATGACGACTAGCATATCCTTTTCGCTGAAAAGAGAAAGCAAGTCTTTCAGATATTACCTTCTCTCTTTTTTTTTTTTTCACATCAATGACTAGATTACAAAAACGTTCATATCAAAGAACCCTGCCTAAATACTGTGCGCCTCGCAATGATGCAAAGAATGCCTCCTCTATACGATGTAATGAAGCCGCACCAGCAAAAAGGTCGACCATTCCATCACTTGAAGAGAGGATTTCCGAAGCATTCTAAAGTGCCAACAATTGAGACTTGTGATCAATGCCATTGAATATGAAAGT

>L0719

CCGGGCTGCAGGATTCGAATTCCCTCATAACACCTAGCTCTTTTCAACAAAGCTTTGCTATATCTGGGCAAAACCTCCAAAGCTAAATTACACTCATTGATAGCGCGAGGATACTCACCTAGACCCATTTGCATATAGCAAGCCGCCATGTTGCTTCGAAGATAGGCGACATCGATGTGGTCTCTCGGAAGCAGCTTGACAGCTTTCTCGTACTTTAAAATGGCACCTTCGTGGTCGCGCTTCTGAAAGAGCTTATTCCCTTCTTCTTTGAGCTCTTGCGACATGTTAATAAACATTGCTGTGTCTTCATCAAAGGCCTTCGATGTCCGAGCCGTCGTGGGTTTGCTCTGCTTACCATTGGCGTCACCGGTTCTTGGTGAAGCCGGATTCTTCTTCTTCCCAGTTGGCTTTCGCATTTGATAGAT

>L0720

CCGGGCTGCAGGAATTCGAATTCAGAGATGCCATGCCACAACGGCTTTCGAACATTATCAAATGTACACAAAGTTTGATTTATTCCTTTTTATTAAGAAACATCACCCAGCATTTATTCTCCATTCCACATTTGAAAAGGTCTATCCTGTTCTGCGCGTGTCTTGTGGTCTGAGGGATTGGGCCTAAGTTCATAAACAATTGTTGCAACAACATCCCTCTTCAACTGGGTGGCTTCACCTTTAAAGCCAATATAGTGTATGCGACTTGTATCACCGCCAAAATTATCAGGGAAATGCAATGTGATGCTTGACACACTTTGAAATTTGGCATATCTTGTCTGGTACTCTAGCACTCCTTGCAAATTTTCAACCAGATCCCACTCCTGAACAGCTTGCATACTATGAGCGTCTGAGAAATCAATGCCATCTCGATTGGTGAACACTCTCATCTTTGAAGGGCTTGTTCCATCAGCATCACCAATAATTGAAATGCTTTTTATCT

>L0721

GGGCTGCAGGAATTCGAATTCCGTGCATCCAATAAGGCCGTACACTTAAATCGGTCCTCATCAGCCTCAATTGGGGACCATTCCTTGTAGCCATTTAACAGGAGTTTGTTAGTGTCCTTTTTGCGAAGTCTCAATAATCCCCATTTTACCCATTTAGAAGTTATCATCTTGCTTTGCCACTGGAATTAGTTATGTAGACTGTCTGGCGGTTATTCAAGGGGAACCTAGTTTTTCGAAATGCTATTTTCAGATGTTTACAAGCCTGGTAAGGATGTCACAAGATTAGACGTGTAAATAAAACTGTATTTACTGGGTGTTATCCCCCAGTTGTTACTATCATAAGGTATAGATACCTGTAACATTATGGCATTTTGGTCCCAGTGTTGTA

>L0722

ATTCGTTTTTTTTTTTTTTTTTTTTTTTTTTTTTTTTTTTTTTTTTTTTTTTTTTTTTTAGGGGGAAAAACTGCCGACTTAATTTCAAATTTGAAAAACATTCCATTCATACCATATTACTCATAACCTGTTAATATTACATCTGGACGGCTCTCCCTCTCTCTCTCTCTCCTGTAAACTGCTCTTGAAACTGGACAATGCCCCATTGACTTGCATTAATCACTTTCAATTCAGGTCTTAATGATGCTGGATTAAATTCGTGCTTGACCTTAAACTCTGCAGGCATAACAAACAAACCCCTCCCATATTGCACCTCTTAACCACAACTGGGCAAAAAATTCCCCAA

>L0723

GAGCCAATTCGAAAGAGCTGGGAACCATATGTGGCCGATGATGTTCAGGTTCAGTTCTACATGATGAGCCCTTATGTGTTGAAAACCCTCTCGACTGACCAGAAGGGTCTCTATCATGCATCATTCAAGGTGCCTGATGTCTATGGGGTTTTCCAGTTTAAGGTCGAGTATCAGAGGCTTGGATACACTAGTTTGTCTCTATCCAAGCAGATTCCTGTTCGGCCCTTCAGACACAATGAATATGAAAGATTTATAACTACTGCCTTTCCCTATTATGGAGCTTCTTTTGCTACGATGGCTGGCTTCTTTATCTTCAGTTTTGTCTACCTGTACAACAAGTGAAGTCCTGCTATTATAGAGGATTGAATTGCTTTGACATGATTAGGATTTCTTTCAAGCCTTTACTAGAAAAAAAAAAATTTTTTTTGAGGACTGCTTTTGCGAAATTTGCACCTCAAATTTGTTACTTAGCACAGAATCAGTGGATAATATTATCGCACTGAAGCATCATCGAGCATCAATTT

>L0724

AGAGGCCAACTGCTGAACAATCTCTCCAGCATCCATTTTTCCATGTGGGTATGTGGGTTCCTTATCCAATCCGAGATCCACTTGAACTGAAGCTAAATAATATAGGAGCGAAGCCAAATCTTGAGTTAAATCTGTGGGATTTTGGTGCTGAACCTGATGATTGTTTCCTTGGCTTGACTTTGGCCGTGAAGCCCAGTATTTCAAACTTGGAAGCAGTACACGATGTTTCTCAAGGTATGGAAGAGAATATTCTGTTTTGCTCAGATTTCAAAAATCATCAAGAACAGTCAGTGTTGTGGTCACTCCTATCGCCTAATCAAAATGGTATCCACCCTCCAGTCGAGTCATCATTGTCACTGTCCTTCAGTTCAATTCAGCATCCAACAGTTGTTCCACAATCATCTGGGTTCGCAATTACATCACTACAGCCTAACATCTTAGATTGTCCATTGTTGGCAACGTCATCCCCATTTCAGCAAAGCCATTACCATTGATCAATTCTA

>L0725

CCGGGCTGCAGGAATTCGAATTCTTTTTTTTTTTTAACGAACGTAAAATGGTCTACAAGCAAACACCTTCATCAGAATGCTACAACCGAACATCTCTATCAGAATCATTTGCTCTGATTTCTTTATACATTCGAGCTACTCTTTTCTTTAAAATCCACATTGGCAACAAGGATTGACCACAAAGAATAACTTATAGAGAAAAGGGAAAAGAAATTAACAAATTACCATTTGAAAACGTCTAAAAGGGAGAGCTGCCTATGCAATTGCAATCTAGCGAACCACCAAAATCCACAGCTAAATATAGTGTACCCTACAAAAAATGCCTTTCAATATTTACAGATGTGCAACATCAGCATGCGATGAATATCTATCTCAGTGTCAATCAAACTGTCTCATTGCTCCTCATTGACTATCTCCATCAAGCTCGGATCATCATATTGGGTGGTGTCCTTGATTTCAATGTCTTCATAGCAGCTTCTGCAAATGCTTGTGCTGCTTCAGCATCTGCTTCTGCAGCCTCTGCCTCCCTTGTAACATCTTCAGCTTCTGCTATAGCAGCTTCTGCATCTGCAACTG

>L0726

TTTACGACTTTCAGTATCTTATTCCCCGGGTTTTCATTTTAGATTAATCTTTATATGCTTCCTCAAAAGTTCTTGAACTTTTTGTTGGGTTGCTTCCTCTTCAAGATCTTATCTTACTCTGCTTAAAAAATAAGCTGAAAGAAAAATTTTCTCCTATTGAGAAAGTGATGCAAATTATTCC

>L0727

CCGGGCTGCAGGAATTCGAATCTGGCTTCTTCTTCTGCTGCTGCTGCTTCTGCCTCTTGTCCTTCGTTATCTTCTGCAATTTTTATTGTAACTGCTTCCACACTGTTTTAGATTTCCAATTCTTGCTTTGTTTCTACTTCAACCTGCAGCTGTTTCCTTTCCCGCCTCTTTTTCAAGATTTTTCCGTGACTTGAAAGCCTGGAAGTTACCAAAAGACTAGATATTTGCCTCTTCTGTGCTTGATTTCTTGGGTTCTCTCAGGGAAAGAAAGAGAGAAATACTTGCNAGACAAGGATGTTCTATGGGCTATAATCTTAAGACACCTTGGAGATTAGCTGAATTGGAAAACCAAAACATTCCTCACATTACTCAAACTCCCGTGTCTTGTAACTTAAGAA

>L0728

TGGATCCCCGGGCTGCAGGAATCGAATTCACAGAGGTGAGTGGGGTGAGACCAATTTTTCTTCTGTTGATTGGGTACTGCGTGGGAACTGTAATCTGAGCAGAAGAGCACAAAGGTTGTTATGGCTGAAGGAGGGGAGCAAATCGAGCTTAAATTCCGCATCTATGATGGAACAGATATAGGGCATGGTACTTATGCATCGTCAATTACTATTGGAACTCTCAAGCAAAGGCTAGTTACAGAGTGGCCTCAAGATAAAACAGTATTACCCAAGTCAGTTAATGATTTGAAACTTATACATGCTGGTAAAATATTGGAGAATAATAGGACACTTGCTGACTCCAGAATAACCTATGGTGACCTTCCTGGTGGGGTTATCACCATGCATGTAGTGGTGCAGCCTCCTG

>L0729

ATTCGCCACACAACAAGTTAAACTTGATTGTTGTGGAAAGGTATATTTATTTTCCTTGTAGTAGACGCCAATTTGGGCTTCCAGGTCCTTCTCTTCTTGAGATTGACCATGACGAGAGACCAGAAGATGGGACTTTAGCATGCTCGTTGGCGGTTATCGACAGAATACATCAAAACTTTTTTACTCACCCCTCCTTAGATGAAGCAGATGTTAGAAATATCCTTGCATCAGAGCAGAGGAAGATTTTGGCTGGCTGCCGAATAGTATTTAGCAGGGTGTTTCCCGTTGGTGAAGCCAACCCCTACCTACATCCGCTGTGGCAGACAGCTGAACAGTTTGGTGCTGCCTGCACAAACCAGATAGACGAACAGGTTACTCATGTAGTTGCCAATTCGCTTGGAACAGATAAGGTGAATTGGGCTTTATCTACTGGACGATTTGTTGTCTATCCTGGGTGGGTGGAAGCTTCAGCTTTGCTATATCGGAGAGCAAATGAGCAAG

>L0730

AATTCAGAGATATTTGCCTCCAGAATCAGAGCAAAAGCAGTTGCTTTATCCTTGGGCATGCATTGGATATCAAATTTTTTCATTGGTCTCTATTTCTTGAGCGTTGTAAACAAGTTTGGGATAAGCACCGTGTATTTGGCATTTGCAGCCATTTGTCTTCTTGCTGTCTTGTACATAGCAGGTAATGTTGTGGAAACAAAGGGAAGATACTTGGAGGAAATAGAGCGTGCTCTTAACCCTGCAATTTGATTTGGAAATTTGTCGAAAGTGCCTGTGGTAATAATTTCGTTGCGGCTTATGATTGACCTTAAGATGGGTGGTGCCTTTTTTGCTTTTTTGAAAGGTATCTTTGT

>L0731

TCAAATGGCATCTATCCCCTTCACGGACTATTCCTCCTCCACGGCTACTCGGCTTCTTCCCTTTAGCCGCACCACTTTCCGCCGTAACAGCGCCCTCTTTCTCAGCCGGTTGGTGGGTCCTCTCTCCATGCGCTCTAGCTCTTTCAGAACCCTCACGGTTGGCTCTTCTTCAGTTTCTTCACTGCCACTCATAAATGCTGCAATCGGTTGCGAGTCGGGTGCTAAGGTGATTGATGGAAAGCTGGTGGCCAAGAAAATAAGGGAGGAGATAGCTGCTGAAGTATCGAGGATGAAGGATG

>L0732

CAATTCCAGCATTCTTGTCATTTGACAACCCAAGGCGTGAGATGGTACGTGCTCATGCTCGCAGTAAAAGTGTGCTATCAGCTTTTTTTGTTAAACAGAAAACACCAAAACAGAAGGCTGGCCCAGTCTCATGAACGATCAAGCCTCAACAGAGCAAATTGTTTATGCCACCCTTGCGCTTGCCGCATTTACATCCACTGCGCTGGCAATGCTCTCCCTTGCTTGTTGATAATGACCTCTTTTGTGGTCAGTGAAAATTAATTGCTCATTCCATCCTAATTCTTACACTTGGTTCTAATAATGAGCATATTATGCATCAGAATCTGGTAATGACGATAATAATGATTGTCTTTACCCCTTGGTTTTGTTGATAATGCTAATAATTGTGGGTTATTGTTGATAATGCTGAATAATGATAATATTGATGGTTCAAGGTACATTATTTCAGGTAGTTGAGACCAATAACGCAAAATTATGTATCACTATTTGAGATAAGAT

>L0733

GGGCTGCAGGAATTCGAATTCGTTTTTTTTTTTTTTTTTGTACTGAAAAAGAAATCTAAACTTGAATTAAATAGCTCATACCCCAATTGCAAGTTCCACAATGTTTAGTCAACTAATTTAACAAGAAAAAACATGATTCAACATACAAGCAAATAAGCTACTGCTCATGGAATTCTACACTTGAAAGAAATTGAACATTCCCATAAAACAAAAACATGATAGCTTTAGGAAACAAACTCCTAAACCAGATATCAGTATAACTCAGGTATCAACCTCCAGTATTCATCACAGTCTGCAATCACTTCTTGGATTTCTTTGCTCCCTCAGTCTGCTGAGGGGCAGGTGCTTGTGGTGCCTTCTCTCCAGGTCCCTGTGCCAAACGCTCCTCTCTTCTGGCAATCTTTCTCTCCCTGCTTGCCTTATTCTTTGCACGCTTAGCCTCAAACTGGTCAGACAATGTCTTCTCTCTTGCCTTTTCTGCCTTCGATTTATGGATACTTTCCATCAGTACGCGTATATTCTTGAAAACATTACCCTTCACCTTCATGTACATGTCATGATGT

>L0734

CCGGGCTGCAGGAATTCGAATTCGATACTACTTGCCGGTGCAGACCCCAACAAGAACGAAGTCCTCACCCTCTCTCGCTCTTTGATTAATCAAACCCTCGTTTTAGCTCTCTCTCTCTCTCT

>L0737

CCGGGCTGCAGGAATTCGAATTCCGAAATCCTTTGTCTTATAAGCTGCATTCCCCAACTCCTTCTCTTTCACCGCTTGAGCTTTCCTCTCCTTCGTTTCTTTCTCCTCTTCTGAGACCTCCATTGGTTCTGGCTCTGGCTCAGGAACCTTCTCCTCCTTTGCTGACTCTGGCTTTCTCTCCGGCTGAGGAGGAGGCGACTCGGCTTCCGGCACCTCCATATCTTCTCCAGCATTTGGTGCTCTGAATTTCACGTTCAATAAAACACCCAAGGCTTGCATCACTCTCTGATCTTTCAAGTACAGATTCAAATTGCTAGGATTCTTTTGAATCTCCTGCATCATCTTAGCAAAATCCGGTTGCTGCAGAAACATCCTCGTAGTTGGATCCGCCGTCAATTTGGCCCACATCTCCGGCCCAG

>L0738

AAGAGTTTTTGCTTTCTCTCCTCTCTTTTTCTGAAGTCGTCAACGCAGATTAACCCAGGGGAGGTCTGGCCGTTGGCCGTCGACCCTCTCCCTACCTCAGTTAGCTTTTCCTTTAGTTTTATTAATTTTCCCTCTTGCTTTCCAAGTTTCCTTAGTCTCCAGTTTGGAGTTATTGGTAAGCATCTTGGATGGGGTTTTGGTTGTGTCTCTTTTGCCGATCTGCATACCGGG

>L0739

GGCGGGGAAAAGGGGCACACAGCTTCAATAATAAAAAATTGAGTGATCAAAGATTCAGTGTTTCATCAATTAAAAAAGAGTGATCAAAGATACACGTAAGCATTAATAAATGGCTTGCTATGCTAGGTTTCTCGGCACAAGGACAGCTACAGGGCTTTCCCAGCCATTAAAAGAGTGCATGCATTAAGGCACAAGAAATTGGTTGCAGCCAAGACCTGATCACACTTTGTTAGAGATTGCTATGTATGACACAACAACAGTGATATCATCAAGTTTGCCACCATAGTAGCGGAATCCAGCATCTTGAGCAGCAGAAGAAAAAGGTGTTTGTCGGTTCCTATCTTGTGCCCGTTGACGTGCCAAAGCCGCAATCTTCTGGGCTGTTACTTGAGGCCCTAACCCAGCTCTTAC

>L0740

CCGGGCTGCAGGAATTCGAATCCGTTTTTTTTTTTTTTTTTACCAAATCAAGAGGCCAACATCTTCATATATAGTAAGTAATCATACAAAAGCACAAGGAAATACCATATGGAGTCATGGGGTAAAATGCTCAAACCCTTCCCCTAAAACAAAATACAAACACTTTTAAATACTAATAAACTAAGAACACACAAAAGAAGGATAAAGAGTGACCCTACTCTAATCACTATATGTAGCAATTTTGTCCATGATATTTCCTAAGACCTTCTTGGACTCTCCAAGCAACTTGATGCTCTTGTTCAGCTTCTCACGTTTAGCAGCAATTGAGGGCGATTCCTCCATCATTCTTTCTATTACACCTCCTTGATTACTCATCAACTCGCTAATGATCTCCTTTTCCATCTCTTTGTTTACAAGATTTTGAACACAGAACTGCAAATGCAGAGCCATAGAATCAACCAATCTGCTCAA

>L0741

ATCCCCCGGGCTGCAGGATTTTTTTCCTTCCCCGTTGAGAACGGTGGCAAGTGAAGAAGCAGCCGCAGTATCACGAGACCCACCGCACTGTAACATTTTCTTGGATCTCTCTCTCTCTCTTTCTTTATTTGCTTCTCTCTATCTTTCCGAGTACCATCTTCAATTTCTTCTTTTCTTCCATACTTTCTATCTTAGTCAATTTTGCTTGCTAGCTGGGAATAATAAGGTCCATCTCTTTCTTTCTTTTCCTTTTAATCATCGTTTGTCTGTCTTTAAGCTATGCCTGACCCAACTTCAATAGCAATTAGCAAAGCAAAATACCAGTGTGTTTGTATCTTATAGTGTCTTTTGGATCTCGATCCATCTGCTTATTAAAGTTGGTGGTCA

>L0742

GGGCTGCAGGAATTCGAATTCCTTCTCTACATCCCTTCGCTGCAAAAAGTAACAATCTCCTCTCTTCTTTCTATATATATCACTATACTTCCGTTTTTATTTTGTTCCTTAAAGACAGCTTAAGCCCTCTTCCACTCTCACTTTCATTTCATTAAATTTCCTCCTTTTTTTTGGGTGTCTTTCTCTTTCTTTGACTCCCAAATCGACTGTTTCAGCTCAAGTTTTCTTCTTCTTCTTCTTCTTCTTCCATTTTGGTGATCTTGGTGAGGTAAAAGAGCAGTCAGATCTAAGACGTATACAGATTCTTCAGCAGCGACTATGTCTTTTACTGGCACCCAGCAAAAATGCAAGGCCTGTGAGAAGACTGTATATCCCATGGAACTTCTAACAGCAGACGGTGTTC

>L0743

ATCCCCCAATTCCCGATCATGAAGAGGGGAGCCTTAAACCCTAATATCAAGCTCAAGCTTTCTCTTCCTCCTCCCGATGAAGTATCCTTCGCTAAGTTCCTAACCCAAAGCGGTACCTTTAAGGATGGTGATCTGCTAGTCAATCGAGATGGAGTTCGAATTGTCTCGCAAAACGAACCCGAAGCTCCACCCCCTATTAAACCATCAGACAACCAGTTGAATTTGGCAGACATAGACACCGTTAAGGTCATTGGGAAGGGAAGCAGTGGAATTGTGCAGCTGGTGCAACATAA

>L0744

ATCACCCGGGCTGCAGGATTCGAATTCTTTTTTTTTTTAACCGAATGCTAATTTCAATTAAAATACCATTTAACTACATGAACCTCCTGAAGCAGCACAATAAACCCACTGCCTTTGCAGGGTAGCCCTGCGCATGTGATCACAAATACATCATCATATCCACAGCTGTAACTAAAATTTACTATGTCCAAATCCCATGTCTAGTTCATGACCATATATCCCCTGACTATGGCCAGCCATTGCAAAAGGATGAACGGGCTTAGATTTTGACCTGAGTTCCATCTCGATGAGCCTTTGCAGAGCCTCCGGATGATATGTTCCACCACCAACGTCAGGAGCTTGTGTTGGTATTCCAATGCCACCAAAGTTGGTTTGCCTTTGACCAAAAGGGAAACCTTGCATTGGG

>L0745

AATTCCAAGTTCTCAAATATTAGTCTTGAGATGCATAAAAGATTTTGAAATGGCTTGTAATTTCTTTCTTTTCTTAGGGCTATTTCTAGCTCTTTCCTCCTTTGCTCTGGCTTCTGATCCAATGGCACTTCAAGATTTCTGTGTGGCTGATGCAAATGCTACAGTTCTAGTGAATGGTGTTGCTTGCAAGGATCCCAAGATGGTTGAAGCCAATGATTTCTTTTTCACTGGACTTCACTTGCCAGGAGATACTTCAAATGCAGTAGGGTCTAATGTTACACCAGTCAATGTAGCCCAAATACCAGGACTTAACACTCTTGGCATCTCTCTTGCTCGTATCGACTATGCACCATGCGGTATTAACCCTCCCCACACACACCCTCGTGCCTCTGAAATCTTGACAGTAGTGGAAGGCAGTCTTGAAATTGGATTCATCACATCTAACCCTGAAAAACGTCTCAT

>L0747

ATTCGTTTTTTTTTTTTTTTTTTAGTCGCGAACATAATCATAATCATAACAAAAAGAATCCAAAACAAAACTTGTCAACAAAATTGGTTTATGATTGCAAAATATAAATGCAGCCTATCCTGAAGATACCACCCTACACCTTAATAGCATACTTTCTCATTGGAAAGTACATTTCTCTCTTCTTCTCACGTTCGGTCTTTAAAGATTGCTGGTGCTTAGTAAGCCTCCTGCGGATAGCCCTGGTCTTCTTAGGACGCAGGTCAAGGGGCAAGAACTTCTTGTTCTTGTATGCTTCTCTAAGTGCAGCCTTCTGCTTTTGAGAAATCACAGTCAAGACCTGAGCGATAGACAACCTCACCACCTTGATCTTGGAGAGCTTGTTAGGAGCACCCCCGGTGACCTTAGCAACGCGAAGCAAAGCGAGCTCAGCCTTGAGATCCTTCGACTGATTCA

>L0748

ACCCCAGCTTATGTGGAGCAGAATCCGAAGTTCAGATTTCCTAGAGATGTAAACAGTAATACTGTGATGCATTGAAGAATTGTGGAAACATTTGGTGGGTTGTACTCCAAATGATCAAGCCGTTGCAATAAAATCCACAACCCGCACATGTCAGGCTAGTTTGATCTGTTCAAAATAATTATTCAGAATACAGAAACAGTGATTGGTATGCAGCATAGCACATCATACCTTTTTAGGTAATTG

>L0749

CCGGGCTGCAGGAATTCGAATTCGAATTCGGGAAAAGAGATGTTGCAACAGCGAATGTCATATAAGATGCTTGATACTTCTCATAAAGGTGGTGTATCTCAAGGGCCGGAGACTGTTTCTGATGCAACTGCTCTCACGTTTGCCGCATTGGTGTCTCATGCTCAGCAGCAGGACCTTGATAAAGTAAGGCATAGCAACGACCTTGCTCAAGTTTCTTCTAAAAGAAATATTGAATCCTTAAGCCATTCACATAATGACCTCCGCAATTACTCTCTTTTGCACCAAGTGCAGGCGATGAATAGTGGTGCAGACAGTGCTTTGGATGTTCAACGTGCTGCTGCTTTGGGGGGACAGCATTTACATGATATAATTTCAAGGTTCAGACACCCTGTGGATGGTAAATCAAATTCAACATCACAAACCAGATCATTTCCATCTGGTGAATAACAAGTGCTTGGCTTCTTGGGAGATGC

>L0750

CCGGGCTGCAGGAATTCGAATCCGCTTACTCTATACTGCAAGTTAGGGAGGTAGATGGACATAAGCTTGTTCAAATTCGGAATCCATGGGCTAATGAAGTTGAGTGGAATGGTCCTTGGTCTGATTCATCACCCGAATGGACTGATAGAATAAAACACAAGCTGAAGCATGTTCCTCAGTCAAAAGATGGTATTTTCTGGATGTCTTGGCAAGACTTCCAGATCCACTTCCGATCAATATATGTATGTCGTGTCTACCCCCCTGAGATGCGGTATTCTGTTCATGGCCAATGGCGAGGCTACAGTGCTGGTGGATGCCAAGATTATGCTTCTTGGAATCAAAATCCACAGTTTCGATTGAGGGCTGCTGGTCACGATGCATCATTACCAATTCATGTGTATATTACCTTAACTCAGGGGGT

>L0751

ATTCCAAAACTGTATGTACTGCAGGCGAACATGAAAAGTAAAAACAAAATAAGAAAACAAGGGAAAGTTTTGTTTGTAGAAGATAAGTTTACCATATGGGTAGAGCAAAACCCTGTTGATATAAGGAGAAATTGGAATATTGCACACACATACAGAAACAGAAATCTTTTATAATCAAAGCTCTCTGAGTTGCCTTAGATTCATAGGAGATATATATCTTTTATCTCCATCCAGCAGTTGCTTGGCAAGTAGAATGTTTGCAGCCTCACTGGGATGGTAAGGATCCCAGAACACATGCGTGGACCGATCTTGGCACATACTTGAGGTAGGTCCACATGGAATTATCCCTGCAAATTGCCCTCCATTTCCACAGCAAGCTCTTGTTGCGGTTGTAAATCCTGCATACACATATGTGATCTTGTTTTAATACCA

>L0752

ATCCGTTTTTTTTTTTTTTTTTTTTTTTAAATCTCATAAAATTTTTTCTTTTCATTTATTAATATGAAAACTTGAATTCATACAGTCCCTGACAGGCTTAATACTACTACTAGCACATGCGGAGTCCTAAATTTACAAATTTAGAGAATAATAATACAATTAAGTAATCTTTTCATAACCTGAGAGGAAAGGGACAGGTTATTCTGAAAAATGTCTCCTCCTGTAACAGTACACCGTCGACTATTGTGGCTGCTGGATGCAAGAAAATAGTTGATCTCGATCACCCGAAGTTTATCTGTGT

>L0753

AATTGGATTTACTTGAAGGGAGGTTAGGGATTTTCAGGCTAAAGCAAGGGGAGTTAGCTATGAGAAGAAGCGAAAAAGGCCTAAACCAAAAGCAACATCAACACCTCAAGCAGCAGCACCACCACCTGATTCAGGCGCGGGCTAGTGAGAGAGAGTTAAAATATGAACAAGGAGAAGCTTGCATTGAAGCTATATGATGCGGGCTCATTATCACCAGCAGCTTTATCTATATGGGTATTATTTTTCTGCTCTTCCTTTATTTTTATCCTATAAAGAGATGGTTATATAATGAACGTTGCTTTGCTTTGCTTTCTAAGAGCATATGTTTGGATTTGTGGGTACAATTGGAACTACTCTTTAAGGGACAAACCGCCTGGCAATGAGCTAGTACCAGTAATCAGTAGTGCTAGTAATAGTGAATCTAGTGATAATAGACTTTTAGCAGAAATTATGATAGCAAGTTATCTTGTAATTGTGGTCATATATGGAATTTGTACTTTGAA

>L0754

ATTCTTTTTGGCTATAATATTCGACCTGTTGCTGTTATAAAATAAAAAAAGGAATGTTTAGTTGTGTAGGTTGCAAAAGTTTTCTCTGGATGATAATTGAACTTTCTTGTTCACATTCCAAGAGTTTCCCTATCATTCTCAACAAATTTGAAACACAATGATGATTCATCACAACAGTAATCTAGCGGAGAAGATGTTGAATTGCAGCAACAGCATCACCTTTATGCTCCCTCAAGGTTCTCTCTGCAACCTTCTTGTCCAACTCTAGTTCATTGGCAATTATGTCAACATCAGCAGCATTGATCTTAACAGCAGCCAATTCTTTCTCCCTCATTCTCATAGCATTCCAATCGGCTTCTGCAGAAGCAGCAATGGATGCCATAGCCTCTTGAACACGAGTGGAATCACGCTGGCGATCTTCGACACGATCAGTGAGCTTATCAAGAGCTTTGCTTTGTTGC

>L0755

ATTCCATCCTTCACCAGGCAGGCCTCAGAGGACGAAAATGGCTTCTCAAACGGACTCAACTCCGATCTATGTGGATCCAATTTTCCACATCCTCAAAATCATCCCTTTCTCCGTCCTCCGCCCTCCCCGCCTCCGCCTCAAGCTTCCTAACCTTACTCTCCCCTCCCCGATGACCGTCTTTGCTCTAGTCCTCCTCACCTACTTCATGGTCGTCTCCGGCATCGTCTACGACGTAATTGTGGAGCCGCCAGGCATAGGCTCTACTCAGGATCCGGCAACCGGCTCCGTTCGCCCTGTTGTCTTCCTACCTGGCCGTGTCAACGGGCAGTACATCATTGAGGGTTTGTCCTCTGGGTTTATGTTTGTGCTTGGTGGAATTGGAATTGTGCTTATGGATCTTGCGCTGGACAAGAATCGTGCTAAGAGCGTAAAGGTTTCATATGCCTCCGCTGGGATTTCATCGCTGATCATCGCCTATGTAATGAGTATGCTCTTTATTCGCA

>L0756

ATTTTTTTTTTTTTTTTTGAACCAGCAAATATGTGCCTGGCTTTATTGATTACGAAATTATTATCTTCATCATTACCACCACGCTTACTTTCATAATTCCATTATTGCAAACACCAGAGGCAAACAAAACGTACGTGCATGCTTATAGAGCAAAAGAGCAAGAAATTAAAACCAAGAGGGCAAAGAGACTTCAGCCTCTAATTATGGTAGGGTGTGTAATTTGATGCTTGATTTCCCAAAGCTCAAAGATTCCTTCAACAAAGTTATAGTAGCCACCTCTCAGTGCTAGTTTTCCCTTTGCCATTGCTGCCCTAACATATGGGTAGCTCTGTATGTTTACTAGTGACAGATTCACTGCTTCCTTTTCACAAATATGGCATTGTTCTTCATGTGAGAGATTTGGATGCTCTGCTCGGACCTTGGCTTTGGCAGGCAAACCAATTTTGACCCAATCATCTATGAAGTCATTGGAAGTAGAACCGTCCTCTGGAAGACTCATAAGTGTATCTATCCCACCACAGTGGCTATGTCCAATGATCAGGATGTTTTCCACTTCTAAATATTTGACAGCATATTCAATGACTGCTCCAACTCCAGAGTACCTCAACTGGTTAAATGCTGGA

>L0757

TGGAGCCAATTCTTTTTTTTTTTTTTTTCTAAACCATTAATTCATATTAACTATCAAGCAAGCAATATAGATTGAAAGGTAAAGTAAAATCCAAATTGACCATTAAACTGCAAAAATTATCATTCAACGTAGCCAAATAGACCATAAAAAATAACAGACATGATAGCAAGTTCAACAACGAGGTCAAAAGAAGATAATACTCAAAACGAAATTTCTCTATGCTTCCTCAGGACGATACTTAAGATCAGTCACCGCCGGCCTTCTGGTAAACGTAAGTGAGGCCACACTTACCGCAGTAGTGCCTGTCAAAATGATTGGCCATAAAAGTACCGGCGCCACATTCGGCGTTGGGACACTCCTTCCTCAGCCTCTGCACTTTACCACTATCATCAACCTTGTAGAACTGAAGAATGGAGAGCTTGACCTTCTTCTTCTTGTGCTTGATCTTCTTGGGCTTGGTGTAGGTCTTCTTCTTCCTCTTCTTGGCTCCACCCCTCAAG

>L0758

ATTCCTCCATCAATCGACCAAAACGAGTGTGAGACTCTGAGTCGCATCTTTTCTCAGAATACGCGATTCGCTGCCATCGCTGACCGACTTCCTTCAGATCCTTCATCAAAACTGCTAAGGTTACGGATACAGAGAATCGGAGATGGGGCTGACCTTTACGAAGCTATTTAGCCGGCTTTTTGCCAAGAGGGAGATGCGAATTCTGATGGTGGGTCTTGATGCCGCCGGTAAGACTACTATTTTGTACAAGCTCAAGCTTGGAGAAATTGTCACCACCATCCCTACCATTGGATTTAATGTGGAGACAGTGGAATATAAGAACATCAGTTTCACTGTTTGGGATGTTGGTGGTCAAGACAAGATTCGCCCTCTGTGGAGGCATTACTTCCAGAACACTCAGGGTCTTATTTTTGTGGTGGATAGTAATGACAGAGATCGAATTGTTGAGGCAAGGGATGAGTTGCATAGAATGCTGAATGAGGATGAACTGAGAGATGCTGTCTTGCTTGTTTTTGCTAACAAACAAGATCTTCCCAATGCCATGAATGCTGCAGAAATAACTGACAAGCTTGGGCTTCATTCTCTCCGTCAACGCCACTGGTACATCCAGAGCACGTGTGCGACATCTGGAGAGGGTCTTTATGAGGGGCTGGAGTGGCTGTC

>L0759

AATTCTTTTTTTTTTGCCAAAAACACCATGCAATTTGAATATTATTGATGGAAAGCATTCAAGTACTTGCATAAAGCTCAAGTGCTACATCATTATCAAAGCAAATCTAGAGAGAAAGAGAGAGAGAGAGCTAGAGCTATTTACAGATAAGGTCAAGGAACTTTAAGGTGCCCAGCATGTCCCATAGACTTCCAGGATGATTCGAGCCTCAGCAGGTGCAATTTCTCAACAAATGGTTCCAGAACTTCTGCCTCTTCTTCTGGAGCAAAGGCCATACAGATGCTAAGCTCTTGCACTGCCTCTCCATATTCCCTATTATAGTACAGCATCATCCCGTGATCTCTTCTCCAGGCCCAATTATGTGGCTGCAAAATCAGCAATCTTTCTGAAGCCATAATGGCCAGCCTAAGATCTCGTGGCCGAAGCAAAGGAACATTTGAATTGGAGGTCATGTTATGTTTCTCATCAGCATTATGAACATGTCTAAGTGGAGAAG

>L0760

AATTCCTTTGGTTTTCCGTAGAGCAGAGACGCTCCAAAATCACCTACAAAACTTGGCCTTCGAGACTCTTACACATCCTTTCTCAATCAGTTTCCCTCTTTCAGTAATATTTTTAAGACTTTGTGAAATGGGTGGGCTGCCTTTGCAAAATGCTTCCTCTACCTTAGCTCTGCCAGGTTCCGAGCAAACTAAGGGTTCAGCAATTACATCATCTGTAGCAGAATCAAAGCCAAAAAAGAAGATATGCTGTGCCTGCCCTGAAACTAAGAAGCTGAGAGATGAATGCATTGTGGAGCATGGTGAAACTGCTTGTGCAAAATGGATTGAGGCTCATCGTCAGTGCCTTCGAGCTGAGGGTTTTAATGTTTGAAATTGATTTAGATGGTTGAGTTGATAAAATGATACCATTTTGGGCATAAGATACTTGCTTTTGGAACTACACACAATAGTTGAGGAATAAAGAAAGAAAAAGGCAACTTTTCTTGACTACTTATGTTTCAAATTGTGCAATTTATTGAAATTATGAGGCAGTCTTAACAATTCAAAATATAATGAGTGCATAGATGCAACCTTAAAAAAAAAAAAAAAAA

>L0761

AATTCGTGAATTCGGGGAAGTGATTGCAATTTGCGTTTGTGAGAGAGAGAGAGAGTCTAATTCGCAAAGATGGCAATGGCAGCAGAAGATGATGAGTTCTACCTTCGATACTACGTGGGACACAAGGGGAAATTCGGCCACGAGTTTCTTGAGTTCGAGTTCAGGCCAGATGGCAAGCTTCGTTATGCCAACAACTCCAATTACAAGAACGACACCATGATCCGCAAGGAAGTCTTCCTCACCCCT

>L0762

AATTCTTTTTTTTTTTTTTTTTTTTTTTTTTAATTACAATAGATCACTGTTATGCATTAACTTCAAAGAAATTGAAAGTCAATCCCAAATTCTGACCATAACAAGCCTAAATGTTTTTATATGTATATTGCTATTAAAAATTATACAGTAAAACCAACGAGCTTTGGCTCAATATACTGTGGTCTCCAGGATTGATGTCTTAAGATCAAACTCTAATCAGTTCCAGAAGTTGCTCATCCCAATAAATTATATTTACAATGAAAAGCATCCTTCCAGCTAAAAAACATTAATCACTGCAGCACAACATCAGAGAAGCTGTTGCTTCACTTCCTGTATGAGAATTCAGTTTCAAACTCCTTTTGAAACCGTTCCTGAGACTCAGTTTTCGCAGCCAACACAACATCATCAAGAAATCCTCTTTGTGGAAATTCATCAGGCACGAGACTATGATATGTCTCAAACTGTTCTGCAGCTTCCTTTTTTCTATCCAATAGACTGTAAATTATTCCCTGGCAAAGATAAGGCCGAAAAT

>L0763

AATTCCTGAGTTTTTTTTTTTTTTTTTGTTTTTTTTTTTTTCATCCCAAATGGACACACATGAAATTAAAAATGTCTATTTAATGAATCACTGAAATACATCCACATTGCTAATGCATATTAAAAAAGTGCACATAAGAGAAATTAAAAATACAAAATACAAATGCATCAATTACTTTTTCTTCCCTGCTTTCCCTTCCTTTAATCTTATAACTTCATTGGCATACCTCACACCTTTACCTTTATATGGCTCAGGAGGCCTCCATTTCCTAACCGAAGCAGCAAACTGGCCAATGTTACACTTGTCATATCCACTAATAATGATCCTCGTGTTCTCTTCCACCTTCACTTTTAGGTCACCAGGTATTTCCATTCTAACTGGATGAGAAAATCCCAGACTTAACACCAAGTCTTTTCCTTCTACTGTTGCACGATACCCAACGCCAACTAGCTGAAGTCTCTTCTCAAACCCTTTTGATACTCCCACCACCATGTTGTCTGTAAGTGTCCTGAAAAGTCCATGCATTTGGTTGGCTCTTCTTGTGTCCAATGCCTTTTTAACACGAATAATTCCATTCTCATCTCTCTC

>L0764

ATTTTTTTTTTTTTTTTTTTAAATGCAACAAACATTACATTCACTGCAGAATATATGGTTCAGCTAAAAAAATTAATAACTGTAATGCACTCAAGATTATATTACACTTTTGACTACCTCCTGTTTCAGTTCAACAAAAGCAAAACAATCTAAGTTCCCTTGCAGGCCAAAGAAGCAGAAAAGAAAGAGCAGCAGATACTGTCAAACTTTCAGATAATTTTCTTCCCCTTTTTCTGTAGTTTCATCACTGGCCTTCAATAATTACTGTACAACCAAAACAGAACCATGCTAATAGCTAAGACCTGCCACAACCACTATCCTTGAACTCTTTCAACAGAGGATAACAGTACCAACACTATACATCCTAAAACCGCCGCCTCACACGCCTGGCGTTGGGATTCACTGATTCTGCTGCAGCCCCATCTGCCACTCCTAGATCGAGCCCATATATGCTGCTACTACTCCTCTTCCGACCTGATAGCAGACTTGAGTTCTCATCTCCTGATGCAAATGTCTTGAGAGAGAGAGAACTAGTGCGCGCTCACTGGCCGT

>L0765

AATTCCTCTGCCCTTTCAAATACGCAGACCAGAAAATCCATTTGACAAAATGGAAGCCCTAATTTGGTAGAAGCCTGATTCAGTGATTCCTACAAATTTTCAAGTAGGAAACTCTAAAATCGAATGCTTTCCAATTACTATTCAGGAACGAGTGGATGCTTAATGCAGCCTCATTATCAGGGAGACCAACACCACGCGACTTCCTTCCGGAAGATGATAGGTCTGCCAGGAAGCCTCAGCCGCATCAAAATTGGCAAGGCTACTGAAGATAGCGAGAAGAAGATTCAAATGACTAGCCAGTCTTCACCAATGTAGCCATGTAACCAGGCTATGAATGATCTAGTTAAGGAACCTGACCATTTTTGAAGGATGCATTTAAGAATAGAGAATATCAACGAGGAAGAACTGCTAAAGGGTTCAACATGTAAGAATGTCTTATTCATATAATTTTTATACTTCTGCTGATTAAGATTTGTGATCATTTTGTACATTTTGGTCATGAAGGCAACAAATGTTCACTGTACTTTGCAAAATGCTGTTATCCCAAGCTCTTGTTCTGTTGGCATACAAACACAATACCTTTTTTAGA

>L0766

TGGAGCCAGCAGGATTTGAGTTATTTTAAAAGCCAAATAAATACACTCTATAGGTCTCATATAAAAATATGAACTTGCTGGTCTGGAATAAAAGAGAAATGAAAGGAAAAACGCAATTACAATAAATACATATCAGTGACAAGTCAGATTGTTCACCAATCTATATCCAATTCCCTAGATAGGAAGAGTTTGTCAATGTAGAGAATGACTGGTGCAGATGCAGCAATATAATACTCCATTTGAATCCTGGCTGAGGTCTCATCTTCATTCAGCTTATTTAAAAGTTGTGCTTTTGTAGGAAGGGCATACATCCCTGCAGCAACAGCTTTTCTAATAGCCCACCCATGGTGAGGAGCAAATACTTGTGCATAAGCCTTGGAAGCTGGGTCCTTCAAAGAATTCCCCTCTGTAACAATAATTTGCTCAAAGAGAACCTTGACCATGTCAAGCCCACGCTTTACCCTCAAAAGATTTCTTGAATGACTACCCGCTTTTCTCACGGTTTTCTGTTCCATGTCCCTATCCAGCATGGCTTCCAAGGTCACAATCACTTTCGATGCGTCTGCAAGATCACGAACCTTAGCAACATAATCCATCTCGGCGAACTTGAAGGCGATACCCAAACAG

>L0767

ATTCCTTATATGCAACCAAATCAAAGCAAGGACTCGCTTCTAAAACCCACTGTGCCATTAACCCAGCTCGCACATGTGCCTCTCTTGCTGCCTGGCACTCGTCCGCTAAAAACTGAGGCAGGACGAAAGTTGTATGGTTGTTCTTATTGTCATCACAACCGGTGCGTTACTGATCAGAAAGGTTCTCCTTGTAGTAACTGCGGTTGTTCAATGACTTATGAACTGCCGTTTATTGGCACCAATACAAATAATAAATCAGCTAGTAATACAGCAACTAGTAATAGTGAGGGAGGTTTTGTGAAGGGCCTGGTTACATACATGGTGACTGATGATTTGTCAGTGTCACCATTGTCAATGATCTCTGGTGTTGCTCTGCTGAACAAGTTTAGTGTAAAGGGTTTTGGTGCACTTAAGGAGAAAATGGTTGAGTTTGGCATTGATGAGGGTGTGGAATTGCTGAAGGCTTCTCTCCAGTCAAAAACAGCTCTTACTAGTGTCTTCCTTGCCAAGAATGAGATCAAGGATGCTGCAGCACCAAATTGAAACTGTGTTGGATATATGATCTATTCTAATGCTAATCAACGC

>L0768

AGAAAGGTTGGCCATAGGCCCAGTTTGTCTGGGCAGAATACATTCCCCTTAACACCCACTCACCCACCCCCAATGCCAGATCTTCATGGCTATTGTCTAATATTGGCTTTCTGTGAGAGGGAGAGTTTCTGAGTTATACAGAGAAGGTTCTACAGAGACAGAACCAATAAAAAAACCAATACCAATTTTCAACATTTTTGTCATTGTTTTATTAAATTAACGCAAAGAGAGAGATCGCCATTTCTTTCTTCCTTCGTCTTGTTTTTCTGCTTGGTCTGTTCATCCTCTGTAACCCTTTTTTGTTGTTTGGTCTTTTGGGTTTCTCAAATTTGCCTTCGAGATATTGTTTTTTGTCGATTTGTGCCCCTTTAATGGCTACGCTTTCTGTTCTCTTGTTTTATATTGGCGCTTTGTCTTCGATTCCATTCCTGTCAGCCTCGGAATCTATTTGCCCTGATGAATGTGCTTTCTTTTTATTTAAACTTAAATCTCAATGCCCTCTTTCGATCTCTTCATATCCCCCT

>L0769

AATTCCCTACATGTTACCCAATATCGGCTCCGGTGACTCGTTCGGAATCTAAGCTGAGTTCTCTAACTCGGGAGCTGCTGGATTCCGCGAGAGAGCCCGAGTTCTTCGATTGGTTAAAAAGGGTTAGGAGGAAAATCCATGAGTACCCAGAGCTGGCCTTTGAAGAATACAACACGAGTGAAATCATAAGATCGGAGCTTCAGTCGCTTGGAATTGAGTATATTTGGCCTATCGCGAAGACTGGGGTGGTGGGTTCGATTGGGTCAGGGATGGAGCCATGGTTCGGTCTTAGAGCAGACATGGACGCCCTTCCCATTCAGGAATTGGTGGAGTGGGAGTACAAGAGCAAGAACAATGGCAAAATGCACGCTTGTGGCCATGACGCTCATGTCACAATGCTGCTTGGAGCAGCAAAGTTACTTCAACGCGGGAAGGATGAATTAAAGGGTACTGTCAAGCTTGTCTTCCAACCTGCAGAAGAAGGTAGTGCTGGTGCTTACCATATGTTGAAAGAAGGAGCTCTTGATAAGTTCCAAGCCATTTTTGGACTCCATGTTGAACCAGAAATGCCTGTTGGTTCAATTGCTTCTAGACCTGTTACAATGGCTGCTGGTTCTGCTAGGTTCTTCGCTGTCATTAAAGGGAAGGGAGGACATGCTGCA

>L0770

ATTCCTCAAGCTCGCTTTTACACAGATCGCCCTCCATTCACAGTCTCGCTTCTACACAGACGGCCCTCAAGCTCTCGGCCCGCTTCTCACAGGTGCCAGCAGCCACAAAATTTGGCATGGGATTAGCTGACAGGGCAGTTGGATTTTTGTTATCATTCATCAGTTTATCTATCTTTACTTATTATACTTTCTGGGTCATTATCCTGCCATTTGTAGATAGTGATCACTTCATCCACCAGTATTTCCCGCCACTAGAATATGCCATACTGATACCTGTATTTGCCGGTGTCACGCTTCTGTTCTTTCTATGCGTTTTTATTGGAATGGTGATGCTTAAATCCAAAAAGAAGAAGCCATAATGCTAATTCTCTTGTCATTATCATGTATTCTACTACATTCTGGAATTCCAAACCTTTTTTCTCACTTAATATGATTTTGATAGCGAGTGCAGTTGTCAGTATGAGTTGCTTTCTAACATTTCTAATGATTTGCTACTTTAAATTTGAAG

>L0771

AATTCGTTTTTTTTTTTTTTTTTATTATTTTTTTTTTTCACATAAAATCGTGATATAGGAATCTCTATTGCAGTAAAGGTTTACTAGCCTCGTATTTAGTACAGATCAACCACAAACAAGCATGAAAATAAGCAAACTAATCGAGCTGATAAAAACCAGATCCGAAAATCACCCATCATCTTCATTTACAATAAAGCCTGGAGATTTGAAGTAGTGAAGTTAAAGGCATTAAGCAAAACAGACAAAACAACACTAAGTTTGCATGGATTCAGCATGATAGTTCTCCAATTCCTTGTCAAGATCATCTGCTGACTTCTCAAAAGGTTGCTTCTTCCCATGGCCTCGGCCTCGGCCTCGGCTTCGCCCACCAGGCCGACCTCGCCCACCAGGCCGACCTCTACCATTTCTCAAACCCCCGCGACGGTTCTGACCAGAGTTAGTTGGAGGAGCAGTGCCTCTCATGCGACCTGGTCCAGACGTCATGACAACTGTCCTCTTCCTCCTTCCATTTACTCCAGTCACATTAACTCTAGCTGAGATAGGCATTTCTGCAGTGGTACCCACAATTTCAATTTTCATGGGCTTTCCATCTAACAGCACATTGTTATACTTCTTAAGAGCTGCAAACGCATCACTTCTTCTTGTGTACATTACTTCAGC

>L0772

AATTCCCCACTTGCCGCTCCTCATGCCATGTCCGCTCTCCGCCATCGCACTCCACCTGGACCGAACCGGTCTAACCGGGCACAAGAACCGGAAGAGGAGCACTACAACATCATACCCATCCACAACCTCTTAGCCGACCACCCCTCACTACGGTATCCTGAGGTGCGAGCAGCCGCTGCTGCTTTGCGCACTGTCGGGAACCTACGGAAGCCGCCGTATTCCCAATGGCACCCTTCTATGGACCTGCTGGATTGGCTGGCTCTTTTCTTTGGGTTTCAGAAGGACAACGTTAGGAACCAGCGGGAGCATATAGTCCTTCACCTTGCCAACGCCCAGATGCGCCTCACTCCTCCTCCTGACAACATCGACACTCTTGACGCTACCGTGCTCCGGAGATTCCGCCGGAAATTGCTGAAAAACTACACCAACTGGTGCTCTTATCTCAATAAGAAATCCAACATCTGGATCTCTGACCGGTCACATCCCGATCTCCGTCG

>L0773

AATTCCTCGCTCTTCTCACTCACTTACTCAGCATCCTCAAAATGGAGCTGTGTACTACTCAATCCCTCTCTAATCTTCCCAAAATTTTCACCGCAAACCCTACTCTCCTCCTCTCCAAAACCCCCCTAACTCTACGACAACCACAAATCCTTCTTTCCGGCCGCCGCCATTCCGTATTTCTCTCAGTACCAAGAGCAACGACACCTGAGGAATCTTCGAGCCAAGCAAGTCTTTATGCCATCGGAGAACGAGACAGTGCAGTGGTAGTAGAAGTTGACCCTCCAAGCGAAAAGAAAGTGTACAATGAGAGCAGCGCACTCCTGTAGATGAACAAGAAAATGATTTTTTGGCAAATATTAAGGTTTGTTTGGGAGGATAGTTTTTCCATTTTATGGTACTTGGAGATGCCAATTCTTTTACTTTAACAATTTTGCCTGTTGTACTTGGGATGATAACTCTGATTTAATTAACTGCATTTTGATGTCAAAGTACTGAATTCATGGCT

>L0774

CAATTCGTTTTTTTTTTTTTTTTTCAATAAACAATCATGACCTTAAAGATGGCTTCAAGTTCCACAAATGTCATACATATCAGTTAAGTTGTTCCATAGCAAACCGTAAGTTCCATTGCCCATTAAATAGCGAAAAACACTAAGGTTAGAATGAACCAACGAAATAAAAGCCTAAAGAAACATCTAAAAGTGGCAAAAAGCACTTCCAATTAACATCAACTGACTCATGAATCCATTACCGATCCCGAGGATCATACATCATCTGGAGCAAAAAATCCTTGCGCTCATTGACTGGGAAGCCAAAAAAGGCTCTTACCCTTGCAGGATCTTGAGTAAGGAAAGTGTAGGCTGTGTATCTTAAGTGCCGCTCAACTCCTATCTTCACAAGTTCTGCATAAACTTCTCTCTCTGAGTAAACATGCTGTCTAGCTCTCTCAATAATAGCATTTCCCTCCCTGATTGCCTCTGCAACATCATTAATAGCATCCTTGATGCTCTTAAATTTCCTCTCCCATGTACGGGCCTTTCGCTTTCTGTCTTTCGGTAACATTACAGGCTCATCACATTGTGTGTGGTAATACACAAGGAGCATACAGATGGCATGTACAACATG

>L0775

ATTCCCTGGCACTTGAACCTGGTCTCTGTTCCAATCCAAGCCATGCTGCTCTTGACTATAAGATTTCCTCTCTGATCCTGTCTTGGATGACTTAGCAACACTAGAACTTTTCCCTTCCCTTCCAATGACCAGAGGAATACGTTTATCATCAGAATGCAGAAGTCTGGATTGGCGAAGCTGAAAAACATTTTCATCCTCAGCCAGCCCACTTTCTTTGTGAAATTGCAACAACCTAGTACACCTTGTAAGAATGAAAAGCATTCGGGTATGAAGCTGCTTCAGCATTCCTGGAGCAAGTTCTTGGCGCCTATCATCCAATTCTTGGACTATGCCTTCACACTGAAGCCAGAATTCACTGGGTGATGTCATAGCACAACTACGAGCTAAAACCAACAAGTCCTCAATGGTTTCCTGCCATTCAGGATGACTTTCTGCATTTTTTTCAAGGACTCCAACCAGATCCGCTGCAAAGATAGCTAAATCAGAATTCACTTCTTCCTTTGCTTTGTCAAATTTTGCCCGTATCACAACCAAGATTTCCTCCAAGTTATTTAGCGCGCGCTCACTGGCCGTC

>L0776

TCTCACCATCCAACCATCCATTCTCTTCTTCCTCCATAACATTCCAAAACCCATCTGTACCAGACCCCAAATTCAGAACCATTTCAGATTTTGTATAGTTCTTCAGCTGTAAGTTGTGTGATTTTTTAGTACGAAAGAGCTCAGACTGCACCTGCGATAGAGAAAAATGTGTTATGTGGGGAAAGCAACGAAGATCTTTATTTTCTTAGTGACAGTTCTTGTGGTTCTTGGTCTTACCTTGGGATTCGGGTTTCTTCGCCATAAATCTCACAAATGCTCCGGTGATTCTTGCCATTCTTCTCAACCCATTACTTTCCCCAATCCAAATACTCCTGTTAATCCATCTCCACCTTATCCGAACCCTGTTTCTACCGGTACCCAGCAGCCTACCCCTCCAATCTCCGGATCCAACCAGCCAAGCCCACCGCAATTTAGCCCCCCTACCTCCAACCCAACTCCTCCTCCTCCACCTGATACTAGTCTTAATACTCCTCCGCCGTCTCCGTTGTTGTTGCCGTCTCCGCCGCCGCCAATTGGGCCATCTATAACCGGGGCGCCACCGCCGTCGTACAGTCCGCCTACTAAGGCGGTGTTGGTGACTCCAGGTCCTGTGCATGCTTAGTCGCT

>L0777

ATTCGTTTTTTTTTTTTTTTTNTGGCTTAAAGCAAGATATAAATTAAGACAAGTATTCATAATTATTATACAAATGAAAATAAATATTTAAATTAGTCATTTGCTATAACCAGGTCTTCGAATATGTCATCTCAGTCGAAGAACTTAATCTTGTCCCCTTTCTTGCTACTCTTCTATATCACCTTGTGCATCTTGGGGTTGGCCAGGTTGCCCTTGTTGCTGTCTGGATGATTCCCCCCTGCAGTATAATGCATAGCACCTTATTCATAGTGTCCAAATAGACCTGAATGGTTTTGCCAACACTGAGAGGAGCAGCTTCGGCGAGGATAAGGTCGCCAAGCTCGGCGCGCTTCAGATGGCTGATGCTAAGGTGAATCCCAGCTACCCTTCGAAATCCGGATGCCATATGGGCCCCCATGCTGGCCAAAGCCTCCGATATCAGAGCCGATACTCCGCCGTGCAACACCTTGAATGGCTGA

>L0778

ACAAACATCTGCGAGACTTAGGAATCTTTTTCATAAATCTCTTCTCCATGGATTTTAGCCAGCCTGCAGGCGAAAACTATGCCAATCCAAAGACATGCTTCTTTCATGTGCTCTTCAAGGCTGCAGCTCTGGCATTCTACATACTCTCAGCTCTGTTTTTTAATAACTTTGTTATTATCTTTGTGGTAACCGTTGTTCTTGCTGCCCTTGATTTTTGGGTGGTGAAGAATGTGAGCGGCCGGATCCTAGTTGGGCTTAGGTGGTGGAATGAGATAAATGAGGAGGGTGAGAGTGTATGGAAGTTTGAATGTCTTGATCAACAGTCGTTGGCTCGTATGAATAAGAAGGATTCATGGCTATTTTGGTGGACATTATACCTTACTGCAGCTGCTTGGATCGTACTTGGAATATTTTCAATTATAAGGTTCGAGGTTGATTATGTCCTGGTAGTGGGAGTTTGTTTGAGCCTCAGCATTGCAAACATTATTGGGTTCACCAAATGTCGCAAAGATGCCAAGAAGCAGATTCAAGCATTTGCCTCCCAGACTATTGCTTCTCATTTCTCATCTACCATACAGTCTGC

>L0779

AATTCATCGAAGAAGCTAGAAGCCAAATCACCGGCGACACCGACTCCGGCACCGGCGGAAGTTAAACAAGGTGCGTCACAAGAGAAAGTTATTGTTCCACCACCTGAAGAATCCATAGCTCTCGCTGTAGTCGATAAGAATCCAGAATCTGTTCCAAAGAAGATTTCAGGGGGATCCCATGACAGAGACACTGCTCTTGCAGAGGTTGAAAAGGAAAAGAAAAATTCCTTTATCAAGGCATGGGAAGATAGCGAGAAAACTAAAGCAGAGAATAAGGCTCAGAAAAAGCTCTCCGCTGTTACCGCCTGGGAGAACAGCAAGAAGGCTGCATTGGAAGCCAAGTTGAGAAAGATTGAGGAAAAATTGGA

>L0780

ATTTTGATATGGTAAACAAACAAATGATTTCATTTTATCAGGTCTCATCGAAGTAGAAAAAAAACTCATCAATTCCAGAAATATTGCACACTATCTACATGTAATGCTTGCGAAAACATCCCAAACTTTTCTGGCAAATGTGACTGTCAAATGAGTAAAAGAGAATGAAACTAAATAAACATAAAATTAAACATGAAAATTTAATTCCCTGCTACGTATGCACTTCCCATCAAAACAATGGAGCAATGGATTGCAAATATTCTTCCAAACCAATGCTCAATGTACCACAACCTAGAGTAAGAATACTTCTTCTGTTCCAACATCTGCTCACCGTATATTCCCTTTGATTCCAGCATTTCCTCACTATGATTTCAACTGGATATATCAGCTCACCTTAACTCCGAGCCTTCCCATTTCCGAGTGTGAGCTCCAGATCATCCAATCCTACCTCATGAATCCTCTCTCCTTCCCATGGCTTCACCTGTCCGCTGCCAAACTCAAATTCCATACTCCTCCCATTCTCTTTGATCACATTGCTGGACAAAATTTGCTCAGCCACAGGTTTCACAAGATTGT

>L0781

AATTCTTTTTTTTTTTTTTTTTTTTTTTTTTTTTTTTTTTTTTTTTAGCCCAAACTTGAGATGCTAATAGAAGCAAAGCAAAAACCAACTCCTGATGCATACAAGAATTTTTGTCTCTCTCCTAAAATATATATAAGAAACAACAGGCCAATAAAAGCCTGTGGTACAGACTTTTTCAGTCGAAATTCAGAATTTATGTAACTGGAATCTCAATATTGGCTGTCATCACTGGTGGTGGAATATACTCTTCTTCTTCTTTGGGAGGATGGATAGTGACCAAATCAGGCAACGGTGTCATTGGACCCACCTTGCCTTTAGGGTCCCAGTCAAGCATGATCTTGACCTTGATACCAAGCACACCCTGTCTTAAAAGAACATGCCTCACAGCAGAGTCAATATATTCATTGACAGGTTGACCAGAGGATATCATGTACCCATCCTTGAATTTCATAGACTTGGCACGCTGAGCTCGGAGCTTTCCACTCACAATAACCTCGCAACCCTTTGCCCCACTCTCCATTATAAATCTCAAGACACCGTAGCAAGCCCTCCGAACAGC

>L0782

AATTCGTTTTTTTTTTTTTTTTTTGCCAGGAATAAAACTGTAAATTTAAAGCCTGCGTTCTCCATAAACTACGCAAAATTTGATAAACAAGCACATAAACTTTTATGGGAAAAAACAATACACATTTAAGGTATTGGACACGTTCTAAAAGGAAAAGAGGATGCTGAAAGAGAGTACTGCTGCAAGATAAAACTAGATATGCAATCTGATTAGTGATCACCAGGGAGGCTCTTCATGATATCAGAATCACCTGGGTCAATAATACTCAAGCAAGAAACACGGAAATACTTGCCACAAGCAGTTCCCAAGTCAATATTGTTTCCATTGTAATGGTGGACGCCAACCTTTGCAAGCATGGCATAATACTCAATCTCGGATTTTCTAAGCGGTGGGCAATTGTTTGAAATTATAATGAGTTTTCCTTTAAAACTTCTGAGAGAGCGAAGCACGGTTTTGTAGCCGAGAGTGAACCTTCCACTCTTCATCACCAGAGCAAGCCTGTTGTTGATGCTCTCATGGGTCTTCTTAGTTTTCTTGCCAACAGCCATTTTCGCAGTGGAAGAAAGCT

>L0783

ATCGAGTTTTTTTTTTTTTTTTTTTTTTTTTTTTTTTTTTTTTTTTTTTTTAGTTAAAATCATTAAACACAGAATAAGGGTGAATAAAATCAAATCAGTTAATCATACATTTAAAGATTAAATTACATAACCTGACAGTTTAGCACAGGATTAGTGGGGAAAAAGGCAAACTTTAAACCTTTGGTAGGATTTGTCATGAACAAAGCATTTTAACACATACACCACATAGAAGGTAATAATACTAATACTACTTCGTTTGCAGCTGGGACATTACTGTCTGTTCCTCTTAAGGCTGCAAGATGGGCACTTGTATTGCTTGATACTCTCAGCCTTAGCAGGTGTTATCTTCACGCACTTTCCATGGAACCACCTCTCACAGATGTCACAGCCAATCCAAAATTCATCTGCGCTATAACTGCCACCACAACTCCCACATAGGGTTTCGTTATGTTCATCTTCATCAGCCTCATAAACATCTTCTGTAAGCTTAGGATTGCTCTTCACTGCTTGAACATCGCTTGATCTCTTTACACTGCCTCTTGATTTGCTTCCACTATCTGCACTGGGCTTTTCTTTTACAGGCTTCCTTCAAAACTAGT

>L0784

AATTCGTTTTTTTTTTTTTTTTTCTCTGAACATTCAAATCGTGGGAGAATTTGCATTTGAAACCTTTAGCACACTGCCCCACTTTATAGAACTCGCACAATATAGACTTGGGATCAACACCAATAGGAACTTTGGGCTGGCTAACAGCAACCTTGAACAAATCGTTCAGCTCCTTCTCTTTGGCCTTGTCTTCCTCTTTCTTCTTCTTAGCTGCGACCTTAGTAGGGTCGGGTTTGGGCTGTACAGATTGCTTGAGACTCTGTACATATTTCTGTACATTCTTG

>L0785

ATTCTTTTTTTTTTTTGCTTTTAACATACTGATTTATTACTCAAAATTCTAAACGTAAAAGCAAGTGTGTAACAGAACCATACTACATTGAGAAAGTAATACTAAAATTACAAACAACCTTCACCAAGTACATGCATCCAAACAAACATCGCTCATTCTTGGCATCTCCATAAGTATTCATTTCCCATATTTTTAATCATGCAGCTACATTTTAATATTACTCATTGGCTTCAGCACCCGGAGGAGTTTCCACAGCCCCATGAGCTGATGGACCATCTTCTGCAGGCTTGGAAACATCTGCTTCTTTAGTTTCTGCAGGTTCGCCAGCAGAAGCAGCTTCGCCAGCAGAAGCAGCTTCATCAGTTACTGGAACAGACTTCTTAATAGTTCCGACTTTAACATACTTGCTCATGAACTTGTACTCCCAATCCTGCAAGGCATCCAACTCGAATGGACCAAGACCAGAGATATCACCAGTCAAATCTTTGTCTTCAAAGGACATCTTTGCCAGCGCTCTGCTGGCATCCTTTCCCGCAAATAATGCATAAGGTCCACCAGGTCCATAAAACATCCTGCTCTGAGAGATGTCATAGATCTGACCCTTAATAGCCATGAGCAGAGGCTTCTTAGGATCAGAGCCATCATATTGC

>L0786

ATTCGATAGAAATGCCTGCACACGCAAATATATATATCCATCAAATAGATTTTCAAATTTTACACGAAAATATCCCCAGTAGTTATTGCCAATGTGAAGGGTTAAAAGAAAAGAAATCTATTAGAAGAAATAAGTTCAACCTCCAATTAACAATCGAATCAAATCACGGGATCAAGTCAAAGCAGATTCTGGCACCACAACTCCTTTTAAATATACACATGGACTACACCAAGTAGTCATCTAACCGCGTATTGACGCCCGAGGGACAGCAGGTAAACCTAGTTCATCTTCAGCCTGGGCATTATTTCTACCCACTGGTGCTGCTGCATGTCCTATTGGTGCCGAGGGCAAGTTGATTTCTGATTCCAGATCAGGTTCCTTATCAGGTTGGAGATAAGAAGGTACACCATCAGATTCAGTTTCCATTCCCATGTCTGCCTCCAATGCATCCAGTTCTCCCATGAGTTCATCCTCATCAATATCATCTGGAACATTGTAGCTTCTACCCAGGGTCTCTTGGATTTCGTTACTCACATCCATCAAGTCCATCATCTCATCTTGCAAGGTATCTATGTCTTGGATCTTCACAGTTTTCATCATTCCTTTCAACTCCTTG

>L0787

GGAGCCATTTTTTTTTTTTTTTTAGTTAAAATTTAACTACTGGTATTAAAGCTCAAAAAATAGCACACGTTGACGATTTTCATTAAAAATAAAAAAAAACGAAAATACAAAAACAAAACAAATCAAAAGCAACATTCGTAGAGAAAATGGCAAAACCGAGTTCAACTTTATACGTGGCACAAGCTAACGTGTAAAGCATGCCATATGGCCTATCTATTTCAATAAATCACTGCTCTGATTGCATAAAAGAGTTGATTTGCTGTACTATGTCCACCCCAAGGACCTCCTCCTCGTGTTTGGGGGTGTGAAGCTAAGCCCAAACATCTCCTTGAGAGCTTGCCCCTGTTCTACAAATCTTACCAGCCTTGCAACTATTGCAGCACTCTCCATGATGTGGTTAATATCCTTTGTGTTGCTCCACAAGCTGTTCGCCAACTGTAGCCTCCTACGTTTCGAGTTCAACCCTATACCCCACTTCTGGTAGATTCTCTTTCTCTCTACTTCTGATAACCTCTTCTGCATCAGCTTGCTTAGCATTCCTCTCTCCTTCTGAAGGGCCTTGATGCTGTCCCCCCTTGTTGCTAATGTTAATGTCCAACCAGCTCCCACAGAATGATTT

>L0788

AATTCCAGGCTAGGGATTCTGAAATCCCTCCTAGTGGTGCTACAAGTTCTTCAGCTTCTAAACTGCCTCAGCAGCAACCAAATTACCAAATCCCTGCCTCAGCTCTATATGAAGCCACTGATGAGCCTGTATCGGTGACTTCAGCACAAACAACATCAGCAGGAAGAACTGTTACTTTGGATATTGCAGCTGCAGGTGAATCTAGTGCTCCAGAAGGTACTGATCCCATAGCAATGATGGAATTCTACATGAAGAAGGCTGCTCAAGAAGAGAGGAGGAGGCAGCCTAAACAGTCAAAGGATGAAATGCCCCCGCCTCCTTCCCTTCAAGGACCTCCAGGAAAAAGAGGTCATCACATGGGTGACTACATTCCACCAGAAGAGCTTGAGAAGTTTTTGGCTAGCTGTAATGATGTAACTGCACAAAAAGCTGCTAGAGAAACTATAGAAAGGTCCAAAATCCAAGCTAATAATGTTGGGCATAAGTTGTTGTCCAAAATGGGTTGGAAAGAAGACATGTTCCCACAGGTGAGGGACTGGGGAG

>L0789

GGATCCCCGGGCTGCAGGTTTTTTTTTTTTTTTTTTTTTTTTTTTTTTTTTTTTTTTTTTTTTTTTTTTTTTTTTTTTTTTTTTTTTTTTTTTTTTTTCCAAAAAACCAAAAACCTTCTTGATTCATAAAAATCCCTTTTTTTATTTTCTTTCACTAAAAACCCATTACTTTTCCCCCTTTGAACCAACTCTTTTTCAAAATTTGGATTTTAATTGAAAATTGAAACAACTTAAACAATATTGGCCTTTAAAAAAATTTTTTTTCCCCACGGGACACAATTCTTTCCAACCTTTTTTTTTTTTTAAAAACCTTCCCCCTTTTTAATAAAACCCCCCTGTTATCCTTTGTTCTTTTTTAAACCCCCCTTTCCCCCCCTGCTTTTAATCCCCCAAATTGGGCTTGGGGGTTCTTCAACCTTTTTTCCACCTTGAATCTTCCCCCACCCAATAAAACCCCATTGCTTCTCAACTCTTCAAGGGATTGCAATCTTCTCCCCCTTGCCCTTGCACAAAGGAAATTTTAACTGAACCTTTGCAAAATTTTTCTTTACTGCAAAAACTCGAGGGGGGGC

>L0790

AATTCTCGGTTTTTTTTTTTTTTTTTTTTTTTTAGTGCAAAATTTCGAGGTGAATTGAAACAATTGAATCATCAGGTTTCTCAATTCTGCTACAGTGTTTGGACTTGATCTTATATATAAGAACAGAAGAGGTTACTGCAGCCCCTAAGCACAACTAAGTATTGGCGAGTAACAGAACCCAAAGCAACTGCTGAAAACAGAGAACAAATTCAAATAATCCTACCCCTACAAAGTTCAGCCAAGAATGGGGAAGATTCGAGTGATGTGCTCTAATGGTATACCATCTGCAGTATTTTTCTTCAGCTCAGGATGGTTAAATTCATTGCTGGGTAGAACAATGAGTTGGCCCTTAACATGCCCTTTCTCTACACTCCAACCACAATTGGTTATCTGCTGTTCAAGGAGTTTGTCCAAGGACAGACCTTCAATGTTGATAGCCTCAGCCAGCACAGACCGGGGAACCTTTTGATAAGTCAAGGAAAGCAGATGAATAGCATATGATTGGATTGCTAGCTCAAAACCTGGCACTGCTTCAACTATGTGGCGACTCTTAGCTGCTTCATCCCAGAATTGGCAGAACCTCCC

>L0791

GGGCTGCAGGAATTCGAATTCCTCCACTGAACAACTTCAGATAAACCATAACACAGAGAGAAAGCGCTTTTGGGATTTGGGGTTTCAGTTAAAAAAGCTAAAAAATGGAGATCGCAGAGAAGAAGGAAACAGAAAACAATAATAATAATAACAATAATAATGCTCAAACGGCATTGGATTCAGGATCATCTGATAATATCGATAAAGAAGCCGAAGAACGCCAAGCACGTGACCTCAAAGCTGGTTTGCATCCTCTTAAGCACAAGTTTGTATTTTGGTACACTCGCCGAACACCAGGAATTCGAACACAAACTTCATATGAGGATAATATAAAGAAAATTGTGGAGTTCAGTACAGTTGAAGGTTTTTGGGTCTGCTATTGCCACTTGGCTCGATCGTCTTCTTTGCCTAGTCCTACTGATCTGCATCTTTTCAAGGAGGGAATCCGTCCTCTATGGGAGGATTCTGCAAACTCCAATGGTGGCAAGTGGATAATACGATTCAAAAAGGTTGTCTCAGGTCGCTTTTGGGAGGACTTGGTGCTTGCCTTAGTGGGTGACCAACTGGATTACGGTGATAACATATGTGGTGCAGTACTAAGCATTCGTTTTAATGAGGATATACTG

>L0792

GCTGCAGGAATTCGAATTCATATTATGGAGGGTTATGCATACATACTTACACATCCAGGAATTCCAACAGTTTTCTATGACCATGTTTATGACTTGGGTGACTCCATTCATGACCAAATTGTGAAACTGATTGACATTAGAAAGCGCCAAGACATCAATAGTCGATCATCTATCAGGATTCTTGAAGCACAGCCAAATCTCTATTCTGCAATTATTGGGGAGAAAGTATGCATGAAACTTGGGGATGGTTCCTGGTGCCCAACTGGCAGGGAATGGACACTGGCAGCCTCCGGCCATCGATATGCTGTGTGGCAAAATGATGCCCCTTCATAATTAGTCACCAGTCAGTTTTGGTAATCTATTTCTGTGGACTTCTGTTTGGTAGAGGTCATGTCTTGCAGCTGGTATTACAATAGTAACTATTTTATGAATAATTTTGCTATTTGTATGAATAATTTGGGATCATCCAAGTTATATTAGAACTCCACGAATCTTTGCTGCCAAAAAAAAAAAAAAAAAAAACTCGG

>L0793

AAAAAAAAAAATCACCCTCTGCAATCAAGAAATGACGAATTGTATGTGTTCTTGTAGCAAGCCTCTGATTGTGGACAAACCCATTCACAATGTGAGCTTCTCTATATCTAACGAGAGGCTGCCAATCAATTTGGGGGTTTTACCTTCTGCGATAACCGCCGCCATCGCCACTTCTAGGGTTTTTCGGGTTAGGGCTTCAATGGTGGATTCATATGAGAGTTCCTCTAATTTCGCTAAGCGCATGGAGCAAGCTTGGTTAATTTCACAGCAACCAAGACCAGTTGCGTGCACTTCTTGCAGCTCAAATGGGCATGTTGAATGCAAATGGTGTGCAGGTACAGGCTTCTTCATTCTTGGTGATAATATGCTCTGTCAAGTCCCCT

>L0794

AATTCGTTTTTTTTTTTTTTTTTCGATCATGAACATCTAGCTGACTATCATTGCATCACATTCAACATCCATAATTATACTAATATTATAGTGATAATTAAAACCATGCAGAATTTAAATGGAGTATTAAAAGTGGAAAACTCTCGATAGATACATACACGATACAGGACTTCGGATCTTATTGATGTCCTCGTCCTCGAACTCGATGTAACCGCCCGCAGCACCTTCATCCTCATCATCGATCCCACCGGCAACCCGTCGTTCACCCCAATGTTATCAGGCAACTCATTATAAGCCTTCAAGAGCCAAGCCTCATCTAGCATGTACTTGAGGATGACATCAGCTTTATCATCCTGCTAATCCCAAGGATGACGAGGATTATATCACCGGCGGCGATCCAAACTTTCATGTGCATCTTCCCTAGGATGTGGCAGAGGCGTTTGGAGCTTCGCAGCGGCTGTTGCCCAGCATGCGGAGGAACTAAGCATATTCTTGACCGTCTCCTTTGAATATGAGCTCTCACTTCTCATCGTCGGCCTCGTTGTTTCCTCTCTTGCGATTCTTGCCTCCCTTTCCCTTATTCTTCGGCATCTTGAATGAGAGAACTATT

>L0795

ATCTTTTTTTTTTTTTTTTTTTTTTTTTTTTTTTTTTTTTTTTTTTTTTTTTTTTTTTTTTTTTTTTTTTCTTTTTTTTTTTTTTTTTTTTTTTAACGGGGGACTTTTCCTATTTTAAATAATTAAAAAACGGTTTTACCCTCCCCCCCTTTCTACCCGGGATTACAAAAACTTTTTGATCAAATATTTAATTTACAAAATTACCCCTAAAACTGTTCAAGCCCTTTCATAATCTCTGCCTTGCAATTTGTTTTATAATAACCACTTGGGTTACTTTTACAAAAATCCCCATTTTTTTTCCCCCGGTCAAGGTCCTCAACACAAATTCTTTTCTTTTCGGGGCTTCCTGTTGGACAAAAAATTTTCGGAGGCCCCACAATTTGACC

>L0796

ACTCGAGTTTTATTTTTTTTTTTTTTTTTTTTTTTTTGAAGCCTAAGCTTGTGGATAGCGTTTGAATTGAATAGGTTTAAAGTTGTTTGCTCACCTGGGCTCAATGAACAGACCAATAATTACCCTCTAAAAATATCTGAGAGCCAATTCCTAGGTCCTAAATTATTATTTATTATTTAAGAGAGACTTTAGGTAGCTAGGGAGAGACACAAAGCATACAACTTCATTCCTATTGAAACATACATATTCATAAAGCATGAAGGAAGCACCCTACAAATAACAGAGAAGTGAATCAATGATACTATGGAGATGGAGGCCAACCATATAATTCAGCATACTCAATTCCTTCATAGAACCTGAACTCAAGCTCCTCCAGCCTTTGCTCATCATCCCACATTTCATCAATACGAAGCAAAGCGCTCAGCAGCGTCTAGTAGATCCTCTAGTAGATGAGGCCCCAAAACTCACTTTTGGGAACTTCTCCTTGAGGAAGTTGCTATCAAGTTTCACATTCCAGCATCCTGTCAAATTCACAAACTCGAGGGGGGGCCCGGT

>L0797

AATTCCTTTCTTCTCTCAATCCCCTTCCTGTAGGCAAGCTATTCATGGTTGATAATGGCTTTCGGTAGTCATTCTAGGATCGGTTTGGCAGCTGAATCCCCTGATTAGGGTGAAGAACTGGCTATGGATGCGGTCAAATTGATGCAATCATTGCTTTATCGAGCTGTTATGAAGTTCGGAGCTCGTAATAATTGCCAGAGAGCAATTTGGCCACCTTATCTCTTTATCCTGGCAGTGATTTTGCAGCTACTTGGAGAAAAAAGAATTGCTATAGTTGGTGGTGAAACGATTTAGGGGTTTCACAACTTCTGACCTGGTGCTTCTTTTCATATCTTTGTCCATGTCTGAACTCCAGGGAGCAACTATTTCGTGCTTCCATTTTCTTTCTGCTGATTTTAATGTTTCTCATATCCCAAATGGATTAC

>L0798

AATTCGCTGCTTCCTTATAGCGTCCCCTCACATGGGCCTGTATCTTAACAATTCTTTGCCGAATTATCAGGAATTCTTTTCTCTTTTTCCAACCACGGAACTTCTTCTGGATTTGAATAGCTGCAGCATTGACTTGTCCATCAGAATGCATAGGCTTGTGTGTCTTGGCGGCTATAAGTGCAAGAGCACGATCATCCAACGTATTGAAATCATCATCACCATACTCAGTTAACTGTTTCCGCTGGAATGATTGCATCCTGAATACTTGATGTAAACGATCAGCTGCTTGTGTAGCATTACGAATGGCAGTCAGTGAATCCTTCAGTGACAGCACATTAGGCACATCACCATCATTCACAGGAGTGGCCATCCGTTCTGCAATTGTTTGCACAGCCATCATCCCTGAAATGTCTGGTGCACCACCTTCCTTTGGATCATTTAGTG

>L0799

AATTCCACCAAGCTTCCTCTTATTGTTTGGTTCCACGGAGGAGGGTTCATACTTTTCAGCGCAGCTTCAACCTTTTTCCACGATTATTGTGCAAGCCTTGCTATTGAGCTCACTGCTGTTGTCGTTTCCATAGAGTATCGCCTCGCCCCCGAGCACCGTCTTCCAGCTGCCTATGAGGACGCTGTGGAGGCATTACACTGGATTAAATCAAGCCCTGATGAGTGGTTGAGAGACTTTGCTGATCTCAGAAAATGTTTCCTTATGGGGGCTAGCGCCGGCGCTAATATAGCTTACCATGCTGGACTACGAGTAGCTGAGACAGTCGAACATCTAGAGCCCTTAAAGATTGAAGGGCTAATATTGCACCAACCCTTCTTCGGTGGGTCAAAGAGGACTGAATCGGAATTGAGGATGATGAACGATCCCATTTTACCACTTTGTTCCAATGATCTGATGTGGGAATTAGCTTTGCCAATTGGTGCTGACCGTGATCATGAGTTTTGCAATCCAACGGTGAAGGGAGAT

>L0800

GGAGCCATGATTATGTGTTGGCCCTGACTGTGGAGAACTTTCTTGAGCGCCGTCTCCAAACTCTCGTGTTCAAGTCGGGTATGGCTAAGTCAATCCATCATGCCAGAGTGCTCATCAGGCAAAGGCACATCAGGGTTGGGAGACAAGTGGTTAATATTCCCTCATTTATGGTGAGGGTGGACTCGCAAAAGCATATTGATTTCTCACTTACAAGTCCCTTTGGTGGTGGGCGGCCTGGTAGAGTGAAGAGAAAGAATCAAAGGGCTGCTAACAAGAAGAGTTCTGGTGGCGATGGGGATGAAGAAGATGAAGAGTGAGCTTTTCCCGTTCTGAAATAGTGATTAATGTNTCTTTTTGGAAGTATGCCTAGAAGTTTTTCTTATTGATCTTTCATTTCATTTTGTCAACCTATGATGATTTTTTTGAGAATCTTTAGCAAGCAATTTCAATTCCTTTTCTGTTCTTTCTTCTTAACTTTGTTGATGTTGATGGAATACATATTTACTTGTTCAACT

>L0801

AATTCCATCAGCAGCTGATGAAGCAATGGCAATGATTTTACGGCTTCAAAGCGAGAAGAGCTCCATGGAGATTGAAGCCAATCAGCAACGTAGATTGGCAGAGCGTAAGCTAGAATACGATCAAGAAATGATTGAATCACTGCAATGGATTCTAATGAAACATGAGTCTGAAAGGACTGTGCTAGAGGAAAAGTTGAGATGGTGTGAAGAAAAGTTGAAGCAGTATATGAAGAGTGGTGAAGTTGATCAATTTCAAGGGTCTTATGCCAGTTCGAGTTTCGATGTTGAATCCGCTATGGAAGAGGGTTTTGAAGACGTGCAAACAGACTTGGTTGAAGAGGACTTCGCACTTTTGGCATGTCAAGGTGAGGGAACCTAAACTTATGGT

>L0802

AATTCTTTTTTTTTTTTTCTCAAAATGGCAATTTAATTAACGTCGAGCAATTTTTCAATTGGGGGAGCTTAGCATACAGTCTGCAATCAGAAAATAAAATTAAAAAATAATAATAATAATAATAAAAGGAAGATCACAAGGGACCACCCAAAAAAAAAAAGGGGATGAATTAGGCCAACACAGGTACTAAAGCCCAGTCTGCCAAATCAGTTGGTCCCAATTCAACATGCGCAGCCACCTCTTTGCTCGCTCTCGGAGACGGCCTCGGGGATGCCTTGGAAGTATATGTCTTGTTCATGCTCATTGGCTAGAGCAGTTTTCGCAATACACAGGAATGCAGGATCAACATTATAATCCTCTTTTGCTGATGTCTCAAAGTAAGGTATGTTTCCTTTCGAAGCACACCACTCCTTTGCTTTCTTCTCAGAAACCACTCGACTGTTTCCCCCATCAATGTCAATCTTGTTTCCAAGCAATATAAATGGAAACGTCTTGGGATCAGGTGGGTTTGCCTAGAAAGAGCAGGCAACTATTAGATCCGAGAGAGAGAGAGAGAACTAGT

>L0803

GGCTGCAGGCGAAACCTCACCAGACGATAAGACAAACCAGAGCAAAACCAAAAAGGAAACGAAGTGAAGGAGGGCATCAAAGCCTTGTGAAAACGCAGGGGGCAAAAACAAAGGAAAGCGAAGAGAAGAAACTGAAGGAAAATGGGGTGGATCGGAGATACTGTCGATTCCATCAAATCTATCCAGATCCGTCAGCTTCTCACTCAGGCTGTCAGTCTAGGAATGATTGTTACATCTGCGCTAATAATATGGAAGGCGTTGATGTGCATCACTGGTAGTGAGTCTCCTGTGGTGGTTGTTCTATCTGGAAGTATGGAACCTGGCTTTAAGAGGGGTGATATTTTGTTTTTGCACATGAGTAAAGATCCTATTCGTGCAGGGGAGATTGTTGTTTTCAATGTAGATGGCCGCGAAATCCCAATTGTCCATCGTGTAATCAAGGTCCATGAAAGGCAAGATACTGGGGAAGTTGAGGTCCTCACAAAAGGAGATAATAATTATGGGGATGACAGGCTTTTGTATGCTCAAGGTCAACTGTGGCTGCAGCGGCACCATATCATGGGGAGAGCTGTTGGGTTC

>L0804

AATTCTGCTTATCTGCTGATTGCTAGTAATTGCGGCTGCGCCGGCCTTGCTCTAGACCTTTGGTTAAAATTCTAACCTAGGTTTAGAGGAGACCTCTCTCGCCAATTTCATTTATTTTCCCCTTTTCCATCTTCAATTTTAATTTTGATCTCGGTATATGTCAGTTTGGCATTTGGGCAGAGCATTGCATGTCGGTGGCGTGGCGTTGGAGGAGTTTCTGTACTTCTATTTCAGGAGGACAACGGCGCCTTTCAGCTTTTAATACTAATTACCGATGGCGGCTAAGAAGGTCTGCCACTCCTTTTCCAAGACCCTCATAGACGAGGTCCACAGATGGGGTTGCATGAAGCAGACTGGTGTTAGCTTGCGCTACATGATGGAGTTTGGCTCCAGGCCTACCAATAGGAATTTGTTGATTTCTGCTCAGTTCCTTCATAAGGAGTTGCCCATTAGGATTGCTAGGAGAGCTATTGAG

>L0805

AATTCGTTTTTTTTTTTTTTTTTTTTTTTTTTTTTAGTATCATCAAACTAACTCATTCAACTATATCTTGAGATCATAGAGACATACAAACCCATCACAACGAGGAAACATAAAAAAAAAAAAAAAAAAGGCTTGTTGCTCAAACAATGTGACAGTGTCACCTCAAAATATGATAATTAAATCTTACTCTAAAAATCCTAAAGATAATACATAGACAAAGTAAGTGCATGCATATTTTACAATAATATGGCAGTTATAGCTTACAAAAAAATCTCCTCCATGCATTACAAGGGTGAGATCATTGAAAGCCGTAAAGAACTTTCCTAGGATCCAACTGCCCACTGCAATAACTTGATGACACATTAAAGAGTTCTTGGTGGCCCTTCCACTCTTCAAAAGGTTTCAAGACAATTGGGGTTTCAATAGCAGCAGAATCCACACATATCATTGTTTTATATTCCTCATCTCCCAAATCTTGGAGAGCCTTGGCCTTTTTGTCCCAAGGATTCCACAGAACTGCATCAGGCATGCCATCCTTCCGCAGCACAAAGGTTCTCTTCTTCTCATGGTCAATAATAGCAATCTTTGTAGGGGTGCTCAAATATACCCTATCAATCTCGGCATCAAA

>L0806

AGGGCACTGTTTTCATAGGGAAAGCTTCAGAGGTGGCTGAGCAAACCTCCATGTATATATAACAACTTTACGGCCATGCTTCTTGAAACCCATATACCTTCCTTCTTCAACCTTATTAAATTATAATTAATTCACTTGATCTCCAGTCTTGAACAAGGTGAAAAGGAATCAACAAGAATGCGTTCAACCGGCGAAACTCAGATGACTCCTACTCAGGTATCAGATGAAGAAGCGATTTTGTTTGCTATGCAACTAGCTAGCGCATCAGTGCTGCCTATGATGCTGAAATCAGCCATTGAGCTCGACCTACTTGAAATCATGGCTAAAGCAGGTCCTGGTGCATTCTTGTCGCCATCCGACATCGCTTCTCAGCTACCCACCACGAACCCAGATGCGCCGGTGATGTTGGACCGTATCTTGCGACTCTTGGCCAGCTACTCCGTTCTTACTTATTCTCTGAGAACCCTTCCTGATGGCAAAGTTGAGAGGCTCTATGGTCTTGCTCCTGTTTGCAAATTCTTGACCAAGAATGAAGATGGTGTGTCTATTGCCGCTCTTTGTCTCACGAACCAGGATAAAGTCCTCATGGAAAGCTGGTAATCTTTCTCTGCAACTCT

>L0807

ACTCGGCATTGGTGGAGGGATTGCTGCAGTGTCGAACTGGTCTTCGAATCTTATAGTTAGTGAAACATATTTAACTCTGACTGAGCATCTTGGAGCTGGTGGTACCTTTTTCCTATTTGCTGCAATTTCCTGCGTTGGACTTGCATGCATCTTCTGGTTTGTTCCTGAAACCAAAGGCCTACAATTTGAGGAGGTTGAGAAAATCTTAGAGGAAGGATACAGGCCAGATTTGTGTGGAGGAAAGAAAAAGAGGAAGAATGTCGATACTGTATAATTCTGAAGAAGCTTGAGCTGAAAGTGAGAGACAAAAATTGCAGATTTGCAGTACCTATTACAATTCCTATAATTATTGTTAATAGTATTCCGTGTTTTTAATGAGGAAGGAATCCTATTGCTCTCATCAAGTAAAAAGATAGTGCCTTGTGCAAATTCAGTCCTATTTAATGACTGAATTATGTAAAGAATAAGGTTCTCATTGGGTTTAAAGTAATGAACAATTGTTTCCTTATTGGCAATTAATGGAAAAGCTTTCTTTTCGTATT

>L0808

GCTGCATGAATTCGAATTCCTCTTTCCCTTATGGGACTTCTCCCATGATAATAATCTCTTTCATCAACCCCATTGTCTAAACCATCAATTTGTTCGTTCTCCTCTTTCTCTTCCTCCTCTTCAAAATCATCTTCCAACACACTTTTTCTTGGCTCAAGTGAACCAATTGATTCAAGAGTCCTTCTTTTCTTAATCCGCGGTAAAGCAATATCACAGGAATAATCTTTCGTCAGAAGCTCATCTATAACCTCATCCACATGTGTTAAGGCGAATTTTCCATCAGCTAGCTTTTGCCTTAATTTTCGATAGTCGTTGTATAAAGGCTCTAGGTACCGGTACACATCCACATCAGTCCCAGTGAGACGCAGATAAAAAGCACCAAGTATTCGCACATATTTGTAATCGTCGTTTTTTATGAACTCGACGACGATGTCCTTCTCGGGCTGGATTTGCAGCATCTTCATGACCAAGCACATAAAAGGTGTAGGTTTGCGGTTGCCACCATAAGTACCACCAAGATGGTCGAGCTCCATAGCCTTGTCCACAAGCGTCTCCGCTGTTAATCCGAAGCATTGCTCCTTCCAGT

>L0809

ATTCAACCAGCTTCAGGGATCTTACCATAACCCATCTTCTTAGCGCTAAGATCATCCTCTTCATCTCCATCCTCGCCATCAAATTCCTCATAGCGGTAATCGTAGCGGGTAGAGAAGAGGGACCAGACCAAGTACATGGTAGCGGCGGTAAGAGCGCCACAACCAACGCCAAAAAGCAAAGCGACCACGACACTGAGAATGTCCTTGGTGCGATCACGGAGAGAACTCATGTCATAGGAAGAAAGCCCAAAAGGCAAGACAGGAGCTTCTTGCGGCTTTTGGATAGCTTTGGGTTGAGAATCTTGATCATCACCATCATCATCAACGGTGGGGAAAACCCGGCGATCGAGGAGGATCTCAGAGGACGAACGTTGTTGAGTAATTTCGGTGACGATGGTGAAGAATCCGGAGGAGGAAGGGTGATGAGAGTTAGGGTTAATGGGCTTGATGGAGAAGGAGT

>L0810

AATTCCCTCTGTTTCTGATAAAGGCATCGCCGTTTCGCTCTGTAAGATACACTCTAGCAACAATGAAGACTACCAAGGGTAAAGGGGCAGCAAAGGTCACAAAGGAAGCCTTGAAGCCTGCTGATGACAGAAAGGTTGGAAAGAGGAAGGCAGCAGCTGTTCCTGATAAAATTAGCAAACAAAAAGCTAAGAAGGAGAAAAAGGCGAAGAAAGACCCAAACAAACCAAAGAGGCCTCCTAGTGCTTTCTTTGTCTTCCTTGAGGAGTTTCGAAAGACTTTCAAGAAAGAGAATCCTAATGTGACATCTGTAGCTGCTGTTGGGAAAGCTGGAGGAGAGAAGTGGAAATCAATGTCTCCTGCTGAAAAAGCTCCGTATGAAGCCAAAGCTGCCAAAAAAAAGGATGAGTATGGAAAGCTTATGAATGCCTACAACAAGA

>L0811

AATTCGTTTTTTTTTTTTTTTTTAATAGACAAAATGGCATACGTTTTAATATTAAATTTCTAATATACCGCATATATATATATCTCATTCTACTGATGTTTCATAACCTTACAACCGATGCTTTTTACAAAGTATACAAGACGCACAATGGTTAATGGCCTAAAATCTACTTTCAGAATACGATAAGACATAAAAGAAAGCAGCAGAAGAGGAAAATTGAGATTCTGTTCAATGATTTGTATGCCTGCATTTCCTGCCTTCTTAACCTGGGGCTACTACTTGCCATTGGGTGATATGAATTTGGATATGTATACAAGCTCTCTGAGTTTCCAAAAACCCTTGCATAAACCATCTCACCAAACATCCCAACAAATGGGTGGTGGAATCCAGTCACTCTGGTGCCTCCAAGATTAACTAGAGGTGATGATGATGCCTCTTCAAAACTGCTGTATGGATCAGGATTATAGCTATGATTTTGATAAGGATTATGGTATGGAAGCTGCTGACCAGTTGGAGATGTACTAAATACCAAAGCTTGCGTACCATAAGCTGACGGCCT

>L0812

ATTCTTTTTTAAAAGGTACCGCAGTAACACACCCAACGTCTTCTAAGAAGCCTTCTAAATTTTGAGACCTTCAGCTTCCCAGGACTGTTTTCTGTTTTCTGCGTAATCAGTTGATGTCTTGAGTGTGCAAAAAGAAATCCAGAACTATAAGCACAAGTTCCAGAACCCAACTCTATGAGATAAACTAGTCCACTGCAAATGTAACAGCACACCAACAAATTCATGATTTACAACAGTTCACAGTGCACAGTGCTCAGCACACCTAATAATGAACCTTCTCATCATTGTCAAAGTTCAGCCCCTCGATTCCCTTGAGAGCAGATTCATAGGTCTTCATGTGCGTGGCATTTCTGGCTGATGACGAACGTCTGGGGTCTGCTGCAGAACCAAGAGGACTCCTTTGCCCACTTGAAATTTTATGCATGTTTCCCGGGCTTGCATCAGTTGTGCGAGACCGTTGGGGATCAGCTTCACTTCCAACAAATATATCACGACTGGTAGAAACGGTAGCAGCTTGT

>L0813

ATTCCATCTTCATCTTAAACCCTAGTATTCCCCATCTACAATTTTCTTGCTTTCCTTAGTCCACTTATTGCTCTAGTTTGTACCAACACTGTGAAATAGTTGGGCGGTCAATACACATTGTGAACCTAGATCCTGCTGCAGAACATTTTGACTATCCTGTGGCTATGGATATCAGGGAACTCATTTCTTTGGATGACGTTATGGAGGAGCTTGGTCTTGGTCCCAATGGAGCTCTTATTTATTGCATGGAAGAGCTTGAAGACAATCTGGATGACTGGTTGACGGAAGAGCTGGATAATTACAGGGATGATGACTATCTAGTTTTTGACTGCCCAGTATACAATATGTTTTGGCGCAAATTGACAACTGCATTCAATATGGAG

>L0814

AATTCCATTCTTCCTCTCCTTAAAACCATCTCTATATATTCTTTCAAGCTTACACACATCCTTCATTTTTGCTTTCCAAATGGCCAGTGTTGAGGTTGAATCAGCTGCAACAGCATTGCCAAAGAATGAGACACCTGAGGTGACCAAGGCTGAGGAGACGAAGACCGAAGAACCTGCAGCACCACCTGCCTCTGAGCAAGAAACCGCCGATGCTACACCTGAAAAAGAGGAACCTACAGCTGCTCCCGCAGAACCGGAAGCTCCAGCTCCTGAAACCGAGAAGGCTGAAGAAGTAGAAAAGATTGAGAAGACCGAGGAGCCTGCACCAGAAGCAGATCAAACGACTCCAGAGGAAAAGCCAGCTGAGCCTGAGCCTGTTGCAGAGGAGGAGCCCAAACATGAGACCAAAGAGACTGAAACAGAGGCCCCTGCAGCTCCTGCAGAGGGAGAGAAGCCAGCTGAAGAAGAGAAGCCAATTACTGAAGCAGCAGAGACAGCCACCACAGAAGTTCCGGTGGAAAAAACAGAGGAATAAATAGGTCTATTATGCATATCAAAGAAAGGGGGGGATCG

>L0815

AATTCGTTTTTTTTTTTTTTTTTGGGGGGATAAAAGATCTTATATTGAAAATTATGACTCGATATTGAGTATACTACGTTTACATTTGGAACAAACTATGATCCAACGACAAACACATACTTAACTGCAGAAACTATTAAAATCTAAATTGCAGCAAATTGTATCAAGAAAAAACATTCCATAATACCTCATTCTATTTTGATGGTCGTAGTTCCTACATATTGTATTCACAAAACATGCTCTCTTTTCTTTGAAAGTTGATCTCAGGAACCCAGAAGTTTTTTGATTCCTGCTTTCTGCTCTGATGTTAGCCTTGTTGGGAACTTAATGTTGAACTTGATTCTCAGATTTCCTCTCTTCGCCGGGTCTTTTTGGATTGGCATTCCTTCCCTTGGGATGACTTCCTCATAATTTGGATGAATCACACTGTTGATTGGAATGGACAAATTCCTTCCATCTAAGGTGGTGAGATGGACAGTATAACCTGTCAGCGCTTCAGCTAAAGAGATATTTTGTGTGACTATTAAGTCATTTCCATCACCAGTGAACACGGGGTGGGGCTTCTCATCGACCATG

>L0816

AATTCATTTTCTTAACAAGTTCACAATCAACTTCGCAGCAATAACTGGTAAAGCGTACTGCATCTCTGCAAGGATGACATATGAGCTCTTAAAACGCAATCTGCTCTCTGCTGTTTTTGTGGAGACTGTCTCATCTCGTTTGTTGGCTGGAATTGCCTTCGTCCTCTCAGCAATATATGCAATTGTGGTTTGTGCCATCTTGAAGGGTGCGATCAATCTTGGGGTTGACTCATATCTTGTATCTTTTCTTGCATGGATGCTTTTGATAGTGGTTCTGGGCTTCTTTGTCCGTGTGCTGGACAATGTGATTGACACTATTTATGTATGCTATGCCATAGATAGAGATAGGGGCGAGGTTTACAAACAAGATGTTCATGAGGTTTATATTCACTTACCCATCAGCAGAAACCATAGATCGCTTATTGTTCCGAGAACTCACGATGTATGATAAAAATCCTTGACCTATCAAGTGCTGCCGTTTGTTTGTCTTTAAAAA

>L0817

ATTCTTTTTTTATACTAATGGAATAAAGCATCAAACATTGATCATAGTAACCAGTAAAATGACAAAAACATCCCACAACTTTAAAAGTAAACATAAACGGTATTTGTTAACCTCAAATATCAAATCTGACAAATCTAAAGATTTGCAAATAAAAGCAATATCCAATAAACAAAAATGACAGGAAGTCTACAAAAATTGTAATCCAGAAATAGACCAACTTTCACCTCTCTCTGCAGTCTGCACCTCATTCATCAGGGTATAGAGTTTACATAAATAAGTTGGGCCTGGATGCCTTGTATGTTGTCTTGAGCTTTCTAGTTGGTGGTCTAACCTTCTTGAACACCAATGGAAACTTTATCTTGGAGTTGTGGAACTGCTTGGTGCTCTCCCTCTTGCATAGCTTAGCAGGGATAGTGGCAGTCTTGATAATCTGGATGCAAGGAAACCTGACTCTGTGGCGAGAAGCCATCTCATTGTACATTTGCTCCACAGCACCATTGAGAGTTGTATCACGATATTCCTTGTACATGTNGTGGTAACCTGTTCGGCTTTGGTAACGCAGCCAAATGCCATAGTTCTTGATCTTGGTTGGGTTTTTCTCAAAGATCTCATTGATAGCAAGAACTTGGCCATTGCTCTTCTTGACCTTCTTC

>L0818

GAGCCAATTCTTTTTTTTTTGGCACACAAGATTATAGTGATTACATTTCTCGTAAGAAAAAAATACTATCGCATTAGGTTCTTACTATTGCATTACCTAAGCTTCTCAATGATAATATAGCATTGCATGAATAATAGTTTTCATGCTAGGCCTGAGAATGAAAACTAACACTACACTCTAGCATGAGGGCAGCTGCTGCTCGCTCATGGCCCTAGACGATAAGGACCATCACCCATATAGTGACCATCCATATAGAGAGCTGCACTCTGTGGATGTAGACCATCCATCACCATGAATTGCATGTCTTCTGATGGCTTCCAATGACGTTTTCTTTGATTTATGAACCAGTTATTTATTTGCTTCTGGTCCAAACCCGTTGATTCTGCCAACGCCACCTTCTCTGTCTCCGAAGGATATGGCCATTTGTAGTGCAACTCCCACCAGCTCAGTAGCTTCTGCCTTGCTTCTTTGGGCAGTTTCCCTTTCTTTTTCTTCTTAGAAAGCTCCTGCTTAAGACTGCTTAAATAACCACTATATNTCCTCAATAAGTGGTTCTTCAGTTCTCGGTCCTCAGCCCT

>L0819

ATTCCTGGAAGGTTATGCTGGTGCTGGCCTTTATGATAGTGTTCCAATGGATGAAGAAGAGAAAGTTGTGCTTGATTACTCATCTGATCGATTGATCACCGATGAAAACTTCCAAAAATCAATCCTCTCTAACATTGCTCGCGCAGGAAGTGCAATTGAAGAGCTCTACGGATCTGCACAAGACATTGAAGGTGTGATAAGGGACGGTAAACTCTATGTGGTCCAGACAAGACCCCAGATGTAGTTTCCATATATTTATTTTCTTTGCCTTATTTTTCCCCATCGAATTTTGCTAATTAATTGGAATTGTGCACAACGGCAAATTAAAATAGGGGACATAATATATATGAATAAGCAACAACTGTAAAAGATTTAATAAATTAGAATTTGAGCT

>L0820

ATTCAATTTAGATCAAAGTCTTATTTTTCGTTGTTTTATGCATAAATAGTGTGTATTTATTTTGGTGGGTATCTCTGCTTATAGCCAGAGCACATTAGTGGAGGACGAAGCATCCACATTTGTGCACTCATTAAGGGTTGTGTATACAGTCTTGGCCTACTTTGTAGAGAGGCTGTTTTCATGACTCAAACTGTAACTTGTAGGCTATAAGTGGAGTAACTTTATTGTTACGCCGAAGCCGCCCTCTCTCTCCTTACTCTTTTATCGTTTCCTCACAATCGACCATATCAATAGAAATAAGGCTCACTCGATTAGTTTAATCCGTGGCAGAAGAGTTGAATATTCTTGATAATAGTCAAAGTAAAGTATTTACCTATGGGCCAAGTTGGTGTTTAGTATATTGAAAAGAAGCGAGAATAACACAAATCTTGGATTATAAAAGCTGTGAACATATACTTTGCGCCATAAGCTACGAAATCCAAAAAAAAAAAAAAAAA

>L0821

ATTCGTCCGTACAAATCCACCCACTAAAGGCAGTAAGAACCTTTTTTTTGGGAAGAAAAAAATGAGATCAAATCTCAATTCCTTCTTTCCCTTTTAAAGCTGGGTAGCACAAAACGACGAATCCAAACGCGTTTCATGCTTGTAATGGCAATAGAGATGATAATATCACTGTTATCCTCCATAACAGACCTGACATTTGCAGATTTGTTACACTGATTTTCTCTCTCTTACTCGTATTCACTCAGTTCTGAGTTGAAAACAGCGCTTGGGGGGAGATTCTGATACAGAAGCTCTGATTTTCTGCCATCTTTTTATGTTTGGTTTCTCTGTTGCCGTTTTGGGATTGGGCTGGAAAATTCTCTGTATCTGTAATCACTGGATCTGATGGTTTTTTTCAAGTGGTACTGACATTTCGTTACTGTTAGTGGGAGAATCTGAGTTTCTGCTTTTCTGGGTTCTGCTTGGTTTTGAGTGTGCCCAATTTGCTGCTCTTTTTACAGCTTCTGAAAAAT

>L0822

TGGAGCCAATTCGTTCTTCTCGTGTCCGGGAGTTCCATGGAATGAGCCGCCGCTTGGTGAAAGCAATGCCAAGCCTAATATTCACAGCTGTTCTAGAGGATAACTGTACTTCAAGAACATGTGCCATATGCCTTGAAGACTATAGCGTTGGAGAGAAGCTCAGGATTCTGCCATGTCAACACAAATTCCATGCTTTTTGTGTCGACTCCTGGCTTACCACATGGAGAACCTTTTGCCCAGTATGCAAGCGTGATGCAAGAACTAGCAATGGTGATCTGCCAGCCTCAGAATCCACACCATTACTTTCATCCAGCCCAGCCTCTATGGCTTCTTCTTCTGTGTTGTCATCCTTTAGATCTACATCATCGCTGGCCATACAAATTGCCCCTACATCATTGCAGTCCCCATCAGTTTCTCATATTCCATCATCACGATCCCCTTCAGTTTCCCGTATTCCTCCTCTTTCTAGCACTCCATATGTCCAGCAGTCTCTTAGGTCATACCATCAATCACCTTCTCTAAGTGTAAGTCGCAGCTCAGCAGATCTTAGGCATGCCTCTTCGCAAAGATCCATTTCACCCCACTCCTTGGGTTACCCCTCAATATCACCTCTTAA

>L0824

ATTCAGAAGATCTTGATACCTTAAGCCTCAAAGAGCTTCAGAGTGTGGAGCAGCAGATTGATTCTGCTCTTAAGCACATAAGGTCAAGAAAGAACCAATTGATGTATGAATCCATTGCAGAGTTGCAGAAGAAGAGCAAGGCATTGCAGGAGCAAAACAACCAATTTGCAAAGAAGGTCAAGGAGAAGGAGAAGGAAATAGCTCAGCAGAATCAAAAGGAGCAACAAAATCATATTATAGATTCATCTACTGTTCTACCACCACCAATGCAGTCTTTGAACATAAGAGGCAATAGAGATGAAGATGAAACAACTCCAATGCAAAATCTAGCCAATGCTGTCTTGCCATCTTGGATGATTCCCTACCTGAACGAGTAAACAAAGGAAGACGAATGAAAATTTTGATGCGTTGCACGTAGTAAGATGTGGGGAAGGGTCCATTGTGGCAGACTCCTATTTATTATTAATGCAGTCAAGTAAATATATTTTCAATATGTTTATGCTCTCCCGATATAGCATTATCCATATATAAGTTTCAAGTATGTTTAATTG

>L0825

AATTCCATAAGGAAGGAGCAGTGGCCCTGTGGTTGCATTCTTTGAACCACAGAAACTACCCTGATCTCCGTGTTAGCAACTATGGAGATACCAATGGTGAGATGGAGAACTTGGCTAAGGAAATATGACTTATTGATTGATAAACAGATTCAATTTGGAACACAAATTGAAAGGGATATGGATTTTTTTGGAGCACAAGGGTATGGAAATTGTATTTAGTTTTGACGTGTTATGCAAAATCAAATACTAAGCAGACGTTGGTTGTTTATGTGACATCTTATTAAACTCTTTGAGTATCCCTTGCGGGAACTGTTTATAGTTGATTGGCATTGTACTAGCTTTGGAGTTCAAAAGAAAAAAAGAAAAACAAAAAAAGGCTACCTAATTAGAATTCATATGTTTGGTTG

>L0826

ATTCCCAAGAAGGGCCAGAAGGTTGCCATTAAGATTGTTGGCTCCAACCCTGAGGAGCAGCAAAAAATGTTTGGCAGACATTTTGAGATTAATGATTTACTTATCAGCCACGTTACAAGGAGATCAATTGATGTTCTGAAGACTAATTATCGGGATGATCTGTCTATGGAGGAGTGGAAGCTGGTGGTCAAACTGAAGAACATTTTCAAGATACCTTAGATATAAGGTAGAAGGATTGGCTGGGTGTGCTTTCACAAATACTTCCAAGGTTGTATTGGTTGTCTAATGTAAAGATGCAGAGGTTTTGTCGGAGGTTCGATTGTGAGACGATATTGTTATACTGGATAAGAGAGTGTTTCTCCTGCATGAGGAGGCCCTTTGTCCAAATCGGTGGAGCGGGAATGGATGTCATGCCTTGCTGAATTTGGATATCATG

>L0827

ATTCAGCCACATCTCAAATACCGGAAATCATCCGAGTATGCAATTGTCAGAGAGTTGTTAACAGGATTTAGAGAGAGAAATGAGAGAAAATAGCAGGGCGCAACAGAGGGTGCAGAGATACTTGCAATATGGATCGGGGATTTATTGTCATTTGAAAGAGACGATCTGGCCTTCCATCAAGTTACCTGCAAGATTCTCAGTCGTCATCTTCATCTTCTTCCTCGTCGTCTGCGCGTTTTTCTCCACGCGCCTCCTTAACACCACTGTAAGTAAACGCTATTTTGTTATCAAAGTCGCTTCCACTCACAGAAAGAAAAGTTACTTCCCGACAAAAATAAAAGCGTAAATTTCAAGTTTATTTTTGAAGTGGGTTAATAATTATCGTCAGTTTGTTTCTTTTCAATTTTCTATTAATTGTAAATTAAATAAATAAAATATTTATTATTCAAAAGAAT

>L0828

ATTCCTACCTTCTCCAAACTCAGCTGCACAAGATCCTTCTGTTATCACTGATCATGGCTTCCACTTCTTCCGCTCCTCCCAATCAGGGGAAGAAATGGGATGTTTTTATTAGTTTTAGAGGCGACGATACGCGTTATGGTATTCTCTCCCATCTCTCTAAAGCCTTGAAGGACAAACAAATCAAGACTTTTACGGATGAAGAGCTCCATAAAGGAGAAGAGATCTCACCAGAGCTCTTGAAAATAATCCGGGAATCAAGTGTCTCAATAGTCATTTTCTCTGAAAATTATGCAGATTCTCCATGGTGTTTGGATGAGCTTGTTGAGATACTTAAATGCAAGGAAGAATCAGCACAAATAGTTCTACCGGTTTTTTACAAAGTAGATCCAACTGACGTTCAAAAGCTTTCAGGGAATTTTGGGAAGGCATTTGCTATTGCTATGCATGGGGAAAAAGGCAGTTCACATGTTAGAAGAAAGAATATAAGTAATGAAGGAAATGATTGTGTATTTATCTGTATATTTATCTGTATCATTTACAGAGATATTACATCTATTTATACATGAGAATATGAACTAATTTAGAAAT

>L0829

CTGGAGCCAATCAGACGATGAATCATCATCTTGATCGCTCCATGGCATTGGCCTCTTCATGGCACAGTATAAATGTATAGCCCTGGAAACTGCTGCACCGGATCGACAGAGGAGGGCTTTAGGGTTTCTGCTTTGCGAGGCAATGCCTCCAGGCGCGAGAGAGTGCACAGCAGGTTAGCTATTTT

>L0830

AATTCTTTTTTTTTTTTTTTTTTTTTTTTAAAAAAAAATTATATCTATATAAAATGGCAAATGAACTTGCCAGCAACAGTTACAAAATAATCAGGCGTACTTTCCAAAATAGAAGAAATCAAACAGGATAGAAGAATAGCAAAGCAAAATACTGTAAACCCATGCTTTCCTTTGGAAGATTTACACAAAAGGAATTTACAAGATGGGAAAATCCTGCTTCTTCTTTTCCCATGTTTATATCACTAATTTGGTCATAATTTTTCCTCTCAACAAAAGCATACAAGTCACGTGCATCAGAATTTGGCAAGTGCTGTATCAATCAATCAATCACCGATCATCCTCTAGACTGGCGCGATGAAAACACAAGACTGTGCAAACAAATATGAAGAATGATGTGAACTCGATCAACATAGCACCTGTTTCAATCATATGAGCATGCCCAAGGATAATATGCTCCCAACAGCTGACGCCCCTGCAAGGAATTTTGCAGCATCTATCCAACAATATTTGCAGTAGAAAGCTAGCTGAAAACATAAAAGCAAGCCCAGCAAGCCTTTCATTGTCATTGCCATTTTTCT

>L0831

TGGAGCCATTCAATTTGAAGTTGTACATATGGCCAAAGACGGACCCAACTGGGAAGGATTGCTAAAGTGGAGTCTCGCTCACTCCGATGGGACTGGTTCCAATCGTAATCTCAGTGAAGAGGATCGAAGATGGTTTATGGAAGCTATGCAAGCGCAGACTGTTGATGTCATCAAACGCATGAAGGAGATAACTCTCGTCATGCAAACCCCAGAGCAAGTCTTGGAGGCTCAGGGAATTGCTCCTGCTGATATTGAAGATATGTTGGATGAACTGCAAGAGCATGTTGAATCTATTGACATGGCAAATGATCTACACTCAATTGGAGGTTTGGTCCCGCTCCTTGCTTATCTAAAGAACTCTCATGCCAACATTCGGGCAAAGGCTGCTGAGGTTGTAACCACCATTGTACAGAACAATCCTCGGAGTCAACAATTGGTTATGGAAGCAAATGGCTTAGAACTTCTTCTTTCTAATTTCACATCAGACCCAGATTTGACTGTTCGAACCAAAGCACTTGGTGCAATATCCTCTTTAATTCGGCACAACAAGCCAGGTATTACTGC

>L0832

AATTCGTTTTTTTTTTTTTTTTTAAAGGTAACACGACAAACATTATCATAATATAAGCATCCATCATCTTTATACCACATTTCATCCTTGTGATACATACACTTCCATATTTTCCCCAAAAACAAACGGAATTAGTGACAAAAGGATGCACCTCCCTAGGTTCTTTTACCCCTTCAAACTAAGAGGAGTATCACCAAAAATCAACTAGTTACTGCTCACCACCTCCATTCCCCTGTTAACACTTAACCATACTTCCCCAAAACAAGAAAAAAAACAAAAATGAAGATGGCACCTGAGACATGTCAAACCATCAATAAGGTATGATCTATGAACGATGGGAGTTTCCATCTGCCCCTCGATACATTCCTTCATCATCTGGTGGTATCATGCCATCTTCATCAGCAGGAATCATGCCTTCCTCTTCTTCCTCCCCAAAACGAGATTGAGGCACATTATTATATCCTCTCCCCTCGTGTCTACTTCCAGGAGATCTTCCTTGATCCTGCTGAGCACCAAATGACGACGACCGGCCTTTTTCCGTCATTGCCCGACCTCTTTCCATCATAGCCTGGTGAAAACTGCGGCCTGGAGACCACTCACGAGTGGACACCTTGTGAGAATTGTTGCCGCCACCATATGGGGATCTTGACCTCTCCCGCTGGTTTTGTGCTGG

>L0833

AATTCGAGAGAGTGAGGTAGAGGGAGAAATTTAGAGAGAGAAATAAGCTTTAGAGAGAGAAAGTTTTTTCTAGAGTGAGAGCGGAGGAGCCAAAAAGGAGATCTGATCGAATCGTGAGAGAGAAAGGTAAACGAGCACGAAACCCTAACCCTACTGTTTCTTCCAATCCCGAATCCTCTCCTTCTCTATGTGTTCCTGTATATAGAGACATGTATGTGTAGATAGTTCTTTGTGTGTGTTGGTACAGATTTAGATTAGAGTAGATCGATAAAAGTTGGAGCTTGCTGTTAGGTTTTTGTTTGTGTTTTGTGTTTTGCCCTAATTGAATTCTGATTCTAGGGCAAAATTAGGGTTTTTGTGGCTGGTTATAAAGGGACTCGAAAAACCCTAGTTTTGGACCAGTCTTGTGATTCGGTTTGTGTGGATAAAAAAGAAATTAGGGTTTTGGAGTTACATGTATGGCTTCGGGGACTATAGTTGGAGAGGGAGGTGGAAATGATGGAGCTAGAGAGAAACAGAGGTTTACAGAGAGTAAAGTTTATACAAGAAAAGCTTTTAAAGGGCCCAAGAAGAACATCAACAACCTCATCACCACCACCACCACCACCACCACCACCAATAACAACAACACCATAAACGCTCCCAACTCCACAATATCCACCACCGCAAC

>L0834

ATCCCAACGGGTAATTGTACAGAGCTCAAAGAAGATGCCAGTAAGTCTCCAGATCTGTCATCCAATGGACATTCTCAAAATGTTAGTGGTTTTTCTCAAAAGCATGGTGCCAATCTGAATGACAGTCAAGAAATTAAGACCACTCTGGTCATTGGTCAGTCTGGGCAAGCTGAACTAGCATACTCAATTCTTCAACAGCTGATCCCTATCCGAGGGGTAAGGCGTGACAATCTCTGTACTGGATATGGCCACGTTATTCCTCCTGTATACCATAGACAAAGTGGTATATCCCCAGCTTGGAGTCCCAAACTGGCCAGCCAAAGAGAACAGTCTCCATTTAGTACCTCAATTCATTCAAATCCTGAAATTCATGACTCTGAACAGAATCATAGACAATCTGATGAGACTACCATCAATTCCGTGGACCAAAATACACATCAGCAGAATAATATGGAACCTGTTGAGGAACTGAGACATGGTTCTCCGGCTGCTGGTCAGAGTACTAATAGTAGCATGTGTAATAGCATTG

>L0835

GATCCCCCGGGCTGCAGGGTTTTTTTCTTTTTTTTTTATCCGGTACAAGTAAAATTGTATCTATATGATAAGGGAAATGACATTAGGACTGGTGTTAATGTCAAAAGATGATATTGGAAATTTAACATGGTGGCTTCATAATATTACAATCATCACAGTTACCATCCAAAGAATGTCTTACAAAATAATAACACACCCTTGAGAAGAGAGGGATTGGAGTGGAAGTATTATTCAGATTAAACTCATAGACCACCCTTGTCCAGCCCTAATGAAGCAAGGATCCTCATGCAATGCACTAAGGAAACCCTTATCCAGCCCTAACAAAGCAGGATGCCTCATGCAATGCACTAAGGAAACCATCTTCCACTGTTTAGATTCCAGCCTCAAACTTACTTGATCAACTTGGATGGCTTGCATGCTACTTGGACTTGTCCCTTGAACTCGAAAAAGTGAGGTCTTTCCTTCATCATAAGTTCCATCCAAAATACCATTTTCTGCAATGAACCT

>L0836

AATTCCAACTGCCATAAACTGAAAAGAAGACATAAGAAGCATCTCATTCAATCTTAGGGTTAGGGTTATCCCTAATCCGGATACCGTAAACCCGTTCTTGGTCTTAGAGCCGAAGAGAAACAACCAAGATGGGCTCGAATTCGCTATCTTCAGATTCAATCACCGATCTCGATGAGCAGATCTCGCAGCTCATAAAGTGCAAACCAATCTCCGAGCCCCAGGTCAGAGCATTATGTGACAAGGCTAAGGAGATACTGATGCAAGAAAGCAGTGTTCAGCCTGTGAAAAGCCCTGTGACAATCTGTGGCGATATTCATGGGCAGTTTCATGATCTTGCAGAACTTTTTCGAATTGGAGGGAAGTGTCCAGATACCAATTACTTATTTATGGGAGATTATGTGGATCGGGGCTATTACTCTGTTGAAACTGTGACGCTCTTAGTGGCGCTTAAAGTACGTTATCCACAGCGAATAACCATTCTTAGAGGAAACCATGAAAGTCGCCAGATTACTCAGGTTTATGGGTTTTATGATGAATGTTTACGAAAGTATGGCAATGCTAACG

>L0837

GGTTTCTGTCCATCCGATTGCGCATTTCTATGTGAGTACTGGGACCGGCCTCGCACTCTACATAGTCCTCATTATTATGCCATTTATTGTTTTATGCCCCATGTACTACTACCACCAGAAGCATCCGGTGAATTATCTTCTTCTTGGGATTTGAACCATCTCTCTTGCTTTCGCTGTTGGATTCACTTGTGCTTTCACCACTGGAAAGGTTATTCTGGAATCAGTTATTCTGACAACTGTAGTGGTCCTGAGTCTTACTCTGTACACATTCTGGGCAGCAAGGAGAGGCCATGATT

>L0838

GATCCCCCGGGCTGCAGGTTTTTTTTCTTTTTTTGCACAAGAATTGTAACATTTGAAATTTGCAATTAATAGAATTAAAAAAAAAAAAAAGCGACCGAGAGTTATATAAGGTTATTCTATATTTATTTACATATTACAACTGATTATTGTGAATATATGTCTGTTATCTACAACGCCAACAGAAGACTGCTATAGTATCCACATTTCCGCTTCACGGTCGAGCCCTCTGAGCCTCTCATTCTTGTCTACAATTCCATCCACAAATATTAACATGGTTAATGAACCTTCCAGCAAAATAAAAGAATAGCTGCACACCTCTAATCAAACTCATCGTCCTCATCTTCAAAATGATTCTCCACTTTTGGGCCCCCACTGTTACTATTTACAAACTTGAGTTGAGCTTCTGTTCTTAAGT

>L0839

AATCCCCACATTGTAGAATAGTACTGAAATCAATTGCATAGGTATAGTCATAACACTAATTAATGTCAGAATTATGTATCACCATCCTATGAGAACATGTAATACGGACCTGCAAGTCATATGCGGGCAACCTTCTGTTCTTTCCACGTAGAATTTGCATTGGGGACATCTGCTCCATTTCTGATCCCTAGCAAGTTCCATCACCATCAGATCTTCTCTCCCTCTCTCGTCCTCATTAAGCTTCTGGAACACCTCACAATCAACGCCAGAATGCCAAGGGACATGACACCGAGCACAAAACAATCTATGGCAGAACGGGCACTCAGCTTCACTAATCTCTTGTCCTTCTTTATCATTCACCAACATGGCCGAACAGTCCTTATATGGGCAGTAAAATCTCTGCGAAACGCTGATTAGCTCCTGGCAAAGCGCATCCTCCCAAAGATCAATCACTCCCTTGTTGAGCTTAATCCTGCAAGTTTCCAGTTCAAGCACAGCCTTACAATTCAATC

>L0840

AATTGTTTTTTTTTTTTTTTTTGAGTTAGATGACATCAAGCCAATCATTCCAGGCATGGGCCTCATGTGCACTCCTCGGTCATTATCAATCACCTGTATTTGCCTATTCTTGATCATGGACTTATTTGTTCTTTCACGGCATAATCTTCCATACTCCTCAATGTTTTCCTCATGGGGCTTCATGTCAAATTTATGTTCCACTTTCCCTTCCATGGCAACTCTTCCTTGACTTGTCTCAGAAAAGACACCCATAGGAACAAAATCTTTAAACATGTTCAGAGAATAACTCTTAGGTACATTGCCAGTCTCACTGCCAGCCATCTCCATAGTGAACTGTAAACCAGAGGAATCCTCGCCACGGAGAGGATCGAGGGAAAGAACGACCTTGGCGAGAGGATGAGAATCTGAGGAAGAGGAATGACTCTGCCAAGACTTCGCTACGACTAACGGGCACTTCATCAACCACACCGATCTATCAGCCTTTGCCGTTTCCAAGACACCGCTACTACTCCCACTACTACCGTGATCTTCATCCATTTCTTTACTTTCTTTTTGCCGTTTGTTTCTCGAGGGGGGGCC

>L0841

AATTCATCATTTCTGCCACAAGGCATTCGCTTACCACTTTGAGGCATGGCTATTTGTTGAGCACCTGCGCATGATATAAATGAAGATGACTGGATTACTAGGTATTTCTTCACTGGAGGAACAATGCCTGCTGCAAATCTGTTGCTTTATTTCCAGGATGATGTTTCTGTTGTCAACCATTGGCTTGTGAATGGGAAACATTATGCACAGACAAGTGAGGCGTGGCTCAAAAGAATGGACCAAAACATGGCTTTGATTAAGCCAATAATAGAATCAACTTATGGCAAGGATCAAGCTATCAAGTGGACCGTCCACTGGAGAACATTCTTTATTGCAGTTGCAGAACTTTTTGGATACAACAATGGGGAAGAATGGATGGTTGCACTATTCCTGTTTAAGAAAAAATGAGCATGAAAAAACTCCTCCCTGTTAAATTTTCTCTTGTTTGAATTAAATAAGCCAAATACTTCAATCAGATACAGTATGTAACTAATTAATTAGTCTTTAAAGACTAATTTAGTCATGATAATTCCACTGCTACCTTCTTTTAAACCCTCT

>L0842

AATTCGTTTTTTTTTTTTTTTTTTTTTAATTTTTAATACTCCTCACTAATATATTTAAGAATGTAATTTTTTTTTTCAACCACTGCTCCAACAGAAATTCCAATGGGCAACACTTGTGAAATTGATCCACCGGATAGCACAGGGAATCTATGTAGGAAACATATATCAACAAAAATGACAGACTACCAAACCAGTTGCATTAGTGTTACAGACAATTAACCAAAACAATTTTGCATCTGCCAAGAACGCAAACATTTCTTTCATCATATGCTTTCTGTAGTAGCTAAGAGCTTTTCTGTTTCAAAACTTCTGCTTCAGCCCTTGCCATTGGGTCATCCTTGATTTCCATTTCATCTTCCTTTTCTCCTTCTCGCTCCTGTGCCATTTTTTCTATGCAACTCATCGGCAGCTTGCATTGCAGCTCTGTTCTCTGCCTGAATTCGATCCATTTCCTCTTGTTGTTGTTGCCGTTCAAACCATGTGTCAGCATCCGCCCAAATAGGCTGGGCGGCCCAACCCTGTTCACCACCCTTAATGCGTTCGGGAAGGGCATAATTAACGGTGAGGACTTTACCATAGAGCTCAGCACCGTCCATATTATCCATGGCGGC

>L0843

AAAAAACATCTCAGGTCTTTAGGGTCTTTTTTTTTTTTTTTTTGAATAAGAGCATCTCAGAGGTAGATTTGAGAAAGGAATGGAATTTTGCCCATCGTGTGGATTATTGTTGCGATACGAGTTGCCAAACATGGGGCAGCCTTCCAGATTCTATTGCCCTACTTGTCCGTTAAGATCAAGAGAAAGCAACAGCTAGTCAAGAAAGAAATTGAGCCAGTTTTCACCCTCGACGACATGACGAAGGGGGGTTCTGAAACTGAAGCAACATGCCCACATTGCAACTTTGGAAGGGCCCGTTACCAACAGCTACAGATCCGGTCAGCTGATGAGCCAGCAACAACATTTTACTTTTGCTTGAATGAGAAGTGCTCAAGGATGTGGCGTGAGGACTAAATATTTTTTGTGGATGTTTTATTTTAAAGTTTTAAAGTTTTGCAAGGAGATTCTTGCATCTTCCATGTTCAGATGTCTCCTACTATTTGATGTGGTGGTCAATATTCAAGCGGTTATTTTGGTCGTTAACTAGGCTGCTCAATGAGTGAACACCAGCCAACAAATGGAAATGATAATGAAACTTTACATGGACTTTTT

>L0844

AATTCAGAGAGAGAGAGAGAGAGAGAGTAATTGTATGTATATTGTTGCCTTGATTGCAAATTAATATTTGTGGTTGTAGCATGTAATAAGCTATAAACACTATGTTGTAATTAGTTCTTCTTTGATAGCTAAAAGAAACATGAAATTTGTTCACTTTTCGAATCACGTGAAAATTCTCTGCTGAAAGAATGCGGGTTCTCTAAAAAAAAAAAAAAAAAAAACTGAGGATGATAAATTAGAAAGAAAAATAACGCGGAAGCTGAACCAGTTCATACAGGGGACGATGGAGAGAGAGGAGAGAGCTTCTTGGATGAAAAATCAGATGGCCTTTGTTCTCTTTCTGCTACGCAGGAAAGGGAGGTTTCCTCCCATCTTGAAGATTTTGGACACTTGCTGCTAGAGGAAAAACTGGAACAGTGTTCCTTAGGACTGCCTGTGTTGGGCTTGCCTTGCCAATTGCCTGAAACCACTTAACACAATTGAATNTGAGAGTNCCCTACGCTTGCGGCGCTTGAATAAGGAAATGCATATTTAGCAAATATATTTTTCTCCTTTTCCAGGTTTTTCCCCTGTGGATCAGGTT

>L0845

AATTCTTTTTTTTTTTTTTTTTTTTTTTTTTTTTTTTTTTTTTTTTAAACAAAACTAAGTTGGAATAAATTCTCGCAACTGTTTTATAAATAATGCAGCATTAAGCCGCCAATGTAGCATCAAAAAATTTGTCCCGCTAATGGCATAAAATCTTAAATAAAAATGGATGACAGAAAAGATGATAATGTCTAGCAAACTAATTAAAAATAGAAACTAAAGCATTAAAATCATGCCGATCAATAGATCGGCCAAATGATTTTTTTCATCACCCTGCTACAACATCAAATAGATTCCTTAGTCAACTTCCTCAATCTTGGGGCCTGCACCGCTGCCACCCGCTGGAGGAGTATCATCATCCATGCCACCACCCATGTCAGCACCAGCACCTTGGTACATCTTGGCAATTATCGGATTGCAAATGCT

>L0846

AGTTTTTTTCTTTTTTTTTAACACAGAGAGAACAGACCATCCTGAAATTCTAACAGCACTGACTTTTGTTTCAATTTCACAAACTCATTCAAGTAAAAAGACGAGTAATCTACATGTCTCCCTCTCTCAGCCAAAAATTGAAAAAGAAACTTTTTTCAAAGAAATGTCAAAAAAATAAAAAGACTACTTGCTAGCAATGCTTTCCTTCTCAAATATGGCATTAAGTAGAGCAGCAACCTCTCTTTGTATTCCCCGAGGTATCAGCAACGTTAATAGTGGTCCCTTGACCAGGAAGTGTAGAACTGCCAGCTGCTTCCTGTGCTGCCAATGCCTTTTTGCTAATTATGTGGTAGATCTCCGTCAATATGGTTTGGAATGCCTTCTCAATATTGGTTGCTTCCAGTGCTGATGTCTCAAGAAATGAGAGACCTTCCCTCTCTGCCAAGCTCTGACCATCTTCCTCTGCAACAGCTCTTAGATGATTCAAGTCAGACTTATTTCCGGCCATCATAATGACAATGTTAGAGTCTGCATGGTCCCTCAATTCACGCAGCCACCTTTGTACATTGTCAAAAGTTTGCCTCTTGGTTATGTCATAAACGAGAAAGGCACCAACAGCTCCTCTATAATATGCACT

>L0847

ATTCCCACCATTGATTTTCCACCAATCTTAATCTCTCTATTCCTGCCTTTTCCTTCTCTCGTCCACCCAGCGTGCCCACGGGTATTACAAAAGACGACAAAAATTCTTTCTTTCACCCTCCTACGATTCCAGATCCAATCTCTCTCTTTCTCTTAAAAAAGGAAGAACCCCAGTGCGGTTGTCTGTGGCGGTGGTGGTGGTTGTTGTTGTGGATGGCAAGGACCAACAAGTATTCCTCCGTTAACTTTAACCATATCTATGACAGAAACTTAACCAACAACGCTACTCCTAATACCGCAAACAACACATCAAAACAGCCTTCCTCTTCTTCTTCGTCGTCGTCGTCTTCTTCTGCTCTTTATTCTGCTATATCTTCTCCCAATACTTACAAAAACCATCTTTCTTCTTCCCGCTCCCATGGCCGTATGCTGGTTCTGACCCGTCCTACTCCCAAACCCGTTTCAGCTATTGCCACCCAGTCCTCTCTTTCACCTTCTTCGCAAATACCATCGACCCAACAGCGCGCTCACTGGCCGTCG

>L0848

ATTCATTAAATCTTTCGAATTTAAATCACTTCCAACTACTCATTTCATAAAAACAAAAATAAAATAAAAACGACAAAGAAGAAGAAAGGAAGATTGCATGTCTTATAGGTATATACATTTCATTCACCTATAACACGGCTGAAACCCTTCATTTGTTTGCCATTGGCTATGGCAGCAATGCATCATAATGCACACCAAATTAGCCGCCGCGTGTAGTTTCTGAATCTCCTATGTCAATGTCTTGGGGGTTAGGATTTGTCACAGTTTGATGAGCAGCTGTAGGAGGCTGATGTGGCTGTGTCTCACCCTGAGAACCATGCTGTTGATTTGCTTGAGCACGCCTTGTTCTCCATCTGCAAATCTCACCTATCACAGAGCTTCCACACATTGTAATGCCGAACCCAGTGAACGTGGCTAGGAGAACTGATAGAACACCTTGCATATGAAGCGATGAGTAGTAAAGATGAGCAGATAGGACCACCAATGCAAATTGAGTTGTGGCGTANACCCAGACATACCTTCTCCTTACCATTGTTGTAGATGTCATGGATGCAAGAAGACCCATTATACAGGAAAAAGGAAGAGAAATGGCAATTGCACCAG

>L2437

CCGGGGCTGCAGGTTTTTTTTTTTTTTTTTGATGTCAAAACAGCTTTATTATACATAATTTAGCCGAATATTACAGCATAAATTATCTGCTTAACTTAAATGATTATCAGTTGAATAAGCAAGCTTACATATGAAAGAGTATAATGAAAATTTGCAGAGGCAGAAATCTACCCAAACCCAAGACCTACCCCTCTGGGCCAGTTATCCAATCCAACAATCTCTGATGGTAGGCAGCTCATGTACCATAATTTTGCTGCACTGTTGTGAACCTGTGAATGGCCTTTTTTAGTGAACCGAGGTTCATGTACCTCAAGGGTCCCATATGCACCATCTCAGTATATCGAAGTCTCCACGGTGAGAAACCTAGATGGCATCTAACAGGTCCATAAATTAATAGCAAGTCAGGTTCCGGCCCTTTGTAACCTATAGCTTTTAGTGCCTCATCCATTTGATCTTCTGTAAAGCATGGTTCTTCCTTTCCTACACCAGTTTTAGCATACTTCAAATACTTCATAAAGAGTACGTTAGCTGCCCTGGTTATTGCTTCCTTTCCATCGGAGCTGGAAGCAAATTCCACAGTCATCTGTTTCTGGTCCAGTAAAACATCTTTTTCAACTGCTTCCTCAAATGGCATAGCATTGTTCAAACTCTCCATGATGGTTTTC

>L0849

AATTCCGAGTTTTTTTTTTTTTTTTTTATCTAAATATCTTCTCCATTACAAATAACAGGTAGTGGCCTCACATTGGGGTAATAAATACAAGACATATGTTAAAAACCAGGCAATCAAAAGAGCTGAATGAATATTTGTCATGACCTTTAATTTCACATGAGAACATGACTATACCCAACCCGTCAAGCCTAAACTGTCGGCAACATTGTACATAACAAGTTTATCCCAAACAGGGCCAAAGAGTTCCTCCCCTCCAATGGCAAATGTCAAGGCCCATGAGCAAAGAAGAAATGTGAATACTGAGAATCCTGCCGCTGATCTCACAACATAGGGGCGAATGCGAGGGAAGGCGCGGGAGTGGCGGAGAACTGGCCAGAAGTAGAAGCAATTGACTGCCCATAGCCAAGGCAAGAGCGCAAATCCGAATTTGTAGAATCTGCGGGCATAGCTCAACGATTCATCCTCCGAGAGACCCAGTGGGCCATCTACAGTGGGCCACACCGGTGCTGATGATAAAATGGAATTGCTGGAGGAATTAGGGTTGCCAGTTGTGGTGGCAGGACCGGCGGCGCTGGTGGTGG

>L0850

AATTCGGAGTACTACTGCAAAACCGAAACCCTAACCCTAACCAACTGCTTTTCCTTCCTCTGATCTTTCTATCGGTAGCTCTACACAACTCCCATAGTCGCACACCAAAACCCTACACCTTCTGCTTCTGTTTGGTATTGCCGTTTTCAGTCGCCGACGCCATATATGTTCAATTTGAAGGCTGGATAATTTAATAATTTCAGCATGCATATAAAGCAGGTTATAATTGAGGGTTTTAAGAGTTACAGAGAACAAATTGCTACTGAGCCTTTCAGTCCAAAAATTAATTGTGTCGTTGGCGCTAATGGGTCTGGCAAAACAAACTTTTTCCATGCCATTCGTTTTGTACTGAGTGACCTCTTCCAAAACCTGCGTAGTGAAGATAGGCATGCACTACTCCATGAAGGTGCAGGGCACCAAGTTTTGTCTGCTTTTGTCGAGATTGTGTTCGATAATTATGACAATCGCATTCCGGTTGATAAGGAAGAAGTACGACTGCGTAGAACAATTGGTTTGAAAAAGGATGAATATTTTCTGGATGGGAAGCACATCACGAAAACAGAAGTGATGAATCTGCTAGAAAGCGCTGGGTTCTCCCGCTCCAATCCATATTATGTAGTGCAGCAAGGAAAGATAGCATCATTGAC

>L0851

CCAATTCGTTTTTTTTTTTTTTTTTGTTTTTTAGCCTTCTGATTCATCTCAGACCTTTTAGAAAAGAGAGAGGGTATGATAACAATTTTACAGTTGCAGTTCACTTAAAAACTTCTAGTGCAAAAAAGACTAAATCAGAGATCATCACAAGTTTCCTATTCTGCAATGAGACAGTTCTCCAAATTAAAATGAGGAGGACAAGCCAAAAGAGCATACAGATTCAAAGTTAGGCTTTGAATATGAACAAAAGATATGTCTATGCCCCATCTGGCTGCATGAAAGGGTTGGCAATTTCATCTGTCCCTTCAGTTGGTGAGACCAGCATTACAGCAGTTCCCCTGCAAACTATTAAGCCAAGGCGCCTGGTCTGGCCAGTGGTCTTCAGTGGATCATCAGGATCTCTTAGATATTCTATAGCTTCATCCAGGACAAGATTTAACAATTGATCATACCCTTTCAGAGTCCCTGTCACTTGTCTACCACCAGTGAGCTTGACTTGGACACCCTTATCCACAAATTTTGCCAAATCCAACACAGTTTCTTTCCTTCCTGACATCTTTACACAGAAATCTGAGAACCCTGAAATTTCGTTTTAGCAGAAAATTGCAGTGTGCCCTAGCTTGCCTCTTCCCCCTTGCCAGAGGT

>L0852

ATTCAGTCCTTCGAAATAGGCTCAAGGAAGCTTCCTCCCGTTTGGTTGGCGAGAAAAACTTCTGTGAAAAGGAGGGAGAATGAAAATTCTGAATTTCAGGCTTGTAACTACTTCCTTTTCAAAATTTCGAGCTTAACATGATAATGCAAGCGAAACGAGCATCAGGTGATTACTGAAGAGGTGCTCTAAAACACTGATGATTAGCAGTTATTGGCTTTGAACTTTTTGAGCTCTGGGATGCAGACATTCGTTCCTTCCGCACACGGAGATCATCCAACAGTTTCTGTGCAAAATCTGATCTTTTCACCATGTTTCTTCTCAGGTGGATCCAAGTTGCAAAGCACTTCAACCAAAAAAATGGAATCATGCAAAGTGAAAATTCTCCGTCTCTGCAAAATTGCTTTTCGATGTGCAAATTCTGTGCGTGTCGGTGGAGCCATGGATGCTACGCTATGGAGTCTGGAGGATAAGCATCTGGAGTGGGTGGAGAAGGGTTTTCGAGTGATCATAATCAAAAGAAAAAGAAGCGTCACAACTCAGAAGACAAAACCGTAAGTGGTAATTCCATCC

>L0853

AATTCGTCTGTTTTTTTTTTTTTCACCATGTCTACCGATCTTCAATTACGTCAAGATTTGCCAAGAATTCAAGTTCCACCCATCAAGATTCAAACCCTAGGATCTTGCAGCGCCACCAGGGAGGATCATGGAGACGTAATTCAACAAGAAAACATCGACGAATGTCGAACGCCGACATCTGAAGAACACAGGATTCCTGCCGTGTTAAGGTGTCCGCCGGCGCCACGGAAACCCAGGAGGAGGACGGTTTCATATAAAAGAAAGTTTTCAGAGTTTGAATTCTTTGAGATCCTGAACCGGCAAGAAGTGGAATCTTTCTTTCTGTCAAGTTTTGAGGCTGTTGCTGCAGCAAAGAAGCGATGCCCTTATAAATGAAACGCGGCGTATTTGTGCCACAAACTCACGTTTCTCTTCTTCTTCTTTTTATATTGATTTAAGGTTCATGGTTGAAAGAAATGTATTTATTACACGCTTAAATCCCATTTATAGGTATGTAATTCTACATCATATATTCATATGATCGATTTGTTTTCTTTTGATTAT

>L0854

AATTCCGAGTTTTCTTTGCCCTTCACCTAAAAAGTGTTCTAGGTCAGTTCAGAGGTGTAAATCTGATCCTACAACCGTACCACAAAAGCCACAATTAGATGTGGAAGGGGGGCGCAGGTTTTATTTATACAATGACTTGCGAGTTGTGTTCCCTCAACGACATTCAGATGCTGATGAAGGCAAGTTGAATGTGGAATATCATTTCCCAGACAATCCAAGATATTTCGATATCAGCAACTGAAATCTCAATAGATTGCTGACATTGTACATAATCACATCATGTTTTTAGTTGTCTAATCTAAAAGTTGCACTAACCCCTCAAGTTGTAAATGCTGCCATTCAATAATATTTGACGGGTTGAAGATTANAAAAAAAAAAAAAAAAAAAAAAAAAAAAAAAAAAAAAAAAAAAAAAAAAAAAAAAAAAAAAAAAAAAAAAAAAAAAAAAAAAAAAAAAAAAA

>L0855

TGGAGCCAATTCCGCATCGGTATCGTCGGCGAGGTTTGACAGCGAGAAATGGCTTCAGGTTGGGGGATCACCGGAAACAAGGGCAGATGCTACGATTTCTGGGTTGACTTCAGTGAATGCATGTCTCGTTGCAGAGAGCCCAAGGACTGCGCTCTCCTGCGCGAGGACTACCTTGAGTGCCTCCACCACTCTAAAGAGTTTCAAAGAAGAAACCGTATTTACAAGGAGGAGCAGCGTAAAATACGAGCAGCTGCTCGGATGACCAAGGACGGTGGAGATGGCATTGATAGCCATCATGCATGACCAATAATTGATCATCTTTTGAAATTCTAGTACTAAAGAACATGTCAAGATGTATTTTTCATTCTTCTGATGCTGATATTCAGACTGTGTTCAAGTGATTCTGTTTTTGTTTGTTTGAGAATCGAAATGCAAATGTGGGTCTTTTTGATTTGTGCAATAACATGAAGTTATGTGTTTCATA

>L0856

AATTCCTCTTATGATCTTGAAGATATTGTTCTTCAAGTGTGCTTCATCTCAAGTAGTTTATACAGTCGGTGAAGATGAAGGATGGAATTCTGAAGCAAACTTCGATTCCTGGTCACAGAAATATAACTTCAGCGTCGGTGATGTTCTTGTTTTCAAGTATGTGAAGGGGCAACATAATGTGTACGAAGTAACAGAGGACACATTTCGATCATGCGATGCAAGCAATGGAGTAATAGCAAAGTATGAGAGTGGAAACGATGAGATAAAGCTGAACAAAGAAAAGAAGTATTGGTTCATATGCAATGTGGTTGGGCATTGCCTTGGAGGAATGAGGCTTAATATTGATGTGGGGAAAGGAAAACAAGCCAATTCCTCTTCAAATGGTGAAACAATCCCACAATTGGAGCCAACGCCACCACCCACTTCTTCATCTTCATCTTCATCTTCAAGTTTTTCCGTGGGGATTTGGATTTGCTTTCTTGCTCTCAGAATTTTGTTCAAATTCTTTCCCTGAAGGAAAATTTAATTTTCTTGTATTGGGTTTTTAGAATTAATTTCGTTTTTAATGTAATTACTTC

>L0857

AATTCGAATTCTCTCCTTCAAGCCGGTCGTCGCTGAGTTGATATCCCTCCCTATGCCCACGGTGGCAGCCATCCAGGGCCATGCCGCGGCCGCTGGATTCTTATTGGCTTTAAGCCACGACTACATCCTCATGAGGAGTGATAAAGGCGTGTTGTATATGAGTGAGGTTGACTTAGGGCTTCGGTTGCCGGATTATTTCGCGGCTGTTTTTCGAGCTAAACTCCACGCGATTTCTGCTCGGCGGGATGTCCTCCTGGGAGGTGCCAAGATGAAAGGGGAGCAAGCGGTGAGGATGGGAATCGTAGATGCGGCGTACGACAGTGAAGGAAGCTTGGCTGAGGCTGCGACGCGCCTGGGGGAGGAGTTGGCATCAAGGAAATGGAACGGCGACGTCTACAAAGAAATAAGGACGAGCCTATACCCAGACCTATGTGGCGTGCTGGGATTGGGTGAAGGGAAATTTCTAGCTAAACTTTAACCAAAAGAAATTGTCGTTGGAGTGCTTGGTACGGTTCTCTCTCTCTCTCTCTCTCTCTCTCTCTCTCTG

>L0858

AATTCCAACGTTTTATTTACATTTTTGAGCCTCTACGGTTTTCCCTGTTGCCCCTCACAGCTTTTTGCAAGGGAAAAAGGGGGGAAAAAAGGGGGCAGCGTGTGCCGCAAATTCTTAATGTGAAAGAAGAAGACCAATTTCTTCAATCCGCGAAAAAGCTGCTTCTCCCATCTTCATATTATCTGCATCTTCTTCTCTCATTTCCAAATTACGACAACCTTCTGCTCAAAGTGGAAAATTTGATTTGGGCTTGTTAATTTTGCAATTAATTTTTGGTTTTCTTGGGGAGAAAATGGCTATCGGCGATGTCTTTGACGGCAATGTTGACACTTCCAGTTCTACAGTATCAGAGTTGATATTGTCTTTGAAATCGGCGTTTCAAAGTGCCCATTTTTCGGAAGTTGAATCAATTTTGAGTTACAGAGAGCTGAAACTGAAACGCGAAATTGAAGAGAAGGCGGAAGAAAATAAGCAG

>L0859

AATTCCGTTTCTCTCTCTAGAACGTTCTTTCTCTGTCTAAAGCTCTTCTAATATGGCATCTCAAGCCAGCCTGCTCCTTCAAAAACAGCTTAAAGATCTTTGTAAAAATCCGGTGGATGGGTTCTCCGCGGGCTTGGTTGATGAGAATAATATCTTTGAATGGAGCGTTACCATTATTGGACCGCCTGATACGCTTTATGAAGGGGGATTTTTTAATGCCATAATGAGCTTTCCATCCAATTATCCAAACAGCCCTCCAACTGTGAAGTTTACCTCAGAGATATGGCATCCTAATGTTTATCCTGATGGGCGTGTTTGCATATCAATTCTTCATCCTCCAGGTGATGATCCAAATGGCTATGAGCTTGCAAGCGAACGCTGGATGCCTGTCCATACAGTTGAAAGTATAGTGTTGAGTATCATATCAATGCTTTCTAGTCCTAATGATGAATCT

>L0089

TGCAGGAATTCGAATTCCTCTTTCCTTGTTCTGTGATCCTGAAACATCAAACATGGGGTCACCACTGAAGAACTTTGCTGTGGCACTAAAACCCAGAAATACCTAGTCACCATCCACCATCGCCTTGTCCAGGCCACGGTTACCTCCTCACGGACTGTGGTAGACTGGTGGATCAAAGGCCACCTTCAAGCTCCATGCCACCATCACCAGAAAATTGTTGGCCTTGACATAGATTGGCGTCCAACTTTCATACGCGGCAGCAGCCAGAATCCGGTGGGAATACTCCAACTCTGCATCAGCAAATGCTGCCTCATCTTCCAAATTTACCAAGCTACCTGCATTCCTTGGTCCCTCTGCAACGCCCTCAGTAATCCTAACATCATCTACACTAGAGTTCGTATATCCGGTGACACAAAGAAGCTTTCCAAGGACCATGACTTGGAGACATCTCGTGAAGCAGATATTGCAAGTCTAGTTGCACAAGCAATTGATCACAAGGAATTTAGGAGATCCGGATTGAAGACTCTGGTGGAGACTATTATTGGGGAAGAATTGGAAAAGC

>L0860

AATTCGTTTTTTTTTTTTTTTTTAAACATGATTCATATTGAAATTCATCTCCTTGGCAATTATTCTTCCCACCAGTGCCAACAAGCACATTTATTCCACTCCCAGCTGCAACTTTATGATTCCTATTTGGCAGATAACTAGAAATACCACATACATTTTCATCCCTCAGTTGTTCTCCTTCTTCAGTGTCTATGTATTCATTAGCTGGAAGTTGAAGATCAAACATCTTTCTCCTCACCTTCGTGGGCCTGGATTCCAGTATCTCTGCATCTTTCAAAGTACCTCCATTTTGAGATAGCAAGGGACTAGCTTGTGCACTGCTTCCTTTCATAGAACTCAGTGGAGGATGCACGTCTTCAATGCCTGAGGTAGATGGTCCGGCACAAACAGAGTTTCCCAATGGGAAGCCAGGGATATGCCATTTCCGAGTTTCTTCAGATGTAATTTGAGATGTTAAGGGGATTGATGACAACGAAGTTTCAATAGGCATCTGATTTTTATATAATTCTTTCCTTTTAACTTCATCCATTAAGTCCCTCTGTATTCTGTATAGGCGGTGAAGTTCACACAGCTGCATCTTAAATATGGCCTCATGCTCCAGCATTTTCTGCTTTACTACATCCTTGTCATATCCTGGATATACATCAGTGATGGGCCTTGGCAAGAATCCATTATAGTACTGCCCATTTGTGAATGCTCGAGGGGGGGCCC

>L0861

ATCGTTTTTTTTTTTTTTTTTTTTTTTTTTTCACCCCAAATTCACTCTAGCTAGTTCACTTGAACAGTAATTATAGTATATCAATGATGGAAGTTTGCATGAACAATGACAAGGTAATAAATTTCCACTCTCTCGCATATCAATGACAGCTGAAATCTGAGGGATTTGAAAAAGAGTTTAATGTTTGCAAAAGGAGAAAATAAAATTAACATGGGAAAATAAAACATGCTGGTGGAGTTTTTATGTGTGTAGTTTCAACAGTCTTCAATGAAAATGATGGTTTACAGTAGACGATTGAGTCGACCTAAGGACAGTAGGTTTTAAGTCCTTTTCTCTTGCATCCATTCTACAAAATTATCCAGGATCAAAGGCCAAGCAAGTTCTGGATCATAATTGCTGAGGTCTGGAAAGCTTATCTCAGTGCAAAAATGGTAAAAGCCCATCCATTGATCCATGTTTATGACTTTGTAATCATTCTGAATCTTAAGGTACTCAATGAAATAAATCACTTGGG

>L0862

AATTCGTTTTTTTTTTTTTTTTTAGGGCTTCAAGAGAGATGTAATTAATAGATTCAAAACTCAGAATTTCTGATATGTCTAGTATATTTTAAGATAAGTTAACAAAATAAGGTGGTTGTTGATTACTTTGGGCCTCAGGTGCATCTGAAACCAAAAAAAATTCCTCCTACCAAGAAAAATATTGTCCTACACCCTGTAGAGAATTATCTGTCTAATAATGAACACTGCTGCATCTGGGAATTCACGTTGGAAAATAAAGATATGGACGGAAACCACTGAGAGAATCACAAGTTAGAACACTAGACCTAACATTTATGGAGGCTATCTCTTTTCAGGACCCTCCAACACCCTTGCCTGCAATAGCTACCTTCTTACAGACTCAATATTTCCTTTTCCATTCATTGACAGAGAATTTGACGAAGGTTGTGAAAATCTGATATCCCTTGCTTTCCTTTTCTCTTCTTCCAGTGTATCTGTTTTAGAAGTTGGTGCAAATCTCTCAAGTCGAGCCTTTTTCTTGGCTTCCTCATC

>L0863

ATTCGCAAAAACAAAAAAAAGAATTAAAAAAATAAAAGACCTCTGTAAACCTTACCGCAGCCAAAAATGGCACTGATGCTCTCCCGCCAGAACGGAGCTCTCGCCCTCTGTAACCCTAAACTTTCCGGCGGTCCTCTTCTCAAACCTGTCTCCACCTTTTCTTCTCCGGTTCACGAACCTCTAAAATCCTTCTCTTTTTCCTTTAACACCCCCAGAACCCCTGTAAAACCAAACAAGTTTCCCTACTCTCCCAGCACTGGCAAATTCCCACTATTTCTTATCCCACCGATTCACAAGACCTTCTCACTGGGCCAACACGGACCCTGACAACTATCTTGACCTTAGCGTTTTCTCTATCACGGCTATTTGTAAATTCAATTCAGAAATAGGCTCTGTCAGTCGGAGGAATTTCGTTTCTGCCTAATCTCAATGAATTGGCGGCAATTCGGGCACAAAGCAACCAGGTTAACTCGGTGGGCCCGTTATTCT

>L0864

AATTCCAAATACCCACAACGGAATTACCAAGCGGAAGACCTTCTCTTATATTTAATCTCGTTGTCTCTCTTTTTGAGCTTCTTCTCCCTCTCTCTGTAGTCGGTAGCTAATAAATGGCGGCGTCTCAAGGCAGTCTTCTCCTCCAAAAGCAGCTCAAAGATCTCTGCAAGAAACCCGTTGATGGATTCTCTGCGGGTTTAGTCGATGAGAACAATGTATTTGAATGGAGTGTTTCAATTATGGGACCCCCTGATACCTTATACGAAGGTGGGTTCTTTAGTGCCATCATGAGCTTTCCACAGAACTATCCCATCAGTCCTCCAACTGTAAGATTCACTACAGAGGTGTGGCACCCAAATGTTTACCCAGATGGAAAGGTTTGCATATCAATTCTTCATCCACCTGGCGATGACCCAAATGGCTATGAGCTTGCGACTGAGCGATGGAGTCCAGTCCACACAGTAGAAAGCATTGTTTTGAGCATCATATCAATGCTTTCAAGTCCTAACGATGAGTCTCCTGCCAATGTTGACGCTGCGAAACAATGGAGAGATAGC

>L0865

CTGGAGCCACACGTTGAAGTCACACCTTATTTCACTTCACGATTGCCTTTCTAGCTCCGTCGTCGTTGTGGTTGTCATTCACCGCTCTGGAGCCGCTGTTTTGATTGAACTAAAGGAAGCAAGGAACCCTAAATAGCTGCCCACTCCTCTGCCTCGTTTCCTTTTTAGAAAATCGTTCTCTTTTAGAGTTTATCGCTCTTTACTGGCCTAAAAGAACTATGCGATTCAAGGAAGTCAATTCGGGAGAGATTCAATAATTTCCCCAATTCGAATTCCAATAAATTGAGATTTTGTAAGTTTTTGGCATGGTCCATGGATCCTGATGCTGCAAAGACCACAAGAGAATCTCTGGATTTGGCATTTCGCATATCAAACATTATGGACACAGGACTTGATCGACAAGTGCTGTCGGTGCTCATTGCTTTGTGTGACTTCGGTTTAAATCCTGAGGCATTGGCTGCTGTCGTAAAAGAACTCACAAGAGAATGCTCTAGCTCTCTACCAACCCCTAGTGCTCTTTCATCTGTCCCATAAATAGTTTTCTAGCATTACCAATGTTATCTTTCTTTGCTTTCCCATGGTTGTATATGC

>L0866

AATTCTTTTTTCTTTGTCGAGCTCCAGCCTCATTTTTTGTCTCCCAGACATATTTTTTCCGATCACTCTCGCATCAAATTCCCAACCTAAAATAAGCGACATGGGTTCTAAATCCAGGAGCGATAACCCACACTCCGGAGATGGTGCTAGCCCTGGGAAGATTTTCATCGGAGGATTGGCTAAGGATACCACATATGCTACCTTTAACAAGCATTTTGGGAAATATGGGGAGATAACTGATTCAGTGATCATGAAAGATCGGTACACGGGTCAGCCTAGGGGTTTTGGTTTTATTACCTATGCCGATCCTTCAGTTGTTGATAAGGTTATTGAAGACACCCACATCATCAATGGGAAACAGGTTGAGATAAAGCGGACCATTCCTAAAGGTTCTGGGCAATCAAAGGATTTTAAAACCAAGAAGATATTTGTTGGTGGAATTCCATCATCAGTTACAGAAGATGAGCTCAAGAATTTCTTTTCGAAATATGGGAAAGTGGTGGAGCACCAGATTATACGAGATCATG

>L0867

ATTTTTTTTTTTTTTTTTTTTCAAAAGAAAGAGTGATTATTTTCTATTTAGTACTTCAAGTATATAGTTACTCCAACGATTACCATGAATCAATTAATTTTGTGAAATGTTTCAGATGCTAACCTCATTGGCTGCAGAAGCAGGACCAAAATACACCATTTTACCAGAAGAAAGGAGGCAAAGATTGTTAAAAAGCTGAAAGACTTCACTGCTAGGCTGATGAATGGAAAAAATGATAGTTCTTCTGATTCCATCATTCCTGCCCAAGCTAGCAATTCTGCTCATGATATAATATGAAGCTGCACTATCAAGGCCACTTGTGGGTTCATCAAGGAAGAGAAGTTTTGGGTGTGTTAAGATCTCTATGCAAATGCTTACTCTCCTCTTTTGGCCACCACTGAGGCCTTTTGCTCCCCAGCCTCCTATTCTTGTGTTCCTGGCATCTTGTAAACCCATTTCTCTTATTGTCCTCTCTGCTCTCTCCTTCTTTTCTGA

>L0868

ATTCGTTTTTTTTTTTTTTTTTGTTTTTTTTCAGAAAATGATAATATCATGTATAATAATTACAACATGATAACACGAAGAAGAAAATCAGCAAAGGTACATAAAGAAGCTTCACATTATTGATGGCCACAAAAATCTGTATACCAGAAAATTTGCGGAGTTATTTCTGATTTCAGGTGAAACAATTGCAGAAAAACAATTGCTGCACTGATACATAAAGGATCCTCATCCAGCTTACTCTGTTTATACCCCTGTAAAGCTTACCTATATTTGTTTCCTATCAAGCAGTATCTAACTAATCAATGCGTTCGAGATTGTACAACACCATTTCTTTGAGATGTGATTGAAATACACCCAGAGGAAGACAATTAAGATTCTGTATTGCAAGATCAGCTTTCTCCTTCGCTAATTCTTGTGCTCTTTCAATTCCCCCACACTGCTTAACCAACTCAACAGCTTCATCCAGAGAACCAGTCTCA

>L0869

ATTAAACACTATGCTTAAAAGCGGGACCAGCCCCAGCCCGCCATTAATGCAATTCTTTTCACCTCCCTCTAGCTCCATATTAATTGTGCTACTTGTCAATCTAAAGCTTAGCTCAAACCAGGAAGAGAGCTTCAAAAGGGTACCTTGTTTTGTGATTAATTTGATGGGGGTTTGCTTGAGCGCCCAAATTAAAGCTGAGAGCCCATTTAACACAGGGCTGAGCTCGAAATATGTTAGCACAGATGGGAATGATCATAGTATGAGTAGCAAAGTCTCATCAGTTTCAGTGCCACCAACTCCTCGGAGCGAAGGTCAGATCTTGCAGTCCACTAATCTGAAGAGCTTCAGCTATTCTGATCTCAAGATGGCCACAAGGAATTTCCGTCCCGATAGTGTCTTAGGAGAAGGTGGTTTTGGTTCTGTNTTTAAGGGATGGATTGATGAGCATTCATTTACTGCTGCCAAG

>L0870

AATTCCTTAGAGATTTGGTCTCTAAAAGCAAACCAGGAGATAAATATGGCAGTTATGCCCTTCAGTATGCTGATGACTTCAAGTACACTGATCCCGTGGATGGAAGCGTGGTAACAAAGCAAGGGGTTCGATTTGTTTTTACTGATGGATCAAGGATAATATTTCGGTTATCTGGAACTGGATCTGCAGGTGCAACTGTTCGAATGTACATCGAACAGTTTGAACCAGTTGCCTCTAAACATGAGATGGATGCCCAAACAGCCTTGAAACCATTAATAGATCTGGCATTGTCTCTGTCAAAATTGAAGGACTTCACAGGCAGGGAGAAGCCTACAGTTATCACATAATGCATATCCTTGAATTGTTAGTATCATGAATCAAGTGGCATCTTGGAATTTCATCTCTTTTCTTTTTGGCTTAATTTGCTACATGTATTAGCTGTTCAATTTTGGTTCTGTAGCCATCTGTTATTGTGATATAATAAGTCCAGGTAAAGTAAATAAAACAGCCAAGTGGAAATAAAATCTGTCATTTGGTTCCAAAAAAAAAAAAAAAAAAAAAA

>L0871
[truncated: 1,209,196 more chars]
