# Supplementary material for: Profiling Ethylene-Responsive Genes Expressed in the Latex of the Mature Virgin Rubber Trees Using cDNA Microarray
Source: PLoS One. 2016 Mar 17;11(3):e0152039. doi: 10.1371/journal.pone.0152039 (PMC4795647; doi:10.1371/journal.pone.0152039)
Supplement: S4 Table — (DOC) [file pone.0152039.s005.doc]

**S4 Table.** The differentially expressed genes in the laticifers of rubber trees stimulated with ethephon for 1 h.

| **EST name** |  | **TSA** | ***q*-value (%)** | **Fold change** | **Seq. Description** |
| --- | --- | --- | --- | --- | --- |
| L2615 |  | JR366931.1 | 0.000 | 2.310 | polyadenylate-binding protein 2 |
| L0236 |  |  | 0.000 | 2.094 | casbene chloroplast |
| L2819 |  | JT918804.1 | 0.000 | 2.031 | Ubiquitin-associated ts-n domain-containing protein octicosapeptide phox bemp1 domain-containing |
| L2592 |  |  | 0.000 | 2.071 | hypothetical protein* |
| L1426 |  | JT931152.1 | 0.000 | 1.784 | E3 ubiquitin-protein ligase RNF181 |
| L1596 |  | JR350036.1 | 0.000 | 1.789 | eukaryotic translation initiation factor 2c |
| L1227 |  | JR345627.1 | 0.000 | 1.791 | hypothetical protein JCGZ_08192 |
| L2819 |  | JT918804.1 | 0.000 | 1.728 | Ubiquitin-associated ts-n domain-containing protein octicosapeptide phox bemp1 domain-containing |
| L0041 |  | JT923948.1 | 0.000 | 1.785 | probable polyamine oxidase 2 |
| L2592 |  |  | 0.000 | 1.604 | hypothetical protein* |
| L2687 |  | JT920567.1 | 0.000 | 1.622 | flocculation protein flo11-like |
| L1092 |  | JT917867.1 | 0.000 | 1.523 | Ubiquitin-associated ts-n domain-containing protein octicosapeptide phox bemp1 domain-containing |
| L0055 |  | JT924152.1 | 0.000 | 1.659 | NAC domain-containing protein 78 |
| L0735 |  | JT923498.1 | 0.000 | 1.518 | polyadenylate-binding protein 2 |
| L1077 |  | JT946738.1 | 0.000 | 1.646 | conserved hypothetical protein |
| L1191 |  | JT960147.1 | 0.000 | 1.572 | conserved hypothetical protein |
| L1592 |  | JT959698.1 | 0.000 | 1.626 | protein binding protein |
| L0249 |  | JT918900.1 | 0.000 | 1.594 | cyclin-dependent kinase f-4-like isoform x2 |
| L2008 |  | JT926846.1 | 0.000 | 1.598 | polyol transporter 5-like |
| L2615 |  | JR366931.1 | 0.000 | 1.510 | polyadenylate-binding protein 2 |
| L2352 |  | JR365074.1 | 0.000 | 1.528 | wound-induced protein 1 |
| L2272 |  | JT917867.1 | 0.000 | 1.505 | zinc ion binding protein |
| L0885 |  | JT925665.1 | 0.000 | 1.547 | ABC transporter B family member 15-like |
| L0004 |  | JT934287.1 | 0.000 | 1.513 | DOF-type zinc finger domain-containing family protein |
| L1763 |  | JT934383.1 | 0.000 | 1.512 | glycerol-3-phosphate acyltransferase 8 |
| L2592 |  |  | 0.000 | 1.530 | hypothetical protein* |
| L2771 |  | JT915676.1 | 0.000 | 1.532 | protein smg7 |
| L1946 |  | JT976119.1 | 0.000 | 1.524 | nudix hydrolase chloroplastic-like |
| L2348 |  | JT971116.1 | 0.000 | 0.652 | avr9 cf-9 rapidly elicited protein |
| L2311 |  | JT958760.1 | 0.000 | 0.605 | conserved hypothetical protein |
| L1739 |  | JT963491.1 | 0.000 | 0.646 | E3 ubiquitin-protein ligase rha1b-like |
| L2279 |  | JR366494.1 | 0.000 | 0.599 | basic leucine zipper 9-like |
| L2522 |  | JR355065.1 | 0.000 | 0.523 | conserved hypothetical protein |
