# Supplementary material for: Profiling Ethylene-Responsive Genes Expressed in the Latex of the Mature Virgin Rubber Trees Using cDNA Microarray
Source: PLoS One. 2016 Mar 17;11(3):e0152039. doi: 10.1371/journal.pone.0152039 (PMC4795647; doi:10.1371/journal.pone.0152039)
Supplement: S5 Table — (DOC) [file pone.0152039.s006.doc]

**S5 Table.** The differentially expressed genes in the laticifers of rubber trees stimulated with ethephon for 4 h.

| EST name | TSA | Fold Change | q-value(%) | Seq. Description |
| --- | --- | --- | --- | --- |
| L1843 | JT964854.1 | 2.500 | 0.000 | hypothetical protein 3 |
| L1911 | JR344767.1 | 2.471 | 0.000 | ETHYLENE-INSENSITIVE3 protein |
| L2487 | JR366057.1 | 2.366 | 0.000 | AP2 domain transcription factor family protein |
| L0025 | JT949537.1 | 2.346 | 0.000 | methylesterase 10-like |
| L0528 | JT963312.1 | 2.216 | 0.000 | profilin 3 family protein |
| L1499 | JT936619.1 | 2.173 | 0.000 | bidirectional sugar transporter SWEET10-like |
| L0459 | JT930276.1 | 2.157 | 0.000 | mitochondrial metalloendopeptidase oma1 |
| L2117 | JT966645.1 | 2.126 | 0.000 | fiber protein fb11 |
| L0055 | JT924152.1 | 2.014 | 0.000 | NAC domain-containing protein 78 |
| L2174 | JT927768.1 | 2.013 | 0.000 | PREDICTED: uncharacterized protein LOC104905041 |
| L1648 | JT960597.1 | 1.962 | 0.441 | acyl carrier protein |
| L2223 | JT942604.1 | 1.926 | 0.000 | hypothetical protein JCGZ_04721 |
| L2347 | JT952580.1 | 1.893 | 0.216 | ADP-ribosylation factor-like protein |
| L0891 | JT966473.1 | 1.891 | 0.000 | dynein light chain cytoplasmic-like |
| L1127 | JR362827.1 | 1.886 | 0.000 | ethylene-responsive element binding protein 2 |
| L1901 | JR364493.1 | 1.877 | 0.000 | NAC domain-containing protein 2 |
| L2914 | JT952575.1 | 1.849 | 0.000 | probable ribose-5-phosphate isomerase 2 |
| L0407 | JT945196.1 | 1.801 | 0.000 | exonuclease chloroplastic/mitochondrial |
| L0294 | JT948170.1 | 1.782 | 0.441 | lupus la ribonucleoprotein* |
| L2723 | JT951227.1 | 1.766 | 0.000 | AP2 domain transcription factor family protein |
| L0567 | JT966885.1 | 1.764 | 0.000 | hypothetical protein JCGZ_04890 |
| L2240 | JT961569.1 | 1.748 | 1.606 | PREDICTED: uncharacterized protein LOC104227135 |
| L0051 | JT957340.1 | 1.726 | 0.000 | diphthamide biosynthesis protein 3-like |
| L2771 | JT915676.1 | 1.717 | 0.000 | protein smg7 |
| L0570 | JT971674.1 | 1.698 | 0.000 | acetyltransferase at1g77540-like |
| L1343 | JR366829.1 | 1.693 | 0.216 | sucrose transporter 1 |
| L1287 | JT969848.1 | 1.688 | 0.000 | protease inhibitor protein 1 (PI1) |
| L1582 | JT930280.1 | 1.684 | 0.441 | proteoglycan 4-like isoform x1 |
| L2677 | JR359565.1 | 1.684 | 0.000 | 23.6 kDa heat shock, mitochondrial -like protein |
| L1186 | JR364404.1 | 1.676 | 0.000 | cysteine proteinase inhibitor |
| L2008 | JT926846.1 | 1.674 | 0.000 | polyol transporter 5-like |
| L1433 | JT944001.1 | 1.673 | 0.000 | fatty acid 2-hydroxylase 1-like |
| L2195 | JT973106.1 | 1.667 | 1.606 | uncharacterized loc101212373 |
| L0046 | JT930900.1 | 1.664 | 0.216 | fatty acyl- reductase 3-like |
| L0383 | JT932391.1 | 1.648 | 0.000 | probable inactive poly[ADP-ribose] polymerase SRO3 |
| L2108 | JT957706.1 | 1.647 | 0.441 | desiccation protectant protein lea14 homolog |
| L1680 | JT948897.1 | 1.638 | 0.000 | histone H1 |
| L0948 | JR357889.1 | 1.637 | 0.000 | PREDICTED: uncharacterized protein LOC104601116 |
| L0793 | JT951926.1 | 1.634 | 0.000 | HSP40 cysteine-rich domain superfamily protein |
| L2303 | JR357022.1 | 1.627 | 0.000 | glutaredoxin-c6-like isoform x3 |
| L0342 | JT923651.1 | 1.615 | 0.000 | rpm1 interacting protein isoform 1 |
| L2852 | JT958968.1 | 1.609 | 0.000 | cyclin-dependent protein kinase inhibitor smr2 |
| L0657 | JT941656.1 | 1.595 | 0.000 | lipid phosphate phosphatase chloroplastic |
| L1072 | JR366225.1 | 1.588 | 0.000 | cell number regulator 8-like |
| L1016 | JT946494.1 | 1.586 | 0.000 | uridylate kinase |
| L0447 | JT950737.1 | 1.573 | 0.441 | protein n-terminal glutamine amidohydrolase |
| L1533 | JT928961.1 | 1.562 | 0.000 | Inositol-tetrakisphosphate 1-kinase |
| L0359 | JT960025.1 | 1.555 | 0.000 | Ribosomal L18p/L6e family protein |
| L0617 | JR365244.1 | 1.554 | 0.000 | hypothetical protein JCGZ_04615 |
| L1091 | JT954366.1 | 1.524 | 0.753 | cyclophilin |
| L2611 | JT954113.1 | 1.523 | 1.606 | conserved hypothetical protein |
| L0399 | JT928683.1 | 1.516 | 0.753 | mediator-associated protein 1-like |
| L0129 | JT946385.1 | 1.510 | 0.000 | hypothetical protein JCGZ_22853 |
| L0012 | JT960862.1 | 1.507 | 0.000 | protein ralf-like 34 |
| L0828 | JT959842.1 | 1.503 | 1.114 | tmv resistance protein n-like |
| L1909 | JT919017.1 | 0.666 | 1.114 | mannan endo-1, 4-beta-mannosidase 7-like |
| L0838 | JT916724.1 | 0.665 | 0.000 | autophagy-related protein 18f-like |
| L1049 | JT945364.1 | 0.665 | 1.606 | glutamine amidotransferase ylr126c |
| L0771 | JR350045.1 | 0.663 | 0.000 | RNA and export factor binding |
| L2768 | JT919210.1 | 0.655 | 0.000 | poly-a binding protein |
| L1355 | JR365416.1 | 0.653 | 0.000 | hypothetical protein JCGZ_08198 |
| L0104 | JT962900.1 | 0.638 | 0.000 | acyl-CoA-binding protein |
| L0792 | JT924112.1 | 0.637 | 0.000 | probable alpha-amylase 2 |
| L0346 | JT955693.1 | 0.632 | 0.000 | conserved hypothetical protein |
| L0334 | JT961724.1 | 0.630 | 0.000 | Ubiquinol-cytochrome c reductase complex 6.7 kDa protein |
| L1890 | JT933643.1 | 0.626 | 0.000 | hypothetical protein JCGZ_17861 |
| L0150 | JT957547.1 | 0.624 | 0.000 | iron-sulfur assembly mitochondrial |
| L0001 | JR366223.1 | 0.624 | 0.371 | C-repeat dehydration-responsive element-binding factor 4 |
| L0013 | JT916877.1 | 0.620 | 0.000 | enolase-phosphatase e1-like |
| L1607 | JT940720.1 | 0.619 | 0.000 | endonuclease or glycosyl hydrolase |
| L2244 | JR366661.1 | 0.613 | 0.000 | f-box protein skip19-like |
| L0417 | JT956666.1 | 0.611 | 0.000 | thioredoxin-like protein yls8 |
| L1067 | JR366204.1 | 0.610 | 0.000 | zinc finger CCCH domain-containing protein 13 |
| L0275 | JT926682.1 | 0.608 | 0.000 | transcription factor-related family protein* |
| L0784 | JT931101.1 | 0.600 | 0.000 | zinc finger CCCH domain-containing protein 11-like |
| L1391 | JT942502.1 | 0.600 | 0.000 | Transmembrane emp24 domain-containing protein 10 precursor |
| L2626 | JT944602.1 | 0.599 | 0.000 | 60S ribosomal protein L11 |
| L2735 | JT928745.1 | 0.588 | 1.114 | Ribosomal protein L16 |
| L1077 | JT946738.1 | 0.588 | 0.000 | conserved hypothetical protein |
| L1587 |  | 0.586 | 2.039 | wat1-related protein at2g39510-like |
| L0815 | JT943548.1 | 0.560 | 0.000 | DNAj chaperone c-terminal domain-containing family protein |
| L0491 | JT915443.1 | 0.546 | 0.000 | FACT complex subunit spt16-like |
| L2921 | JR366604.1 | 0.530 | 0.000 | annexin-like protein rj4 |
| L0799 | JT944013.1 | 0.515 | 0.000 | gibberellin receptor |
| L0896 | JT935611.1 | 0.515 | 0.000 | quinone-oxidoreductase chloroplastic |
| L0118 | JT949014.1 | 0.511 | 0.000 | cytochrome c oxidase subunit 5b-like |
| L2760 | JT935810.1 | 0.509 | 0.000 | hypothetical protein JCGZ_18332 |
| L0331 | JT942435.1 | 0.505 | 0.000 | calcium-binding ef hand family protein |
| L0960 | JT945149.1 | 0.477 | 0.000 | two-component response regulator arr5-like |
| L1853 | JT953751.1 | 0.469 | 0.000 | SAUR family protein (SAUR23) |
| L0060 | JT940201.1 | 0.447 | 0.000 | fasciclin-like arabinogalactan protein 7 |
| L1809 | JT941974.1 | 0.425 | 0.000 | formin-like protein 18 |
| L1645 | JT925274.1 | 0.413 | 0.000 | UDP-D-glucuronate 4-epimerase 6 |
| L2191 | JT929780.1 | 0.394 | 0.000 | probable wrky transcription factor 21 |
| L1227 | JR345627.1 | 0.268 | 0.000 | hypothetical protein JCGZ_08192 |

*The unigene was annotated by comparison with the NCBI Non-redundant protein sequences (nr) database (http://www.ncbi.nlm.nih.gov/) with the BlastN algorithm using an *E*-value cut-off of 10^−5^.
